# Organocatalytic enantioselective dearomatization of thiophenes by 1,10-conjugate addition of indole imine methides

**DOI:** 10.1038/s41467-021-25165-7

**Published:** 2021-08-12

**Authors:** Xingguang Li, Meng Duan, Peiyuan Yu, K. N. Houk, Jianwei Sun

**Affiliations:** 1 Department of Chemistry, the Hong Kong University of Science and Technology, Clear Water Bay, Kowloon, Hong Kong SAR, China; 2 The Hong Kong Branch of Chinese National Engineering Research Centre for Tissue Restoration & Reconstruction, Clear Water Bay, Kowloon, Hong Kong SAR, China; 3 Department of Chemistry and Biochemistry, University of California, Los Angeles, CA California, USA; 4 Department of Chemistry and Shenzhen Grubbs Institute, Guangdong Provincial Key Laboratory of Catalysis, Southern University of Science and Technology, Shenzhen, China

**Keywords:** Asymmetric catalysis, Synthetic chemistry methodology

## Abstract

Catalytic asymmetric dearomatization (CADA) is a powerful tool for the rapid construction of diverse chiral cyclic molecules from cheap and easily available arenes. This work reports an organocatalytic enantioselective dearomatization of substituted thiophenes in the context of a rare remote asymmetric 1,10-conjugate addition. By suitable stabilization of the thiophenyl carbocation with an indole motif in the form of indole imine methide, excellent remote chemo-, regio-, and stereocontrol in the nucleophilic addition can be achieved with chiral phosphoric acid catalysis under mild conditions. This protocol can be successfully extended to the asymmetric dearomatization of other heteroarenes including selenophenes and furans. Control experiments and DFT calculations demonstrate a possible pathway in which hydrogen bonding plays an important role in selectivity control.

## Abstract (web summary)

Catalytic asymmetric dearomatization is a powerful tool for the rapid construction of diverse chiral cyclic molecules from cheap and easily available arenes. Here the authors show an organocatalytic enantioselective dearomatization of substituted thiophenes in the context of a rare remote asymmetric 1,10-conjugate addition.

## Introduction

**Fig. 1:**
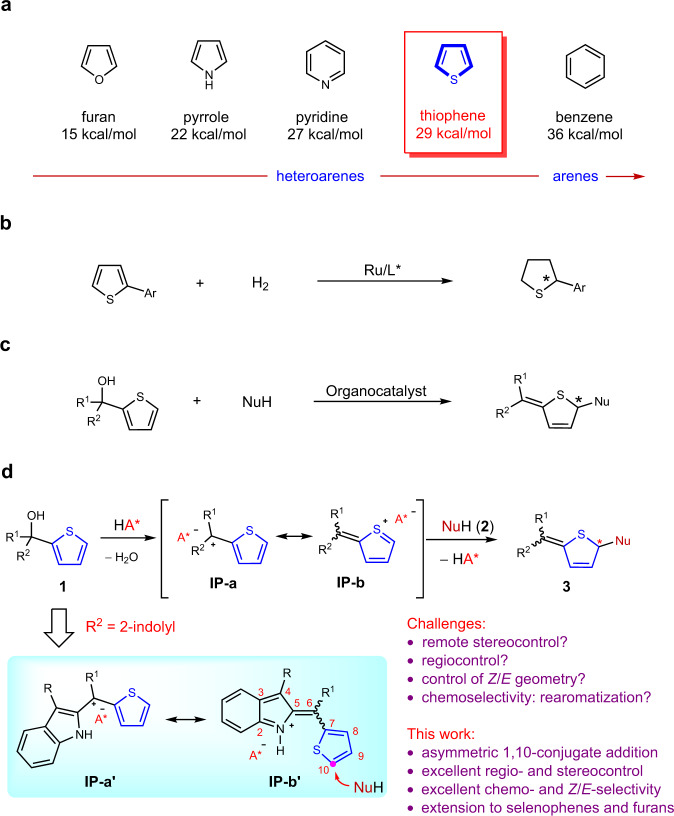
Asymmetric dearomatization of thiophenes. **a** Resonance stabilization of simple aromatic compounds. **b** Metal-catalyzed asymmetric dearomatization of thiophenes (by Glorius). **c** Organocatalytic asymmetric dearomatization of thiophenes (our strategy). **d** Our reaction design and the challenges (this work).

Catalytic asymmetric dearomatization (CADA) reactions have gained attention in the past few years since they offer direct and rapid access to enantioenriched functionalized ring systems and complex heterocyclic skeletons from simple and readily available arenes^1–7^. For example, CADA has been a pivotal step in the syntheses of many natural products and drugs^8–13^. Generally, thiophene is less prone to dearomative transformations than other common heteroarenes (e.g., furan, pyrrole, pyridine), owing to its relatively high resonance stabilization energy (Fig. 1a)^6,14^. Consequently, a high energy barrier is typically encountered, resulting in often harsh conditions for this type of transformations and imposing formidable challenges in controlling regioselectivity and stereoselectivity^15–18^. Beyond that, the strong coordination ability of the generated sulfur-containing product may deactivate the metal catalyst or interfere with stereocontrol by competing for binding, thus representing another important issue to address. These challenges have hampered the development of CADA of thiophenes. To the best of our knowledge, the only general example of this type was achieved by Glorius and coworkers via metal-catalyzed hydrogenation, leading to enantioenriched tetrahydrothiophenes (Fig. 1b)^19^. In contrast, the application of organocatalysis for such processes would be natural owning to the metal-free nature. However, challenges still remain in order to overcome the high barrier and achieve good stereocontrol^20^. Herein we report an organocatalytic approach (Fig. 1c).

As depicted in Fig. 1d, we hypothesized that, upon chiral acid activation, a 2-thiophenyl tertiary alcohol might generate a benzylic carbocation **IP-a**, with a chiral counter anion. The adjacent cation should activate the thiophene ring, for example, with charge delocalization represented in the resonance form **IP-b**. Subsequently, nucleophilic attack might take place in the fifth position of the thiophene ring. Furthermore, the chiral counter anion might induce asymmetric control in this step, leading to enantioenriched dearomatization product **3**. Notably, in addition to the inevitable remote enantiocontrol, this process will encounter other challenges, including regiocontrol on the nucleophilic sites and control over the double bond *Z/E*-configuration^18^. Product side reactions, such as rearomatization^21^, could provide chemoselectivity challenges. To address these potential problems, we envisioned that further stabilization of the positive charge by extended conjugation (e.g., quinone methide (QM) or indole imine methide) in a rigid framework might help selectivity control.

Indole-based imine methides have been demonstrated to be versatile intermediates in a range of organocatalytic asymmemtric transformations, particularly in 1,4- or 1,6-conjugate addition^22–29^ and cycloaddition^30–34^, leading to diverse enantioenriched indole derivatives^35–38^. Among them, we have reported an asymmetric 1,6-conjugate addition of such intermediates for the synthesis of chiral tetraarylmethanes containing an indole unit^29^. Recently, Antilla’s group and our group have developed remote asymmetric 1,8-addition to such species with chiral phosphoric acid (CPA) catalysis, providing efficient access to enantioenriched triarylmethanes and allenes, respectively^39,40^. Inspired by these studies and in continuation of our interests in remote stereocontrol, we envisioned the possibility of achieving further remote stereocontrol with this system. When the thiophene benzylic cation is adorned with a 2-indolyl group, this cation is stabilized in the form of highly conjugated indole imine methide **IP-b′** (Fig. 1d). Further nucleophilic conjugate addition is expected to take place at the tenth position based on our preliminary results, thereby representing not only thiophene dearomatization but also remote 1,10-conjugate addition. Notably, such remote stereocontrol has been rarely observed^41–43^. In addition, this dearomative 1,10-addition entails additional selectivity control, such as regioselectivity (1,6- vs. 1,10-addition), double bond *Z/E* selectivity, and axial chirality in some cases, none of which was a major problem in previous precedents of 1,6- and 1,8-addition of indole imine-methides. With the powerful bifunctional activation with CPA catalysis for remote control^44–55^, herein we have realized such an efficient asymmetric process.

## Results and discussion

### Reaction development

Our initial studies took advantage of QM intermediates for the asymmetric conjugate addition (see the SI for details)^50–55^. Unfortunately, these proved to be rather difficult to control regarding enantioselectivity and/or *Z/E* ratio. Subsequently, we employed indole-substituted tertiary alcohol **1a** as the substrate and 2-phenylpyrrole **2a** as the nucleophile (Table 1). With different CPA catalysts, the reaction in DCM proceeded successfully at room temperature to form the desired dearomatization product **3a** (entries 1–10). Among these catalysts, the [H_8_]BINOL-derived (*R*)-**B2** was identified as the best, resulting in 91% yield, 85% e.e., and >20:1 *E*/*Z* ratio. While comparable results could be obtained with catalysts **A3**, **A4**, and **B1** (entries 3–5), **B2** was used for further optimization on other parameters, which indicated that PhCl served as the superior solvent (entry 13). Finally, when the temperature was decreased to −40 ^o^C, **3a** was obtained in 99% yield, 96% e.e., and >20:1 *E*/*Z* (entry 14).

**Table 1:** Optimization of the reaction conditions^a^.


Entry	Catalyst	Solvent	Time (h)	Conv (%)	Yield (%)^b^	e.e. (%)^c^	*E*/*Z*
1	(*S*)-**A1**	DCM	0.5	98	88	−11	6:1
2	(*R*)-**A2**	DCM	0.5	100	80	34	18:1
3	(*R*)-**A3**	DCM	0.5	100	85	83	20:1
4	(*R*)-**A4**	DCM	0.5	100	92	79	>20:1
5	(*R*)-**B1**	DCM	0.5	98	94	84	>20:1
6	(*R*)-**B2**	DCM	0.5	93	91	85	>20:1
7	(*R*)-**C1**	DCM	0.5	100	82	−26	20:1
8	(*S*)-**C2**	DCM	0.5	100	82	72	18:1
9	(*R*)-**C3**	DCM	0.5	37	32	−77	6:1
10	(*S*)-**C4**	DCM	0.5	18	17	44	4:1
11	(*R*)-**B2**	DCE	1	87	88	76	>20:1
12	(*R*)-**B2**	EA	1	24	26	86	>20:1
13	(*R*)-**B2**	PhCl	1	100	99	89	>20:1
14^d^	(*R*)-**B2**	PhCl	6	100	99	96	>20:1

^a^Reaction conditions: **1** (0.20 mmol), **2** (0.24 mmol), catalyst (0.02 mmol), solvent (0.5 mL), RT. ^b^Determined by ^1^H NMR spectrum of the crude mixture using 1,3,5-^*i*^Pr_3_C_6_H_3_ as an internal standard. ^c^Determined by chiral HPLC.

^d^−40 ^o^C.

### Substrates scope exploration

Having identified the suitable directing group and optimal conditions, next we examined the substrate scope of this asymmetric process (Table 2). A wide range of 2-thiophenyl tertiary alcohols **1** and pyrroles **2** reacted to form the dearomatization products. In most cases, good yields *E*/*Z* ratios, and enantioselectivities were obtained. Electronically different phenyl substituents bearing various functional groups at the *ortho*, *meta,* and *para* positions showed good performance (**3a**–**h**, 87–97% yields, 92–99% e.e., and >20/1 *E*/*Z*). Moreover, polycyclic and heterocyclic aryl substituents were also well tolerated (**3i**–**k**). Notably, substrates **1j** and **1k** have two thiophenyl groups, but only one thiophene ring was dearomatized with high efficiency and chemoselectivity. Particularly, the case of **1k** also represented an efficient enantioselective desymmetrization. An alkyl-substituent could also be engaged in this process with excellent stereoselectivity, but in low yield. The low efficiency was caused by a competitive 1,6-addition pathway leading to chiral triarylethane **3l′** due to lower steric hindrance of this pathway (see the SI for details). Next, alcohols bearing differently-substituted indolyl groups were examined. All these reactions produced the desired products **3m**–**r** with satisfactory results, including the 3-unsubstituted indolyl one (**3r**). Substitution on the thiophene ring also led to the desired product **3s**, albeit with compromised efficiency and stereoselectivity. Finally, various pyrroles successfully served as nucleophiles, giving the corresponding enantioenriched sulfur heterocycles with respectable efficiency and selectivity (**3t**–**w**). Other electron-rich arenes, such as indole, naphthol, furan, thiophene, and 1,3,5-trimethoxybenzene, were also examined. Among them, indole reacted to form the desired product in good yield, but with a low *Z/E* selectivity and enantioselectivity. Other nucleophiles were not successful (see the SI for details).

**Table 2:**
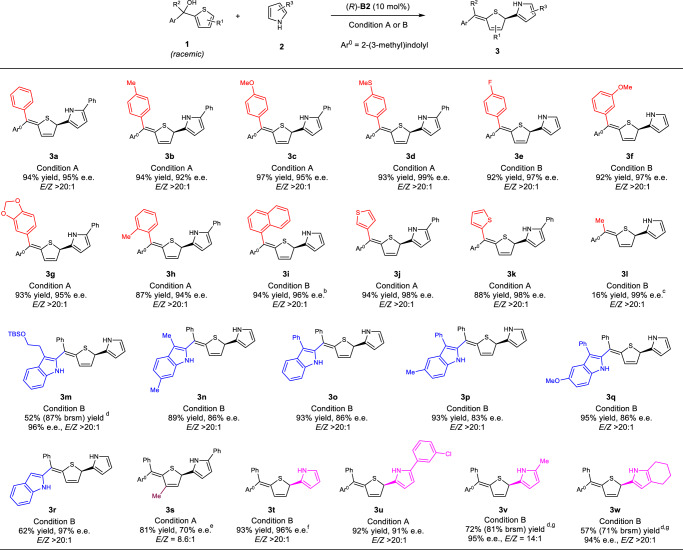
Scope study on asymmetric dearomatization of thiophenes^a^.

^a^Condition A: **1** (0.20 mmol), **2** (0.24 mmol), (*R*)-**B2** (10 mol%), PhCl (4 mL), −40 ^o^C, 12 h. Condition B: **1** (0.20 mmol), **2** (2 mmol), (*R*)-**B2** (10 mol%), toluene (4 mL), −20 ^o^C, 24 h. Isolated yields. The e.e. values were determined by chiral HPLC.

^b^30 h.

^c^−40 ^o^C.

^d^48 h, yield in parentheses is based on recovered starting material.

^e^−20 ^o^C, 48 h.

^f^Run in 0.4-mmol scale.

^g^Run with 2 equiv of **2**, 48 h.

We also applied this strategy to the asymmetric dearomatization of other heteroarenes, such as selenophenes (Table 3). After slight modification of the conditions (see the SI for details), the analogous selenophene substrates **4** reacted efficiently to form the corresponding chiral *Se*-heterocycles **5** with excellent stereoselectivity. The free alcohol functional group was also compatible with this catalytic system.

**Table 3:**
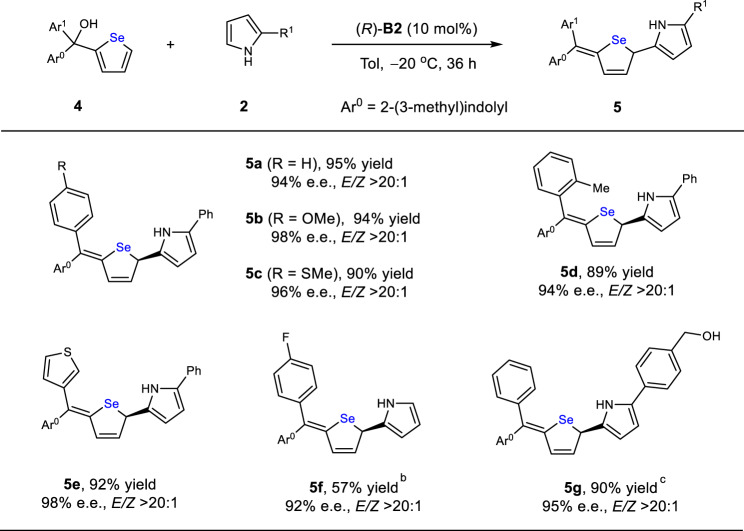
Enantioselective dearomatization of selenophenes^a^.

^a^**4** (0.20 mmol), **2** (0.24 mmol), (*R*)-**B2** (10 mol%), toluene (4 mL), 0 ^o^C, 36 h. Isolated yields. The e.e. values were determined by chiral HPLC.

^b^Run in 2-mmol scale, 24 h.

^c^Run with **4** (0.21 mmol) and **2** (0.20 mmol), 30 h.

### Mechanistic studies and synthetic applications

**Fig. 2:**
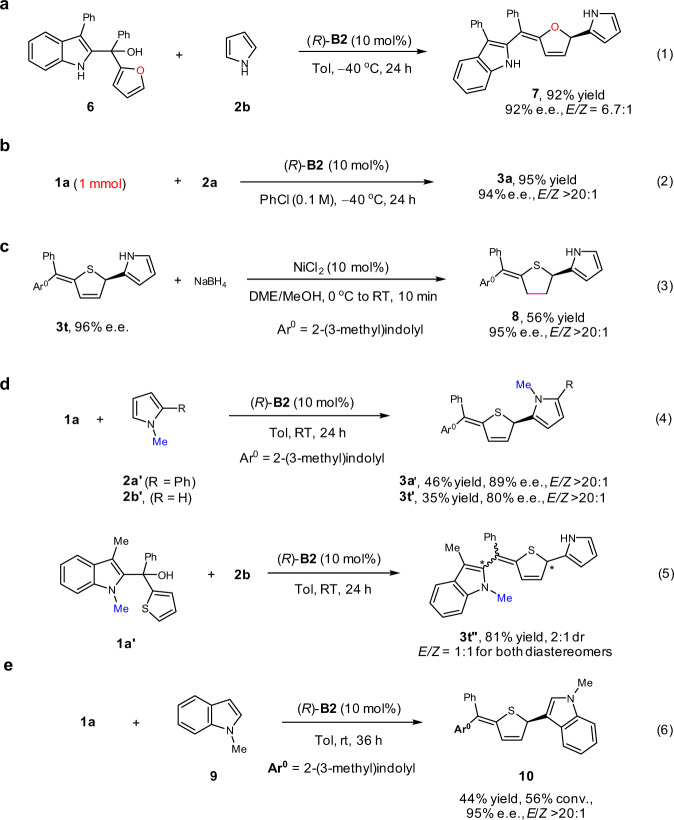
Scale-up synthesis, synthetic application, and control experiments. **a** Reaction extension to asymmetric dearomatization of the furan ring. **b** Mmol-scale synthesis. **c** Selective reduction of C-C double bond. **d** Control experiments with *N*-substituted substrates. **e** Reaction extension to *N*-methyl indole as the nucleophile.

This process could also be directly extended to the dearomatization of the furan ring. At −40 ^o^C, the reaction between furan carbinol **6** with pyrrole proceeded smoothly to form the desired product **7** in 92% yield with 92% e.e. and 6.7:1 *E*/*Z* ratio (Fig. 2, Eq. 1). The practicality was further demonstrated by a 1-mmol-scale reaction of **1a** and **2a**, resulting in comparably excellent results to the small-scale one (Eq. 2). Moreover, the double bond in the sulfur heterocycle of product **3t** could be further reduced to form enantioenriched tetrahydrothiophene **8** (Eq. 3). It is worth noting that enantioenriched tetrahydrothiophenes and tetrahydroselenophenes are useful molecules in organic synthesis and medicinal chemistry^56–62^. For example, they are known chiral ligands or organocatalysts for asymmetric synthesis ^56–59^.

Control experiments were carried out to gain some insights into the mechanism (see the SI for details). First, we examined the *N*-methylated pyrroles **2a′** and **2b′**, which are more nucleophilic than **2a** and **2b**, respectively. However, their reactivity was found to be lower, requiring a much higher temperature (RT vs. −40 ^o^C under the standard conditions). The corresponding products **3a′** and **3t′** were obtained in only moderate yields with slightly decreased enantioselectivities (Eq. 4). The results indicated that hydrogen bonding with *N*−H motif of the nucleophile is not necessary for good stereocontrol, but this interaction might help reduce the barrier of the nucleophilic addition. In contrast, *N*-methylation in the substrate indole motif (**1a′**) led to dramatic changes in both reactivity and stereoselectivity. At room temperature, the reaction with **2b** produced a mixture of diastereomers in 2:1 dr and 1:1 *E*/*Z* (Eq. 5). The presence of so many isomers made the enantioselectivity determination difficult. This observation strongly suggested that the *N*−H motif in the indole unit plays a crucial role in stereocontrol, which is likely to facilitate the key imine methide intermediate generation and allow subsequent hydrogen-bonding interaction with the catalyst. Notably, other nucleophiles were also examined (Supplementary Table 12), and it was found that *N*-methyl indole performed well in this system to furnish the desired product **10** in moderate yield and excellent stereoselectivity (44% yield, 95% e.e., *E/Z* >20:1, Eq. 6).

### Density functional theory studies

**Fig. 3:**
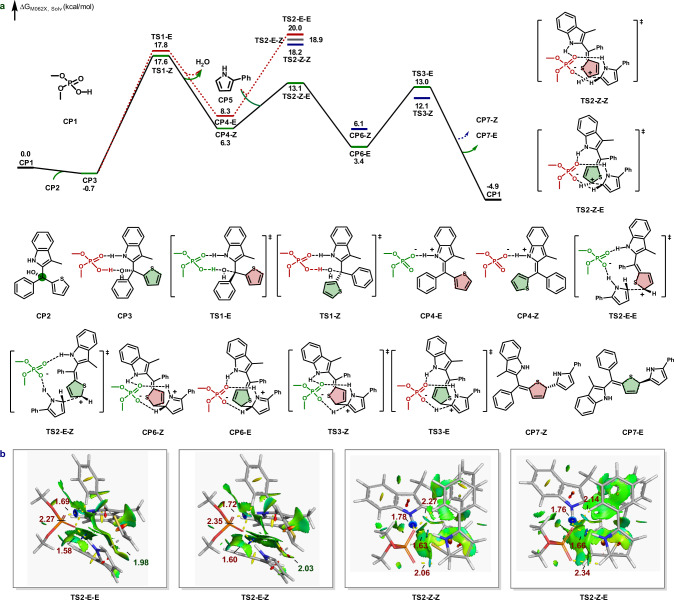
DFT calculations for the enantioselective dearomatization of thiophenes. **a** Free energy profile of dimethyl phosphoric acid-catalyzed dearomatization of thiophenes. **b** Color-filled NCI isosurfaces of the transition state **TS2-E-E**, **TS2-E-Z**, **TS2-Z-Z**, and **TS2-Z-E** (blue, strong attraction; green, weak interaction; red, steric effect). The distances are given in Ångstroms.

**Fig. 4:**
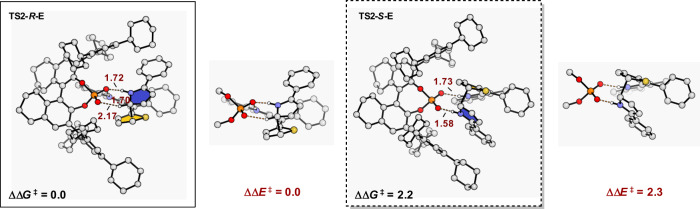
DFT-optimized chiral phosphoric acid catalyzed transition states TS2-R-E and TS2-S-E. The distances are given in Ångstroms, and energies are given in kcal/mol.

To better understand the mechanism and origins of selectivity, density functional theory (DFT) calculations were conducted on the reaction of tertiary alcohol **1a** and 2-phenylpyrrole **2a** by dimethyl phosphoric acid (Fig. 3) or chiral phosphoric acid (*R*)-**B2** (Fig. 4) using Gaussian 16^63^. Geometry optimizations were performed at B3LYP-D3BJ/6-31 G(d) the level of theory^64,65^, and single-point energies were computed with M06-2X/6-311+G(d,p)-CPCM(chlorobenzene)^66–69^. The calculated potential energy profile is given in Fig. 3. The binding of substrate **CP2** to catalyst **CP1** affords relatively stable complex **CP3**. Then intermediate **CP3** undergoes acid-catalyzed dehydration via transition states **TS1-E** and **TS1-Z** to obtain the imine methide intermediates **CP4-E** and **CP4-Z**, with activation free energies of 18.5 and 18.3 kcal/mol, respectively. Subsequently, the nucleophilic attack of 2-phenylpyrrole **CP5** to the *Z*-isomer **CP4-Z** via **TS2-Z-Z** and **TS2-Z-E** lead to **CP6-Z** and **CP6-E**. The difference in energy between these two transition states is 5.1 kcal/mol, showing great stereoselectivity (>20/1, *E*/*Z*). The subsequent intermolecular hydrogen shift forms the final product **CP7** and regenerates the free catalyst via **TS3-E** and **TS3-Z**. However, the formation of the preferred product **CP7-E** through the nucleophile 2-phenylpyrrole **CP5** attacks the *E*-isomer **CP4-E** via **TS2-E-E** and **TS2-E-Z** is very difficult, and has an overall barrier of 20.7 and 19.6 kcal/mol with respect to the stable intermediate **CP3**, respectively. The enantio-determining step for this reaction is the nucleophilic attack of 2-phenylpyrrole on the indole imine methide **CP4-Z**, **TS2**.

We first investigated the dearomative transformations from different configurations of imine methide **CP4**. As depicted in Fig. 3a, the transition states **TS2-E-E** and **TS2-E-Z** from the *E*-isomer are higher in energy than **TS2-Z-E** by 6.9 and 5.8 kcal/mol, respectively. To gain further insight into the energy difference, the non-covalent interaction (NCI) analysis was performed^70,71^. Figure 3b shows color-filled NCI isosurfaces for all non-covalent interactions in the transition states. The crucial difference between these competing transition structures is the location of the phenyl group of thiophene, which is orientated toward the catalyst in unfavored **TS2-E-E** and **TS2-E-Z**. To accommodate the phenyl group, it is hard for transition states **TS2-E-E** and **TS2-E-Z** to form additional stabilizing hydrogen-bonding interactions. In contrast, **TS2-Z-E** enjoys additional favorable C–H···O–P interaction (shown in light blue). Therefore, *E*-isomer **CP4-E** is less prone to dearomative transformations than *Z*-isomer **CP4-Z**, basically owing to the weaker hydrogen-bonding interactions.

To elucidate the origins of stereoselectivity, we explored the structures of dimethyl phosphoric acid-catalyzed transition states **TS2-Z-Z** and **TS2-Z-E**, which generate *Z-*selectivity product **CP7-*Z*** and *E-*selectivity product **CP7-*E***, respectively. The 5.1 kcal/mol difference in energy between **TS2-Z-Z** and **TS2-Z-E** corresponds to the excellent stereoselectivity (>20:1 ratio). The primary difference between these two transitions states (Fig. 3b) is the orientation of the electron-deficient sulfur center of the thiophene relative to the electron-rich pyrrole ring. In minor **TS2-Z-Z**, the electron-deficient sulfur center is far away from the electron-rich pyrrole ring, while it points toward the center of the electron-rich pyrrole ring in major **TS2-Z-E** (shown in light blue), suggesting the presence of attractive chalcogen-bonding interaction in **TS2-Z-E**^72,73^. In addition, the π–π interactions between the phenyl rings in **TS2-Z-Z** are weaker than those in **TS2-Z-E** (shown as large green disks). Therefore, the favorable chalcogen-bonding and π–π interactions are the main contributions to the 5.1 kcal/mol preference for forming an *E*-selectivity product.

We also studied chiral phosphoric acid (*R*)**-B2** catalyzed enantioselective addition of the 2-phenylpyrrole to the imine methide. The calculated *si*-face attack in **TS2-*R*-E** is found to be 2.2 kcal/mol lower in free energy than the re-face attack in **TS2-*S*-E**, which is consistent with the 96% e.e. observed experimentally. To better recognize the factors that impact the enantioselectivity, optimized structures of transition states **TS2-*R*-E** and **TS2-*S*-E** were compared (Fig. 4). Apparently, transition state **TS2-*R*-E** enjoys additional stabilizing C–H···O–P interaction compared to **TS2-*S*-E**. Such favorable hydrogen-bonding interactions decrease the energies of transition state **TS2-*R*-E**. In contrast, there is a lack of C–H···O–P interaction in unfavored **TS2-*S*-E**. Further calculations by removing the substituents of the catalyst with methyl groups, then computed the single-point ΔΔE^‡^ without optimization, show a 2.3 kcal/mol advance for the transition state **TS2-*R*-E**. The energetics provide reasonable agreement with experimental observations. Consequently, hydrogen-bonding interactions play a leading role in determining high enantioselectivity.

In summary, we have developed an efficient organocatalytic enantioselective dearomatization of thiophenes in the context of asymmetric 1,10-conjugate addition. It is also a rare example of excellent remote stereocontrol. By suitable stabilization of 2-thiophenyl carbocation with an indole motif in the form of an extended indole imine methide, chiral phosphoric acid serves as a superior bifunctional catalyst to promote intermolecular C−C bond formation with excellent chemo-, regio- and enantioselectivity as well as the product double bond *Z/E* ratio. This protocol can be extended to the asymmetric dearomatization of selenophenes and furans. Control experiments and DFT calculations illustrated a possible pathway in which multiple hydrogen-bonding interactions play a crucial role in achieving excellent stereocontrol.

## Methods

### General procedure for the catalytic asymmetric dearomatization of thiophenes

#### Condition **A**

At −40 ^o^C, to an oven-dried 8-mL vial charged with a solution of the tertiary alcohol **1** (0.2 mmol) and pyrrole **2** (0.24 mmol) in PhCl (3.6 mL) was slowly added a solution of catalyst (*R*)-**B2** (20 mg, 0.015 mmol, 10 mol%) in PhCl (0.4 mL). The reaction mixture was stirred at the same temperature for 12 h. After that, triethylamine (two drops) was added to quench the reaction. The mixture was concentrated under reduced pressure and purified by silica gel (deactivated by triethylamine) flash chromatography to afford the desired product **3**.

#### Condition B

At −20 ^o^C, to an oven-dried 8-mL vial charged with a solution of the tertiary alcohol **1** (0.2 mmol) and pyrrole **2** (2.0 mmol) in toluene (3.6 mL) was slowly added a solution of catalyst (*R*)-**B2** (20 mg, 0.015 mmol, 10 mol%) in toluene (0.4 mL). The reaction mixture was stirred at the same temperature for 24 h. After that, triethylamine (two drops) was added to quench the reaction. The mixture was concentrated under reduced pressure and purified by silica gel (deactivated by triethylamine) flash chromatography to afford the desired product **3**.

## Supplementary information

Supplementary Information

## Data Availability

All data generated and analyzed during this study are included in this article and its Supplementary Information, or also available from the authors upon reasonable request. The X-ray crystallographic coordinate for structure **5a** has been deposited at the Cambridge Crystallographic Data Centre under deposition numbers CCDC 2022226, respectively, and can be obtained free of charge from the CCDC via http://www.ccdc.cam.ac.uk/data_request/cif.
