# Supplementary material for: Organocatalytic enantioselective dearomatization of thiophenes by 1,10-conjugate addition of indole imine methides
Source: Nat Commun. 2021 Aug 12;12:4881. doi: 10.1038/s41467-021-25165-7 (PMC8361129; doi:10.1038/s41467-021-25165-7)
Supplement: Supplementary file 1 — Supplementary Information [file 41467_2021_25165_MOESM1_ESM.pdf]

Supplementary Information

Organocatalytic Enantioselective Dearomatization of Thiophenes by 1,10-Conjugate Addition of Indole Imine Methides

Xingguang Li†,[1,4] Meng Duan†,[2,3] Peiyuan Yu,[3] K. N. Houk,*[2] Jianwei Sun*[1,4]

[1] Department of Chemistry, The Hong Kong University of Science and Technology, Clear Water Bay, Kowloon, Hong Kong SAR, China

[2] Department of Chemistry and Biochemistry, University of California, Los Angeles, California 90095, United States

[3] Department of Chemistry and Shenzhen Grubbs Institute, Guangdong Provincial Key Laboratory of Catalysis, Southern University of Science and Technology, Shenzhen, 518055, China

[4] The Hong Kong Branch of Chinese National Engineering Research Centre for Tissue Restoration & Reconstruction, Clear Water Bay, Kowloon, Hong Kong SAR, China

*E-mail: houk@ucla.edu; sunjw@ust.hk

Table of Contents

Supplementary Methods **S-3**

I. General Information **S-3**

II. Synthesis of the Tertiary Alcohol Substrates **S-4**

III. Condition Optimization **S-22**

IV. Dearomatization of Thiophenes and Selenophenes **S-33**

V. Scale-up Reaction and Application **S-60**

VI. Mechanistic Experiments **S-62**

VII. DFTCalculations **S-69**

VIII. Determination of the Absolute Stereochemistry **S-109**

Supplementary References **S-115**

NMR Spectra and HPLC Traces

Supplementary Methods

I. General Information

Flash column chromatography was performed over silica gel (200-300 mesh) purchased from Qindao Puke Co., China. All air or moisture sensitive reactions were conducted in oven-dried glassware under nitrogen atmosphere using anhydrous solvents. Anhydrous dichloromethane, toluene, diethyl ether, and tetrahydrofuran were purified by the Innovative® solvent purification system. Chemicals were purchased from commercial suppliers and used without further purification unless otherwise stated. ^{1}H, ^{13}C, ^{19}F NMR spectra were collected on a Bruker AV 400 MHz NMR spectrometer using residue solvent peaks as an internal standard (^{1}H NMR: $CDCl_3$ at 7.26 ppm, CD_2Cl_2 at 5.32 ppm, acetone-d_6 at 2.05 ppm; ^{13}C NMR: $CDCl_3$ at 77.16 ppm, CD_2Cl_2 at 53.84 ppm, acetone-d_6 at 29.84 ppm). Data for ^{1}H NMR are recorded as follows: chemical shift (δ, ppm), multiplicity (s = singlet; d = doublet; t = triplet; q = quarter; p = pentet; sept = septet; m = multiplet; br = broad), coupling constant (Hz), integration. Mass spectra were collected on an Agilent GC/MS 5975C system, a MALDI Micro MX mass spectrometer, or an API QSTAR XL System. IR spectra were recorded on Bruker TENSOR 27 spectrometer and reported in terms of frequency of absorption (cm^{-1}). Optical rotations were measured on JASCO P-2000 polarimeter with $[\alpha]^D$ values reported in degrees; concentration (c) is in 10 mg/mL. The enantiomeric excess values were determined by chiral HPLC using an Agilent 1200 LC instrument with Daicel CHIRALPAK® AD-H, IC, AS-H, or CHIRALCEL® OD-H columns.

II. Synthesis of the Tertiary Alcohol Substrates

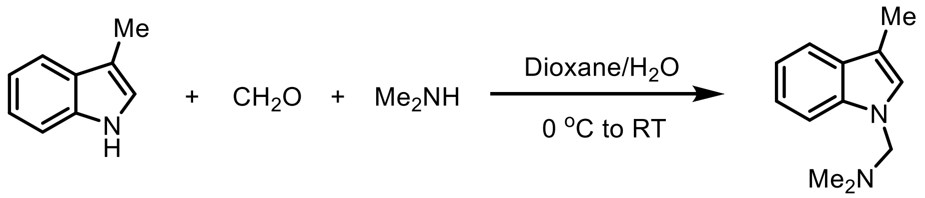

S1

3-Methyl-1-[(*N*,*N*-dimethylamino)methyl]indole (S1). To a suspension of 3-methyl-indole (13.12 g, 100 mmol) in water/dioxane (1:1, 50 mL) was added formaldehyde (7.5 mL, 37% aqueous solution) and dimethylamine (15 mL, 33% aqueous solution) over 30 min at 0 °C. The mixture was stirred for 1 hour, then warmed to room temperature overnight. The solution was extracted twice with diethyl ether (2 x 30 mL), and the organic phases were combined and extracted with an aqueous HCl solution (2 N, 2 x 50 mL). The acid extracts were made alkaline with aqueous NaOH solution (40 wt%, 30 mL), then extracted with diethyl ether (2 x 50 mL), dried over Na_2SO_4, filtered, concentrated in vacuo to give **S1** as a light yellow oil (15.0 g, 80% yield) which was directly used in the next step. The NMR spectra are consistent with reported literature.[1]

General Procedure A.

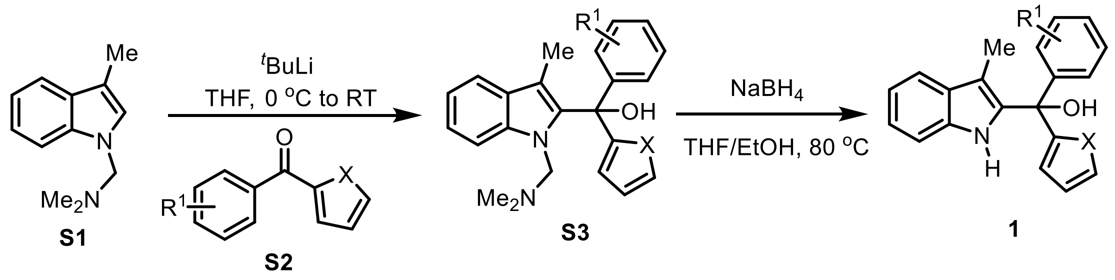

At 0 °C, to a stirred solution of **S1** (0.56 g, 3 mmol) in THF (30 mL) was slowly added *tert*-butyllithium (1.6 M in pentane, 2.1 mL, 3.3 mmol). The resulting mixture was kept stirring at 0 °C for 30 min. Next, a solution of **S2**[2,3] (3 mmol) in THF (10 mL) was added and the reaction mixture was kept stirring overnight. A saturated aqueous solution of NH_4Cl was added to quench the reaction. The reaction mixture was extracted with EtOAc (3 × 30 mL). The combined organic layers were dried over anhydrous Na_2SO_4, filtered, and concentrated. The residue was purified by silica gel flash chromatography to afford the crude tertiary alcohol **S3**.

To the solution of **S3** in the THF/EtOH (1:1, 2 mL/mmol), $NaBH_4$ (1.5 equiv.) was added. The resulting mixture was kept refluxing overnight for 12 h. After cooling to room temperature, saturated aqueous solution of NH_4Cl was slowly added to quench the reaction. The reaction mixture was extracted with EtOAc (2 × 30 mL). The combined organic layers were dried over anhydrous Na_2SO_4, filtered, and concentrated. The residue was purified by silica gel flash chromatography to afford the pure tertiary alcohol **1**.

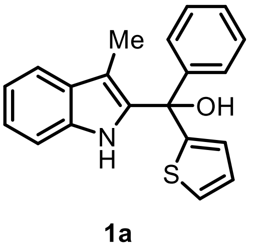

1a

(3-Methyl-*1H*-indol-2-yl)(phenyl)(thiophen-2-yl)methanol (1a) was prepared as slightly yellow foam from phenyl(thiophen-2-yl)methanone (5 mmol) according to the General Procedure A (eluent: hexanes/EtOAc = 10:1 → 5:1) in 85% yield (2 steps, 1.36 g).

^{1}H NMR (400 MHz, acetone-*d_6*) δ 9.73 (s, 1H), 7.52-7.49 (m, 3H), 7.42-7.28 (m, 5H), 7.11-7.07 (m, 1H), 7.03-6.97 (m, 2H), 6.88 (dd, J_1 = 0.9 Hz, J_2 = 3.6 Hz, 1H), 5.78 (s,

1H), 1.88 (s, 3H).

^{13}C NMR (100 MHz, acetone-d_6) δ 152.6, 146.9, 139.5, 135.7, 130.7, 128.7, 128.4, 127.9, 127.2, 127.1, 126.4, 122.2, 119.4, 119.0, 112.0, 108.0, 77.2, 9.6.

IR (thin film) 3522, 3447, 3055, 1694, 1451, 1258, 1136, 733, 698 cm^{-1}.

HRMS (CI+) Calcd for $C_{20}H_{17}NOS$ (M^+): 319.1031, Found: 319.1038.

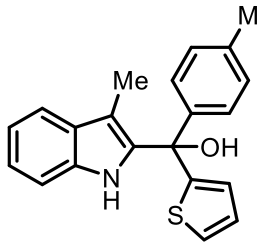

1b

(3-Methyl-*1H*-indol-2-yl)(thiophen-2-yl)(*p*-tolyl)methanol (1b) was prepared as yellowish foam from (*p*-tolyl)(thiophen-2-yl)methanone (3 mmol) according to the General Procedure A (eluent: hexanes/EtOAc = 10:1 → 5:1) in 32% yield (2 steps, 0.324 g).

^{1}H NMR (400 MHz, acetone-d_6) *δ* 9.73 (s, 1H), 7.52 (d, *J* = 7.8 Hz, 1H), 7.43-7.38 (m, 4H), 7.18 (d, *J* = 8.2 Hz, 2H), 7.14-6.92 (m, 4H), 5.73 (s, 1H), 2.34 (s, 3H), 1.94 (s, 3H)

^{13}C NMR (100 MHz, acetone-d_6) *δ* 152.7, 143.9, 139.6, 137.8, 135.6, 130.7, 129.2, 127.9, 127.1, 126.9, 126.2, 122.1, 119.3, 119.0, 111.9, 107.8, 77.0, 21.1, 9.7.

IR (thin film) 3572, 3450, 3051, 1456, 1264, 1186, 995, 732, 704 cm^{-1}.

HRMS (CI+) Calcd for $C_{21}H_{19}NOS$ (M^+): 333.1187, Found: 333.1199.

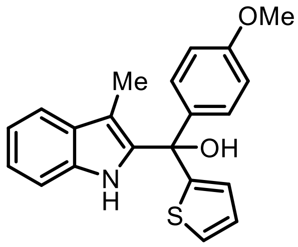

1c

(4-Methoxyphenyl)(3-methyl-1*H*-indol-2-yl)(thiophen-2-yl)methanol (1c) was prepared as slightly green-yellow foam from (4-methoxyphenyl)

(thiophen-2-yl)methanone (2 mmol) according to the General Procedure A (eluent: hexanes/EtOAc = 10:1 → 5:1) in 46% yield (2 steps, 0.370 g).

^{1}H NMR (400 MHz, acetone-*d$_6$*) δ 9.71 (s, 1H), 7.48 (d, J = 7.9 Hz, 1H), 7.40-7.37 (m, 4H), 7.11-7.06 (m, 1H), 7.03-6.96 (m, 2H), 6.91-6.88 (m, 3H), 5.66 (s, 1H), 3.79 (s, 3H), 1.89 (s, 3H).

^{13}C NMR (100 MHz, acetone-*d$_6$*) δ 160.0, 153.0, 139.8, 139.0, 135.6, 130.7, 129.3, 127.1, 126.9, 126.2, 122.1, 119.3, 119.0, 113.9, 111.9, 107.7, 76.9, 55.5, 9.7.

IR (thin film) 3510, 3446, 3052, 2999, 1695, 1607, 1507, 1246, 1174, 1123, 1028, 831, 733, 701 cm^{-1}.

HRMS (CI+) Calcd for $C_{21}H_{19}NO_2S$ (M^+): 349.1136, Found: 349.1144.

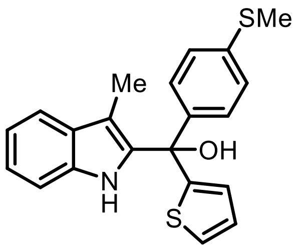

1d

(3-Methyl-1*H*-indol-2-yl)(4-(methylthio)phenyl)(thiophen-2-yl)methanol (1d) was prepared as pale yellow foam from (4-(methylthio)phenyl)(thiophen-2-yl)methanone (3 mmol) according to the General Procedure A (eluent: hexanes/EtOAc = 10:1 → 5:1) in 76% yield (2 steps, 0.830 g).

^{1}H NMR (400 MHz, acetone-*d$_6$*) δ 9.72 (s, 1H), 7.49-7.37 (m, 5H), 7.24 (d, J = 8.4 Hz, 2H), 7.10-6.89 (m, 4H), 5.77 (s, 1H), 2.48 (s, 3H), 1.90 (s, 3H).

^{13}C NMR (100 MHz, acetone-*d$_6$*) δ 152.5, 143.6, 139.4, 138.9, 135.7, 130.7, 128.5, 127.2, 127.0, 126.4, 126.2, 122.2, 119.4, 119.0, 112.0, 108.0, 76.9, 15.3, 9.73.

IR (thin film) 3534, 3445, 3051, 2919, 1699, 1488, 1302, 1264, 994, 819, 732, 702 cm^{-1}.

HRMS (CI+) Calcd for $C_{21}H_{19}NOS_2$ (M^+): 365.0908, Found: 365.0901.

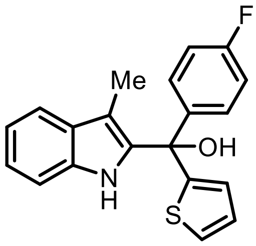

1e

(4-Fluorophenyl)(3-methyl-1*H*-indol-2-yl)(thiophen-2-yl)methanol (1e) was prepared as viscous yellow oil from (4-fluorophenyl)(thiophen-2-yl)methanone (2.5 mmol) according to the General Procedure A (eluent: hexanes/EtOAc = 10:1 → 5:1) in 41% yield (2 steps, 0.350 g).

^{1}H NMR (400 MHz, acetone-*d$_6$*) δ 9.76 (s, 1H), 7.55-7.38 (m, 5H), 7.14-6.89 (m, 6H), 5.89 (s, 1H), 1.89 (s, 3H).

^{13}C NMR (100 MHz, acetone-*d$_6$*) δ 162.9 (d, J = 243.2 Hz), 152.4, 143.1 (d, J = 3.3 Hz), 139.3, 135.7, 130.6, 130.0 (d, J = 8.1 Hz), 127.3, 127.1, 126.5, 122.3, 119.4, 119.1, 115.2 (d, J = 21.3 Hz), 112.0, 108.0, 76.8, 9.6.

^{19}F NMR (376 MHz, acetone-*d$_6$*) δ -116.6.

IR (thin film) 3520, 3438, 3060, 2989, 1698, 1605, 1502, 1227, 1152, 999, 833, 746 cm^{-1}.

HRMS (CI+) Calcd for $C_{20}H_{16}FNOS$, (M^+): 337.0937, Found: 337.0939.

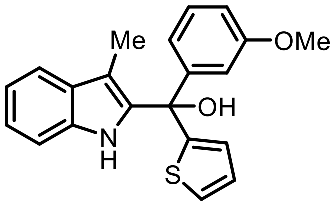

1f

(3-Methoxyphenyl)(3-methyl-1*H*-indol-2-yl)(thiophen-2-yl)methanol (1f) was prepared as slightly yellowish oil from (3-methoxyphenyl)(thiophen-2-yl)methanone (3 mmol) according to the General Procedure A (eluent: hexanes/EtOAc = 10:1 → 5:1) in 81% yield (2 steps, 0.850 g).

^{1}H NMR (400 MHz, acetone-*d$_6$*) δ 9.71 (s, 1H), 7.50-748 (m, 1H), 7.42-7.37 (m, 2H), 7.26 (t, J = 8.0 Hz, 1H), 7.15-6.91 (m, 5H), 6.92-6.86 (m, 2H), 5.78 (s, 1H), 3.73 (s, 3H), 1.92 (s, 3H).

^{13}C NMR (100 MHz, acetone-*d$_6$*) δ 160.3, 152.5, 148.5, 139.4, 135.7, 130.7, 129.7,

127.11, 127.07, 126.4, 122.2, 120.3, 119.4, 119.0, 114.0, 113.4, 112.0, 108.0, 77.1, 55.4, 9.7.

IR (thin film) 3363, 3056, 2998, 1701, 1590, 1441, 1243, 1143, 1036, 739 cm^{-1}.

HRMS (CI+) Calcd for $C_{21}H_{19}NO_2S$ (M^+): 349.1136, Found: 349.1131.

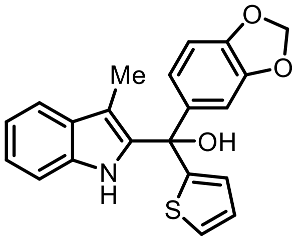

1g

Benzo[*d*][1,3]dioxol-5-yl(3-methyl-1*H*-indol-2-yl)(thiophen-2-yl)methanol (1g) was prepared as slightly yellow gum-like liquid from benzo[*d*][1,3]dioxol-5-yl(thiophen-2-yl)methanone (3 mmol) according to the General Procedure A (eluent: hexanes/EtOAc = 10:1 → 5:1) in 75% yield (2 steps, 0.820 g).

^{1}H NMR (400 MHz, acetone-d_6) δ 9.70 (s, 1H), 7.52 (d, J = 7.9 Hz, 1H), 7.42-7.39 (m, 2H), 7.14-6.95 (m, 6H), 6.82 (d, J = 8.2 Hz, 1H), 5.98 (d, J = 0.9 Hz, 2H), 5.74 (s, 1H), 1.97 (s, 3H).

^{13}C NMR (100 MHz, acetone-d_6) δ 152.6, 148.3, 147.8, 141.0, 139.4, 135.6, 130.6, 127.1, 127.0, 126.3, 122.2, 121.5, 119.4, 119.0, 111.9, 108.6, 108.0, 107.9, 102.0, 77.0, 9.7.

IR (thin film) 3522, 3445, 3054, 2980, 1487, 1237, 1089, 932, 858, 731 cm^{-1}.

HRMS (CI+) Calcd for $C_{21}H_{17}NO_3S$ (M^+): 363.0929, Found: 363.0935.

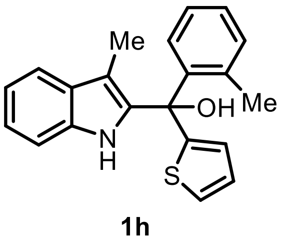

1h

(3-Methyl-1*H*-indol-2-yl)(thiophen-2-yl)(*o*-tolyl)methanol (1h) was prepared as pale yellow foam from (*o*-tolyl)(thiophen-2-yl)methanone (3 mmol) according to the General Procedure A (eluent: hexanes/EtOAc = 10:1 → 5:1) in 64% yield (2 steps,

0.640 g).

^{1}H NMR (400 MHz, CD_2Cl_2) δ 8.38 (s, 1H), 7.54 (d, J = 7.9 Hz, 1H), 7.38 (dd, J_1 = 5.1 Hz, J_2 = 1.2 Hz, 1H), 7.31-7.23 (m, 3H), 7.19-7.08 (m, 3H), 7.03-6.98 (m, 2H), 6.83 (dd, J_1 = 3.6 Hz, J_2 = 1.2 Hz, 1H), 3.38 (s, 1H), 2.20 (s, 3H), 1.86 (s, 3H).

^{13}C NMR (100 MHz, CD_2Cl_2) δ 150.9, 142.7, 138.2, 137.6, 134.6, 132.9, 130.2, 129.0, 128.8, 127.2, 126.7, 126.2, 125.8, 122.4, 119.5, 119.0, 111.2, 108.2, 78.5, 21.3, 9.0.

IR (thin film) 3531, 3448, 3054, 2919, 1455, 1265, 986, 838, 733, 702 cm^{-1}.

HRMS (CI+) Calcd for $C_{21}H_{19}NOS$ (M^+): 333.1187, Found: 333.1192.

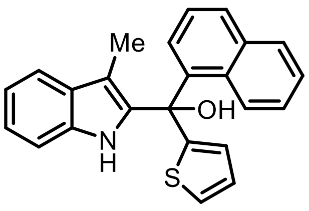

1i

(3-Methyl-1*H*-indol-2-yl)(naphthalen-1-yl)(thiophen-2-yl)methanol (1i) was prepared as white solid from naphthalen-1-yl(thiophen-2-yl)methanone (3 mmol) according to the General Procedure A (eluent: hexanes/EtOAc = 10:1 → 5:1) in 36% yield (2 steps, 0.398 g).

^{1}H NMR (400 MHz, acetone-d_6) δ 9.98 (s, 1H), 8.43 (d, J = 8.8 Hz, 1H), 7.91 (d, J = 8.2 Hz, 2H), 7.48-7.36 (m, 5H), 7.24-7.20 (m, 2H), 7.15-7.11 (m, 1H), 7.06-7.02 (m, 3H), 6.13 (s, 1H), 1.68 (s, 3H).

^{13}C NMR (100 MHz, acetone-d_6) δ 153.1, 142.0, 139.7, 135.8, 135.6, 132.6, 130.7, 130.3, 129.2, 128.3, 127.5, 127.4, 126.7, 126.2, 126.1, 125.8, 125.2, 122.2, 119.4, 119.0, 112.0, 107.3, 78.6, 9.1.

IR (thin film) 3528, 3435, 3049, 2921, 1699, 1601, 1449, 1315, 1231, 1134, 980, 726 cm^{-1}.

HRMS (CI+) Calcd for $C_{24}H_{19}NOS$ (M^+): 369.1187, Found: 369.1172.

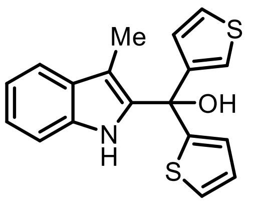

1j

(3-Methyl-1*H*-indol-2-yl)(thiophen-2-yl)(thiophen-3-yl)methanol (1j) was prepared as slightly greenish gum-like liquid from thiophen-2-yl(thiophen-3-yl)methanone (3 mmol) according to the General Procedure A (eluent: hexanes/EtOAc = 10:1 → 5:1) in 54% yield (2 steps, 0.530 g).

^{1}H NMR (400 MHz, acetone-d_6) δ 9.78 (s, 1H), 7.50 (d, J = 7.9 Hz, 1H), 7.42-7.38 (m, 3H), 6.82 (dd, J_1 = 1.3 Hz, J_2 = 3.0 Hz, 1H), 7.19 (dd, J_1 = 1.3 Hz, J_2 = 5.1 Hz, 1H), 7.12-6.94 (m, 4H), 5.83 (s, 1H), 1.94 (s, 3H).

^{13}C NMR (100 MHz, acetone-d_6) δ 152.3, 148.6, 139.3, 135.5, 130.7, 128.4, 127.0, 126.5, 126.4, 126.2, 123.6, 122.2, 119.3, 119.0, 112.0, 107.3, 74.8, 9.4.

IR (thin film) 3438, 3101, 2976, 1699, 1514, 1415, 1228, 1128, 1003, 839, 732, 701 cm^{-1}.

HRMS (CI+) Calcd for $C_{18}H_{15}NOS_2$ (M^+): 325.0595, Found: 325.0609.

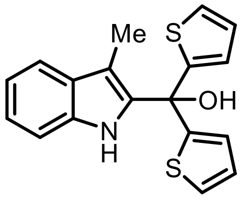

1k

(3-Methyl-1*H*-indol-2-yl)di(thiophen-2-yl)methanol (1k) was prepared as slightly green foam from di(thiophen-2-yl)methanone (3 mmol) according to the General Procedure A (eluent: hexanes/EtOAc = 10:1 → 5:1) in 32% yield (2 steps, 0.310 g).

^{1}H NMR (400 MHz, acetone-d_6) δ 9.86 (s, 1H), 7.47 (d, J = 7.8 Hz, 1H), 7.42-7.39 (m, 3H), 7.08 (t, J = 7.1 Hz, 1H), 7.02-6.97 (m, 5H), 6.06 (s, 1H), 1.95 (s, 3H).

^{13}C NMR (100 MHz, acetone-d_6) δ 152.1, 139.4, 135.5, 130.7, 127.1, 126.8, 126.5, 122.4, 119.4, 119.1, 112.0, 107.7, 74.8, 9.7.

IR (thin film) 3528, 3443, 3096, 2918, 1691, 1456, 1324, 1301, 1233, 1115, 979, 838, 733, 700 cm^{-1}.

HRMS (CI+) Calcd for $C_{18}H_{15}NOS_2$ (M^+): 325.0595, Found: 325.0585.

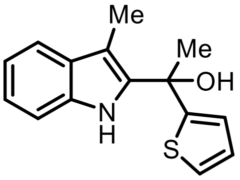

1l

1-(3-Methyl-1*H*-indol-2-yl)-1-(thiophen-2-yl)ethan-1-ol (1l) was prepared as white solid from 1-(thiophen-2-yl)ethan-1-one (2 mmol) according to the General Procedure A (eluent: hexanes/EtOAc = 10:1 → 5:1) in 76% yield (2 steps, 0.392 g).

^{1}H NMR (400 MHz, acetone-d_6) δ 9.88 (s, 1H), 7.48 (d, J = 7.8 Hz, 1H), 7.41 (d, J = 8.0 Hz, 1H), 7.33-7.31 (m, 1H), 7.10-7.06 (m, 3H), 6.93 (dd, J_1 = 3.6 Hz, J_2 = 5.0 Hz, 1H), 5.22 (s, 1H), 2.15 (s, 3H), 2.12 (s, 3H).

^{13}C NMR (100 MHz, acetone-d_6) δ 153.8, 140.5, 135.3, 130.9, 127.0, 125.6, 124.5, 121.7, 119.2, 118.7, 111.9, 105.6, 72.1, 31.1, 9.5.

IR (thin film) 3518, 3433, 3062, 2997, 1451, 1312, 1228, 901, 738, 698 cm^{-1}.

HRMS (CI+) Calcd for $C_{15}H_{15}NOS$ (M^+): 257.0874, Found: 257.0876.

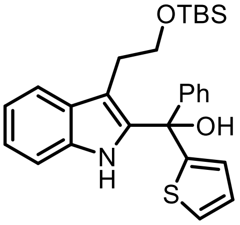

1m

(3-(2-((*tert*-Butyldimethylsilyl)oxy)ethyl)-1*H*-indol-2-yl)(phenyl)(thiophen-2-yl)methanol (1m) was prepared as colorless oil from 1-(3-(2-((*tert*-butyldimethylsilyl)oxy)ethyl)-1*H*-indol-1-yl)-*N*,*N*-dimethylmethanamine (3 mmol) and phenyl(thiophen-2-yl)methanone (3 mmol) according to the General Procedure A (eluent: hexanes/EtOAc = 10:1 → 5:1) in 66% yield (2 steps, 0.920 g).

^{1}H NMR (400 MHz, acetone-d_6) δ 9.42 (s, 1H), 7.58-7.54 (m, 3H), 7.40-7.28 (m, 5H), 7.13-7.03 (m, 2H), 6.97 (dd, J_1 = 3.6 Hz, J_2 = 5.1 Hz, 1H), 6.86 (dd, J_1 = 1.2 Hz, J_2 = 3.6 Hz, 1H), 6.12 (s, 1H), 3.82 (t, J = 6.5 Hz, 2H), 2.97-2.78 (m, 2H), 0.86 (s, 9H), 0.01 (s, 6H).

^{13}C NMR (100 MHz, acetone-d_6) δ 152.9, 147.5, 141.2, 135.7, 129.7, 128.6, 128.2,

127.5, 127.2, 126.7, 126.2, 122.3, 119.7, 119.1, 112.3, 109.6, 77.0, 64.1, 28.5, 26.4, 19.0, -5.2.

IR (thin film) 3443, 3340, 3066, 1448, 1342, 1253, 1070, 898, 828, 732, 700 cm^{-1}.

HRMS (CI+) Calcd for $C_{27}H_{33}NO_2SSi$ (M^+): 463.2001, Found: 463.1992.

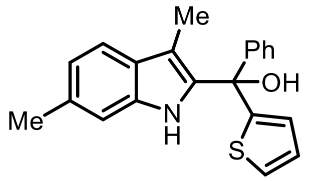

1n

(3,6-Dimethyl-1*H*-indol-2-yl)(phenyl)(thiophen-2-yl)methanol (1n) was prepared as viscous brown oil from 1-(3,6-dimethyl-1*H*-indol-1-yl)- *N*,*N*-dimethylmethanamine (3 mmol) and phenyl(thiophen-2-yl)methanone (3 mmol) according to the General Procedure A (eluent: hexanes/EtOAc = 10:1 → 5:1) in 39% yield (2 steps, 0.387 g).

^{1}H NMR (400 MHz, acetone-d_6) δ 9.52 (s, 1H), 7.52-7.50 (m, 2H), 7.40-7.30 (m, 5H), 7.20 (s, 1H), 6.97 (dd, J_1 = 3.6 Hz, J_2 = 5.1 Hz, 1H), 6.89-6.87 (m, 2H), 5.73 (s, 1H), 2.41 (s, 3H), 1.86 (s, 3H).

^{13}C NMR (100 MHz, acetone-d_6) δ 152.7, 147.0, 138.8, 136.1, 131.5, 128.7, 128.6, 128.3, 127.9, 127.1, 127.0, 126.4, 121.3, 118.8, 111.9, 107.9, 77.2, 21.9, 9.7.

IR (thin film) 3521, 3439, 3026, 2919, 1625, 1450, 1323, 1230, 994, 749, 702 cm^{-1}.

HRMS (CI+) Calcd for $C_{21}H_{19}NOS$ (M^+): 333.1187, Found: 333.1175.

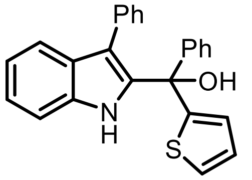

1o

Phenyl(3-phenyl-1*H*-indol-2-yl)(thiophen-2-yl)methanol (1o) was prepared as pale white solid from *N*,*N*-dimethyl-1-(3-phenyl-1*H*-indol-1-yl) methanamine (3 mmol) and phenyl(thiophen-2-yl)methanone (3 mmol) according to the General Procedure A (eluent: hexanes/EtOAc = 10:1 → 5:1) in 54% yield (2 steps, 0.615 g).

^{1}H NMR (400 MHz, acetone-d_6) δ 9.98 (s, 1H), 7.50-7.44 (m, 3H), 7.35 (d, J = 8.0

Hz, 1H), 7.28-7.10 (m, 10H), 7.05-7.02 (m, 1H), 6.81-6.79 (m, 1H), 6.76 (d, *J* = 3.5 Hz, 1H), 5.55 (s, 1H).

^{13}C NMR (100 MHz, acetone-*d$_6$*) *δ* 152.1, 146.9, 139.1, 136.2, 135.6, 131.3, 130.3, 128.4, 128.3, 128.2, 128.1, 127.5, 126.9, 126.4, 126.1, 122.8, 120.2, 119.9, 115.7, 112.2, 77.8.

IR (thin film) 3517, 3430, 3044, 1599, 1481, 1328, 1241, 1025, 837, 936, 696 cm^{-1}.

HRMS (CI+) Calcd for $C_{25}H_{19}NOS$ (M^+): 381.1187, Found: 381.1176.

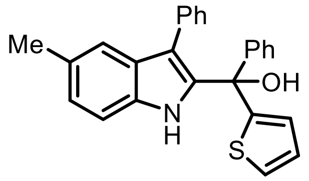

1p

(5-Methyl-3-phenyl-1*H*-indol-2-yl)(phenyl)(thiophen-2-yl)methanol (1p) was prepared as pale white solid from *N,N*-dimethyl-1-(5-methyl-3-phenyl-1*H*-indol-1-yl)methanamine (3 mmol) and phenyl(thiophen-2-yl) methanone (3 mmol) according to the General Procedure A (eluent: hexanes/EtOAc = 10:1 → 5:1) in 46% yield (2 steps, 0.513 g).

^{1}H NMR (400 MHz, acetone-*d$_6$*) *δ* 9.88 (s, 1H), 7.42-7.39 (m, 2H), 7.35 (d, *J* = 8.3 Hz, 1H), 7.27 (dd, J_1 = 1.2 Hz, J_2 = 5.1 Hz, 1H), 7.24-7.19 (m, 3H), 7.13-7.06 (m, 6H), 6.98 (dd, J_1 = 1.3 Hz, J_2 = 8.3 Hz, 1H), 6.78 (dd, J_1 = 3.6 Hz, J_2 = 5.1 Hz, 1H), 6.71 (dd, J_1 = 1.2 Hz, J_2 = 3.6 Hz, 1H), 5.56 (s, 1H), 2.36 (s, 3H).

^{13}C NMR (100 MHz, acetone-*d$_6$*) *δ* 152.3, 147.0, 139.2, 136.5, 134.0, 131.4, 130.5, 129.1, 128.4, 128.3, 128.17, 128.16, 127.5, 126.9, 126.4, 126.1, 124.4, 119.4, 115.2, 112.0, 77.8, 21.6.

IR (thin film) 3515, 3434, 3045, 2920, 1600, 1480, 1270, 1027, 751, 699 cm^{-1}.

HRMS (CI+) Calcd for $C_{26}H_{21}NOS_2$ (M^+): 395.1344, Found: 395.1351.

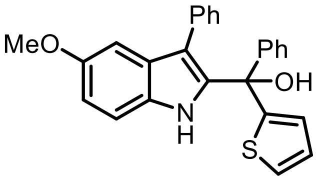

1q

(5-Methoxy-3-phenyl-1*H*-indol-2-yl)(phenyl)(thiophen-2-yl)methanol (1q) was prepared as pale white solid from 1-(5-methoxy-3-phenyl-1*H*-indol-1-yl)-*N*,*N*-dimethylmethanamine (3 mmol) and phenyl(thiophen-2-yl) methanone (3 mmol) according to the General Procedure A (eluent: hexanes/EtOAc = 10:1 → 5:1) in 20% yield (2 steps, 0.167 g).

^{1}H NMR (400 MHz, acetone-d_6) δ 9.86 (s, 1H), 7.42-7.39 (m, 2H), 7.36 (dd, J_1 = 1.0 Hz, J_2 = 5.1 Hz, 1H), 7.28 (dd, J_1 = 1.2 Hz, J_2 = 5.1 Hz, 1H), 7.23-7.19 (m, 3H), 7.13-7.08 (m, 5H), 6.82-6.78 (m, 3H), 6.71 (dd, J_1 = 1.2 Hz, J_2 = 3.6 Hz, 1H), 5.55 (s, 1H), 3.69 (s, 3H).

^{13}C NMR (100 MHz, acetone-d_6) δ 155.3, 152.3, 147.0, 139.9, 136.4, 131.3, 130.7, 130.5, 129.5, 128.4, 128.3, 128.1, 127.5, 126.9, 126.4, 126.1, 115.5, 113.2, 113.0, 101.3, 77.8, 55.8.

IR (thin film) 3538, 3424, 3055, 2933, 1604, 1481, 1444, 1268, 1141, 836, 745, 698 cm^{-1}.

HRMS (CI+) Calcd for $C_{26}H_{21}NO_2S$ (M^+): 411.1293, Found: 411.1297.

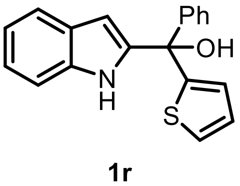

1r

(1*H*-Indol-2-yl)(phenyl)(thiophen-2-yl)methanol (1r) At -78 °C, to a stirred solution of thiophene (0.277 g, 3.3 mmol) in THF (15 mL) was slowly added *n*-butyllithium (2.4 M in hexane, 1.4 mL, 3.3 mmol). The resulting mixture was then kept stirring at -20 °C for 1 h. Next, the mixture was slowly added to the solution of (1*H*-indol-2-yl)(phenyl)methanone (0.243 g, 1.1 mmol) in THF (10 mL) at -20 ºC. After that, the reaction mixture was warmed up and kept stirring overnight. A saturated aqueous solution of NH_4Cl was added to quench the reaction. The reaction mixture was extracted with EtOAc (3 × 30 mL). The combined organic layers were

dried over anhydrous Na_2SO_4, filtered, and concentrated. The residue was purified by silica gel flash chromatography to afford the product **1r** as yellow-green foam (eluent: hexanes/EtOAc = 10:1 → 5:1) in 95% yield (0.320 g).

^{1}H NMR (400 MHz, acetone-d_6) δ 10.08 (s, 1H), 7.52-7.48 (m, 3H), 7.39 (d, J = 6.5 Hz, 2H), 7.35-7.27 (m, 3H), 7.10-7.06 (m, 1H), 7.00-6.96 (m, 2H), 6.88 (d, J = 3.4 Hz, 1H), 6.16 (s, 1H), 5.81 (s, 1H).

^{13}C NMR (100 MHz, acetone-d_6) δ 152.7, 147.0, 144.8, 137.6, 128.5, 128.2, 127.6, 127.0, 126.8, 126.1, 122.4, 121.1, 120.0, 112.14, 112.09, 102.5, 77.0.

IR (thin film) 3520, 3410, 3051, 1442, 1282, 1133, 1133 1015, 733, 695 cm^{-1}.

HRMS (CI+) Calcd for $C_{19}H_{15}NOS$ (M^+): 305.0874, Found: 305.0881.

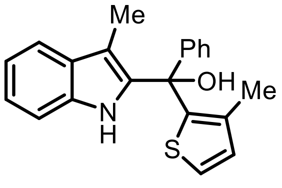

1s

(3-Methyl-1*H*-indol-2-yl)(3-methylthiophen-2-yl)(phenyl)methanol (1s) was prepared as slightly yellowish foam from *N*,*N*-dimethyl-1-(3-methyl-1*H*-indol-1-yl)methanamine (3 mmol) and (3-methylthiophen-2-yl)(phenyl)methanone (3 mmol) according to the General Procedure A (eluent: hexanes/EtOAc = 10:1 → 5:1) in 73% yield (2 steps, 0.730 g).

^{1}H NMR (400 MHz, acetone-d_6) δ 9.70 (s, 1H), 7.57-7.50 (m, 3H), 7.40 (d, J = 8.0 Hz, 1H), 7.37-7.29 (m, 3H), 7.20 (d, J = 5.1 Hz, 1H), 7.12-7.01 (m, 2H), 6.89 (d, J = 5.1 Hz, 1H), 5.60 (s, 1H), 2.01 (s, 3H), 1.92 (s, 3H).

^{13}C NMR (100 MHz, acetone-d_6) δ 146.7, 144.1, 138.6, 136.7, 135.7, 132.5, 130.6, 128.5, 128.3, 128.0, 124.0, 122.1, 119.3, 119.0, 112.0, 107.9, 76.7, 15.3, 9.3.

IR (thin film) 3529, 3438, 3052, 2921, 1611, 1450, 1319, 1270, 981, 745 cm^{-1}.

HRMS (CI+) Calcd for $C_{21}H_{19}NOS$ (M^+): 333.1187, Found: 333.1189.

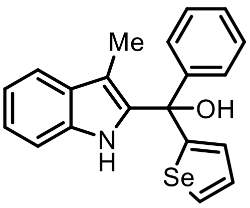

4a

(3-Methyl-1*H*-indol-2-yl)(phenyl)(selenophen-2-yl)methanol (4a) was prepared as slightly yellow foam from phenyl(selenophen-2-yl)methanone (3 mmol) according to the General Procedure A (eluent: hexanes/EtOAc = 10:1 → 5:1) in 81% yield (2 steps, 0.89 g).

^{1}H NMR (400 MHz, acetone-d_6) δ 9.70 (s, 1H), 8.15-8.01 (m, 1H), 7.53-7.45 (m, 3H), 7.38-7.30 (m, 4H), 7.20 (dd, J_1 = 3.8 Hz, J_2 = 5.6 Hz, 1H), 7.09-7.05 (m, 1H), 7.01-6.98 (m, 2H), 5.82 (s, 1H), 1.89 (s, 3H).

^{13}C NMR (100 MHz, acetone-d_6) δ 160.3, 147.1, 139.6, 135.7, 131.8, 130.7, 129.8, 128.7, 128.7, 128.3, 127.9, 122.2, 119.4, 119.0, 112.0, 107.9, 78.7, 9.8.

IR (thin film) 3568, 3447, 3053, 1486, 1451, 1325, 1264, 1232, 994, 842, 731, 694 cm^{-1}.

HRMS (CI+) Calcd for $C_{20}H_{17}NOSe$ (M^+): 367.0475, Found: 367.0464.

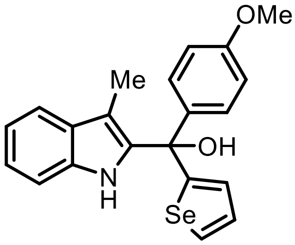

4b

(4-Methoxyphenyl)(3-methyl-1*H*-indol-2-yl)(selenophen-2-yl)methanol (4b) was prepared as slightly yellow foam from (4-methoxyphenyl)(selenophen-2-yl)methanone (3 mmol) according to the General Procedure A (eluent: hexanes/EtOAc = 10:1 → 5:1) in 68% yield (2 steps, 0.81 g).

^{1}H NMR (400 MHz, acetone-d_6) δ 9.70 (s, 1H), 8.12-8.00 (m, 1H), 7.49-7.38 (m, 4H), 7.21-7.00 (m, 4H), 6.89 (d, J = 8.8 Hz, 2H), 5.74 (s, 1H), 3.78 (s, 3H), 1.93 (s, 3H).

^{13}C NMR (100 MHz, acetone-d_6) δ 160.7, 160.0, 139.8, 139.1, 135.6, 131.6, 130.7, 129.7, 129.3, 128.4, 122.1, 119.3, 119.0, 113.9, 112.0, 107.7, 78.4, 55.5, 9.9.

IR (thin film) 3446, 3051, 2921, 1607, 1506, 1454, 1300, 1251, 1175, 1028, 988, 831,

732, 694 cm^{-1}.

HRMS (CI+) Calcd for $C_{21}H_{19}NO_2Se$ (M^+): 397.0581, Found: 397.0578.

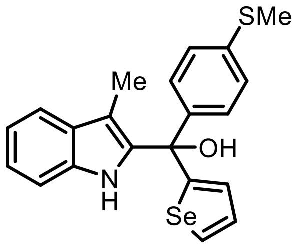

4c

(3-Methyl-1*H*-indol-2-yl)(4-(methylthio)phenyl)(selenophen-2-yl)methanol (4c) was prepared as slightly yellow foam from (4-(methylthio)phenyl)(selenophen-2-yl)methanone (3 mmol) according to the General Procedure A (eluent: hexanes/EtOAc = 10:1 → 5:1) in 67% yield (2 steps, 0.83 g).

^{1}H NMR (400 MHz, acetone-*d_6*) δ 9.71 (s, 1H), 8.13-8.00 (m, 1H), 7.49-7.45 (m, 3H), 7.39 (d, J = 8.0 Hz, 1H), 7.25-7.19 (m, 3H), 7.11-7.00 (m, 3H), 5.83 (s, 1H), 2.48 (s, 3H), 1.95 (s, 3H).

^{13}C NMR (100 MHz, acetone-*d_6*) δ 160.2, 143.8, 139.4, 138.9, 135.6, 131.8, 130.7, 129.8, 128.6, 128.5, 126.2, 122.2, 119.4, 119.0, 112.0, 107.9, 78.4, 15.24, 9.9.

IR (thin film) 3514, 3441, 3050, 2918, 1693, 1487, 1450, 1325, 1260, 1121, 1001, 821, 732, 694 cm^{-1}.

HRMS (CI+) Calcd for $C_{21}H_{19}NOSSe$ (M^+): 413.0353, Found: 413.0363.

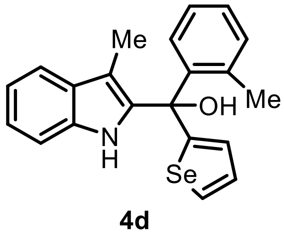

4d

(3-Methyl-1*H*-indol-2-yl)(selenophen-2-yl)(*o*-tolyl)methanol (4d) was prepared as slightly yellow foam from (*o*-tolyl)(selenophen-2-yl)methanone (2.5 mmol) according to the General Procedure A (eluent: hexanes/EtOAc = 10:1 → 5:1) in 65% yield (2 steps, 0.62 g).

^{1}H NMR (400 MHz, acetone-d_6) δ 9.79 (s, 1H), 8.09-8.07 (m, 1H), 7.47 (d, J = 7.8 Hz, 1H), 7.41 (d, J = 8.0 Hz, 1H), 7.25-7.18 (m, 3H), 7.12-7.00 (m, 5H), 5.75 (s, 1H), 2.20 (s, 3H), 1.81 (s, 3H).

^{13}C NMR (100 MHz, acetone-d_6) δ 160.7, 144.1, 139.6, 139.1, 135.6, 132.9, 131.6, 130.7, 130.0, 129.2, 128.9, 128.2, 125.8, 122.1, 119.4, 119.0, 112.0, 106.6, 79.6, 21.5, 9.3.

IR (thin film) 3528, 3442, 3053, 2972, 1453, 1323, 1300, 1265, 1137, 984, 842, 735, 693 cm^{-1}.

HRMS (CI+) Calcd for $C_{21}H_{19}NOSe$ (M^+): 381.0632, Found: 381.0631.

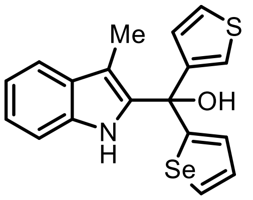

4e

(3-Methyl-1*H*-indol-2-yl)(selenophen-2-yl)(thiophen-3-yl)methanol (4e) was prepared as slightly green liquid from selenophen-2-yl(thiophen-3-yl) methanone (2.5 mmol) according to the General Procedure A (eluent: hexanes/EtOAc = 10:1 → 5:1) in 60% yield (2 steps, 0.56 g).

^{1}H NMR (400 MHz, acetone-d_6) δ 9.77 (s, 1H), 8.06-8.05 (m, 1H), 7.49 (d, J = 7.9 Hz, 1H), 7.42-7.37 (m, 2H), 7.27-7.00 (m, 6H), 5.87 (s, 1H), 1.96 (s, 3H).

^{13}C NMR (100 MHz, acetone-d_6) δ 159.9, 148.7, 139.4, 135.5, 131.6, 130.7, 129.6, 128.4, 128.1, 126.5, 123.7, 122.2, 119.3, 119.0, 112.0, 107.3, 76.2, 9.6.

IR (thin film) 3437, 3050, 2918, 1691, 1452, 1327, 1228, 1002, 840, 734, 691 cm^{-1}.

HRMS (CI+) Calcd for $C_{18}H_{15}NOSSe$ (M^+): 373.0040, Found: 373.0042.

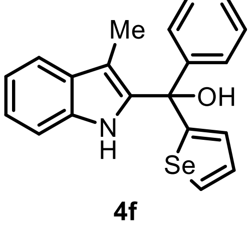

4f

(4-Fluorophenyl)(3-methyl-1*H*-indol-2-yl)(selenophen-2-yl)methanol (4f) was prepared as slightly green viscous oil from (4-fluorophenyl)(selenophen-2-yl)methanone (2.5 mmol) according to the General Procedure A (eluent: hexanes/EtOAc = 10:1 → 5:1) in 48% yield (2 steps, 0.460 g).

^{1}H NMR (400 MHz, acetone-*d$_6$*) δ 9.75 (s, 1H), 8.09-8.07 (m, 1H), 7.58-7.55 (m, 2H), 7.50 (d, J = 7.8 Hz, 1H), 7.39 (d, J = 8.0 Hz, 1H), 7.22-7.01 (m, 6H), 5.95 (s, 1H), 1.94 (s, 3H).

^{13}C NMR (100 MHz, acetone-*d$_6$*) δ 162.9 (d, J = 243.0 Hz), 160.1, 143.3 (d, J = 2.9 Hz), 139.3, 135.6, 131.9, 130.6, 130.0 (d, J = 8.3 Hz), 129.9, 128.7, 122.3, 119.4, 119.1, 115.3 (d, J = 21.3 Hz), 112.0, 108.0, 78.2, 9.9.

^{19}F NMR (376 MHz, acetone-*d$_6$*) δ -116.6.

IR (thin film) 3523, 3440, 3054, 2999, 1600, 1502, 1452, 1320, 1227, 996, 832, 751, 694 cm^{-1}.

HRMS (CI+) Calcd for $C_{20}H_{16}FNOSe$ (M^+): 385.0381, Found: 385.0392.

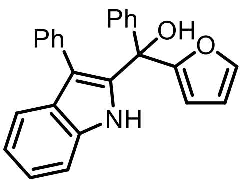

6

Furan-2-yl(phenyl)(3-phenyl-1*H*-indol-2-yl)methanol (6) At -78 °C, to a stirred solution of furan (0.204 g, 3 mmol) in THF (15 mL) was slowly added *tert*-butyllithium (1.3 M in pentane, 2.3 mL, 3 mmol). The resulting mixture was slowly warmed up to 0 °C and kept stirring at the same temperature for 1 h. Next, a solution of phenyl(3-phenyl-1*H*-indol-2-yl)methanone (0.357 g, 1.2 mmol) in THF (5 mL) was slowly added to the reaction mixture at 0 ºC. After that, the reaction mixture was warmed up to room temperature and kept stirring overnight. A saturated aqueous solution of NH_4Cl was added to quench the reaction. The reaction mixture was extracted with EtOAc (3 × 30 mL). The combined organic layers were dried over anhydrous Na_2SO_4, filtered, and concentrated. The residue was purified by silica gel flash chromatography to afford the product as yellowish foam (eluent: hexanes/EtOAc = 30:1 → 20:1) in 94% yield (0.410 g).

^{1}H NMR (400 MHz, acetone-*d$_6$*) δ 10.01 (s, 1H), 7.49 (d, J = 8.1 Hz, 1H), 7.43-7.40

(m, 2H), 7.32-7.21 (m, 5H), 7.17-7.10 (m, 6H), 7.03-6.99 (m, 1H), 6.16 (dd, J_1 = 1.8 Hz, J_2 = 3.2 Hz, 1H), 5.91 (d, J = 3.2 Hz, 1H), 5.61 (s, 1H).

^{13}C NMR (100 MHz, acetone-d_6) δ 157.7, 145.0, 143.1, 137.8, 136.0, 135.8, 131.2, 130.2, 128.35, 128.30, 128.2, 128.0, 126.4, 122.7, 120.1, 119.8, 115.8, 112.1, 110.7, 110.1, 75.3.

IR (thin film) 3536, 3432, 3050, 1602, 1489, 1444, 1321, 1259, 1007, 741, 697 cm^{-1}.

HRMS (CI+) Calcd for $C_{25}H_{19}NO_2$ (M^+): 365.1416, Found: 365.1401.

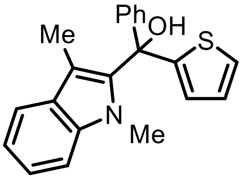

1a'

(1,3-Dimethyl-1*H*-indol-2-yl)(phenyl)(thiophen-2-yl)methanol (1a') At -78 °C, to a stirred solution of 1,3-dimethyl-1*H*-indole (0.363 g, 2.5 mmol) in THF (20 mL) was slowly added *tert*-butyllithium (1.3 M in pentane, 1.9 mL, 2.5 mmol). The resulting mixture was slowly warmed up to 0 °C and kept stirring at the same temperature for 1 h. Next, a solution of phenyl(thiophen-2-yl)methanone (0.470 g, 2.5 mmol) in THF (5 mL) was slowly added to the reaction mixture at 0 ºC. After that, the reaction mixture was warmed up to room temperature and kept stirring overnight. A saturated aqueous solution of NH_4Cl was added to quench the reaction. The reaction mixture was extracted with EtOAc (3 × 30 mL). The combined organic layers were dried over anhydrous Na_2SO_4, filtered, and concentrated. The residue was purified by silica gel flash chromatography to afford the product as slightly purple foam (eluent: hexanes/EtOAc = 40:1 → 20:1) in 41% yield (0.340 g).

^{1}H NMR (400 MHz, acetone-d_6) δ 7.50 (d, J = 7.8 Hz, 1H), 7.46-7.44 (m, 3H), 7.40-7.33 (m, 3H), 7.28 (d, J = 8.3 Hz, 1H), 7.20-7.16 (m, 1H), 7.06-7.02 (m, 1H), 6.98 (dd, J_1 = 3.6 Hz, J_2 = 5.1 Hz, 1H), 6.78 (dd, J_1 = 1.2 Hz, J_2 = 3.6 Hz, 1H), 5.99 (s, 1H), 3.57 (s, 3H), 1.57 (s, 3H).

^{13}C NMR (100 MHz, acetone-d_6) δ 153.5, 147.2, 138.41, 138.38, 129.4, 128.9, 128.4, 127.6, 127.3, 127.2, 126.6, 122.9, 119.4, 119.3, 110.3, 109.6, 77.7, 32.8, 9.1.

IR (thin film) 3520, 3050, 1602, 1465, 1353, 1238, 1142, 996, 839, 733, 704 cm^{-1}.

HRMS (CI+) Calcd for $C_{21}H_{19}NOS$ (M^+): 333.1187, Found: 333.1187.

III. Condition Optimization

Supplementary Table 1. Initial condition study with *para*-quinone methide as intermediate[a]

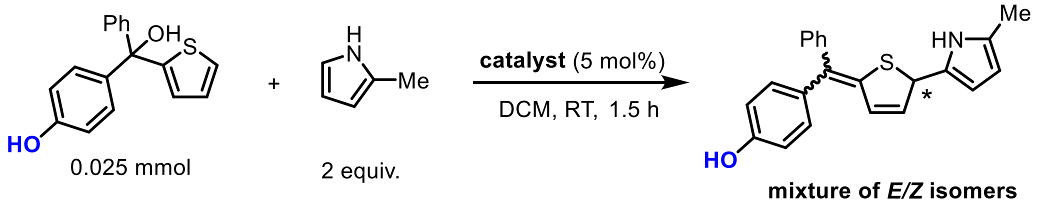

Entry	Catalyst	Conv.(%)	Major		Minor		Ratio
			yield(%)	ee(%)	yield(%)	ee(%)	
1	(*R*)-BINOL-TRIP-OH	>95	47	7	23	22	2:1
2	(*R*)-8H-BINOL-TRIP-OH	94	46	10	25	23	1.8:1
3	(*R*)-SPINOL-TRIP-OH	39	12	7	9	11	1.3:1
4	(*R*)-BINOL-TRIP-NHTf	84	47	1	28	5	1.7:1
5	(*rac*-)-BINOL-H-OH	100	rt	-	-	-	-

[a] Reaction conditions: tertiary alcohol (0.025 mmol), 2-methylpyrrole (2 equiv.), catalyst (5 mol%), DCM (0.5 mL). Yield is based on analysis of the ^{1}H NMR spectrum of the crude reaction mixture using 1,3,5-triisopropylbenzene as an internal standard.

Supplementary Table 2. Initial condition study with aza-*para*-quinone methide as intermediate[a]

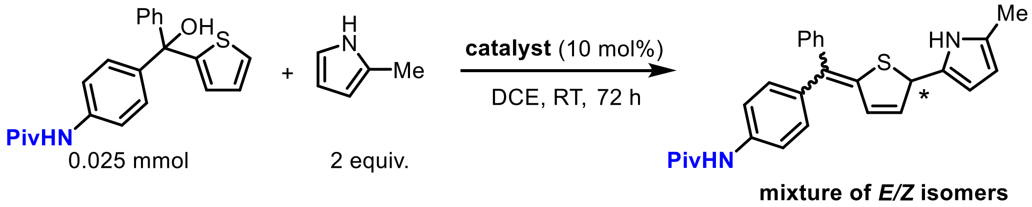

Entry	Catalyst	Conv.(%)	Major(%)	Minor(%)	Ratio
1	(*R*)-BINOL-TRIP-OH	>95	48	53	1.1:1
2	(*R*)-8H-BINOL-TRIP-OH	95	32	35	1.1:1
3	(*R*)-SPINOL-TRIP-OH	31	18	15	1.2:1
4	(*R*)-8H-BINOL-TRIP-NHTf	62	29	28	1:1

[a] Reaction conditions: tertiary alcohol (0.025 mmol), 2-methylpyrrole (2 equiv.), catalyst (5 mol%), DCE (0.5 mL). [b] Yield is based on analysis of the ^{1}H NMR spectrum of the crude reaction mixture using 1,3,5-triisopropylbenzene as an internal standard.

Supplementary Table 3. Initial condition study with *ortho*-quinone methide as intermediate[a]

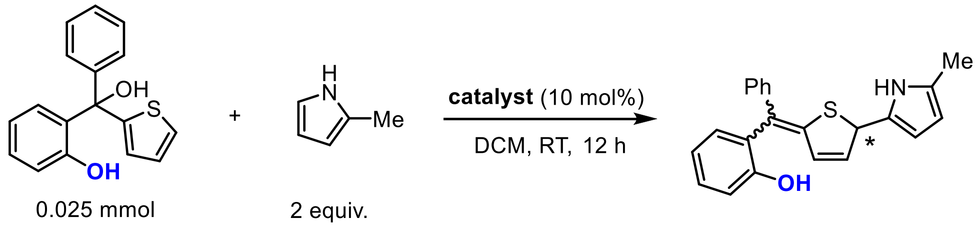

Entry	Catalyst	Conv.(%)	Yield	*E/Z (or Z/E)*	ee
1	(*R*)-BINOL-1-TRIP-OH	100	98	>20/1	28
2	(*R*)-BINOL-2,6-(^{i}Pr)$_2$-4-Ad-C$_6$H$_2$-OH	100	79	12.6/1	0
3	(*R*)-BINOL-2,4,6-Cy$_3$-C$_6$H$_2$-OH	91	96	>20/1	11
4	(*R*)-BINOL-SiPh$_3$-OH	93	95	>20/1	21
5	(*R*)-BINOL-2,6-Me$_2$-4-OMe-C$_6$H$_2$-OH	>95	90	12.6/1	-1
6	(*R*)-BINOL-C$_6$F$_5$-OH	100	69	4/1	7
7	(*S*)-BINOL-1-Nap-OH	100	76	7/1	-8
8	(*S*)-BINOL-9-Phen-OH	100	74	5.6/1	-5
9	(*R*)-BINOL-9-An-OH	100	82	12.71	-1
10	(*R*)-BINOL-1-Pyrene-OH	100	83	4.7/1	6

[a] Reaction conditions: tertiary alcohol (0.025 mmol), 2-methylpyrrole (2 equiv.), catalyst (10 mol%), DCM (0.5 mL). Yield is based on analysis of the ^{1}H NMR spectrum of the crude reaction mixture using 1,3,5-triisopropylbenzene as an internal standard.

Supplementary Table 4. Initial condition study with *ortho*-quinone methide as intermediate[a]

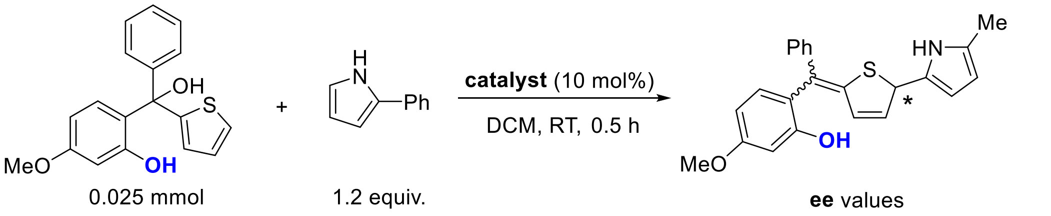

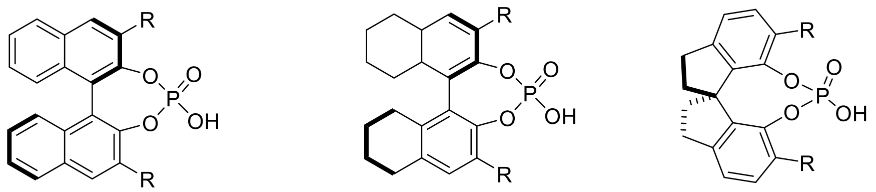

(*R*)-**A1**: R = 1-Ph, **-9%**
(*R*)-**A2**: R = 2,4,6-(*i*Pr)$_3$C$_6$H$_2$, **-32%**
(*S*)-**A3**: R = 1-Nap, **4%**
(*R*)-**A4**: R = 9-anthryl, **-41%**
(*S*)-**A5**: R = 9-phenanthryl, **22%**

(*R*)-**B1**: R = 2,4,6-(*i*Pr)$_3$C$_6$H$_2$, **-54%**
(*R*)-**B3**: R = 1-Nap, **-6%**
(*R*)-**B4**: R = 9-anthryl, **-31%**
(*R*)-**B5**: R = 9-phenanthryl, **-27%**

(*R*)-**C1:** R = 2,4,6-(iP*r*)3C$_6$H$_2$, **-8%**
(*S*)-**C2:** R = 1-Nap, **-70%**
(*R*)-**C3:** R = 9-anthryl, **75%**
(*R*)-**C4:** R = 9-phenanthryl, **77%**

Entry	Catalyst	Conv.(%)	Yield (%)	*Z*/*E* (*E*/*Z*)	ee
1	(*R*)-BINOL-1-Ph-C$_6$H$_4$OH	63	39	6.3/1	-9
2	(*R*)-BINOL-TRIP-OH	100	79	8/1	-30
3	(*S*)-BINOL-1-Nap-OH	100	71	3.5/1	4
4	(*R*)-BINOL-9-An-OH	100	89	9.5/1	-41
5	(*S*)-BINOL-9-Phen-OH	100	70	3.5/1	22
6	(*R*)-8H-BINOL-TRIP-OH	50	46	9.5/1	-54
7	(*R*)-8H-BINOL-1-Nap-OH	100	86	3.5/1	-6
8	(*R*)-8H-BINOL-9-An-OH	100	96	10.8/1	-31
9	(*R*)-8H-BINOL-9-Phen-OH	100	92	9.2/1	-27
10	(*R*)-SPINOL-TRIP-OH	30	14	2.4/1	8
11	(*S*)-SPINOL-1-Nap-OH	82	68	11.2/1	-70
12	(*R*)-SPINOL-9-An-OH	100	74	11.8/1	75
13	(*R*)-SPINOL9-Phen-OH	100	91	11.8/1	77

[a] Reaction conditions: tertiary alcohol (0.025 mmol), 2-phenylpyrrole (1.2 equiv.), catalyst (10 mol%), DCM (0.5 mL). Yield is based on analysis of the ^{1}H NMR spectrum of the crude reaction mixture using 1,3,5-triisopropylbenzene as an internal standard.

Supplementary Table 5. Catalyst screening of the reaction of **1a** and **2a**[a]

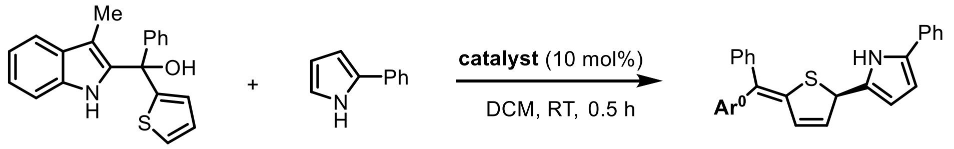

1a 0.025 mmol; **2a** 1.2 equiv.; **Ar^0** = 2-(3-methyl)indolyl; **3a**

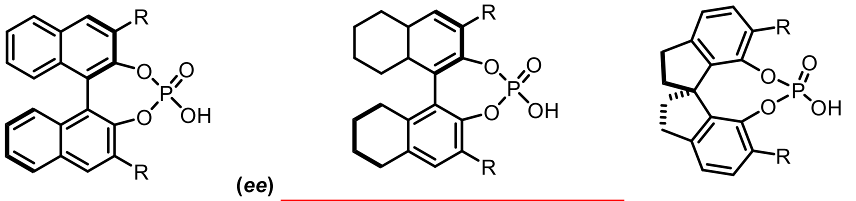

(*ee*)

(*R*)-**A1**: R = 2,4,6-(*i*Pr)$_3$C$_6$H$_2$, **83%**
(*R*)-**A2**: R = 2,6-(*i*Pr)$_2$-4-AdC$_6$H$_2$,**80%**
(*R*)-**A3**: R = 2,4,6-Cy$_3$C$_6$H$_2$, **79%**
(*S*)-**A4**: R = 1-Nap, **-11%**
(*R*)-**A5**: R = 9-anthryl, **34%**
(*S*)-**A6**: R = 9-phenanthryl, **-5%**
(*R*)-**A7**: R = SiPh$_3$ **58%**

(*R*)-**B1**: R = 2,4,6-(*i*Pr)$_3$C$_6$H$_2$, **84%**
(*R*)-**B2**: R = 2,4,6-Cy$_3$C$_6$H$_2$, **85%**
(*R*)-**B3**: R = 1-Nap, **33%**
(*R*)-**B4**: R = 9-anthryl, **17%**
(*R*)-**B5**: R = 9-phenanthryl, **22%**

(*R*)-**C1:** R = 2,4,6-(iP*r*)$_3$C$_6$H$_2$, **-77%**
(*S*)-**C2**: R = 2,4,6-Cy$_3$C$_6$H$_2$, **44%**
(*R*)-**C3:** R = 1-Nap, **-26%**
(*S*)-**C4:** R = 9-anthryl, **72%**
(*S*)-**C5:** R = 9-phenanthryl, **25%**
(*R*)-**C6:** R = SiPh$_3$ **-20%**

Entry	Catalyst	Conv.(%)	Yield (%)	*Z*/*E* (*E*/*Z*)	ee (%)
1	(*R*)-BINOL-TRIP-OH	100	85	20.3/1	83.2
2	(*R*)-BINOL-2,6-(i*P*r)2_4-AdC$_6$H$_2$-OH	100	89	23.1/1	80.2
3	(*R*)-BINOL-2,4,6-Cy$_3$C$_6$H$_2$-OH,	100	92	25.2/1	78.5
4	(*S*)-BINOL-1-Nap-OH	98	88	6/1	-11.3
5	(*R*)-BINOL-9-An-OH	100	80	17.7/1	34.4
6	(*S*)-BINOL-9-Phen-OH	100	68	8.3/1	-4.6
7	(*R*)-BINOL-SiPh$_3$-OH	94	90	27.3/1	57.8
8	(*R*)-8H-BINOL-TRIP-OH	98	94	30/1	84.3
9	(*R*)-8H-BINOL-2,4,6-Cy$_3$C$_6$H$_2$-OH,	93	91	28.1/1	84.6
10	(*R*)-8H-BINOL-1-Nap-OH	100	86	6.3/1	32.6
11	(*R*)-8H-BINOL-9-An-OH	100	81	18.4/1	17.1
12	(*R*)-8H-BINOL-9-Phen-OH	95	85	23.4/1	21.5
13	(*R*)-SPINOL-TRIP-OH	37	32	6.3/1	-76.6
14	(*S*)-SPINOL-2,4,6-Cy$_3$C$_6$H$_2$-OH,	18	17	3.5/1	43.5
15	(*R*)-SPINOL-1-Nap-OH	100	82	20/1	-25.7
16	(*S*)-SPINOL-9-An-OH	100	82	17.7/1	72.4
17	(*S*)-SPINOL9-Phen-OH	100	62	14/1	24.6
18	(*R*)-SPINOL-SiPh$_3$-OH	36	36	8.4/1	-19.8

[a] Reaction conditions: tertiary alcohol (0.025 mmol), 2-phenylpyrrole (1.2 equiv.), catalyst (10 mol%), DCM (0.5 mL). Yield is based on analysis of the ^{1}H NMR spectrum of the crude reaction mixture using 1,3,5-triisopropylbenzene as an internal standard.

Supplementary Table 6. Solvent screening of the reaction of **1a** and **2a** by using (*R*)-8H-BINOL-TRIP-OH as the catalyst[a]

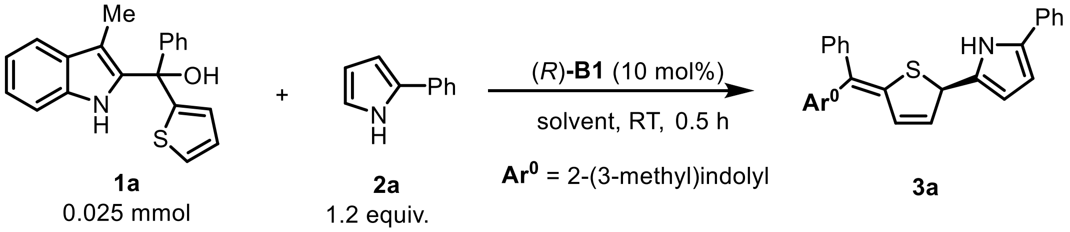

Entry	Solvent	Conv.(%)	Yield (%)	*E*/*Z*	Ee (%)
1	DCM	100	91	>20/1	86
2	DCE	100	94	>20/1	77
3	$CHCl_3$	76	73	17.3/1	79
4	***toluene***	***91***	***87***	>20/1	***88***
5	$PhCF_3$	100	93	>20/1	83
6	PhF	100	87	18.4/1	86
7	CCl_4	100	>99	>20/1	81
8	Et_2O	100	91	9.6/1	(<80)
9	***PhCl***	***100***	***>99***	>20/1	***87***
10	EtOAc	100	90	13.1/1	74

[a] Reaction conditions: tertiary alcohol (0.025 mmol), 2-phenylpyrrole (1.2 equiv.), catalyst (10 mol%), solvent (0.5 mL). Yield is based on analysis of the ^1H NMR spectrum of the crude reaction mixture using 1,3,5-triisopropylbenzene as an internal standard.

Supplementary Table 7. Solvent screening of the reaction of **1a** and **2a** by using (*R*)-8H-BINOL-2,4,6-$Cy_3C_6H_2$-OH as the catalyst[a]

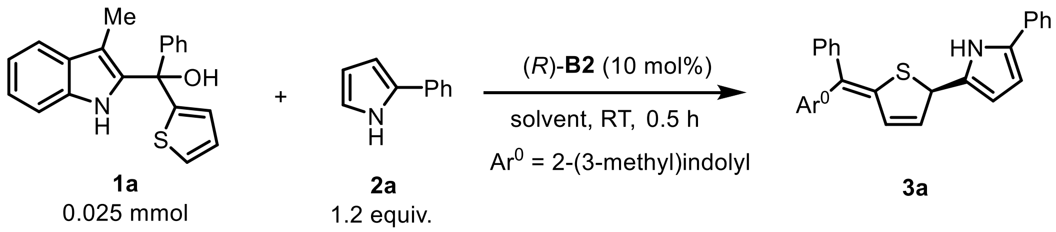

Entry	Solvent	Conv.(%)	Yield (%)	*E*/*Z*	Ee (%)
1	DCM	97	93	>20/1	84
2	DCE	87	88	>20/1	76
3	$CHCl_3$	85-90	83	20/1	85
4	toluene	98	95	>20/1	88
5	$PhCF_3$	100	91	>20/1	89 (88)
6	PhF	100	98	>20/1	88
7	CCl_4	100	96	>20/1	85
8	Et_2O	50-60	47	9.3/1	82
9	PhCl	100	>99	>20/1	>90 (89)
10	EtOAc	24	26	>20/1	86

[a]Reaction conditions: tertiary alcohol (0.025 mmol), 2-phenylpyrrole (1.2 equiv.), catalyst (10 mol%), solvent (0.5 mL). Yield is based on analysis of the ^{1}H NMR spectrum of the crude reaction mixture using 1,3,5-triisopropylbenzene as an internal standard.

Supplementary Table 8. Reaction temperature screening of the reaction of **1a** and **2a**[a]

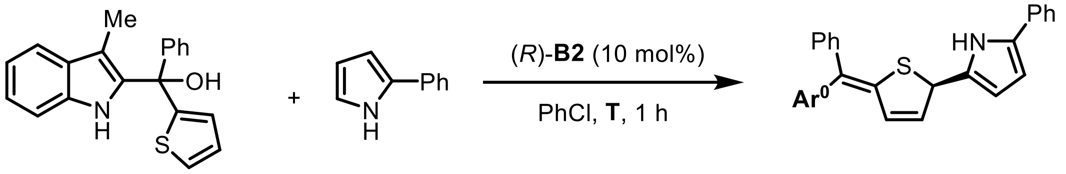

1a 0.025 mmol   **2a** 1.2 equiv.   **Ar^0** = 2-(3-methyl)indolyl   **3a**

Entry	T (°C)	Conv.(%)	Yield (%)	*E*/*Z*	Ee (%)
1	40	100	69	>20/1	81
2	rt	100	99	>20/1	88
3	0	97	96	>20/1	93
4	-20	79	88	>20/1	95

[a] Reaction conditions: tertiary alcohol (0.025 mmol), 2-phenylpyrrole (1.2 equiv.), catalyst (10 mol%), PhCl (0.5 mL). Yield is based on analysis of the ^{1}H NMR spectrum of the crude reaction mixture using 1,3,5-triisopropylbenzene as an internal standard.

Supplementary Table 9. Reaction temperature screening of the reaction of **1a** and **2a**[a]

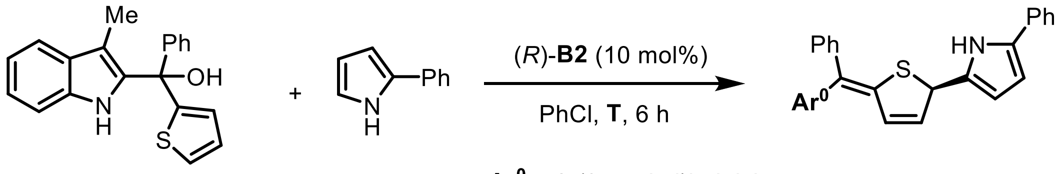

1a 0.025 mmol   **2a** 1.2 equiv.   **3a**

Entry	T (°C)	Conv.(%)	Yield	*E/Z*	Ee (%)
1	0	100	98	>20/1	93
2	-20	100	>99	>20/1	95
3	***-40***	***100***	***>99***	***>20/1***	***96***

[a] Reaction conditions: tertiary alcohol (0.025 mmol), 2-phenylpyrrole (1.2 equiv.), catalyst (10 mol%), PhCl (0.5 mL). Yield is based on analysis of the ^{1}H NMR spectrum of the crude reaction mixture using 1,3,5-triisopropylbenzene as an internal standard.

Supplementary Table 10. Solvent screening of the reaction of **4a** and **2a**[a]

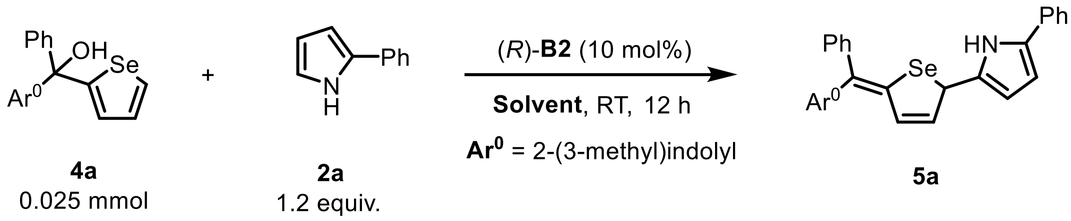

Entry	Solvent	Conv.(%)	Yield (%)	*E/Z*	ee (%)
1	DCM	100	84	>20/1	80
2	DCE	100	96	>20/1	73
3	$CHCl_3$	100	91	>20/1	78
4	toluene	100	99	>20/1	90
5	$PhCF_3$	100	89	>20/1	89
6	PhF	100	90	>20/1	86
7	CCl_4	96	86	>20/1	90
8	Et_2O	44	44	>20/1	83
9	PhCl	100	97	>20/1	86
10	EtOAc	68	76	>20/1	76

[a] Reaction conditions: tertiary alcohol (0.025 mmol), 2-phenylpyrrole (1.2 equiv.), catalyst (10 mol%), solvent (0.5 mL). Yield is based on analysis of the ^{1}H NMR spectrum of the crude reaction mixture using 1,3,5-triisopropylbenzene as an internal standard.

Supplementary Table 11. Reaction temperature screening of the reaction of **4a** and **2a**[a]

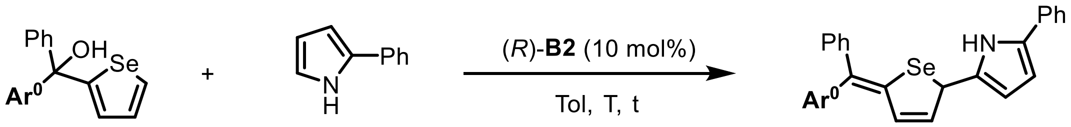

4a 0.025 mmol | **2a** 1.2 equiv. | **5a**

Entry	T (°C)	t (h)	Conv.(%)	Yield (%)	*E*/*Z*	ee (%)
1	40	4	100	99	>20/1	86
2	rt	12	100	99	>20/1	90
3	0	48	100	97	>20/1	94
4	-20	48	100	98	>20/1	94
5	-40	48	72	70	>20/1	93

[a] Reaction conditions: tertiary alcohol (0.025 mmol), 2-phenylpyrrole (1.2 equiv.), catalyst (10 mol%), toluene (0.5 mL). Yield is based on analysis of the ^{1}H NMR spectrum of the crude reaction mixture using 1,3,5-triisopropylbenzene as an internal standard.

Supplementary Table 12. The reaction of **1a** and other nucleophiles[a]

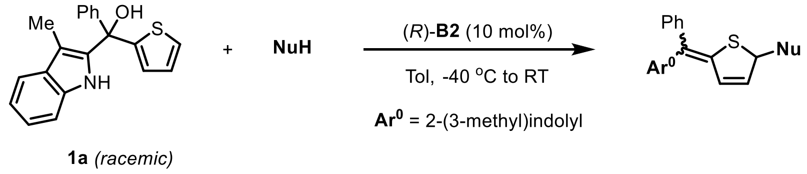

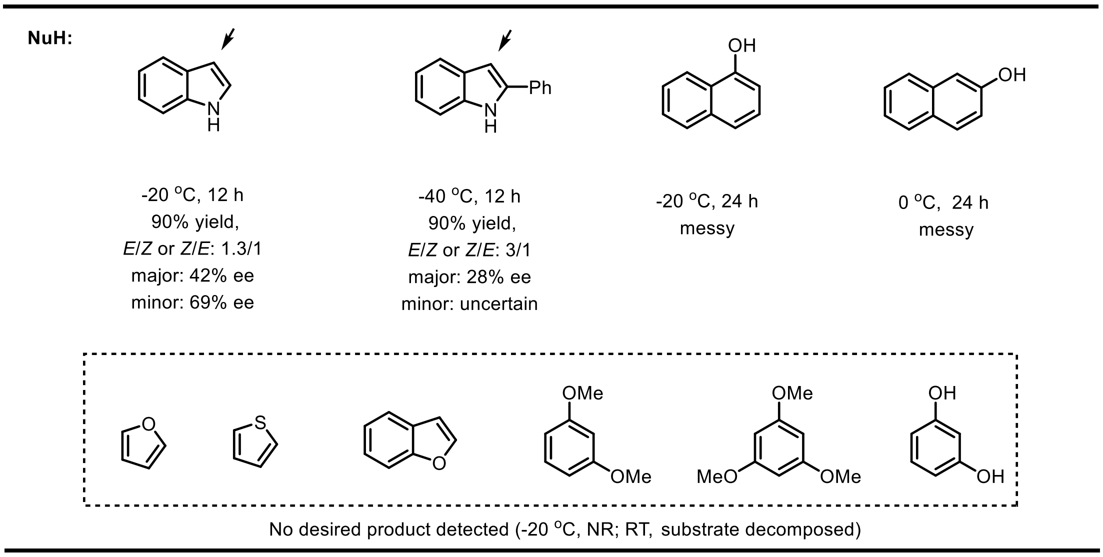

[a] Reaction conditions: **1a** (0.025 mmol), NuH (1.2 equiv.), (*R*)-**B2** (10 mol%), toluene (0.5 mL). Yield is based on analysis of the 1H NMR spectroscopy of the crude reaction mixture using 1,3,5-triisopropylbenzene as an internal standard.

IV. Dearomatization of Thiophenes and Selenophenes

General Procedure B.

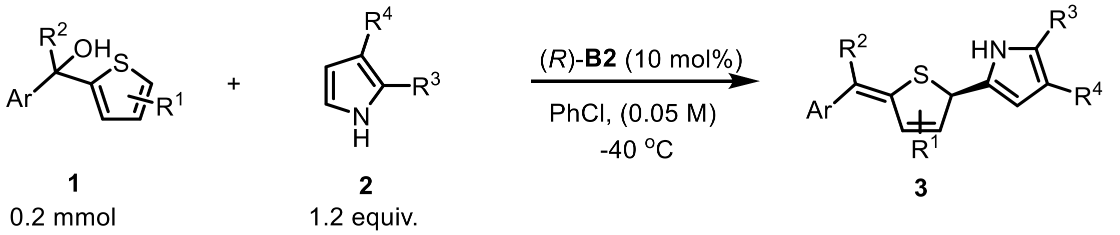

At -40 °C, to an oven-dried 8-mL vial charged with a solution of the tertiary alcohol **1** (0.2 mmol) and pyrrole **2** (0.24 mmol) in PhCl (3.6 mL) was slowly added a solution of catalyst (*R*)-**B2** (20 mg, 0.015 mmol, 10 mol%) in PhCl (0.4 mL). The reaction mixture was stirred at the same temperature for 12 h. After that, triethylamine (2 drops) was added to quench the reaction. The mixture was concentrated under reduced pressure and purified by silica gel (deactivated by triethylamine) flash chromatography to afford the desired product **3**.

General Procedure C.

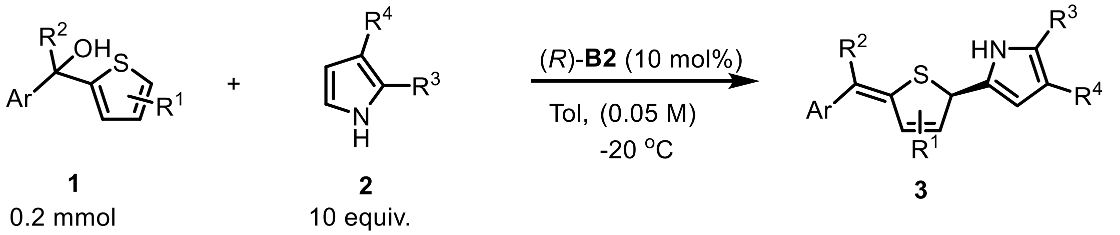

At -20 °C, to an oven-dried 8-mL vial charged with a solution of the tertiary alcohol **1** (0.2 mmol) and pyrrole **2** (2.0 mmol) in toluene (3.6 mL) was slowly added a solution of catalyst (*R*)-**B2** (20 mg, 0.015 mmol, 10 mol%) in toluene (0.4 mL). The reaction mixture was stirred at the same temperature for 24 h. After that, triethylamine (2 drops) was added to quench the reaction. The mixture was concentrated under reduced pressure and purified by silica gel (deactivated by triethylamine) flash chromatography to afford the desired product **3**.

Unless noted otherwise, all the racemic products (used for HPLC reference in determining the ee value) were prepared from the same reaction using racemic BINOL-TRIP-OH as the catalyst.

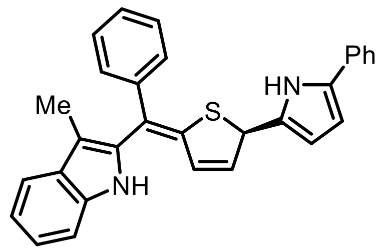

3a

(*R*,*E*)-3-Methyl-2-(phenyl(5-(5-phenyl-1*H*-pyrrol-2-yl)thiophen-2(5*H*)-ylidene)methyl)-1*H*-indole (3a) was prepared as pale green foam from (3-methyl-1*H*-indol-2-yl)(phenyl)(thiophen-2-yl)methanol (63.9 mg, 0.2 mmol) and 2-phenylpyrrole (34.3 mg, 0.24 mmol) according to the General Procedure B (eluent: hexanes/EtOAc = 15:1 → 10:1) in 94% yield (83.7 mg, 95% ee, *E*/*Z* > 20:1).

$[\alpha]_D^{26}$: +176.1 (*c* = 1.0, CH_2Cl_2). HPLC analysis of the product: Daicel CHIRALPAK OD-H column; 10% *i*-PrOH in hexanes; 1.0 mL/min; retention times: 25.6 min (major), 34.9 min (minor).

^{1}H NMR (400 MHz, acetone-d_6) δ 10.48 (s, 1H), 9.82 (s, 1H), 7.62 (d, *J* = 7.6 Hz, 2H), 7.57 (d, *J* = 7.7 Hz, 1H), 7.39-7.28 (m, 7H), 7.21-7.05 (m, 4H), 6.52 (dd, J_1 = 2.1 Hz, J_2 = 6.2 Hz, 1H), 6.48 (t, *J* = 2.9 Hz, 1H), 6.40 (dd, J_1 = 3.0 Hz, J_2 = 6.2 Hz, 1H), 6.12 (t, *J* = 2.6 Hz, 1H), 5.94 (t, *J* = 2.5 Hz, 1H), 2.19 (s, 3H).

^{13}C NMR (100 MHz, acetone-d_6) δ 147.1, 141.9, 137.2, 135.3, 133.9, 133.4, 133.3, 132.5, 129.8, 129.5, 129.2, 128.9, 127.8, 126.5, 124.4(2C), 122.4, 121.4, 119.5, 119.2, 111.6, 110.7, 108.8, 106.9, 54.0, 9.8.

IR (thin film) 3408, 3051, 2915, 2859, 1690, 1598, 1484, 1253, 1139, 732, 690 cm^{-1}.

HRMS (CI+) Calcd for $C_{30}H_{24}N_2S$ (M^+): 444.1660, Found: 444.1668.

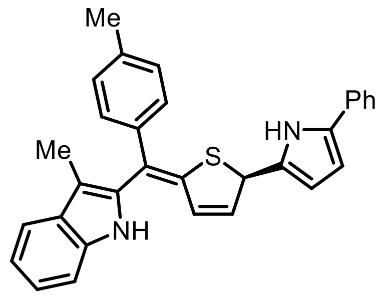

3b

(*R*,*E*)-3-Methyl-2-((5-(5-phenyl-1*H*-pyrrol-2-yl)thiophen-2(5*H*)-ylidene)(*p*-tolyl)methyl)-1*H*-indole (3b) was prepared as slightly green foam from

(3-methyl-1*H*-indol-2-yl)(thiophen-2-yl)(*p*-tolyl)methanol (66.7 mg, 0.2 mmol) and 2-phenylpyrrole (34.3 mg, 0.24 mmol) according to the General Procedure B (eluent: hexanes/EtOAc = 15:1 → 10:1) in 94% yield (86.0 mg, 92% ee, *E*/*Z* > 20:1).

$[\alpha]_D^{26}$: +119.5 (c = 1.0, CH_2Cl_2). HPLC analysis of the product: Daicel CHIRALPAK OD-H column; 20% *i*-PrOH in hexanes; 1.0 mL/min; retention times: 11.1 min (major), 13.8 min (minor).

^{1}H NMR (400 MHz, acetone-d_6) δ 10.47 (s, 1H), 9.79 (s, 1H), 7.62 (d, J = 7.4 Hz, 2H), 7.57 (d, J = 7.8 Hz, 1H), 7.35-7.26 (m, 5H), 7.17-7.04 (m, 5H), 6.50 (dd, J_1 = 2.2 Hz, J_2 = 6.2 Hz, 1H), 6.48 (t, J = 3.0 Hz, 1H), 6.37 (dd, J_1 = 3.0 Hz, J_2 = 6.2 Hz, 1H), 6.11 (t, J = 2.7 Hz, 1H), 5.94 (t, J = 2.5 Hz, 1H), 2.29 (s, 3H), 2.18 (s, 3H).

^{13}C NMR (100 MHz, acetone-d_6) δ 146.4, 139.0, 137.4, 137.2, 136.9, 135.5, 133.9, 133.4, 133.3, 132.6, 129.8, 129.6, 129.5, 129.2, 126.5, 124.4, 122.4, 121.4, 119.5, 119.2, 111.6, 110.6, 108.8, 106.9, 54.0, 21.2, 9.8.

IR (thin film) 3418, 3049, 2919, 1723, 1601, 1455, 1260, 1237, 1040, 731, 695 cm^{-1}.

HRMS (CI+) Calcd for $C_{31}H_{26}N_2S$ (M^+): 458.1817, Found: 458.1810.

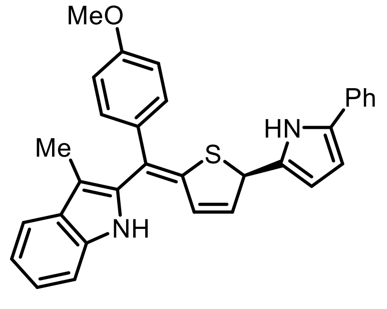

3c

(*R*,*E*)-2-((4-Methoxyphenyl)(5-(5-phenyl-1*H*-pyrrol-2-yl)thiophen-2(5*H*)-ylidene) methyl)-3-methyl-1*H*-indole (3c) was prepared as slightly green foam from (4-methoxyphenyl)(3-methyl-1*H*-indol-2-yl)(thiophen-2-yl)methanol (69.9 mg, 0.2 mmol) and 2-phenylpyrrole (34.3 mg, 0.24 mmol) according to the General Procedure B (eluent: hexanes/EtOAc = 15:1 → 10:1) in 97% yield (92.1 mg, 95% ee, *E*/*Z* > 20:1).

$[\alpha]_D^{26}$: +132.1 (c = 1.0, CH_2Cl_2). HPLC analysis of the product: Daicel CHIRALPAK OD-H column; 20% *i*-PrOH in hexanes; 1.0 mL/min; retention times: 16.4 min (major), 21.4 min (minor).

^{1}H NMR (400 MHz, acetone-d_6) δ 10.46 (s, 1H), 9.6 (s, 1H), 7.62 (d, J = 7.5 Hz, 2H),

7.57 (d, J = 7.7 Hz, 1H), 7.36-7.31 (m, 5H), 7.17-7.05 (m, 3H), 6.86 (d, J = 8.8 Hz, 2H), 6.51-6.48 (m, 2H), 6.34 (dd, J_1 = 3.0 Hz, J_2 = 6.1 Hz, 1H), 6.12 (t, J = 2.5 Hz, 1H), 5.92 (t, J = 2.6 Hz, 1H), 3.75 (s, 3H), 2.19 (s, 3H).

^{13}C NMR (100 MHz, acetone-d_6) δ 159.5, 145.5, 137.2, 136.4, 135.5, 134.2, 133.9, 133.4, 133.3, 132.7, 130.6, 129.8, 129.5, 126.5, 124.4, 122.4, 121.2, 119.5, 119.2, 114.2, 111.6, 110.5, 108.8, 106.9, 55.5, 53.9, 9.8.

IR (thin film) 3441, 3051, 2923, 1694, 1505, 1455, 1252, 1177, 1142, 1030, 730, 573 cm^{-1}.

HRMS (CI+) Calcd for $C_{31}H_{26}N_2OS$ (M^+): 474.1766, Found: 474.1772.

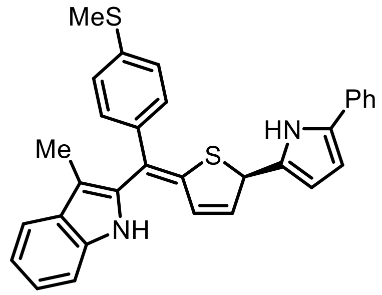

3d

(*R*,*E*)-3-Methyl-2-((4-(methylthio)phenyl)(5-(5-phenyl-1*H*-pyrrol-2-yl)thiophen-2(5*H*)-ylidene)methyl)-1*H*-indole (3d) was prepared as slightly green foam from (3-methyl-1*H*-indol-2-yl)(4-(methylthio)phenyl)(thiophen-2-yl)- methanol (73.1 mg, 0.2 mmol) and 2-phenylpyrrole (34.3 mg, 0.24 mmol) according to the General Procedure B (eluent: hexanes/EtOAc = 15:1 → 10:1) in 93% yield (91.3 mg, 99% ee, $E/Z > 20:1$).

$[\alpha]_D^{26}$: +123.4 (c = 1.0, CH_2Cl_2). HPLC analysis of the product: Daicel CHIRALPAK OD-H column; 20% *i*-PrOH in hexanes; 1.0 mL/min; retention times: 17.1 min (major), 21.5 min (minor).

^{1}H NMR (400 MHz, acetone-d_6) δ 10.47 (s, 1H), 9.80 (s, 1H), 7.61 (d, J = 7.3 Hz, 2H), 7.57 (d, J = 7.8 Hz, 1H), 7.35-7.30 (m, 5H), 7.20-7.05 (m, 5H), 6.50 (dd, J_1 = 2.2 Hz, J_2 = 6.2 Hz, 1H), 6.48 (t, J = 3.0 Hz, 1H), 6.38 (dd, J_1 = 3.0 Hz, J_2 = 6.2 Hz, 1H), 6.12 (t, J = 2.6 Hz, 1H), 5.95 (t, J = 2.5 Hz, 1H), 2.44 (s, 3H), 2.18 (s, 3H).

^{13}C NMR (100 MHz, acetone-d_6) δ 146.7, 138.4, 138.3, 137.2, 137.1, 135.2, 133.9 (2C), 133.5, 133.3, 132.5, 129.7, 129.5, 126.55, 126.49, 124.4, 122.5, 120.9, 119.5, 119.3, 111.6, 110.7, 108.9, 106.9, 54.1, 15.3, 9.8.

IR (thin film) 3404, 3050, 2918, 1693, 1595, 1484, 1254, 1191, 730, 694 cm^{-1}.

HRMS (CI+) Calcd for $C_{31}H_{26}N_2S_2$ (M^+): 490.1537, Found: 490.1553.

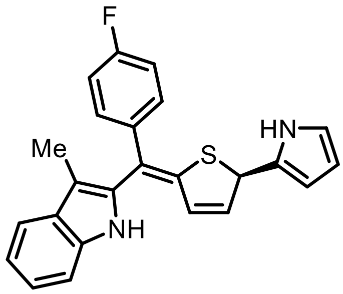

3e

(*R*,*E*)-2-((5-(1*H*-Pyrrol-2-yl)thiophen-2(5*H*)-ylidene)(4-fluorophenyl)methyl)-3-methyl-1*H*-indole (3e) was prepared as slightly purple foam from (3-methyl-1*H*-indol-2-yl)(4-fluorophenyl)(thiophen-2-yl)-methanol (67.5 mg, 0.2 mmol) and pyrrole (134.0 mg, 2.0 mmol) according to the General Procedure C (eluent: hexanes/EtOAc = 40:1 → 20:1) in 92% yield (71.2 mg, 97% ee, *E*/*Z* >20:1). $[\alpha]_D^{26}$: +130.1 (c = 1.0, CH_2Cl_2). HPLC analysis of the product: Daicel CHIRALPAK AD-H column; 20% *i*-PrOH in hexanes; 1.0 mL/min; retention times: 9.2 min (major), 18.5 min (minor).

^{1}H NMR (400 MHz, acetone-d_6) δ 10.03 (s, 1H), 9.83 (s, 1H), 7.55 (d, J = 7.8 Hz, 1H), 7.41-7.38 (m, 2H), 7.33 (d, J = 8.0 Hz, 1H), 7.14-7.04 (m, 4H), 6.78-6.76 (m, 1H), 6.50-6.47 (m, 1H), 6.34-6.32 (m, 1H), 6.06-6.02 (m, 2H), 5.93-5.90 (m, 1H), 2.17 (s, 3H).

^{13}C NMR (100 MHz, acetone-d_6) δ 162.2 (d, J = 244.0 Hz), 147.2, 138.3 (d, J = 3.1 Hz), 137.9, 137.2, 135.1, 132.9, 131.3 (d, J = 8.0 Hz), 130.2, 129.7, 122.5, 120.3, 119.6, 119.32, 119.27, 115.7 (d, J = 21.5 Hz), 111.6, 110.7, 108.7, 107.2, 54.1, 9.8.

^{19}F NMR (376 MHz, acetone-d_6) δ -115.4.

IR (thin film) 3417, 3050, 2965, 1597, 1441, 1307, 1084, 1017, 793, 730 cm^{-1}.

HRMS (CI+) Calcd for $C_{24}H_{19}FN_2S$ (M^+): 386.1253, Found: 386.1239.

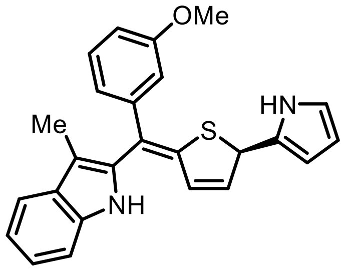

3f

(*R*,*E*)-2-((5-(1*H*-pyrrol-2-yl)thiophen-2(5*H*)-ylidene)(3-methoxyphenyl)methyl)-3-methyl-1*H*-indole (3f) was prepared as slightly purple foam from (3-methyl-1*H*-indol-2-yl)(3-methoxyphenyl)(thiophen-2-yl)-methanol (69.9 mg, 0.2 mmol) and pyrrole (134.0 mg, 2.0 mmol) according to the General Procedure C (eluent: hexanes/EtOAc = 40:1 → 20:1) in 92% yield (73.2 mg, 97% ee, *E*/*Z* > 20:1). $[\alpha]_D^{26}$: -55.1 (*c* = 1.0, CH_2Cl_2). HPLC analysis of the product: Daicel CHIRALPAK AD-H column; 20% *i*-PrOH in hexanes; 1.0 mL/min; retention times: 11.1 min (major), 21.2 min (minor).

^{1}H NMR (400 MHz, acetone-d_6) δ 10.02 (s, 1H), 9.82 (s, 1H), 7.55 (d, *J* = 7.8 Hz, 1H), 7.33 (d, *J* = 8.0 Hz, 1H), 7.22-7.15 (m, 4H), 6.90 (d, *J* = 7.8 Hz, 1H), 6.80-6.75 (m, 2H), 6.47 (dd, J_1 = 2.2 Hz, J_2 = 6.2 Hz, 1H), 6.47 (dd, J_1 = 3.0 Hz, J_2 = 6.2 Hz, 1H), 6.06-6.02 (m, 2H), 5.90 (t, *J* = 2.5 Hz, 1H), 3.71 (s, 3H), 2.78 (s, 3H).

^{13}C NMR (100 MHz, acetone-d_6) δ 160.4, 147.3, 143.2, 137.9, 137.2, 135.2, 133.0, 130.2, 130.0, 129.7, 122.4, 121.8, 121.3, 119.5, 119.3, 119.2, 114.9, 113.0, 111.6, 110.6, 108.7, 107.2, 55.4, 54.2, 9.8.

IR (thin film) 3395, 3049, 2925, 1584, 1448, 1259, 1135, 1030, 870, 792, 722 cm^{-1}.

HRMS (CI+) Calcd for $C_{25}H_{22}N_2OS$, (M^+): 398.1453, Found: 398.1442.

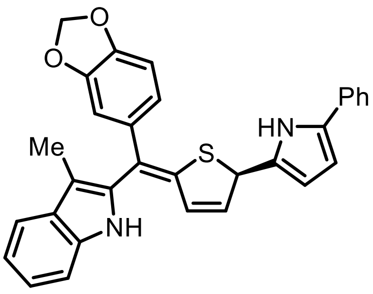

3g

(*R*,*E*)-2-(Benzo[*d*][1,3]dioxol-5-yl(5-(5-phenyl-1*H*-pyrrol-2-yl)thiophen-2(5*H*)-ylidene)methyl)-3-methyl-1*H*-indole (3g) was prepared as slightly green foam from

(3-methyl-1*H*-indol-2-yl)di(thiophen-2-yl)methanol (72.7 mg, 0.2 mmol) and 2-phenylpyrrole (34.3 mg, 0.24 mmol) according to the General Procedure B (eluent: hexanes/EtOAc = 15:1 → 10:1) in 93% yield (91.1 mg, 95% ee, *E*/*Z* > 20:1).

$[\alpha]_D^{26}$: +438.0 (c = 1.0, CH_2Cl_2). HPLC analysis of the product: Daicel CHIRALPAK OD-H column; 20% *i*-PrOH in hexanes; 1.0 mL/min; retention times: 18.8 min (major), 31.5 min (minor).

^{1}H NMR (400 MHz, acetone-d_6) δ 10.46 (s, 1H), 9.79 (s, 1H), 7.62 (d, J = 7.4 Hz, 2H), 7.57 (d, J = 7.8 Hz, 1H), 7.36-7.31 (m, 3H), 7.17-7.05 (m, 3H), 6.92 (d, J = 8.2 Hz, 1H), 6.87 (s, 1H), 6.79 (J = 8.2 Hz, 1H), 6.50-6.48 (m, 2H), 6.37 (dd, J_1 = 3.0 Hz, J_2 = 6.2 Hz, 1H), 6.12 (t, J = 2.6 Hz, 1H), 5.94 (s, 2H), 5.93 (t, J = 2.6 Hz, 1H), 2.20 (s, 3H).

^{13}C NMR (100 MHz, acetone-d_6) δ 148.4, 147.4, 146.1, 137.2, 136.9, 136.0, 135.3, 133.9, 133.31, 133.28, 132.6, 129.7, 129.5, 126.5, 1243, 123.3, 122.4, 121.1, 119.5, 119.2, 111.6, 110.6, 109.4, 108.8, 108.7, 106.9, 102.0, 53.9, 9.8.

IR (thin film) 3445, 3054, 2981, 2901, 1697, 1603, 1487, 1442, 1256, 1238, 1037, 730, 700 cm^{-1}.

HRMS (CI+) Calcd for $C_{31}H_{24}N_2O_2S$ (M^+): 488.1558, Found: 488.1569.

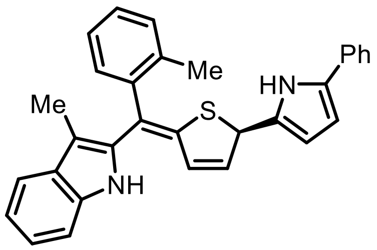

3h

(*R*,*E*)-3-Methyl-2-((5-(5-phenyl-1*H*-pyrrol-2-yl)thiophen-2(5*H*)-ylidene)(o-tolyl) methyl)-1*H*-indole (3h) was prepared as slightly green foam from (3-methyl-1*H*-indol-2-yl)(thiophen-2-yl)(*o*-tolyl)methanol (66.7 mg, 0.2 mmol) and 2-phenylpyrrole (34.3 mg, 0.24 mmol) according to the General Procedure B (eluent: hexanes/EtOAc = 15:1 → 10:1) in 87% yield (80.1 mg, 94% ee, *E*/*Z* > 20:1).

$[\alpha]_D^{26}$: +117.7 (c = 1.0, CH_2Cl_2). HPLC analysis of the product: Daicel CHIRALPAK OD-H column; 20% *i*-PrOH in hexanes; 1.0 mL/min; retention times: 9.8 min (major), 16.2 min (minor).

^{1}H NMR (400 MHz, acetone-d_6) δ 10.42 (s, 1H), 9.63 (s, 1H), 7.59 (d, J = 7.6 Hz,

2H), 7.57 (d, J = 7.7 Hz, 1H), 7.41-7.39 (m, 1H), 7.34-7.25 (m, 3H), 7.19-7.00 (m, 6H), 6.73 (dd, J_1 = 2.1 Hz, J_2 = 6.2 Hz, 1H), 6.48-6.46 (m, 2H), 6.09 (t, J = 2.5 Hz, 1H), 5.84 (t, J = 2.5 Hz, 1H), 2.23 (s, 3H), 2.11 (s, 3H).

^{13}C NMR (100 MHz, acetone-d_6) δ 147.7, 141.6, 138.2, 137.5, 137.3, 134.9, 133.9, 133.2, 132.9, 132.1, 131.3, 130.6, 130.1, 129.5, 128.6, 126.7, 126.5, 124.4, 122.4, 121.0, 119.5, 119.1, 111.5, 109.6, 108.6, 106.9, 53.2, 19.7, 10.3.

IR (thin film) 3448, 3051, 2960, 1605, 1456, 1332, 1133, 1116, 733, 702 cm^{-1}.

HRMS (CI+) Calcd for $C_{31}H_{26}N_2S$ (M^+): 458.1817, Found: 458.1821.

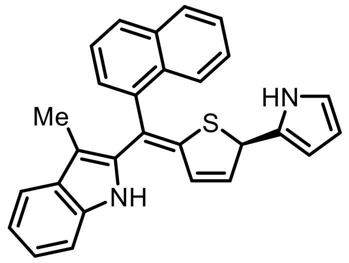

3i

(*R*,*E*)-2-((5-(1*H*-pyrrol-2-yl)thiophen-2(5*H*)-ylidene)(naphthalen-1-yl)methyl)-3-methyl-1*H*-indole (3i) was prepared as slightly yellowish foam from (3-methyl-1*H*-indol-2-yl)(naphthalen-1-yl)(thiophen-2-yl)methanol (69.9 mg, 0.2 mmol) and pyrrole (134.0 mg, 2.0 mmol) according to the General Procedure C (30 h, eluent: hexanes/EtOAc = 40:1 → 20:1) in 94% yield (78.7 mg, 96% ee, *E*/*Z* > 20:1). $[\alpha]_D^{26}$: +74.3 (c = 1.0, CH_2Cl_2). HPLC analysis of the product: Daicel CHIRALPAK AD-H column; 30% *i*-PrOH in hexanes; 1.0 mL/min; retention times: 7.8 min (major), 25.7 min (minor).

^{1}H NMR (400 MHz, acetone-d_6) δ 9.97 (s, 1H), 9.69 (s, 1H), 7.98 (d, J = 8.3 Hz, 1H), 7.89-7.85 (m, 2H), 7.66 (d, J = 7.0 Hz, 1H), 7.55-7.48 (m, 2H), 7.44-7.34 (m, 2H), 7.23 (d, J = 7.7 Hz, 1H), 7.09-7.01 (m, 2H), 6.83 (d, J = 6.2 Hz, 1H), 6.77-6.74 (m, 1H), 6.46-6.44 (m, 1H), 6.04-6.01 (m, 2H), 5.82-5.80 (m, 1H), 2.34 (s, 3H).

^{13}C NMR (100 MHz, acetone-d_6) δ 149.2, 139.8, 139.1, 137.2, 135.8, 135.0, 131.92, 131.86, 130.4, 130.2, 129.2, 129.0, 128.7, 126.8, 126.6, 126.42, 126.41, 122.4, 119.7, 119.5, 119.21, 119.19, 111.5, 109.4, 108.6, 107.1, 53.5, 10.5.

IR (thin film) 3403, 3048, 2917, 1574, 1455, 1331, 1260, 1139, 994, 785, 730 cm^{-1}.

HRMS (CI+) Calcd for $C_{28}H_{22}N_2S$ (M^+): 418.1504, Found: 418.1502.

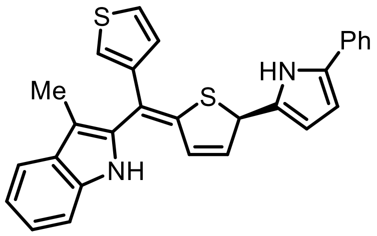

3j

(*R*,*E*)-3-Methyl-2-((5-(5-phenyl-1*H*-pyrrol-2-yl)thiophen-2(5*H*)-ylidene)(thiophen-3-yl)methyl)-1*H*-indole (3j) was prepared as slightly green foam from (3-methyl-1*H*-indol-2-yl)di(thiophen-3-yl)methanol (63.8 mg, 0.2 mmol) and 2-phenylpyrrole (34.3 mg, 0.24 mmol) according to the General Procedure B (eluent: hexanes/EtOAc = 15:1 → 10:1) in 94% yield (84.5 mg, 98% ee, *E*/*Z* > 20:1).

$[\alpha]_D^{26}$: +152.7 (c = 1.0, CH_2Cl_2). HPLC analysis of the product: Daicel CHIRALPAK OD-H column; 20% *i*-PrOH in hexanes; 1.0 mL/min; retention times: 14.9 min (major), 18.4 min (minor).

^{1}H NMR (400 MHz, acetone-d_6) δ 10.49 (s, 1H), 9.87 (s, 1H), 7.62 (d, J = 7.4 Hz, 2H), 7.57 (d, J = 7.8 Hz, 1H), 7.39-7.31 (m, 5H), 7.17-7.10 (m, 4H), 6.49 (t, J = 3.0 Hz, 1H), 6.43 (dd, J_1 = 2.1 Hz, J_2 = 6.1 Hz, 1H), 6.35 (dd, J_1 = 3.0 Hz, J_2 = 6.1 Hz, 1H), 6.12 (t, J = 2.6 Hz, 1H), 5.94 (t, J = 2.4 Hz, 1H), 2.18 (s, 3H).

^{13}C NMR (100 MHz, acetone-d_6) δ 146.3, 142.6, 137.0, 136.8, 135.2, 133.8, 133.31, 133.27, 132.2, 129.7, 129.5, 129.0, 126.5, 125.7, 124.4, 124.0, 122.4, 119.5, 119.3, 116.7, 111.7, 110.1, 108.9, 107.0, 54.6, 9.6.

IR (thin film) 3403, 3050, 2914, 1690, 1598, 1506, 1137, 1080, 784, 735, 687 cm^{-1}.

HRMS (CI+) Calcd for $C_{28}H_{22}N_2S_2$ (M^+): 450.1224, Found: 450.1219.

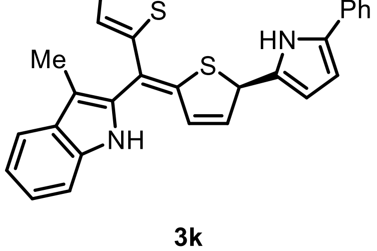

3k

(*R*,*E*)-3-Methyl-2-((5-(5-phenyl-1*H*-pyrrol-2-yl)thiophen-2(5*H*)-ylidene)(thiophen-2-yl)methyl)-1*H*-indole (3k) was prepared as slightly green foam from

(3-methyl-1*H*-indol-2-yl)di(thiophen-2-yl)methanol (63.8 mg, 0.2 mmol) and 2-phenylpyrrole (34.3 mg, 0.24 mmol) according to the General Procedure B (eluent: hexanes/EtOAc = 15:1 → 10:1) in 88% yield (79.5 mg, 98% ee, *E*/*Z* > 20:1).

$[\alpha]_D^{26}$: +96.9 (*c* = 1.0, CH_2Cl_2). HPLC analysis of the product: Daicel CHIRALPAK OD-H column; 20% *i*-PrOH in hexanes; 1.0 mL/min; retention times: 15.6 min (major), 20.3 min (minor).

^{1}H NMR (400 MHz, acetone-d_6) δ 10.54 (s, 1H), 10.02 (s, 1H), 7.63-7.59 (m, 3H), 7.42 (d, *J* = 8.0 Hz, 1H), 7.37-7.31 (m, 3H), 7.17-7.10 (m, 3H), 7.01-6.96 (m, 2H), 6.49 (t, *J* = 3.0 Hz, 1H), 6.37-6.32 (m, 2H), 6.12 (t, *J* = 2.8 Hz, 1H), 6.03 (t, *J* = 2.0 Hz, 1H), 2.20 (s, 3H).

^{13}C NMR (100 MHz, acetone-d_6) δ 145.9, 145.4, 137.04, 136.98, 134.4, 133.8, 133.4, 133.3, 131.8, 129.5, 129.4, 127.9, 127.2, 126.6, 126.0, 124.4, 122.5, 119.6, 119.4, 115.5, 111.8, 110.4, 108.9, 107.0, 55.8, 9.5.

IR (thin film) 3410, 3051, 2996, 2858, 1694, 1604, 1455, 1262, 732, 692 cm^{-1}.

HRMS (CI+) Calcd for $C_{28}H_{22}N_2S_2$ (M^+): 450.1224, Found: 450.1234.

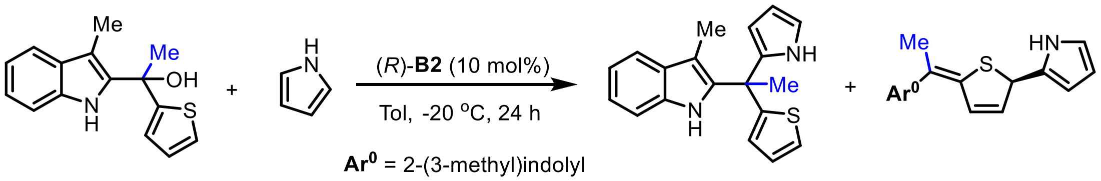

1l 0.2 mmol — **2b** 10 equiv. — **3l'** 77% yield, 84% ee — **3l**, *E*/*Z* >20:1 16% yield, 99% ee

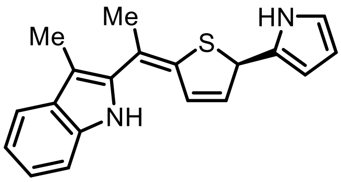

3l

(*R*,*E*)-2-(1-(5-(1*H*-Pyrrol-2-yl)thiophen-2(5*H*)-ylidene)ethyl)-3-methyl-1*H*-indole (3l) was prepared as slightly green oil from 1-(3-methyl-1*H*-indol-2-yl)-1-(thiophen-2-yl)ethan-1-ol (51.5 mg, 0.2 mmol) and pyrrole (134.0 mg, 2.0 mmol) according to the General Procedure C (-40 °C, eluent: hexanes/EtOAc = 40:1 → 20:1) in 16% yield (10.7 mg, 99% ee, *E*/*Z* > 20:1).

Additionally, an undesired product chiral triarylethane **3l'** was isolated in 77% yield (50.2 mg, 84% ee).

$[\alpha]_D^{26}$: -80.5 (c = 1.0, CH_2Cl_2). HPLC analysis of the product: Daicel CHIRALPAK AD-H column; 20% *i*-PrOH in hexanes; 1.0 mL/min; retention times: 7.8 min (major), 19.4 min (minor).

^{1}H NMR (400 MHz, acetone-d_6) δ 9.99 (s, 1H), 9.89 (s, 1H), 7.48 (d, J = 7.7 Hz, 1H), 7.31 (d, J = 8.0 Hz, 1H), 7.10-6.99 (m, 2H), 6.76-6.73 (m, 1H), 6.41-6.39 (m, 1H), 6.19-6.17 (m, 1H), 6.02-6.00 (m, 2H), 5.85-5.83 (m, 1H), 2.20 (s, 3H), 2.10 (s, 3H).

^{13}C NMR (100 MHz, acetone-d_6) δ 145.1, 137.2, 136.8, 136.4, 131.5, 130.9, 130.1, 122.2, 119.5, 119.2, 119.1, 115.6, 111.5, 108.7, 108.2, 107.0, 53.7, 22.5, 10.1.

IR (thin film) 3395, 3050, 2960, 1607, 1453, 1255, 1088, 1021, 794, 731 cm^{-1}.

HRMS (CI+) Calcd for $C_{19}H_{18}N_2S$ (M^+): 306.1191, Found: 306.1176.

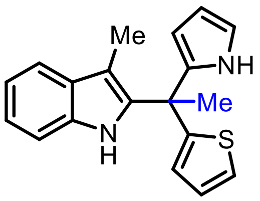

3l'

2-(1-(1*H*-Pyrrol-2-yl)-1-(thiophen-2-yl)ethyl)-3-methyl-1*H*-indole (3l').

$[\alpha]_D^{26}$: -3.2 (c = 1.0, CH_2Cl_2). HPLC analysis of the product: Daicel CHIRALPAK OD-H column; 20% *i*-PrOH in hexanes; 1.0 mL/min; retention times: 6.9 min (major), 13.5 min (minor).

^{1}H NMR (400 MHz, acetone-d_6) δ 9.75 (s, 1H), 9.42 (s, 1H), 7.45 (d, J = 7.7 Hz, 1H), 7.32-7.28 (m, 2H), 7.08-6.94 (m, 3H), 6.88-6.86 (m, 1H), 6.76-6.74 (m, 1H), 6.04-6.02 (m, 1H), 5.89-5.87 (m, 1H), 2.26 (s, 3H), 1.80 (s, 3H).

^{13}C NMR (100 MHz, acetone-d_6) δ 153.1, 140.0, 137.8, 135.8, 130.9, 127.2, 125.8, 125.1, 121.9, 119.3, 118.8, 118.3, 111.7, 108.0, 107.9, 106.9, 43.8, 30.1, 8.9.

IR (thin film) 3422, 3055, 2973, 2923, 1551, 1455, 1239, 1094, 1026, 801, 715 cm^{-1}.

HRMS (CI+) Calcd for $C_{19}H_{18}N_2S$ (M^+): 306.1191, Found: 306.1179.

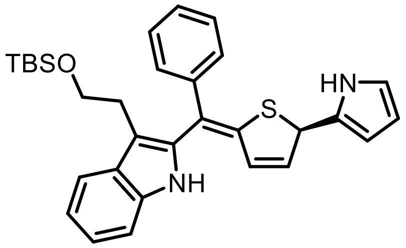

3m

(*R*,*E*)-2-((5-(1*H*-Pyrrol-2-yl)thiophen-2(5*H*)-ylidene)(phenyl)methyl)-3-(2-((*tert*-butyldimethylsilyl)oxy)ethyl)-1*H*-indole (3m) was prepared as slightly purple foam from (3-(2-((*tert*-butyldimethylsilyl)oxy)ethyl)-1*H*-indol-2-yl)(phenyl)(thiophen-2-yl)methanol (92.7 mg, 0.2 mmol) and pyrrole (134.0 mg, 2.0 mmol) according to the General Procedure C (RT, 48 h, eluent: hexanes/EtOAc = 40:1 → 20:1) in 52% yield (53.1 mg, 96% ee, *E*/*Z* > 20:1). In addition, 37.2 mg (40%) of starting material was recovered.

$[\alpha]_D^{26}$: +141.7 (c = 1.0, CH_2Cl_2). HPLC analysis of the product: Daicel CHIRALPAK AD-H column; 10% *i*-PrOH in hexanes; 1.0 mL/min; retention times: 6.6 min (major), 9.9 min (minor).

^{1}H NMR (400 MHz, acetone-d_6) δ 10.00 (s, 2H), 7.62 (d, J = 7.8 Hz, 1H), 7.42-7.28 (m, 5H), 7.22-7.18 (m, 1H), 7.13-7.03 (m, 2H), 6.77-6.74 (m, 1H), 6.45 (dd, J_1 = 2.2 Hz, J_2 = 6.2 Hz, 1H), 6.33 (dd, J_1 = 3.0 Hz, J_2 = 6.2 Hz, 1H), 6.05-6.01 (m, 2H), 5.91 (t, J = 2.5 Hz, 1H), 3.76 (t, J = 7.8 Hz, 2H), 2.88-2.83 (m, 2H), 0.86 (s, 9H), 0.00 (s, 3H), -0.01 (s, 3H).

^{13}C NMR (100 MHz, acetone-d_6) δ 147.6, 141.9, 137.8, 137.2, 135.9, 133.1, 130.3, 129.4, 129.1, 128.9, 127.8, 122.4, 121.3, 119.68, 119.66, 119.2, 111.8, 111.7, 108.8, 107.2, 64.1, 54.3, 29.7, 26.4, 18.9, -5.0.

IR (thin film) 3408, 3049, 2947, 1587, 1441, 1256, 1080, 1020, 791, 720 cm^{-1}.

HRMS (CI+) Calcd for $C_{31}H_{36}N_2OSSi$ (M^+): 512.2318, Found: 512.2325.

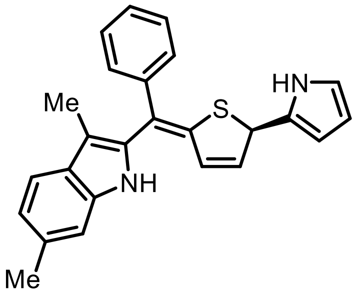

3n

(*R*,*E*)-2-((5-(1*H*-Pyrrol-2-yl)thiophen-2(5*H*)-ylidene)(phenyl)methyl)-3,6-dimethyl-1*H*-indole (3n) was prepared as slightly purple foam from (3,6-dimethyl-1*H*-indol-2-yl)(phenyl)(thiophen-2-yl)methanol (66.6 mg, 0.2 mmol) and pyrrole (134.0 mg, 2.0 mmol) according to the General Procedure C (eluent: hexanes/EtOAc = 40:1 → 20:1) in 89% yield (68.1 mg, 86% ee, *E*/*Z* > 20:1).
$[\alpha]_D^{26}$: +79.8 (c = 1.0, CH_2Cl_2). HPLC analysis of the product: Daicel CHIRALPAK AD-H column; 20% *i*-PrOH in hexanes; 1.0 mL/min; retention times: 8.4 min (major), 19.9 min (minor).
^{1}H NMR (400 MHz, acetone-d_6) δ 10.02 (s, 1H), 9.65 (s, 1H), 7.43-7.17 (m, 6H), 7.13 (s, 1H), 6.90 (dd, J_1 = 0.8 Hz, J_2 = 8.0 Hz, 1H), 7.67-7.65 (m, 1H), 6.48 (dd, J_1 = 2.2 Hz, J_2 = 6.2 Hz, 1H), 6.31 (dd, J_1 = 3.0 Hz, J_2 = 6.2 Hz, 1H), 6.05-6.01 (m, 2H), 5.89 (t, J = 2.5 Hz, 1H), 2.41 (s, 3H), 2.14 (s, 3H).
^{13}C NMR (100 MHz, acetone-d_6) δ 146.9, 142.0, 137.7, 137.6, 134.7, 133.1, 131.8, 130.3, 129.3, 128.9, 127.75, 127.73, 121.6, 121.2, 119.3, 119.0, 111.6, 110.5, 108.7, 107.2, 54.1, 21.9, 9.8.
IR (thin film) 3391, 3039, 2916, 1577, 1446, 1324, 1258, 1087, 1020, 793, 724 cm^{-1}.
HRMS (CI+) Calcd for $C_{25}H_{22}N_2S$ (M^+): 382.1504, Found: 382.1516.

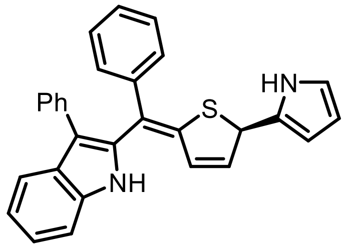

3o

(*R*,*E*)-((5-(1*H*-Pyrrol-2-yl)thiophen-2(5*H*)-ylidene)(phenyl)methyl)-3-phenyl-1*H*-indole (3o) was prepared as yellowish foam from

phenyl(3-phenyl-1*H*-indol-2-yl)(thiophen-2-yl)methanol (76.3 mg, 0.2 mmol) and pyrrole (134.0 mg, 2.0 mmol) according to the General Procedure C (eluent: hexanes/EtOAc = 40:1 → 20:1) in 93% yield (80.3 mg, 86% ee, *E*/*Z* > 20:1).

$[\alpha]_D^{26}$: -215.0 (*c* = 1.0, CH_2Cl_2). HPLC analysis of the product: Daicel CHIRALPAK AD-H column; 20% *i*-PrOH in hexanes; 1.0 mL/min; retention times: 7.2 min (major), 18.6 min (minor).

^{1}H NMR (400 MHz, acetone-d_6) δ 10.27 (s, 1H), 9.28 (s, 1H), 7.80 (d, *J* = 7.9 Hz, 1H), 7.59 (d, *J* = 7.6 Hz, 2H), 7.48-737 (m, 5H), 7.32-7.13 (m, 6H), 6.73-6.71 (m, 1H), 6.37-6.34 (m, 1H), 6.01-5.97 (m, 2H), 5.90-5.87 (m, 1H), 5.71-5.69 (m, 1H).

^{13}C NMR (100 MHz, acetone-d_6) δ 148.0, 141.7, 137.4, 137.3, 136.8, 135.4, 133.0, 129.8, 129.6, 129.3, 129.2, 129.0, 127.85, 127.84, 126.3, 123.0, 120.68, 120.65, 119.8, 119.1, 117.5, 112.1, 108.7, 107.2, 53.9.

IR (thin film) 3414, 3051, 2965, 1596, 1549, 1441, 1528, 1084, 1019, 792, 733, 699 cm^{-1}.

HRMS (CI+) Calcd for $C_{29}H_{22}N_2S$ (M^+): 430.1504, Found: 430.1499.

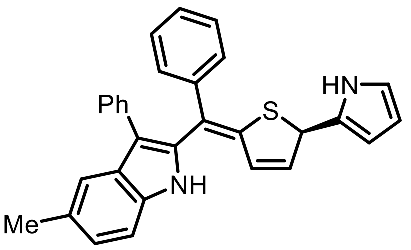

3p

(*R*,*E*)-2-((5-(1*H*-Pyrrol-2-yl)thiophen-2(5*H*)-ylidene)(phenyl)methyl)-5-methyl-3-phenyl-1*H*-indole (3p) was prepared as slightly yellowish foam from (5-methyl-3-phenyl-1*H*-indol-2-yl)(phenyl)(thiophen-2-yl)methanol (79.1 mg, 0.2 mmol) and pyrrole (134.0 mg, 2.0 mmol) according to the General Procedure C (eluent: hexanes/EtOAc = 40:1 → 20:1) in 93% yield (82.6 mg, 83% ee, *E*/*Z* > 20:1).

$[\alpha]_D^{26}$: -240.9 (*c* = 1.0, CH_2Cl_2). HPLC analysis of the product: Daicel CHIRALPAK AD-H column; 20% *i*-PrOH in hexanes; 1.0 mL/min; retention times: 6.5 min (major), 16.5 min (minor).

^{1}H NMR (400 MHz, acetone-d_6) δ 10.13 (s, 1H), 9.26 (s, 1H), 7.59-7.56 (m, 3H), 7.46-7.16 (m, 9H), 7.03 (dd, J_1 = 1.2 Hz, J_2 = 8.2 Hz, 1H), 6.72-6.70 (m, 1H), 6.35 (dd, J_1 = 2.1 Hz, J_2 = 6.2 Hz, 1H), 5.99-5.96 (m, 2H), 5.88-5.86 (m, 1H), 5.69 (t, *J* =

2.5 Hz, 1H), 2.44 (s, 3H).

^{13}C NMR (100 MHz, acetone-*d$_6$*) δ 147.8, 141.8, 137.1, 137.0, 135.8, 135.5, 133.0, 129.8, 129.7, 129.5, 129.3, 129.2, 129.0, 128.1, 127.8, 126.2, 124.6, 120.9, 119.4, 119.1, 117.1, 111.9, 108.7, 107.2, 53.9, 21.7.

IR (thin film) 3412, 3042, 2919, 1591, 1478, 1259, 1139, 1018, 721 cm^{-1}.

HRMS (CI+) Calcd for $C_{30}H_{24}N_2S$ (M^+): 444.1660, Found: 444.1654.

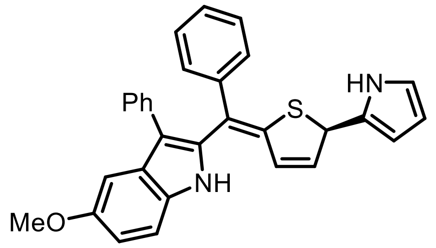

3q

(*R*,*E*)-2-((5-(1*H*-Pyrrol-2-yl)thiophen-2(5*H*)-ylidene)(phenyl)methyl)-5-methoxy-3-phenyl-1*H*-indole (3q) was prepared as slightly yellowish foam from (5-methoxy-3-phenyl-1*H*-indol-2-yl)(phenyl)(thiophen-2-yl)methanol (82.3 mg, 0.2 mmol) and pyrrole (134.0 mg, 2.0 mmol) according to the General Procedure C (eluent: hexanes/EtOAc = 40:1 → 20:1) in 95% yield (87.7 mg, 86% ee, *E*/*Z* > 20:1). $[\alpha]_D^{26}$: -214.4 (*c* = 1.0, CH_2Cl_2). HPLC analysis of the product: Daicel CHIRALPAK AD-H column; 20% *i*-PrOH in hexanes; 1.0 mL/min; retention times: 7.6 min (major), 15.6 min (minor).

^{1}H NMR (400 MHz, acetone-*d$_6$*) δ 10.13 (s, 1H), 9.27 (s, 1H), 7.59-7.57 (m, 2H), 7.46-7.44 (m, 2H), 7.39-7.27 (m, 6H), 7.25-7.16 (m, 2H), 6.87 (dd, J_1 = 2.4 Hz, J_2 = 8.8 Hz, 1H), 6.72-6.70 (m, 1H), 6.35 (dd, J_1 = 2.1 Hz, J_2 = 6.1 Hz, 1H), 6.00-5.96 (m, 2H), 5.89-5.87 (m, 1H), 5.69 (t, J = 2.5 Hz, 1H), 3.81 (s, 3H).

^{13}C NMR (100 MHz, acetone-*d$_6$*) δ 155.6, 147.8, 141.8, 137.2, 137.0, 136.1, 133.0, 132.5, 129.8, 129.5, 129.3, 129.2, 129.0, 128.2, 127.8, 127.7, 126.3, 120.9, 119.1, 117.4, 113.2, 112.9, 108.7, 107.1, 55.9, 53.9.

IR (thin film) 3416, 3049, 1592, 1478, 1440, 1260, 1138, 1090, 1020, 793, 723 cm^{-1}.

HRMS (CI+) Calcd for $C_{30}H_{24}N_2OS$ (M^+): 460.1609, Found: 460.1620.

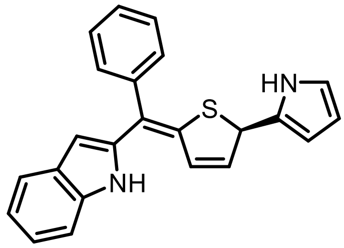

3r

(*R*,*E*)-2-((5-(1*H*-Pyrrol-2-yl)thiophen-2(5*H*)-ylidene)(phenyl)methyl)-1*H*-indole (3r) was prepared as slightly yellowish foam from (1*H*-indol-2-yl)(phenyl)(thiophen-2-yl)methanol (61.0 mg, 0.2 mmol) and pyrrole (134.0 mg, 2.0 mmol) according to the General Procedure C (eluent: hexanes/EtOAc = 40:1 → 20:1) in 62% yield (44.1 mg, 97% ee, *E*/*Z* > 20:1).

$[\alpha]_D^{26}$: +39.4 (c = 1.0, CH_2Cl_2). HPLC analysis of the product: Daicel CHIRALPAK AD-H column; 20% *i*-PrOH in hexanes; 1.0 mL/min; retention times: 11.7 min (major), 19.7 min (minor).

^{1}H NMR (400 MHz, acetone-d_6) δ 10.04 (s, 1H), 9.93 (s, 1H), 7.56 (d, J = 7.7 Hz, 1H), 7.40-7.31 (m, 5H), 7.28-7.24 (m, 1H), 7.10-7.00 (m, 3H), 6.76-6.74 (m, 1H), 6.49-6.48 (m, 1H), 6.36 (dd, J_1 = 3.0 Hz, J_2 = 6.2 Hz, 1H), 6.04-5.99 (m, 2H), 5.85 (t, J = 2.6 Hz, 1H).

^{13}C NMR (100 MHz, acetone-d_6) δ 147.0, 142.2, 138.7, 138.5, 138.5, 137.6, 132.0, 130.3, 129.9, 129.4, 129.1, 128.2, 122.4, 120.8, 120.3, 119.3, 111.9, 108.6, 107.2, 104.2, 53.5.

IR (thin film) 3396, 3050, 2962, 1575, 1445, 1259, 1086, 1015, 793, 721 cm^{-1}.

HRMS (CI+) Calcd for $C_{23}H_{18}N_2S$ (M^+): 354.1191, Found: 354.1181.

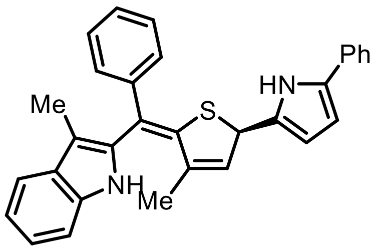

3s

(*R*,*E*)-3-Methyl-2-((3-methyl-5-(5-phenyl-1*H*-pyrrol-2-yl)thiophen-2(5*H*)-ylidene) (phenyl)methyl)-1*H*-indole (3s) was prepared as slightly yellowish foam from

(3-methyl-1*H*-indol-2-yl)(3-methylthiophen-2-yl)(phenyl)methanol (66.7 mg, 0.2 mmol) and 2-phenylpyrrole (34.3 mg, 0.24 mmol) according to the General Procedure B (-20 ºC, 48 h, eluent: hexanes/EtOAc = 40:1 → 20:1) in 81% yield (74.4 mg, 70% ee, *E*/*Z* = 8.6:1).

$[\alpha]_D^{26}$: +199.8 (c = 1.0, CH_2Cl_2). HPLC analysis of the product: Daicel CHIRALPAK OD-H column; 10% *i*-PrOH in hexanes; 1.0 mL/min; retention times: 14.8 min (major), 24.1 min (minor).

^{1}H NMR (400 MHz, acetone-d_6) δ 10.43 (s, 1H), 9.98 (s, 1H), 7.61 (d, J = 7.6 Hz, 2H), 7.53 (d, J = 7.8 Hz, 1H), 7.39-7.25 (m, 7H), 7.29-7.03 (m, 4H), 6.49-6.46 (m, 1H), 6.26-6.24 (m, 1H), 6.12-6.10 (m, 1H), 5.62-5.58 (m, 1H), 2.21 (s, 3H), 1.51 (s, 3H).

^{13}C NMR (100 MHz, acetone-d_6) δ 148.3, 143.7, 140.9, 138.4, 136.9, 134.7, 134.0, 133.3, 133.1, 131.2, 129.5, 129.4, 129.3, 128.9, 128.5, 127.8, 126.5, 124.3, 122.3, 119.5, 119.2, 111.6, 108.5, 106.8, 48.9, 15.6, 9.3.

IR (thin film) 3407, 3049, 2966, 1599, 1256, 1015, 738, 693 cm^{-1}.

HRMS (CI+) Calcd for $C_{31}H_{26}N_2S$ (M^+): 458.1817, Found: 458.1813.

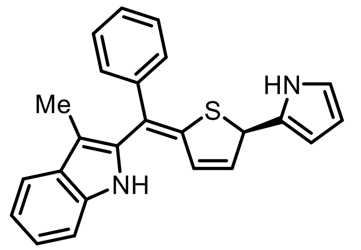

3t

(*R*,*E*)-2-((5-(1*H*-Pyrrol-2-yl)thiophen-2(5*H*)-ylidene)(phenyl)methyl)-3-methyl-1*H*-indole (3t) was prepared as slightly purple foam from (3-methyl-1*H*-indol-2-yl)(phenyl)(thiophen-2-yl)methanol (127.6 mg, 0.4 mmol) and pyrrole (268 mg, 4.0 mmol) according to the General Procedure C (8 mL of toluene used, eluent: hexanes/EtOAc = 40:1 → 20:1) in 93% yield (136.5 mg, 96% ee, *E*/*Z* >20:1).

$[\alpha]_D^{26}$: 371.5 (c = 1.0, CH_2Cl_2). HPLC analysis of the product: Daicel CHIRALPAK AD-H column; 20% *i*-PrOH in hexanes; 1.0 mL/min; retention times: 8.5 min (major), 17.0 min (minor).

^{1}H NMR (400 MHz, acetone-d_6) δ 10.02 (s, 1H), 9.82 (s, 1H), 7.56 (d, J = 7.8 Hz,

1H), 7.39-7.21 (m, 5H), 7.22-7.17 (m, 1H), 7.14-7.04 (m, 2H), 6.77-6.75 (m, 1H), 6.49 (dd, J_1 = 2.2 Hz, J_2 = 6.2 Hz, 1H), 6.32 (dd, J_1 = 3.0 Hz, J_2 = 6.2 Hz, 1H), 6.05-6.02 (m, 2H), 5.90 (t, J = 2.6 Hz, 1H), 2.17 (s, 3H).

^{13}C NMR (100 MHz, acetone-d_6) δ 147.1, 141.9, 137.8, 137.2, 135.3, 133.0, 130.2, 129.7, 129.2, 128.9, 127.8, 122.4, 121.3, 119.5, 119.3, 119.2, 111.6, 110.6, 108.7, 107.2, 54.1, 9.8.

IR (thin film) 3398, 3050, 2977, 1564, 1446, 1306, 1254, 1137, 1086, 1137, 787, 725 cm^{-1}.

HRMS (CI+) Calcd for $C_{24}H_{20}N_2S$ (M^+): 368.1347, Found: 368.1337.

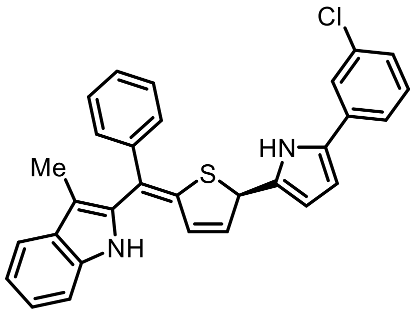

3u

(*R*,*E*)-3-Methyl-2-((5-(5-phenyl-1*H*-pyrrol-2-yl)thiophen-2(5*H*)-ylidene)(thiophen -2-yl)methyl)-1*H*-indole (3u) was prepared as slightly green foam from (3-methyl-1*H*-indol-2-yl)(phenyl)(thiophen-2-yl)methanol (63.8 mg, 0.2 mmol) and 2-(3-chlorophenyl)-1*H*-pyrrole (42.3 mg, 0.24 mmol) according to the General Procedure B (eluent: hexanes/EtOAc = 15:1 → 10:1) in 92% yield (94.6 mg, 91% ee, *E*/*Z* >20:1).

$[\alpha]_D^{26}$: +228.3 (c = 1.0, CH_2Cl_2). HPLC analysis of the product: Daicel CHIRALPAK OD-H column; 20% *i*-PrOH in hexanes; 1.0 mL/min; retention times: 8.0 min (major), 11.2 min (minor).

^{1}H NMR (400 MHz, acetone-d_6) δ 10.61 (s, 1H), 9.83 (s, 1H), 7.65 (t, J = 1.8 Hz, 1H), 7.56 (dd, J_1 = 0.7 Hz, J_2 = 7.8 Hz, 2H), 7.38-7.28 (m, 6H), 7.22-7.04 (m, 4H), 6.56 (dd, J_1 = 2.6 Hz, J_2 = 3.4 Hz, 1H), 6.53 (dd, J_1 = 2.6 Hz, J_2 = 6.6 Hz, 1H), 6.40 (dd, J_1 = 3.0 Hz, J_2 = 6.2 Hz, 1H), 6.14 (t, J = 2.3 Hz, 1H), 5.94 (t, J = 2.6 Hz, 1H), 2.18 (s, 3H).

^{13}C NMR (100 MHz, acetone-d_6) δ 147.0, 141.9, 137.2, 137.1, 135.9, 135.3, 135.1, 133.5, 133.5, 131.7, 131.2, 129.7, 129.3, 129.0, 127.8, 126.1, 123.9, 122.8, 122.5,

121.6, 119.5, 119.3, 111.6, 110.7, 109.1, 108.1, 53.8, 9.8.

IR (thin film) 3414, 3052, 2912, 2878, 1695, 1599, 1492, 1263, 1238, 1147, 1089, 770, 732, 696 cm^{-1}.

HRMS (LD+) Calcd for $C_{30}H_{22}ClN_2S$ $[(M-H)^+]$: 477.1187, Found: 477.1184.

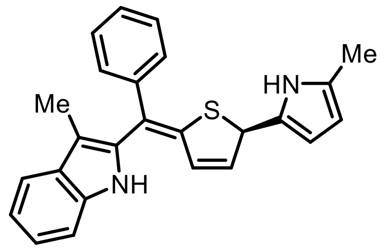

3v

(*R*,*E*)-3-Methyl-2-((5-(5-methyl-1*H*-pyrrol-2-yl)thiophen-2(5*H*)-ylidene)(phenyl) methyl)-1*H*-indole (3v) was prepared as slightly purple foam from (3-methyl-1*H*-indol-2-yl)(phenyl)(thiophen-2-yl)methanol (63.8 mg, 0.2 mmol) and 2-methylpyrrole (32.4 mg, 0.40 mmol) according to the General Procedure C (48 h, eluent: hexanes/EtOAc = 40:1 → 20:1) in 72% yield (55.2 mg, 95% ee, *E*/*Z* = 14:1). In addition, 7.2 mg (11%) of starting material was recovered.

$[\alpha]_D^{26}$: +563.8 (c = 1.0, CH_2Cl_2). HPLC analysis of the product: Daicel CHIRALPAK AD-H column; 20% *i*-PrOH in hexanes; 1.0 mL/min; retention times: 11.7 min (major), 19.7 min (minor).

^{1}H NMR (400 MHz, acetone-d_6) δ 9.80 (s, 1H), 9.72 (s, 1H), 7.55 (d, J = 7.8 Hz, 1H), 7.38-7.27 (m, 5H), 7.21-7.17 (m, 1H), 7.13-7.03 (m, 2H), 6.46 (dd, J_1 = 2.2 Hz, J_2 = 6.2 Hz, 1H), 6.30 (dd, J_1 = 3.0 Hz, J_2 = 6.2 Hz, 1H), 5.90-5.88 (m, 1H), 5.84 (t, J = 2.5 Hz, 1H), 5.70-5.68 (m, 1H), 2.20 (s, 3H), 2.16 (s, 3H).

^{13}C NMR (100 MHz, acetone-d_6) δ 147.4, 142.0, 138.0, 137.2, 135.4, 132.9, 129.8, 129.2, 129.1, 128.9, 128.6, 127.7, 122.4, 121.2, 119.5, 119.2, 111.6, 110.6, 107.5, 106.6, 54.5, 13.0, 9.8.

IR (thin film) 3393, 3048, 2959, 1586, 1444, 1260, 1130, 1023, 856, 779, 741 cm^{-1}.

HRMS (CI+) Calcd for $C_{25}H_{22}N_2S$ (M^+): 382.1504, Found: 382.1493.

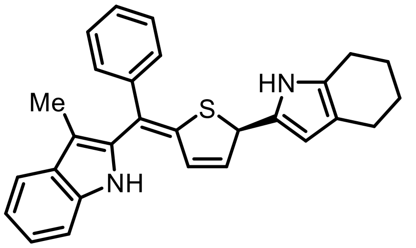

3w

(*R*,*E*)-3-Methyl-2-(phenyl(5-(4,5,6,7-tetrahydro-1*H*-indol-2-yl)thiophen-2(5*H*)-ylidene)methyl)-1*H*-indole (3w) was prepared as slightly purple foam from (3-methyl-1*H*-indol-2-yl)(phenyl)(thiophen-2-yl)methanol (63.8 mg, 0.2 mmol) and 4,5,6,7-tetrahydro-1*H*-indole (48.4 mg, 0.40 mmol) according to the General Procedure C (48 h, eluent: hexanes/EtOAc = 40:1 → 20:1) in 57% yield (48.1 mg, 94% ee, *E*/*Z* >20:1). In addition, 13.1 mg (20%) of starting material was recovered.

$[\alpha]_D^{26}$: +146.4 (c = 1.0, CH_2Cl_2). HPLC analysis of the product: Daicel CHIRALPAK AD-H column; 12% *i*-PrOH in hexanes; 1.0 mL/min; retention times: 12.8 min (major), 30.0 min (minor).

^{1}H NMR (400 MHz, acetone-d_6) δ 9.79 (s, 1H), 9.41 (s, 1H), 7.54 (d, J = 7.8 Hz, 1H), 7.37-7.27 (m, 5H), 7.21-7.17 (m, 1H), 7.12-7.02 (m, 2H), 6.44 (dd, J_1 = 2.2 Hz, J_2 = 6.2 Hz, 1H), 6.29 (dd, J_1 = 3.0 Hz, J_2 = 6.1 Hz, 1H), 5.82 (t, J = 2.5 Hz, 1H), 5.75-5.74 (m, 1H), 2.51 (t, J = 5.7 Hz, 2H), 2.51 (t, J = 6.0 Hz, 2H), 2.16 (s, 3H), 1.77-1.65 (m, 4H).

^{13}C NMR (100 MHz, acetone-d_6) δ 147.5, 142.0, 138.1, 137.2, 135.4, 132.8, 129.8, 129.3, 128.9, 128.3, 128.1, 127.7, 122.4, 121.1, 119.5, 119.2, 117.1, 111.6, 110.5, 106.5, 54.7, 24.7, 24.2, 23.7, 23.4, 9.8.

IR (thin film) 3395, 3052, 2923, 1591, 1443, 1257, 1083, 1015, 798, 730 cm^{-1}.

HRMS (CI+) Calcd for $C_{28}H_{26}N_2S$ (M^+): 422.1817, Found: 422.1810.

Note: With 5-methylthiophenyl-substituted alcohol (**1t**), the thiophene dearomatization product **3x** was not observed. Instead, the nucleophilic addition to the indole benzylic position by 1,6-addition to the indole imine methide was observed, leading to the enantioenriched tetraarylmethane **3x'**. The results are as shown below.

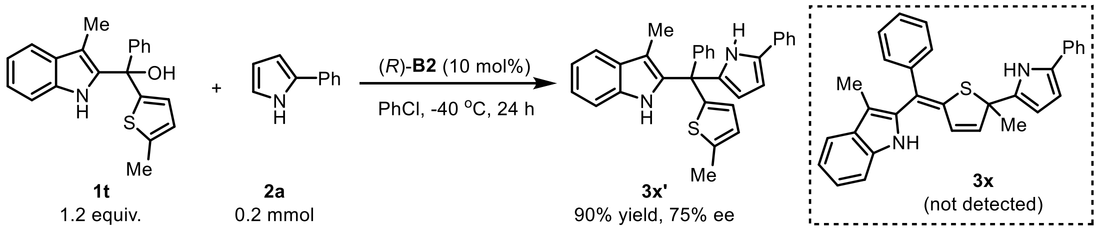

3-Methyl-2-((5-methylthiophen-2-yl)(phenyl)(5-phenyl-*1H*-pyrrol-2-yl)methyl)-1 H-indole (3x') was prepared as yellowish foam from (3-methyl-1*H*-indol-2-yl)(5-methylthiophen-2-yl)(phenyl)methanol **1t** (79.9 mg, 0.24 mmol) and 2-phenylpyrrole (28.6 mg, 0.2 mmol) according to the General Procedure B (eluent: hexanes/EtOAc = 15:1 → 10:1) for 24 h in 90% yield (82.6 mg, 75% ee). $[\alpha]_D^{26}$: +245.5 (*c* = 1.0, CH_2Cl_2). HPLC analysis of the product: Daicel CHIRALPAK AD-H column; 15% *i*-PrOH in hexanes; 1.0 mL/min; retention times: 9.4 min (minor), 15.0 min (major).

^{1}H NMR (400 MHz, acetone-*d$_6$*) *δ* 10.16 (s, 1H), 9.83 (s, 1H), 7.63 (d, *J* = 7.8 Hz, 2H), 7.59 (d, *J* = 7.8 Hz, 1H), 7.38-7.21 (m, 7H), 7.21-7.07 (m, 4H), 6.46-6.37 (m, 3H), 6.16-6.14 (m, 1H), 2.21 (s, 3H), 2.03 (s, 3H).

^{13}C NMR (100 MHz, acetone-*d$_6$*) *δ* 146.2, 143.1, 141.8, 137.2, 136.8, 135.1, 133.9, 133.5, 130.8, 129.7, 129.4, 129.3, 128.9, 128.8, 126.5, 124.6, 122.4, 121.7, 119.5, 119.2, 111.6, 110.8, 107.6, 106.6, 63.3, 29.5, 9.8.

IR (thin film) 3418, 3046, 2967, 1593, 1447, 1245, 1096, 1032, 742, 692 cm^{-1}.

HRMS (CI+) Calcd for $C_{31}H_{26}N_2S$ (M^+): 458.1817, Found: 458.1808.

General Procedure D.

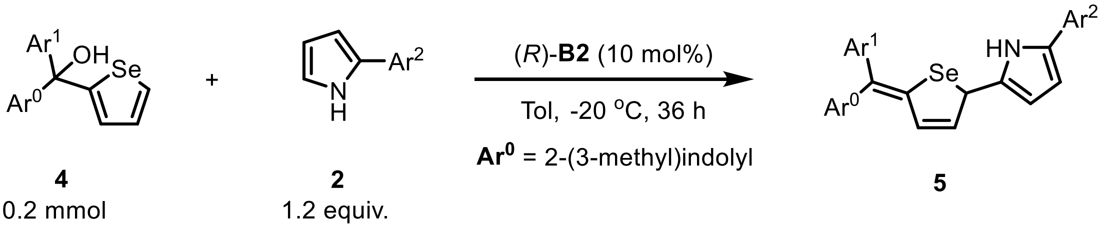

At -20 °C, to an oven-dried 8-mL vial charged with a solution of the tertiary alcohol **4** (0.2 mmol) and pyrrole **2** (0.24 mmol) in toluene (3.6 mL) was slowly added a solution of the catalyst (*R*)-**B2** (20 mg, 0.015 mmol, 10 mol%) in toluene (0.4 mL). The reaction mixture was stirred at the same temperature for 36 h. After that,

triethylamine (2 drops) was added to quench the reaction. The mixture was concentrated under reduced pressure and purified by silica gel (deactivated by triethylamine) flash chromatography to afford the desired product

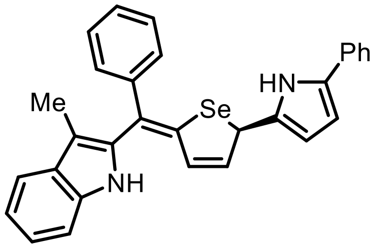

5a

(*R*,*E*)-3-Methyl-2-(phenyl(5-(5-phenyl-1*H*-pyrrol-2-yl)selenophen-2(5*H*)-ylidene) methyl)-1*H*-indole (5a) was prepared as slightly yellow-brown foam from (3-methyl-1*H*-indol-2-yl)(phenyl)(selenophen-2-yl)methanol (73.2 mg, 0.2 mmol) and 2-phenylpyrrole (34.3 mg, 0.24 mmol) according to the General Procedure D (eluent: hexanes/EtOAc = 15:1 → 10:1) in 95% yield (93.5 mg, 94% ee, *E*/*Z* >20:1).

$[\alpha]_D^{26}$: +87.5 (c = 1.0, CH_2Cl_2). HPLC analysis of the product: Daicel CHIRALPAK OD-H column; 10% *i*-PrOH in hexanes; 1.0 mL/min; retention times: 24.7 min (major), 34.8 min (minor).

^{1}H NMR (400 MHz, acetone-d_6) δ 10.48 (s, 1H), 9.85 (s, 1H), 7.61 (dd, J_1 = 1.2 Hz, J_2 = 8.5 Hz, 2H), 7.58 (d, J = 7.9 Hz, 1H), 7.39-7.05 (m, 11H), 6.63 (dd, J_1 = 2.2 Hz, J_2 = 6.6 Hz, 1H), 6.47 (t, J = 3.2 Hz, 1H), 6.30 (dd, J_1 = 3.1 Hz, J_2 = 6.6 Hz, 1H), 6.14 (t, J = 2.6 Hz, 1H), 6.10 (t, J = 2.8 Hz, 1H), 2.20 (s, 3H).

^{13}C NMR (100 MHz, acetone-d_6) δ 145.3, 143.3, 138.0, 137.2, 135.4, 135.3, 133.9, 133.9, 133.1, 129.7, 129.5, 129.1, 128.7, 128.1, 126.4, 126.3, 124.3, 122.5, 119.5, 119.2, 111.6, 110.7, 108.6, 107.0, 47.2, 9.9.

IR (thin film) 3418, 3076, 2920, 2854, 1682, 1548, 1505, 1296, 1264, 1187, 1122, 770, 733, 693 cm^{-1}.

HRMS (CI+) Calcd for $C_{30}H_{24}N_2Se$ (M^+): 492.1105, Found: 492.1121.

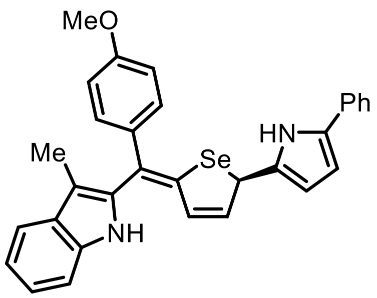

5b

(*R*,*E*)-2-((4-Methoxyphenyl)(5-(5-phenyl-1*H*-pyrrol-2-yl)selenophen-2(5*H*)-yliden e)methyl)-3-methyl-1*H*-indole (5b) was prepared as slightly yellow-brown foam from (4-methoxyphenyl)(3-methyl-1*H*-indol-2-yl)- (selenophen-2-yl)-methanol (78.9 mg, 0.2 mmol) and 2-phenylpyrrole (34.3 mg, 0.24 mmol) according to the General Procedure D (eluent: hexanes/EtOAc = 15:1 → 10:1) in 94% yield (97.2 mg, 98% ee, *E*/*Z* >20:1).

$[\alpha]_D^{26}$: +154.9 (c = 1.0, CH_2Cl_2). HPLC analysis of the product: Daicel CHIRALPAK OD-H column; 20% *i*-PrOH in hexanes; 1.0 mL/min; retention times: 16.7 min (major), 21.2 min (minor).

^{1}H NMR (400 MHz, acetone-d_6) δ 10.45 (s, 1H), 9.79 (s, 1H), 7.63-7.57 (m, 3H), 7.36-7.29 (m, 5H), 7.16-7.06 (m, 3H), 6.86 (d, J = 8.9 Hz, 2H), 6.61 (dd, J_1 = 2.2 Hz, J_2 = 6.6 Hz, 1H), 6.48 (dd, J_1 = 2.8 Hz, J_2 = 3.3 Hz, 1H), 6.25 (dd, J_1 = 3.1 Hz, J_2 = 6.6 Hz, 1H), 6.13 (t, J = 2.5 Hz, 1H), 6.11 (t, J = 2.7 Hz, 1H), 3.75 (s, 3H), 2.20 (s, 3H).

^{13}C NMR (100 MHz, acetone-d_6) δ 159.7, 143.7, 137.2, 137.2, 135.6, 135.5, 135.4, 134.1, 133.9, 133.1, 130.1, 129.7, 129.5, 126.4, 126.0, 124.3, 122.4, 119.5, 119.2, 114.4, 111.6, 110.6, 108.6, 107.0, 55.5, 47.1, 9.9.

IR (thin film) 3441, 3049, 2930, 2814, 1604, 1506, 1243, 1175, 756, 733, 701 cm^{-1}.

HRMS (CI+) Calcd for $C_{31}H_{26}N_2OSe$ (M^+): 522.1210, Found: 522.1207.

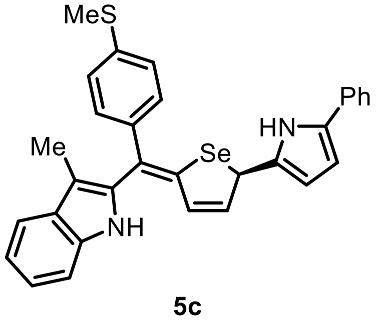

5c

(R,E)-3-Methyl-2-((4-(methylthio)phenyl)(5-(5-phenyl-1H-pyrrol-2-yl)selenophen-2(5H)-ylidene)methyl)-1H-indole (5c) was prepared as slightly yellow-brown foam from (3-methyl-1H-indol-2-yl)(4-(methylthio)phenyl) (selenophen-2-yl)methanol (82.6 mg, 0.2 mmol) and 2-phenylpyrrole (34.3 mg, 0.24 mmol) according to the General Procedure D (eluent: hexanes/EtOAc = 15:1 → 10:1) in 90% yield (96.6 mg, 96% ee, E/Z >20:1).

$[\alpha]_D^{26}$: +357.0 (c = 1.0, CH_2Cl_2). HPLC analysis of the product: Daicel CHIRALPAK OD-H column; 20% i-PrOH in hexanes; 1.0 mL/min; retention times: 17.3 min (major), 21.2 min (minor).

^{1}H NMR (400 MHz, acetone-d_6) δ 10.45 (s, 1H), 9.83 (s, 1H), 7.61 (d, J = 7.4 Hz, 2H), 7.57 (d, J = 7.7 Hz, 1H), 7.34-7.28 (m, 5H), 7.20-7.05 (m, 5H), 6.61 (dd, J_1 = 2.2 Hz, J_2 = 6.6 Hz, 1H), 6.47 (t, J = 2.6 Hz, 1H), 6.29 (dd, J_1 = 3.1 Hz, J_2 = 6.6 Hz, 1H), 6.15 (t, J = 2.5 Hz, 1H), 6.10 (t, J = 2.2 Hz, 1H), 2.44 (s, 3H), 2.19 (s, 3H).

^{13}C NMR (100 MHz, acetone-d_6) δ 144.8, 139.7, 138.7, 137.9, 137.2, 135.5, 135.2, 133.9, 133.8, 133.1, 129.7, 129.5, 129.3, 126.5, 126.5, 125.7, 124.3, 122.5, 119.6, 119.3, 111.6, 110.8, 108.7, 107.0, 47.5, 15.1, 9.9.

IR (thin film) 3439, 3046, 2918, 2881, 1605, 1508, 1489, 1455, 1264, 1238, 1123, 756, 735, 695 cm^{-1}.

HRMS (LD+) Calcd for $C_{31}H_{25}N_2SSe$ [(M-H)$^+$]: 537.0898, Found: 537.0929.

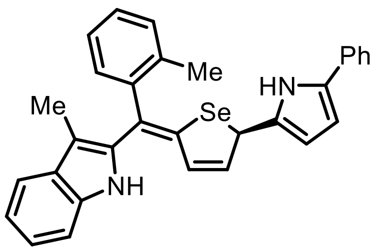

5d

(R,E)-3-Methyl-2-((5-(5-phenyl-1H-pyrrol-2-yl)selenophen-2(5H)-ylidene)(o-tolyl)methyl)-1H-indole (5d) was prepared as slightly yellow-green foam from (3-methyl-1H-indol-2-yl)(selenophen-2-yl)(o-tolyl)methanol (76.0 mg, 0.2 mmol) and pyrrole (34.3 mg, 2.0 mmol) according to the General Procedure D (eluent: hexanes/EtOAc = 15:1 → 10:1) in 89% yield (90.1 mg, 97% ee, E/Z >20:1).

$[\alpha]_D^{26}$: +63.1 (c = 1.0, CH_2Cl_2). HPLC analysis of the product: Daicel CHIRALPAK OD-H column; 20% i-PrOH in hexanes; 1.0 mL/min; retention times: 9.8 min (major),

11.6 min (minor).

^{1}H NMR (400 MHz, acetone-d_6) δ 10.40 (s, 1H), 9.64 (s, 1H), 7.60-7.44 (m, 4H), 7.34-7.02 (m, 9H), 6.82-6.81 (m, 1H), 6.47 (t, J = 2.6 Hz, 1H), 6.37 (dd, J_1 = 3.0 Hz, J_2 = 6.4 Hz, 1H), 6.10-6.06 (m, 2H), 2.25 (s, 3H), 2.10 (s, 3H).

^{13}C NMR (100 MHz, acetone-d_6) δ 146.2, 143.1, 139.0, 137.3, 137.2, 134.7, 134.3, 134.1, 133.9, 133.0, 131.4, 130.1, 130.0, 129.5, 128.9, 126.8, 126.4, 125.7, 124.3, 122.5, 119.6, 119.2, 111.6, 109.7, 108.5 107.0, 46.6, 19.6, 10.4.

IR (thin film) 3438, 3054, 2916, 1605, 1508, 1455, 1298, 1264, 1123, 1040, 755, 734, 693 cm^{-1}.

HRMS (CI+) Calcd for $C_{31}H_{26}N_2Se$ (M^+): 506.1261, Found: 506.1277.

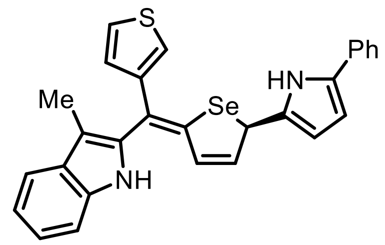

5e

(*R*,*E*)-3-Methyl-2-((5-(5-phenyl-1*H*-pyrrol-2-yl)selenophen-2(5*H*)-ylidene)(thiophen-3-yl)methyl)-1*H*-indole (5e) was prepared as slightly yellowish foam from (3-methyl-1*H*-indol-2-yl)(selenophen-2-yl)(thiophen-3-yl)methanol (74.4 mg, 0.2 mmol) and 2-phenylpyrrole (34.3 mg, 0.24 mmol) according to the General Procedure D (eluent: hexanes/EtOAc = 15:1 → 10:1) in 92% yield (91.6 mg, 98% ee, *E*/*Z* >20:1).

$[\alpha]_D^{26}$: +120.7 (c = 1.0, CH_2Cl_2). HPLC analysis of the product: Daicel CHIRALPAK OD-H column; 20% *i*-PrOH in hexanes; 1.0 mL/min; retention times: 14.7 min (major), 18.8 min (minor).

^{1}H NMR (400 MHz, acetone-d_6) δ 10.47 (s, 1H), 9.91 (s, 1H), 7.62-7.56 (m, 3H), 7.39-7.30 (m, 5H), 7.17-7.05 (m, 3H), 6.99 (dd, J_1 = 1.4 Hz, J_2 = 5.0 Hz, 1H), 6.55 (dd, J_1 = 2.2 Hz, J_2 = 6.6 Hz, 1H), 6.47 (t, J = 2.8 Hz, 1H), 6.27 (dd, J_1 = 3.1 Hz, J_2 = 6.6 Hz, 1H), 6.16 (t, J = 2.6 Hz, 1H), 6.09 (t, J = 2.6 Hz, 1H), 2.18 (s, 3H).

^{13}C NMR (100 MHz, acetone-d_6) δ 144.5, 144.0, 137.7, 137.0, 135.4, 135.2, 133.9, 133.7, 133.1, 129.7, 129.5, 128.6, 126.5, 126.1, 124.3, 124.0, 122.5, 121.4, 119.6, 119.3, 111.7, 110.1, 108.7, 107.0, 48.0, 9.7.

IR (thin film) 3404, 3049, 2912, 1694, 1603, 1507, 1455, 1263, 1122, 1039, 780, 731, 691 cm^{-1}.

HRMS (CI+) Calcd for $C_{28}H_{22}N_2SSe$ (M^+): 498.0669, Found: 498.0678.

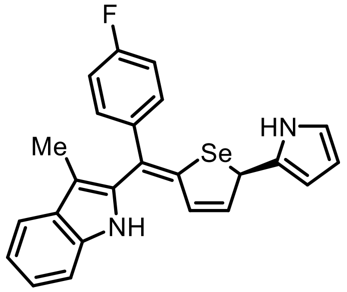

5f

(*R*,*E*)-2-((5-(1*H*-pyrrol-2-yl)selenophen-2(5*H*)-ylidene)(4-fluorophenyl)methyl)-3-methyl-1*H*-indole (5f) was prepared as slightly yellow-brown foam from (4-fluorophenyl)(3-methyl-1*H*-indol-2-yl)(selenophen-2-yl)methanol (76.9 mg, 0.2 mmol) and pyrrole (134 mg, 2.0 mmol) according to the General Procedure D (24 h, eluent: hexanes/EtOAc = 30:1 → 20:1) in 57% yield (49.6 mg, 92% ee, *E*/*Z* >20:1). $[\alpha]_D^{26}$: +34.2 (c = 1.0, CH_2Cl_2). HPLC analysis of the product: Daicel CHIRALPAK OD-H column; 20% *i*-PrOH in hexanes; 1.0 mL/min; retention times: 10.2 min (major), 21.1 min (minor).

^{1}H NMR (400 MHz, acetone-d_6) δ 9.88 (s, 1H), 9.86 (s, 1H), 7.55 (d, J = 7.9 Hz, 1H), 7.40-7.36 (m, 2H), 7.31 (d, J = 8.0 Hz, 1H), 7.14-7.03 (m, 4H), 6.76-6.74 (m, 1H), 6.57 (dd, J_1 = 2.2 Hz, J_2 = 6.5 Hz, 1H), 6.25 (dd, J_1 = 3.1 Hz, J_2 = 6.6 Hz, 1H), 6.12 (t, J = 2.6 Hz, 1H), 6.03-5.99 (m, 2H), 2.16 (s, 3H).

^{13}C NMR (100 MHz, acetone-d_6) δ 162.5 (d, J = 241.9 Hz), 145.5, 139.7 (d, J = 3.5 Hz), 138.9, 137.3, 135.1, 134.9, 131.5, 130.9 (d, J = 8.0 Hz), 129.7, 125.2, 122.6, 119.6, 119.3, 119.2, 115.9 (d, J = 21.6 Hz), 111.7, 110.7, 108.8, 107.0, 47.7, 9.8.

^{19}F NMR (376 MHz, acetone-d_6) δ -114.9.

IR (thin film) 3393, 3049, 2963, 1595, 1501, 1258, 1226, 1087, 1019, 795, 729 cm^{-1}.

HRMS (LD+) Calcd for $C_{24}H_{19}FN_2Se$ [M^+]: 434.0697, Found: 434.0715.

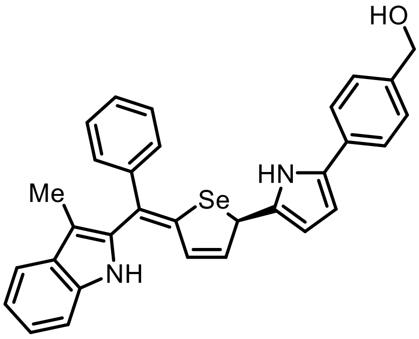

5g

***(R,E)*-(4-(5-(5-((3-Methyl-1*H*-indol-2-yl)(phenyl)methylene)-2,5-dihydroselenophen-2-yl)-1*H*-pyrrol-2-yl)phenyl)methanol (5g)** was prepared as slightly yellowish foam from (3-methyl-1*H*-indol-2-yl)(phenyl)(selenophen-2-yl) methanol (76.9 mg, 0.21 mmol) and (4-(1*H*-pyrrol-2-yl)phenyl)methanol (34.6 mg, 0.2 mmol) according to the General Procedure D (30 h, eluent: hexanes/EtOAc = 10:1 → 3:1) in 90% yield (93.9 mg, 95% ee, *E*/*Z* >20:1).

$[\alpha]_D^{26}$: +123.4 (c = 1.0, CH_2Cl_2). HPLC analysis of the product: Daicel CHIRALPAK OD-H column; 30% *i*-PrOH in hexanes; 1.0 mL/min; retention times: 15.9 min (major), 20.8 min (minor).

^{1}H NMR (400 MHz, acetone-d_6) δ 10.44 (s, 1H), 9.87 (s, 1H), 7.58-7.56 (m, 3H), 7.37-7.20 (m, 8H), 7.14-7.04 (m, 2H), 6.62 (dd, J_1 = 2.2 Hz, J_2 = 6.6 Hz, 1H), 6.45 (t, J = 3.2 Hz, 1H), 6.31 (dd, J_1 = 3.1 Hz, J_2 = 6.5 Hz, 1H), 6.13 (t, J = 2.6 Hz, 1H), 6.08 (t, J = 2.9 Hz, 1H), 4.61 (s, 2H), 2.18 (s, 3H).

^{13}C NMR (100 MHz, acetone-d_6) δ 145.4, 143.4, 140.8, 138.1, 137.3, 135.4, 135.3, 133.7, 133.2, 132.6, 129.7, 129.1, 128.8, 128.2, 127.9, 126.3, 124.2, 122.5, 119.6, 119.3, 111.7, 110.7, 108.6, 106.8, 64.5, 47.3, 9.9.

IR (thin film) 3540, 3413, 3329, 3031, 2915, 1613, 1457, 1263, 768, 732, 698 cm^{-1}.

HRMS (LD+) Calcd for $C_{31}H_{25}N_2OSe$ $[(M-H)^+]$: 521.1127, Found: 521.1109.

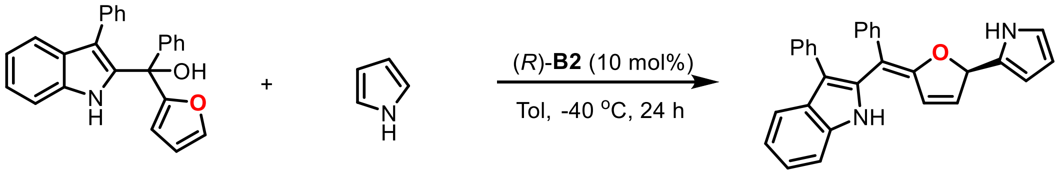

6 2b 7

***(R,E)*-2-((5-(1*H*-pyrrol-2-yl)furan-2(5*H*)-ylidene)(phenyl)methyl)-3-phenyl-1*H*-indole (7)** was prepared as pink foam from

furan-2-yl(phenyl)(3-phenyl-1*H*-indol-2-yl)methanol (73.0 mg, 0.2 mmol) and pyrrole (134.0 mg, 2.0 mmol) according to the General Procedure C (-40 °C, eluent: hexanes/EtOAc = 40:1 → 20:1) in 92% yield (76.5 mg, 92% ee, *E*/*Z* = 6.7:1).

$[\alpha]_D^{26}$: -7.1 (*c* = 1.0, CH_2Cl_2). HPLC analysis of the product: Daicel CHIRALPAK AD-H column; 20% *i*-PrOH in hexanes; 1.0 mL/min; retention times: 6.1 min (major), 8.5 min (minor).

^{1}H NMR (400 MHz, acetone-d_6) δ 10.23 (s, 1H), 9.75 (s, 1H), 7.82 (d, *J* = 7.9 Hz, 1H), 7.61-7.53 (m, 4H), 7.46 (d, *J* = 7.9 Hz, 1H), 7.37-7.33 (m, 2H), 7.23-7.06 (m, 6H), 6.81-6.79 (m, 1H), 6.31-6.26 (m, 2H), 7.03 (dd, J_1 = 2.0 Hz, J_2 = 5.8 Hz, 1H), 6.07-6.05 (m, 1H), 6.03-6.01 (m, 1H).

^{13}C NMR (100 MHz, acetone-d_6) δ 161.1, 139.3, 137.4, 137.0, 135.7, 134.8, 129.5, 129.2, 129.0, 128.6, 128.2, 128.0, 127.7, 126.3, 126.2, 122.7, 120.5, 119.9, 119.7, 117.3, 112.0, 108.9, 108.7, 102.2, 85.5.

IR (thin film) 3408, 3049, 2919, 1607, 1439, 1259, 1174, 1081, 1019, 913, 787, 727, 698 cm^{-1}.

HRMS (CI+) Calcd for $C_{29}H_{22}N_2O$ (M^+): 414.1732, Found: 414.1726.

V. Scale-up Reaction and Application

1-Mmol-scale synthesis

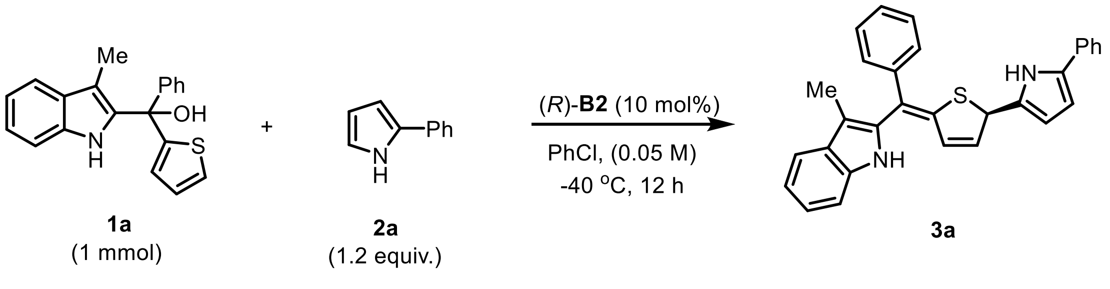

At -40 °C, to an oven-dried 50 mL flask charged with a solution of the tertiary alcohol **1a** (1 mmol, 319 mg) and 2-phenylpyrrole **2a** (0.24 mmol, 171.6 mg) in PhCl (9 mL) was slowly added a solution of catalyst (*R*)-**B2** (100 mg, 0.015 mmol, 10 mol%) in PhCl (1 mL). The reaction mixture was stirred at the same temperature for 12 h. After that, triethylamine (0.2 mL) was added to quench the reaction. The mixture was concentrated under reduced pressure and purified by silica gel (deactivated by triethylamine) flash chromatography to afford the desired product **3a** in 95% yield

(420 mg, 94% ee, *E*/*Z* >20:1) as pale green foam.

Regioselective reduction

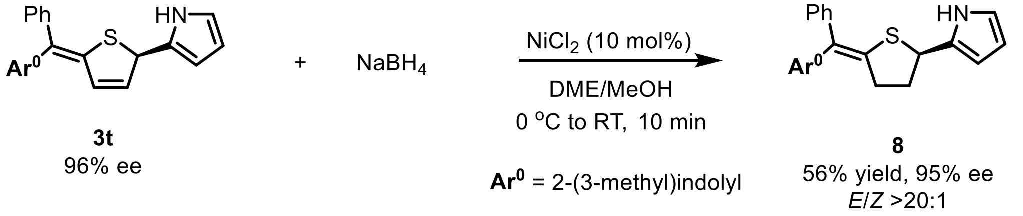

At 0 ºC, to an oven-dried 8-mL vial charged with a solution of **3t** (0.2 mmol, 73.6 mg) and $NiCl_2$ (0.02 mmol, 2.6 mg) in DME/MeOH (1 mL/1 mL) was slowly added $NaBH_4$ (76 mg, 2 mmol, 10 equiv.) in 3 portions over 5 min at room temperature with the formation of a black solid and evolution of hydrogen. After that, the reaction mixture was stirred at room temperature for 10 min. TLC showed the reaction was finished. Water (5 mL) was slowly added to quench the reaction and the final mixture was extracted with ethyl acetate (2 x 10 mL). The combined organic phase was dried, concentrated and then purified by silica gel (deactivated by triethylamine) flash chromatography (eluent: hexanes/EtOAc = 30:1 → 15:1) to afford the desired product **8** in 56% yield (41.1 mg, 95% ee, *E*/*Z* >20:1) as slightly purple foam.

$[\alpha]_D^{26}$: -98.6 (c = 1.0, CH_2Cl_2). HPLC analysis of the product: Daicel CHIRALPAK OD-H column; 20% *i*-PrOH in hexanes; 1.0 mL/min; retention times: 8.1 min (major), 11.7 min (minor).

^{1}H NMR (400 MHz, acetone-d_6) δ 10.01 (s, 1H), 9.79 (s, 1H), 7.51 (d, J = 7.8 Hz, 1H), 7.35-7.25 (m, 5H), 7.18-7.14 (m, 1H), 7.10-6.99 (m, 2H), 6.74-6.72 (m, 1H), 6.10-6.08 (m, 1H), 6.02-5.99 (m, 1H), 4.95 (dd, J_1 = 5.3 Hz, J_2 = 9.7 Hz, 1H), 2.94-2.87 (m, 1H), 2.82-2.74 (m, 1H), 2.44-2.37 (m, 1H), 2.31-2.23 (m, 1H), 2.15 (s, 3H).

^{13}C NMR (100 MHz, acetone-d_6) δ 147.2, 142.4, 137.1, 136.2, 130.9, 129.8, 129.3, 128.8, 127.3, 122.0, 120.6, 119.3, 119.1, 118.7, 111.6, 109.1, 108.5, 106.8, 49.0, 37.8, 37.5, 9.6.

IR (thin film) 3398, 3048, 2924, 1590, 1447, 1311, 1247, 1088, 1024, 791, 724 cm^{-1}.

HRMS (CI+) Calcd for $C_{24}H_{22}N_2S$ (M^+): 370.1504, Found: 370.1500.

VI. Mechanistic Experiments

Control experiments.

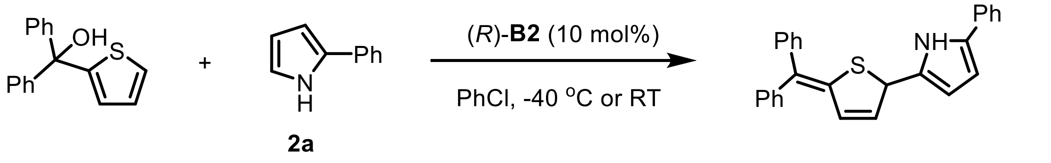

-40 °C, 72 h, NR
RT, 12 h, <5% Conv., trace pdt

At -40 °C or rt to an oven-dried 8-mL vial charged with a solution of diphenyl(thiophen-2-yl)methanol (6.7 mg, 0.025 mmol) and 2-phenylpyrrole **2a** (4.3 mg, 0.03 mmol) in PhCl (0.4 mL) was slowly added a solution of catalyst (*R*)-**B2** (2.5 mg, 0.015 mmol, 10 mol%) in PhCl (0.1 mL). The reaction mixture was stirred for 12 h at room temperature or for 72 h at -40 °C. After that, the reaction mixture was filtered through a short pad of silica gel and then concentrated. The crude mixture was examined by NMR and the ee value of the product was determined by HPLC. For the reaction at room temperature, crude NMR showed <5% conversion and <3% yield. For the reaction at -40 °C, crude ^{1}H NMR showed no reaction at all.

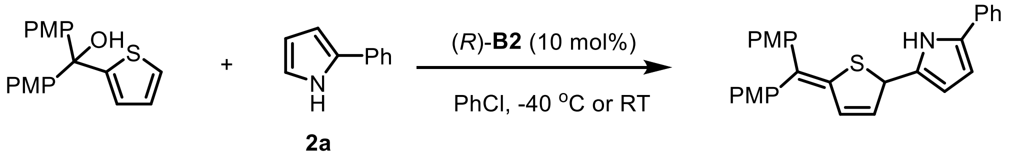

-40 °C, 72 h, 100% Conv., 75% yield, 16% ee
RT, 12 h, 100% Conv., 75% yield, 2% ee

At -40 °C or rt to an oven-dried 8-mL vial charged with a solution of bis(4-methoxyphenyl)(thiophen-2-yl)methanol (8.2 mg, 0.025 mmol) and 2-phenylpyrrole **2a** (4.3 mg, 0.03 mmol) in PhCl (0.4 mL) was slowly added a solution of catalyst (*R*)-**B2** (2.5 mg, 0.015 mmol, 10 mol%) in PhCl (0.1 mL). The reaction mixture was stirred at the same temperature for 12 h at room temperature or 72 h at -40 °C. After that, the reaction mixture was filtered through a short pad of silica gel and then concentrated. The crude mixture was examined by NMR and the ee value of the product was determined by HPLC. For the reaction at rt, crude ^{1}H NMR showed 100% conversion, 75% yield and 2% ee. For the reaction at -40 °C, crude NMR showed 75% yield and 2% ee

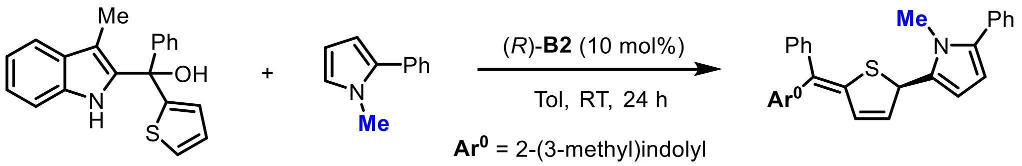

1a **2a'** **3a'**

46% yield, 89% ee
E/*Z* >20:1

(*R*,*E*)-3-Methyl-2-((5-(1-methyl-5-phenyl-1*H*-pyrrol-2-yl)thiophen-2(5*H*)-ylidene)(phenyl)methyl)-1*H*-indole (3a'). At room temperature, to an oven-dried 8-mL vial charged with a solution of the tertiary alcohol **1a** (63.8 mg, 0.2 mmol) and pyrrole **2a'** (37.7 mg, 0.24 mmol) in toluene (3.6 mL) was added a solution of catalyst (*R*)-**B2** (20 mg, 0.015 mmol, 10 mol%) in toluene (0.4 mL). The reaction mixture was stirred at the same temperature for 24 h. After that, triethylamine (2 drops) was added to quench the reaction. The mixture was concentrated under reduced pressure and purified by silica gel (deactivated by triethylamine) flash chromatography to afford the desired product **3a'** in 46% yield (41.7 mg, 89% ee, *E*/*Z* >20:1) as slightly green foam.

$[\alpha]_D^{26}$: -140.3 (c = 1.0, CH_2Cl_2). HPLC analysis of the product: Daicel CHIRALPAK AD-H column; 20% *i*-PrOH in hexanes; 1.0 mL/min; retention times: 8.0 min (major), 27.4 min (minor).

^{1}H NMR (400 MHz, acetone-d_6) δ 9.76 (s, 1H), 7.56-7.21 (m, 12H), 7.12-7.09 (m, 1H), 6.06-6.03 (m, 1H), 6.58-6.55 (m, 1H), 6.47-6.44 (m, 1H), 6.08-6.05 (m, 3H), 3.65 (s, 3H), 2.18 (s, 3H).

^{13}C NMR (100 MHz, acetone-d_6) δ 146.0, 141.0, 136.3, 136.0, 134.4, 133.4, 132.9, 132.0, 131.6, 128.7, 128.5, 128.4, 128.1, 127.0, 126.8, 121.6, 118.7, 118.4, 111.5, 110.80, 110.75, 109.9, 107.7, 107.3, 52.7, 31.6, 8.8.

IR (thin film) 3409, 3050, 2920, 1595, 1452, 1258, 1219, 1139, 1080, 1014, 802, 793 cm^{-1}.

HRMS (LD+) Calcd for C31H26N2S (M^+):458.1817, Found: 458.1812.

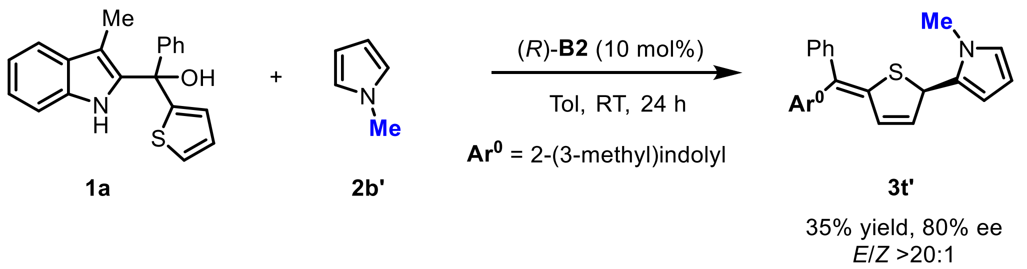

(*R*,*E*)-3-Methyl-2-((5-(1-methyl-1*H*-pyrrol-2-yl)thiophen-2(5*H*)-ylidene)(phenyl) methyl)-1*H*-indole (3t'). At room temperature, to an oven-dried 8-mL vial charged with a solution of the tertiary alcohol **1a** (63.8 mg, 0.2 mmol) and pyrrole **2b'** (162 mg, 2.0 mmol) in toluene (3.6 mL) was added a solution of catalyst (*R*)-**B2** (20 mg, 0.015 mmol, 10 mol%) in toluene (0.4 mL). The reaction mixture was stirred at the same temperature for 24 h. After that, triethylamine (2 drops) was added to quench the reaction. The mixture was concentrated under reduced pressure and purified by silica gel (deactivated by triethylamine) flash chromatography to afford the desired product **3t'** in 35% yield (26.9 mg, 80% ee, *E*/*Z* >20:1) as slightly pink foam.

$[\alpha]_D^{26}$: -51.3 (c = 1.0, CH_2Cl_2). HPLC analysis of the product: Daicel CHIRALPAK AD-H column; 20% *i*-PrOH in hexanes; 1.0 mL/min; retention times: 6.5 min (major), 20.3 min (minor).

^{1}H NMR (400 MHz, acetone-d_6) δ 9.85 (s, 1H), 7.54 (d, J = 7.8 Hz, 1H), 7.36-7.28 (m, 5H), 7.22-7.19 (m, 1H), 7.12-7.02 (m, 2H), 6.67-6.66 (m, 1H), 6.52 (dd, J_1 = 2.2 Hz, J_2 = 6.2 Hz, 1H), 6.37 (dd, J_1 = 3.0 Hz, J_2 = 6.2 Hz, 1H), 5.97-5.92 (m, 3H), 3.65 (s, 3H), 2.17 (s, 3H).

^{13}C NMR (100 MHz, acetone-d_6) δ 147.0, 141.9, 137.4, 137.3, 135.4, 133.6, 130.4, 129.8, 129.3, 129.0, 127.9, 124.4, 122.5, 121.6, 119.5, 119.3, 111.7, 110.7, 108.4, 107.5, 53.2, 34.2, 9.7.

IR (thin film) 3399, 3048, 2923, 1576, 1448, 1307, 1258, 1080, 1018, 797, 716 cm^{-1}.

HRMS (LD+) Calcd for $C_{25}H_{22}N_2S$ (M^+): 382.1504, Found: 382.1508.

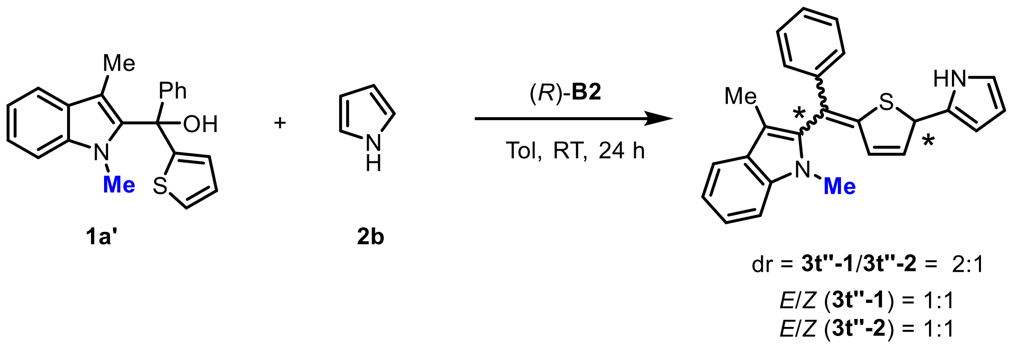

At room temperature, to an oven-dried 8-mL vial charged with a solution of the tertiary alcohol **1a'** (66.6 mg, 0.2 mmol) and pyrrole **2b** (134 mg, 2.0 mmol) in toluene (3.6 mL) was added a solution of catalyst (*R*)-**B2** (20 mg, 0.015 mmol, 10 mol%) in toluene (0.4 mL). The reaction mixture was stirred at the same temperature for 24 h. After that, triethylamine (2 drops) was added to quench the reaction. The mixture was concentrated under reduced pressure and purified by silica gel (deactivated by triethylamine) flash chromatography to afford the desired product **3t"-1** in 55% yield (42.3 mg, *E*/*Z* = 1:1) as slightly green foam and product **3t"-2** in 26% yield (19.5 mg, *E*/*Z* = 1:1) as slightly green foam. Various HPLC conditions by using different chiral column were tried, however neither **3t"-1** nor **3t"-2** could be well separated to give their isomers. However, the mixture of isomers for **3t"-1** and **3t"-2** was reported as below.

For product **3t"-1**, *E*/*Z* = 1:1.

^{1}H NMR (400 MHz, acetone-d_6) δ 10.08 (s, 1H), 10.00 (s, 1H), 7.58-7.56 (m, 2H), 7.36-7.29 (m, 10H), 7.21-7.15 (m, 4H), 7.10-7.06 (m, 2H), 6.78-6.72 (m, 2H), 6.35-6.32 (m, 2H), 6.26 (dd, J_1 = 2.1Hz, J_1 = 6.2 Hz, 2H), 6.08-6.00 (m, 4H), 5.97 (t, J = 2.5 Hz, 1H), 5.95 (t, J = 2.5 Hz, 1H), 3.40 (s, 3H), 3.39 (s, 3H), 2.21 (s, 3H), 2.19 (s, 3H).

^{13}C NMR (100 MHz, acetone-d_6) δ 149.1, 148.9, 141.50, 141.49, 138.3, 138.2, 138.1, 138.0, 137.6, 137.5, 133.1, 133.0, 130.1, 130.0, 129.9, 129.22, 129.15, 129.1, 128.9, 128.7, 128.6, 127.8, 127.7, 127.6, 122.38, 122.36, 119.77, 119.76, 119.6, 119.4, 119.3, 119.2, 119.1, 110.55, 110.50, 109.99, 109.97, 108.8, 107.4, 107.2, 54.8, 54.5, 30.63, 30.56, 9.7, 9.6.

IR (thin film) 3438, 3050, 2922, 1568, 1465, 1358, 1256, 1140, 1016, 722 cm^{-1}.

HRMS (CI+) Calcd for $C_{25}H_{22}N_2S$ (M^+): 382.1504, Found: 382.1495.

For product **3t"-2**, *E*/*Z* = 1:1.

^{1}H NMR (400 MHz, acetone-d_6) δ 9.99 (s, 2H), 7.54-7.51 (m, 2H), 7.37-7.33 (m, 4H),

7.28-7.22 (m, 8H), 7.15-7.10 (m, 2H), 7.04-7.00 (m, 2H), 6.96-6.92 (m, 2H), 6.75-6.73 (m, 1H), 6.72-6.70 (m, 1H), 6.49-6.45 (m, 2H), 6.03-5.96 (m, 4H), 5.84 (t, *J* =2.6 Hz), 5.79 (t, *J* =2.6 Hz), 3.45 (s, 3H), 3.41 (s, 3H), 2.26 (s, 3H), 2.24 (s, 3H).

^{13}C NMR (100 MHz, acetone-d_6) δ 151.2, 151.1, 141.2, 141.10, 141.05, 141.0, 139.3, 139.1, 138.3, 138.2, 130.5, 130.3, 129.93, 129.92, 129.5, 129.4, 129.28, 129.25, 129.2, 127.70, 127.67, 122.4, 119.7, 119.62, 119.56, 119.45, 119.41 (2C), 119.3, 119.2, 119.14, 119.07, 110.0, 109.9, 109.7, 109.4, 108.7, 108.6, 107.3, 107.1, 53.2, 53.1, 30.7 (2C), 9.9, 9.8.

IR (thin film) 3397, 3047, 2919, 1584, 1464, 1359, 1254, 1137, 1090, 1047, 787, 728 cm^{-1}.

HRMS (CI+) Calcd for $C_{25}H_{22}N_2S$ (M^+): 382.1504, Found: 382.1496.

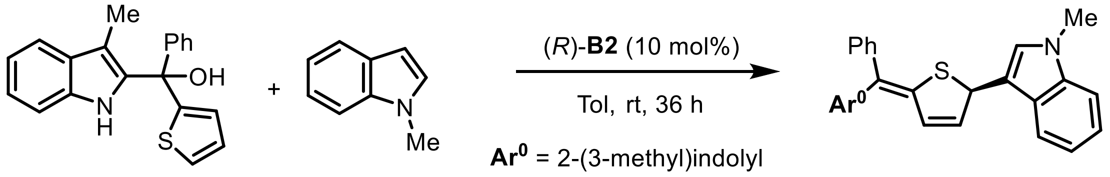

1a **9** **10**

44% yield, 56% conv.,
95% e.e., *E*/*Z* >20:1

(*R*,*E*)-1-Methyl-3-(5-((3-methyl-1*H*-indol-2-yl)(phenyl)methylene)-2,5-dihydrothiophen-2-yl)-1*H*-indole (10). At room temperature, to an oven-dried 8-mL vial charged with a solution of the tertiary alcohol **1a** (63.8 mg, 0.2 mmol) and pyrrole **9** (32.8 mg, 0.25 mmol) in toluene (3.6 mL) was added a solution of catalyst (*R*)-**B2** (20 mg, 0.015 mmol, 10 mol%) in toluene (0.4 mL). The reaction mixture was stirred at the same temperature for 36 h. After that, triethylamine (2 drops) was added to quench the reaction. The mixture was concentrated under reduced pressure and purified by silica gel (deactivated by triethylamine) flash chromatography to afford the desired product **10** in 44% yield (37.9 mg, 95% e.e., *E*/*Z* >20:1) as slightly purple foam. Additionally, 28.0 mg (44%) of **1a** was recovered.

$[\alpha]_D^{26}$: +106.9 (c = 1.0, CH_2Cl_2). HPLC analysis of the product: Daicel CHIRALPAK OD-H column; 15% *i*-PrOH in hexanes; 1.0 mL/min; retention times: 17.7 min (major), 29.1 min (minor).

^{1}H NMR (400 MHz, acetone-d_6) δ 9.88 (s, 1H), 7.65 (d, *J* = 8.0 Hz, 1H), 7.56 (d, *J* = 7.7 Hz, 1H), 7.40-7.36 (m, 3H), 7.34-7.25 (m, 3H), 7.21-7.03 (m, 6H), 6.53-6.51 (m,

1H), 6.45-6.43 (m, 1H), 6.12-6.11 (m, 1H), 3.79 (s, 3H), 2.24 (s, 3H).

^{13}C NMR (100 MHz, acetone-d_6) δ 148.0, 142.1, 139.4, 138.6, 137.3, 135.6, 132.7, 129.8, 129.2, 128.9, 128.0, 127.7, 127.5, 122.6, 122.4, 120.8, 120.2, 119.8, 119.5, 119.2, 113.8, 111.7, 110.5 (2C), 53.7, 32.8, 9.8.

IR (thin film) 3402, 3051, 2919, 2861, 1724, 1591, 1459, 1330, 1253, 1139, 1084, 735, 695 cm^{-1}.

HRMS (ES-) Calcd for C29H23N2S (M-H)$^-$: 431.1587, Found: 431.1585.

Non-linear effects.

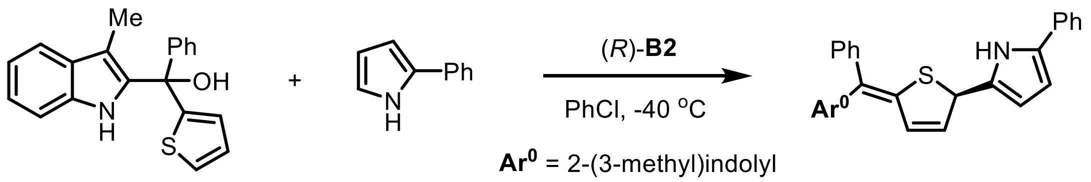

1a 2a 3a

At -40 ºC, to an oven-dried 4-mL vial charged with a solution of catalysts (*R*)-**B2** (2.5 mg, 10 mol%) with different enantiopurities (1st run: 0% ee, 2nd run: 20% ee; 3rd run: 40% ee; 4th run: 60% ee; 5th run: 80% ee; 6th run: 100% ee.) in PhCl (0.4 mL) was added a solution of **1a** (8.3 mg, 0.025 mmol,) and **2a** (4.3 mg, 0.03 mmol) in DCE (0.1 mL). The reaction mixture was stirred at 0 ºC for 12 h. After that, the reaction mixture was filtered through a short pad of silica gel and then concentrated. The ee value of the product **3a** was determined by HPLC.

ee-(**B2**)	0	20	40	60	80	100
ee-(**3a**)	0	17	37	56	77	98

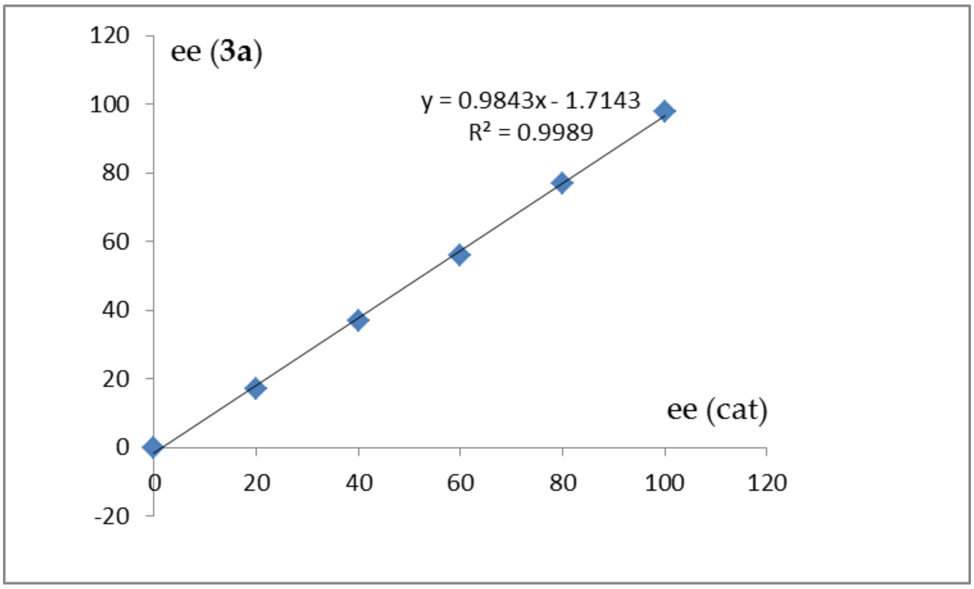

Linear relationship between product **3a** and catalyst's ee.

VII. DFTCalculations

Supplementary Table. 13. Absolute Calculation Energies, Enthalpies, and Free Energies

Geometry	$E_{(elec\text{-}B3LYP\text{-}D3BJ)}$[1]	$H_{(corr\text{-}B3LYP\text{-}D3BJ)}$[2]	$G_{(corr\text{-}B3LYP\text{-}D3BJ)}$[3]	$E_{(solv\text{-}M062X)}$[4]	IF[5]
**(R)-B2**	-3287.728073	1.518087	1.344659	-3286.830749	-
**CP1**	-722.762442	0.11661	0.072389	-722.7234764	-
**CP2**	-1300.614965	0.339407	0.268633	-1300.395637	-
**CP3**	-2023.417706	0.458625	0.365809	-2023.144971	-
**CP4-E**	-1946.962856	0.428696	0.339611	-1946.679999	-
**CP4-Z**	-1946.969004	0.428817	0.340623	-1946.684194	-
**CP5**	-441.266509	0.173545	0.13036	-441.1611084	-
**CP6-E**	-2388.28183	0.606584	0.498993	-2387.877831	-
**CP6-Z**	-2388.269229	0.606188	0.49552	-2387.870028	-
**CP7-E**	-1665.475587	0.487805	0.397048	-1665.138196	-
**CP7-Z**	-1665.478331	0.487886	0.398532	-1665.139526	-
**H2O**	-76.409528	0.024936	0.002835	-76.42737793	-
**TS1-E**	-2023.391742	0.453828	0.362046	-2023.11152	-269.93
**TS1-Z**	-2023.392778	0.453679	0.36266	-2023.112753	-382.17
**TS2-E-E**	-2388.257248	0.604358	0.49699	-2387.849406	-428.43
**TS2-E-Z**	-2388.261357	0.604354	0.498272	-2387.852443	-403.01
**TS2-R-E**	-4953.281913	2.008315	1.781288	-4951.995688	-432.32
**TS2-S-E**	-4953.271719	2.007285	1.778362	-4951.989286	-445.85
**TS2-Z-E**	-2388.274781	0.605123	0.498495	-2387.861903	-427.57
**TS2-Z-Z**	-2388.262244	0.604811	0.49653	-2387.851768	-431.2
**TS3-E**	-2388.258439	0.601328	0.491142	-2387.854771	-1328.87
**TS3-Z**	-2388.260518	0.601585	0.492136	-2387.857128	-956.97

[1]The electronic energy calculated by B3LYP-D3BJ in gas phase. [2]The thermal correction to enthalpy calculated by B3LYP-D3BJ in gas phase. [3] The thermal correction to Gibbs free energy calculated by B3LYP-D3BJ in gas phase. [4]The electronic energy calculated by M062X in chlorobenzene solvent. [5]The B3LYP-D3BJ calculated imaginary frequencies for the

transition states.

Supplementary Table. 14. B3LYP-D3BJ Geometries for All the Optimized Compounds and Transition States

CP1

P	-0.035562	0.461249	-0.108770
O	-0.009323	0.575770	1.516751
O	-0.619884	1.637011	-0.779919
O	1.462573	0.133399	-0.551583
O	-0.739491	-0.968005	-0.309915
C	-2.139685	-1.083243	0.000458
C	2.175026	-0.974661	0.026180
H	-0.068316	1.511549	1.770290
H	-2.715101	-0.345135	-0.565246
H	-2.437219	-2.091162	-0.291012
H	-2.300750	-0.942546	1.073645
H	1.701512	-1.917699	-0.258470
H	3.187543	-0.927123	-0.376366
H	2.202716	-0.884593	1.116211

CP2

C	-0.886709	0.038295	0.617371
C	4.079381	-1.166270	0.427788
C	2.793613	-0.609407	0.323507
C	5.144819	-0.516238	-0.180237
C	2.623478	0.601308	-0.405462
C	1.497613	-1.005041	0.812090
C	4.953484	0.685219	-0.894615
H	6.144024	-0.935884	-0.107176
C	3.694362	1.260059	-1.017510
C	0.606043	-0.043708	0.377472
H	5.807393	1.170814	-1.358174
H	3.547791	2.185223	-1.567910
H	0.835756	1.721973	-0.768620
H	4.234754	-2.091809	0.975456
N	1.284305	0.917077	-0.357269
C	1.189159	-2.254154	1.581771
H	1.297113	-3.145024	0.949598
H	1.872719	-2.376011	2.431239
H	0.166620	-2.246537	1.965810
C	-1.760747	1.629646	-1.264602
C	-2.201242	2.979280	-1.430194
H	-1.680657	0.909978	-2.071371
C	-2.232118	3.670058	-0.249556
H	-2.493786	3.405472	-2.383595

H	-2.548641	4.690698	-0.081620
C	-1.465096	1.319688	0.038349
S	-1.733751	2.683597	1.088672
C	-1.624893	-1.159157	0.010844
C	-2.813293	-1.604024	0.597729
C	-1.161513	-1.775634	-1.154360
C	-3.523837	-2.658956	0.028112
H	-3.166640	-1.126290	1.504410
C	-1.878181	-2.827112	-1.727977
H	-0.229905	-1.445340	-1.603475
C	-3.060369	-3.272618	-1.137603
H	-4.442069	-3.003086	0.496073
H	-1.504846	-3.302623	-2.630718
H	-3.615501	-4.095175	-1.579685
O	-1.131243	0.051374	2.040918
H	-0.268014	0.168144	2.467055

CP3

P	-3.093926	0.257509	-0.984802
O	-4.655659	0.375101	-1.307010
O	-2.975086	0.078356	0.611926
O	-2.324864	1.410029	-1.523781
O	-2.714628	-1.192527	-1.523466
C	0.866323	-0.948665	-0.286546
C	0.021056	-1.432925	0.894712
C	0.028254	-0.757131	2.116247
C	-0.765702	-2.582944	0.753864
C	-0.750693	-1.215900	3.179012
C	-1.551151	-3.033835	1.812007
C	-1.547882	-2.349985	3.028515
H	-0.767552	-3.118318	-0.189103
H	-0.735906	-0.680112	4.124194
H	-2.167765	-3.918865	1.684143
H	-2.160683	-2.701207	3.853919
H	-1.729076	-1.303511	-1.501452
O	-0.011848	-1.102056	-1.452662
H	0.463848	-0.782294	-2.237453
H	0.635580	0.134110	2.229353
C	2.073212	-1.836815	-0.492406
C	2.392161	-3.030307	0.099673
H	1.799408	-3.467503	0.893039
C	3.577846	-3.623428	-0.432224
C	4.139935	-2.878521	-1.431009
H	3.990701	-4.562682	-0.081498

H	5.036528	-3.085965	-1.999341
S	3.249137	-1.418645	-1.721115
C	0.247216	3.978008	-0.464781
C	0.812792	2.715609	-0.251251
C	0.958687	5.078011	-0.003707
C	2.062262	2.539352	0.403503
C	2.202363	4.928294	0.649040
H	0.550668	6.074724	-0.146710
C	2.759743	3.673600	0.854823
C	2.320443	1.122604	0.460316
C	1.245837	0.508783	-0.164035
H	2.729445	5.813663	0.993503
H	3.718747	3.569673	1.355549
C	3.514287	0.507195	1.128591
H	3.354692	-0.544787	1.373719
H	4.409903	0.563608	0.496587
H	3.741035	1.038945	2.060460
N	0.339426	1.470151	-0.582936
H	-0.562683	1.306405	-1.035252
H	-0.710179	4.078814	-0.967008
C	-2.797474	1.252356	1.426590
H	-3.649340	1.931383	1.318474
H	-2.732532	0.892943	2.453098
H	-1.875075	1.769565	1.153880
C	-5.554725	-0.661337	-0.874589
H	-5.470787	-0.815197	0.205430
H	-6.558987	-0.317480	-1.125310
H	-5.335353	-1.594620	-1.399930

CP4-E

P	-1.432228	-1.706537	-0.911940
O	-1.352721	-2.643504	-2.252923
O	-3.021991	-1.815276	-0.502494
O	-0.678946	-2.357419	0.231415
O	-1.097481	-0.300002	-1.340477
C	-0.052516	1.136782	0.562161
C	-1.012931	0.664028	1.554381
C	-0.575550	0.096603	2.767342
C	-2.396069	0.777123	1.308851
C	-1.498606	-0.334298	3.710932
C	-3.312513	0.330736	2.250032
C	-2.867336	-0.222183	3.453106
H	-2.724313	1.145733	0.345764
H	-1.153285	-0.763821	4.646284

H	-4.375123	0.386876	2.035870
H	-3.586750	-0.577627	4.185236
H	0.486764	0.012830	2.963856
C	-0.382946	2.301341	-0.209975
C	-1.101356	3.418888	0.203486
H	-1.480885	3.521088	1.212851
C	-1.226056	4.398629	-0.804538
C	-0.633609	4.005237	-1.987728
H	-1.736512	5.345621	-0.674354
H	-0.622756	4.547257	-2.924552
S	0.078006	2.445900	-1.889631
C	3.357132	-2.371538	0.517064
C	2.710751	-1.123611	0.475517
C	4.721914	-2.384481	0.300773
C	3.425680	0.082360	0.175700
C	5.456233	-1.193138	0.041766
H	5.255216	-3.330335	0.332147
C	4.826497	0.031662	-0.019676
C	2.479018	1.133724	0.131368
C	1.221617	0.537186	0.430124
H	6.528318	-1.260557	-0.116269
H	5.386641	0.937786	-0.231884
C	2.823508	2.582748	-0.022365
H	2.023962	3.234224	0.337024
H	3.011158	2.843598	-1.070456
H	3.735178	2.814176	0.540527
N	1.405703	-0.818032	0.687428
H	0.595604	-1.503541	0.599122
H	2.796458	-3.278906	0.715350
C	-3.958151	-1.231148	-1.402426
H	-3.721124	-0.177100	-1.586835
H	-4.945497	-1.314554	-0.939156
H	-3.963293	-1.759000	-2.364033
C	-1.532836	-4.048469	-2.078045
H	-0.804052	-4.447959	-1.365479
H	-1.389345	-4.512653	-3.057424
H	-2.543093	-4.272748	-1.713655

CP4-Z

P	-1.402559	-1.818821	0.892554
O	-1.601244	-2.966041	2.042932
O	-2.953755	-1.523196	0.421810
O	-0.672094	-2.507149	-0.251332
O	-0.878715	-0.536235	1.470871

C	0.064803	1.097541	-0.602447
C	3.216858	-2.652425	-0.653531
C	2.659823	-1.362838	-0.598620
C	4.575231	-2.763296	-0.427623
C	3.452965	-0.214312	-0.273054
C	5.389329	-1.629406	-0.144993
H	5.041430	-3.743663	-0.466725
C	4.846206	-0.365912	-0.065869
C	2.577901	0.892594	-0.200649
C	1.279171	0.388519	-0.517563
H	6.452830	-1.774151	0.018629
H	5.466892	0.494344	0.167039
C	3.011480	2.299125	0.071250
H	2.417147	3.029527	-0.482244
H	2.910366	2.551519	1.133060
H	4.062635	2.429097	-0.205055
N	1.375580	-0.973079	-0.802204
H	0.547192	-1.631798	-0.645714
H	2.592929	-3.515106	-0.861430
C	-3.639341	-2.574013	-0.252462
H	-3.782136	-3.437165	0.409033
H	-4.616063	-2.181442	-0.550644
H	-3.082940	-2.895408	-1.138797
C	-2.180002	-2.547169	3.278118
H	-3.209026	-2.196095	3.130251
H	-2.186070	-3.418191	3.938752
H	-1.592464	-1.740915	3.728783
C	-0.057264	2.354079	0.162978
C	0.123082	2.321471	1.555273
C	-0.341551	3.569406	-0.480234
C	0.037912	3.502392	2.286354
H	0.258377	1.356255	2.029746
C	-0.401352	4.749782	0.257945
H	-0.478006	3.587950	-1.556999
C	-0.210061	4.717033	1.640361
H	0.156834	3.475435	3.365532
H	-0.598637	5.691806	-0.244972
H	-0.265198	5.636703	2.215978
C	-1.036009	0.672100	-1.392752
C	-1.103253	-0.332423	-2.362628
H	-0.239696	-0.908838	-2.661401
C	-2.387786	-0.491193	-2.907045
C	-3.308281	0.373688	-2.346185
H	-2.636326	-1.216174	-3.672614

H	-4.363244	0.443545	-2.577176
S	-2.626263	1.393072	-1.154995

CP5

C	3.122894	-0.690235	-0.190222
C	3.211044	0.639799	0.167152
C	1.885057	1.132645	0.295555
C	1.009850	0.091099	0.018784
N	1.790585	-1.015268	-0.261970
H	1.426864	-1.891055	-0.604029
H	3.888160	-1.423856	-0.397056
H	4.125965	1.192965	0.328379
H	1.593322	2.128171	0.600067
C	-0.448704	0.038507	0.012787
C	-1.143331	-1.168373	0.208501
C	-1.199729	1.210219	-0.189671
C	-2.535859	-1.203771	0.190824
H	-0.591081	-2.082687	0.410496
C	-2.590535	1.174846	-0.192223
H	-0.679798	2.147134	-0.364795
C	-3.268087	-0.032833	-0.007661
H	-3.049961	-2.148321	0.346761
H	-3.149394	2.092950	-0.351957
H	-4.353779	-0.059851	-0.017025

CP6-E

P	-3.115576	-1.598666	0.276873
O	-4.253688	-1.611166	1.443134
O	-3.743443	-0.583322	-0.847150
O	-1.888846	-0.825505	0.785549
O	-2.938786	-2.991467	-0.236446
C	1.147514	0.760695	-1.333126
C	-2.904451	2.952407	0.488526
C	-1.647777	2.729131	-0.086366
C	-3.284632	4.270893	0.700163
C	-0.775380	3.797282	-0.445563
C	-2.439715	5.346850	0.344019
H	-4.254992	4.482155	1.141386
C	-1.195569	5.122803	-0.227498
C	0.414424	3.208687	-0.987580
C	0.230930	1.827466	-0.941783
H	-2.775607	6.364901	0.521013
H	-0.553014	5.956523	-0.498660
C	1.584620	3.970828	-1.530917

H	2.183744	3.365565	-2.215030
H	2.256702	4.315440	-0.733328
H	1.244428	4.859362	-2.076064
N	-1.027295	1.552327	-0.410565
H	-1.351274	0.634623	-0.080359
H	-3.552902	2.119134	0.738082
C	-4.889906	-1.038307	-1.570686
H	-4.682462	-1.993174	-2.064255
H	-5.118851	-0.273274	-2.315830
H	-5.747872	-1.165118	-0.900143
C	-4.545253	-0.385706	2.116243
H	-3.629928	0.078138	2.498020
H	-5.209963	-0.630448	2.947958
H	-5.051036	0.317586	1.443428
C	2.586867	0.966673	-1.042521
C	2.981440	1.481347	0.202615
C	3.579579	0.640252	-1.979371
C	4.328846	1.637388	0.511691
H	2.219767	1.741755	0.928106
C	4.929718	0.804950	-1.672228
H	3.288187	0.282674	-2.962326
C	5.309723	1.299379	-0.424158
H	4.613480	2.020512	1.487598
H	5.683930	0.555074	-2.413180
H	6.361695	1.428237	-0.185033
C	0.700318	-0.419518	-1.861443
C	-0.905107	-2.089904	-2.335135
C	0.260949	-2.968805	-1.987760
H	0.393611	-3.813922	-2.667507
C	0.070389	-3.587645	-0.512381
C	1.239785	-4.422651	-0.130678
C	1.961351	-3.768637	0.805115
C	1.244034	-2.542994	1.143516
N	0.126345	-2.513223	0.452284
H	-0.644254	-1.789639	0.531559
H	-0.919355	-4.056419	-0.488757
H	1.496619	-5.354672	-0.618039
H	2.897345	-4.077382	1.248049
C	1.655843	-1.490837	2.063332
C	0.762878	-0.467406	2.437451
C	2.975995	-1.468343	2.545103
C	1.194698	0.550089	3.280969
H	-0.261476	-0.471552	2.078939
C	3.400136	-0.441996	3.381434

H	3.679840	-2.235646	2.241609
C	2.509774	0.567389	3.753306
H	0.501793	1.336409	3.564008
H	4.425379	-0.425853	3.737673
H	2.841283	1.368858	4.407234
H	-1.882932	-2.525313	-2.493499
S	1.733343	-1.875371	-2.063674
C	-0.667273	-0.773995	-2.217368
H	-1.435475	-0.017261	-2.311204

CP6-Z

P	1.395925	-2.783182	-0.384552
O	2.077262	-3.855132	0.640277
O	-0.042542	-3.461123	-0.744499
O	1.004828	-1.526883	0.413200
O	2.282381	-2.660982	-1.584175
C	-1.671629	1.500422	-0.661344
C	-2.626539	-3.097039	0.966096
C	-2.752753	-1.769502	0.535239
C	-3.777555	-3.744552	1.394014
C	-4.007754	-1.094766	0.545685
C	-5.034153	-3.097883	1.396775
H	-3.712079	-4.776352	1.729542
C	-5.157902	-1.783010	0.975699
C	-3.778924	0.238826	0.080155
C	-2.414051	0.336401	-0.195434
H	-5.911714	-3.641828	1.735570
H	-6.124821	-1.286548	0.984772
C	-4.850336	1.270969	-0.104751
H	-4.583225	2.011930	-0.861136
H	-5.058434	1.820533	0.823051
H	-5.785672	0.792918	-0.418133
N	-1.808444	-0.892166	0.063576
H	-0.799762	-1.070403	0.096864
H	-1.664884	-3.593140	0.937551
C	-0.071088	-4.530937	-1.691607
H	0.361499	-4.210665	-2.643508
H	-1.121418	-4.798699	-1.824366
H	0.485767	-5.398034	-1.317129
C	1.419010	-4.113819	1.880414
H	1.213253	-3.181714	2.417032
H	2.090415	-4.741190	2.471611
H	0.474476	-4.646944	1.718427
C	-2.131431	2.817072	-0.141209

C	-2.349973	2.988942	1.234855
C	-2.391515	3.896533	-1.000186
C	-2.795831	4.208402	1.737827
H	-2.171921	2.151906	1.902566
C	-2.840386	5.116941	-0.497137
H	-2.266297	3.760952	-2.070257
C	-3.041850	5.278463	0.874112
H	-2.954270	4.324201	2.806529
H	-3.046799	5.937256	-1.179173
H	-3.397606	6.226985	1.266384
C	-0.599427	1.448198	-1.510100
C	1.501089	0.783659	-2.932406
C	1.344065	2.241709	-2.612889
H	2.108292	2.952557	-2.909453
C	2.722214	0.156821	-2.162460
C	4.004663	0.874996	-2.389722
C	4.470708	1.347845	-1.212908
C	3.545320	0.921547	-0.167246
N	2.555891	0.267088	-0.726732
H	1.833252	-0.339592	-0.228954
H	2.761738	-0.926234	-2.380586
H	4.454319	1.006196	-3.366187
H	5.380349	1.906545	-1.043870
C	3.673253	1.128846	1.270695
C	2.845426	0.433021	2.173655
C	4.630769	2.032952	1.762194
C	2.983819	0.650355	3.539917
H	2.115857	-0.285627	1.809288
C	4.755849	2.247687	3.130577
H	5.267513	2.580376	1.075177
C	3.933008	1.555722	4.021483
H	2.348064	0.107902	4.232823
H	5.493293	2.952681	3.501646
H	4.032615	1.720918	5.090350
S	-0.083554	-0.005187	-2.421920
C	0.294717	2.548768	-1.834101
H	0.117707	3.539097	-1.433357
H	1.666042	0.581719	-3.995774

CP7-E

C	1.741153	-0.928029	-0.242674
C	3.229739	3.818592	-0.354345
C	3.216510	2.422014	-0.315681
C	4.465601	4.450808	-0.269854

C	4.407356	1.654459	-0.186113
C	5.660140	3.710238	-0.156036
H	4.511069	5.535759	-0.295712
C	5.641679	2.322204	-0.115413
C	4.029681	0.267040	-0.158850
C	2.649855	0.221116	-0.272845
H	6.607695	4.238062	-0.097518
H	6.566258	1.758809	-0.023988
C	4.978897	-0.886804	-0.056683
H	4.509955	-1.824043	-0.363719
H	5.339128	-1.025564	0.971293
H	5.857891	-0.722581	-0.691435
N	2.168826	1.530602	-0.396412
H	1.194607	1.770465	-0.297785
H	2.310304	4.390058	-0.448692
C	2.039743	-1.973722	0.768005
C	2.427483	-1.604354	2.067112
C	1.976150	-3.340786	0.453504
C	2.720822	-2.570111	3.025695
H	2.499885	-0.550330	2.316067
C	2.274405	-4.307122	1.412597
H	1.715586	-3.644567	-0.554680
C	2.643996	-3.926810	2.702996
H	3.011046	-2.263676	4.026844
H	2.226862	-5.359316	1.146450
H	2.877409	-4.680472	3.449647
C	0.650676	-0.993596	-1.063936
C	-0.838601	-0.298350	-2.781760
C	-1.680230	-1.448662	-2.286858
H	-1.795149	-2.203813	-3.072443
C	-3.044232	-1.049013	-1.834840
C	-4.294450	-1.363327	-2.335857
C	-5.257963	-0.686161	-1.542535
C	-4.578885	0.030381	-0.565422
N	-3.231346	-0.185634	-0.779979
H	-2.501630	0.048909	-0.122661
H	-4.485726	-1.997266	-3.191307
H	-6.329215	-0.686881	-1.687567
C	-5.066526	0.887173	0.510240
C	-4.252993	1.882012	1.080506
C	-6.374339	0.734019	1.003205
C	-4.727124	2.687623	2.113046
H	-3.251657	2.047593	0.691628
C	-6.850208	1.549164	2.025285

H	-7.007749	-0.045060	0.590213
C	-6.028550	2.527585	2.590182
H	-4.081066	3.451053	2.537751
H	-7.863674	1.412071	2.392145
H	-6.399306	3.158207	3.392850
H	-1.211309	0.304517	-3.603888
S	-0.650486	-2.216291	-0.920398
C	0.327656	-0.082476	-2.156990
H	1.019570	0.703338	-2.436462

CP7-Z

C	1.853639	-0.941702	-0.259548
C	2.256950	4.004825	-0.052014
C	2.551812	2.638199	-0.071777
C	3.319194	4.886420	0.108899
C	3.879382	2.145636	0.069281
C	4.644680	4.421470	0.245028
H	3.125249	5.955075	0.127417
C	4.932576	3.063947	0.226218
C	3.819949	0.712463	0.005728
C	2.481432	0.370981	-0.164818
H	5.448961	5.141177	0.367765
H	5.955372	2.713950	0.335904
C	5.015653	-0.189008	0.070567
H	4.835886	-1.145744	-0.423562
H	5.308780	-0.411234	1.105039
H	5.873408	0.287619	-0.417697
N	1.730127	1.548119	-0.216706
H	0.725247	1.576992	-0.300643
H	1.237212	4.363327	-0.160729
C	2.501960	-2.042847	0.503070
C	2.835828	-1.863795	1.854900
C	2.820323	-3.265041	-0.109380
C	3.455143	-2.881415	2.575766
H	2.609404	-0.914990	2.331503
C	3.441300	-4.283991	0.612173
H	2.599023	-3.399918	-1.163850
C	3.759661	-4.096599	1.957626
H	3.701264	-2.726653	3.622640
H	3.687767	-5.219943	0.118513
H	4.248503	-4.887726	2.518923
C	0.708243	-1.187017	-0.966378
C	-1.522531	-1.175735	-2.448586
C	-1.189864	-2.431966	-1.683530

H	-1.876360	-3.271380	-1.732712
C	-2.836091	-0.587864	-2.067699
C	-3.874844	-0.088795	-2.833870
C	-4.888404	0.355663	-1.945781
C	-4.450204	0.126106	-0.646644
N	-3.196081	-0.440407	-0.748559
H	-2.656247	-0.803478	0.023123
H	-3.888643	-0.039263	-3.914373
H	-5.821862	0.828968	-2.216313
C	-5.080564	0.397124	0.641356
C	-4.318463	0.548457	1.813211
C	-6.478203	0.516805	0.736724
C	-4.933053	0.800531	3.037416
H	-3.233704	0.501164	1.762732
C	-7.088725	0.781846	1.958497
H	-7.083263	0.380792	-0.154451
C	-6.320983	0.920419	3.117272
H	-4.323599	0.916170	3.929415
H	-8.170545	0.869024	2.009202
H	-6.799627	1.120929	4.071270
S	-0.077000	-0.029164	-2.086836
C	-0.080989	-2.410976	-0.927538
H	0.226322	-3.238466	-0.299455
H	-1.523716	-1.348594	-3.530418

H2O

H	0.761649	-0.479066	0.000000
O	0.000000	0.119751	0.000000
H	-0.761649	-0.478941	0.000000

(R)-B2

P	0.298512	0.173692	-1.259675
O	0.945383	1.551179	-0.770047
O	-0.230616	0.148833	-2.631915
O	1.442496	-0.960782	-1.010586
O	-0.757337	-0.128268	-0.052081
C	-1.358519	-1.394931	-0.157268
C	-0.588173	-2.522331	0.141900
C	-2.699931	-1.471120	-0.530218
C	-1.100535	-3.798592	-0.176524
C	-3.212663	-2.750501	-0.742602
C	-2.423272	-3.902632	-0.638322
H	-4.256879	-2.846960	-1.027319
C	0.703888	-2.301217	0.850046

C	0.899753	-2.752264	2.178427
C	1.662669	-1.462780	0.277537
C	2.112563	-2.459982	2.826957
C	2.827743	-1.075829	0.942980
C	3.059530	-1.651811	2.190535
H	3.980899	-1.409283	2.711594
C	3.685583	0.016704	0.394335
C	3.546425	1.325561	0.932128
C	4.571144	-0.223408	-0.671976
C	4.317167	2.356059	0.384258
C	5.322066	0.843021	-1.178156
C	5.210699	2.136053	-0.669734
C	-3.553535	-0.249347	-0.590844
C	-3.989420	0.264872	-1.826269
C	-3.933992	0.372814	0.619732
C	-4.845694	1.371385	-1.824376
C	-4.778095	1.482660	0.568351
C	-5.253474	1.992101	-0.643730
C	-3.025790	-5.231835	-1.056258
H	-4.043851	-5.312668	-0.654964
H	-3.135205	-5.229180	-2.150757
C	-0.203781	-5.020693	-0.090949
H	0.815609	-4.739175	-0.377144
H	-0.136800	-5.372836	0.946255
C	-0.220888	-3.447273	2.932083
H	-0.267150	-4.507622	2.654550
H	-1.180671	-3.017407	2.626735
C	2.398700	-2.897701	4.253840
H	2.449741	-1.996423	4.882074
H	3.397133	-3.351066	4.300389
C	1.348900	-3.848212	4.834603
H	1.499545	-4.861973	4.438364
H	1.466249	-3.911210	5.922276
C	-2.186072	-6.444148	-0.645302
H	-2.546096	-7.339570	-1.164540
H	-2.301342	-6.631578	0.431537
C	-0.050652	-3.366692	4.451098
H	-0.824674	-3.970239	4.938555
H	-0.190357	-2.331168	4.790574
C	-0.710390	-6.181500	-0.951799
H	-0.103119	-7.073669	-0.760594
H	-0.595175	-5.936020	-2.016318
H	1.880972	1.459952	-0.491944
C	-6.168194	3.198619	-0.679154

C	-7.498144	2.946323	0.060487
C	-5.478283	4.462261	-0.124207
H	-6.413674	3.397919	-1.732807
C	-8.421052	4.170112	0.001871
H	-7.284462	2.703123	1.110785
H	-7.994126	2.066748	-0.367605
C	-6.402050	5.685278	-0.181828
H	-5.181083	4.279458	0.918055
H	-4.553281	4.648434	-0.682874
C	-7.724875	5.422731	0.549773
H	-9.345673	3.972186	0.558565
H	-8.716234	4.349636	-1.042378
H	-5.896157	6.560356	0.245058
H	-6.614013	5.927902	-1.233485
H	-8.386508	6.294104	0.467566
H	-7.521576	5.281566	1.621657
C	-3.426286	-0.119069	1.963477
C	-2.453189	0.886405	2.614605
C	-4.547690	-0.490779	2.951473
H	-2.857918	-1.038998	1.796842
C	-1.855626	0.310112	3.904180
H	-2.991512	1.818094	2.840034
H	-1.662539	1.138943	1.901694
C	-3.955623	-1.078505	4.240272
H	-5.143561	0.400031	3.194440
H	-5.232021	-1.207721	2.481536
C	-2.950333	-0.117992	4.890711
H	-1.188263	1.041811	4.376592
H	-1.231809	-0.558359	3.646206
H	-4.755931	-1.327083	4.948618
H	-3.446223	-2.023721	3.999061
H	-2.503626	-0.581236	5.779880
H	-3.485504	0.777662	5.238770
C	-3.534386	-0.350496	-3.135621
C	-4.633797	-1.214957	-3.786079
C	-3.014625	0.692103	-4.144101
H	-2.687409	-1.008529	-2.916543
C	-4.113927	-1.904047	-5.054341
H	-5.494576	-0.576654	-4.033471
H	-4.997757	-1.962244	-3.070086
C	-2.477031	0.001659	-5.402603
H	-3.825609	1.378176	-4.427227
H	-2.225850	1.284911	-3.674038
C	-3.544057	-0.888450	-6.053999

H	-4.913531	-2.493669	-5.521364
H	-3.322568	-2.613628	-4.771108
H	-2.120291	0.751407	-6.120152
H	-1.605169	-0.603863	-5.121081
H	-3.130231	-1.408407	-6.927578
H	-4.363652	-0.254818	-6.425075
C	2.586191	1.613509	2.080883
C	3.272541	1.484774	3.458459
C	1.886906	2.986433	2.006835
H	1.794519	0.855047	2.047231
C	2.242030	1.593825	4.589096
H	4.024575	2.281303	3.553047
H	3.812746	0.537113	3.530208
C	0.838919	3.114170	3.119468
H	2.627936	3.789119	2.123539
H	1.411992	3.125265	1.031322
C	1.457175	2.909913	4.508759
H	2.737676	1.504989	5.564071
H	1.543950	0.747158	4.510081
H	0.350366	4.094547	3.062834
H	0.054946	2.364887	2.951864
H	0.677111	2.935508	5.280117
H	2.139417	3.745197	4.724442
C	4.720215	-1.604369	-1.278388
C	4.297178	-1.638728	-2.761527
C	6.149590	-2.158308	-1.108151
H	4.051085	-2.284508	-0.739582
C	4.418184	-3.055049	-3.336979
H	4.936753	-0.956113	-3.338826
H	3.270154	-1.272069	-2.854804
C	6.270060	-3.573171	-1.688310
H	6.862148	-1.493987	-1.616870
H	6.421835	-2.153074	-0.045299
C	5.835598	-3.614875	-3.159348
H	4.136872	-3.054817	-4.397146
H	3.701161	-3.712090	-2.823208
H	7.299686	-3.937299	-1.582581
H	5.634242	-4.255179	-1.104888
H	5.892930	-4.640907	-3.543678
H	6.536861	-3.015124	-3.758228
C	6.016651	3.274719	-1.257918
C	5.111110	4.340199	-1.911132
C	6.951449	3.927789	-0.219130
H	6.652346	2.855279	-2.051459

C	5.932247	5.480999	-2.524804
H	4.432259	4.749202	-1.149731
H	4.477273	3.867045	-2.670605
C	7.771474	5.068233	-0.835484
H	6.347915	4.322934	0.610034
H	7.611602	3.165190	0.211694
C	6.865073	6.120133	-1.487997
H	5.262581	6.236099	-2.954301
H	6.533611	5.085748	-3.356484
H	8.405616	5.531408	-0.069441
H	8.449808	4.654397	-1.595892
H	7.469673	6.908317	-1.953279
H	6.259130	6.606332	-0.709317
H	-5.069142	1.963279	1.498457
H	-5.194996	1.771979	-2.772215
H	4.211950	3.358535	0.784841
H	6.005587	0.663156	-2.003975

TS1-E

P	-2.957300	0.567059	-0.790030
O	-4.085527	1.298583	-1.704451
O	-3.803441	0.043928	0.501498
O	-1.953679	1.618953	-0.401937
O	-2.483266	-0.704586	-1.491000
C	0.862556	-0.957817	0.048296
C	-0.278983	-1.201921	0.967407
C	-0.487495	-0.372921	2.080482
C	-1.120837	-2.309503	0.768543
C	-1.504206	-0.661036	2.986346
C	-2.144433	-2.582137	1.666258
C	-2.334095	-1.762663	2.780343
H	-0.984663	-2.929073	-0.110227
H	-1.649431	-0.020248	3.850782
H	-2.806619	-3.423141	1.487469
H	-3.136071	-1.977200	3.480834
H	-1.132531	-0.822719	-1.588952
O	-0.042833	-0.851277	-1.688951
H	0.159834	0.480213	2.243905
C	1.698474	-2.126851	-0.239167
C	1.905738	-3.221169	0.576553
H	1.403211	-3.340601	1.528102
C	2.863987	-4.130862	0.053982
C	3.355365	-3.730132	-1.163142
H	3.165275	-5.045013	0.552039

H	4.062913	-4.249910	-1.795494
S	2.664247	-2.238419	-1.689378
C	1.158868	3.955481	-0.023821
C	1.499083	2.596310	0.044374
C	2.186898	4.872393	0.122768
C	2.841208	2.159372	0.242773
C	3.527204	4.461228	0.329414
H	1.962007	5.934347	0.080702
C	3.862457	3.120064	0.391371
C	2.832086	0.728070	0.245056
C	1.494882	0.352398	0.056139
H	4.300283	5.216262	0.437825
H	4.893119	2.812021	0.543872
C	4.044239	-0.112729	0.510077
H	3.789300	-1.097746	0.905733
H	4.639707	-0.268068	-0.397627
H	4.688925	0.388247	1.240908
N	0.703762	1.491642	-0.051906
H	-0.333598	1.503227	-0.171751
H	0.129346	4.260923	-0.181032
C	-4.229830	1.027196	1.445089
H	-4.937790	1.728177	0.987350
H	-4.722970	0.489749	2.258663
H	-3.372574	1.584353	1.834110
C	-5.105748	0.480635	-2.283948
H	-5.709282	-0.000072	-1.505064
H	-5.738758	1.142394	-2.879488
H	-4.665139	-0.290504	-2.923166
H	0.186185	0.046456	-1.988090

TS1-Z

P	-3.079417	0.241237	0.586061
O	-4.151001	0.733464	1.707007
O	-4.023130	-0.334872	-0.608721
O	-2.301331	1.401260	0.026223
O	-2.339491	-0.925416	1.240042
C	1.025805	-0.775185	0.020812
H	-1.035761	-0.833172	1.458037
O	0.057089	-0.722228	1.657946
C	0.319560	4.088267	-0.399451
C	0.932910	2.829633	-0.311122
C	1.152185	5.189749	-0.505770
C	2.348040	2.669445	-0.314543
C	2.563015	5.055389	-0.522235

H	0.716077	6.181919	-0.580304
C	3.165439	3.813314	-0.428313
C	2.623815	1.270844	-0.179821
C	1.374608	0.635472	-0.118124
H	3.177120	5.947090	-0.607704
H	4.248018	3.722220	-0.434824
C	4.011351	0.699687	-0.169010
H	4.084554	-0.236820	-0.725576
H	4.365339	0.493671	0.847606
H	4.706287	1.413952	-0.621532
N	0.367172	1.592939	-0.206484
H	-0.658124	1.429361	-0.116929
H	-0.762052	4.176687	-0.385876
C	-4.737748	-1.548595	-0.362133
H	-4.042096	-2.370706	-0.171308
H	-5.324632	-1.755274	-1.260370
H	-5.410166	-1.438737	0.496206
C	-4.948404	1.879684	1.394349
H	-4.313329	2.744326	1.178535
H	-5.567373	2.080799	2.271461
H	-5.592275	1.681742	0.529432
C	2.149465	-1.693424	0.400932
C	2.720845	-1.664869	1.678698
C	2.710301	-2.522488	-0.581074
C	3.829349	-2.454563	1.969635
H	2.279090	-1.038395	2.442496
C	3.829802	-3.301665	-0.290563
H	2.285623	-2.534895	-1.578459
C	4.390674	-3.271257	0.985347
H	4.255763	-2.433319	2.968186
H	4.258950	-3.932882	-1.062946
H	5.258365	-3.883063	1.214417
C	0.002393	-1.332479	-0.863456
C	-0.735401	-0.703604	-1.848202
H	-0.594609	0.333150	-2.118983
C	-1.697604	-1.550656	-2.450971
C	-1.690422	-2.816256	-1.920277
H	-2.376423	-1.228371	-3.230496
H	-2.319990	-3.652523	-2.193784
S	-0.509427	-3.002302	-0.680631
H	0.156558	0.204635	1.939067

TS2-E-E

P	-1.968201	-0.854605	1.792433

O	-2.105255	-0.300341	3.332521
O	-3.265722	-1.835067	1.631453
O	-0.687399	-1.640567	1.762090
O	-2.234602	0.297548	0.843582
C	-0.044247	-0.695173	-1.651648
C	-0.966747	-1.845256	-1.696568
C	-0.427943	-3.105686	-2.027741
C	-2.343535	-1.751731	-1.427461
C	-1.242748	-4.227745	-2.113558
C	-3.151074	-2.883521	-1.497612
C	-2.610685	-4.120556	-1.848314
H	-2.758806	-0.822818	-1.062632
H	-0.810434	-5.188058	-2.379517
H	-4.203002	-2.796511	-1.244462
H	-3.246556	-5.000096	-1.900652
H	0.635289	-3.188880	-2.226352
C	-0.360748	0.592600	-2.018109
C	0.539299	1.717126	-2.012826
H	1.570619	1.598123	-1.713028
C	-0.016519	2.899940	-2.390022
C	-1.461807	2.842693	-2.479749
H	0.527429	3.832243	-2.456833
S	-1.954355	1.138902	-2.622704
C	3.392541	-2.180500	1.530667
C	2.770138	-1.702778	0.371063
C	4.780974	-2.162839	1.567196
C	3.518312	-1.205473	-0.737483
C	5.540855	-1.687762	0.473821
H	5.295490	-2.530051	2.451036
C	4.923473	-1.217221	-0.676405
C	2.571299	-0.752041	-1.717116
C	1.312479	-0.977770	-1.168370
H	6.625283	-1.694683	0.539370
H	5.515020	-0.849723	-1.511093
C	2.901677	-0.236985	-3.084394
H	2.000516	-0.101857	-3.689482
H	3.424307	0.729134	-3.048447
H	3.562541	-0.932987	-3.616172
N	1.441105	-1.588870	0.068281
H	0.647570	-1.682379	0.729947
H	2.803920	-2.548944	2.365452
C	-3.143899	-3.175996	2.109696
H	-2.937330	-3.194189	3.186237
H	-4.102473	-3.664506	1.916585

H	-2.344515	-3.702788	1.581389
C	-3.234721	0.514965	3.639856
H	-4.168247	-0.041496	3.495545
H	-3.142534	0.805795	4.689370
H	-3.255489	1.409524	3.007540
C	-2.103862	3.495127	-0.718834
C	-1.500683	4.808913	-0.641309
C	-0.343140	4.689134	0.084696
C	-0.277670	3.341636	0.577385
N	-1.395420	2.705368	0.195863
H	-1.643492	1.701746	0.425171
H	-3.167450	3.312302	-0.818418
H	-1.855732	5.683339	-1.170919
H	0.402503	5.449456	0.270869
C	0.814452	2.677012	1.278648
C	0.579603	1.533850	2.057384
C	2.136894	3.106767	1.068180
C	1.644865	0.804381	2.572105
H	-0.432975	1.215362	2.256684
C	3.199143	2.374269	1.588560
H	2.329822	3.979580	0.451164
C	2.956909	1.213774	2.329082
H	1.434431	-0.100597	3.131317
H	4.219309	2.692618	1.395785
H	3.787834	0.617494	2.691440
H	-2.013429	3.523458	-3.121215

TS2-E-Z

P	-2.174188	0.101330	1.741939
O	-2.072777	0.696524	3.274802
O	-3.752448	-0.327557	1.642253
O	-1.284077	-1.107755	1.713256
O	-2.022274	1.244879	0.756569
C	-0.467508	-0.693898	-1.813778
C	-1.666624	-1.558582	-1.718150
C	-1.533551	-2.917923	-2.057507
C	-2.916452	-1.088142	-1.285565
C	-2.629976	-3.771226	-2.006663
C	-4.008847	-1.950325	-1.222981
C	-3.874579	-3.288379	-1.592206
H	-3.006383	-0.075294	-0.914735
H	-2.513443	-4.815376	-2.282742
H	-4.957159	-1.573374	-0.852916
H	-4.728271	-3.958516	-1.539456

H	-0.562809	-3.291575	-2.367796
C	-0.525035	0.573829	-2.336108
C	-1.696900	1.292175	-2.773153
H	-2.645073	0.791794	-2.914567
C	-1.508670	2.625138	-2.934117
C	-0.159698	3.072905	-2.617382
H	-2.286816	3.326602	-3.213992
H	0.269630	3.889413	-3.186197
S	0.885814	1.671637	-2.404662
C	2.430269	-2.883713	1.485409
C	1.974615	-2.262637	0.317289
C	3.774142	-3.223959	1.548580
C	2.844375	-1.986434	-0.779171
C	4.654750	-2.957074	0.474341
H	4.159567	-3.710375	2.440693
C	4.200908	-2.350842	-0.687892
C	2.058275	-1.347102	-1.794091
C	0.767612	-1.248156	-1.275428
H	5.699768	-3.241050	0.562265
H	4.881889	-2.152655	-1.511647
C	2.532095	-1.005453	-3.173256
H	1.696208	-0.772943	-3.839173
H	3.206331	-0.138984	-3.177633
H	3.085715	-1.845814	-3.610368
N	0.719810	-1.834133	-0.018168
H	-0.055802	-1.656761	0.647095
H	1.751252	-3.080043	2.308904
C	-4.128445	-1.555972	2.268015
H	-3.907125	-1.534172	3.341672
H	-5.206620	-1.665165	2.123394
H	-3.603257	-2.398734	1.809944
C	-2.850713	1.852465	3.582235
H	-3.920605	1.643420	3.468445
H	-2.637861	2.113925	4.621986
H	-2.583826	2.689805	2.927596
C	-0.250330	3.983654	-0.806306
C	1.017510	4.671560	-0.860085
C	1.928151	3.916008	-0.167207
C	1.214163	2.834899	0.446384
N	-0.090872	2.965516	0.139068
H	-0.836075	2.254746	0.385464
H	-1.220986	4.457583	-0.883333
H	1.211392	5.562109	-1.443760
H	2.988451	4.092147	-0.055923

C	1.762261	1.803489	1.314015
C	0.988404	1.242109	2.338999
C	3.094266	1.381244	1.144580
C	1.534326	0.282731	3.184583
H	-0.032883	1.562274	2.488647
C	3.635248	0.423066	1.991514
H	3.684775	1.778946	0.324948
C	2.859195	-0.120067	3.019963
H	0.907160	-0.152012	3.955465
H	4.648603	0.069750	1.831716
H	3.284665	-0.876558	3.671014

TS2-R-E

C	0.791911	3.525099	1.721484
C	0.497638	-1.111622	3.406914
C	0.640513	0.280617	3.341449
C	0.562431	-1.693022	4.664099
C	0.851363	1.085079	4.501273
C	0.753535	-0.917122	5.832966
H	0.467520	-2.769541	4.751842
C	0.896358	0.459967	5.764185
C	0.964921	2.442811	4.062139
C	0.808749	2.423232	2.670407
H	0.791977	-1.415088	6.797548
H	1.047654	1.047137	6.665863
C	1.140688	3.628543	4.960796
H	0.810342	4.554767	4.485494
H	2.190565	3.770490	5.249505
H	0.564385	3.497103	5.884011
N	0.605899	1.109786	2.257990
H	0.623733	0.778386	1.280588
H	0.332513	-1.699882	2.512218
C	1.726843	4.646308	1.951893
C	3.043093	4.383552	2.365985
C	1.333991	5.982200	1.763256
C	3.949700	5.422069	2.548403
H	3.348265	3.355644	2.520437
C	2.240645	7.021846	1.957575
H	0.306337	6.204304	1.494489
C	3.553240	6.745405	2.343492
H	4.969946	5.197099	2.845295
H	1.918078	8.049447	1.816741
H	4.260377	7.556571	2.491659
C	-0.049228	3.492531	0.627590

C	-1.623221	2.599315	-0.880213
C	-0.973527	3.567759	-1.747125
H	-1.555268	4.078648	-2.506451
C	0.192816	2.462556	-2.945104
C	0.687495	3.461605	-3.857720
C	1.930254	3.848434	-3.423374
C	2.271529	3.029780	-2.296372
N	1.273852	2.145923	-2.119502
H	1.191007	1.416689	-1.382114
H	-0.491692	1.651551	-3.166151
H	0.116427	3.887494	-4.671979
H	2.547861	4.633816	-3.833186
C	3.454708	3.107969	-1.454187
C	3.686938	2.181374	-0.422392
C	4.383190	4.144874	-1.665157
C	4.824204	2.304926	0.370069
H	3.004042	1.355190	-0.252143
C	5.514872	4.258513	-0.868587
H	4.209826	4.873017	-2.449506
C	5.738339	3.336077	0.155222
H	4.992528	1.591406	1.165162
H	6.218121	5.067276	-1.042674
H	6.620789	3.419755	0.782972
H	-2.389942	1.934625	-1.244886
S	0.074393	4.608356	-0.772124
C	-1.096014	2.537800	0.364601
H	-1.421060	1.831818	1.112700
P	-0.361303	-0.666827	-0.914801
O	-0.962902	-0.244169	-2.213334
O	0.758780	0.154654	-0.291696
O	-1.490475	-0.834391	0.303114
O	0.116574	-2.229035	-1.088578
C	0.363567	-3.027948	0.010979
C	-0.737861	-3.513172	0.718289
C	1.686922	-3.366347	0.338544
C	-0.531918	-4.283454	1.885976
C	1.844658	-4.253820	1.405334
C	0.775459	-4.686127	2.199441
H	2.844516	-4.588620	1.661078
C	-2.102424	-3.161095	0.240720
C	-3.018805	-4.165151	-0.149307
C	-2.467145	-1.813278	0.126065
C	-4.340574	-3.802262	-0.447711
C	-3.784445	-1.433638	-0.161814

C	-4.707316	-2.454408	-0.389730
H	-5.741602	-2.180014	-0.577506
C	-4.214124	-0.005592	-0.219508
C	-4.458626	0.604535	-1.471336
C	-4.415199	0.712549	0.975825
C	-4.914005	1.925370	-1.496067
C	-4.822766	2.048983	0.895134
C	-5.080135	2.672071	-0.325392
C	2.824363	-2.713326	-0.372368
C	3.754573	-1.927321	0.354420
C	2.918443	-2.778317	-1.783841
C	4.691089	-1.172942	-0.357883
C	3.869271	-1.995929	-2.442087
C	4.737534	-1.151403	-1.750922
C	1.075271	-5.546995	3.412691
H	1.820384	-6.305795	3.142364
H	1.550575	-4.923799	4.185685
C	-1.698606	-4.594947	2.809400
H	-2.292971	-3.682802	2.941908
H	-2.375233	-5.315456	2.337499
C	-2.541756	-5.594992	-0.349290
H	-2.445422	-6.115594	0.611638
H	-1.532703	-5.574754	-0.775732
C	-5.380732	-4.816454	-0.888666
H	-5.618991	-4.630417	-1.946426
H	-6.314206	-4.643942	-0.337516
C	-4.922341	-6.268350	-0.732641
H	-4.960901	-6.562153	0.325839
H	-5.603276	-6.935488	-1.273648
C	-0.174622	-6.204043	4.001956
H	0.066728	-6.681158	4.958882
H	-0.529075	-6.996916	3.328298
C	-3.485721	-6.409792	-1.238181
H	-3.169932	-7.459442	-1.244571
H	-3.426438	-6.050370	-2.274864
C	-1.274962	-5.155352	4.171832
H	-2.149635	-5.579990	4.677920
H	-0.906669	-4.344868	4.813289
C	5.700366	-0.228923	-2.466884
C	6.923758	-0.970279	-3.042174
C	5.017327	0.592643	-3.577102
H	6.075843	0.487852	-1.722081
C	7.910793	0.009028	-3.692340
H	6.579386	-1.702182	-3.786862

H	7.417328	-1.542184	-2.246902
C	5.994937	1.591579	-4.205051
H	4.645410	-0.085564	-4.357201
H	4.142250	1.107651	-3.170418
C	7.231645	0.873988	-4.763791
H	8.757805	-0.537509	-4.125927
H	8.326542	0.664582	-2.912881
H	5.495541	2.163173	-4.998041
H	6.304117	2.318717	-3.440207
H	7.945150	1.600366	-5.173223
H	6.922572	0.231003	-5.600970
C	2.060599	-3.718476	-2.612505
C	1.334303	-3.028990	-3.784609
C	2.889551	-4.912082	-3.134483
H	1.288939	-4.145730	-1.967200
C	0.426191	-4.024740	-4.515268
H	2.073174	-2.635039	-4.497065
H	0.752414	-2.180790	-3.417096
C	1.998643	-5.912175	-3.883982
H	3.679535	-4.542624	-3.803937
H	3.395989	-5.404950	-2.295104
C	1.219772	-5.236895	-5.021443
H	-0.085257	-3.528331	-5.349804
H	-0.359839	-4.361674	-3.823430
H	2.603232	-6.740854	-4.274677
H	1.284643	-6.353047	-3.172527
H	0.551647	-5.960946	-5.505427
H	1.930318	-4.901627	-5.791707
C	3.804145	-1.897107	1.875912
C	4.894356	-2.859000	2.401435
C	4.038392	-0.501443	2.483664
H	2.843150	-2.242806	2.265022
C	4.898723	-2.921214	3.933159
H	5.872884	-2.509070	2.041364
H	4.757105	-3.859173	1.974899
C	4.049160	-0.543029	4.016648
H	5.012067	-0.119799	2.150098
H	3.270490	0.192925	2.124728
C	5.099761	-1.527891	4.542721
H	5.678485	-3.609853	4.282870
H	3.936926	-3.330092	4.276942
H	4.232931	0.463331	4.414203
H	3.062749	-0.843782	4.379097
H	5.056194	-1.579667	5.637920

H	6.106577	-1.166967	4.283166
C	-4.192109	-0.143805	-2.765672
C	-5.460968	-0.787173	-3.362072
C	-3.504752	0.711101	-3.846396
H	-3.491182	-0.950252	-2.531420
C	-5.105174	-1.662579	-4.571157
H	-6.160286	0.007107	-3.662501
H	-5.975921	-1.386318	-2.604420
C	-3.112396	-0.161355	-5.043899
H	-4.179099	1.510420	-4.186632
H	-2.616181	1.182932	-3.421591
C	-4.336477	-0.871407	-5.638571
H	-6.013446	-2.101279	-5.004149
H	-4.483383	-2.500506	-4.223818
H	-2.620175	0.447895	-5.813455
H	-2.374128	-0.899410	-4.703847
H	-4.033288	-1.535610	-6.457883
H	-5.008910	-0.118768	-6.077155
C	-4.219897	0.061983	2.335106
C	-2.959766	0.572425	3.062975
C	-5.463453	0.206740	3.235782
H	-4.074683	-1.011790	2.174171
C	-2.769793	-0.120342	4.417346
H	-3.038228	1.659742	3.210476
H	-2.084055	0.392483	2.434915
C	-5.264348	-0.505308	4.579733
H	-5.665222	1.270562	3.421966
H	-6.342639	-0.190918	2.713638
C	-4.014030	0.011878	5.302247
H	-1.888302	0.284396	4.924781
H	-2.559185	-1.185293	4.243816
H	-6.154190	-0.377280	5.209293
H	-5.157100	-1.585693	4.402402
H	-3.868510	-0.526341	6.247499
H	-4.162393	1.070652	5.563016
C	-5.469527	4.134862	-0.377060
C	-4.335592	4.995405	-0.975569
C	-6.787452	4.381807	-1.136621
H	-5.620755	4.474543	0.658323
C	-4.703646	6.483289	-1.008279
H	-4.132816	4.646573	-1.999007
H	-3.414622	4.834561	-0.403703
C	-7.154769	5.871095	-1.162245
H	-6.684812	4.018634	-2.168607

H	-7.590923	3.792821	-0.678177
C	-6.021728	6.717376	-1.757685
H	-3.892590	7.062509	-1.467832
H	-4.804848	6.851304	0.023203
H	-8.081762	6.021099	-1.729579
H	-7.356883	6.210897	-0.135869
H	-6.288914	7.781311	-1.738125
H	-5.887756	6.445698	-2.815313
H	3.918696	-2.038076	-3.525678
H	5.398200	-0.555537	0.185801
H	-5.101335	2.397111	-2.455786
H	-4.948465	2.621505	1.810769

TS2-S-E

C	-3.250655	2.934387	0.416979
C	-0.962760	0.827095	4.259953
C	-1.821969	1.610504	3.477168
C	-1.128741	0.893325	5.635171
C	-2.825998	2.446032	4.046580
C	-2.117477	1.717338	6.226933
H	-0.473680	0.310389	6.276170
C	-2.961652	2.492704	5.450249
C	-3.499067	3.095407	2.968501
C	-2.891762	2.637824	1.781147
H	-2.207719	1.740586	7.309147
H	-3.715852	3.124229	5.911782
C	-4.589037	4.108038	3.151963
H	-4.621528	4.835846	2.338964
H	-5.581836	3.643477	3.204628
H	-4.432407	4.654426	4.088688
N	-1.882933	1.746109	2.119567
H	-1.189455	1.287994	1.516996
H	-0.189647	0.227758	3.793483
C	-4.662536	3.337014	0.174183
C	-5.702580	2.501856	0.604010
C	-4.975138	4.528461	-0.496509
C	-7.028468	2.843619	0.355392
H	-5.458053	1.584938	1.126436
C	-6.304979	4.877203	-0.730720
H	-4.172397	5.186736	-0.814457
C	-7.334083	4.034159	-0.309171
H	-7.824212	2.179504	0.681192
H	-6.536078	5.808675	-1.239376
H	-8.369658	4.305002	-0.494582

C	-2.422148	2.778914	-0.677351
C	-1.014377	2.496461	-0.773710
H	-0.353728	2.508624	0.081777
C	-0.574897	2.224483	-2.031049
C	-1.626766	2.181965	-3.041352
H	0.456210	1.996359	-2.267440
H	-1.415201	2.533021	-4.047290
S	-3.115469	2.810746	-2.329637
C	-1.918795	0.282227	-3.493696
C	-3.147491	0.235288	-4.252598
C	-4.159956	-0.082051	-3.382173
C	-3.564371	-0.351809	-2.098782
N	-2.239906	-0.249916	-2.243348
H	-1.493237	-0.430599	-1.532360
H	-0.940699	0.043953	-3.895308
H	-3.246668	0.527283	-5.289491
H	-5.219794	-0.098726	-3.591159
C	-4.193920	-0.633853	-0.816471
C	-3.415981	-0.755032	0.350164
C	-5.591706	-0.753982	-0.720686
C	-4.026131	-0.978003	1.580423
H	-2.335535	-0.694083	0.299881
C	-6.195078	-0.996551	0.507857
H	-6.205399	-0.658145	-1.609805
C	-5.414286	-1.103535	1.663907
H	-3.410768	-1.051371	2.470597
H	-7.275012	-1.094468	0.566772
H	-5.887177	-1.281686	2.625230
P	0.778862	-0.434395	0.308802
O	-0.140686	-0.828086	-0.813527
O	0.394153	0.633164	1.291103
O	1.201469	-1.738306	1.221047
O	2.225061	-0.115929	-0.423507
C	3.319958	0.043343	0.413601
C	3.891808	-1.092583	1.001248
C	3.812601	1.336355	0.621366
C	4.899676	-0.916878	1.976238
C	4.888479	1.461717	1.499817
C	5.402457	0.373696	2.214196
H	5.308844	2.448832	1.671602
C	3.394475	-2.433551	0.570550
C	4.234992	-3.374855	-0.069484
C	2.022842	-2.702793	0.660395
C	3.694883	-4.609779	-0.470279

C	1.447648	-3.884348	0.179269
C	2.326154	-4.849285	-0.315094
H	1.912157	-5.786412	-0.679773
C	-0.030181	-4.012447	0.010018
C	-0.556332	-4.049095	-1.300724
C	-0.909762	-4.023456	1.114087
C	-1.942279	-4.133350	-1.476962
C	-2.284154	-4.094371	0.885209
C	-2.826797	-4.157181	-0.401638
C	3.190880	2.508866	-0.062703
C	2.551646	3.518875	0.690375
C	3.235549	2.609277	-1.473422
C	1.988952	4.609139	0.015761
C	2.652578	3.714868	-2.097675
C	2.024015	4.729950	-1.371893
C	6.454622	0.648691	3.275193
H	7.225579	1.311621	2.861715
H	5.980749	1.215258	4.090732
C	5.364109	-2.094410	2.814694
H	4.515258	-2.763071	2.994193
H	6.102090	-2.690144	2.263099
C	5.665528	-3.015324	-0.429913
H	6.331307	-3.168252	0.429023
H	5.719743	-1.946550	-0.663182
C	4.519861	-5.664755	-1.189887
H	4.121759	-5.771864	-2.209993
H	4.367004	-6.639241	-0.708126
C	6.013506	-5.338499	-1.273859
H	6.501061	-5.561047	-0.314428
H	6.491681	-5.974437	-2.027876
C	7.090134	-0.616498	3.857040
H	7.648212	-0.368717	4.767420
H	7.813619	-1.034668	3.143063
C	6.204107	-3.854689	-1.591107
H	7.261828	-3.619566	-1.757555
H	5.667839	-3.602138	-2.516845
C	6.006180	-1.659498	4.134824
H	6.422600	-2.534027	4.648221
H	5.244211	-1.228752	4.799017
C	1.388850	5.921310	-2.060521
C	2.383959	6.684575	-2.957693
C	0.134965	5.523653	-2.867382
H	1.058705	6.617358	-1.275051
C	1.728512	7.897549	-3.630004

H	2.765320	6.006033	-3.733371
H	3.251197	6.993723	-2.361926
C	-0.515563	6.733828	-3.547702
H	0.423725	4.785647	-3.630230
H	-0.580718	5.022193	-2.208396
C	0.485498	7.488808	-4.431171
H	2.452212	8.405219	-4.279895
H	1.436396	8.624590	-2.858070
H	-1.383164	6.411531	-4.137704
H	-0.899668	7.416207	-2.775149
H	0.011742	8.371430	-4.878850
H	0.792347	6.839247	-5.264453
C	3.937817	1.573869	-2.334305
C	2.997246	0.904507	-3.356750
C	5.174947	2.162705	-3.043018
H	4.308539	0.777502	-1.683783
C	3.736955	-0.168469	-4.165587
H	2.600322	1.665894	-4.044786
H	2.149178	0.459378	-2.827036
C	5.915915	1.085680	-3.845328
H	4.862994	2.973511	-3.716514
H	5.841417	2.614516	-2.298113
C	4.984776	0.400123	-4.853459
H	3.063791	-0.611996	-4.908729
H	4.031537	-0.982115	-3.486063
H	6.781030	1.522769	-4.359936
H	6.312068	0.330843	-3.149974
H	5.520324	-0.393853	-5.389264
H	4.673248	1.135228	-5.610647
C	0.316110	-3.970995	-2.543703
C	0.306022	-5.293251	-3.337275
C	-0.056087	-2.785903	-3.456010
H	1.348341	-3.792942	-2.233123
C	1.226340	-5.219746	-4.561851
H	-0.720618	-5.514364	-3.662382
H	0.606754	-6.120665	-2.682506
C	0.865894	-2.721517	-4.679126
H	-1.099413	-2.875095	-3.790277
H	0.021167	-1.871761	-2.863646
C	0.858247	-4.037051	-5.467211
H	1.185909	-6.160674	-5.125284
H	2.266093	-5.101177	-4.221051
H	0.576181	-1.885156	-5.330805
H	1.889920	-2.511996	-4.341205

H	1.547876	-3.978773	-6.318985
H	-0.146094	-4.203381	-5.884606
C	-0.402742	-3.985637	2.542526
C	-0.996959	-2.814821	3.348820
C	-0.652307	-5.320995	3.273778
H	0.680996	-3.837816	2.518903
C	-0.430888	-2.775840	4.772260
H	-2.089547	-2.923793	3.403987
H	-0.791350	-1.875983	2.826793
C	-0.088802	-5.286965	4.700248
H	-1.733041	-5.519988	3.308941
H	-0.202272	-6.141878	2.701705
C	-0.656883	-4.107298	5.500446
H	-0.887891	-1.952261	5.332453
H	0.647370	-2.564737	4.720957
H	-0.298934	-6.233896	5.213929
H	1.005920	-5.193202	4.649529
H	-0.210487	-4.076623	6.502616
H	-1.737557	-4.258052	5.642777
C	-4.332534	-4.258253	-0.567842
C	-4.836019	-4.021938	-2.000007
C	-4.860309	-5.616267	-0.053418
H	-4.779614	-3.480927	0.068125
C	-6.366633	-4.080281	-2.073815
H	-4.421504	-4.794100	-2.663621
H	-4.471670	-3.058925	-2.375244
C	-6.390555	-5.691218	-0.124596
H	-4.417998	-6.418042	-0.662110
H	-4.518597	-5.782591	0.974609
C	-6.900800	-5.417428	-1.545010
H	-6.700648	-3.918072	-3.106770
H	-6.786390	-3.261654	-1.472739
H	-6.738721	-6.671737	0.223749
H	-6.816544	-4.943721	0.561054
H	-7.997936	-5.427004	-1.566638
H	-6.565366	-6.226597	-2.210037
H	2.697386	3.786211	-3.181169
H	1.502396	5.390785	0.592909
H	-2.330845	-4.161280	-2.488684
H	-2.960719	-4.095436	1.736028
C	2.474492	3.479300	2.207065
C	3.444154	4.502056	2.838505
C	1.049763	3.696883	2.751619
H	2.780295	2.482388	2.538138

C	3.417907	4.433561	4.369954
H	3.158353	5.512104	2.510864
H	4.462140	4.336125	2.465530
C	1.023596	3.627373	4.283351
H	0.666308	4.676314	2.433395
H	0.391523	2.935838	2.330384
C	1.996316	4.632161	4.911147
H	4.100144	5.181831	4.793777
H	3.791581	3.449125	4.689421
H	0.003774	3.793675	4.649194
H	1.298012	2.612129	4.598307
H	1.987953	4.541145	6.004857
H	1.664096	5.655024	4.677883

TS2-Z-E

P	-3.281129	-1.361252	0.154126
O	-4.497939	-1.315061	1.239467
O	-3.741593	-0.249900	-0.966614
O	-2.033414	-0.716223	0.770091
O	-3.201619	-2.742491	-0.410975
C	1.228634	0.639360	-1.239187
C	-2.715395	3.061244	0.483278
C	-1.458494	2.763024	-0.059512
C	-3.018999	4.399056	0.684396
C	-0.513272	3.778726	-0.390898
C	-2.101328	5.425618	0.352669
H	-3.986506	4.669319	1.098525
C	-0.859340	5.129644	-0.183624
C	0.652636	3.124909	-0.895487
C	0.383397	1.748620	-0.858457
H	-2.382688	6.461025	0.522398
H	-0.160624	5.922964	-0.435353
C	1.869032	3.827720	-1.417584
H	2.419905	3.217831	-2.136736
H	2.570110	4.087406	-0.613658
H	1.582126	4.761357	-1.914815
N	-0.905966	1.552813	-0.368408
H	-1.305122	0.656687	-0.048795
H	-3.417626	2.267913	0.713384
C	-4.869239	-0.577069	-1.782235
H	-4.709078	-1.527957	-2.300467
H	-4.982227	0.231353	-2.508280
H	-5.778965	-0.653579	-1.175229
C	-4.712216	-0.106014	1.966681

H	-3.781085	0.246881	2.421784
H	-5.443297	-0.329030	2.747452
H	-5.113001	0.677261	1.310943
C	2.691164	0.790233	-1.054806
C	3.197302	1.297763	0.150797
C	3.591808	0.428071	-2.069791
C	4.569127	1.417987	0.346406
H	2.504880	1.573979	0.936262
C	4.965691	0.561218	-1.876671
H	3.208232	0.067327	-3.018952
C	5.458657	1.052272	-0.666724
H	4.943616	1.792638	1.294689
H	5.650242	0.288112	-2.674566
H	6.529721	1.153758	-0.516059
C	0.706098	-0.550538	-1.703407
C	-0.898587	-2.165728	-2.301186
C	0.217286	-3.035122	-1.955845
H	0.355594	-3.976527	-2.477301
C	-0.268462	-3.761572	-0.162032
C	0.860110	-4.602058	0.156144
C	1.701759	-3.884593	0.970138
C	1.060935	-2.633639	1.262810
N	-0.139752	-2.644611	0.664655
H	-0.855706	-1.882254	0.693200
H	-1.282267	-4.077194	-0.383007
H	1.042607	-5.578696	-0.272254
H	2.675175	-4.185033	1.329929
C	1.545805	-1.511040	2.056167
C	0.708792	-0.417519	2.352667
C	2.869544	-1.504313	2.530711
C	1.190992	0.644023	3.113193
H	-0.319195	-0.400939	2.007909
C	3.343520	-0.439860	3.288459
H	3.533892	-2.327686	2.290607
C	2.504867	0.637436	3.586375
H	0.534095	1.480377	3.332877
H	4.369571	-0.447885	3.643980
H	2.875446	1.468261	4.180060
H	-1.875272	-2.569338	-2.531016
S	1.674446	-2.055222	-1.767132
C	-0.646066	-0.848014	-2.098436
H	-1.394743	-0.073093	-2.194820

TS2-Z-Z

P	-3.267232	-1.260342	0.153086
O	-4.360304	-1.601879	1.315361
O	-3.852441	0.112690	-0.521490
O	-1.968088	-0.791727	0.824706
O	-3.246034	-2.392942	-0.823054
C	1.346903	0.864542	-1.050839
C	-2.856646	2.904559	0.589040
C	-1.556896	2.713080	0.098666
C	-3.246800	4.206900	0.860307
C	-0.658329	3.801754	-0.099457
C	-2.376849	5.305365	0.653278
H	-4.250065	4.392677	1.234398
C	-1.092590	5.115423	0.174896
C	0.575757	3.256715	-0.561612
C	0.394646	1.863090	-0.633478
H	-2.728215	6.309082	0.875227
H	-0.426061	5.960005	0.022488
C	1.764368	4.083881	-0.950670
H	2.349254	3.622275	-1.748671
H	2.448080	4.245229	-0.107518
H	1.435140	5.068529	-1.299943
N	-0.912433	1.558740	-0.258120
H	-1.267057	0.628601	0.010411
H	-3.522398	2.060196	0.716417
C	-4.953213	-0.007392	-1.424903
H	-4.703843	-0.687463	-2.244268
H	-5.150755	0.994492	-1.813032
H	-5.843646	-0.382163	-0.906305
C	-4.491994	-0.687916	2.404301
H	-3.524250	-0.511376	2.884295
H	-5.183630	-1.142218	3.117861
H	-4.902532	0.270576	2.063121
C	2.781708	1.182949	-0.813130
C	3.207600	1.536184	0.475075
C	3.724854	1.145029	-1.851465
C	4.546565	1.824118	0.724284
H	2.480534	1.567109	1.277647
C	5.063063	1.447295	-1.604270
H	3.395769	0.898395	-2.856405
C	5.478814	1.783850	-0.314793
H	4.862600	2.079020	1.731991
H	5.779691	1.426928	-2.420475
H	6.521931	2.018023	-0.122228
C	1.038590	-0.352622	-1.629330

C	-0.019477	-2.547784	-2.402564
C	1.429655	-2.554730	-2.377713
H	1.996659	-3.450165	-2.597696
C	-0.568443	-3.589872	-0.834369
C	0.368580	-4.688220	-0.727363
C	1.324753	-4.341179	0.194664
C	0.941730	-3.074146	0.759081
N	-0.223058	-2.725713	0.208571
H	-0.828442	-1.902985	0.452919
H	-1.633298	-3.653591	-1.045211
H	0.358280	-5.571441	-1.352576
H	2.210124	-4.900344	0.462946
C	1.632269	-2.225577	1.721538
C	0.998599	-1.087183	2.258478
C	2.958794	-2.507943	2.090148
C	1.683669	-0.266429	3.148955
H	-0.028540	-0.851650	1.997716
C	3.640045	-1.673958	2.970116
H	3.463029	-3.372333	1.669780
C	3.004141	-0.550938	3.504024
H	1.182010	0.603310	3.563247
H	4.668170	-1.898188	3.238167
H	3.535524	0.098478	4.193759
S	-0.571280	-0.862081	-2.212966
C	1.976137	-1.418967	-1.870018
H	3.027091	-1.309289	-1.638464
H	-0.568843	-3.110883	-3.151047

TS3-E

C	1.502281	-1.098256	-1.210623
C	2.887339	2.936030	1.323608
C	2.905596	1.721728	0.628315
C	4.090846	3.393659	1.844674
C	4.099168	0.962461	0.455643
C	5.288321	2.663463	1.676157
H	4.114134	4.333788	2.389138
C	5.303066	1.458003	0.987961
C	3.747820	-0.218270	-0.282115
C	2.383844	-0.139382	-0.533564
H	6.211089	3.055582	2.094854
H	6.229253	0.902410	0.865615
C	4.695878	-1.295408	-0.711253
H	4.259044	-1.933031	-1.483662
H	4.979133	-1.947575	0.126162

H	5.620956	-0.863831	-1.112817
N	1.887547	1.045072	0.008806
H	0.896744	1.278747	0.111257
H	1.963122	3.495045	1.439156
C	1.657968	-2.523782	-0.845326
C	1.925784	-2.877072	0.489876
C	1.525632	-3.551305	-1.792438
C	2.022507	-4.212571	0.868554
H	2.058011	-2.088756	1.224502
C	1.630017	-4.888882	-1.413426
H	1.373610	-3.298886	-2.837284
C	1.871991	-5.225777	-0.081686
H	2.218282	-4.464756	1.907216
H	1.530249	-5.668233	-2.163676
H	1.952353	-6.268233	0.212924
C	0.523004	-0.665664	-2.059614
C	-0.955488	0.986940	-2.882222
C	-1.912946	-0.176437	-2.826390
H	-2.532023	-0.253434	-3.724001
C	-2.841672	-0.140829	-1.595926
C	-4.102653	-0.829414	-1.571578
C	-4.261084	-1.451689	-0.349961
C	-3.072601	-1.220280	0.396027
N	-2.239446	-0.500432	-0.358552
H	-1.453999	0.044440	0.022931
H	-3.009772	1.195432	-1.319292
H	-4.797872	-0.850735	-2.401601
H	-5.106548	-2.032792	-0.009277
C	-2.705288	-1.650917	1.742883
C	-1.350948	-1.843866	2.065635
C	-3.687316	-1.898563	2.713370
C	-0.990238	-2.266023	3.340583
H	-0.588469	-1.700413	1.306772
C	-3.320115	-2.319925	3.988604
H	-4.733648	-1.738187	2.471446
C	-1.972219	-2.502489	4.305421
H	0.058239	-2.422033	3.576090
H	-4.085325	-2.501523	4.737491
H	-1.688597	-2.833319	5.300313
H	-1.314154	1.974889	-3.144064
S	-0.796645	-1.670456	-2.743330
C	0.282945	0.725214	-2.445972
H	1.050003	1.478380	-2.317295
P	-1.681966	2.755658	-0.074700

O	-2.893500	2.392918	-0.963086
O	-0.834341	1.651155	0.503029
O	-0.687183	3.729849	-0.903234
O	-2.312910	3.698979	1.075747
C	-1.442045	4.107159	2.142909
C	-1.247404	4.849872	-1.602969
H	-0.637084	4.745935	1.761896
H	-2.057388	4.672966	2.844207
H	-1.010430	3.233772	2.639391
H	-1.974628	4.512629	-2.347685
H	-1.738634	5.535891	-0.904985
H	-0.415005	5.356211	-2.094318

TS3-Z

P	-1.713041	2.193934	-0.700427
O	-2.813952	3.216610	-0.091830
O	-0.449607	3.125167	-1.078564
O	-1.223863	1.224162	0.353152
O	-2.371079	1.665297	-1.988027
C	2.208977	-1.292529	-0.438110
C	1.910526	3.429810	1.073168
C	2.391785	2.173882	0.683465
C	2.843180	4.376735	1.475526
C	3.781020	1.858662	0.704739
C	4.226366	4.089455	1.491948
H	2.502620	5.362656	1.781072
C	4.700845	2.842873	1.110663
C	3.916250	0.499795	0.269244
C	2.630420	0.035924	-0.000058
H	4.924870	4.858385	1.810616
H	5.765721	2.625855	1.130965
C	5.219081	-0.223314	0.108502
H	5.141557	-1.056103	-0.593788
H	5.580323	-0.637211	1.059592
H	5.990935	0.460773	-0.263586
N	1.719678	1.062984	0.244204
H	0.702593	0.989687	0.218843
H	0.849605	3.647162	1.042222
C	-0.513863	3.990225	-2.218873
H	-0.675738	3.407167	-3.129153
H	0.447826	4.503290	-2.265274
H	-1.321285	4.721995	-2.105578
C	-2.506335	3.863251	1.149682
H	-2.276874	3.125011	1.923982

H	-3.392279	4.435531	1.430763
H	-1.653822	4.541784	1.031899
C	2.956556	-2.445434	0.128142
C	3.232975	-2.499253	1.504103
C	3.432105	-3.482969	-0.689967
C	3.943823	-3.566442	2.046357
H	2.887223	-1.690965	2.140706
C	4.144547	-4.551303	-0.147008
H	3.262434	-3.429630	-1.761117
C	4.401274	-4.598739	1.223660
H	4.142646	-3.593062	3.114262
H	4.512194	-5.339507	-0.798415
H	4.962007	-5.427449	1.646690
C	1.168489	-1.507184	-1.298441
C	-1.029682	-1.318161	-2.739867
C	-0.586732	-2.704619	-2.351761
H	-1.215421	-3.554365	-2.599854
C	-2.346857	-0.940776	-2.046987
C	-3.609160	-1.491372	-2.468193
C	-4.377401	-1.758605	-1.357012
C	-3.600335	-1.422270	-0.210190
N	-2.387476	-1.044582	-0.625670
H	-1.732595	-0.487786	-0.058412
H	-2.436871	0.370266	-2.134567
H	-3.897253	-1.625880	-3.503302
H	-5.405528	-2.090956	-1.334055
C	-4.015523	-1.388901	1.192564
C	-3.372214	-0.549408	2.119308
C	-5.077199	-2.202378	1.620548
C	-3.787762	-0.538043	3.448007
H	-2.574857	0.113371	1.795318
C	-5.483406	-2.187044	2.951280
H	-5.569683	-2.862147	0.912756
C	-4.839177	-1.354695	3.868884
H	-3.290435	0.117473	4.156781
H	-6.300277	-2.826147	3.273068
H	-5.157450	-1.341328	4.907256
S	0.371561	-0.217478	-2.249071
C	0.509901	-2.774822	-1.581258
H	0.873535	-3.694903	-1.140216
H	-1.187348	-1.205140	-3.817914

VIII. Determination of the Absolute Stereochemistry

The structure and absolute stereochemistry of product **5a** were determined by X-ray diffraction. The X-ray data have been deposited at the Cambridge Crystallographic Data Center (CCDC 2022226). The stereochemistry of other products was assumed by analogy.

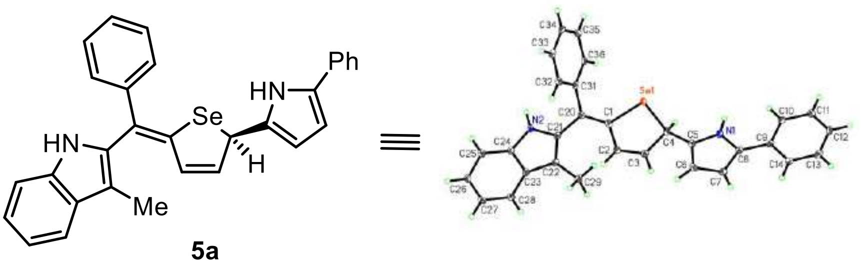

5a

Supplementary Table. 15. Crystal data and structure refinement for **5a.**

Identification code	**5a**
Empirical formula	$C_{30}H_{24}N_2Se$
Formula weight	491.47
Temperature/K	100.01(10)
Crystal system	monoclinic
Space group	$P2_1$
a/Å	14.3021(3)
b/Å	5.48120(8)
c/Å	15.6544(3)
α/°	90
β/°	113.745(2)
γ/°	90
Volume/Å^3	1123.30(4)
Z	2
ρ_{calc}g/cm^3	1.453
μ/mm^{-1}	2.414
F(000)	504.0
Crystal size/mm^3	0.08 × 0.02 × 0.02
Radiation	CuKα (λ = 1.54184)
2Θ range for data collection/°	7.078 to 134.828
Index ranges	$-17 \leq h \leq 16, -5 \leq k \leq 6, -14 \leq l \leq 18$
Reflections collected	6097
Independent reflections	3629 [R_{int} = 0.0358, R_{sigma} = 0.0518]
Data/restraints/parameters	3629/1/299
Goodness-of-fit on F^2	1.006
Final R indexes [I>=2σ (I)]	R_1 = 0.0268, wR_2 = 0.0576

Final R indexes [all data]	R_1 = 0.0301, wR_2 = 0.0590
Largest diff. peak/hole / e Å^{-3}	0.28/-0.30
Flack parameter	-0.023(16)

Supplementary Table. 16. Fractional Atomic Coordinates (×10^4) and Equivalent Isotropic Displacement Parameters (Å^2×10^3) for 5a. U_{eq} is defined as 1/3 of the trace of the orthogonalised U_{IJ} tensor.

**Atom**	***x***	***y***	***z***	**U(eq)**
Se1	5685.8(2)	4626.5(8)	2629.1(2)	18.20(10)
N1	7985(2)	5243(5)	4967.7(19)	14.4(7)
N2	1582.5(19)	4397(7)	1132.4(18)	15.1(6)
C1	4301(2)	4698(11)	2528(2)	15.2(6)
C2	4320(2)	4478(9)	3460(2)	14.4(6)
C3	5220(3)	4126(6)	4169(2)	16.1(9)
C4	6179(3)	4064(6)	4000(2)	15.7(8)
C5	6970(3)	5806(7)	4610(2)	14.5(7)
C6	6866(3)	7986(7)	4996(3)	16.6(8)
C7	7854(3)	8770(7)	5599(3)	16.8(8)
C8	8545(3)	7057(7)	5570(2)	14.0(7)
C9	9660(3)	7002(7)	6018(2)	13.9(7)
C10	10221(3)	5088(7)	5861(2)	15.8(9)
C11	11279(3)	5111(7)	6266(2)	16.7(9)
C12	11797(3)	7020(8)	6837(3)	19.0(8)
C13	11252(3)	8910(6)	7014(3)	18.4(8)
C14	10197(3)	8893(6)	6618(2)	16.0(8)
C20	3473(2)	4948(8)	1717(2)	12.6(7)
C21	2473(3)	5589(7)	1717(2)	14.7(7)
C22	2218(3)	7448(6)	2165(2)	13.4(7)
C23	1132(3)	7440(6)	1843(2)	14.0(7)
C24	752(3)	5488(6)	1207(2)	13.6(7)
C25	-288(2)	4976(8)	764(2)	15.9(8)
C26	-950(3)	6499(7)	964(3)	16.4(8)
C27	-589(3)	8498(7)	1579(3)	18.0(8)
C28	439(3)	8971(6)	2017(2)	15.7(8)
C29	2911(3)	9298(8)	2828(2)	19.1(8)
C31	3533(2)	4729(10)	792(2)	13.7(6)
C32	3083(3)	6519(7)	117(3)	17.0(8)
C33	3132(3)	6372(7)	-747(3)	17.8(8)
C34	3604(2)	4406(10)	-958(2)	18.9(8)
C35	4040(3)	2583(7)	-304(3)	19.0(8)
C36	4009(3)	2752(7)	574(2)	15.6(7)

Supplementary Table. 17. Anisotropic Displacement Parameters (Å^2×10^3) for 5a. The

Anisotropic displacement factor exponent takes the form: $-2\pi^2[h^2a^{*2}U_{11}+2hka^*b^*U_{12}+\ldots]$.

Atom	U_{11}	U_{22}	U_{33}	U_{23}	U_{13}	U_{12}
Se1	9.64(15)	31.2(2)	12.74(15)	0.7(2)	3.50(11)	2.0(2)
N1	11.6(13)	15.3(19)	13.9(13)	-0.6(11)	2.7(11)	0.3(11)
N2	11.6(12)	17.2(16)	14.9(12)	-2.9(15)	3.5(10)	-1.0(15)
C1	11.3(13)	16.4(15)	17.8(15)	0(2)	5.7(11)	-1(2)
C2	12.9(14)	16.0(17)	15.0(14)	-0.3(19)	6.4(11)	-1.8(19)
C3	17.6(17)	16(2)	14.2(15)	0.9(14)	6.4(13)	-1.6(14)
C4	13.7(16)	19(2)	11.8(15)	2.5(13)	2.3(13)	2.3(13)
C5	15.1(18)	16.7(17)	11.1(17)	4.8(14)	4.7(14)	1.8(14)
C6	13.9(18)	18.8(19)	19.2(19)	3.0(15)	8.9(15)	1.8(14)
C7	19.6(19)	16.5(18)	15.0(17)	0.6(13)	8.0(15)	-0.4(13)
C8	15.2(17)	14.9(17)	12.0(16)	-0.2(13)	5.5(14)	-2.9(13)
C9	15.8(18)	16.8(18)	8.2(16)	1.0(13)	3.9(14)	-3.1(14)
C10	16.1(16)	19(2)	12.1(14)	-2.6(14)	5.6(12)	-3.2(14)
C11	17.2(16)	18(3)	15.3(15)	2.4(14)	7.3(13)	0.7(14)
C12	11.5(18)	29(2)	14.3(18)	2.6(15)	3.1(14)	-2.2(15)
C13	21.5(18)	19(2)	13.8(16)	-0.4(13)	6.2(14)	-7.1(13)
C14	17.9(18)	15.9(19)	14.9(17)	-1.3(13)	7.4(14)	-0.8(13)
C20	11.8(14)	11(2)	16.3(14)	-1.1(15)	6.7(12)	-3.0(15)
C21	15.4(18)	16.1(16)	11.5(16)	1.4(13)	4.4(14)	-0.7(13)
C22	15.2(18)	13.5(18)	10.4(16)	1.3(14)	4.0(14)	-0.4(14)
C23	14.4(18)	15.3(18)	11.5(16)	2.2(14)	4.4(14)	0.6(14)
C24	15.0(17)	13.7(17)	12.2(16)	3.0(13)	5.7(14)	0.8(13)
C25	13.3(15)	17(2)	16.3(14)	2.7(16)	5.0(12)	-0.6(16)
C26	10.4(17)	22(2)	15.3(18)	5.0(15)	3.8(14)	0.7(15)
C27	18.8(19)	18.5(18)	20.7(19)	5.5(15)	12.2(16)	8.0(15)
C28	17.7(17)	16(2)	14.7(16)	0.0(13)	8.3(14)	1.7(13)
C29	16.4(15)	18(2)	18.7(16)	-3.8(17)	3.3(13)	-0.4(17)
C31	7.9(13)	17.9(16)	13.6(13)	-3(2)	2.7(11)	-6(2)
C32	12.7(18)	19.0(19)	19.0(19)	-1.6(15)	5.9(15)	-0.3(14)
C33	16.9(19)	20(2)	12.5(18)	0.8(14)	1.5(15)	-0.8(15)
C34	16.0(15)	28(2)	11.7(14)	-5.7(19)	4.8(12)	-4(2)
C35	14.3(19)	22(2)	20.4(19)	-5.5(16)	6.4(16)	0.6(15)
C36	9.9(17)	18.5(19)	15.2(17)	-1.2(14)	1.6(14)	-1.0(14)

Supplementary Table. 18. Bond Lengths for **5a.**

Atom	Atom	Length/Å	Atom	Atom	Length/Å
Se1	C1	1.923(3)	C12	C13	1.390(6)
Se1	C4	1.995(3)	C13	C14	1.382(5)
N1	C5	1.365(5)	C20	C21	1.472(5)
N1	C8	1.383(5)	C20	C31	1.490(4)

N2	C21	1.396(5)	C21	C22	1.367(5)
N2	C24	1.377(5)	C22	C23	1.427(5)
C1	C2	1.454(4)	C22	C29	1.503(5)
C1	C20	1.350(4)	C23	C24	1.413(5)
C2	C3	1.332(5)	C23	C28	1.406(5)
C3	C4	1.499(5)	C24	C25	1.393(5)
C4	C5	1.494(5)	C25	C26	1.390(5)
C5	C6	1.373(5)	C26	C27	1.412(6)
C6	C7	1.414(5)	C27	C28	1.373(5)
C7	C8	1.378(5)	C31	C32	1.395(6)
C8	C9	1.460(5)	C31	C36	1.393(6)
C9	C10	1.402(5)	C32	C33	1.385(5)
C9	C14	1.402(5)	C33	C34	1.380(6)
C10	C11	1.385(5)	C34	C35	1.387(6)
C11	C12	1.382(5)	C35	C36	1.396(5)

Supplementary Table. 19. Bond Angles for **5a.**

**Atom**	**Atom**	**Atom**	**Angle/°**	**Atom**	**Atom**	**Atom**	**Angle/°**
C1	Se1	C4	89.87(14)	C1	C20	C21	120.6(3)
C5	N1	C8	110.2(3)	C1	C20	C31	122.3(3)
C24	N2	C21	109.4(3)	C21	C20	C31	117.1(3)
C2	C1	Se1	108.3(2)	N2	C21	C20	121.2(3)
C20	C1	Se1	124.4(2)	C22	C21	N2	108.9(3)
C20	C1	C2	127.4(3)	C22	C21	C20	129.6(3)
C3	C2	C1	118.0(3)	C21	C22	C23	107.1(3)
C2	C3	C4	120.1(3)	C21	C22	C29	128.3(3)
C3	C4	Se1	103.5(2)	C23	C22	C29	124.5(3)
C5	C4	Se1	115.9(2)	C24	C23	C22	107.8(3)
C5	C4	C3	112.6(3)	C28	C23	C22	133.0(3)
N1	C5	C4	121.6(3)	C28	C23	C24	119.2(3)
N1	C5	C6	107.6(3)	N2	C24	C23	106.8(3)
C6	C5	C4	130.4(3)	N2	C24	C25	130.9(3)
C5	C6	C7	107.6(3)	C25	C24	C23	122.3(4)
C8	C7	C6	108.0(3)	C26	C25	C24	117.0(4)
N1	C8	C9	122.2(3)	C25	C26	C27	121.6(3)
C7	C8	N1	106.6(3)	C28	C27	C26	120.8(3)
C7	C8	C9	131.1(3)	C27	C28	C23	119.1(3)
C10	C9	C8	121.5(3)	C32	C31	C20	119.4(4)
C14	C9	C8	120.3(3)	C36	C31	C20	121.7(4)
C14	C9	C10	118.2(3)	C36	C31	C32	118.9(3)
C11	C10	C9	120.7(3)	C33	C32	C31	120.7(4)
C12	C11	C10	120.4(3)	C34	C33	C32	120.1(4)
C11	C12	C13	119.7(3)	C33	C34	C35	120.2(3)

C14	C13	C12	120.3(3)	C34	C35	C36	119.8(3)
C13	C14	C9	120.7(3)	C31	C36	C35	120.4(3)

Supplementary Table. 20. Torsion Angles for **5a.**

A	B	C	D	Angle/°	A	B	C	D	Angle/°
Se1	C1	C2	C3	-4.1(6)	C11	C12	C13	C14	-0.3(6)
Se1	C1	C20	C21	-164.8(3)	C12	C13	C14	C9	-1.3(6)
Se1	C1	C20	C31	11.8(8)	C14	C9	C10	C11	-1.9(5)
Se1	C4	C5	N1	-97.8(3)	C20	C1	C2	C3	176.8(5)
Se1	C4	C5	C6	90.1(4)	C20	C21	C22	C23	173.0(4)
N1	C5	C6	C7	0.6(4)	C20	C21	C22	C29	-3.5(6)
N1	C8	C9	C10	-0.2(5)	C20	C31	C32	C33	179.6(3)
N1	C8	C9	C14	179.4(3)	C20	C31	C36	C35	179.1(3)
N2	C21	C22	C23	-0.6(4)	C21	N2	C24	C23	1.0(4)
N2	C21	C22	C29	-177.1(3)	C21	N2	C24	C25	179.5(4)
N2	C24	C25	C26	-177.4(4)	C21	C20	C31	C32	45.6(5)
C1	C2	C3	C4	2.4(6)	C21	C20	C31	C36	-133.0(4)
C1	C20	C21	N2	-136.0(5)	C21	C22	C23	C24	1.2(4)
C1	C20	C21	C22	51.1(7)	C21	C22	C23	C28	-176.5(4)
C1	C20	C31	C32	-131.1(5)	C22	C23	C24	N2	-1.4(4)
C1	C20	C31	C36	50.3(7)	C22	C23	C24	C25	-180.0(3)
C2	C1	C20	C21	14.2(8)	C22	C23	C28	C27	178.8(4)
C2	C1	C20	C31	-169.2(5)	C23	C24	C25	C26	0.8(5)
C2	C3	C4	Se1	0.6(4)	C24	N2	C21	C20	-174.5(3)
C2	C3	C4	C5	126.6(4)	C24	N2	C21	C22	-0.3(4)
C3	C4	C5	N1	143.3(3)	C24	C23	C28	C27	1.3(5)
C3	C4	C5	C6	-28.9(5)	C24	C25	C26	C27	0.9(5)
C4	C5	C6	C7	173.6(4)	C25	C26	C27	C28	-1.5(6)
C5	N1	C8	C7	1.2(4)	C26	C27	C28	C23	0.4(5)
C5	N1	C8	C9	-176.7(3)	C28	C23	C24	N2	176.7(3)
C5	C6	C7	C8	0.1(4)	C28	C23	C24	C25	-1.9(5)
C6	C7	C8	N1	-0.8(4)	C29	C22	C23	C24	177.9(3)
C6	C7	C8	C9	176.9(4)	C29	C22	C23	C28	0.2(6)
C7	C8	C9	C10	-177.5(4)	C31	C20	C21	N2	47.3(6)
C7	C8	C9	C14	2.1(6)	C31	C20	C21	C22	-125.6(4)
C8	N1	C5	C4	-174.8(3)	C31	C32	C33	C34	1.8(6)
C8	N1	C5	C6	-1.1(4)	C32	C31	C36	C35	0.5(5)
C8	C9	C10	C11	177.7(3)	C32	C33	C34	C35	-0.6(6)
C8	C9	C14	C13	-177.2(3)	C33	C34	C35	C36	-0.6(6)
C9	C10	C11	C12	0.3(5)	C34	C35	C36	C31	0.7(5)
C10	C9	C14	C13	2.4(5)	C36	C31	C32	C33	-1.7(5)
C10	C11	C12	C13	0.8(6)					

Supplementary Table. 21. Hydrogen Atom Coordinates (Å×10^4) and Isotropic Displacement Parameters (Å^2×10^3) for **5a.**

**Atom**	***x***	***y***	***z***	**U(eq)**
H1	8246	3916	4834	17
H2	1556	3143	772	18
H2A	3709	4581	3561	17
H3	5253	3914	4783	19
H4	6469	2378	4138	19
H6	6243	8815	4879	20
H7	8013	10222	5961	20
H10	9873	3762	5472	19
H11	11651	3807	6151	20
H12	12523	7039	7108	23
H13	11607	10221	7408	22
H14	9832	10177	6753	19
H25	-533	3646	343	19
H26	-1664	6187	681	20
H27	-1062	9529	1690	22
H28	679	10313	2433	19
H29A	2594	10916	2680	29
H29B	3567	9325	2768	29
H29C	3024	8861	3469	29
H32	2740	7853	251	20
H33	2840	7625	-1195	21
H34	3630	4302	-1554	23
H35	4358	1223	-452	23
H36	4314	1513	1025	19

Supplementary References

1. Yu, J. O. et al. 2-Indolylphosphines, a new class of tunable ligands: their synthesis, facile derivatization, and coordination to palladium(II). *Organometallics* **24**, 37−47 (2005).
2. Ip, H.-W., Ng, C.-F., Chow, H.-F. & Kuck, D. Three-fold scholl-type cycloheptatriene ring formation around a tribenzotriquinacene core: toward warped graphenes. *J. Am. Chem. Soc.* **138**, 13778−13781 (2016).
3. Ma, S. et al. Development of a general and practical iron nitrate/TEMPO-catalyzed aerobic oxidation of alcohols to aldehydes/ketones: catalysis with table salt. *Adv. Synth. Catal.* **353**, 1005−1017 (2011).

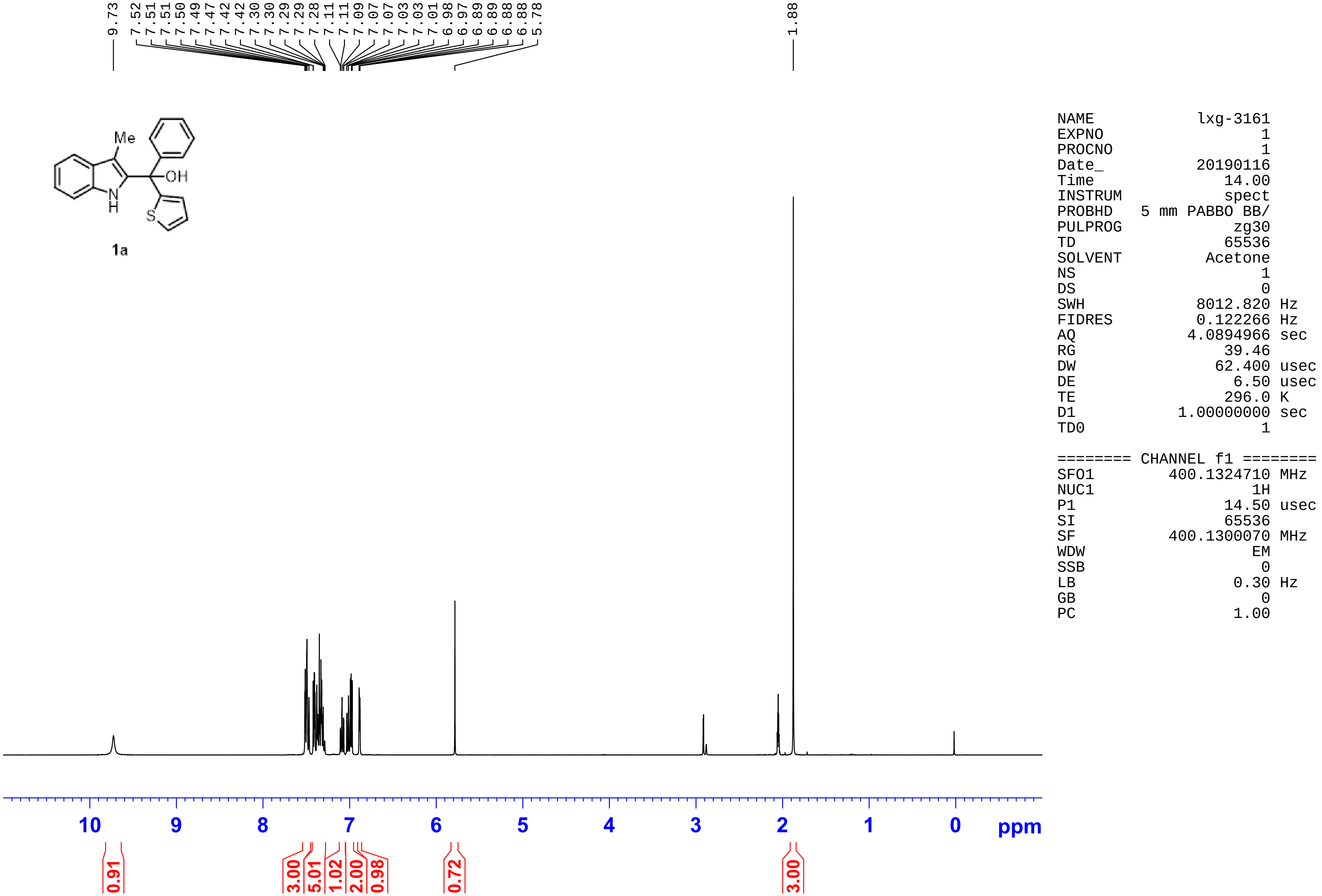

Supplementary Figure 1. ^{1}H NMR spectrum of **1a**

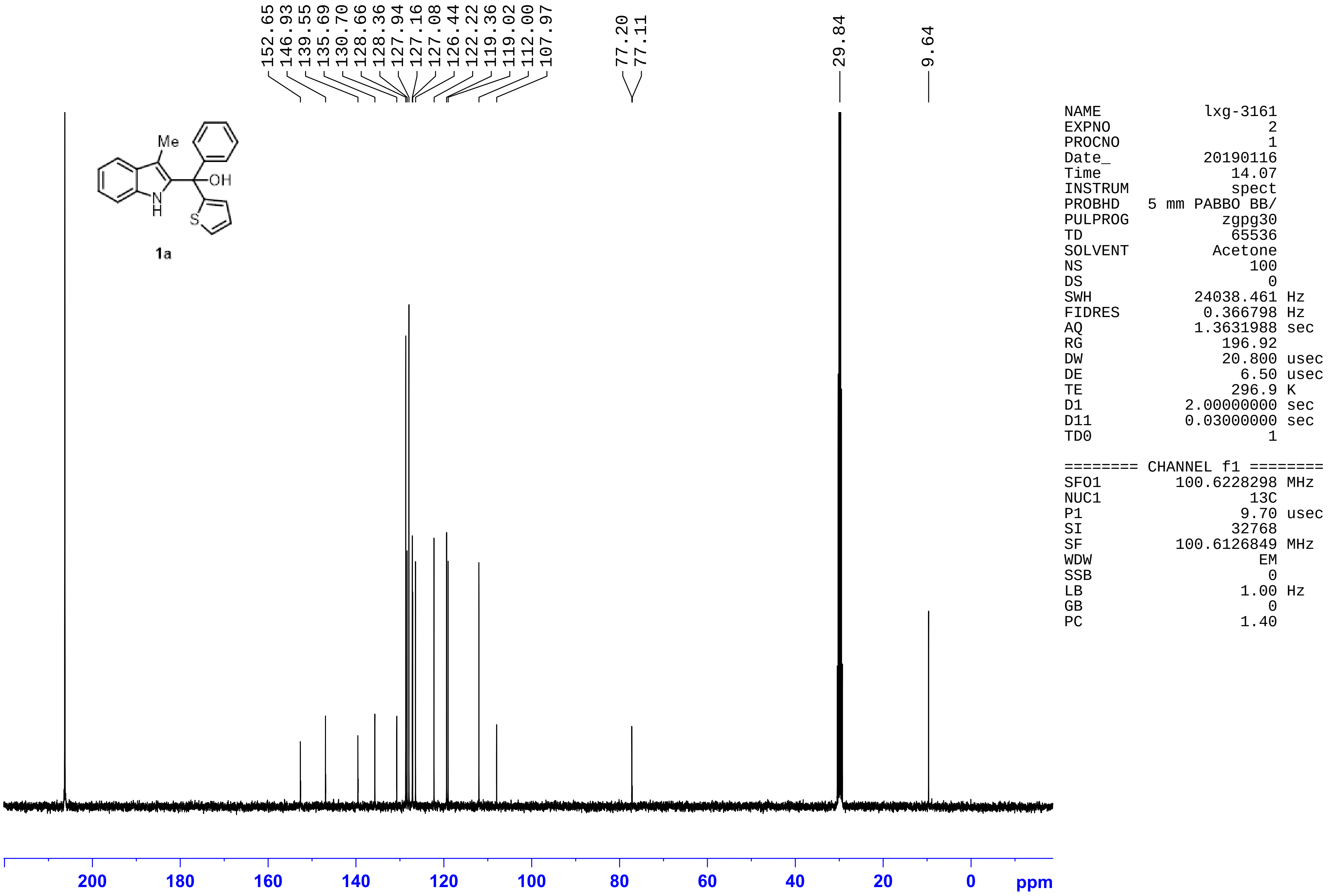

Supplementary Figure 2. ^{13}C NMR spectrum of **1a**

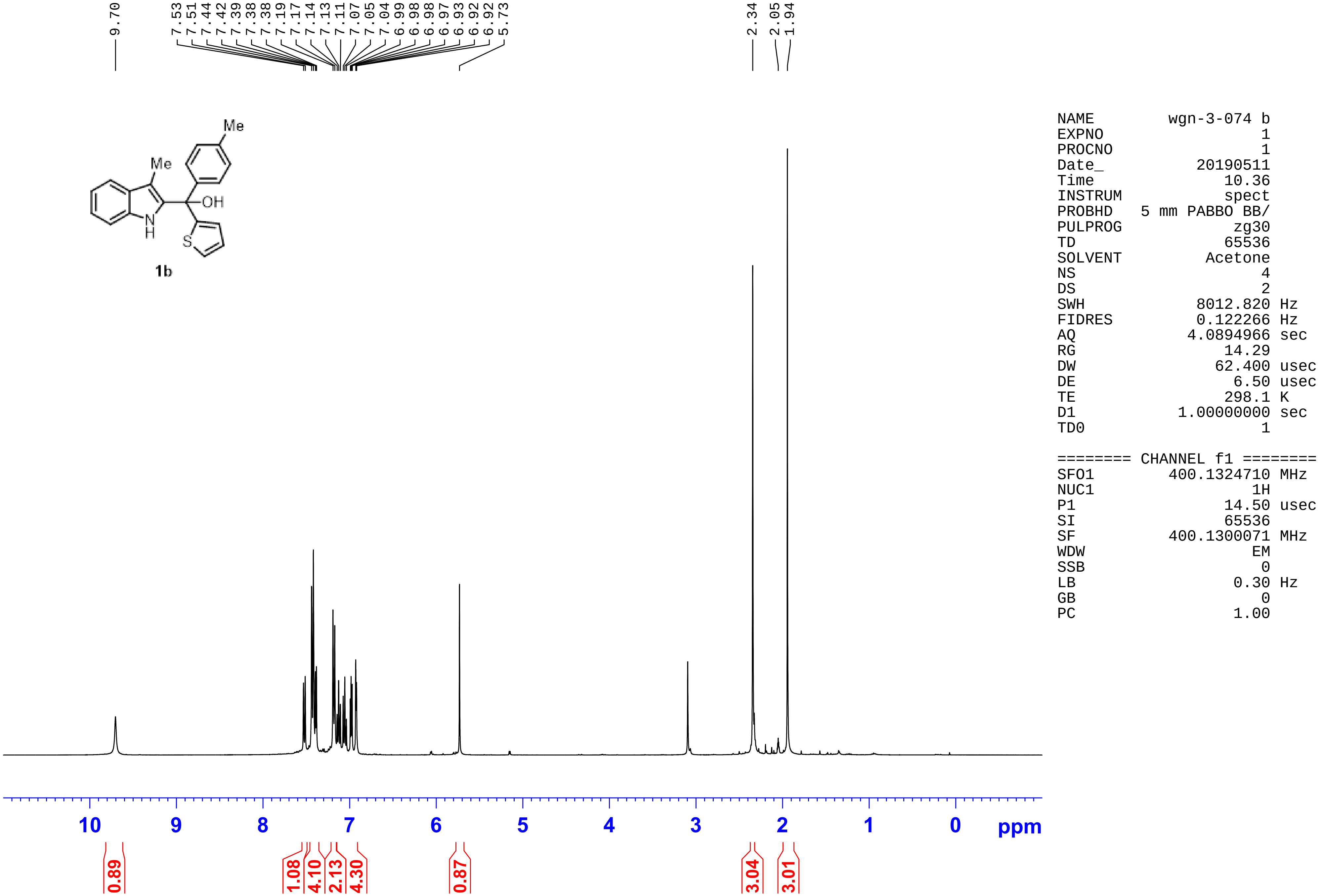

Supplementary Figure 3. ^{1}H NMR spectrum of **1b**

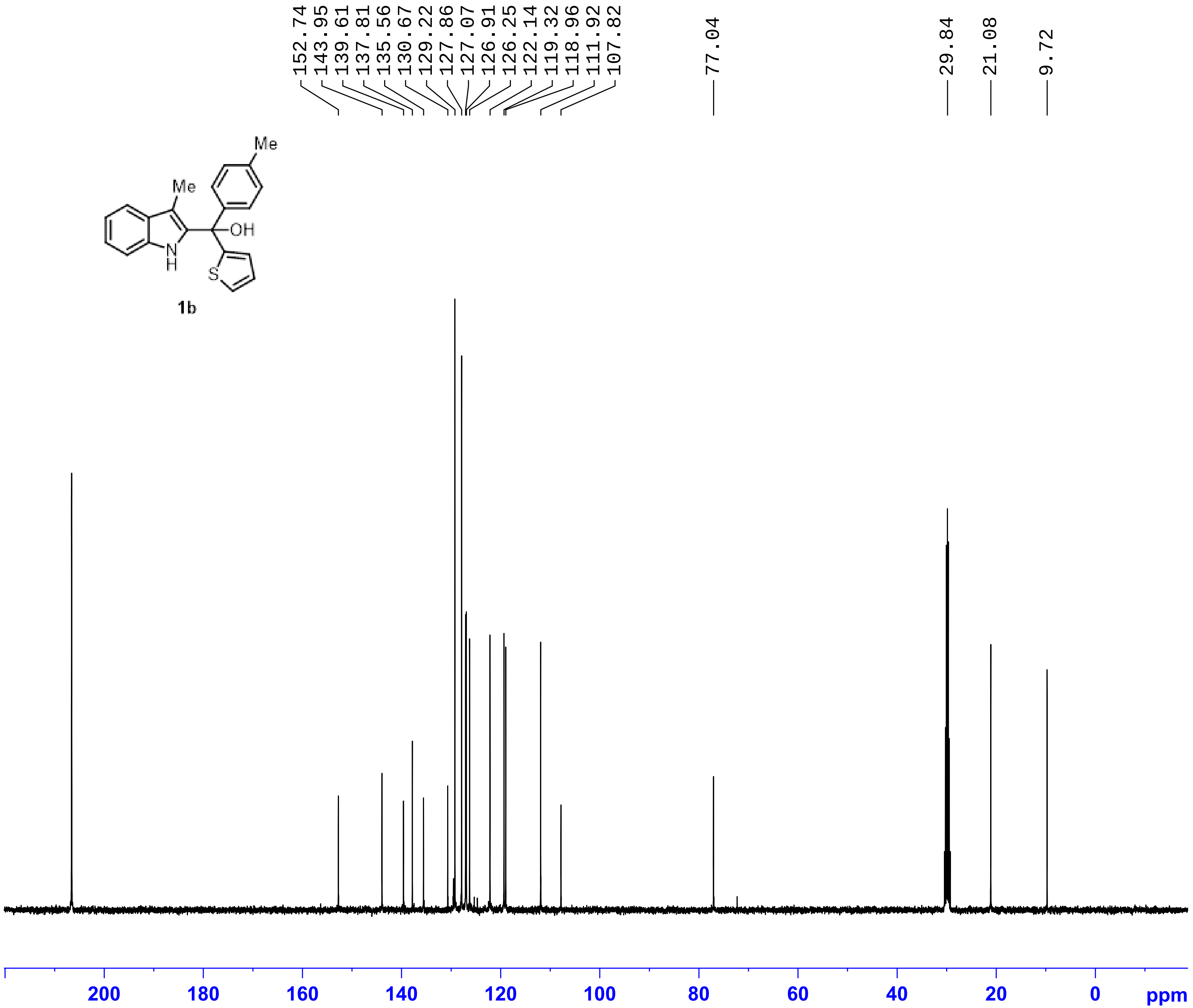

NAME	wgn-3-074 b-C
EXPNO	1
PROCNO	1
Date_	20190511
Time	10.39
INSTRUM	spect
PROBHD	5 mm PABBO BB/
PULPROG	zgpg30
TD	65536
SOLVENT	Acetone
NS	48
DS	2
SWH	24038.461 Hz
FIDRES	0.366798 Hz
AQ	1.3631988 sec
RG	196.92
DW	20.800 usec
DE	6.50 usec
TE	299.0 K
D1	2.00000000 sec
D11	0.03000000 sec
TD0	1

======== CHANNEL f1 ========

SFO1	100.6228298 MHz
NUC1	13C
P1	9.70 usec
SI	32768
SF	100.6126991 MHz
WDW	EM
SSB	0
LB	1.00 Hz
GB	0
PC	1.40

Supplementary Figure 4. ^{13}C NMR spectrum of **1b**

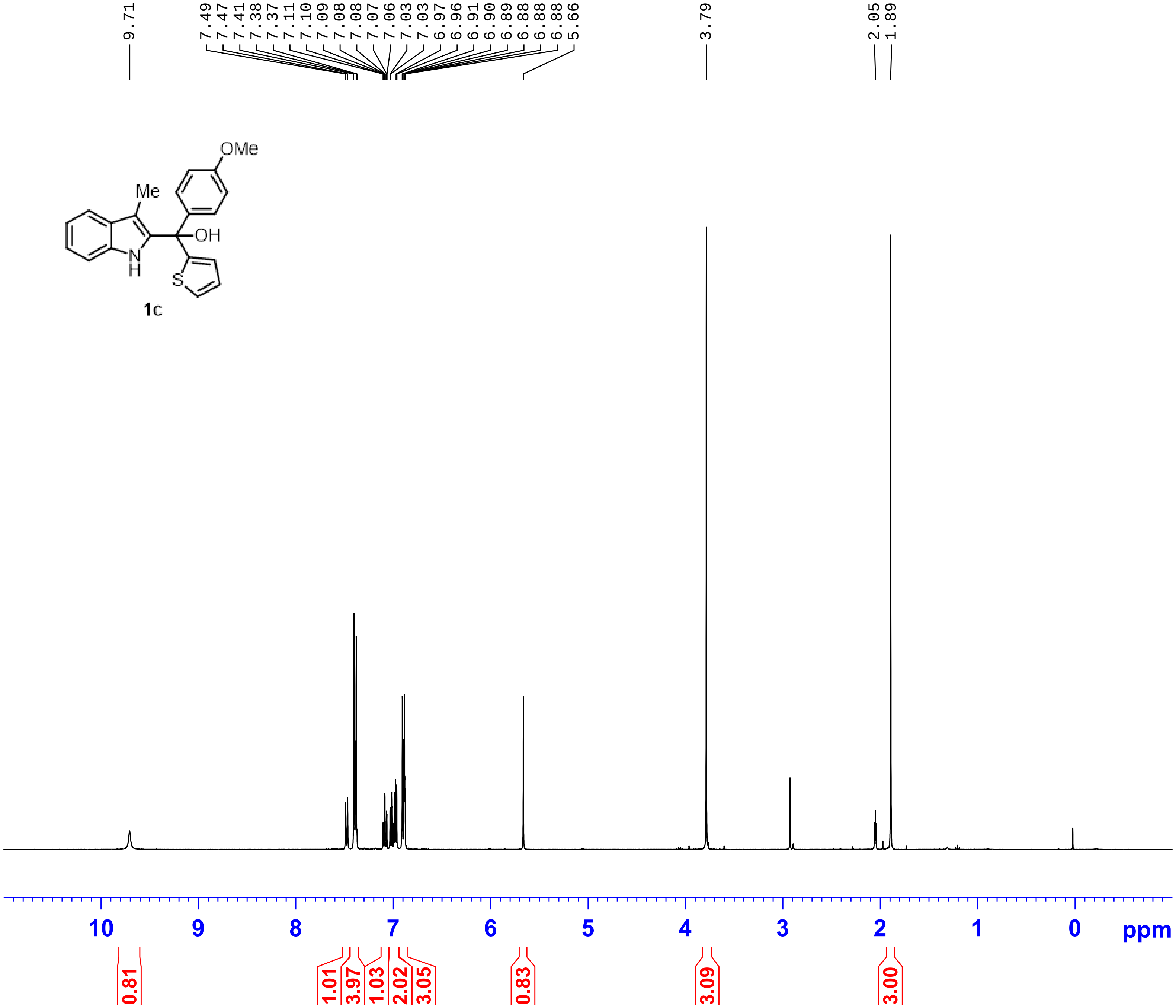

NAME	lxg-4013B	
EXPNO	2	
PROCNO	1	
Date_	20190218	
Time	19.03	
INSTRUM	spect	
PROBHD	5 mm PABBO BB/	
PULPROG	zg30	
TD	65536	
SOLVENT	Acetone	
NS	2	
DS	0	
SWH	8012.820	Hz
FIDRES	0.122266	Hz
AQ	4.0894966	sec
RG	45.67	
DW	62.400	usec
DE	6.50	usec
TE	296.3	K
D1	1.00000000	sec
TD0	1	

======== CHANNEL f1 ========

SFO1	400.1324710	MHz
NUC1	1H	
P1	14.50	usec
SI	65536	
SF	400.1300070	MHz
WDW	EM	
SSB	0	
LB	0.30	Hz
GB	0	
PC	1.00	

Supplementary Figure 5. ^{1}H NMR spectrum of **1c**

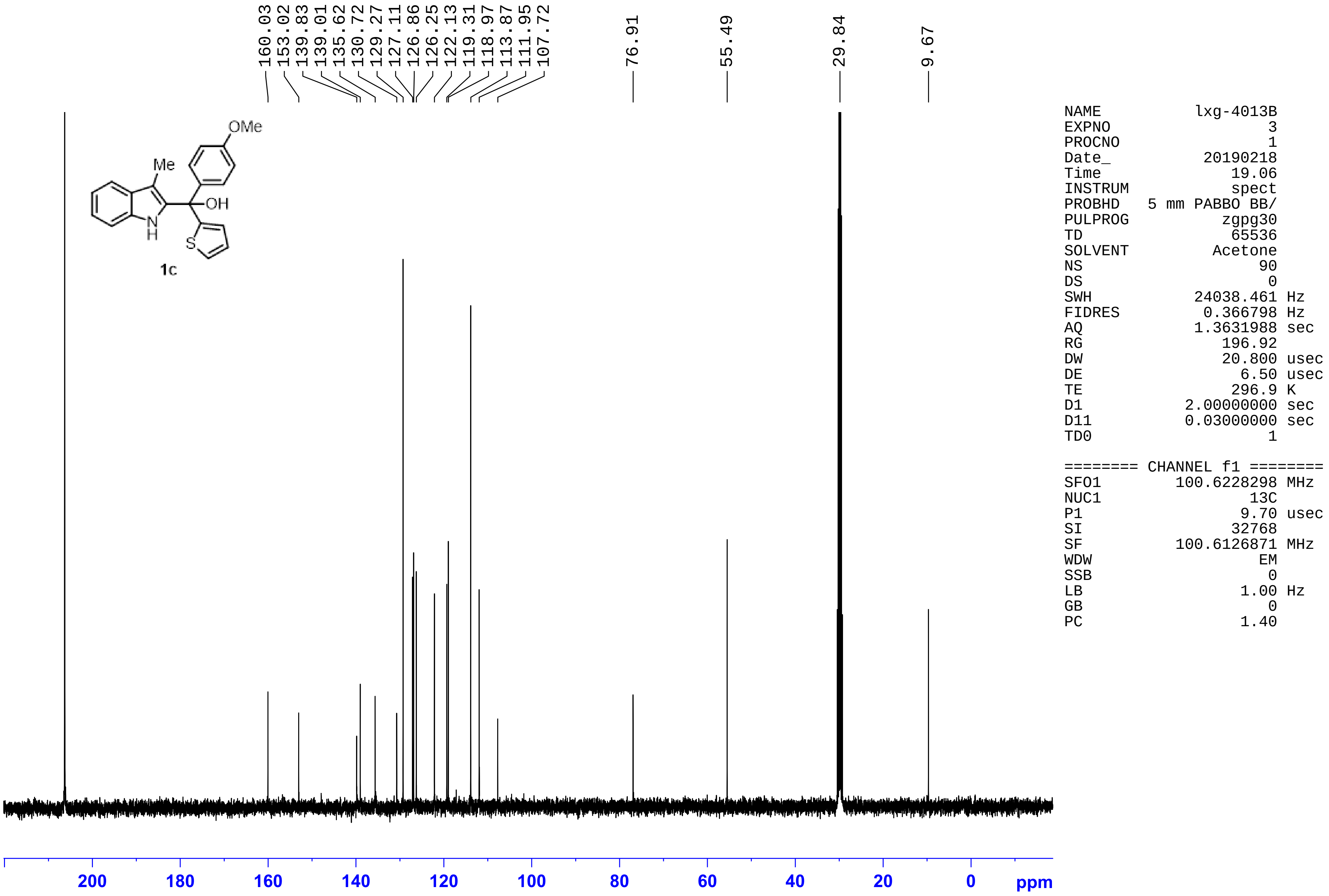

Supplementary Figure 6. ^{13}C NMR spectrum of **1c**

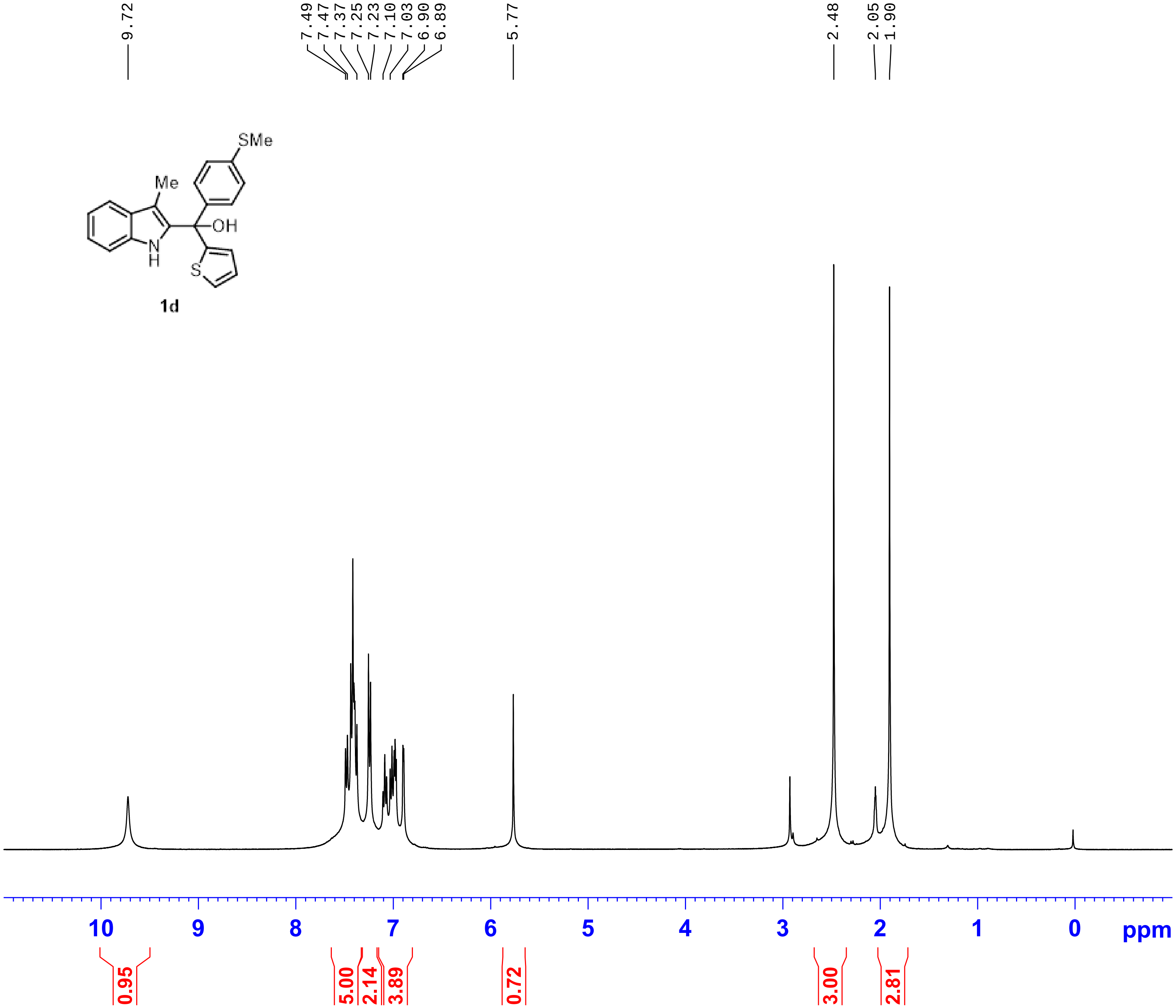

NAME	lxg-4019A
EXPNO	1
PROCNO	1
Date_	20190226
Time	20.10
INSTRUM	spect
PROBHD	5 mm PABBO BB/
PULPROG	zg30
TD	65536
SOLVENT	Acetone
NS	2
DS	0
SWH	8012.820 Hz
FIDRES	0.122266 Hz
AQ	4.0894966 sec
RG	31.55
DW	62.400 usec
DE	6.50 usec
TE	295.7 K
D1	1.00000000 sec
TD0	1

======== CHANNEL f1 ========

SFO1	400.1324710 MHz
NUC1	1H
P1	14.50 usec
SI	65536
SF	400.1300075 MHz
WDW	EM
SSB	0
LB	0.30 Hz
GB	0
PC	1.00

Supplementary Figure 7. ^{1}H NMR spectrum of **1d**

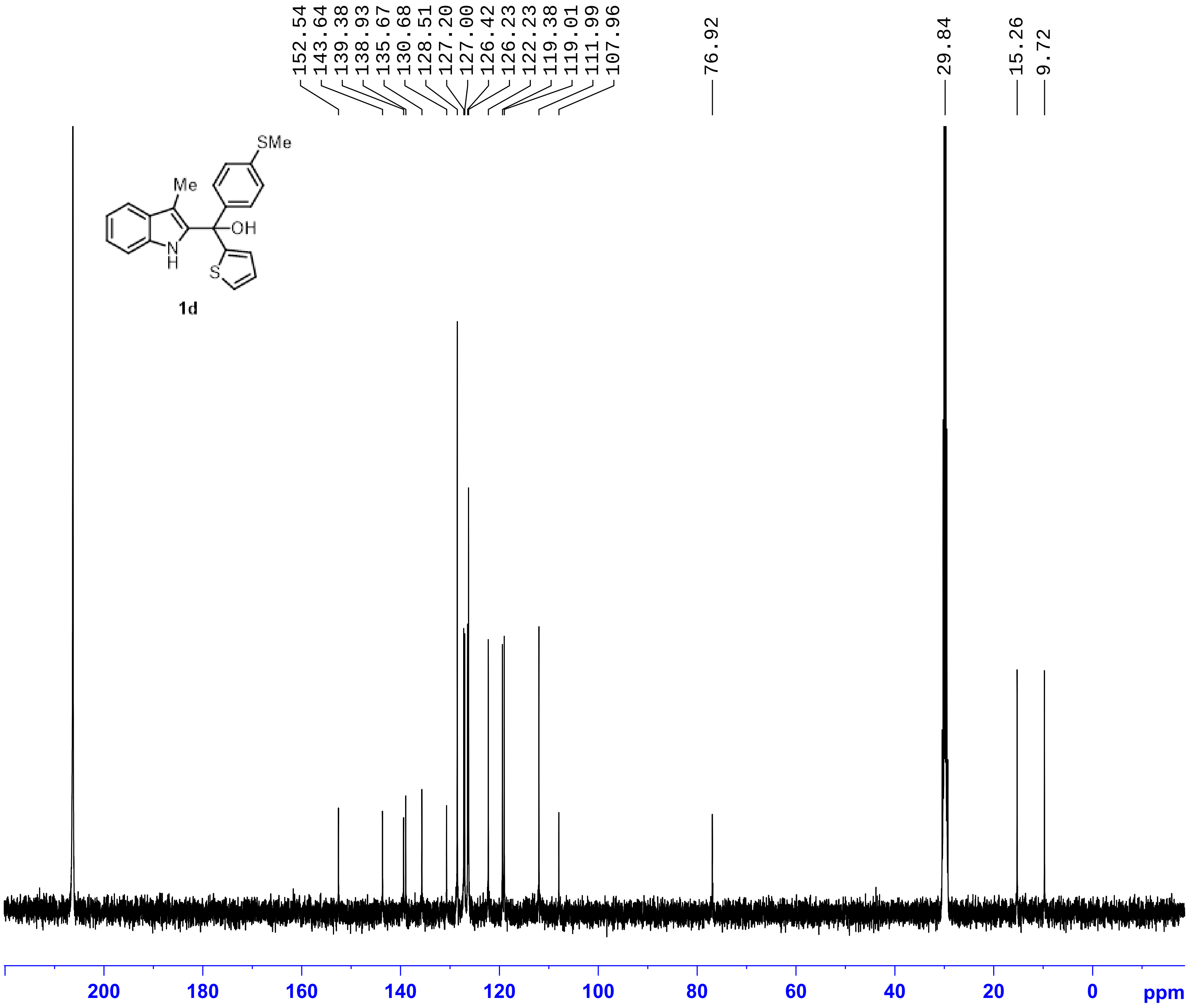


```
NAME             lxg-4019A
EXPNO                    3
PROCNO                   1
Date_             20190226
Time                 20.14
INSTRUM              spect
PROBHD   5 mm PABBO BB/
PULPROG            zgpg30
TD                  65536
SOLVENT            Acetone
NS                      81
DS                       0
SWH          24038.461 Hz
FIDRES        0.366798 Hz
AQ           1.3631988 sec
RG                  196.92
DW             20.800 usec
DE               6.50 usec
TE                 296.6 K
D1          2.00000000 sec
D11         0.03000000 sec
TD0                      1

======== CHANNEL f1 ========
SFO1       100.6228298 MHz
NUC1                   13C
P1               9.70 usec
SI                   32768
SF         100.6126871 MHz
WDW                     EM
SSB                      0
LB                 1.00 Hz
GB                       0
PC                    1.40
```


Supplementary Figure 8. ^{13}C NMR spectrum of **1d**

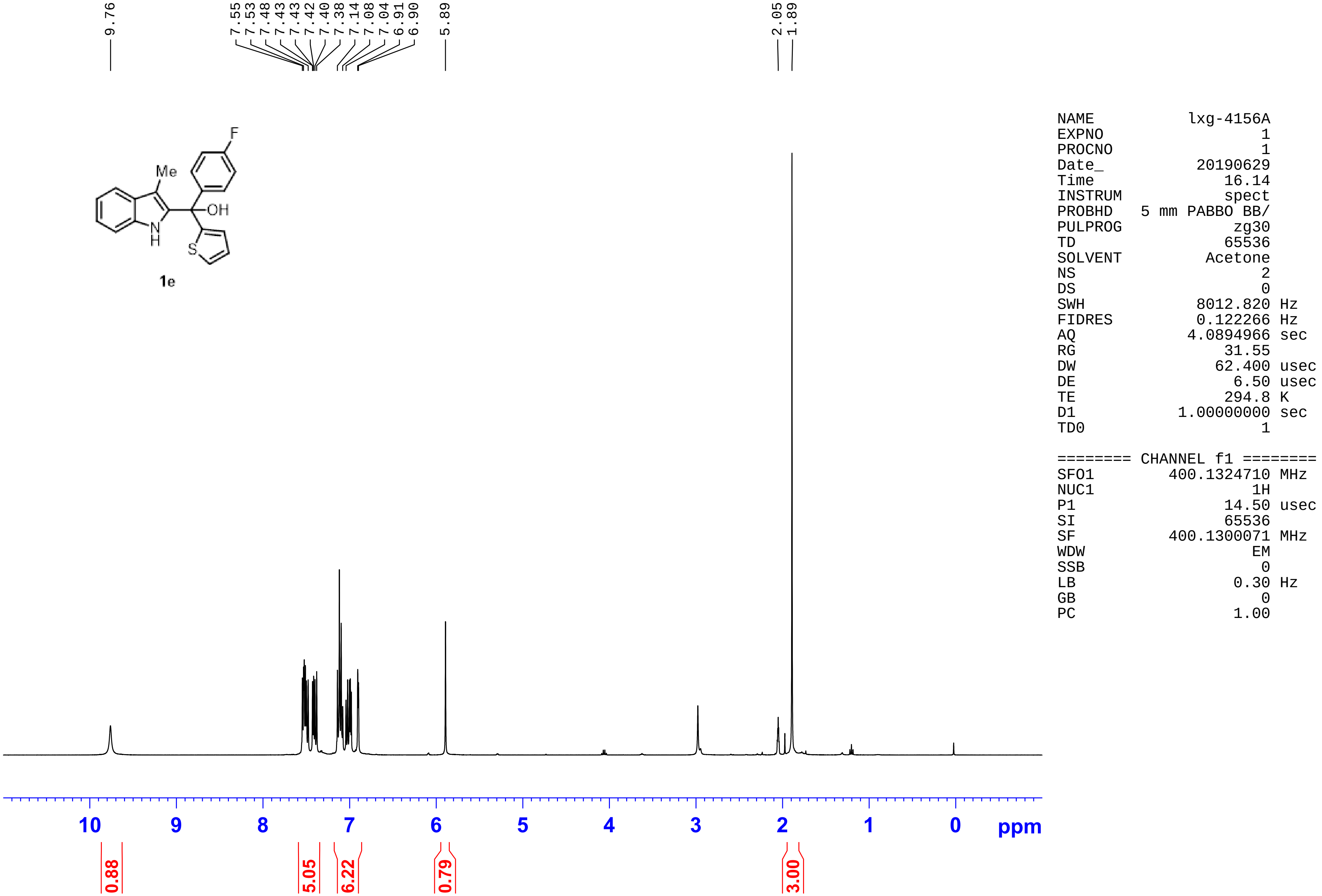

Supplementary Figure 9. ^{1}H NMR spectrum of **1e**

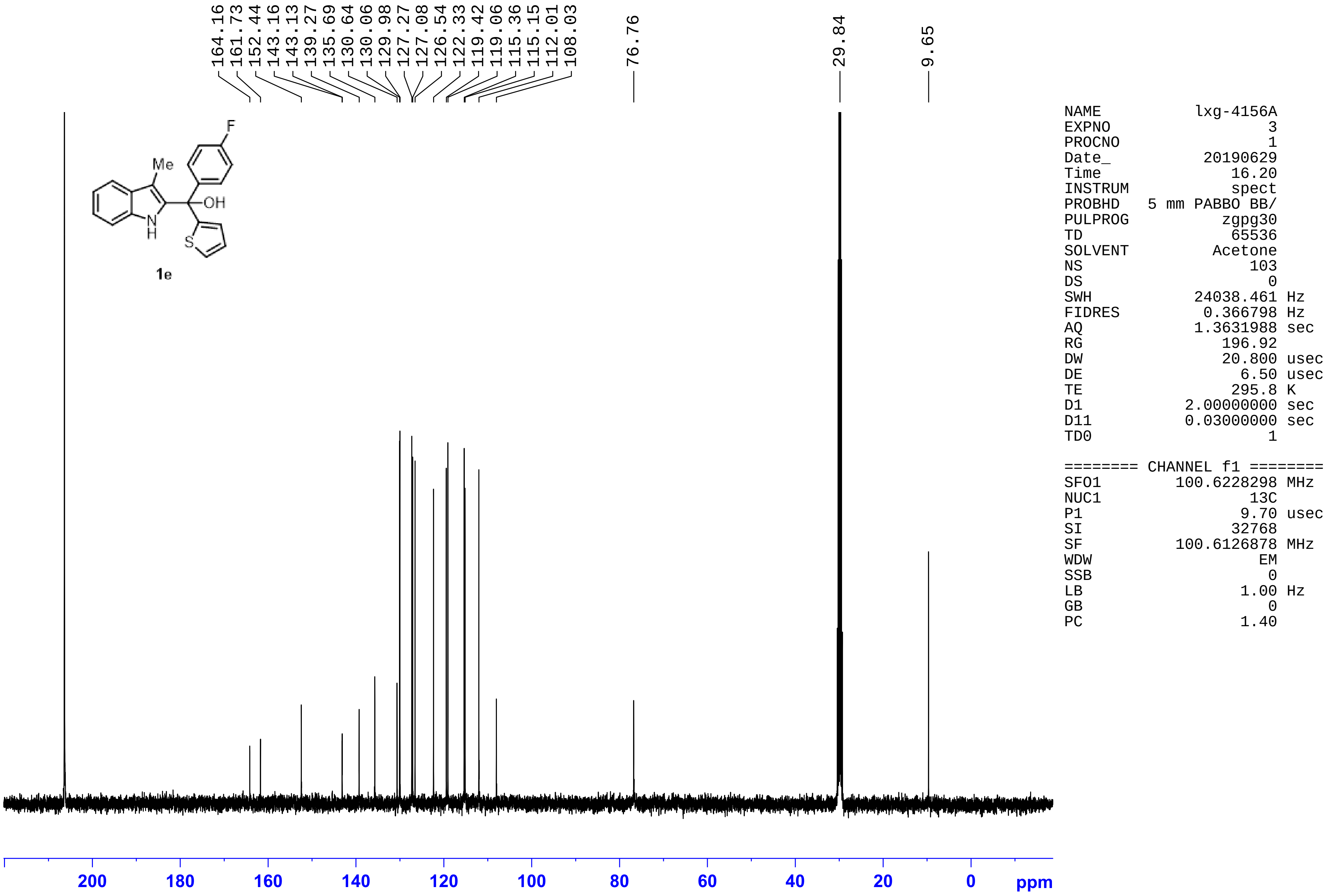

Supplementary Figure 10. ^{13}C NMR spectrum of **1e**

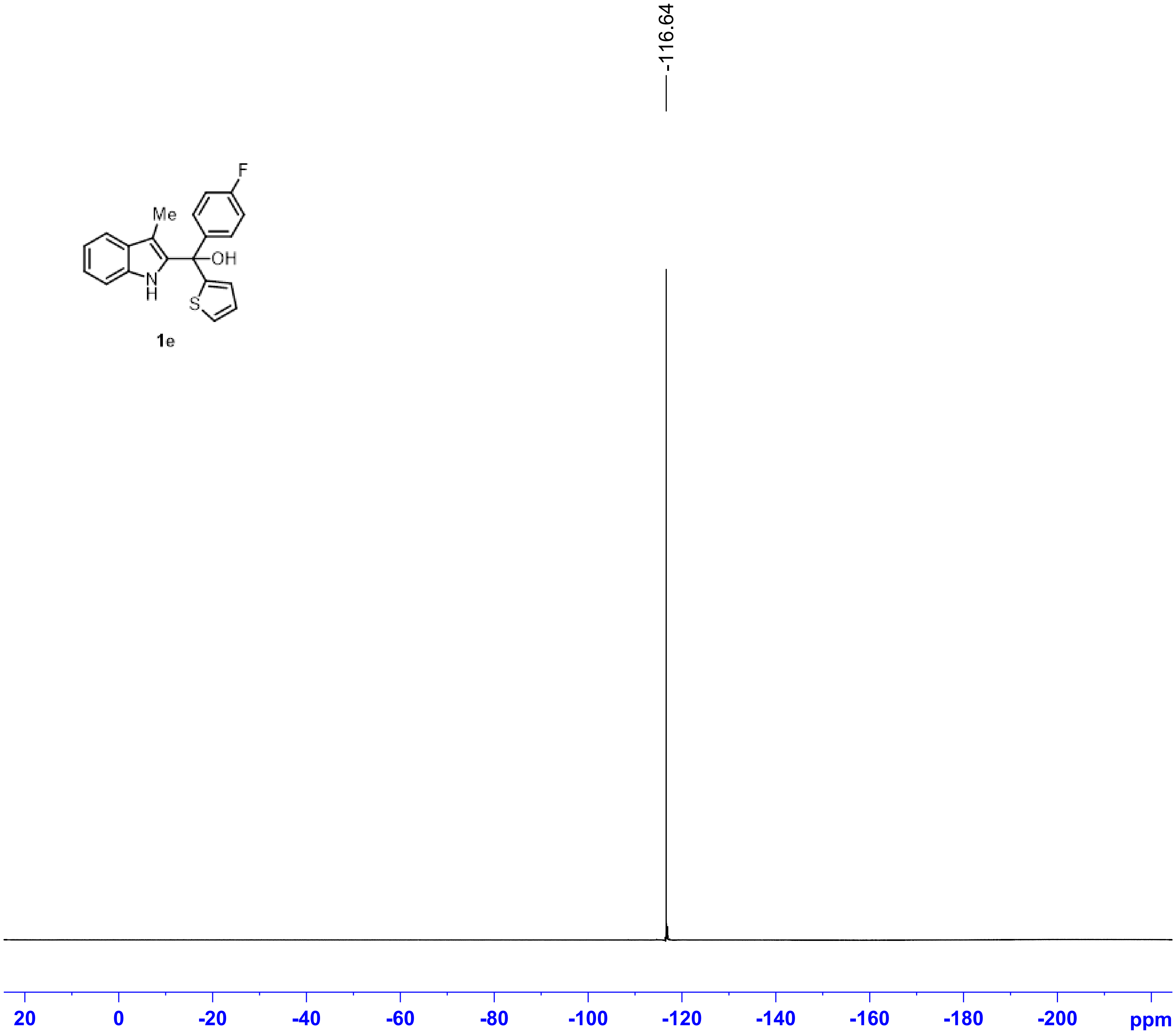

NAME	lxg-4156A	
EXPNO	4	
PROCNO	1	
Date_	20190629	
Time	16.27	
INSTRUM	spect	
PROBHD	5 mm PABBO BB/	
PULPROG	zgpg30	
TD	65536	
SOLVENT	Acetone	
NS	20	
DS	0	
SWH	93750.000	Hz
FIDRES	1.430511	Hz
AQ	0.3495753	sec
RG	196.92	
DW	5.333	usec
DE	6.50	usec
TE	295.7	K
D1	2.00000000	sec
D11	0.03000000	sec
TD0	1	

======== CHANNEL f1 ========		
SFO1	376.4607162	MHz
NUC1	19F	
P1	14.70	usec
SI	32768	
SF	376.4983660	MHz
WDW	EM	
SSB	0	
LB	1.00	Hz
GB	0	
PC	1.40	

Supplementary Figure 11. ^{19}F NMR spectrum of **1e**

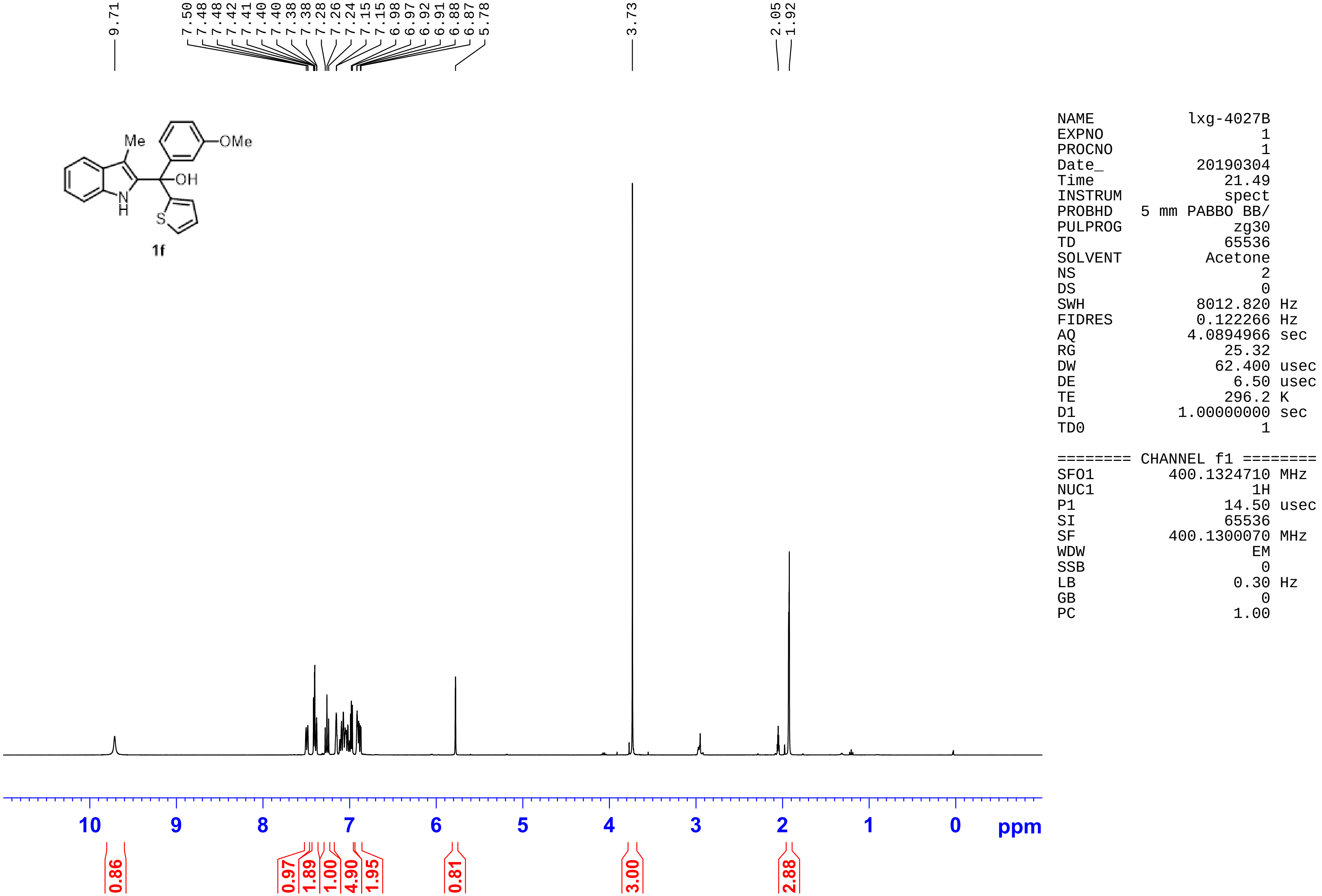

Supplementary Figure 12. ^{1}H NMR spectrum of **1f**

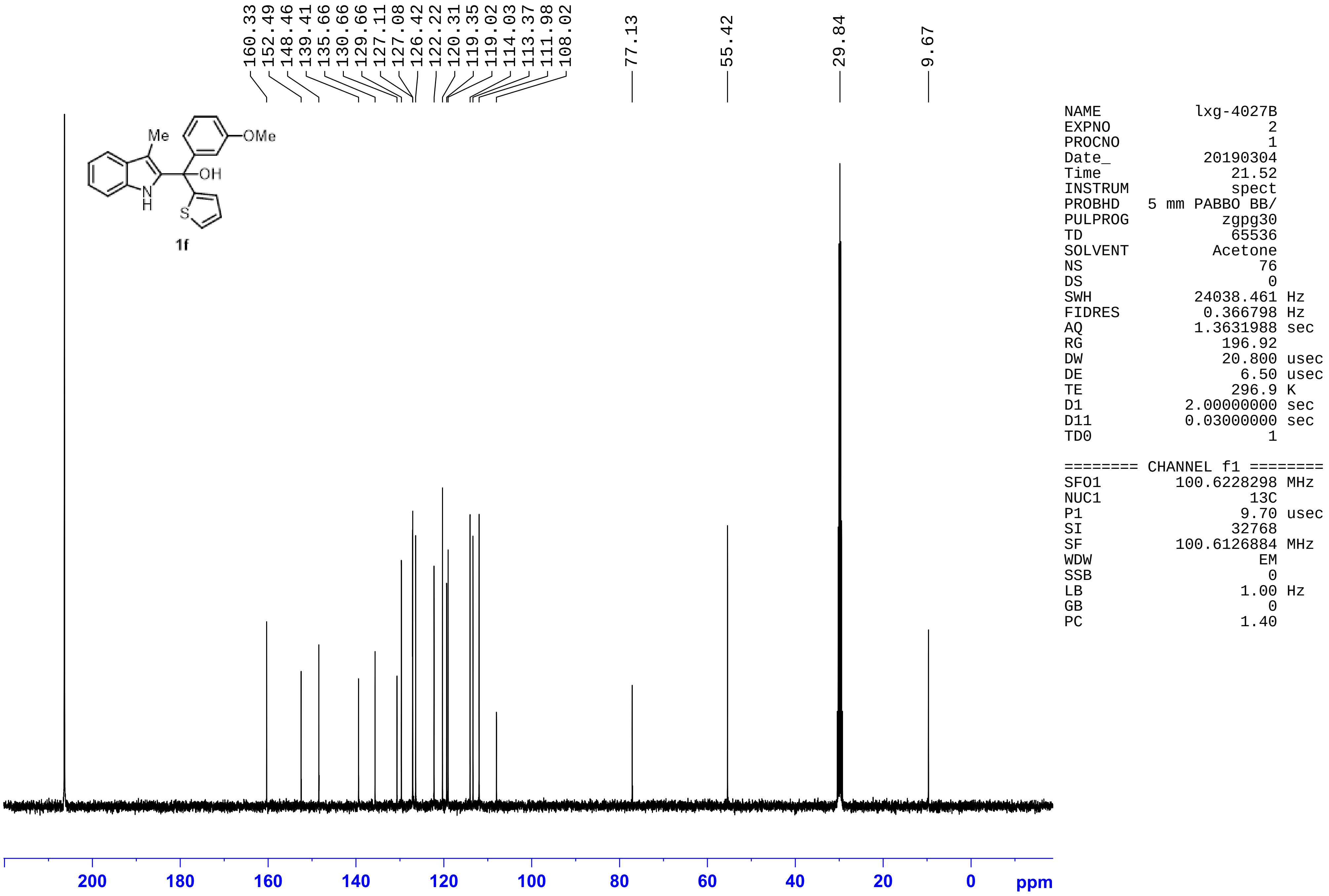

Supplementary Figure 13. ^{13}C NMR spectrum of **1f**

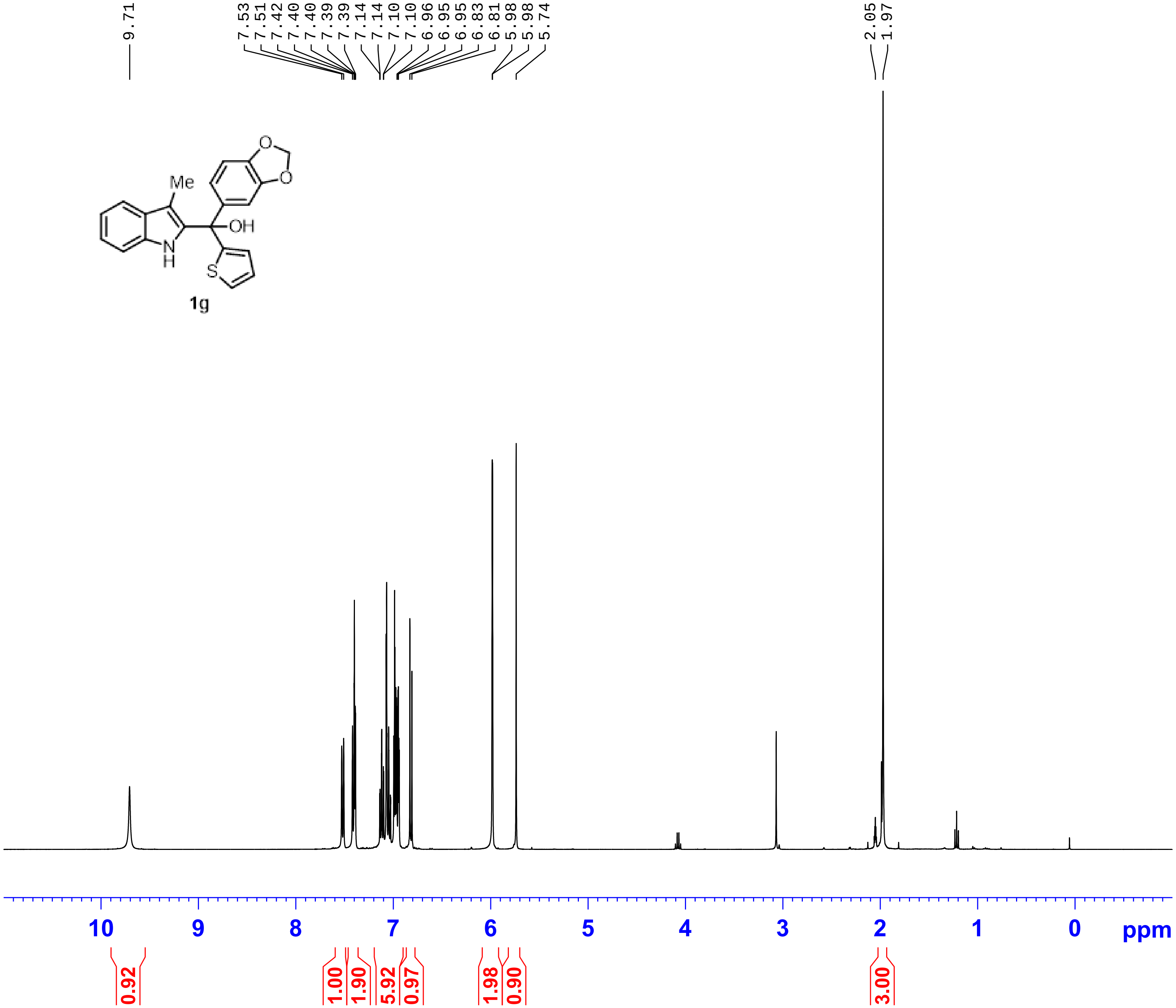

1g

NAME lxg-4030A
EXPNO 1
PROCNO 1
Date_ 20190423
Time 20.01
INSTRUM spect
PROBHD 5 mm PABBO BB/
PULPROG zg30
TD 65536
SOLVENT Acetone
NS 2
DS 0
SWH 8012.820 Hz
FIDRES 0.122266 Hz
AQ 4.0894966 sec
RG 19.7
DW 62.400 usec
DE 6.50 usec
TE 297.6 K
D1 1.00000000 sec
TD0 1

======== CHANNEL f1 ========
SFO1 400.1324710 MHz
NUC1 1H
P1 14.50 usec
SI 65536
SF 400.1300071 MHz
WDW EM
SSB 0
LB 0.30 Hz
GB 0
PC 1.00

Supplementary Figure 14. ^{1}H NMR spectrum of **1g**

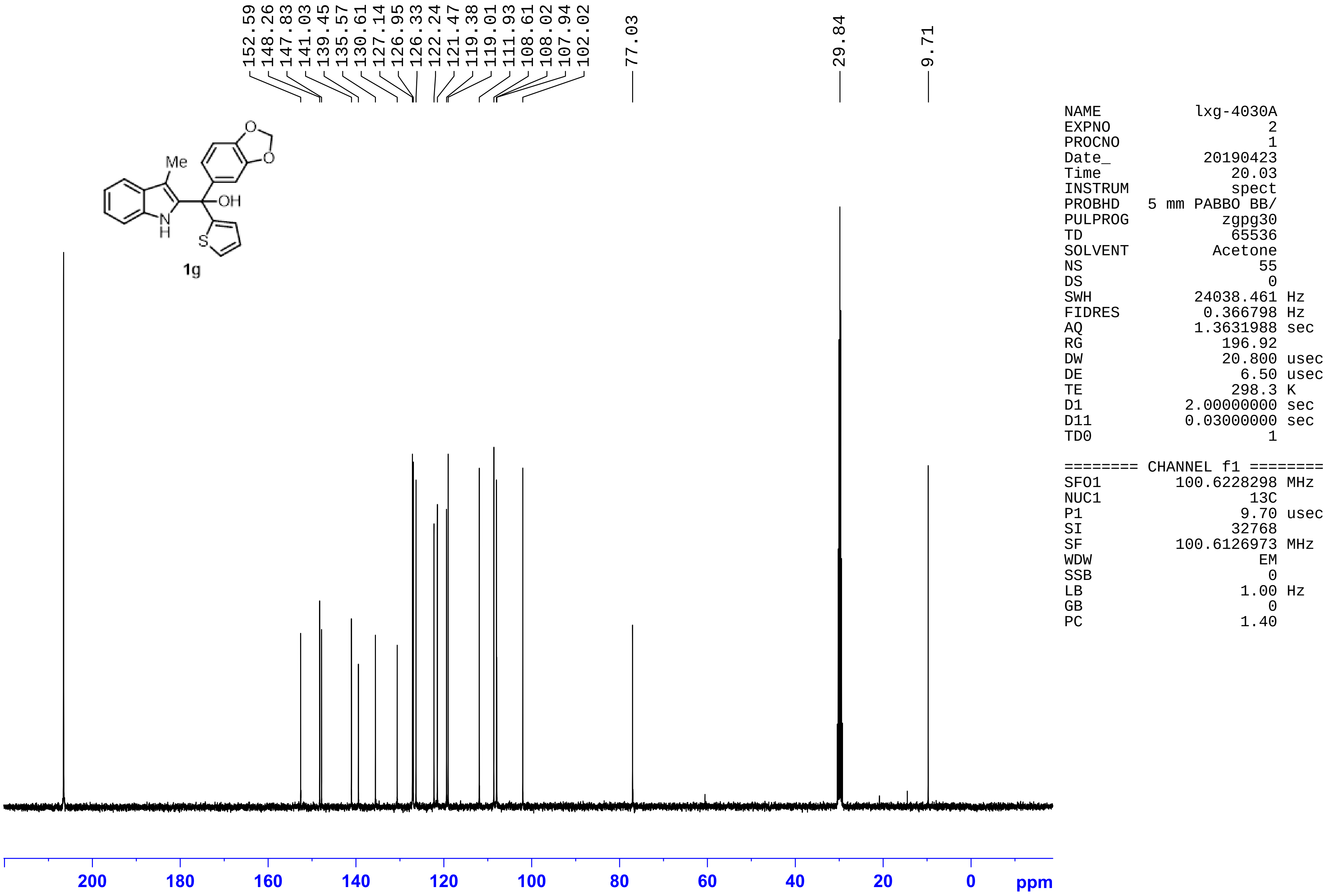

Supplementary Figure 15. ^{13}C NMR spectrum of **1g**

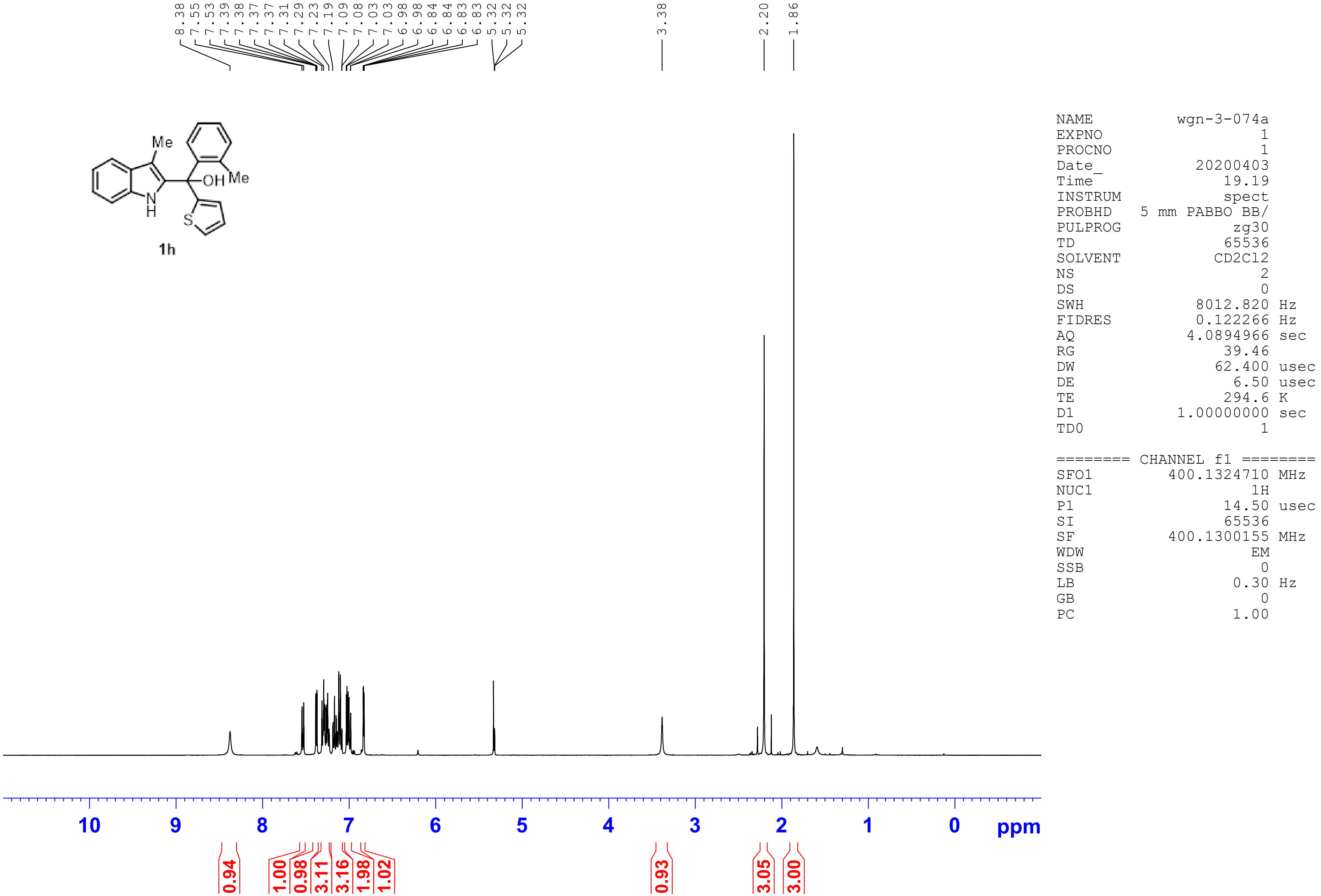

Supplementary Figure 16. ^{1}H NMR spectrum of **1h**

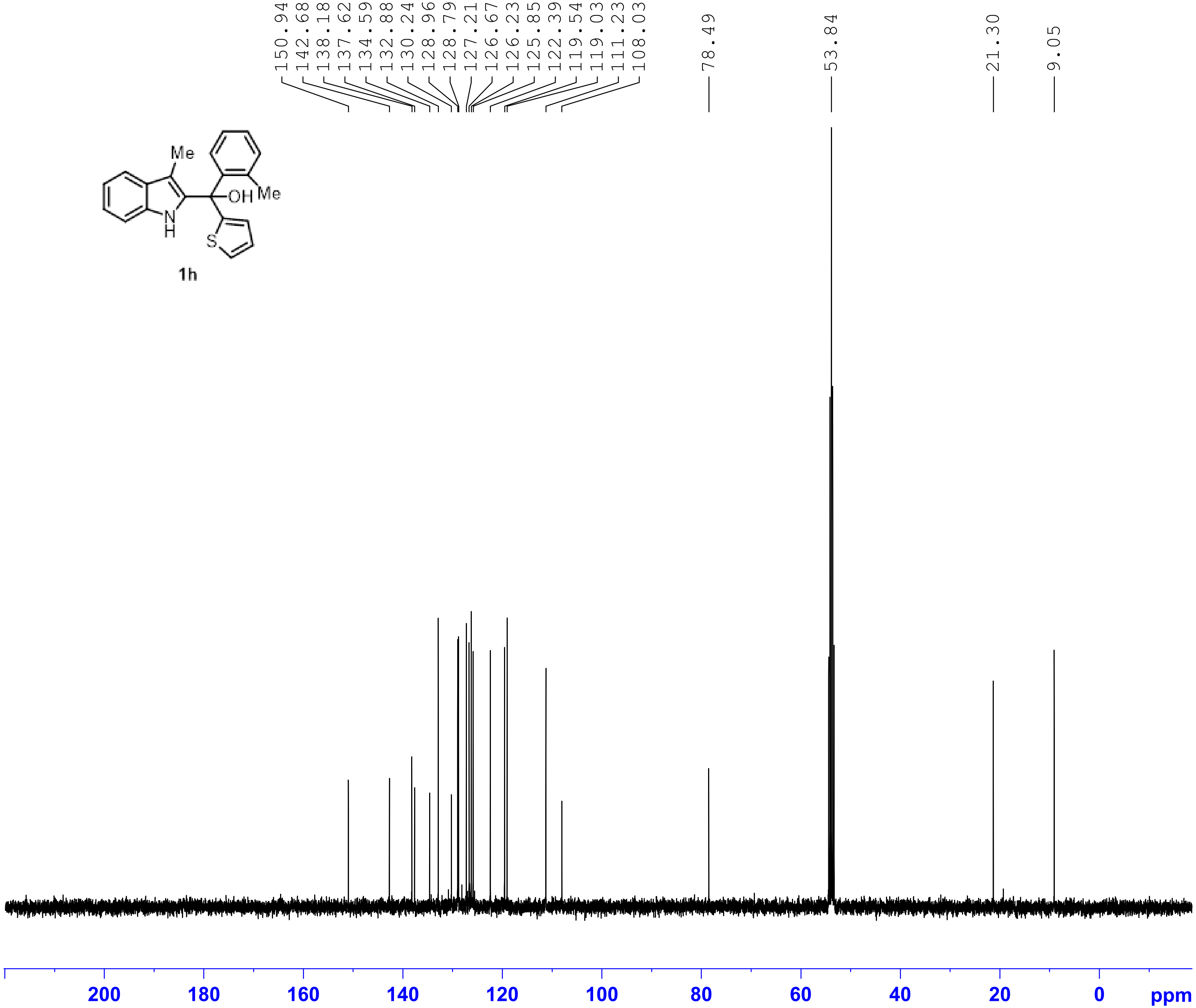

NAME	wgn-3-074a	
EXPNO	3	
PROCNO	1	
Date_	20200403	
Time	19.23	
INSTRUM	spect	
PROBHD	5 mm PABBO BB/	
PULPROG	zgpg30	
TD	65536	
SOLVENT	CD2Cl2	
NS	55	
DS	0	
SWH	24038.461	Hz
FIDRES	0.366798	Hz
AQ	1.3631988	sec
RG	196.92	
DW	20.800	usec
DE	6.50	usec
TE	295.0	K
D1	2.00000000	sec
D11	0.03000000	sec
TD0	1	
========	CHANNEL f1	========
SFO1	100.6228298	MHz
NUC1	13C	
P1	9.70	usec
SI	32768	
SF	100.6127314	MHz
WDW	EM	
SSB	0	
LB	1.00	Hz
GB	0	
PC	1.40	

Supplementary Figure 17. ^{13}C NMR spectrum of **1h**

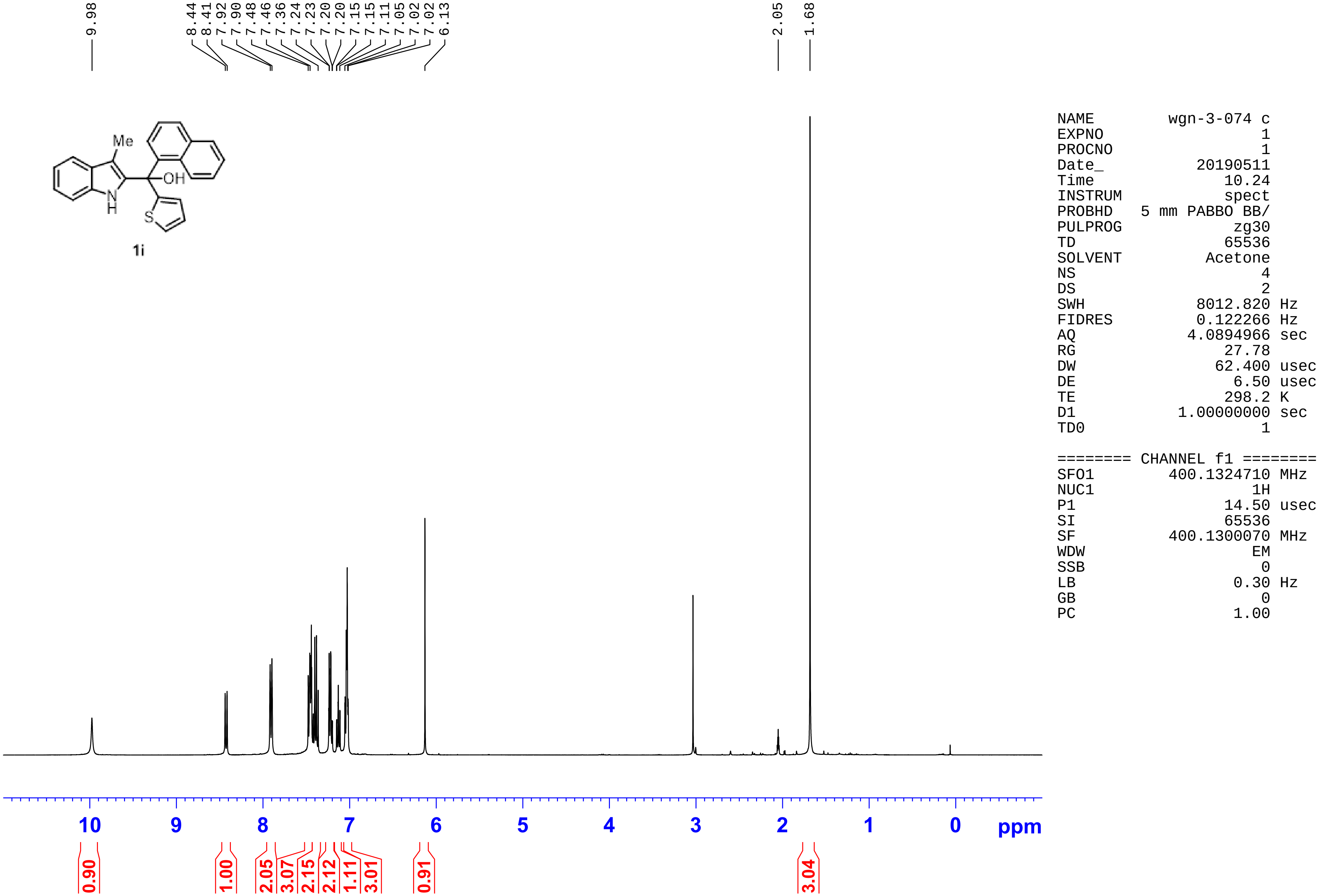

Supplementary Figure 18. ^{1}H NMR spectrum of **1i**

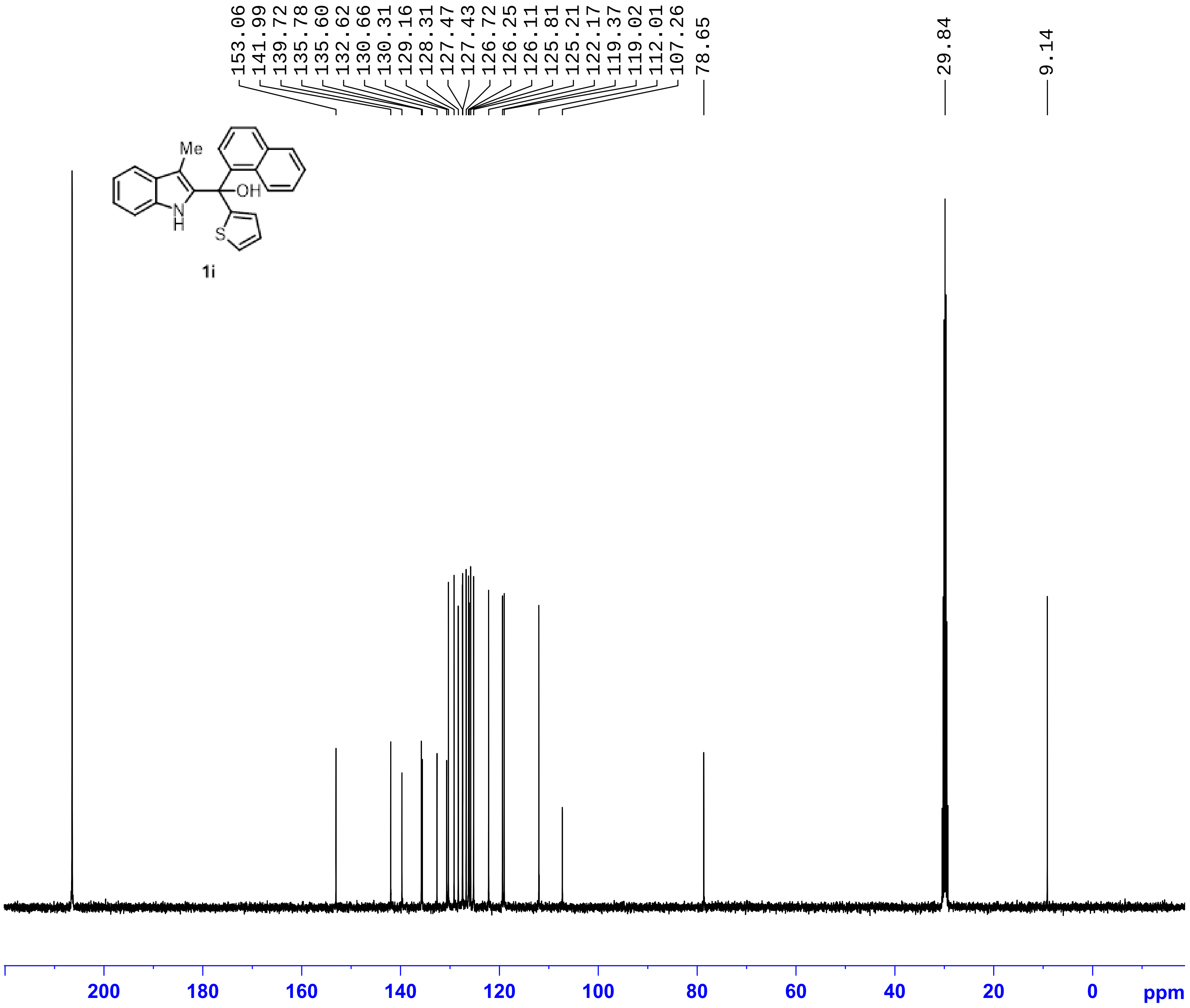

NAME	wgn-3-074 c-C	
EXPNO	1	
PROCNO	1	
Date_	20190511	
Time	10.27	
INSTRUM	spect	
PROBHD	5 mm PABBO BB/	
PULPROG	zgpg30	
TD	65536	
SOLVENT	Acetone	
NS	56	
DS	2	
SWH	24038.461	Hz
FIDRES	0.366798	Hz
AQ	1.3631988	sec
RG	196.92	
DW	20.800	usec
DE	6.50	usec
TE	299.0	K
D1	2.00000000	sec
D11	0.03000000	sec
TD0	1	

======== CHANNEL f1 ========

SFO1	100.6228298	MHz
NUC1	13C	
P1	9.70	usec
SI	32768	
SF	100.6126952	MHz
WDW	EM	
SSB	0	
LB	1.00	Hz
GB	0	
PC	1.40	

Supplementary Figure 19. ^{13}C NMR spectrum of **1i**

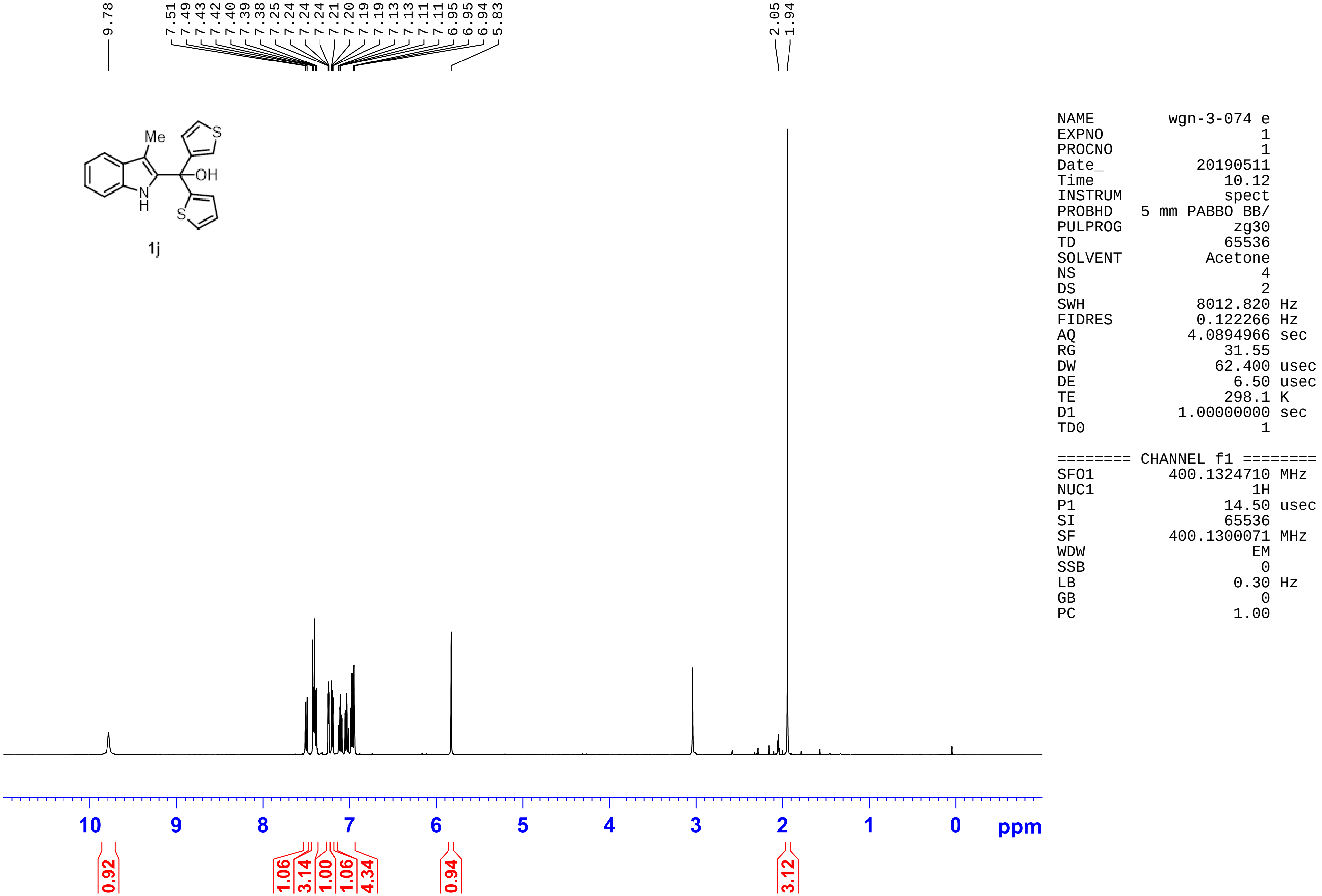

Supplementary Figure 20. ^{1}H NMR spectrum of **1j**

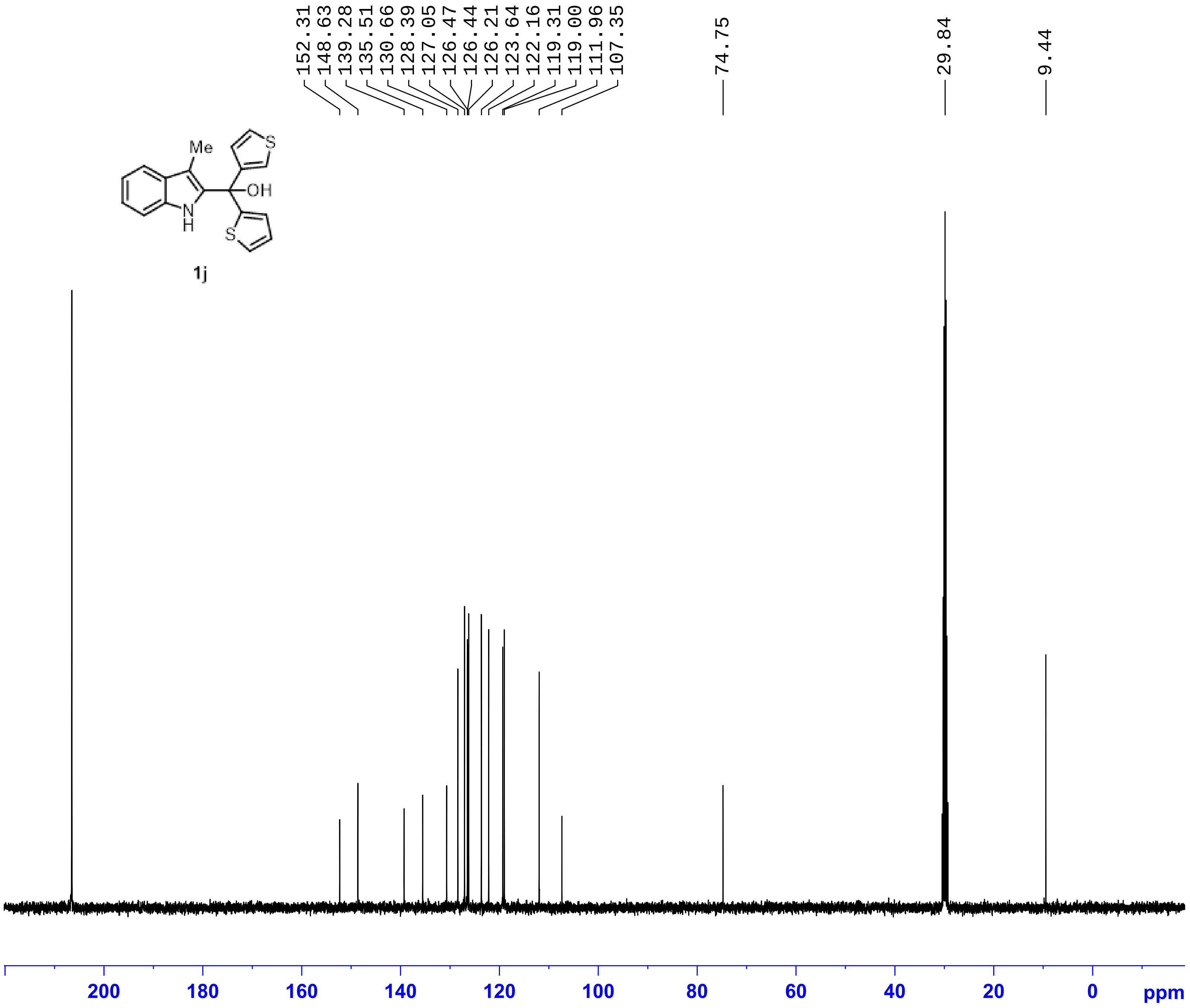

NAME	wgn-3-074 e-C
EXPNO	1
PROCNO	1
Date_	20190511
Time	10.14
INSTRUM	spect
PROBHD	5 mm PABBO BB/
PULPROG	zgpg30
TD	65536
SOLVENT	Acetone
NS	24
DS	2
SWH	24038.461 Hz
FIDRES	0.366798 Hz
AQ	1.3631988 sec
RG	196.92
DW	20.800 usec
DE	6.50 usec
TE	298.7 K
D1	2.00000000 sec
D11	0.03000000 sec
TD0	1

======== CHANNEL f1 ========

SFO1	100.6228298 MHz
NUC1	13C
P1	9.70 usec
SI	32768
SF	100.6126951 MHz
WDW	EM
SSB	0
LB	1.00 Hz
GB	0
PC	1.40

Supplementary Figure 21. ^{13}C NMR spectrum of **1j**

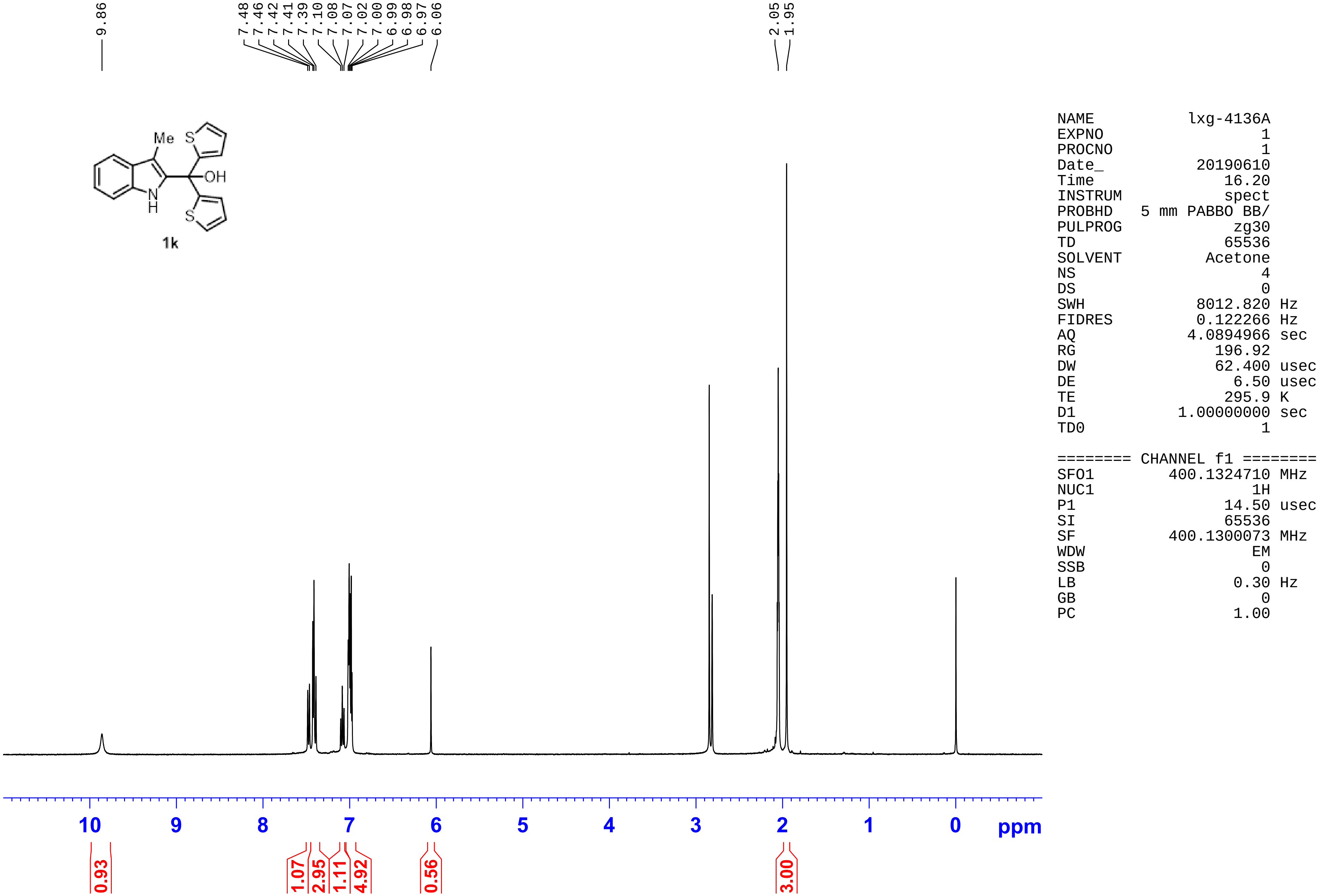

Supplementary Figure 22. ^{1}H NMR spectrum of **1k**

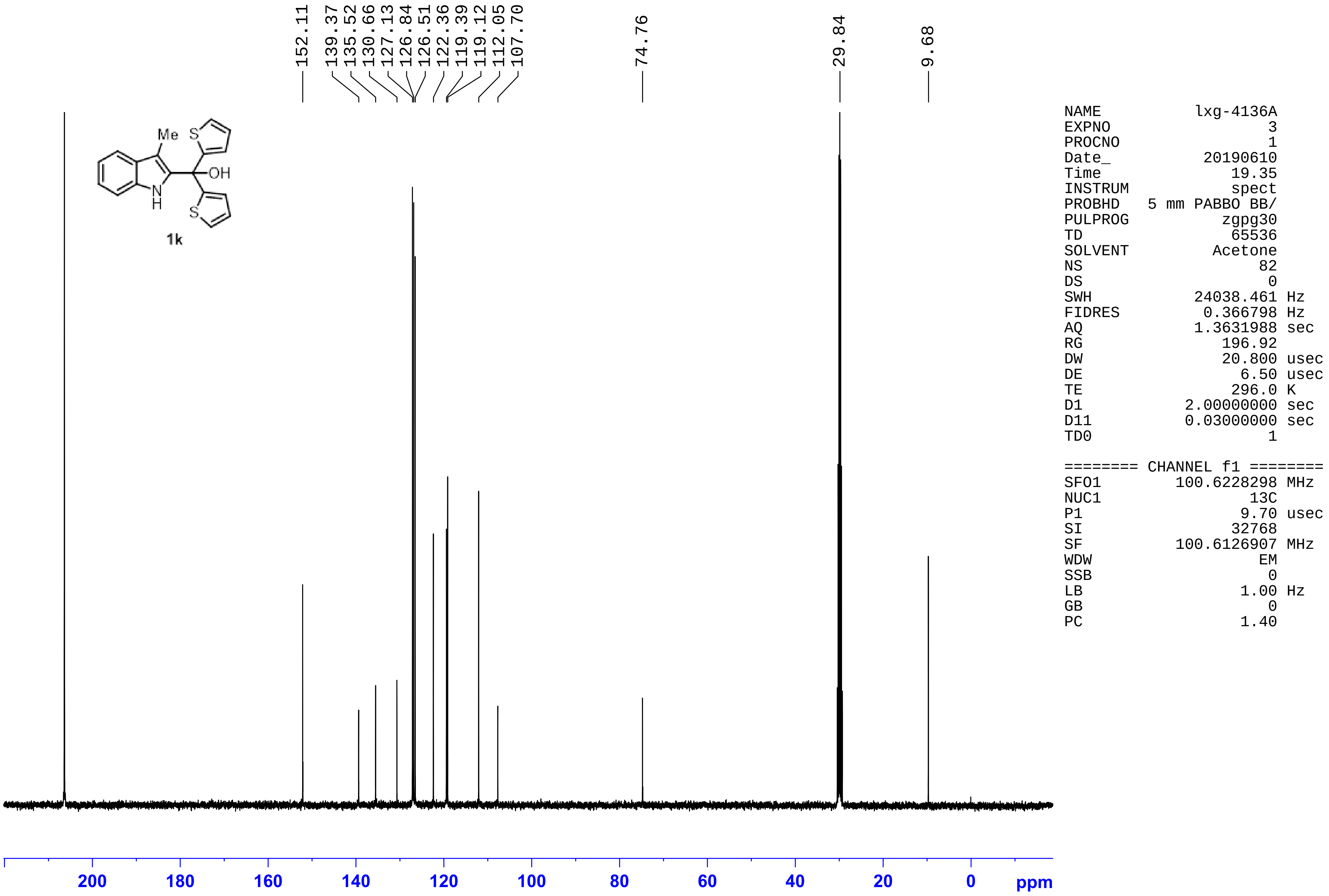

Supplementary Figure 23. ^{13}C NMR spectrum of **1k**

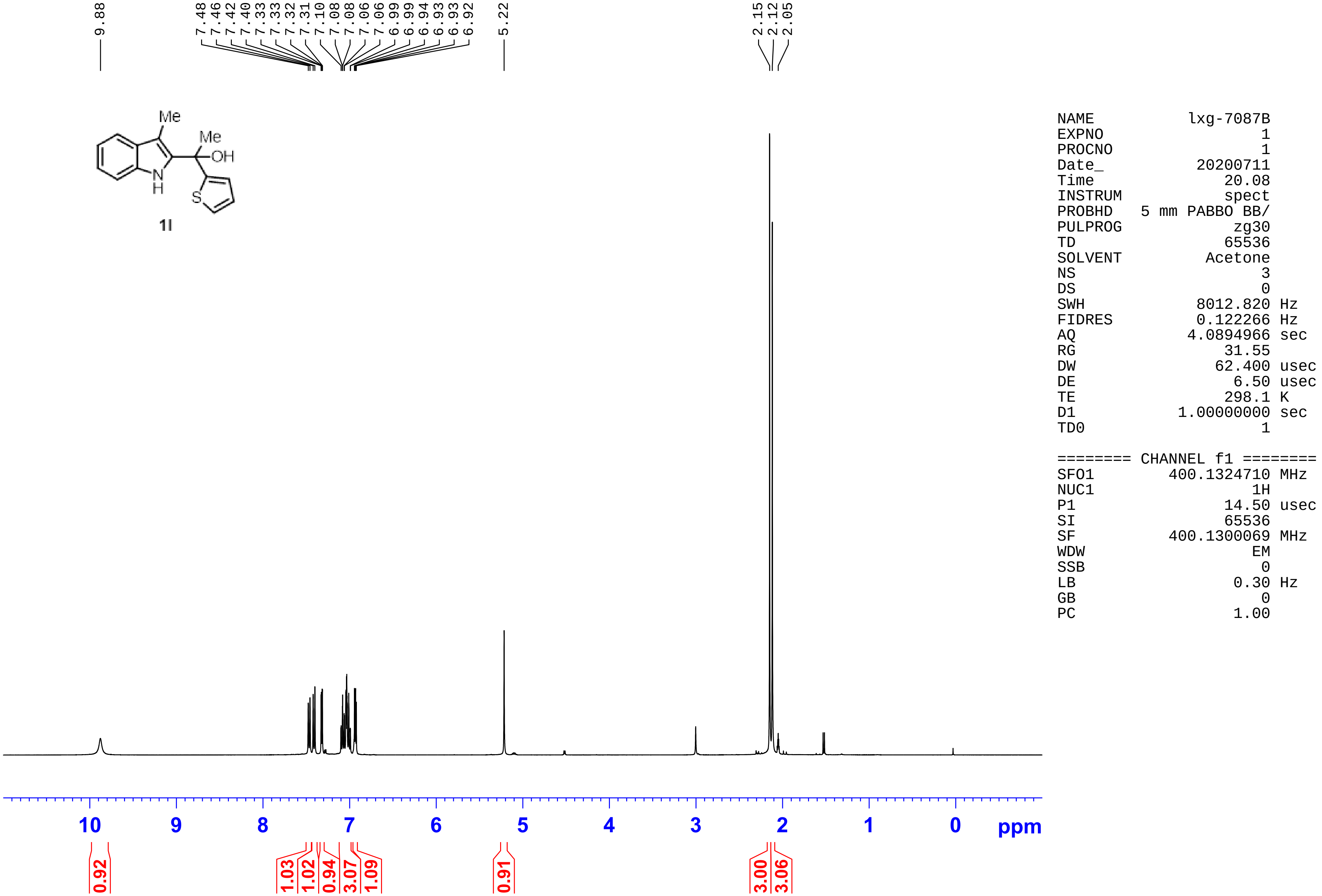

Supplementary Figure 24. ^{1}H NMR spectrum of **1l**

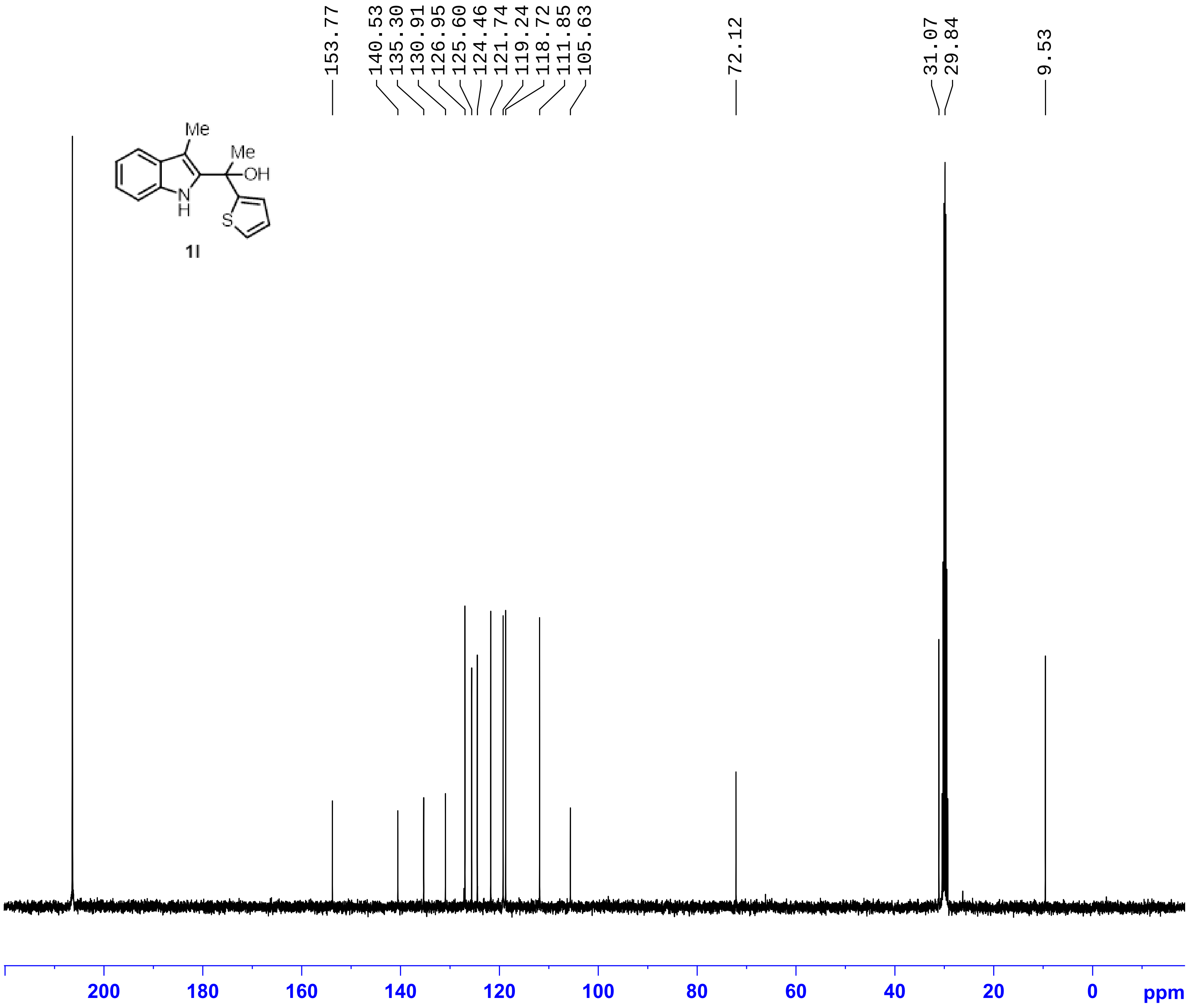

NAME	lxg-7087B
EXPNO	2
PROCNO	1
Date_	20200711
Time	20.10
INSTRUM	spect
PROBHD	5 mm PABBO BB/
PULPROG	zgpg30
TD	65536
SOLVENT	Acetone
NS	38
DS	0
SWH	24038.461 Hz
FIDRES	0.366798 Hz
AQ	1.3631988 sec
RG	196.92
DW	20.800 usec
DE	6.50 usec
TE	298.8 K
D1	2.00000000 sec
D11	0.03000000 sec
TD0	1

======== CHANNEL f1 ========

SFO1	100.6228298 MHz
NUC1	13C
P1	9.70 usec
SI	32768
SF	100.6126878 MHz
WDW	EM
SSB	0
LB	1.00 Hz
GB	0
PC	1.40

Supplementary Figure 25. ^{13}C NMR spectrum of **1l**

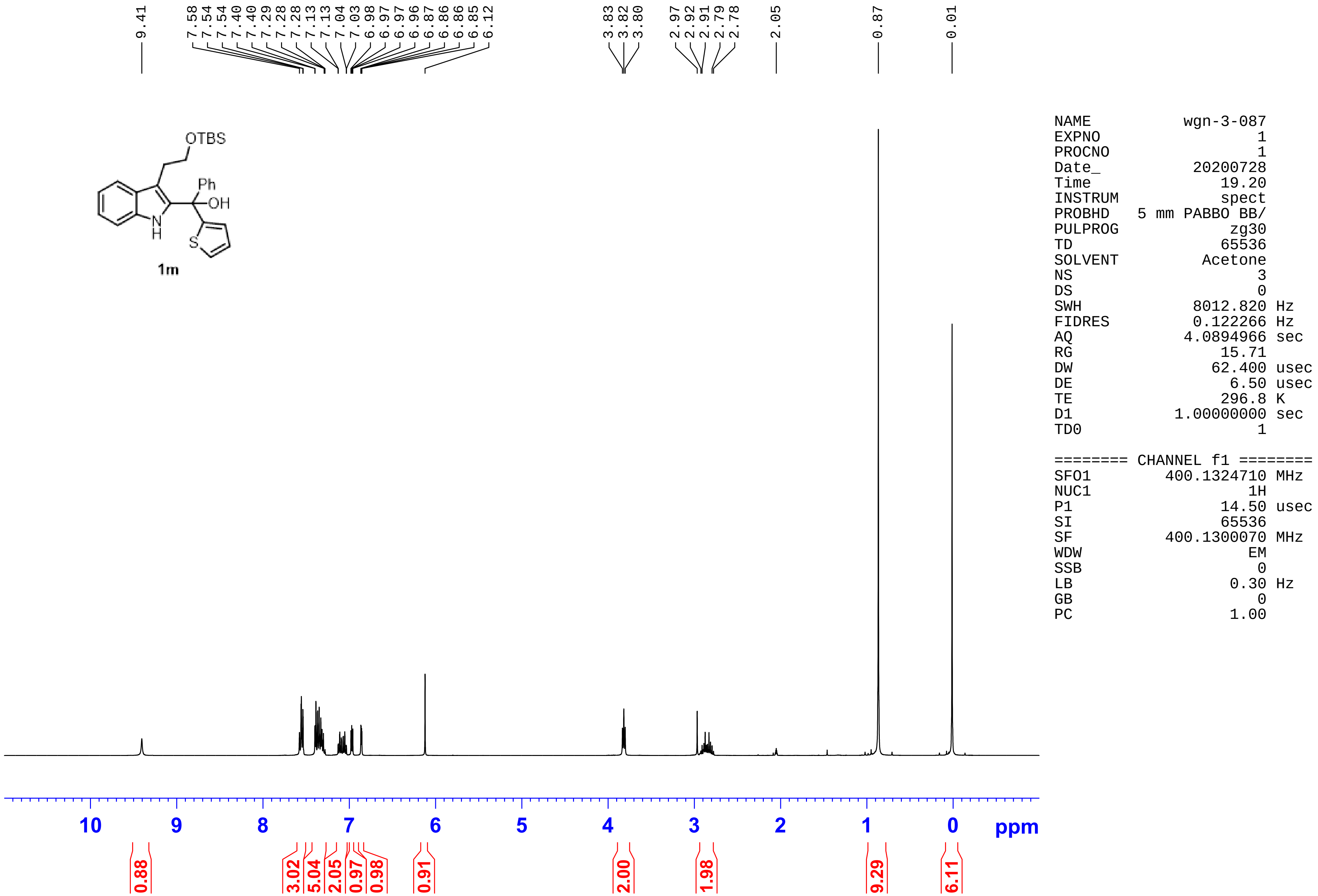

Supplementary Figure 26. ^{1}H NMR spectrum of **1m**

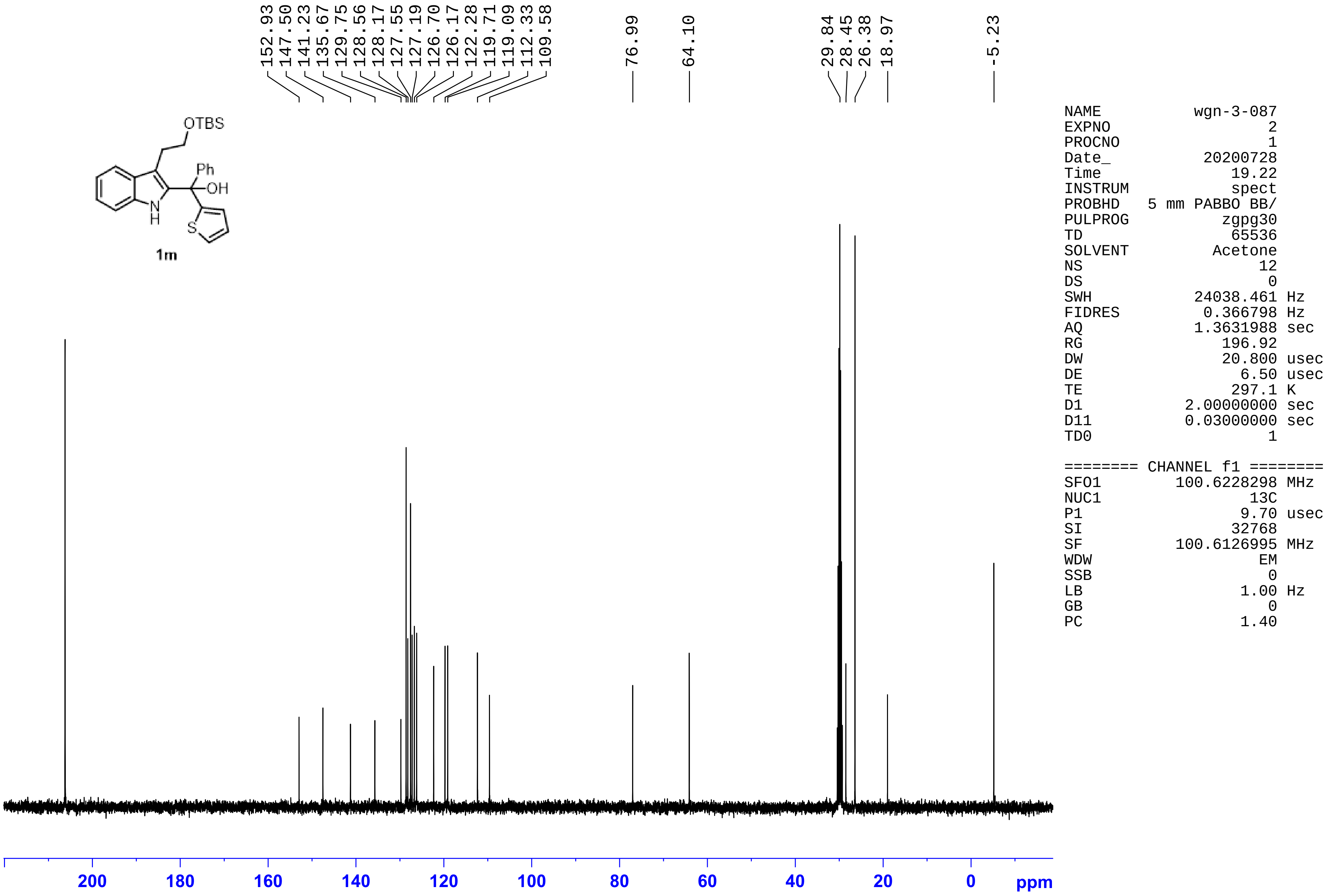

1m

Supplementary Figure 27. ^{13}C NMR spectrum of **1m**

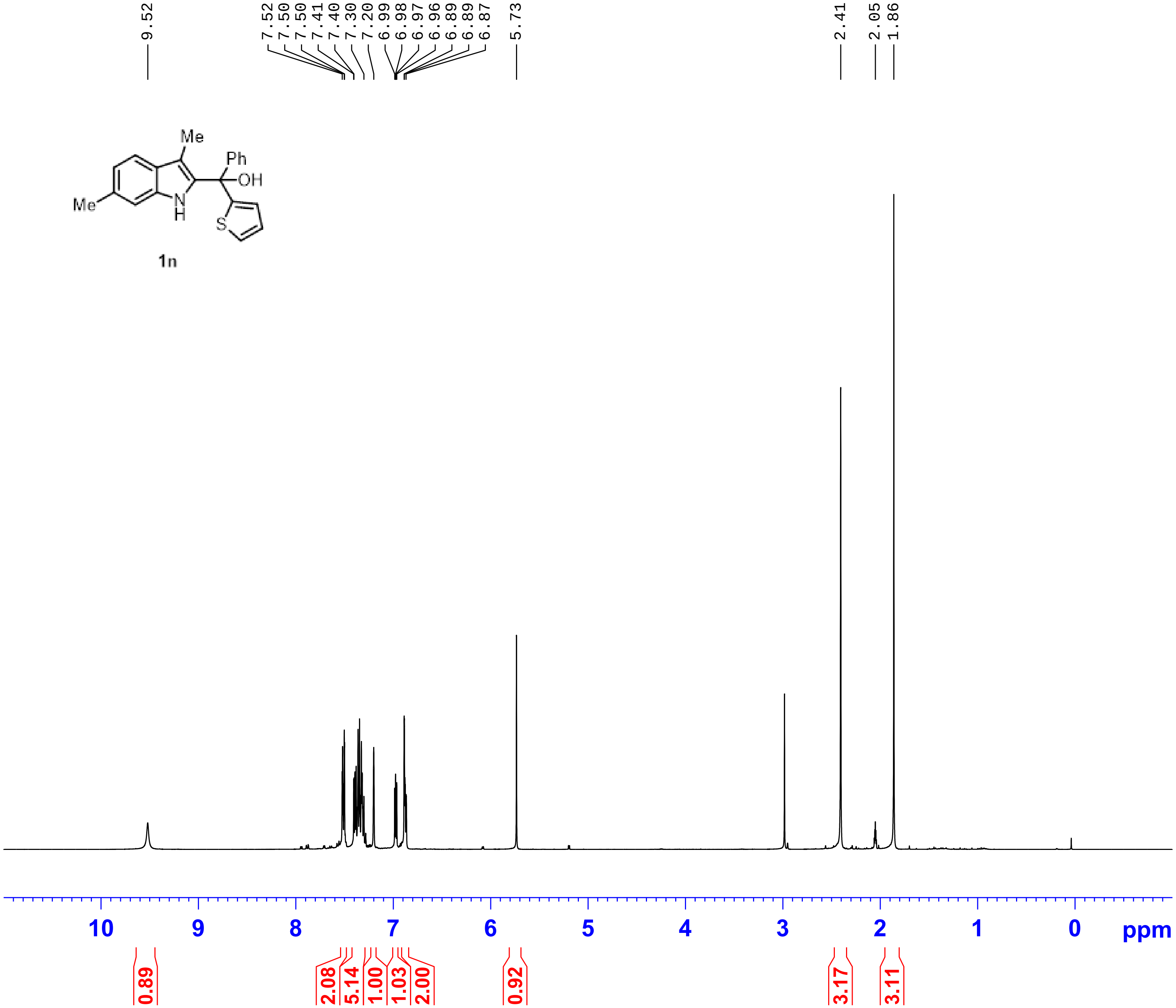

NAME	wgn-3-078
EXPNO	1
PROCNO	1
Date_	20190511
Time	10.42
INSTRUM	spect
PROBHD	5 mm PABBO BB/
PULPROG	zg30
TD	65536
SOLVENT	Acetone
NS	4
DS	2
SWH	8012.820 Hz
FIDRES	0.122266 Hz
AQ	4.0894966 sec
RG	31.55
DW	62.400 usec
DE	6.50 usec
TE	298.2 K
D1	1.00000000 sec
TD0	1
======== CHANNEL f1 ========	
SFO1	400.1324710 MHz
NUC1	1H
P1	14.50 usec
SI	65536
SF	400.1300070 MHz
WDW	EM
SSB	0
LB	0.30 Hz
GB	0
PC	1.00

Supplementary Figure 28. ^{1}H NMR spectrum of **1n**

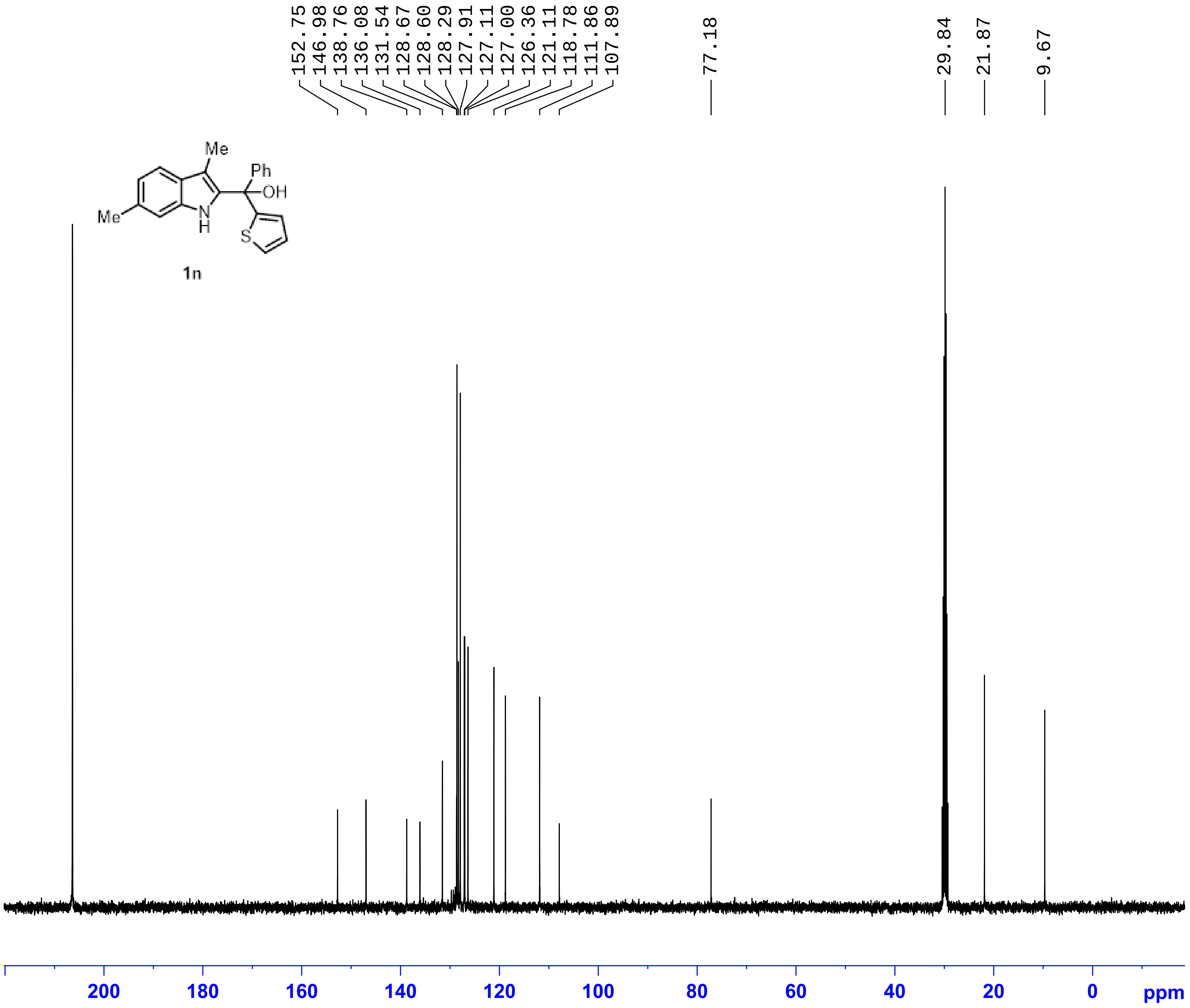

NAME	wgn-3-078-C
EXPNO	2
PROCNO	1
Date_	20190511
Time	10.45
INSTRUM	spect
PROBHD	5 mm PABBO BB/
PULPROG	zgpg30
TD	65536
SOLVENT	Acetone
NS	28
DS	2
SWH	24038.461 Hz
FIDRES	0.366798 Hz
AQ	1.3631988 sec
RG	196.92
DW	20.800 usec
DE	6.50 usec
TE	298.8 K
D1	2.00000000 sec
D11	0.03000000 sec
TD0	1

======== CHANNEL f1 ========

SFO1	100.6228298 MHz
NUC1	13C
P1	9.70 usec
SI	32768
SF	100.6126907 MHz
WDW	EM
SSB	0
LB	1.00 Hz
GB	0
PC	1.40

Supplementary Figure 29. ^{13}C NMR spectrum of **1n**

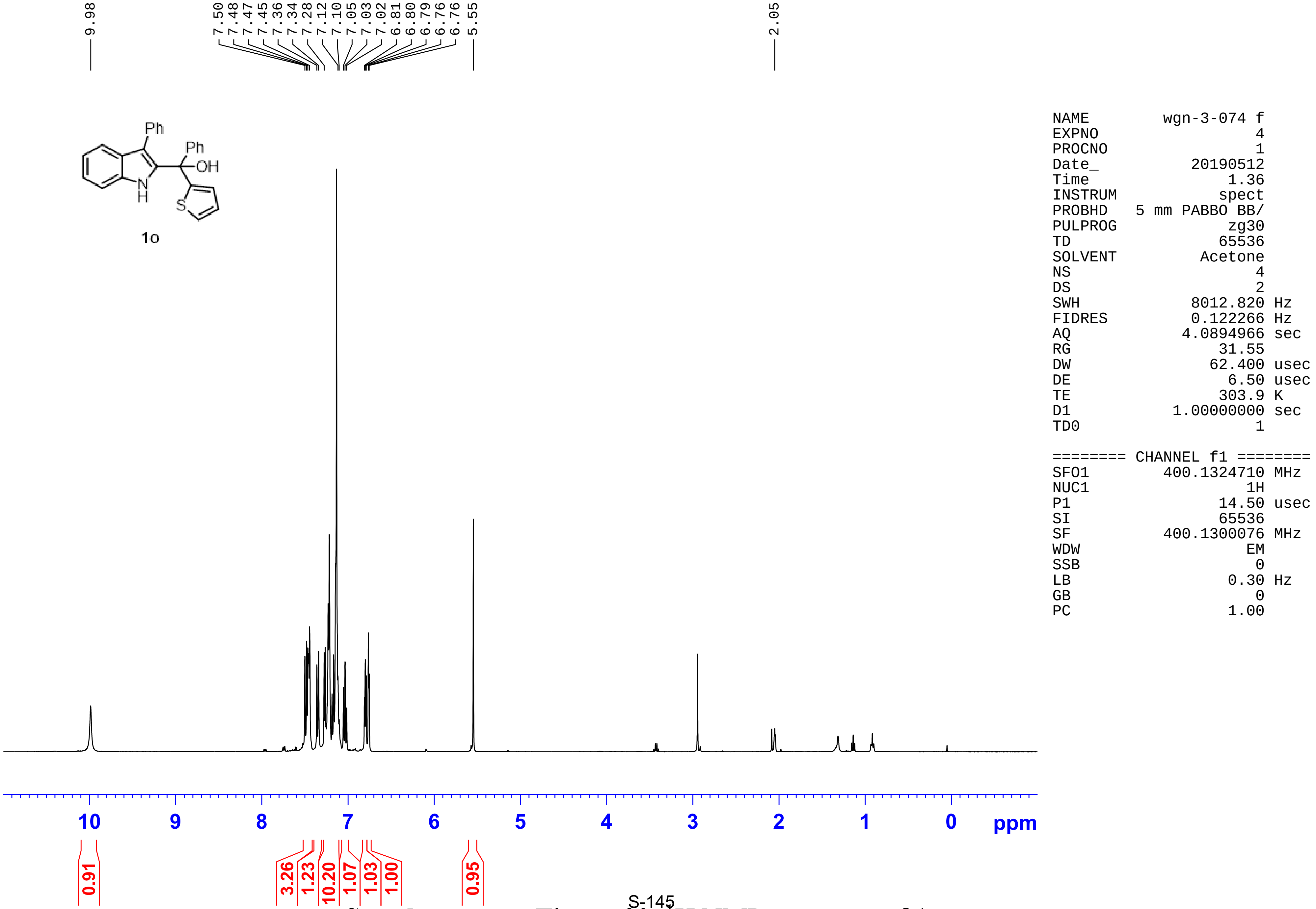

Supplementary Figure 30. ^{1}H NMR spectrum of **1o**

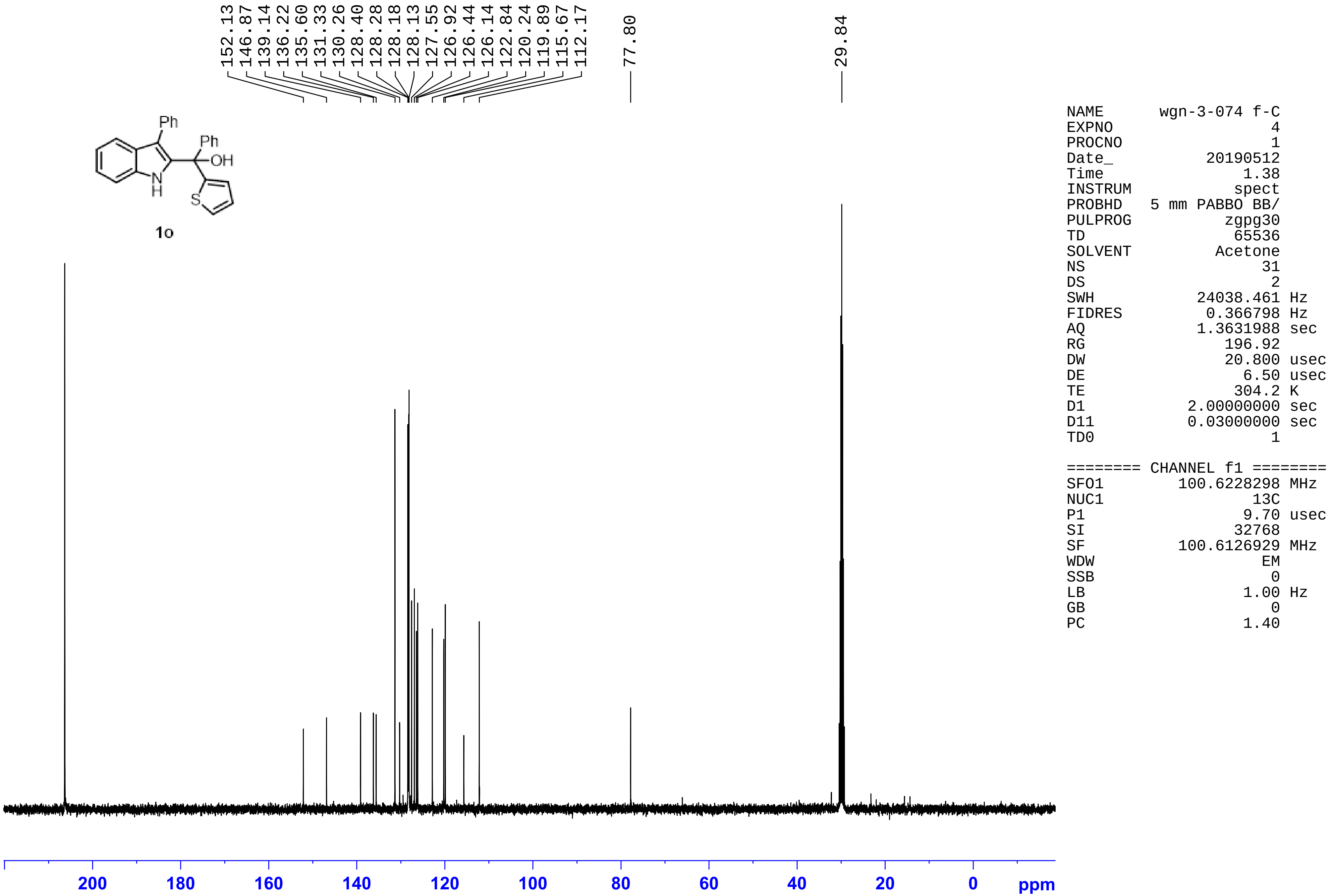

Supplementary Figure 31. ^{13}C NMR spectrum of **1o**

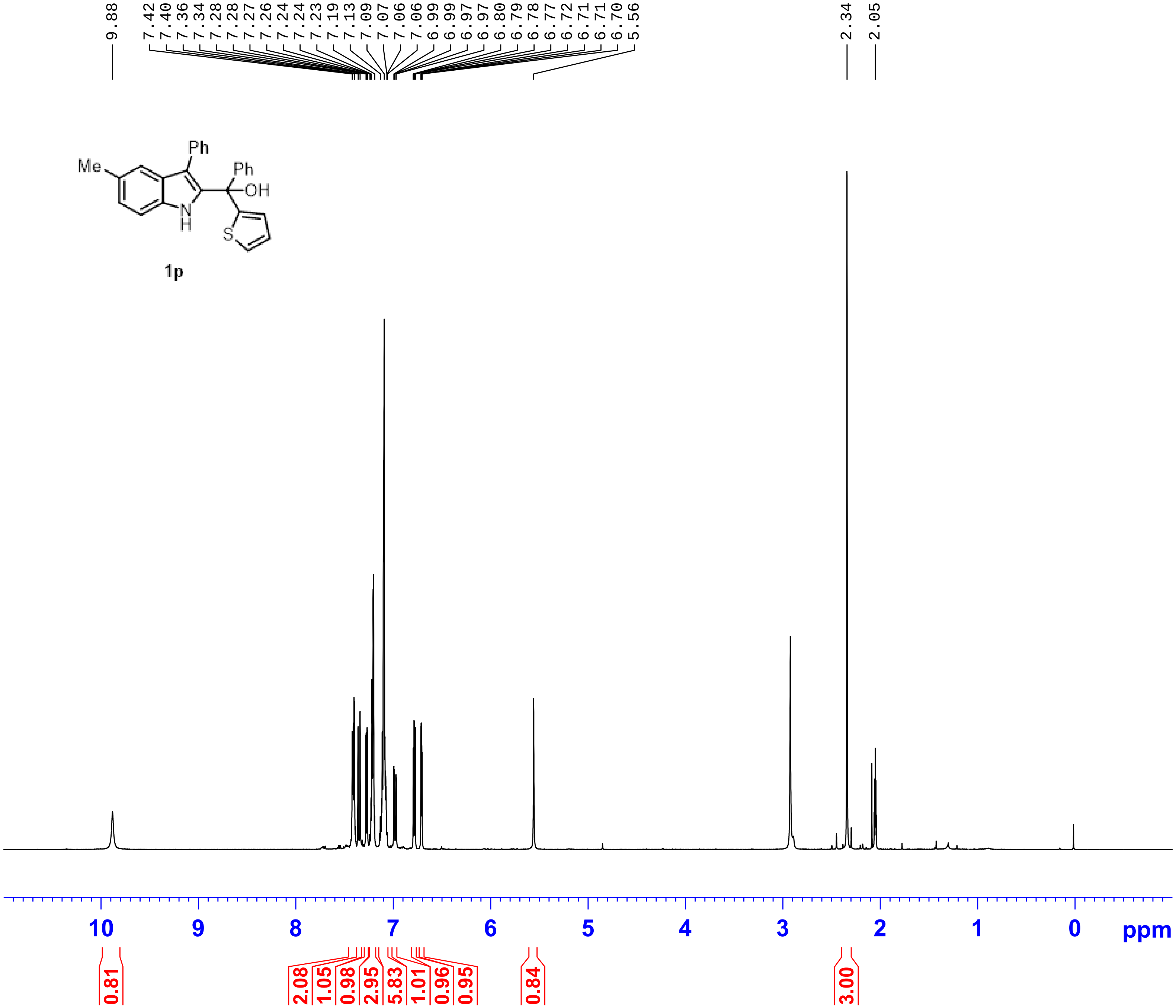

NAME	wgn-3-084b
EXPNO	1
PROCNO	1
Date_	20200728
Time	19.25
INSTRUM	spect
PROBHD	5 mm PABBO BB/
PULPROG	zg30
TD	65536
SOLVENT	Acetone
NS	4
DS	0
SWH	8012.820 Hz
FIDRES	0.122266 Hz
AQ	4.0894966 sec
RG	34.77
DW	62.400 usec
DE	6.50 usec
TE	296.8 K
D1	1.00000000 sec
TD0	1

======== CHANNEL f1 ========

SFO1	400.1324710 MHz
NUC1	1H
P1	14.50 usec
SI	65536
SF	400.1300069 MHz
WDW	EM
SSB	0
LB	0.30 Hz
GB	0
PC	1.00

Supplementary Figure 32. ^{1}H NMR spectrum of **1p**

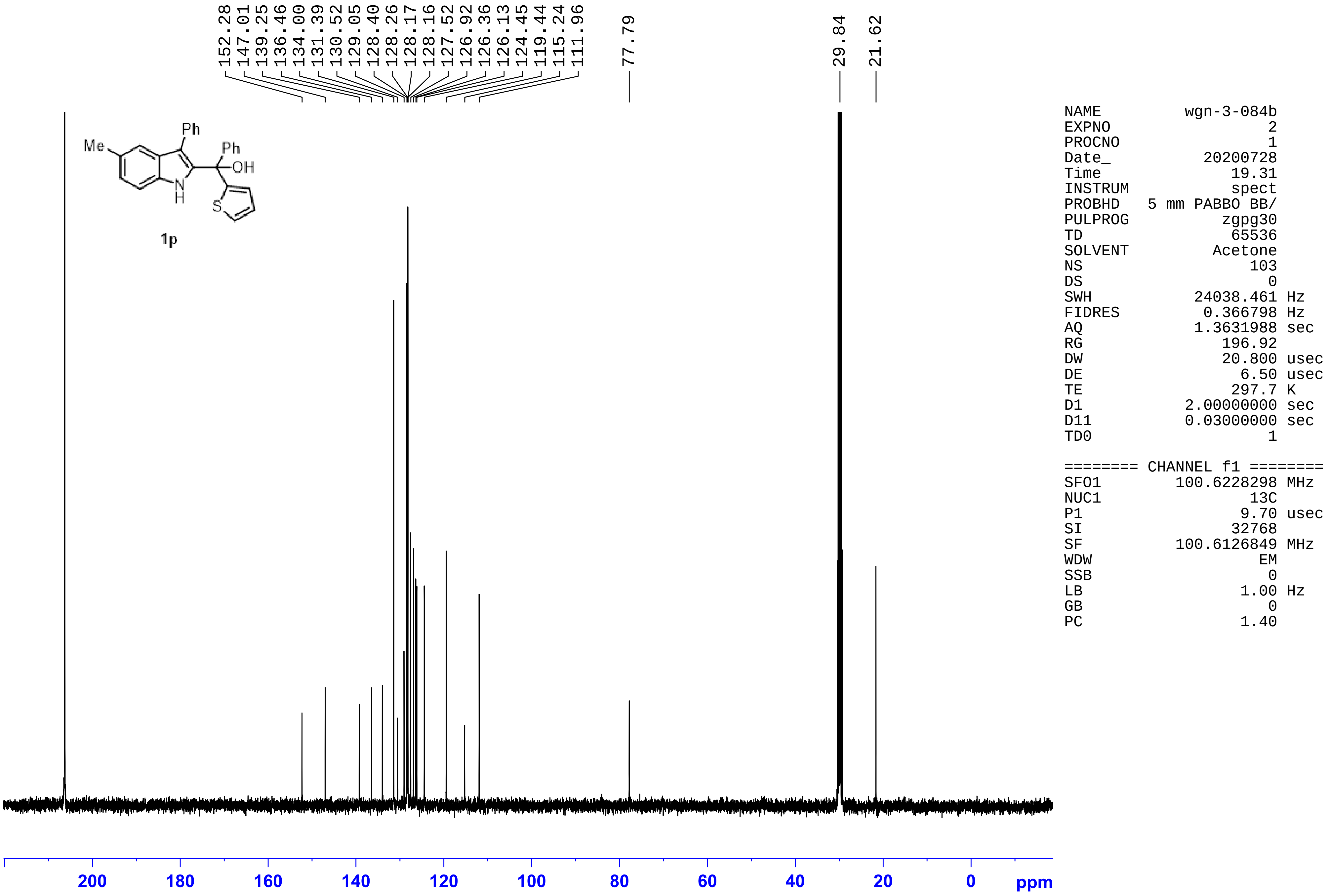

Supplementary Figure 33. ^{13}C NMR spectrum of **1p**

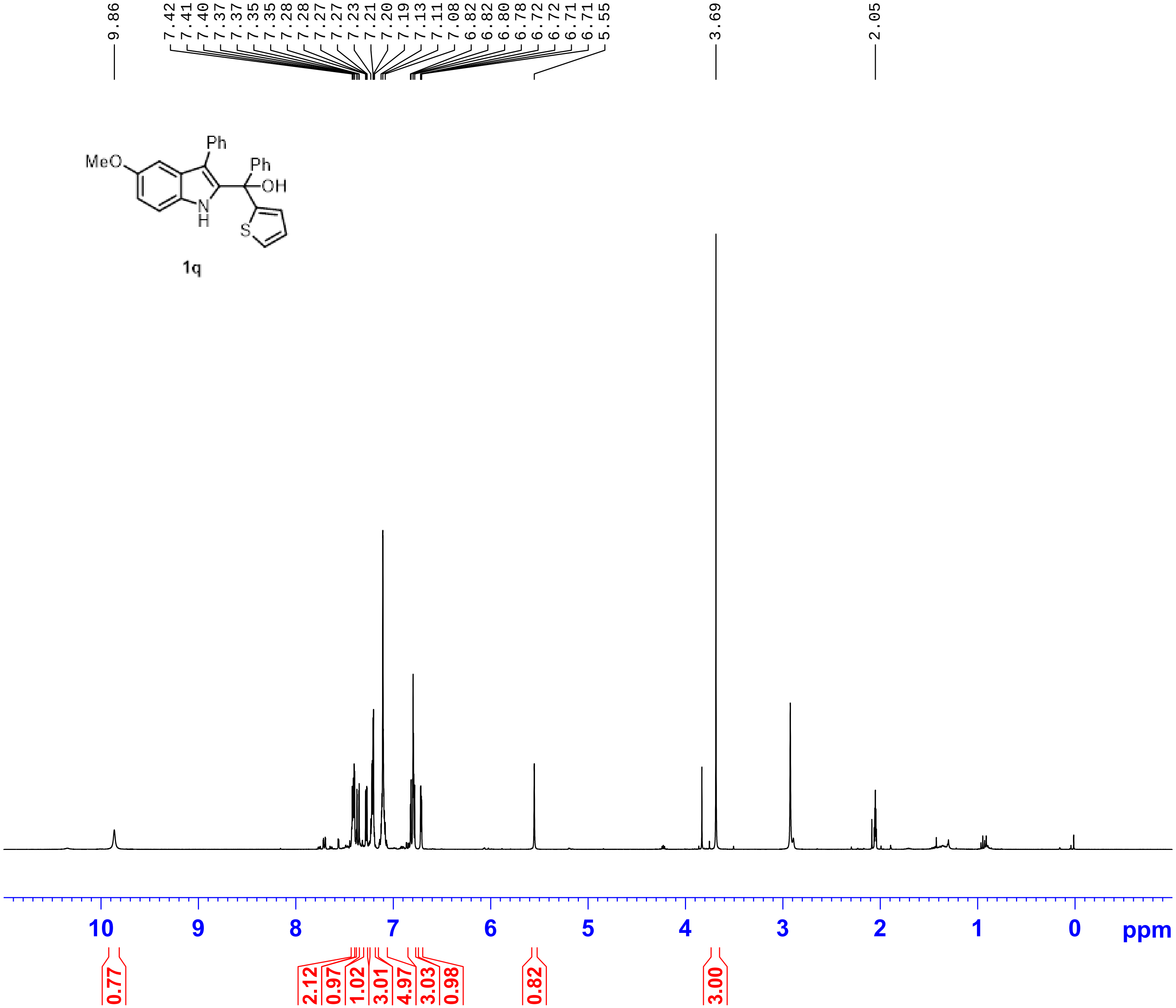

NAME	wgn-3-084c	
EXPNO	1	
PROCNO	1	
Date_	20200728	
Time	19.34	
INSTRUM	spect	
PROBHD	5 mm PABBO BB/	
PULPROG	zg30	
TD	65536	
SOLVENT	Acetone	
NS	4	
DS	0	
SWH	8012.820	Hz
FIDRES	0.122266	Hz
AQ	4.0894966	sec
RG	34.77	
DW	62.400	usec
DE	6.50	usec
TE	297.1	K
D1	1.00000000	sec
TD0	1	

======== CHANNEL f1 ========

SFO1	400.1324710	MHz
NUC1	1H	
P1	14.50	usec
SI	65536	
SF	400.1300069	MHz
WDW	EM	
SSB	0	
LB	0.30	Hz
GB	0	
PC	1.00	

Supplementary Figure 34. ^{1}H NMR spectrum of **1q**

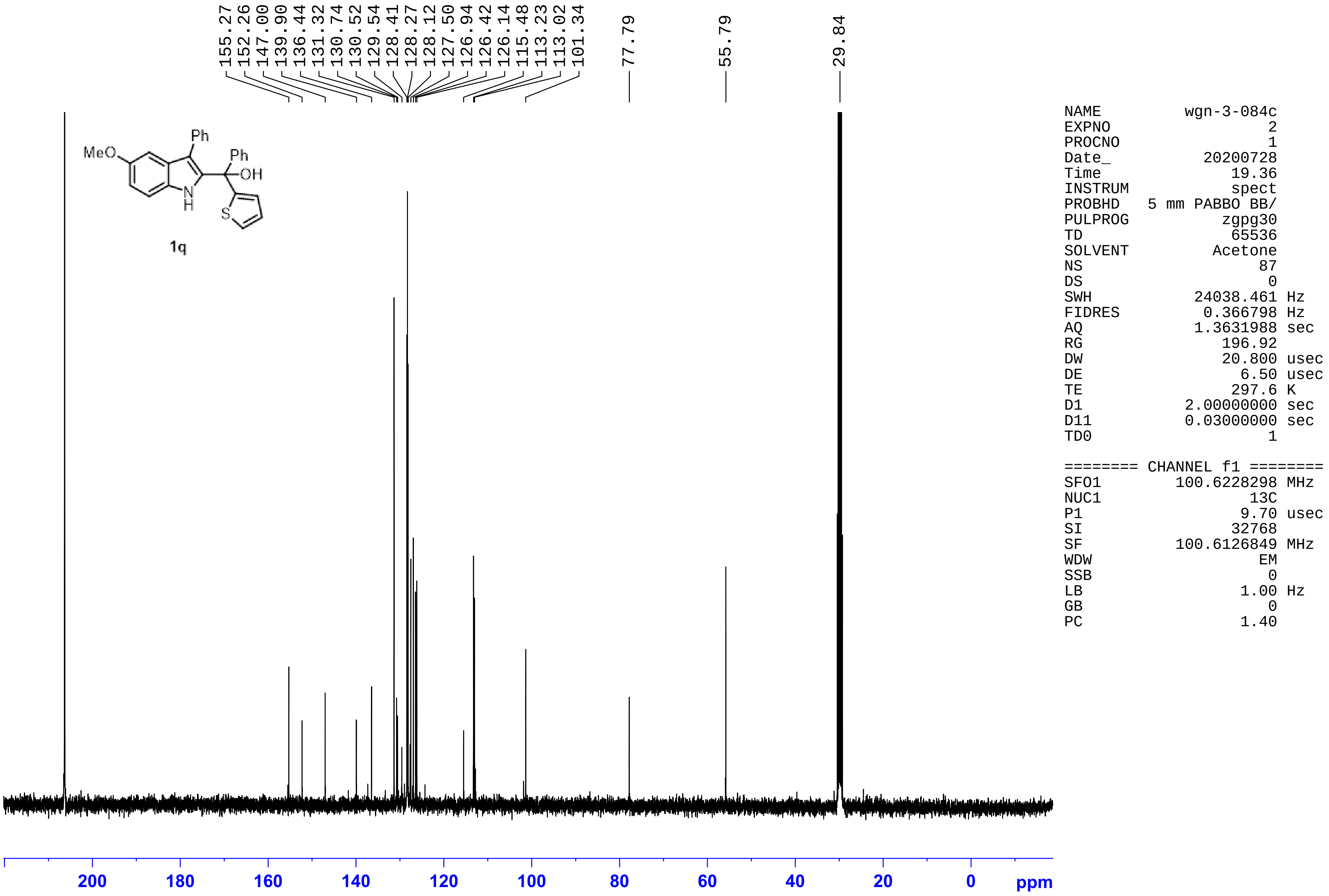

Supplementary Figure 35. ^{13}C NMR spectrum of **1q**

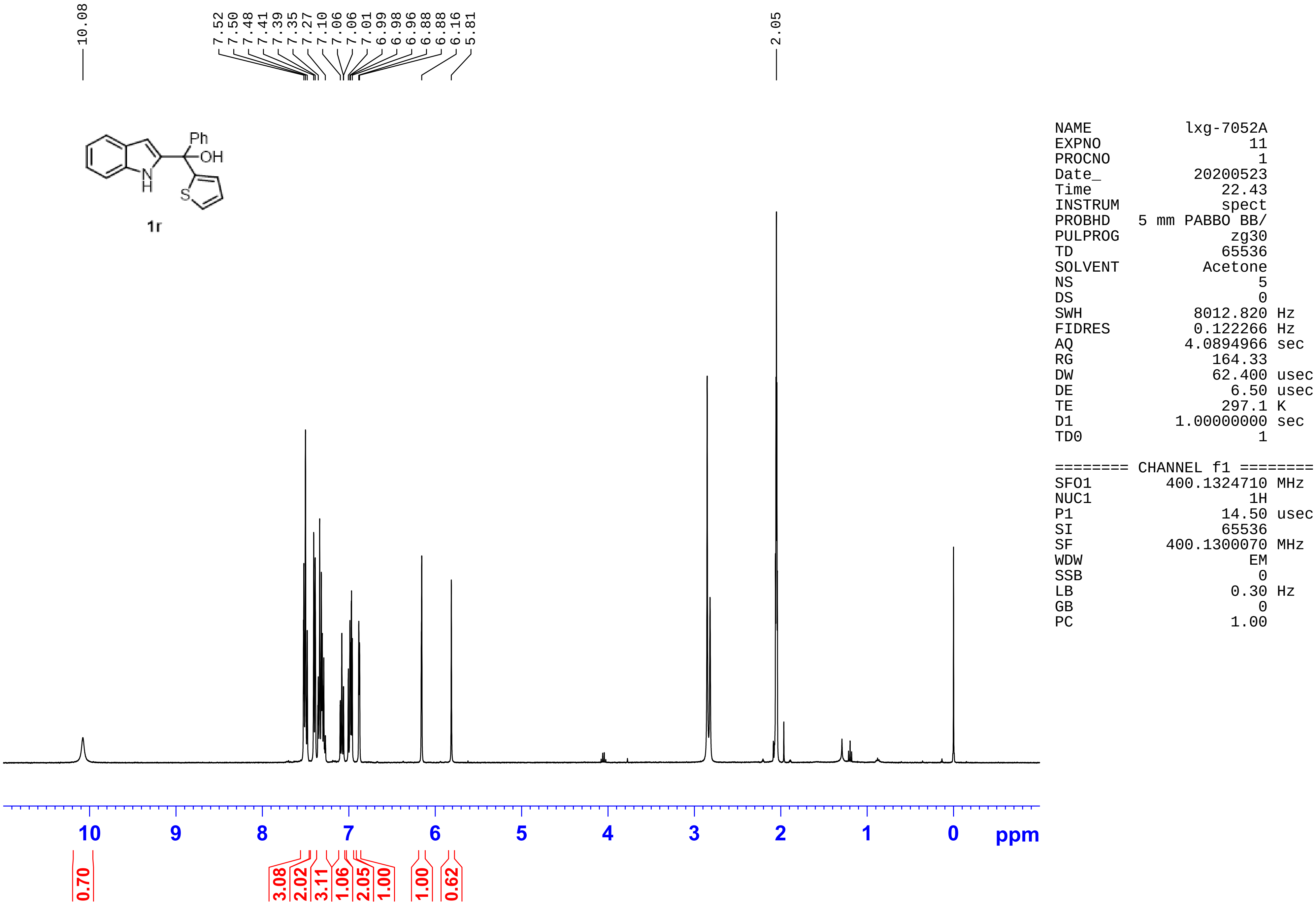

Supplementary Figure 36. ^{1}H NMR spectrum of **1r**

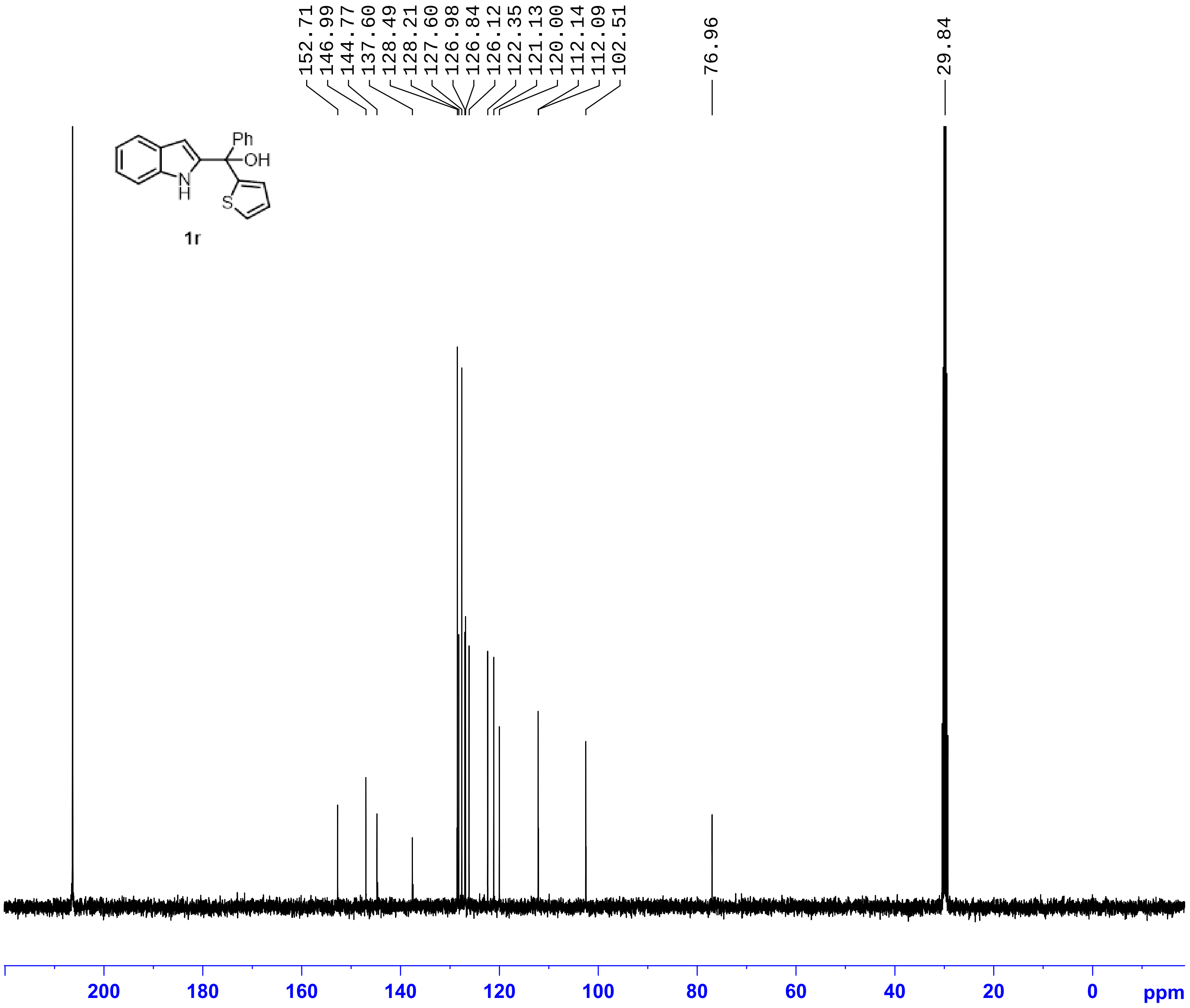

NAME	lxg-7052A	
EXPNO	2	
PROCNO	1	
Date_	20200523	
Time	10.42	
INSTRUM	spect	
PROBHD	5 mm PABBO BB/	
PULPROG	zgpg30	
TD	65536	
SOLVENT	Acetone	
NS	36	
DS	0	
SWH	24038.461	Hz
FIDRES	0.366798	Hz
AQ	1.3631988	sec
RG	196.92	
DW	20.800	usec
DE	6.50	usec
TE	296.4	K
D1	2.00000000	sec
D11	0.03000000	sec
TD0	1	

======== CHANNEL f1 ========

SFO1	100.6228298	MHz
NUC1	13C	
P1	9.70	usec
SI	32768	
SF	100.6126878	MHz
WDW	EM	
SSB	0	
LB	1.00	Hz
GB	0	
PC	1.40	

Supplementary Figure 37. ^{13}C NMR spectrum of **1r**

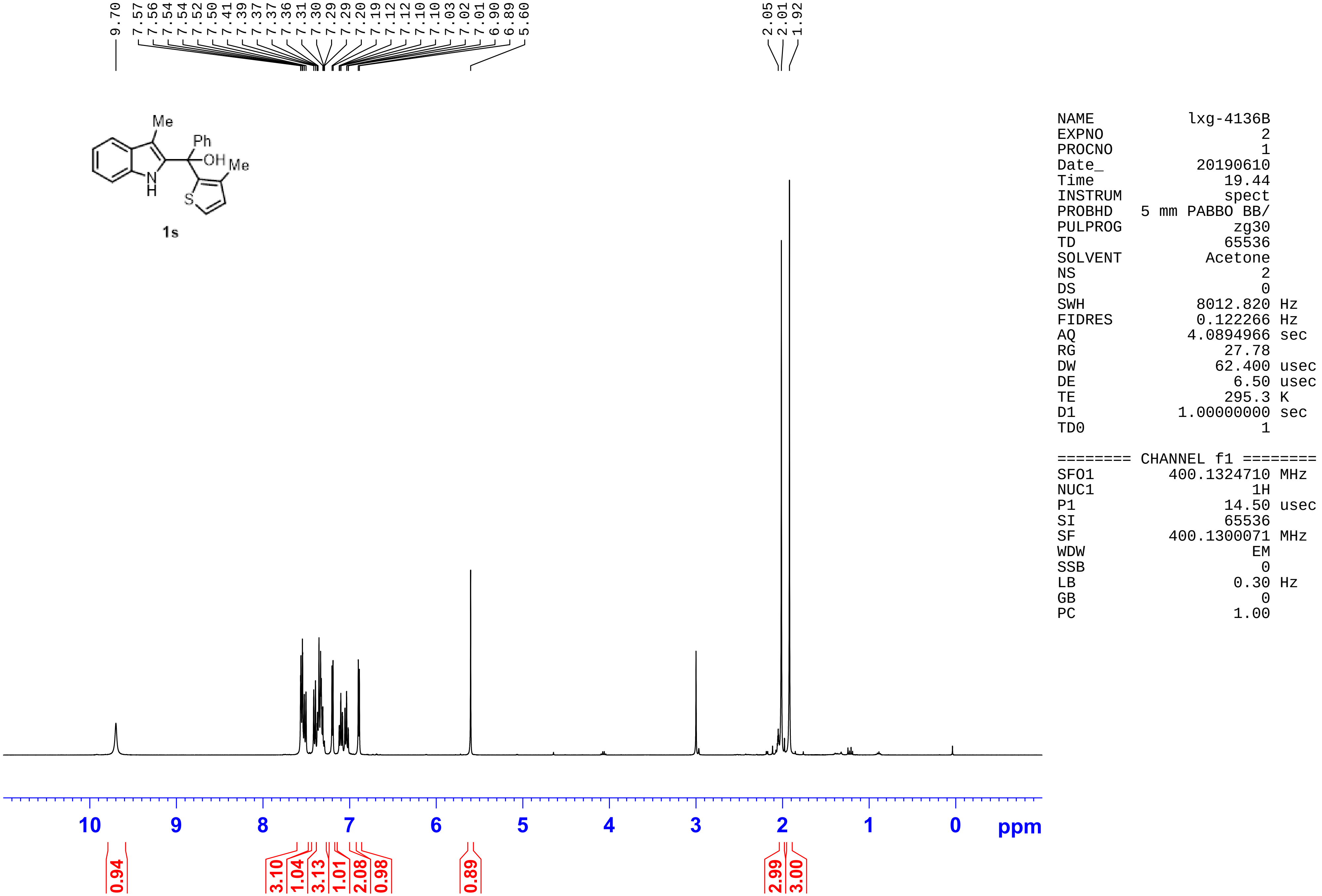

Supplementary Figure 38. ^{1}H NMR spectrum of **1s**

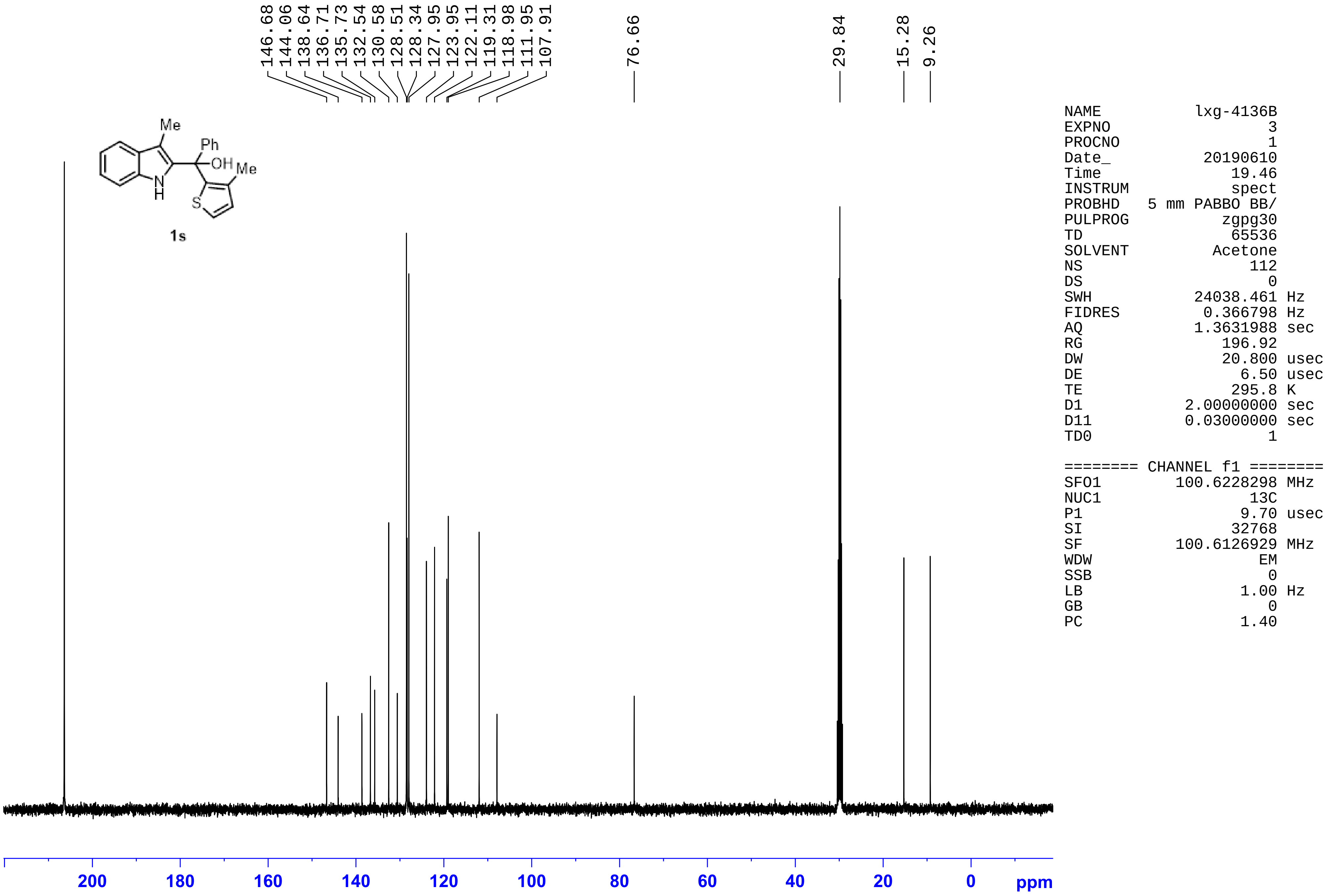

Supplementary Figure 39. ^{13}C NMR spectrum of **1s**

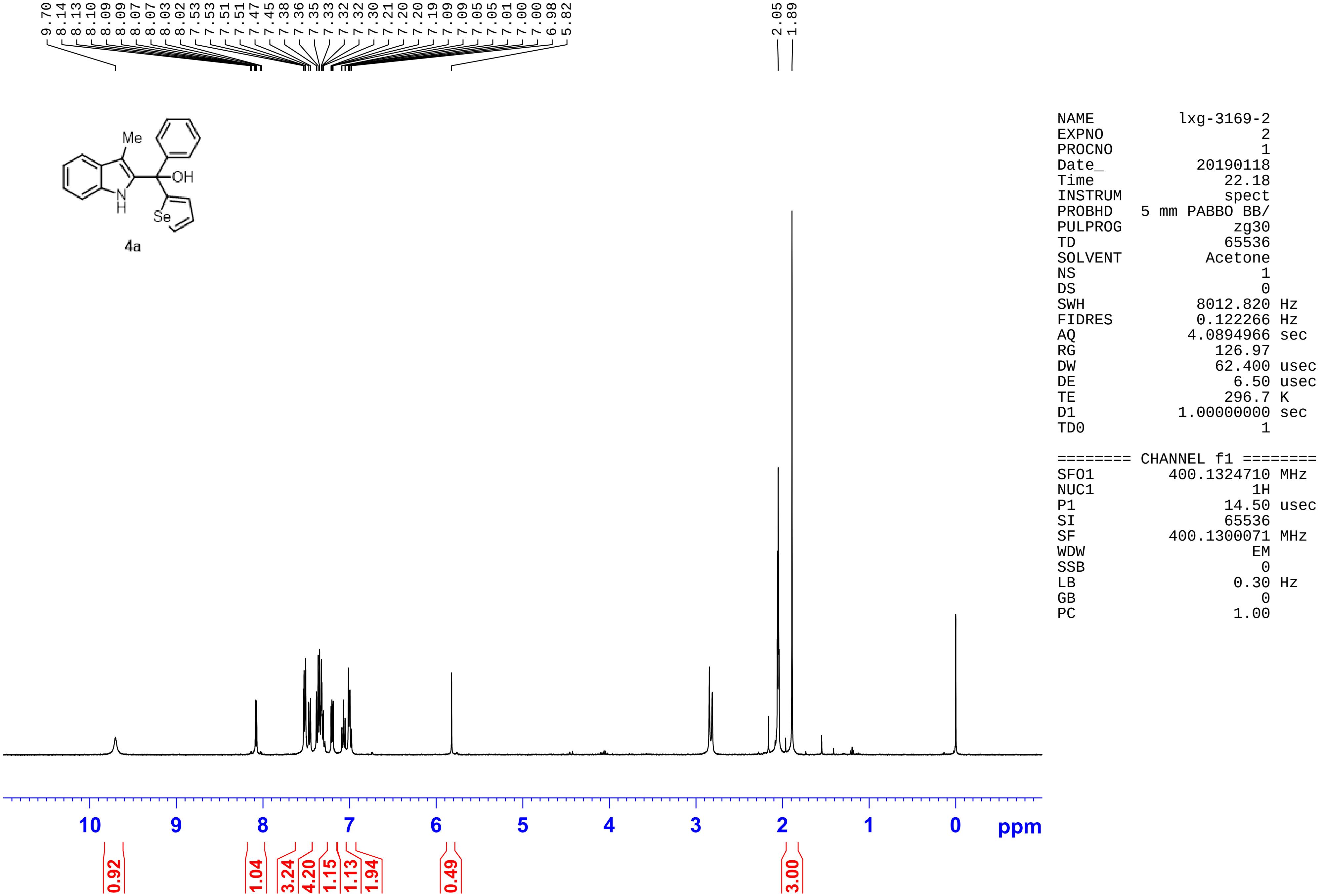

Supplementary Figure 40. ^{1}H NMR spectrum of **4a**

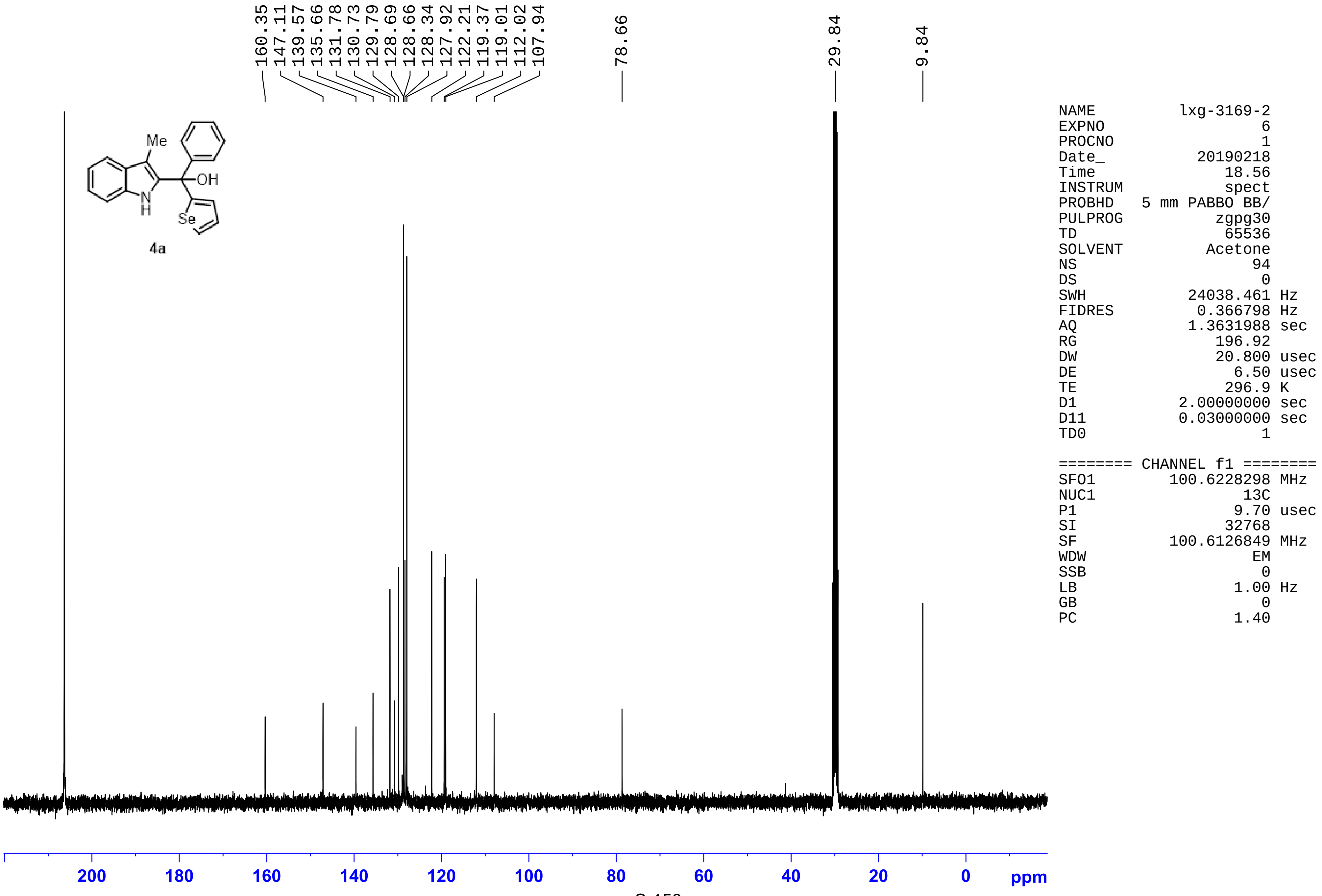

Supplementary Figure 41. ^{13}C NMR spectrum of **4a**

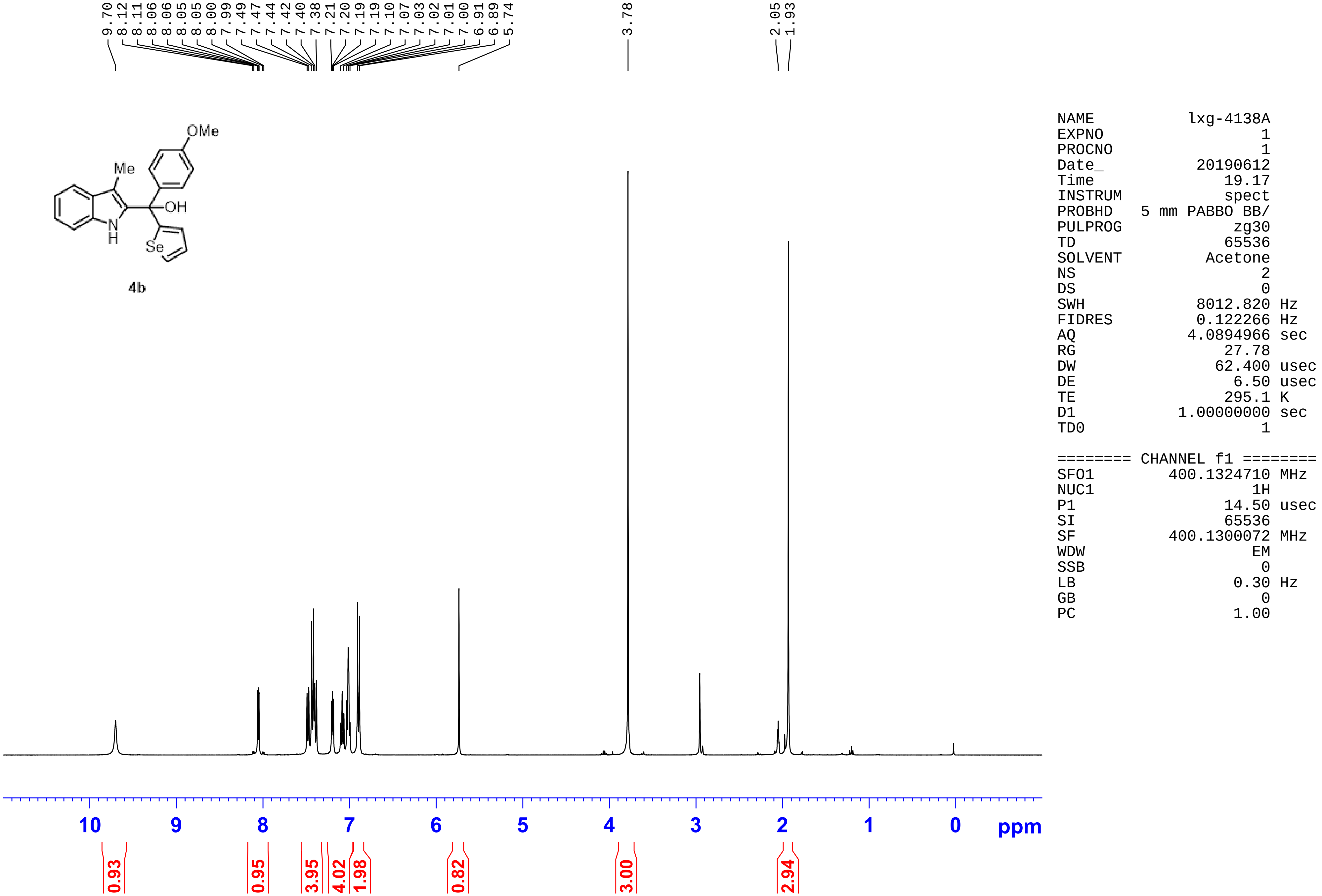

Supplementary Figure 42. ^{1}H NMR spectrum of **4b**

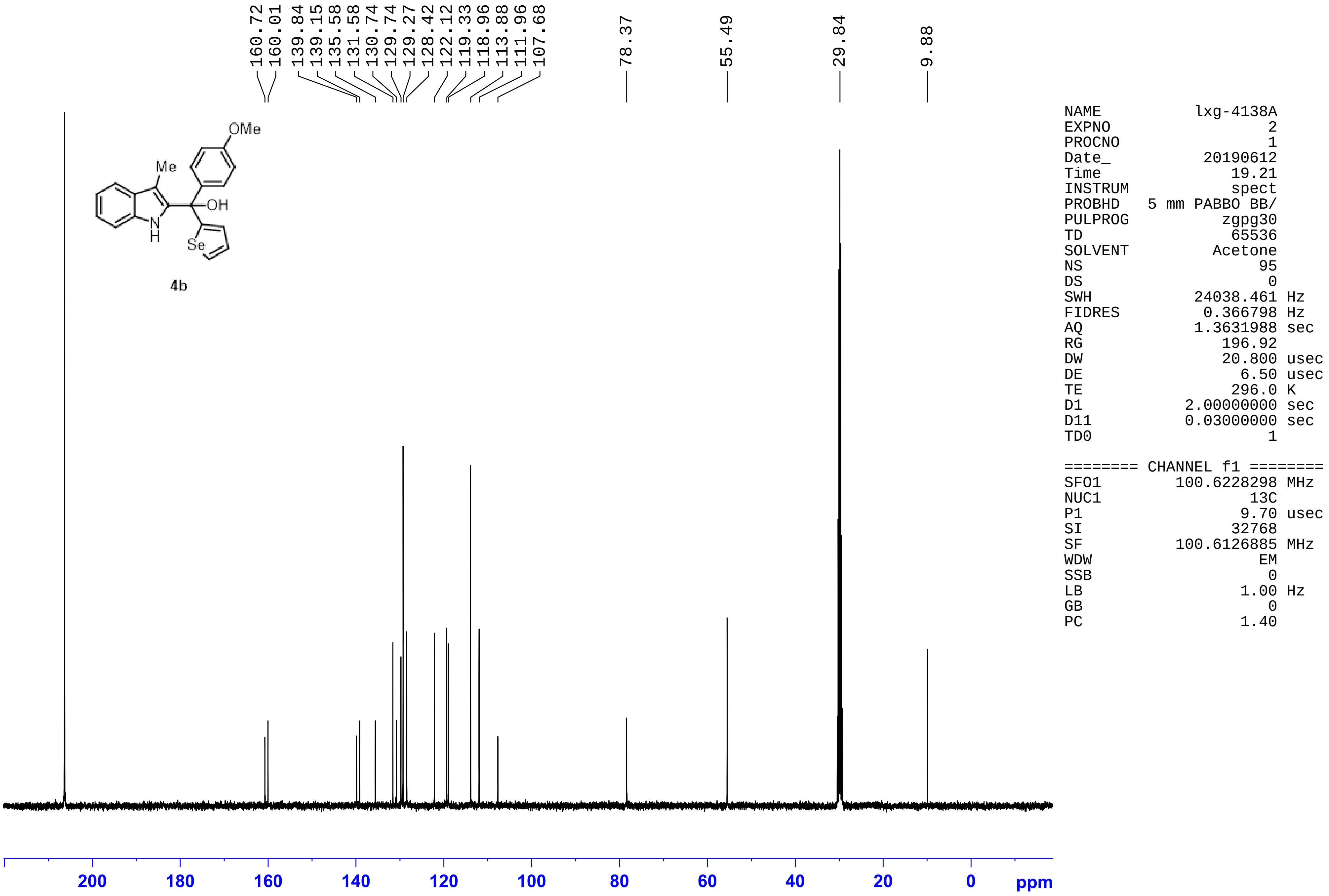

Supplementary Figure 43. ^{13}C NMR spectrum of **4b**

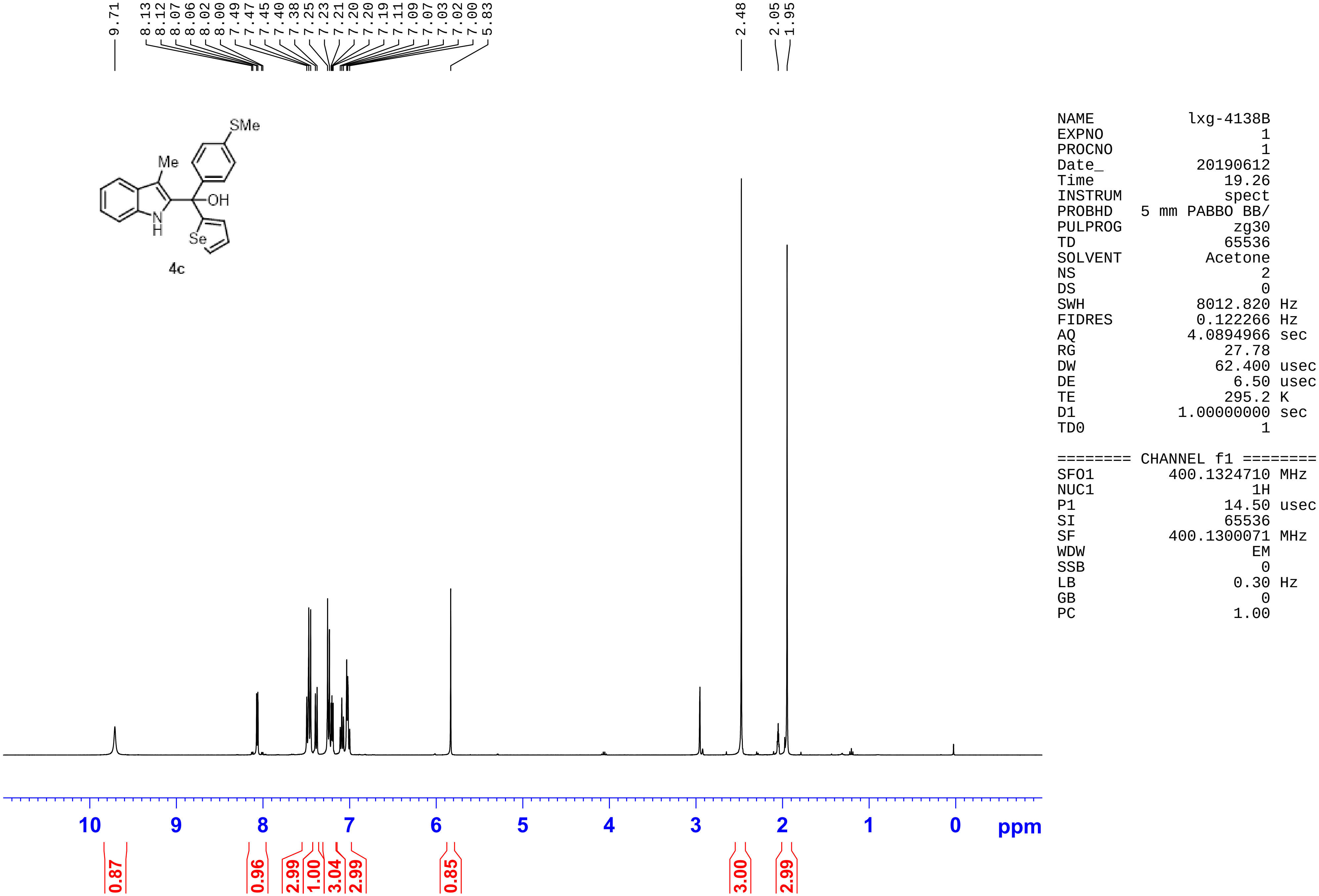

Supplementary Figure 44. ^{1}H NMR spectrum of **4c**

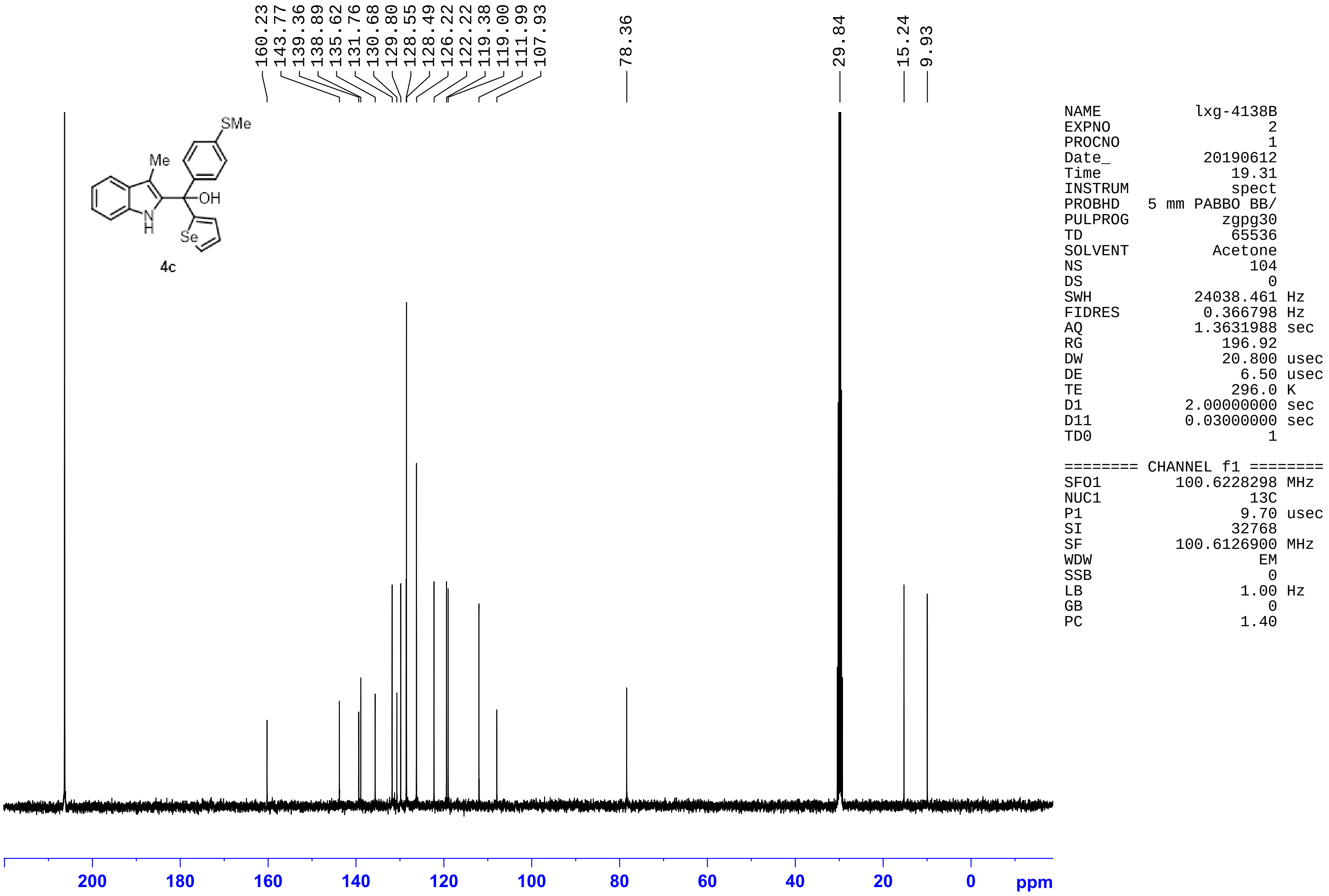

Supplementary Figure 45. ^{13}C NMR spectrum of **4c**

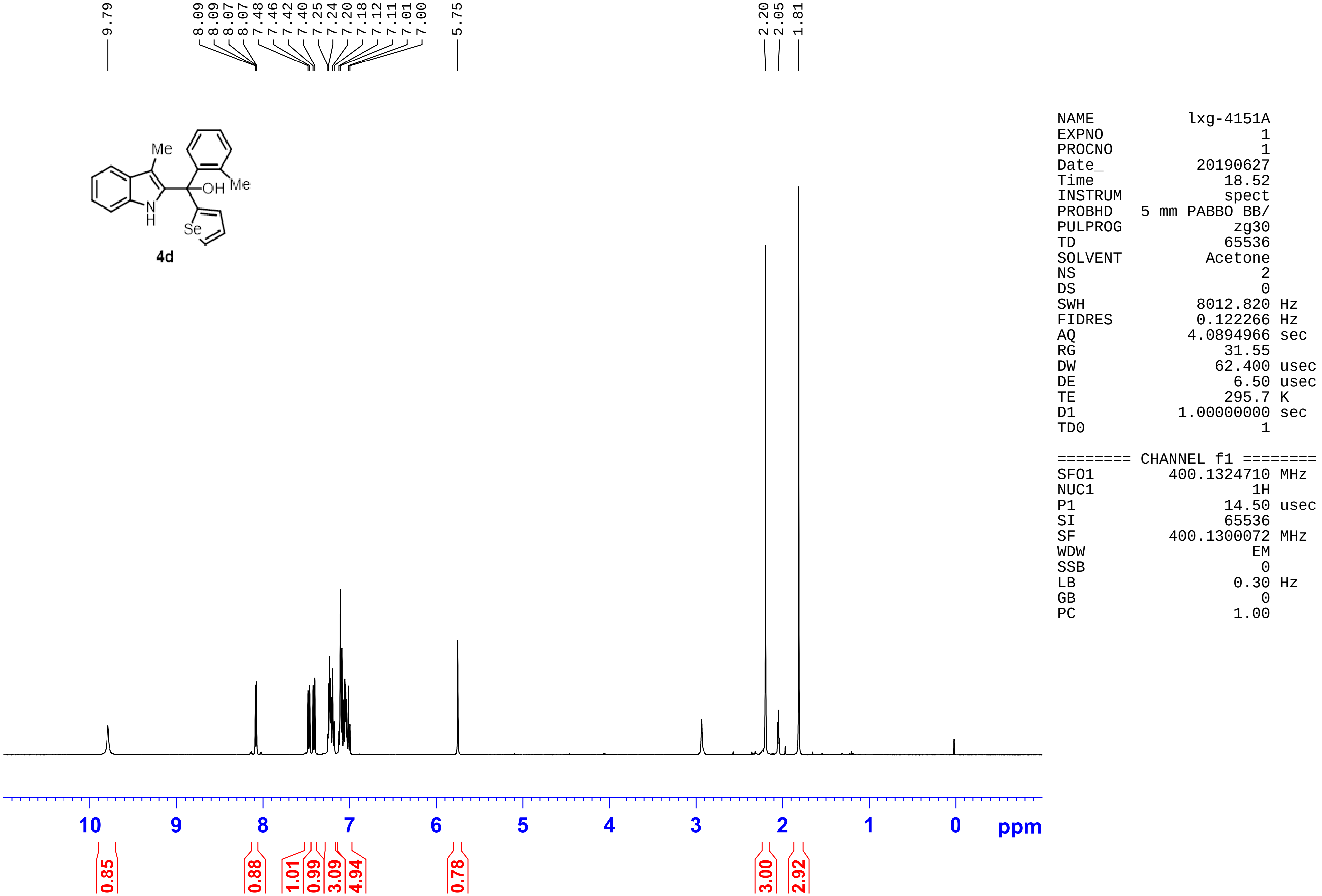

Supplementary Figure 46. ^{1}H NMR spectrum of **4d**

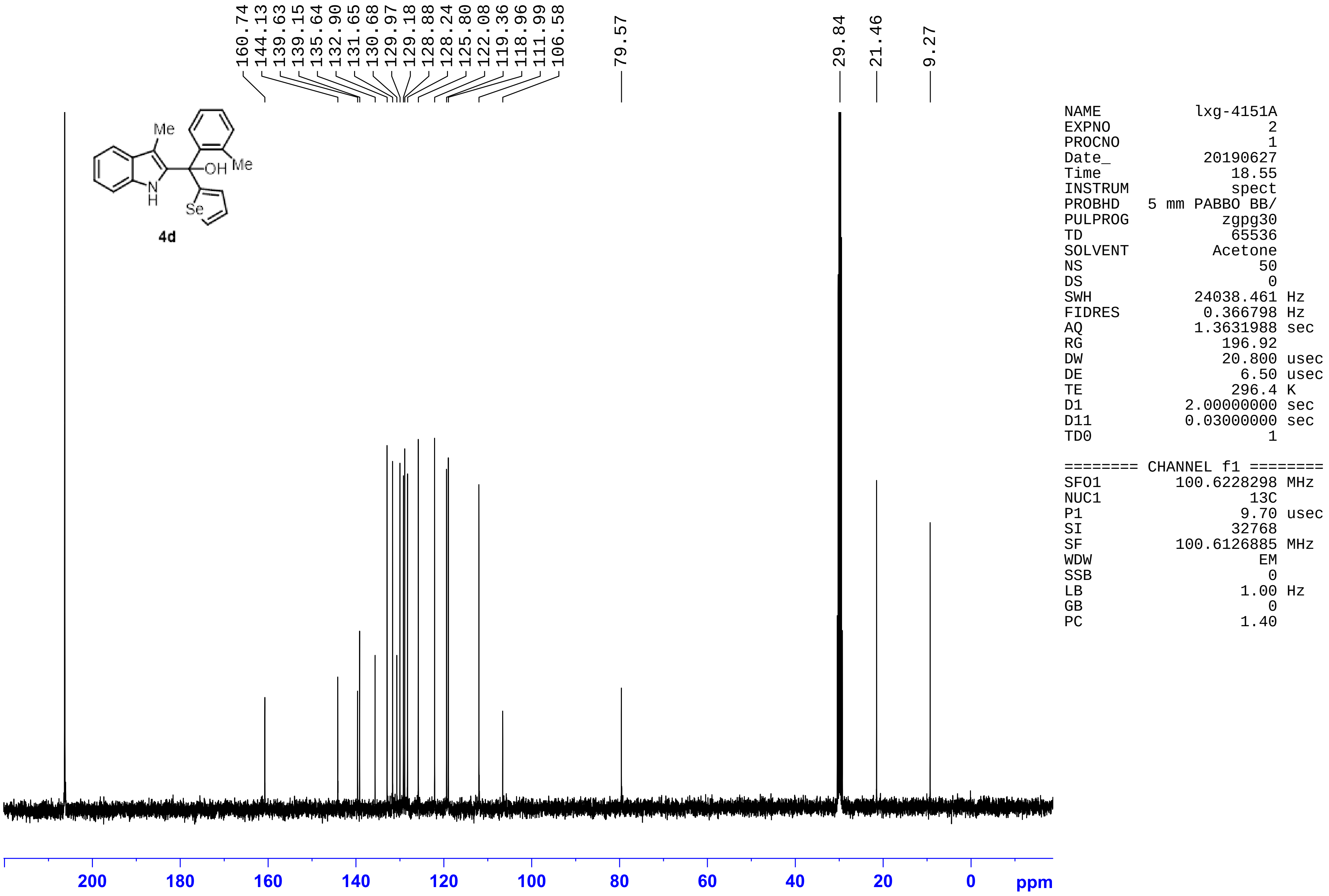

Supplementary Figure 47. ^{13}C NMR spectrum of **4d**

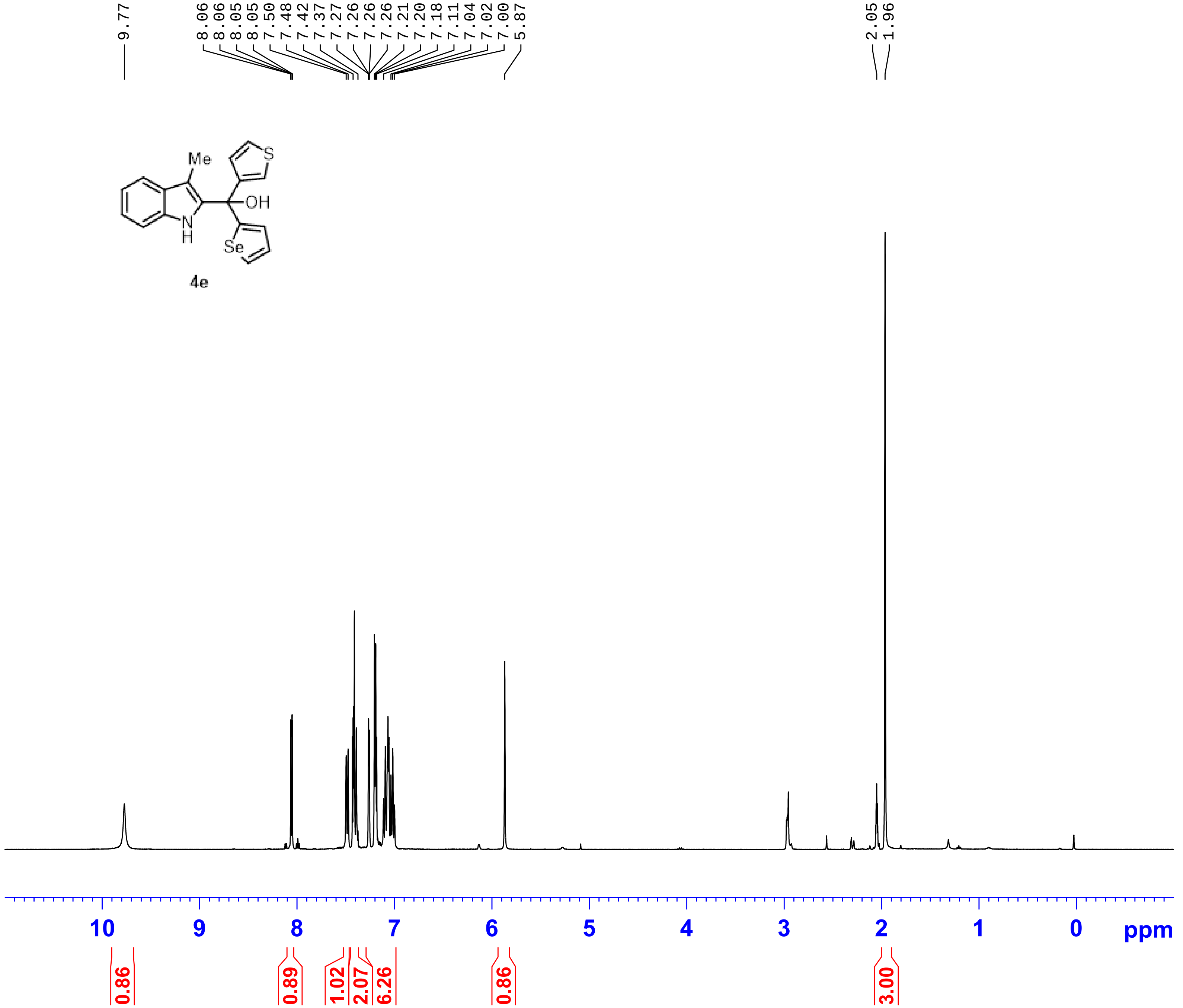


```
NAME            lxg-4151B
EXPNO                   1
PROCNO                  1
Date_            20190627
Time                18.58
INSTRUM             spect
PROBHD   5 mm PABBO BB/
PULPROG              zg30
TD                  65536
SOLVENT           Acetone
NS                      2
DS                      0
SWH              8012.820 Hz
FIDRES           0.122266 Hz
AQ              4.0894966 sec
RG                  31.55
DW                 62.400 usec
DE                   6.50 usec
TE                  295.7 K
D1             1.00000000 sec
TD0                     1

======== CHANNEL f1 ========
SFO1          400.1324710 MHz
NUC1                   1H
P1                  14.50 usec
SI                  65536
SF            400.1300072 MHz
WDW                    EM
SSB                     0
LB                   0.30 Hz
GB                      0
PC                   1.00
```


Supplementary Figure 48. ^{1}H NMR spectrum of **4e**

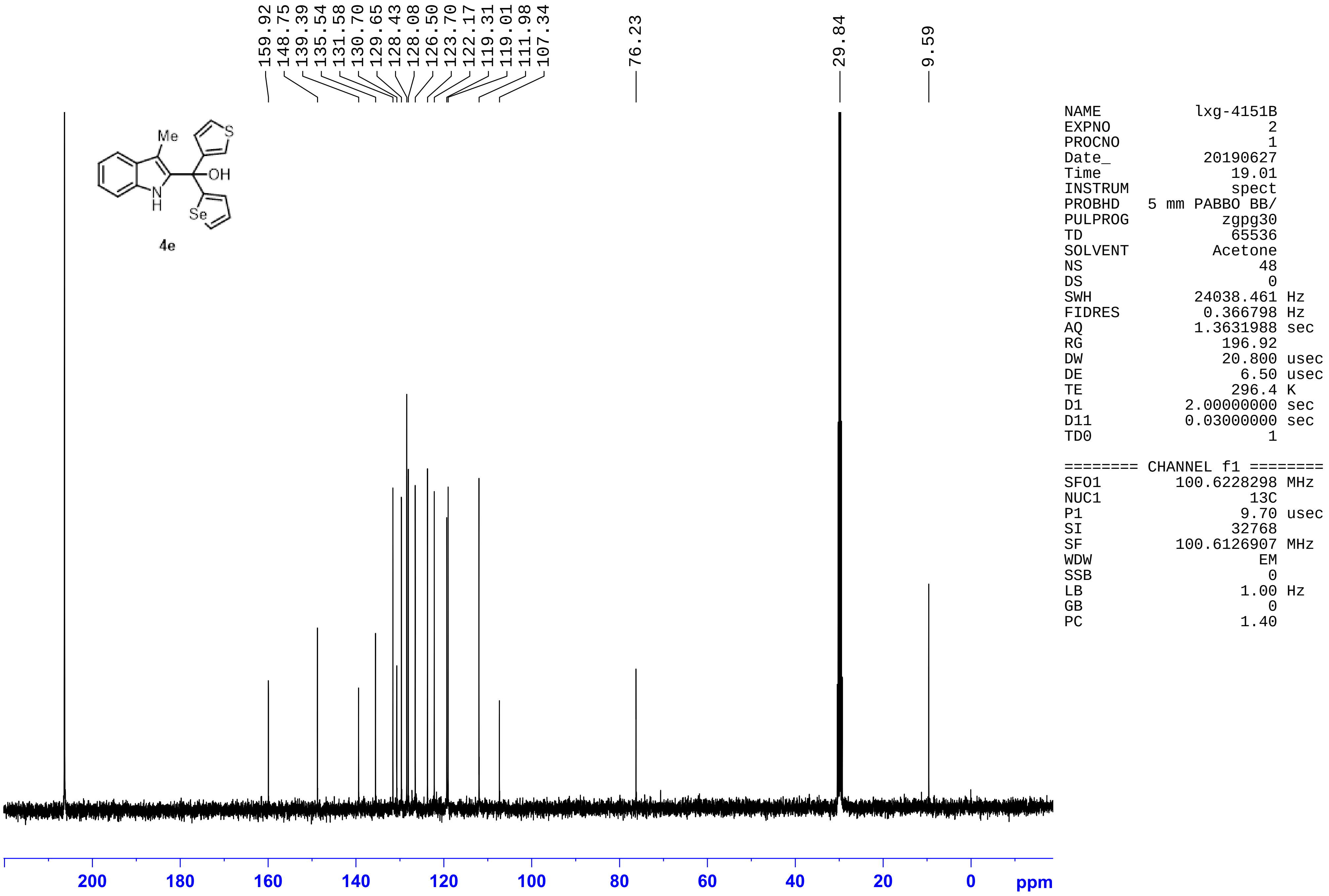

Supplementary Figure 49. ^{13}C NMR spectrum of **4e**

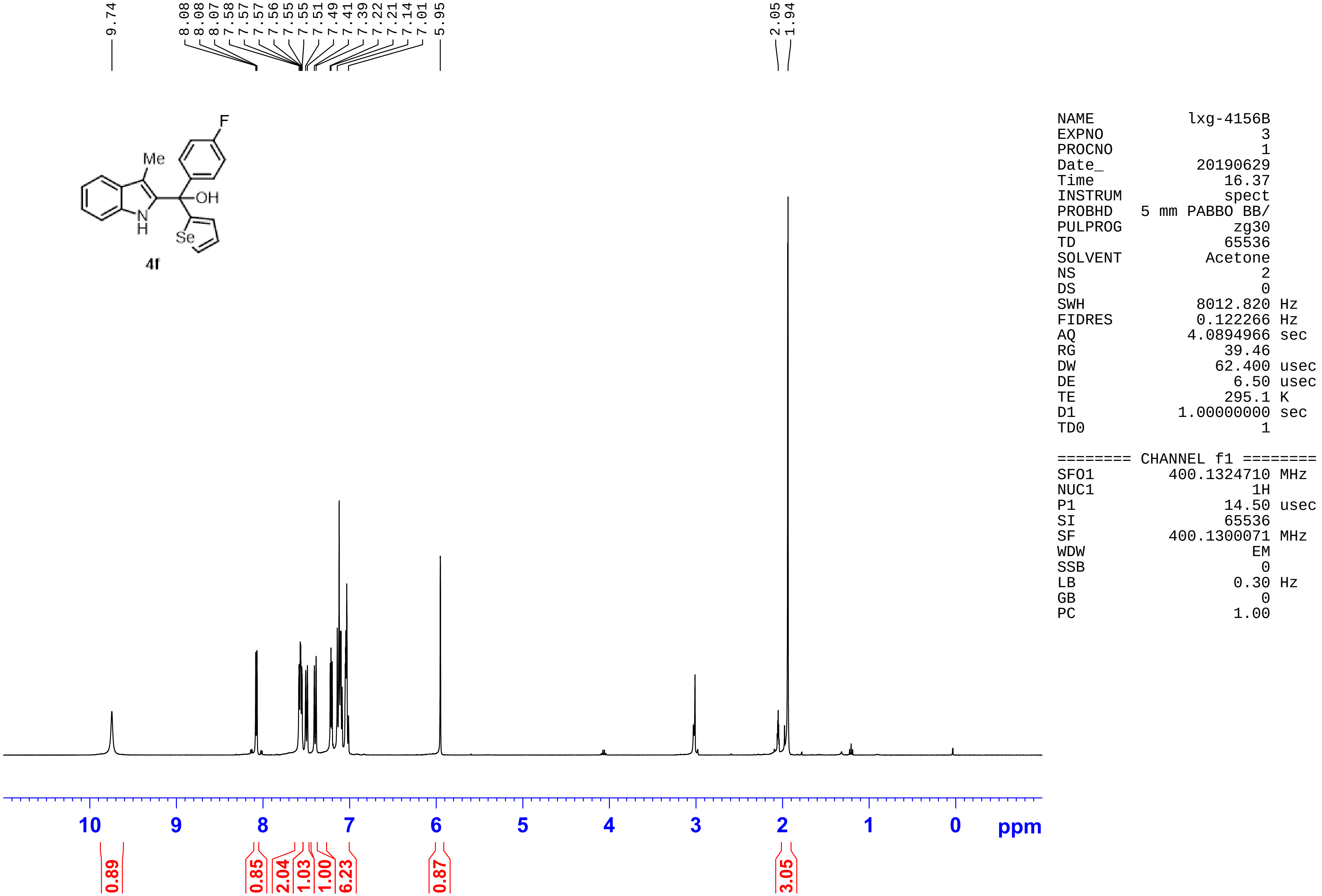

Supplementary Figure 50. ^{1}H NMR spectrum of **4f**

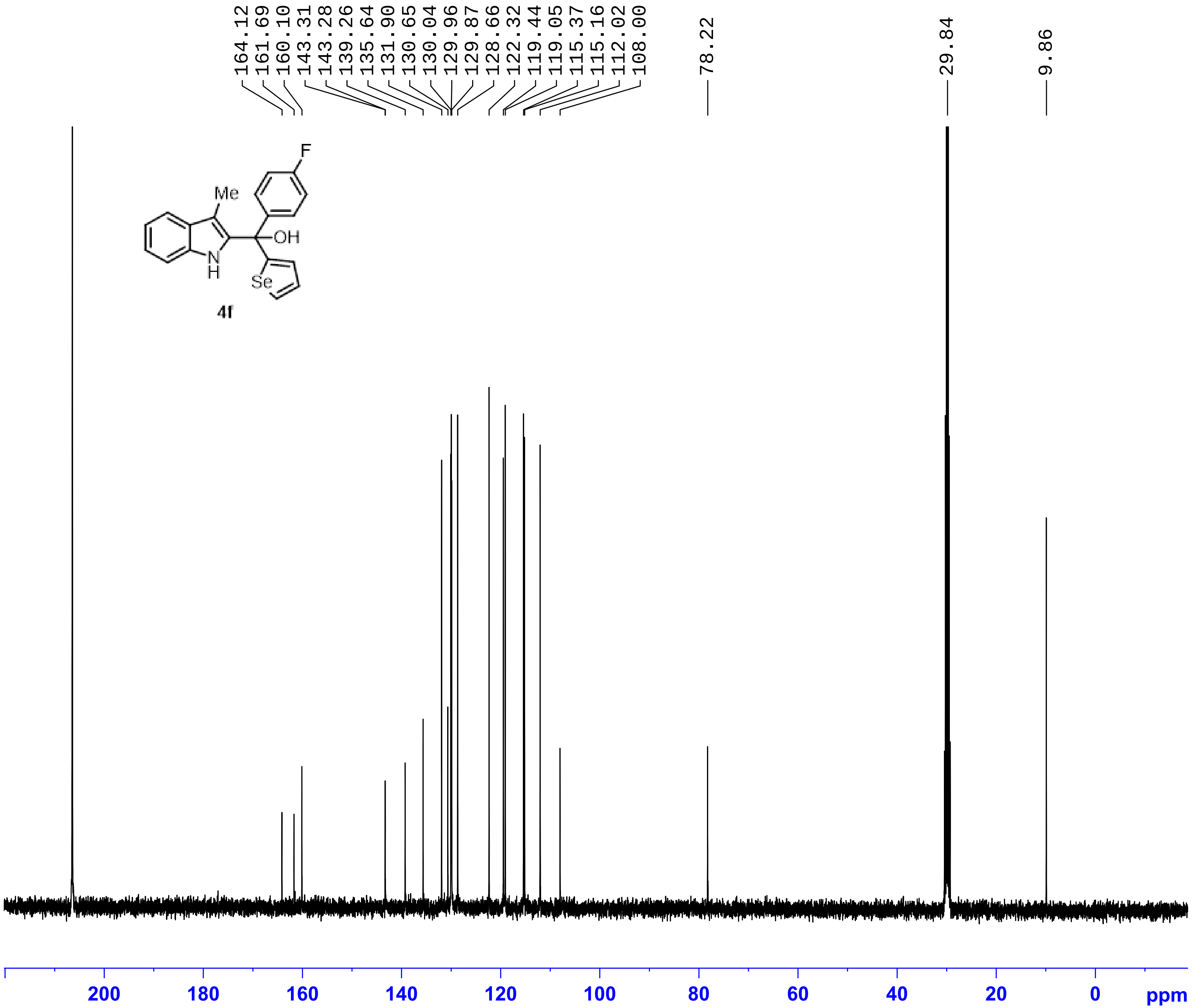

NAME	lxg-4156B
EXPNO	5
PROCNO	1
Date_	20190629
Time	16.40
INSTRUM	spect
PROBHD	5 mm PABBO BB/
PULPROG	zgpg30
TD	65536
SOLVENT	Acetone
NS	59
DS	0
SWH	24038.461 Hz
FIDRES	0.366798 Hz
AQ	1.3631988 sec
RG	196.92
DW	20.800 usec
DE	6.50 usec
TE	296.0 K
D1	2.00000000 sec
D11	0.03000000 sec
TD0	1

======== CHANNEL f1 ========

SFO1	100.6228298 MHz
NUC1	13C
P1	9.70 usec
SI	32768
SF	100.6126907 MHz
WDW	EM
SSB	0
LB	1.00 Hz
GB	0
PC	1.40

Supplementary Figure 51. ^{13}C NMR spectrum of **4f**

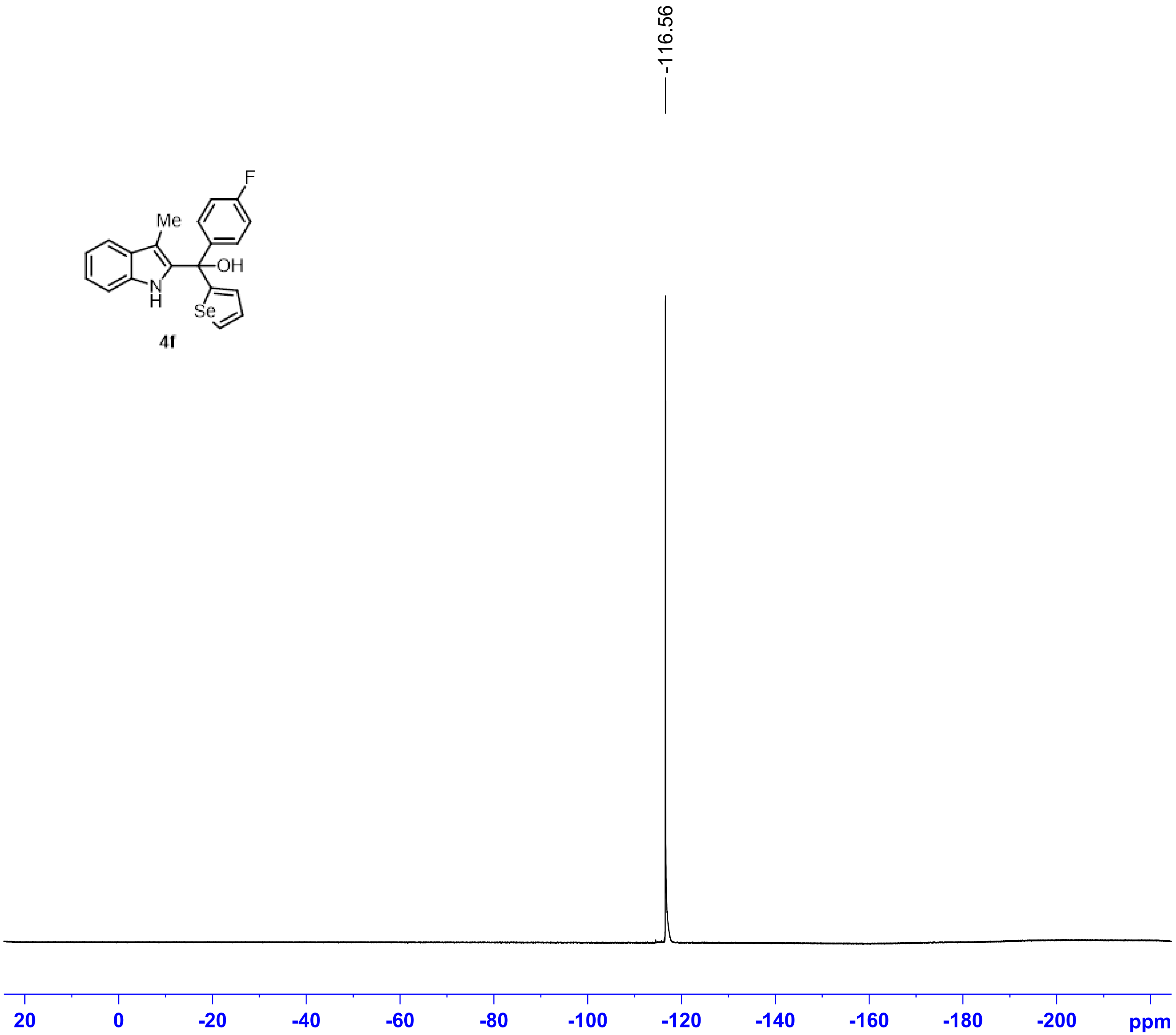


```
NAME           lxg-4156B
EXPNO                  4
PROCNO                 1
Date_           20190629
Time               16.28
INSTRUM            spect
PROBHD    5 mm PABBO BB/
PULPROG           zgpg30
TD                 65536
SOLVENT          Acetone
NS                    20
DS                     0
SWH            93750.000 Hz
FIDRES          1.430511 Hz
AQ             0.3495753 sec
RG                196.92
DW                 5.333 usec
DE                  6.50 usec
TE                 295.6 K
D1            2.00000000 sec
D11           0.03000000 sec
TD0                    1

======== CHANNEL f1 ========
SFO1         376.4607162 MHz
NUC1                 19F
P1                 14.70 usec
SI                 32768
SF           376.4983660 MHz
WDW                   EM
SSB                    0
LB                  1.00 Hz
GB                     0
PC                  1.40
```


Supplementary Figure 52. ^{19}F NMR spectrum of **4f**

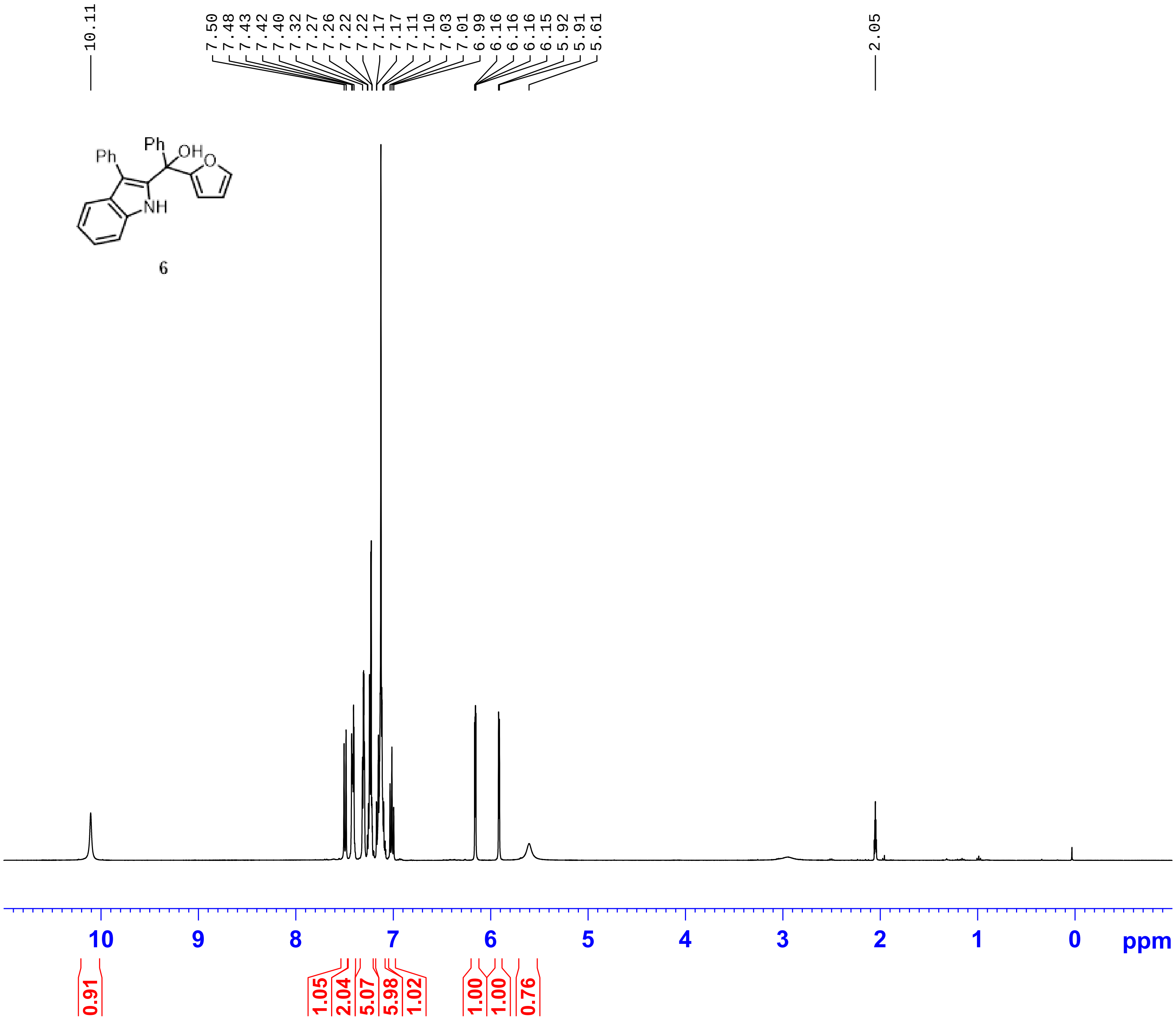

NAME	lxg-7088A	
EXPNO	1	
PROCNO	1	
Date_	20200711	
Time	15.40	
INSTRUM	spect	
PROBHD	5 mm PABBO BB/	
PULPROG	zg30	
TD	65536	
SOLVENT	Acetone	
NS	3	
DS	0	
SWH	8012.820	Hz
FIDRES	0.122266	Hz
AQ	4.0894966	sec
RG	31.55	
DW	62.400	usec
DE	6.50	usec
TE	297.3	K
D1	1.00000000	sec
TD0	1	
======== CHANNEL f1 ========		
SFO1	400.1324710	MHz
NUC1	1H	
P1	14.50	usec
SI	65536	
SF	400.1300069	MHz
WDW	EM	
SSB	0	
LB	0.30	Hz
GB	0	
PC	1.00	

Supplementary Figure 53. ^{1}H NMR spectrum of **6**

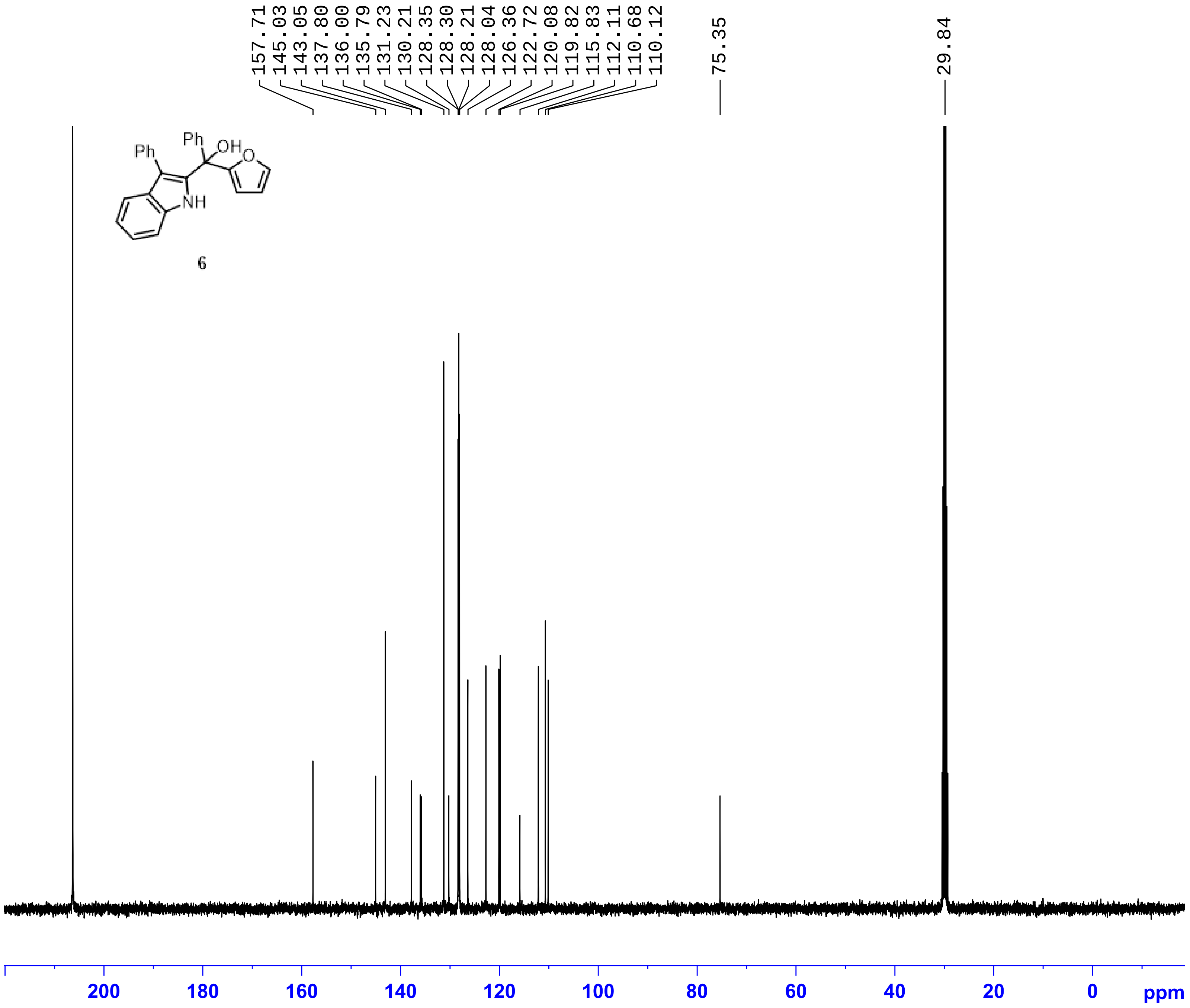

NAME	lxg-7088A
EXPNO	2
PROCNO	1
Date_	20200711
Time	15.41
INSTRUM	spect
PROBHD	5 mm PABBO BB/
PULPROG	zgpg30
TD	65536
SOLVENT	Acetone
NS	56
DS	0
SWH	24038.461 Hz
FIDRES	0.366798 Hz
AQ	1.3631988 sec
RG	196.92
DW	20.800 usec
DE	6.50 usec
TE	297.9 K
D1	2.00000000 sec
D11	0.03000000 sec
TD0	1

======== CHANNEL f1 ========

SFO1	100.6228298 MHz
NUC1	13C
P1	9.70 usec
SI	32768
SF	100.6126885 MHz
WDW	EM
SSB	0
LB	1.00 Hz
GB	0
PC	1.40

Supplementary Figure 54. ^{13}C NMR spectrum of **6**

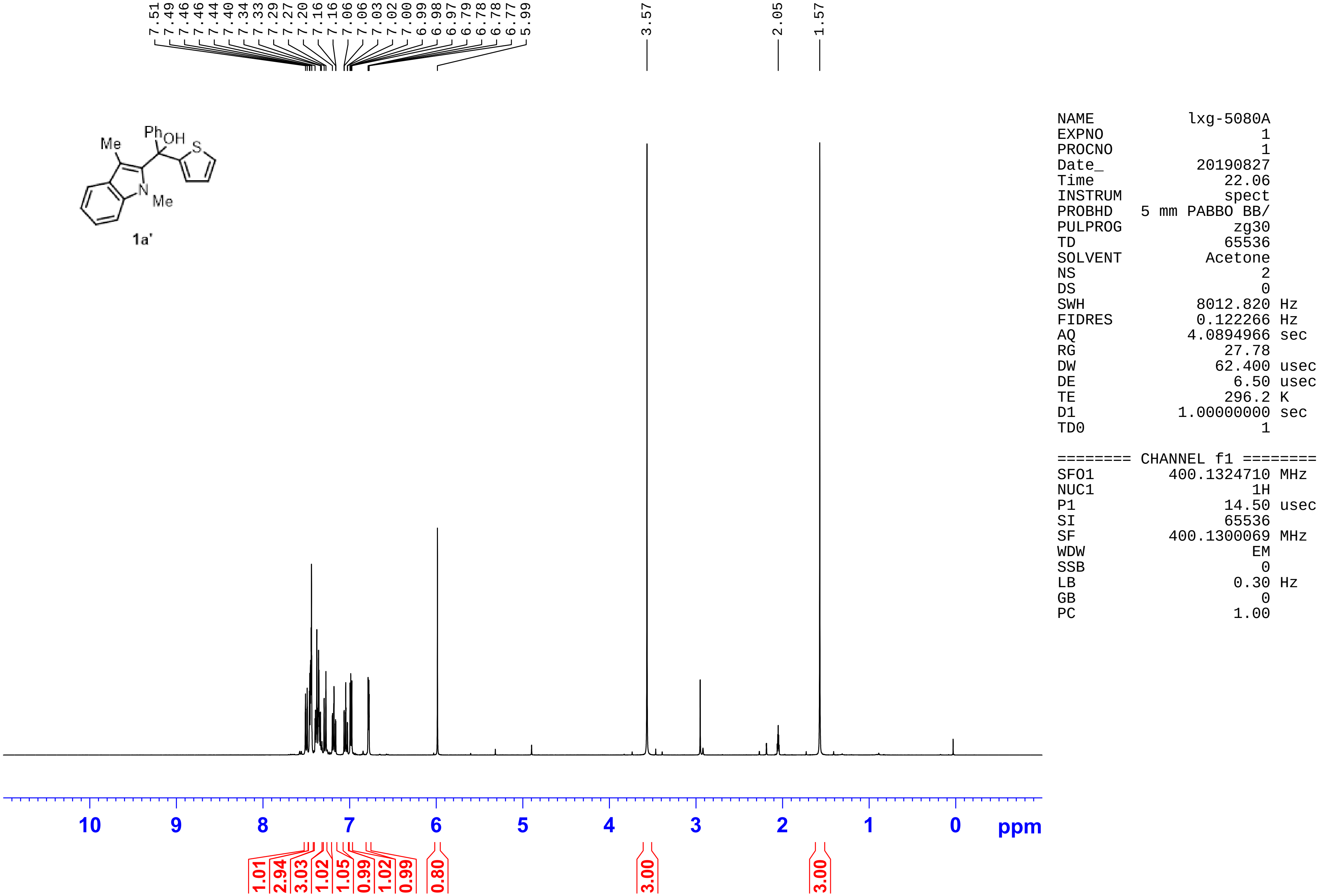

Supplementary Figure 55. ^{1}H NMR spectrum of **1a'**

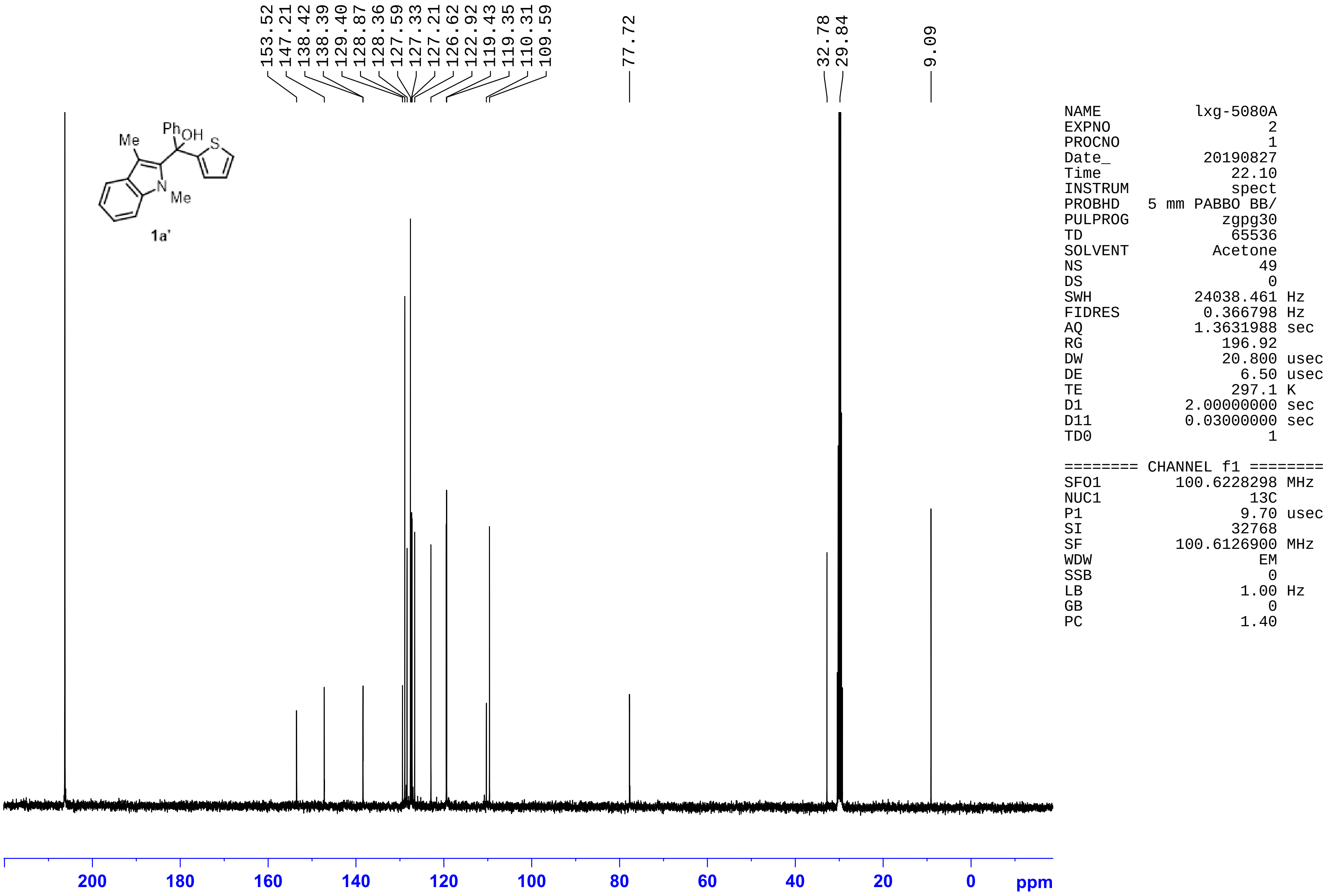

Supplementary Figure 56. ^{13}C NMR spectrum of **1a'**

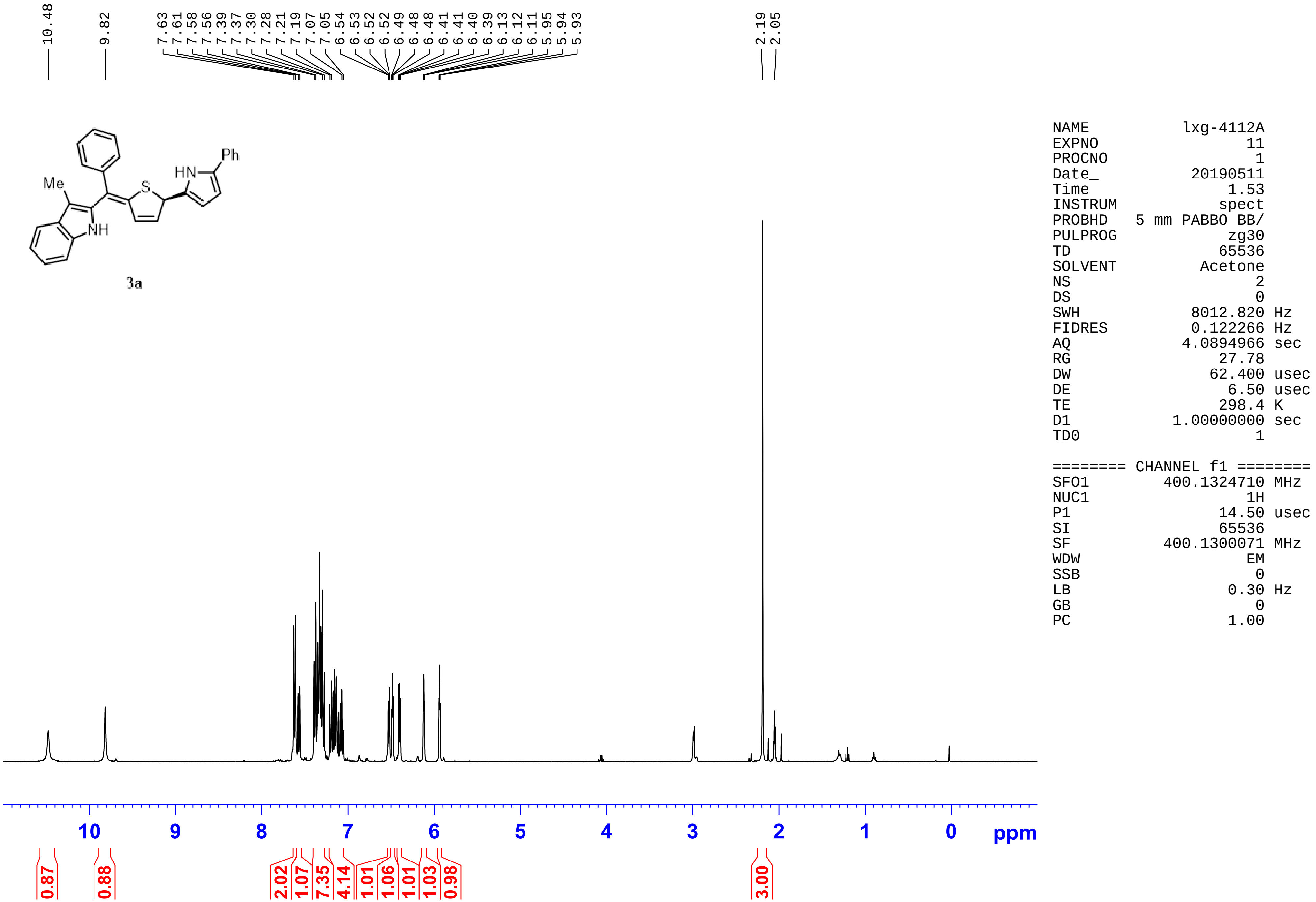

Supplementary Figure 57. ^{1}H NMR spectrum of **3a**

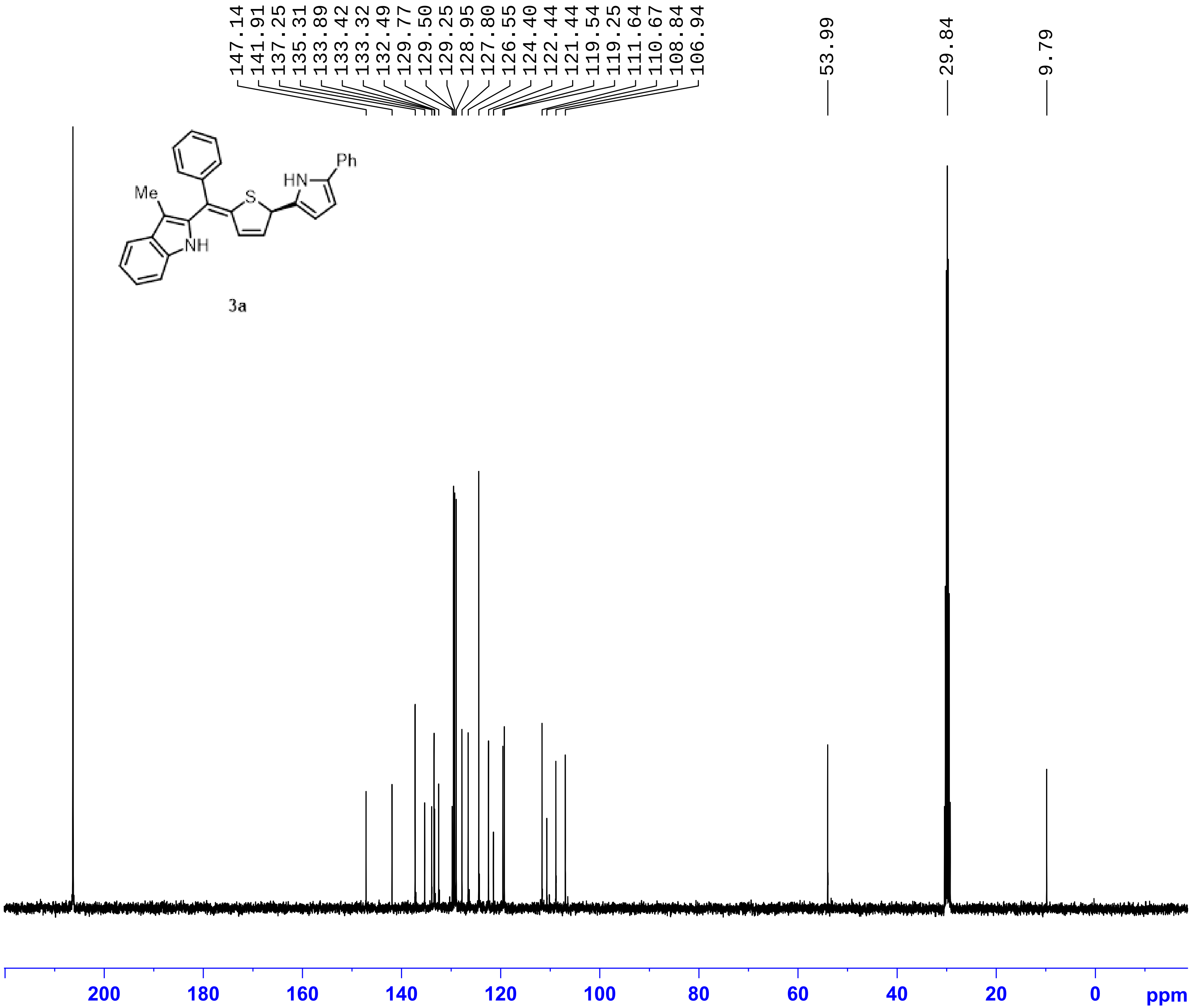

NAME	lxg-4112A
EXPNO	12
PROCNO	1
Date_	20190511
Time	1.58
INSTRUM	spect
PROBHD	5 mm PABBO BB/
PULPROG	zgpg30
TD	65536
SOLVENT	Acetone
NS	57
DS	0
SWH	24038.461 Hz
FIDRES	0.366798 Hz
AQ	1.3631988 sec
RG	196.92
DW	20.800 usec
DE	6.50 usec
TE	299.2 K
D1	2.00000000 sec
D11	0.03000000 sec
TD0	1

======== CHANNEL f1 ========

SFO1	100.6228298 MHz
NUC1	13C
P1	9.70 usec
SI	32768
SF	100.6126885 MHz
WDW	EM
SSB	0
LB	1.00 Hz
GB	0
PC	1.40

Supplementary Figure 58. ^{13}C NMR spectrum of **3a**

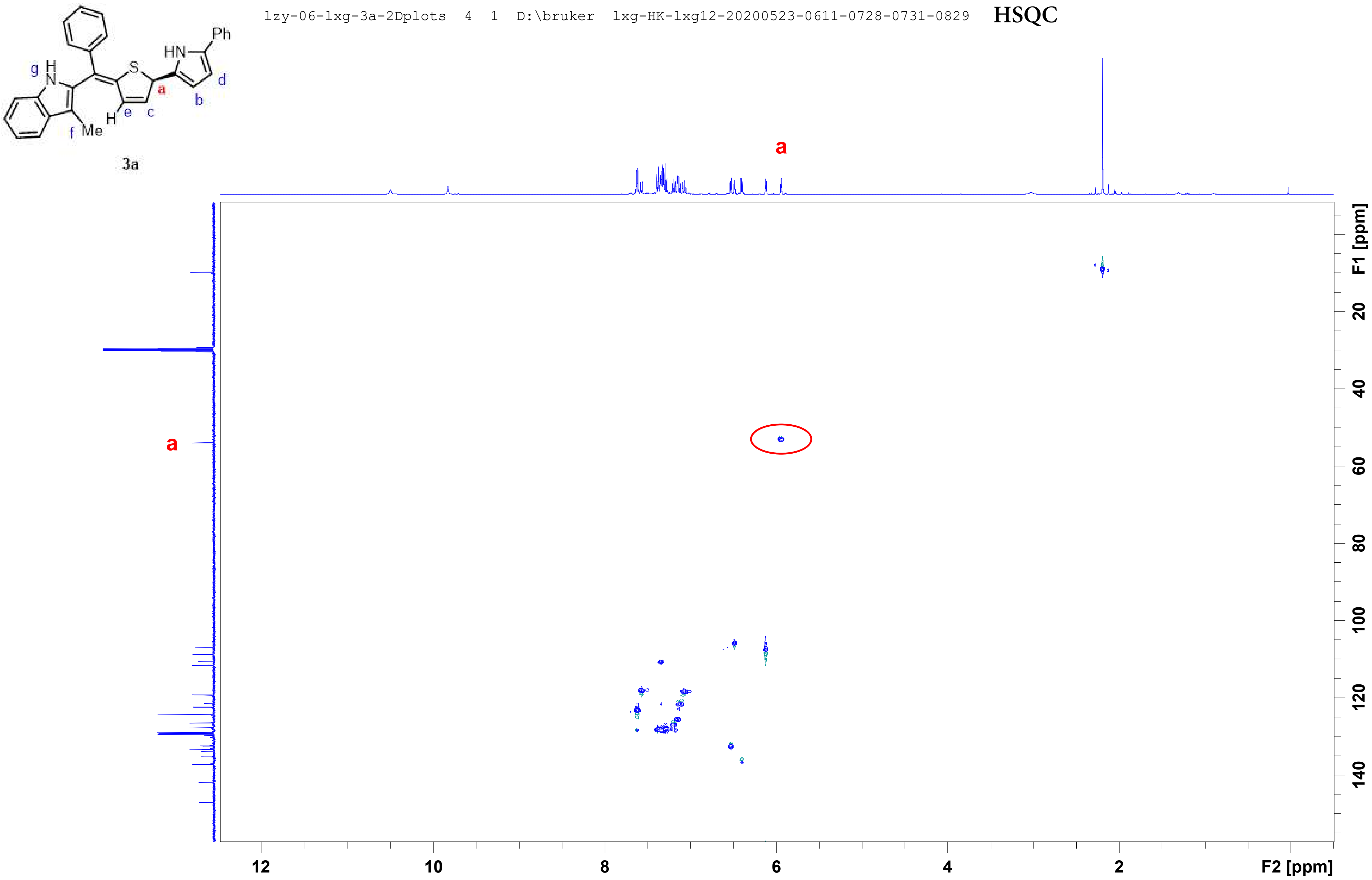

Supplementary Figure 59. HSQC spectrum of **3a**

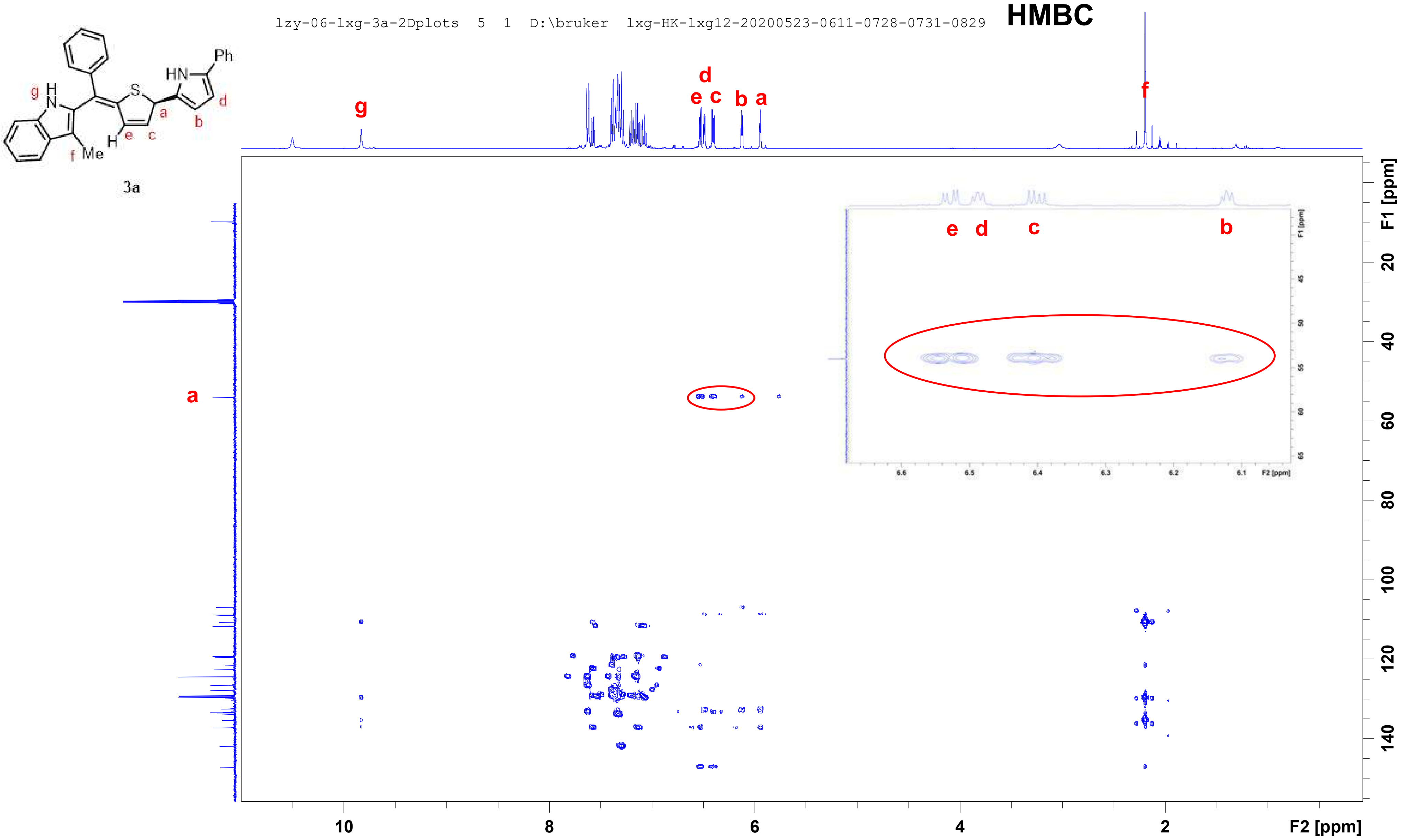

Supplementary Figure 60. HMBC spectrum of **3a**

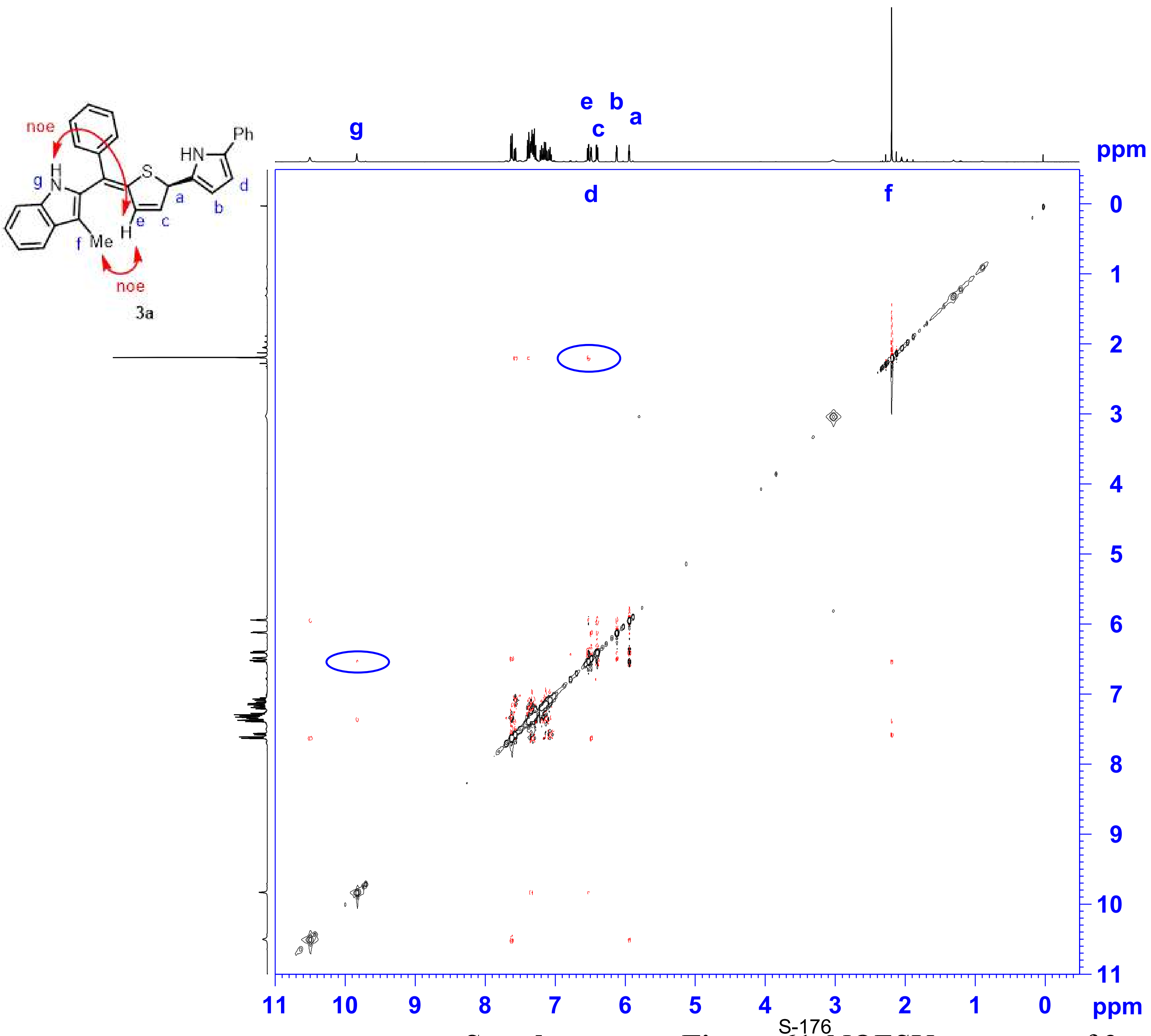

NAME	lzy-06-lxg-3a-2Dplots
EXPNO	6
PROCNO	1
Date_	20210307
Time	6.28
INSTRUM	spect
PROBHD	5 mm PABBO BB/
PULPROG	noesygpphpp
TD	2048
SOLVENT	Acetone
NS	8
DS	4
SWH	4795.396 Hz
FIDRES	2.341502 Hz
AQ	0.2135881 sec
RG	82.92
DW	104.267 usec
DE	6.50 usec
TE	296.7 K
D0	0.00008564 sec
D1	2.00000000 sec
D8	0.50000000 sec
D11	0.03000000 sec
D12	0.00002000 sec
D16	0.00020000 sec
IN0	0.00020820 sec
======== CHANNEL f1 ========	
SFO1	400.1320007 MHz
NUC1	1H
P1	14.50 usec
P2	29.00 usec
P17	2500.00 usec
ND0	1
TD	256
SFO1	400.132 MHz
FIDRES	18.762007 Hz
SW	12.004 ppm
FnMODE	States-TPPI
SI	1024
SF	400.1300069 MHz
WDW	QSINE
SSB	2
LB	0.00 Hz
GB	0
PC	1.00
SI	1024
MC2	States-TPPI
SF	400.1300000 MHz
WDW	QSINE
SSB	2
LB	0.00 Hz
GB	0

Supplementary Figure 61. NOESY spectrum of **3a**

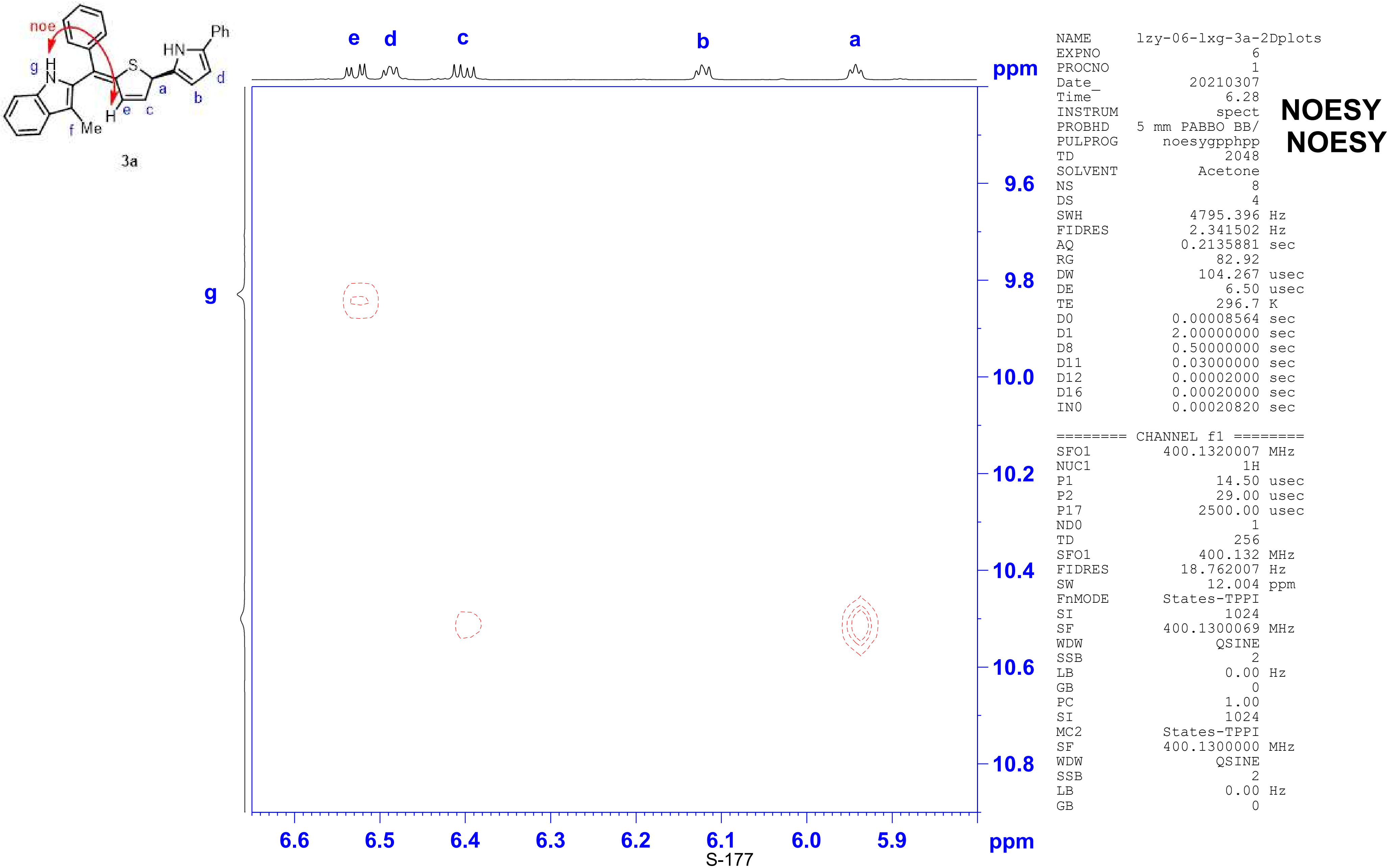

Supplementary Figure 62. NOESY spectrum (enlarged) of **3a**

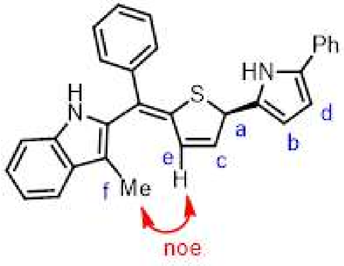

3a

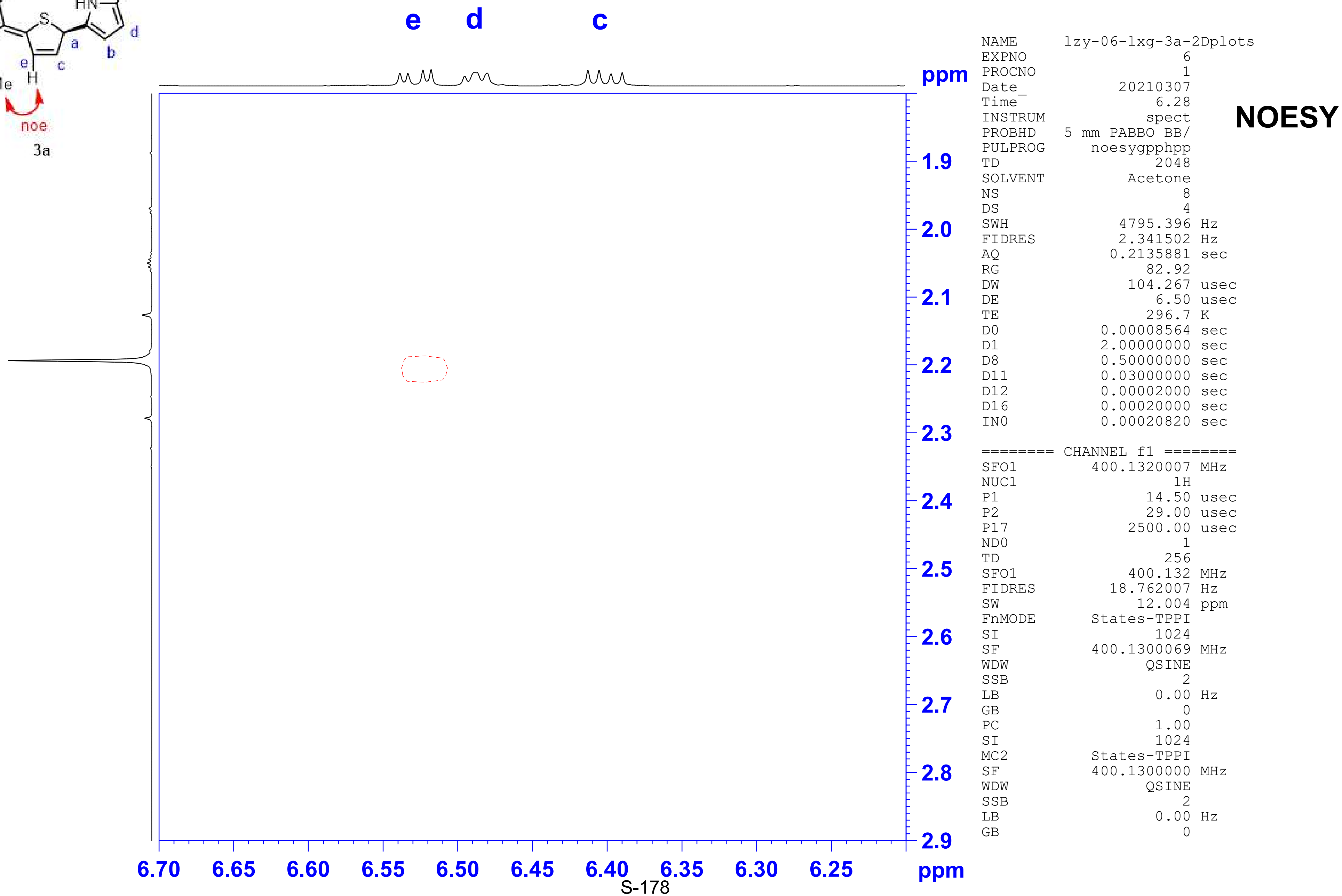

Supplementary Figure 63. NOESY spectrum (enlarged) of **3a**

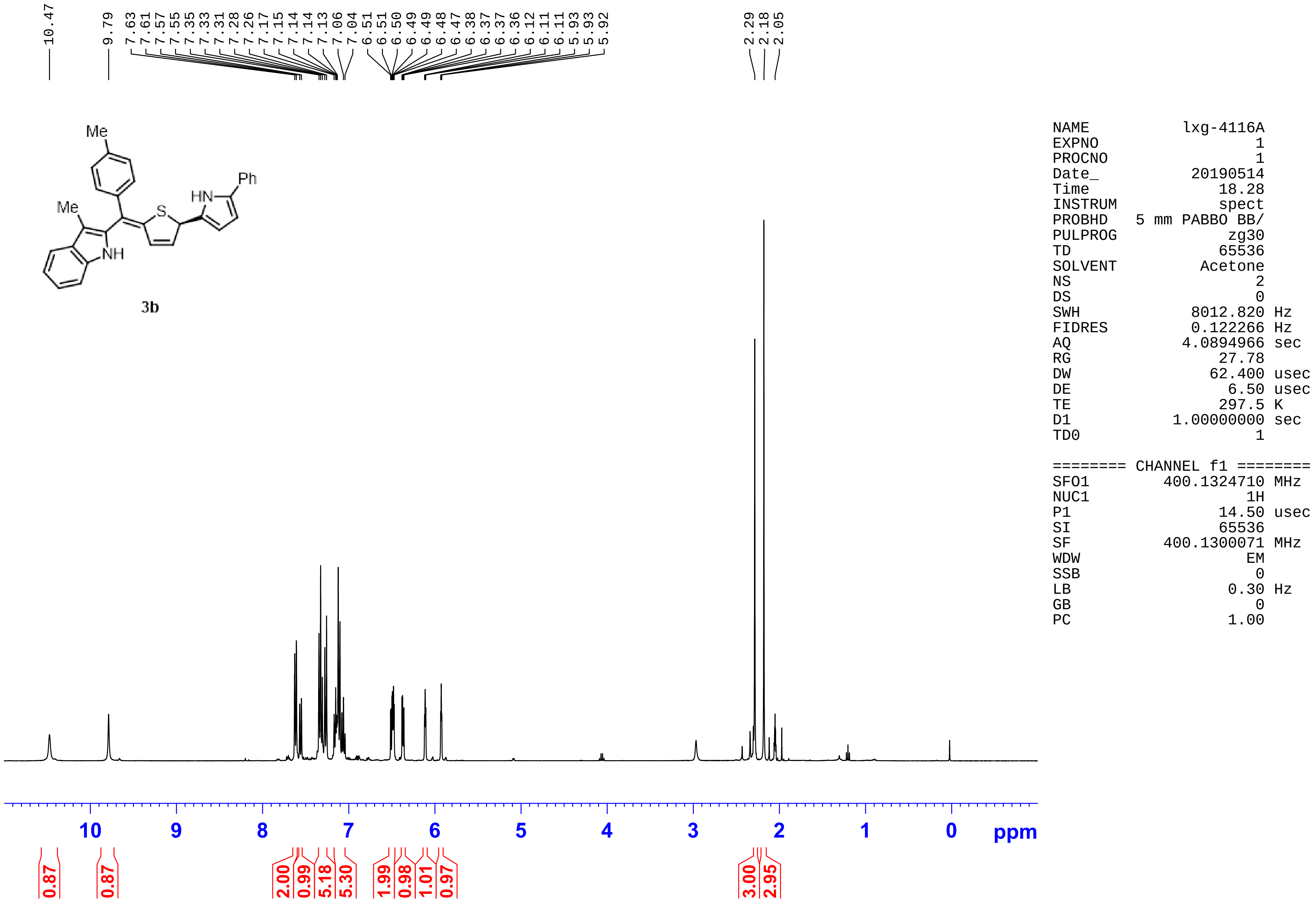

Supplementary Figure 64. ^{1}H NMR spectrum of **3b**

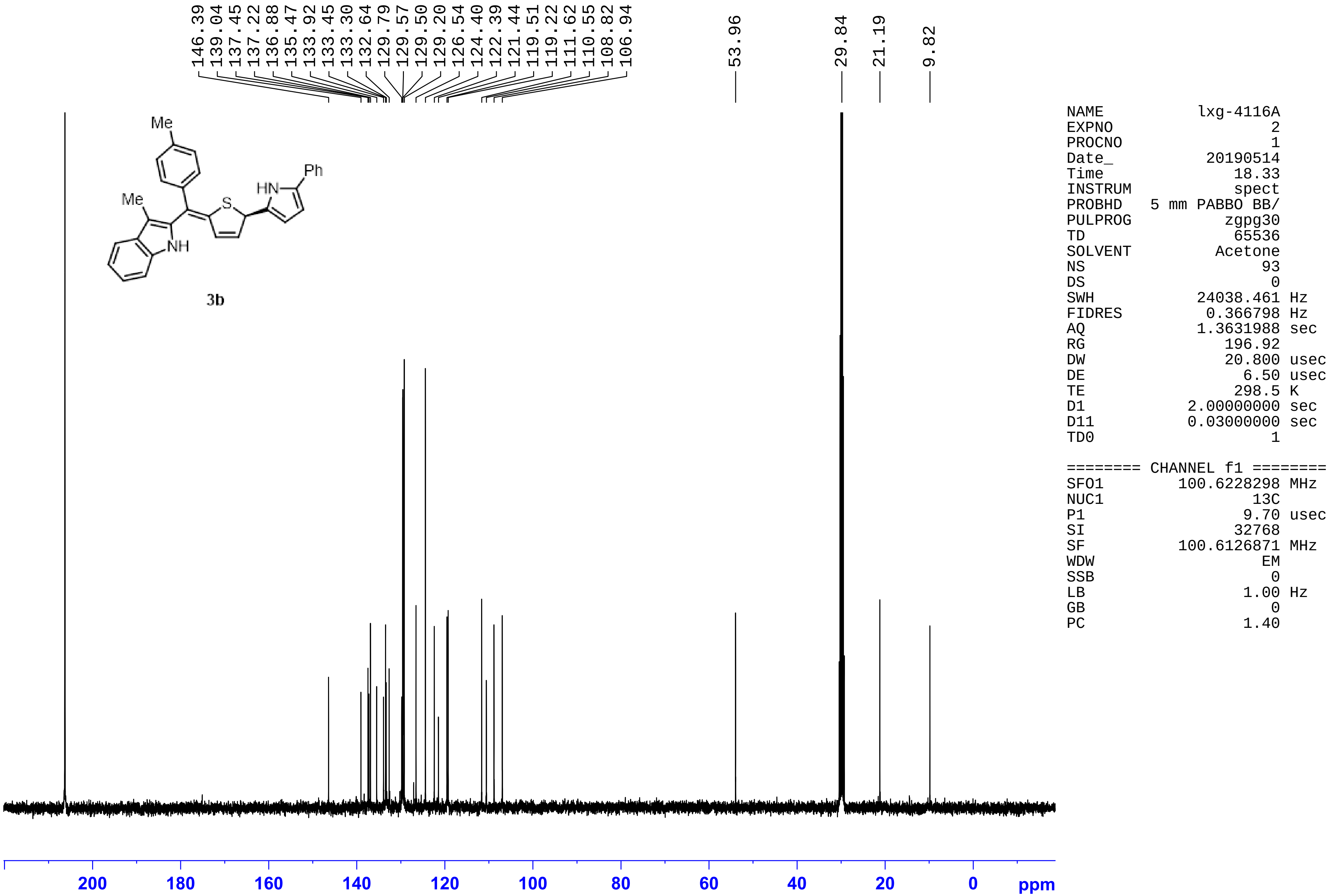

Supplementary Figure 65. ^{13}C NMR spectrum of **3b**

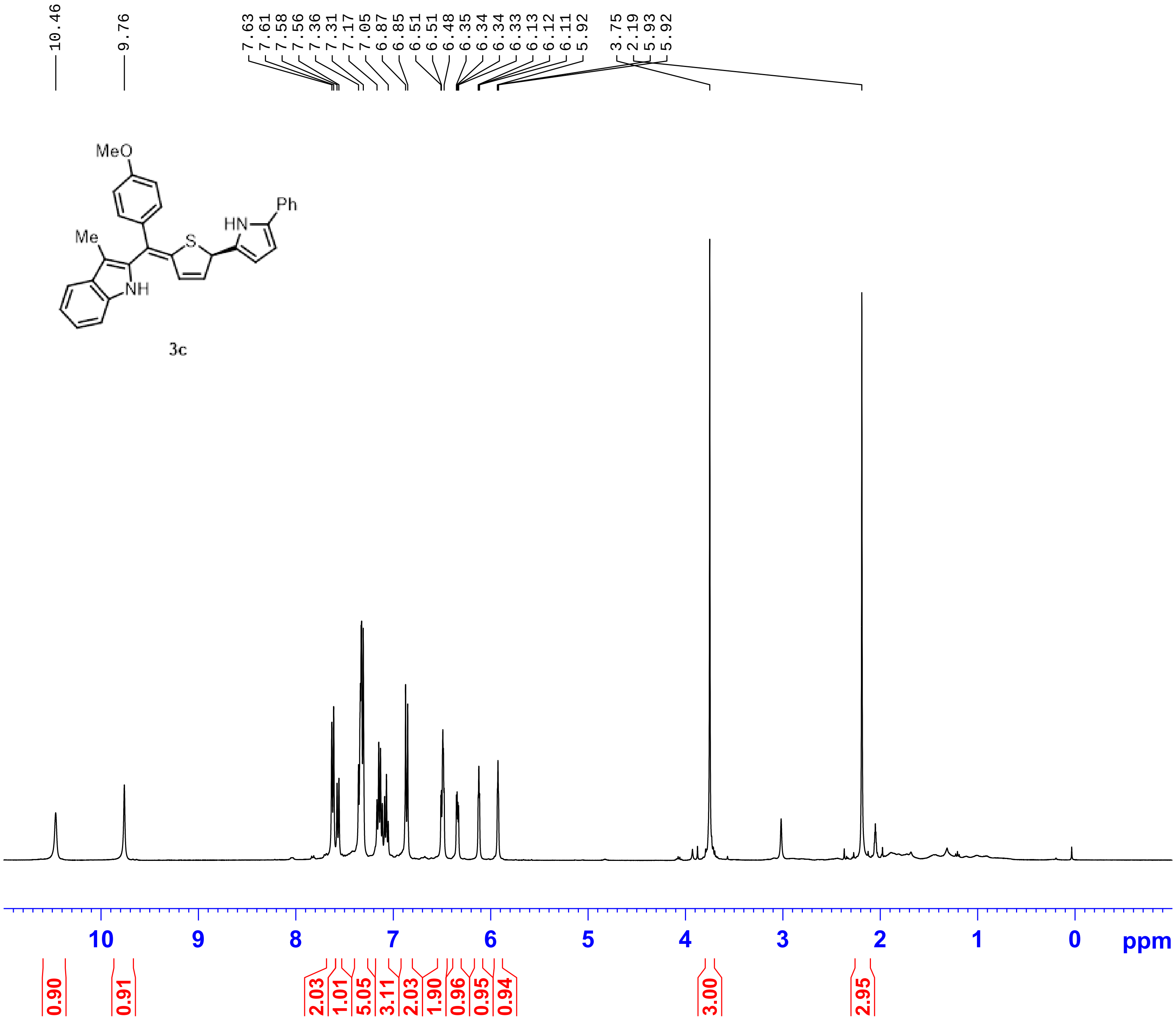

NAME	lxg-4112B	
EXPNO	1	
PROCNO	1	
Date_	20190511	
Time	1.43	
INSTRUM	spect	
PROBHD	5 mm PABBO BB/	
PULPROG	zg30	
TD	65536	
SOLVENT	Acetone	
NS	2	
DS	0	
SWH	8012.820	Hz
FIDRES	0.122266	Hz
AQ	4.0894966	sec
RG	22.47	
DW	62.400	usec
DE	6.50	usec
TE	298.4	K
D1	1.00000000	sec
TD0	1	

======== CHANNEL f1 ========

SFO1	400.1324710	MHz
NUC1	1H	
P1	14.50	usec
SI	65536	
SF	400.1300072	MHz
WDW	EM	
SSB	0	
LB	0.30	Hz
GB	0	
PC	1.00	

Supplementary Figure 66. ^{1}H NMR spectrum of **3c**

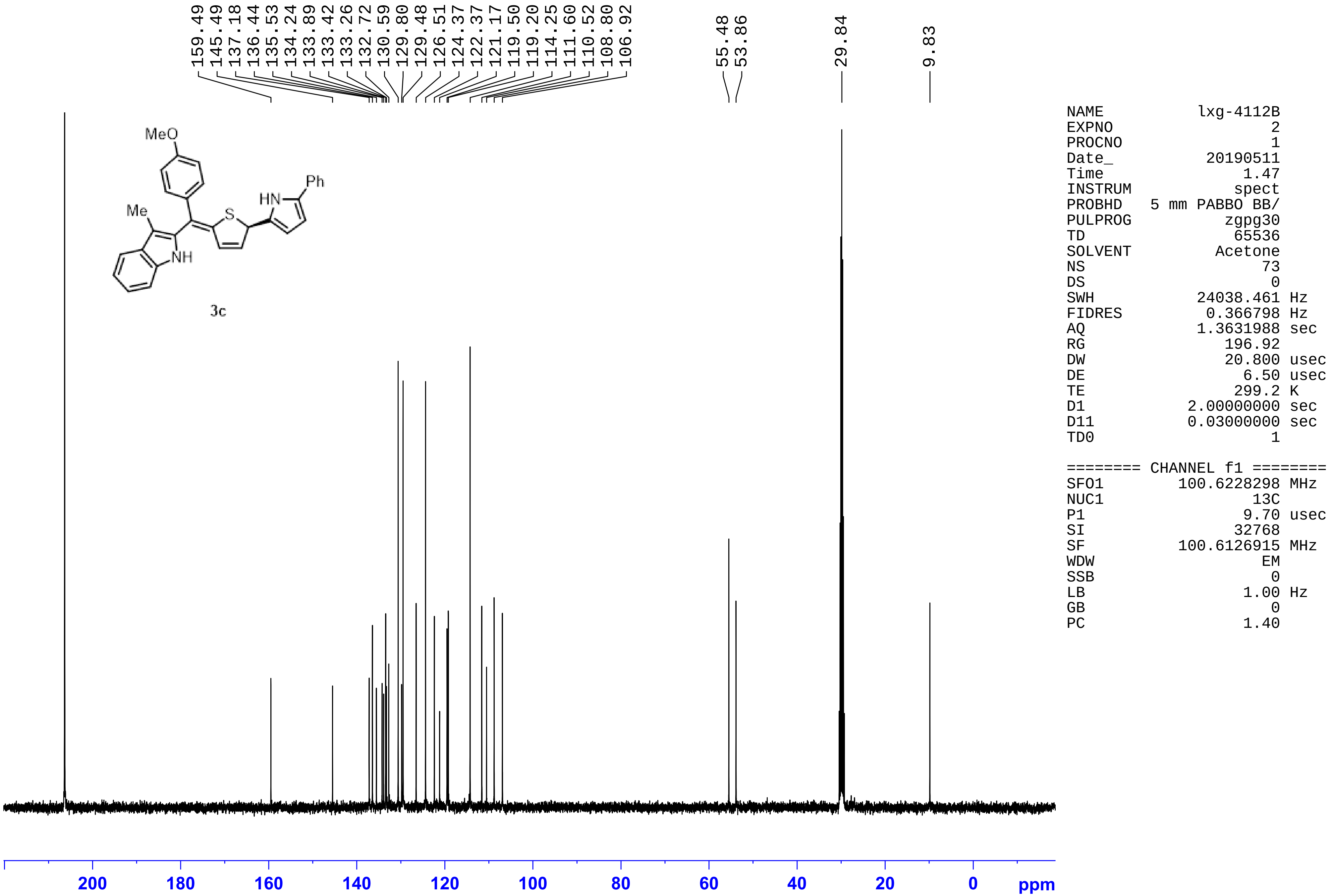

Supplementary Figure 67. ^{13}C NMR spectrum of **3c**

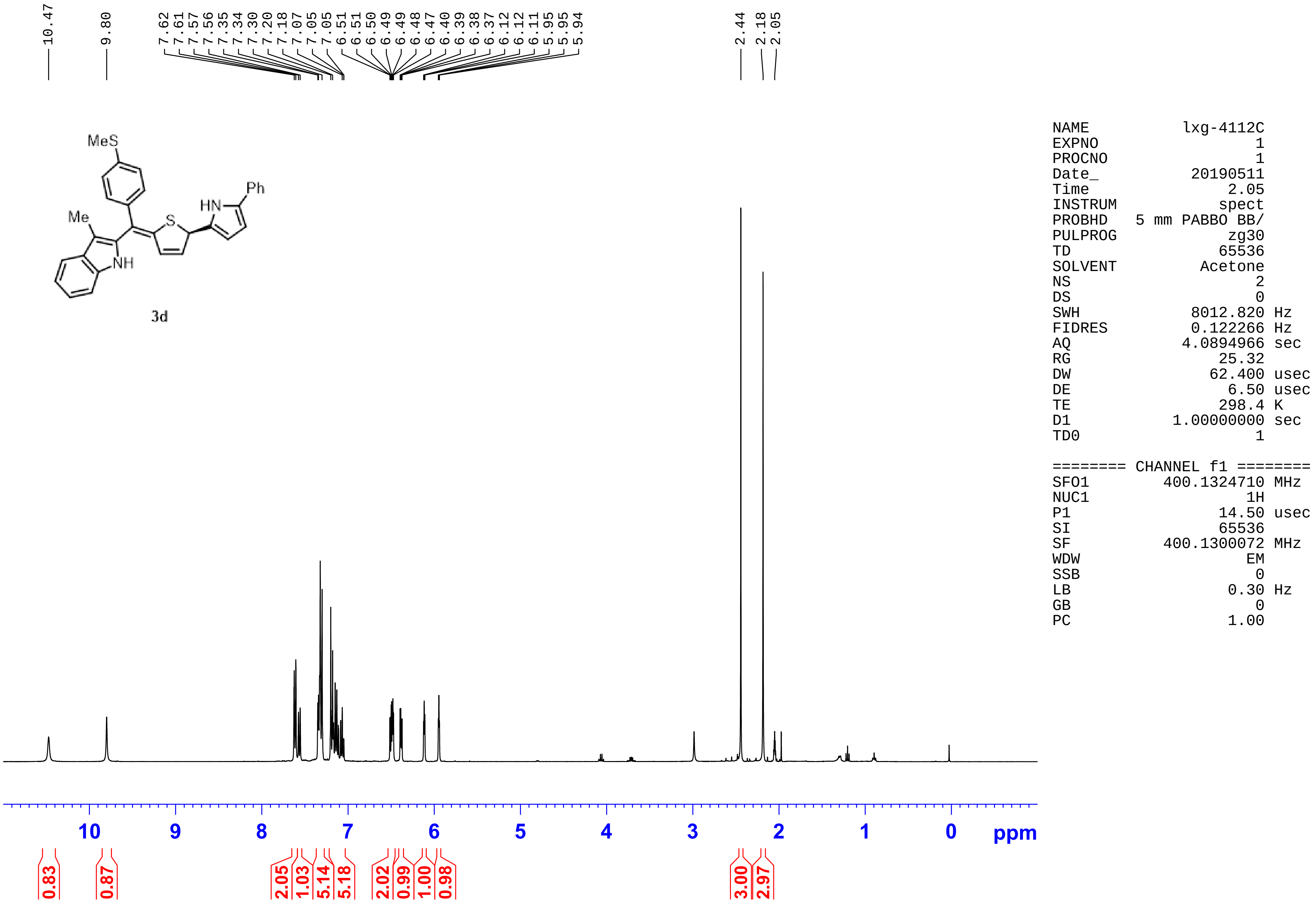

Supplementary Figure 68. ^{1}H NMR spectrum of **3d**

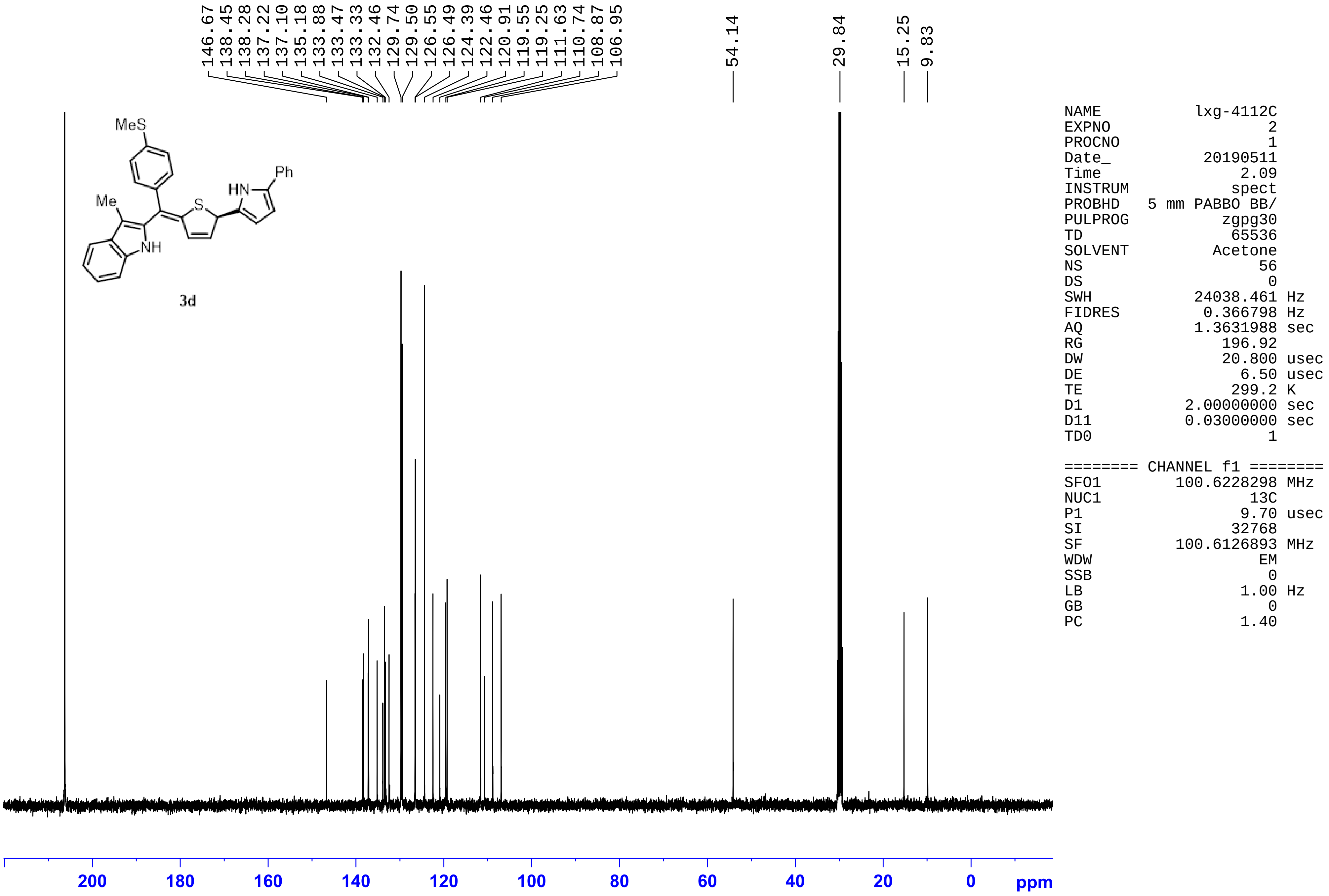

Supplementary Figure 69. ^{13}C NMR spectrum of **3d**

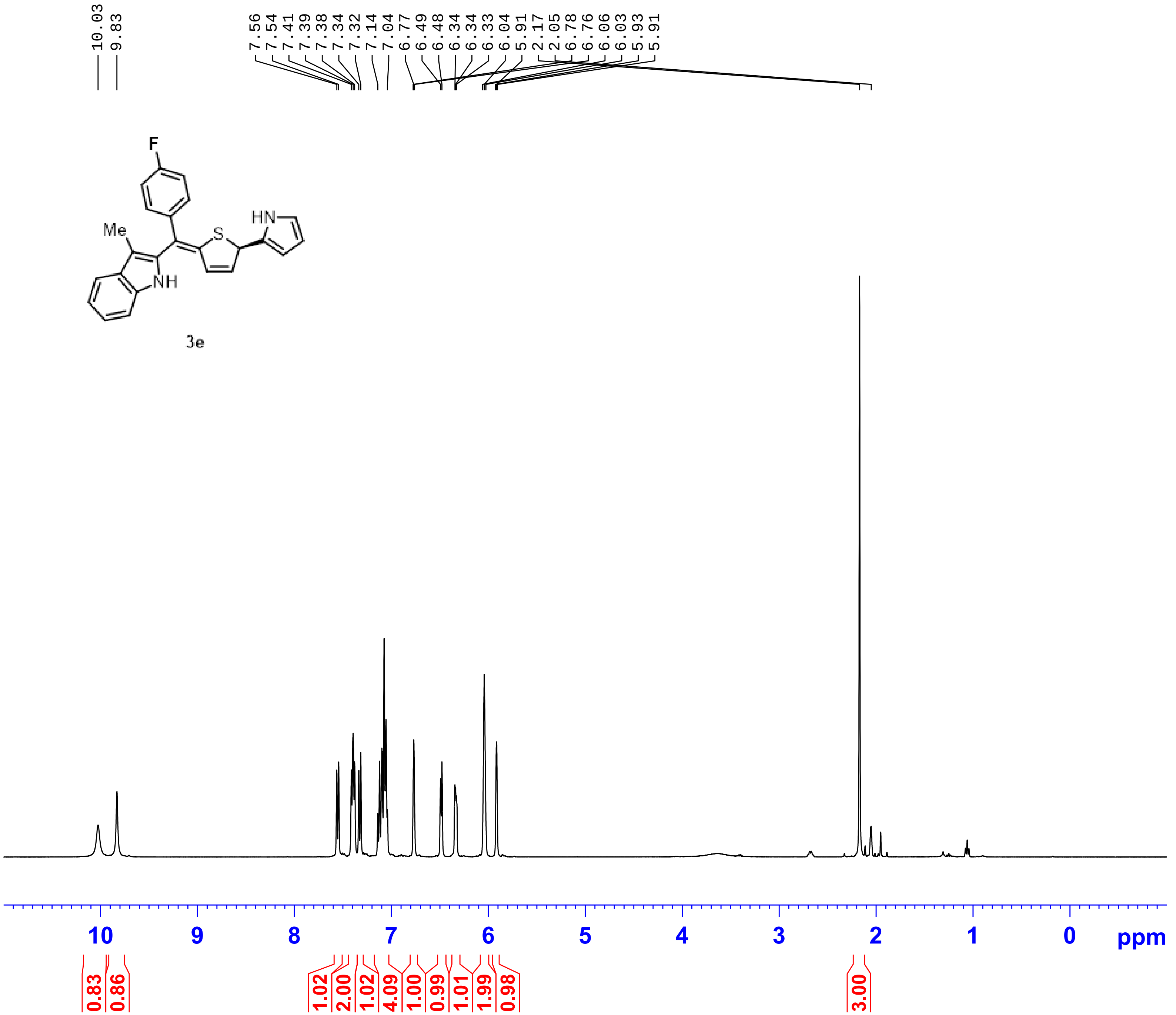

NAME	lxg-7077A
EXPNO	1
PROCNO	1
Date_	20200701
Time	15.26
INSTRUM	spect
PROBHD	5 mm PABBO BB/
PULPROG	zg30
TD	65536
SOLVENT	Acetone
NS	3
DS	0
SWH	8012.820 Hz
FIDRES	0.122266 Hz
AQ	4.0894966 sec
RG	31.55
DW	62.400 usec
DE	6.50 usec
TE	296.7 K
D1	1.00000000 sec
TD0	1

======== CHANNEL f1 ========

SFO1	400.1324710 MHz
NUC1	1H
P1	14.50 usec
SI	65536
SF	400.1300063 MHz
WDW	EM
SSB	0
LB	0.30 Hz
GB	0
PC	1.00

Supplementary Figure 70. ^{1}H NMR spectrum of **3e**

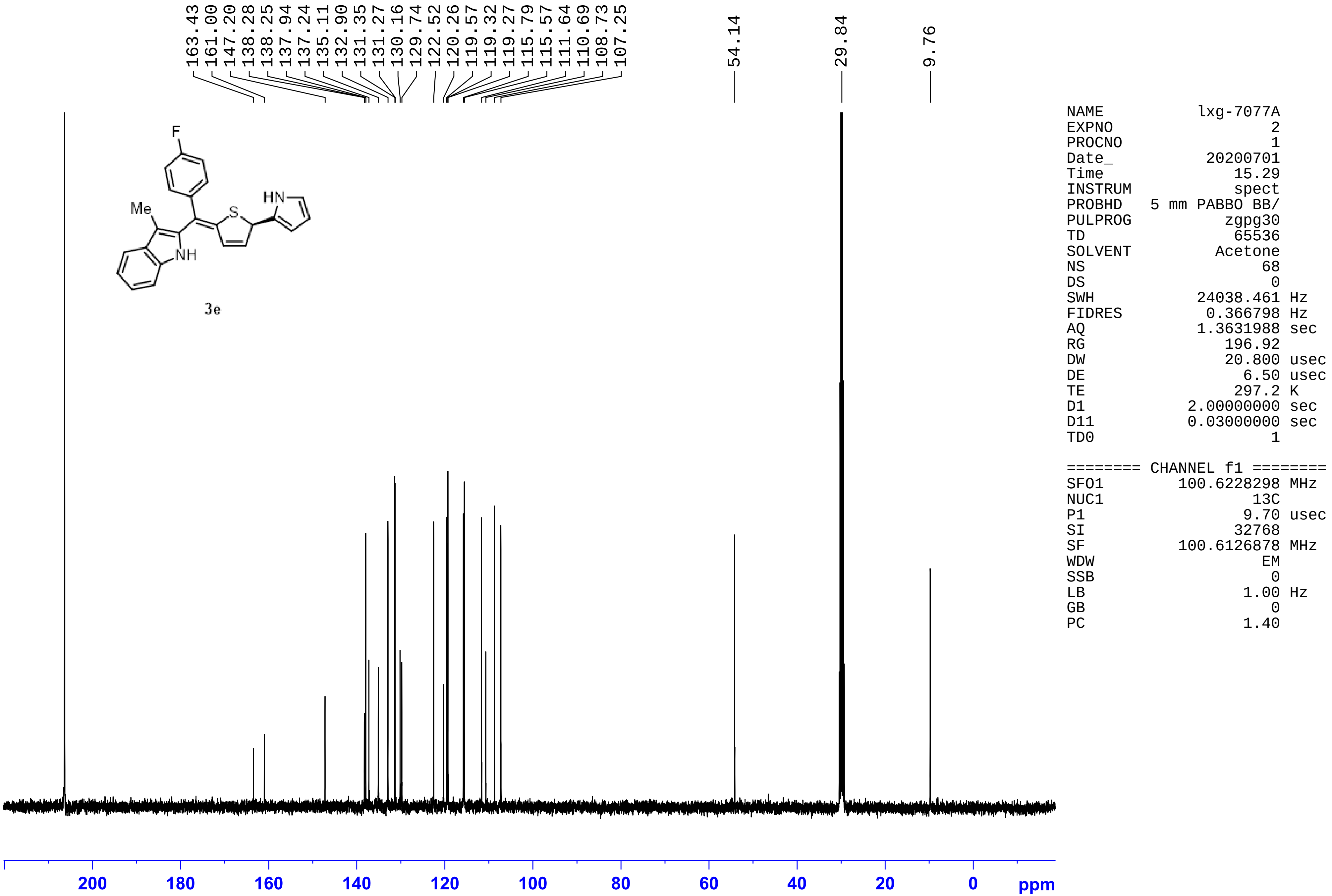

Supplementary Figure 71. ^{13}C NMR spectrum of **3e**

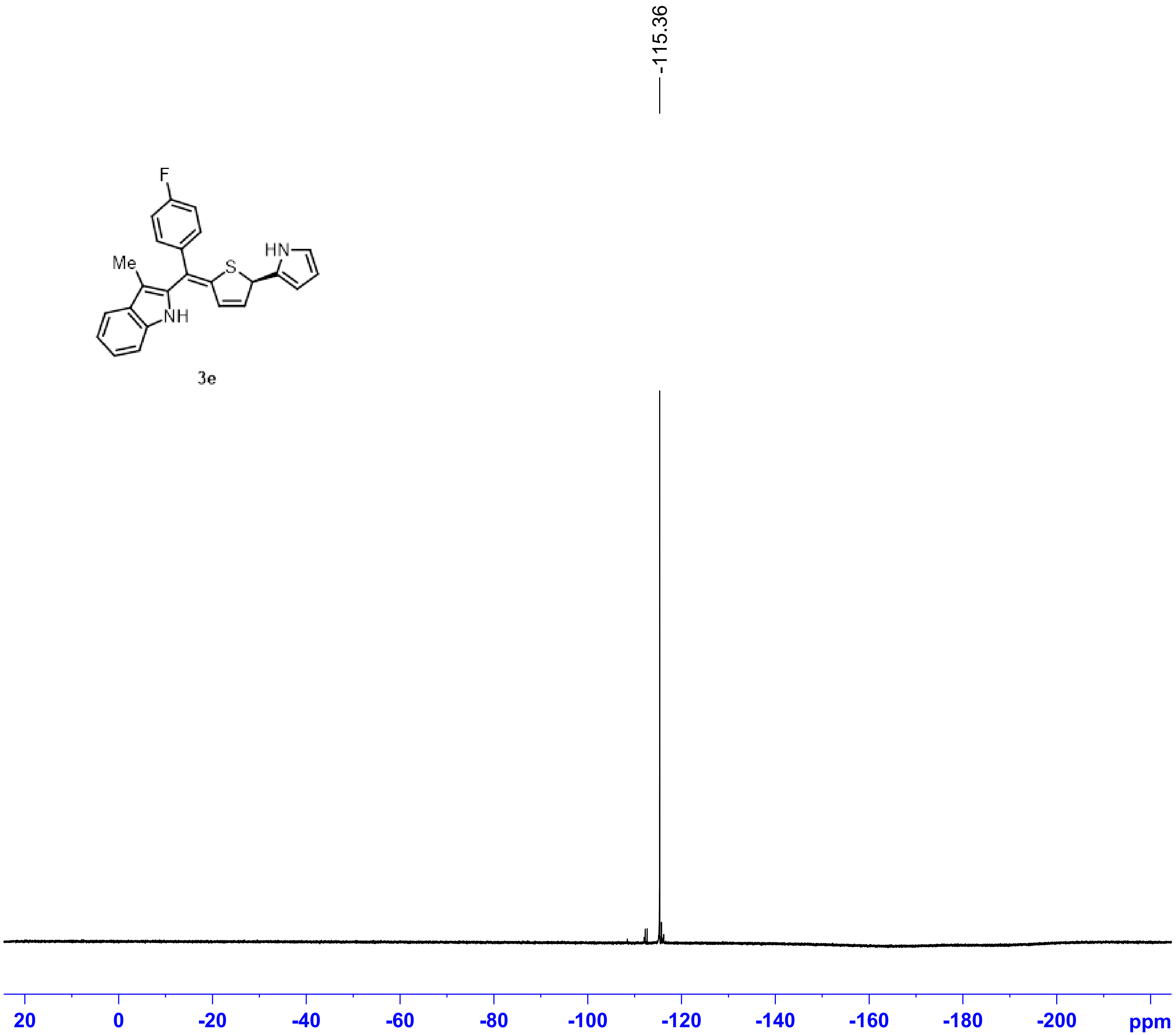

NAME	lxg-7077A
EXPNO	11
PROCNO	1
Date_	20200724
Time	15.02
INSTRUM	spect
PROBHD	5 mm PABBO BB/
PULPROG	zgpg30
TD	65536
SOLVENT	Acetone
NS	13
DS	0
SWH	93750.000 Hz
FIDRES	1.430511 Hz
AQ	0.3495753 sec
RG	196.92
DW	5.333 usec
DE	6.50 usec
TE	296.8 K
D1	2.00000000 sec
D11	0.03000000 sec
TD0	1

======== CHANNEL f1 ========	
SFO1	376.4607162 MHz
NUC1	19F
P1	14.70 usec
SI	32768
SF	376.4983660 MHz
WDW	EM
SSB	0
LB	1.00 Hz
GB	0
PC	1.40

Supplementary Figure 72. ^{19}F NMR spectrum of **3e**

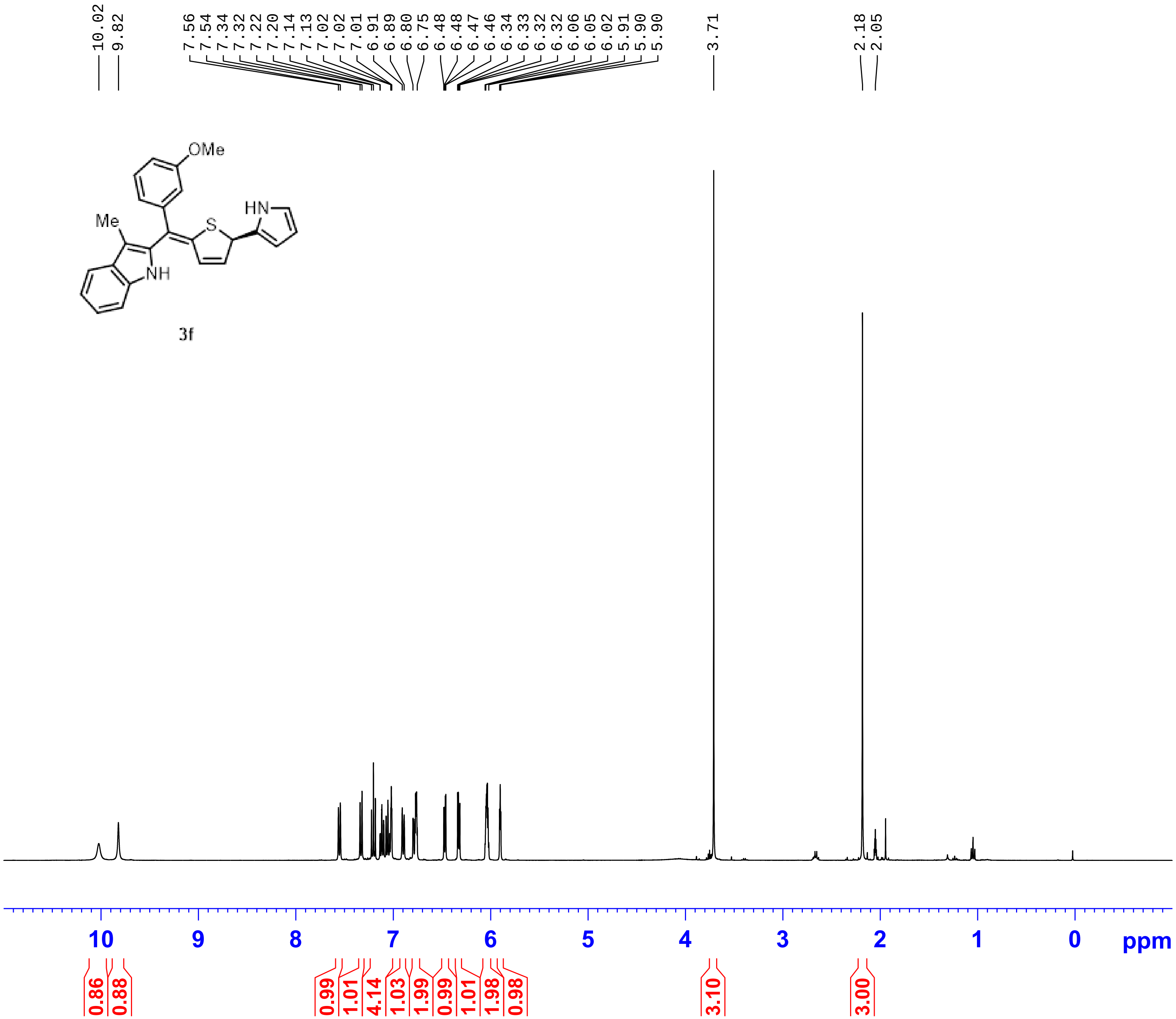

NAME	lxg-7079A
EXPNO	1
PROCNO	1
Date_	20200703
Time	20.02
INSTRUM	spect
PROBHD	5 mm PABBO BB/
PULPROG	zg30
TD	65536
SOLVENT	Acetone
NS	4
DS	0
SWH	8012.820 Hz
FIDRES	0.122266 Hz
AQ	4.0894966 sec
RG	27.78
DW	62.400 usec
DE	6.50 usec
TE	296.7 K
D1	1.00000000 sec
TD0	1

======== CHANNEL f1 ========

SFO1	400.1324710 MHz
NUC1	1H
P1	14.50 usec
SI	65536
SF	400.1300069 MHz
WDW	EM
SSB	0
LB	0.30 Hz
GB	0
PC	1.00

Supplementary Figure 73. ^{1}H NMR spectrum of **3f**

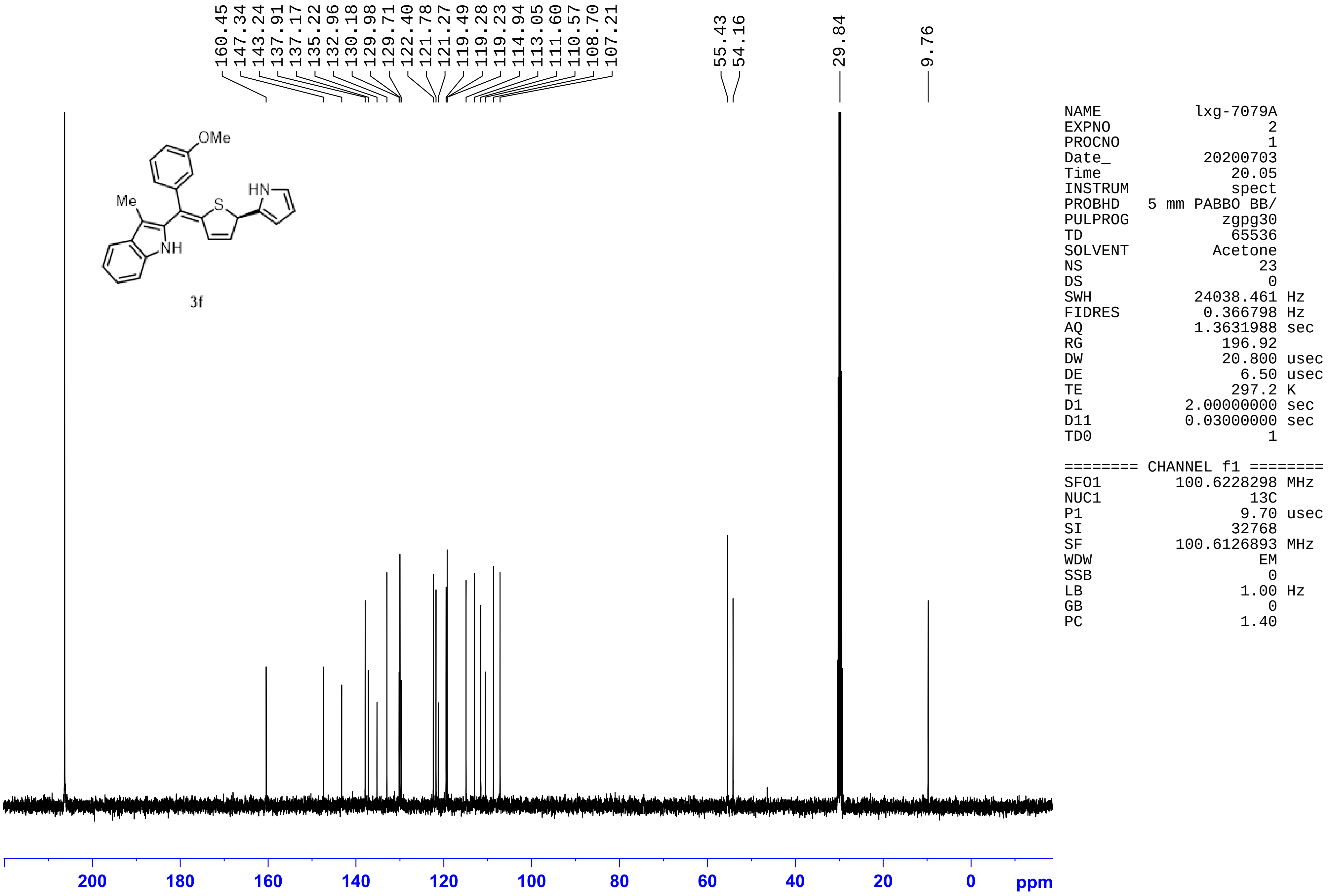

Supplementary Figure 74. ^{13}C NMR spectrum of **3f**

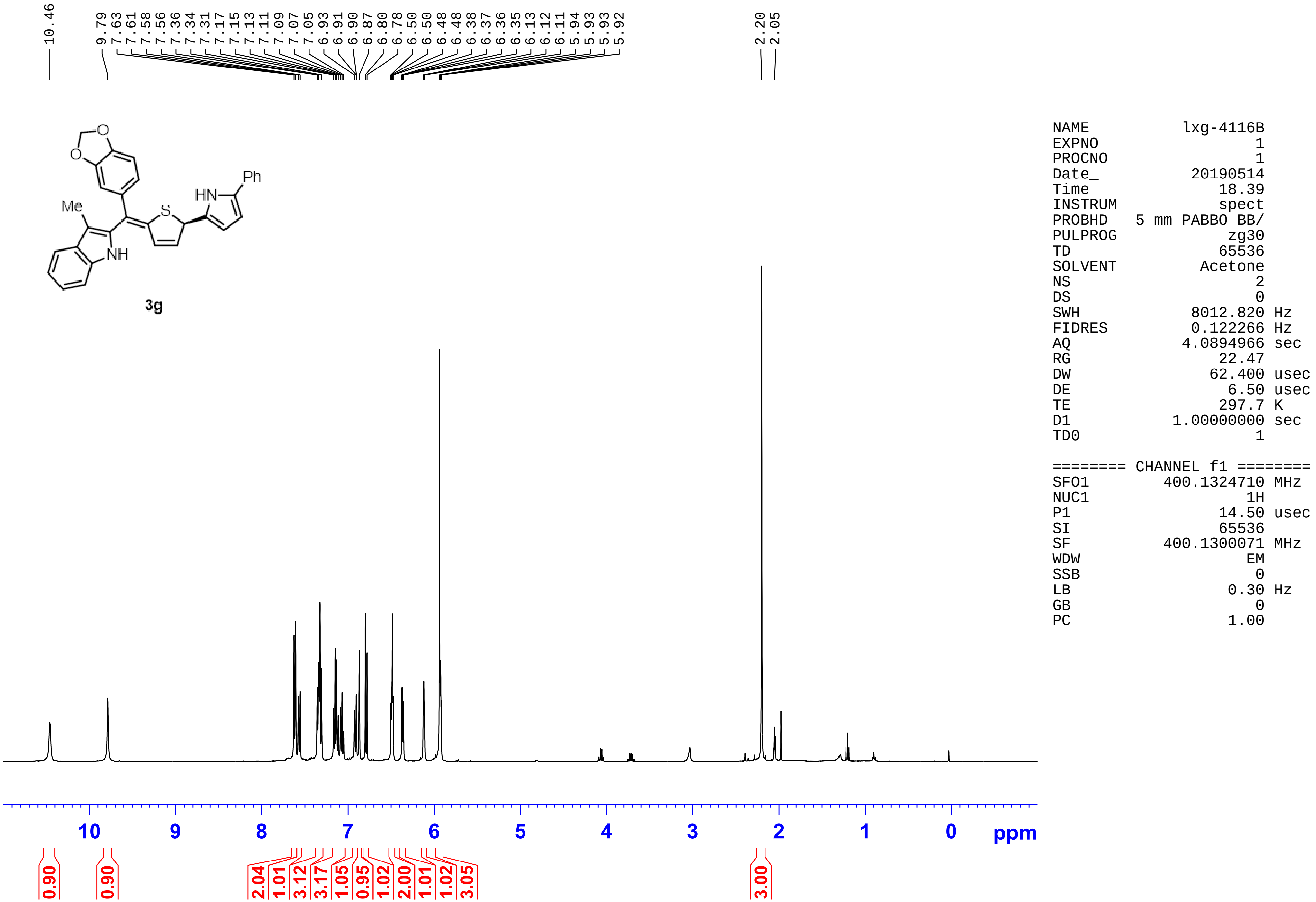

Supplementary Figure 75. ^{1}H NMR spectrum of **3g**

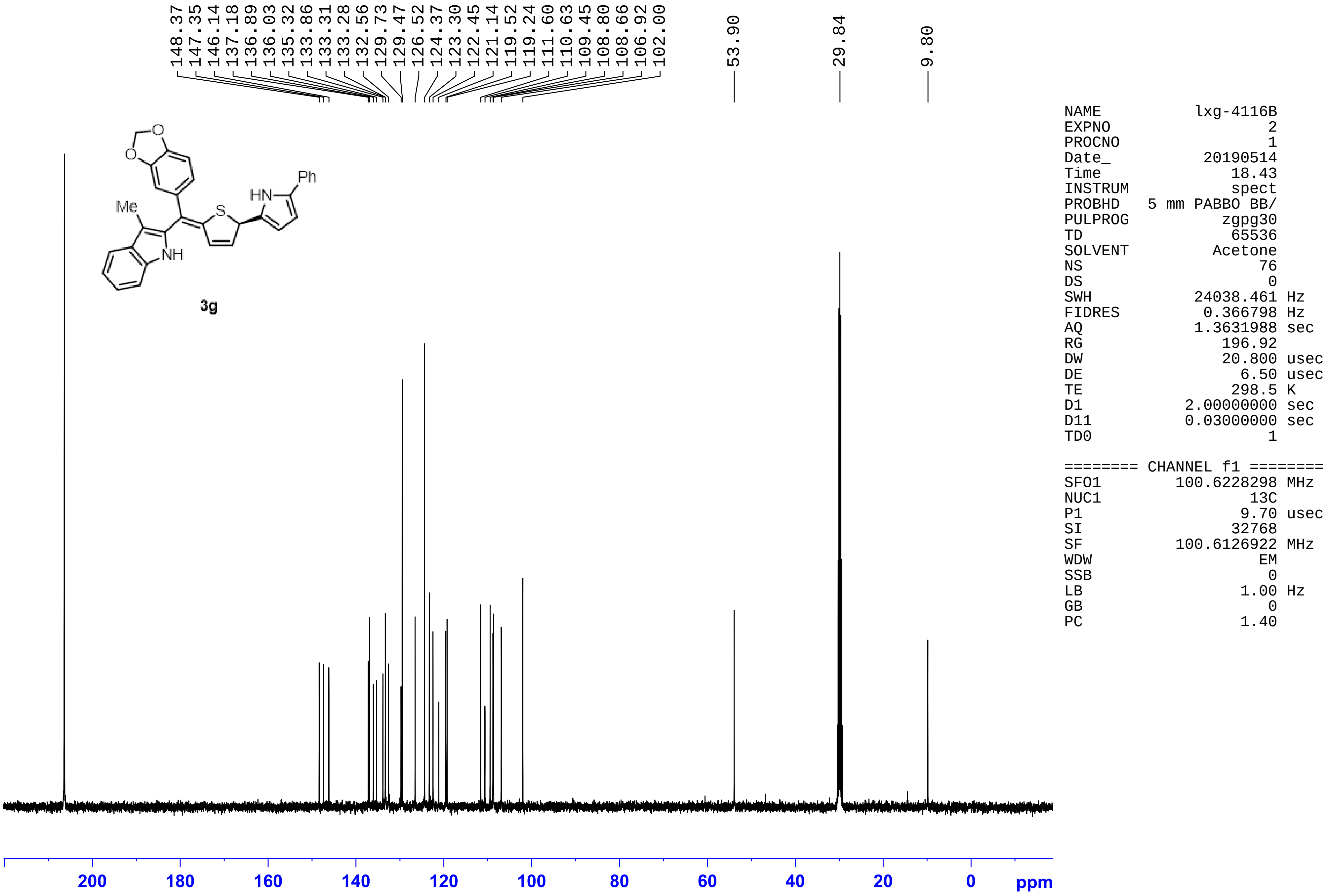

Supplementary Figure 76. ^{13}C NMR spectrum of **3g**

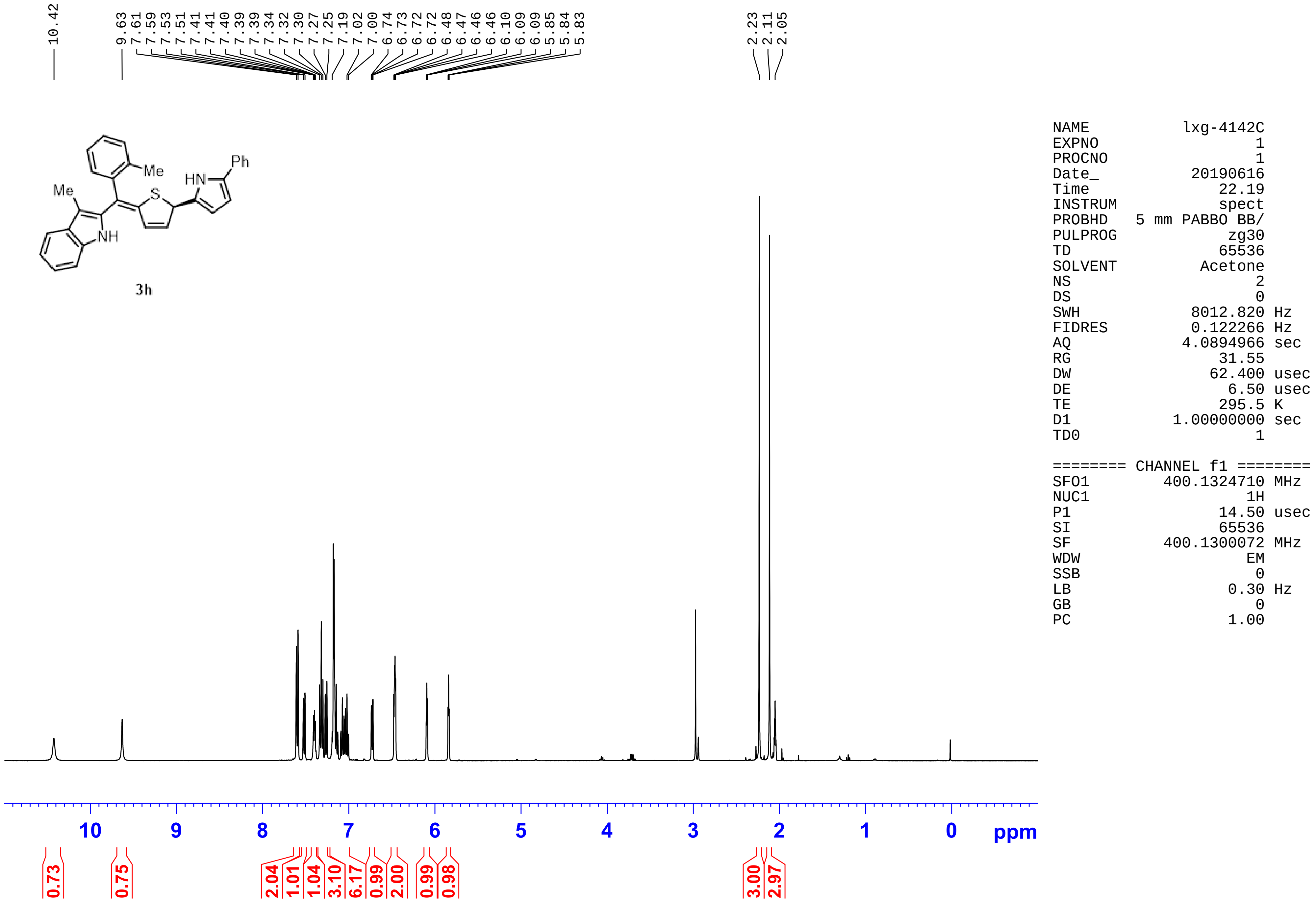

Supplementary Figure 77. ^{1}H NMR spectrum of **3h**

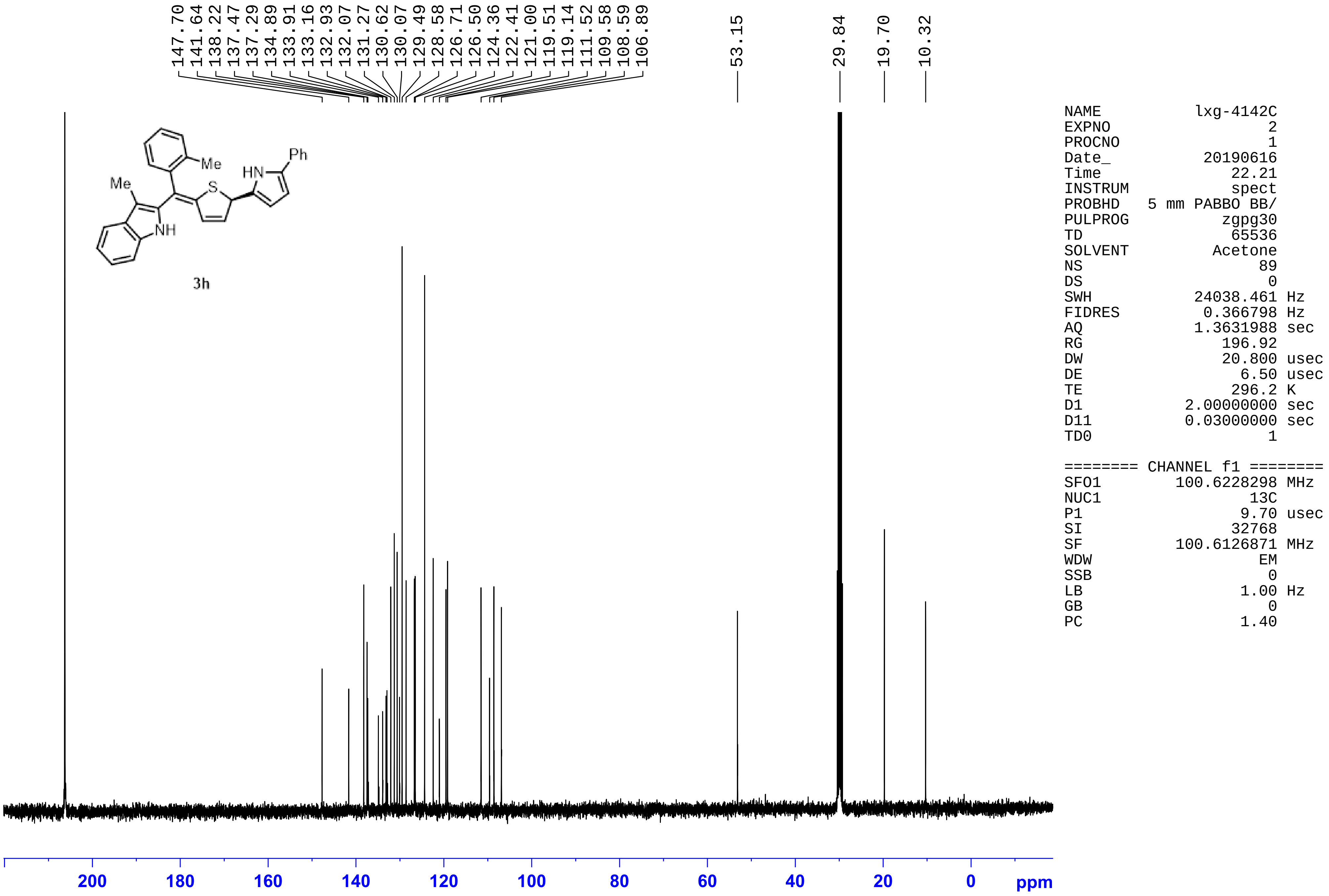

Supplementary Figure 78. ^{13}C NMR spectrum of **3h**

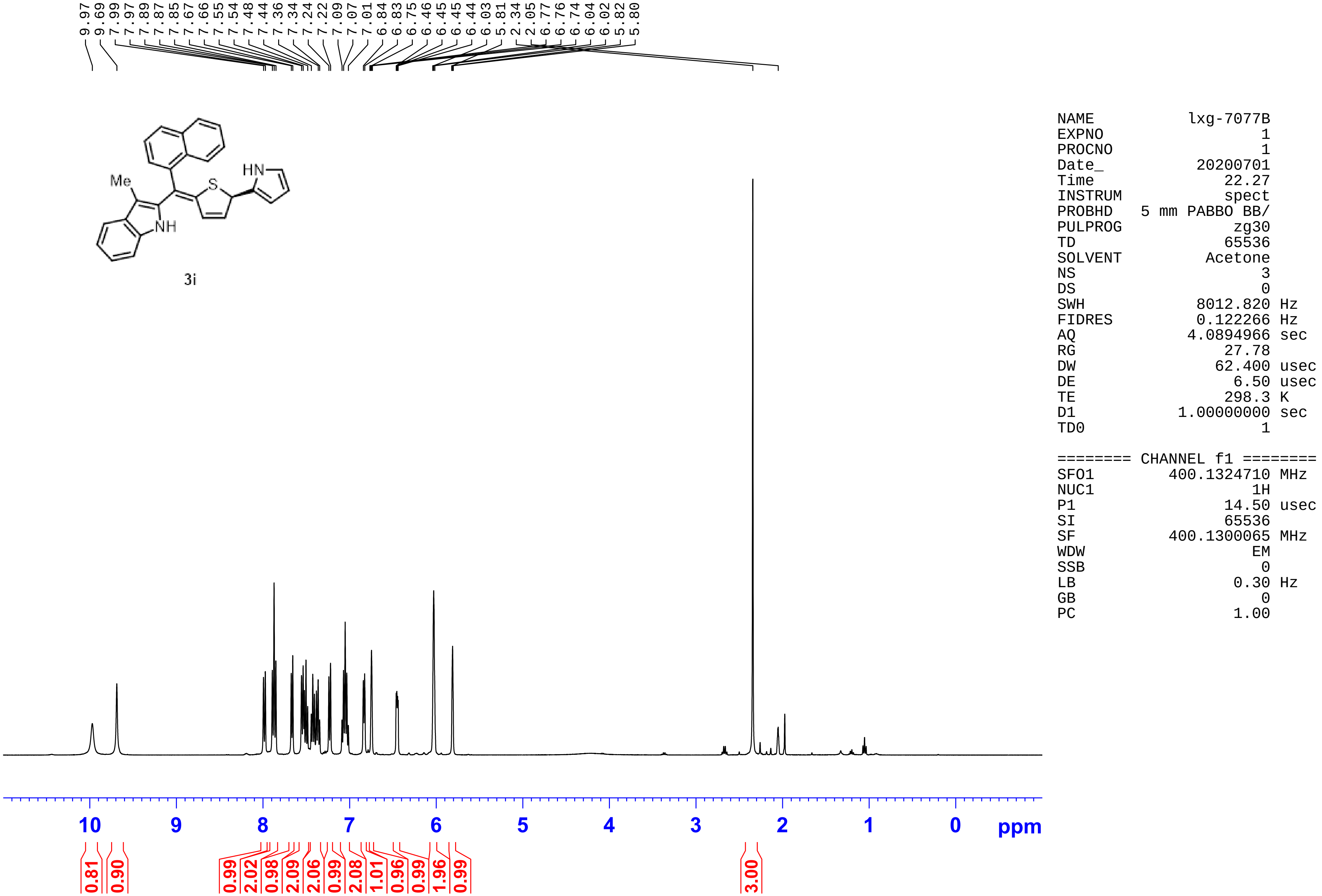

Supplementary Figure 79. ^{1}H NMR spectrum of **3l**

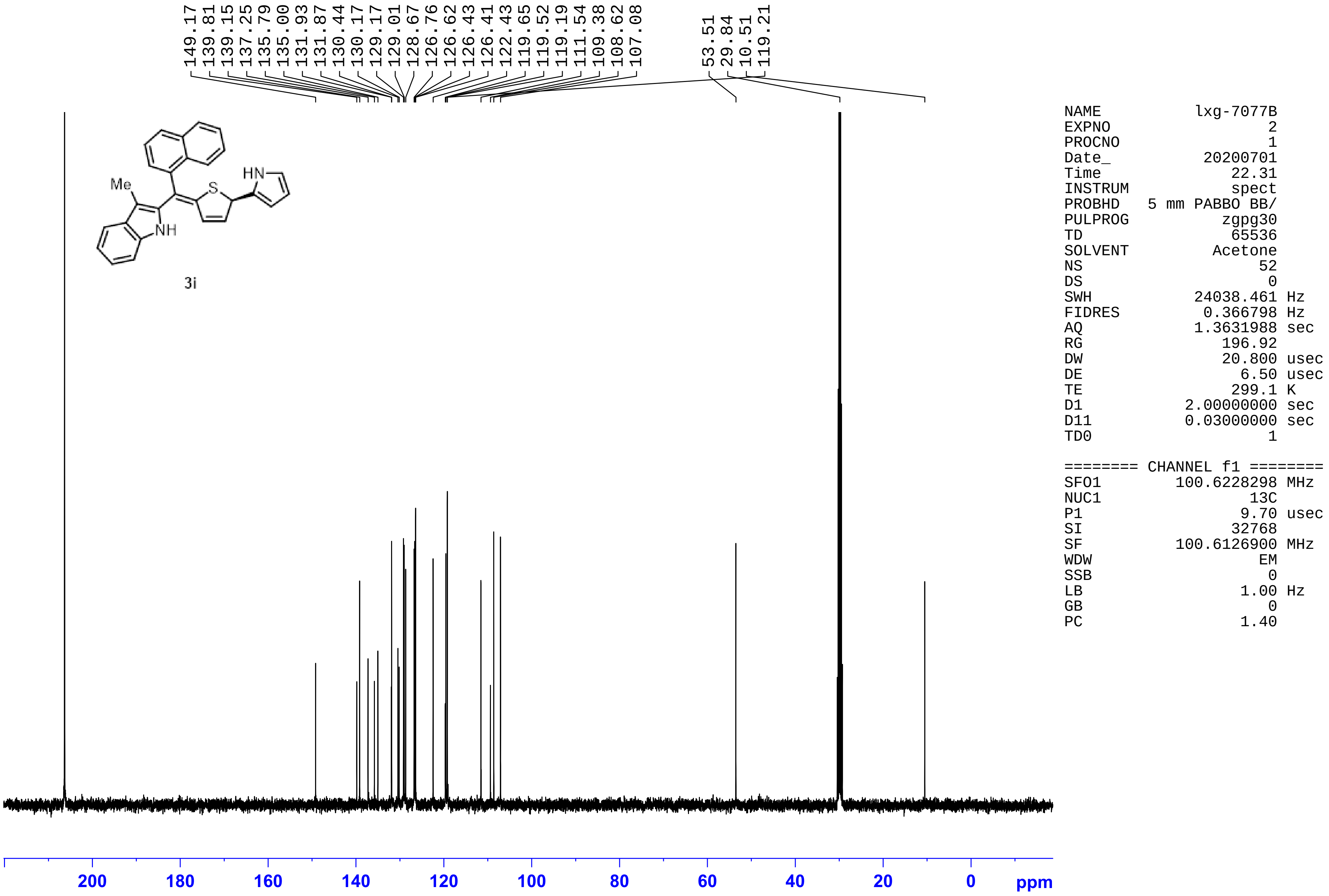

Supplementary Figure 80. ^{13}C NMR spectrum of **3l**

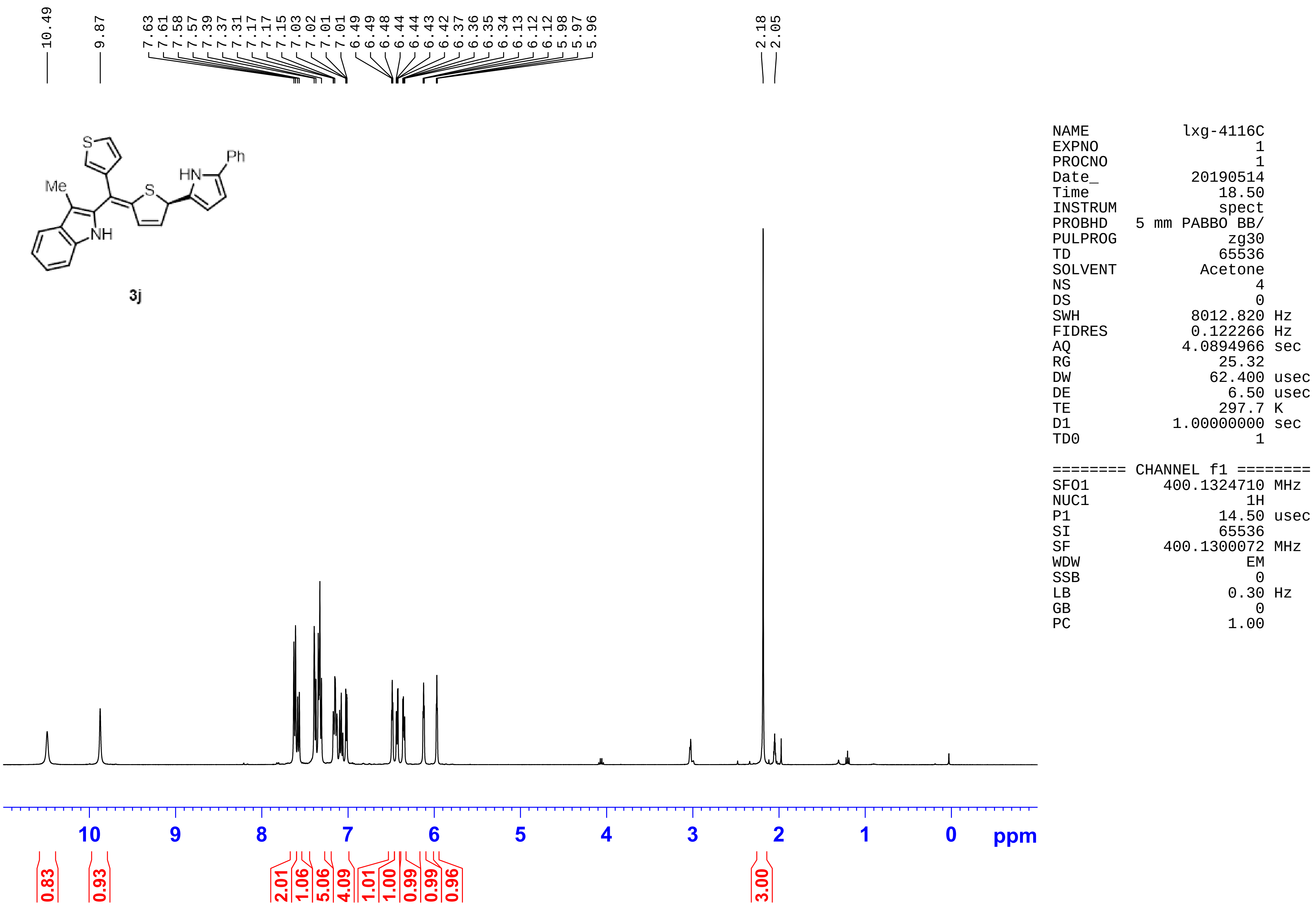

Supplementary Figure 81. ^{1}H NMR spectrum of **3j**

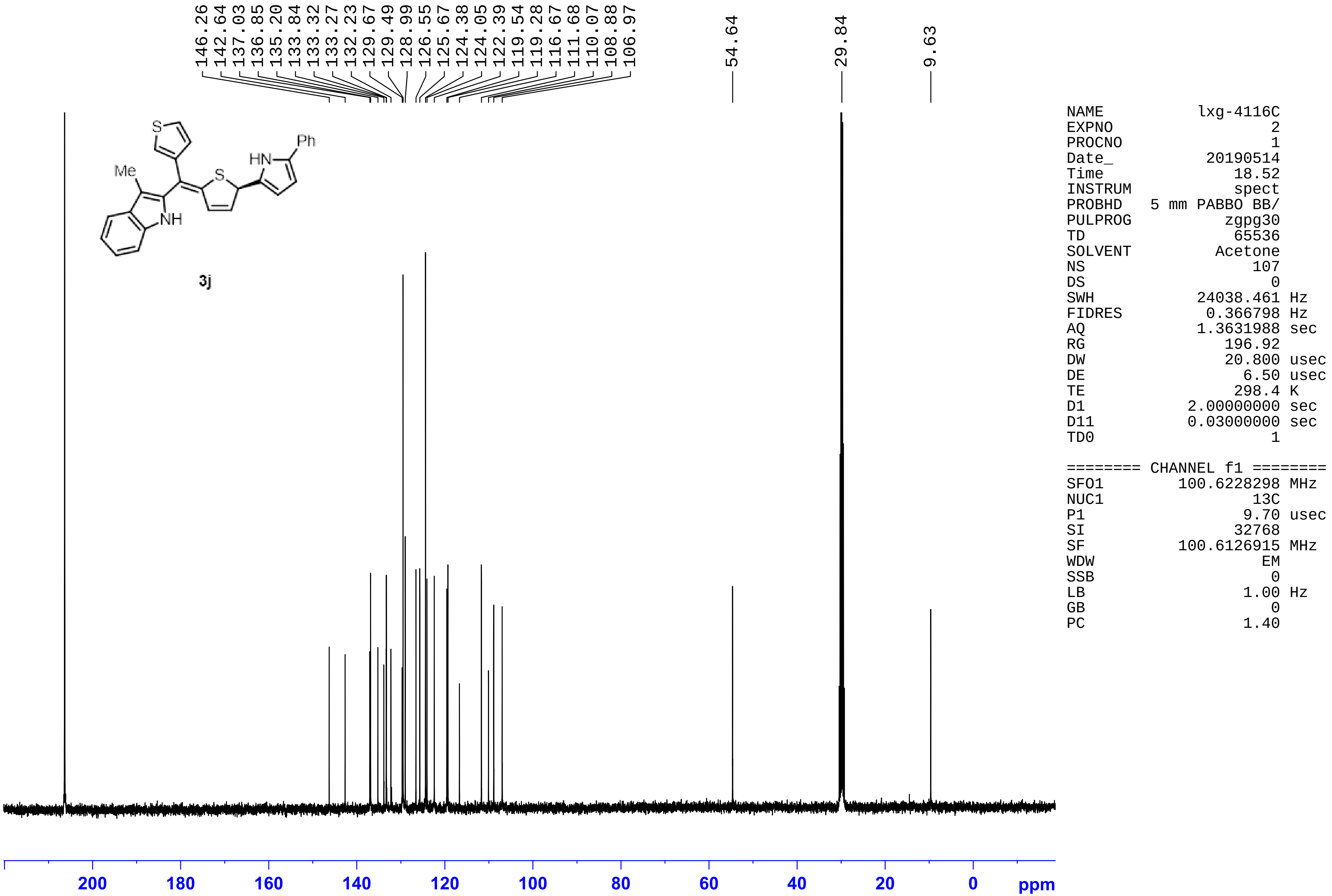

Supplementary Figure 82. ^{13}C NMR spectrum of **3j**

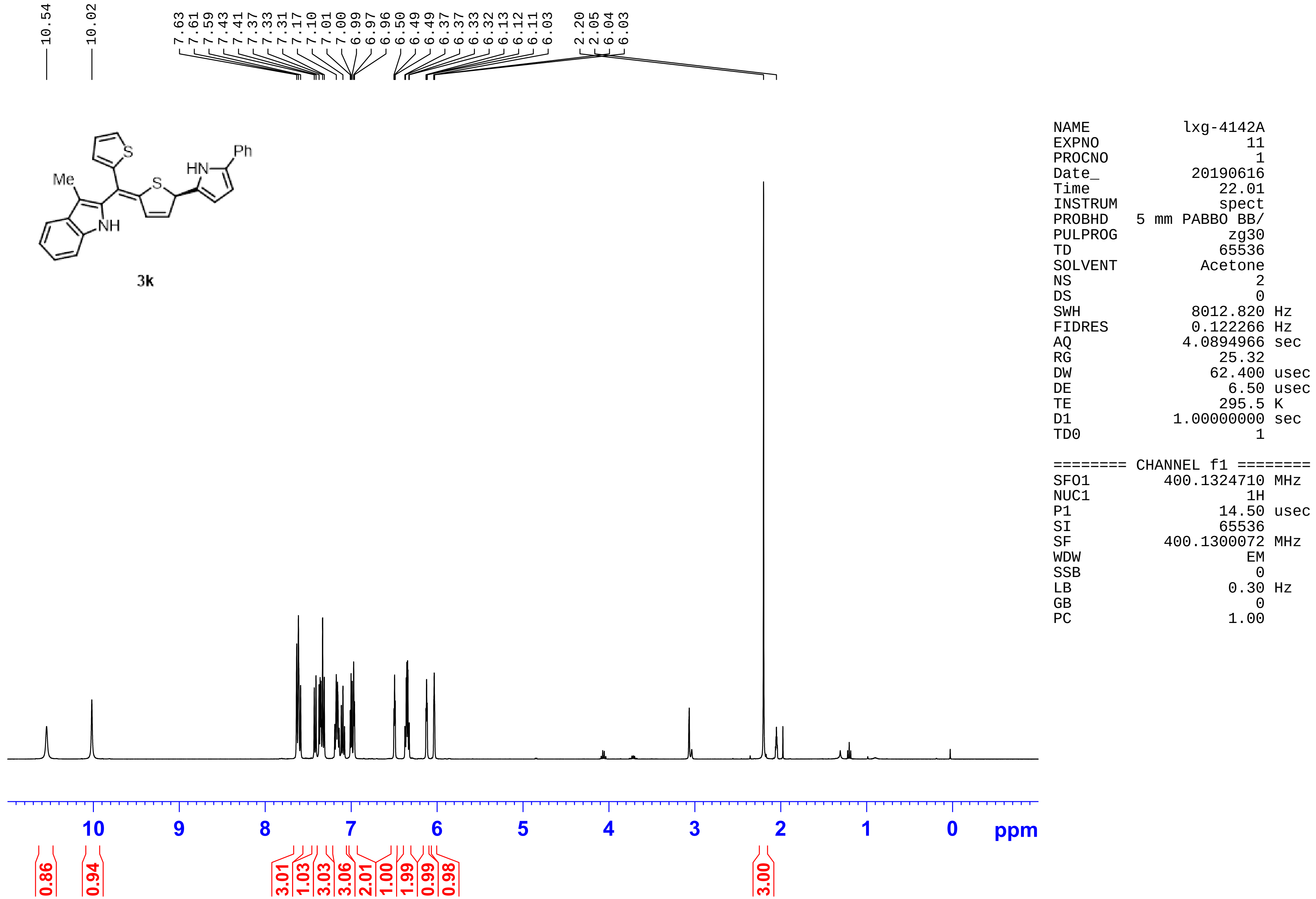

Supplementary Figure 83. ^{1}H NMR spectrum of **3k**

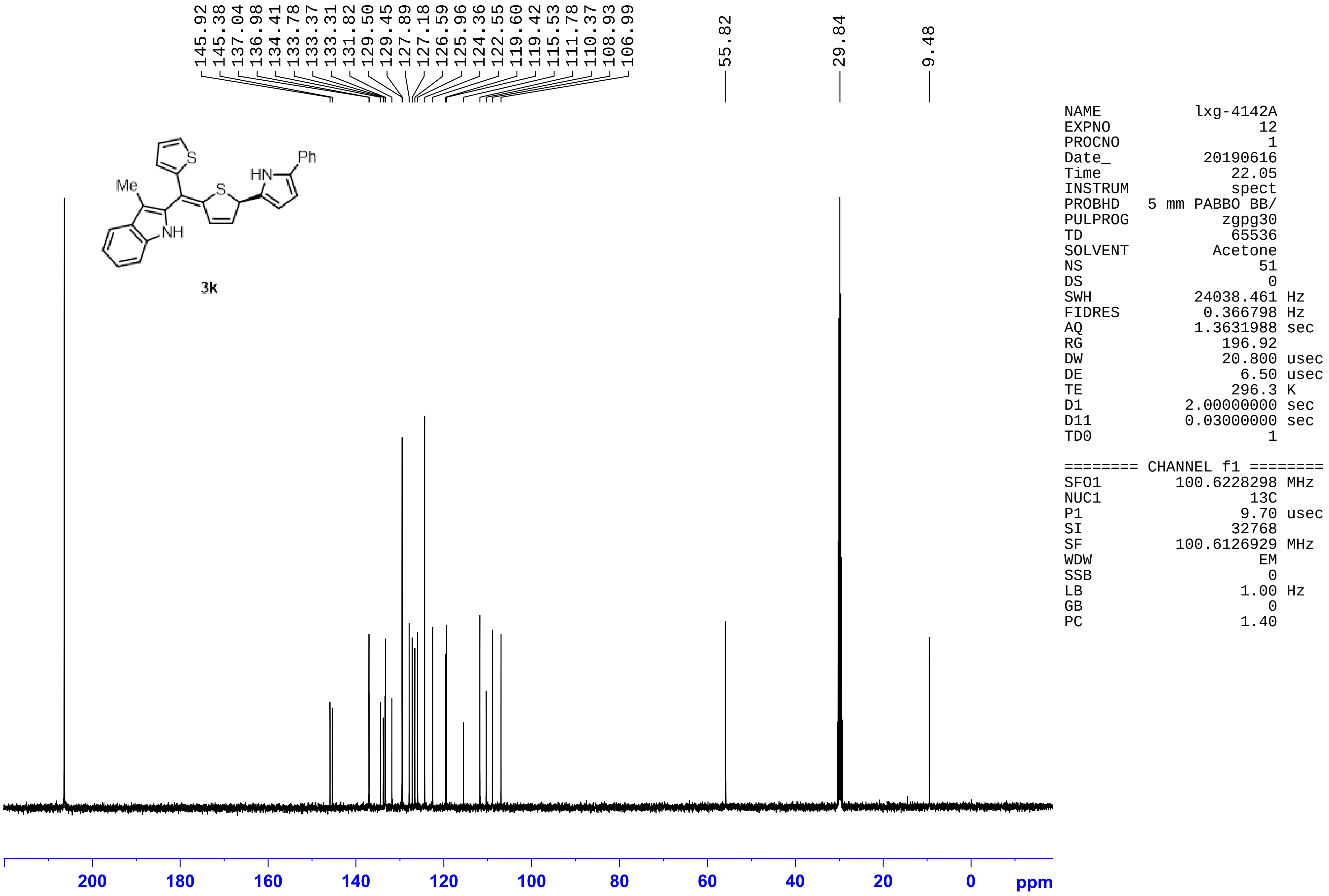

Supplementary Figure 84. ^{13}C NMR spectrum of **3k**

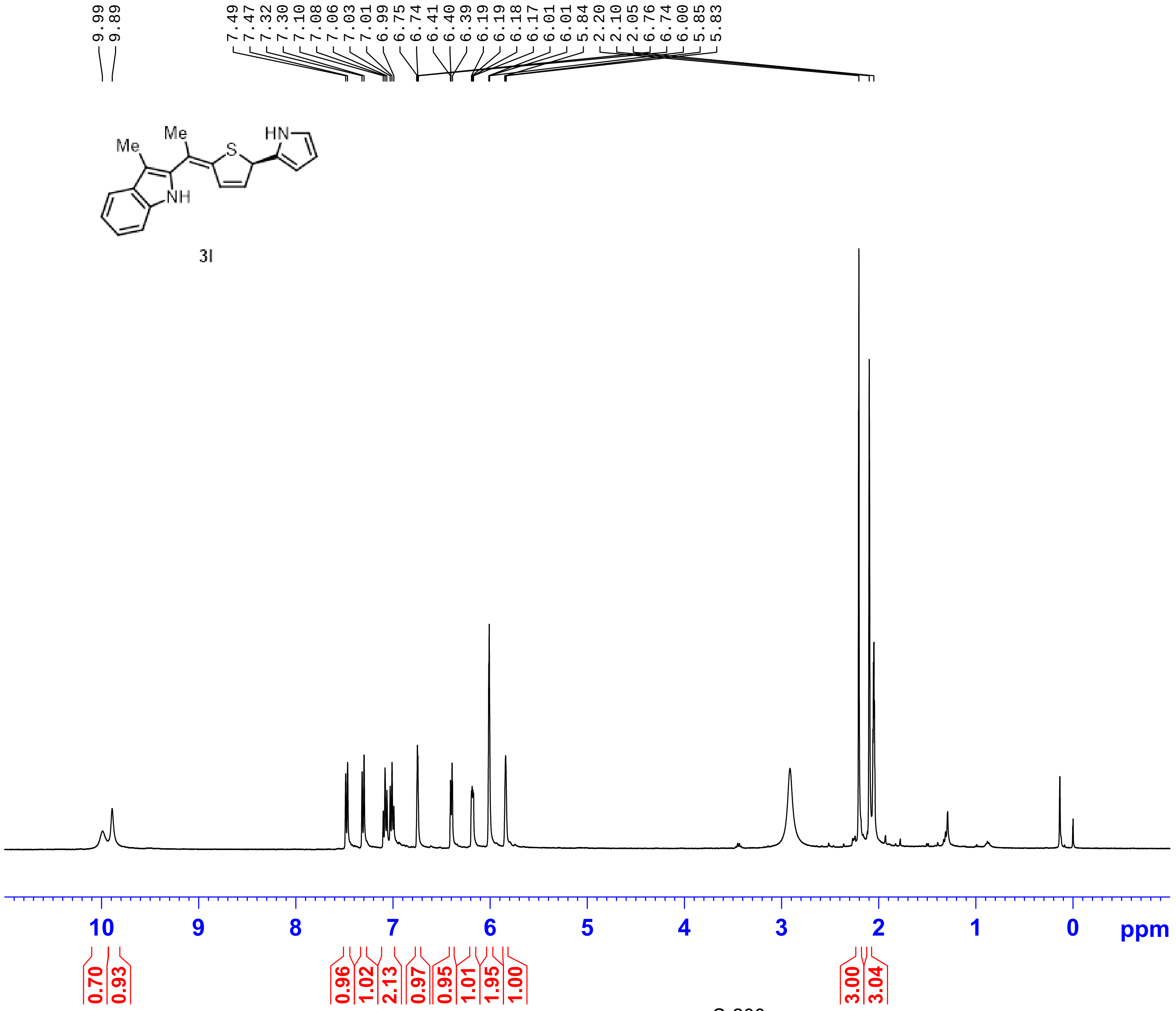

3l

NAME	lxg-7090B3
EXPNO	1
PROCNO	1
Date_	20200716
Time	10.53
INSTRUM	spect
PROBHD	5 mm PABBO BB/
PULPROG	zg30
TD	65536
SOLVENT	Acetone
NS	5
DS	0
SWH	8012.820 Hz
FIDRES	0.122266 Hz
AQ	4.0894966 sec
RG	112.31
DW	62.400 usec
DE	6.50 usec
TE	297.2 K
D1	1.00000000 sec
TD0	1

======== CHANNEL f1 ========

SFO1	400.1324710 MHz
NUC1	1H
P1	14.50 usec
SI	65536
SF	400.1300066 MHz
WDW	EM
SSB	0
LB	0.30 Hz
GB	0
PC	1.00

Supplementary Figure 85. ^{1}H NMR spectrum of **3l**

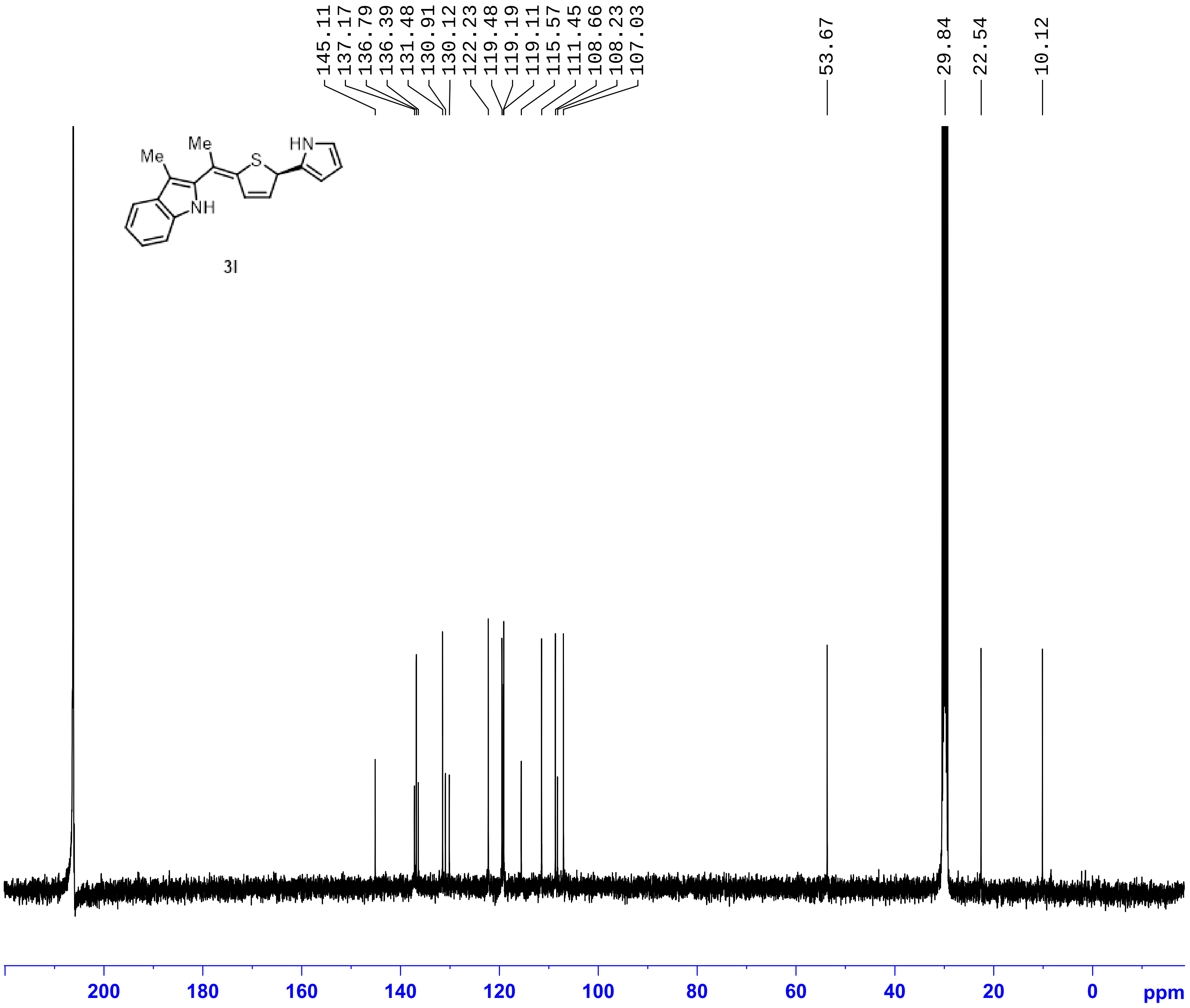

NAME	lxg-7090B3	
EXPNO	2	
PROCNO	1	
Date_	20200716	
Time	10.57	
INSTRUM	spect	
PROBHD	5 mm PABBO BB/	
PULPROG	zgpg30	
TD	65536	
SOLVENT	Acetone	
NS	895	
DS	0	
SWH	24038.461	Hz
FIDRES	0.366798	Hz
AQ	1.3631988	sec
RG	196.92	
DW	20.800	usec
DE	6.50	usec
TE	297.6	K
D1	2.00000000	sec
D11	0.03000000	sec
TD0	1	

======== CHANNEL f1 ========

SFO1	100.6228298	MHz
NUC1	13C	
P1	9.70	usec
SI	32768	
SF	100.6126790	MHz
WDW	EM	
SSB	0	
LB	1.00	Hz
GB	0	
PC	1.40	

Supplementary Figure 86. ^{13}C NMR spectrum of **3l**

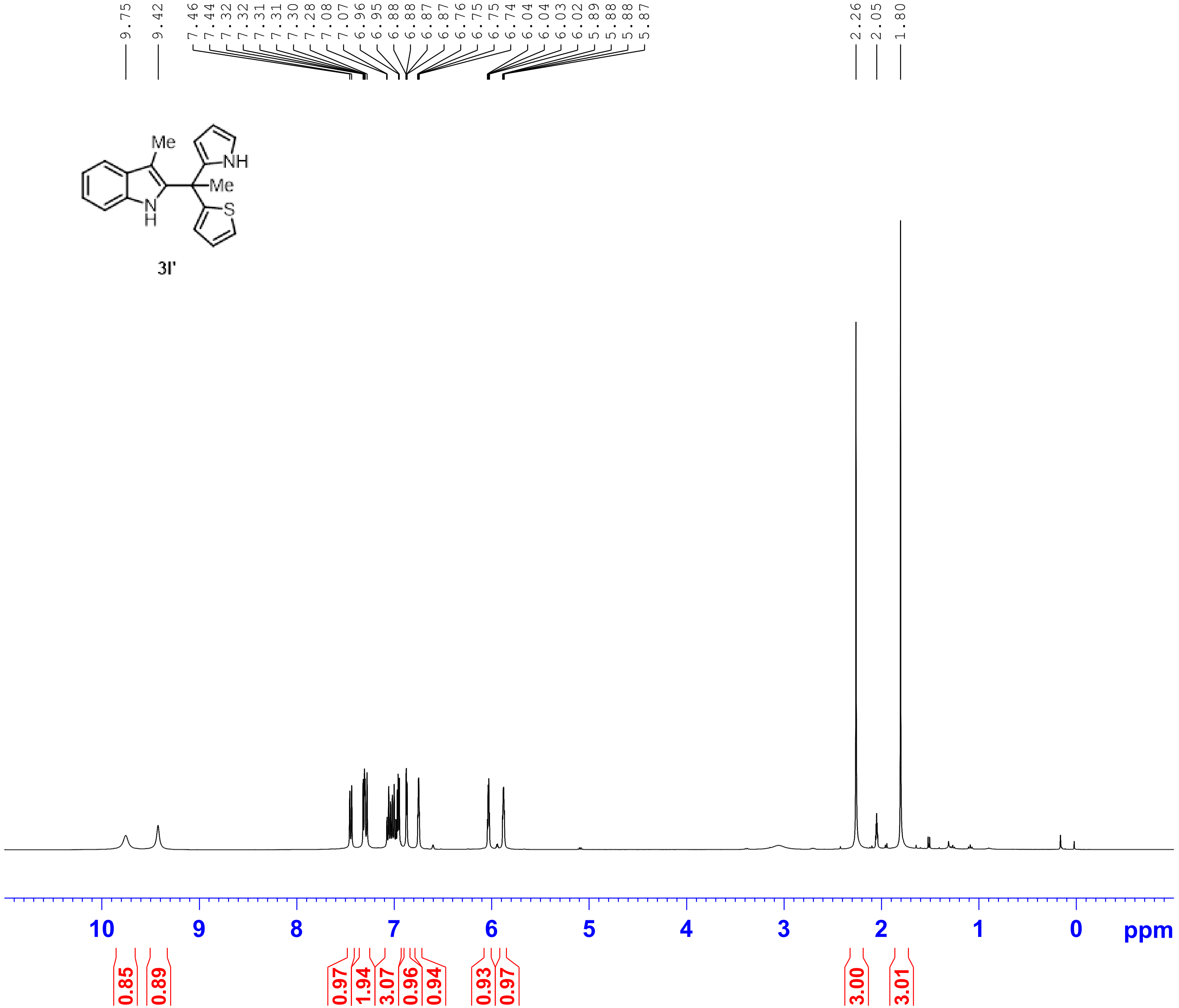

NAME	lxg-7090B2
EXPNO	1
PROCNO	1
Date_	20200716
Time	10.43
INSTRUM	spect
PROBHD	5 mm PABBO BB/
PULPROG	zg30
TD	65536
SOLVENT	Acetone
NS	4
DS	0
SWH	8012.820 Hz
FIDRES	0.122266 Hz
AQ	4.0894966 sec
RG	34.77
DW	62.400 usec
DE	6.50 usec
TE	297.1 K
D1	1.00000000 sec
TD0	1

======== CHANNEL f1 ========

SFO1	400.1324710 MHz
NUC1	1H
P1	14.50 usec
SI	65536
SF	400.1300070 MHz
WDW	EM
SSB	0
LB	0.30 Hz
GB	0
PC	1.00

Supplementary Figure 87. ^{1}H NMR spectrum of **3l'**

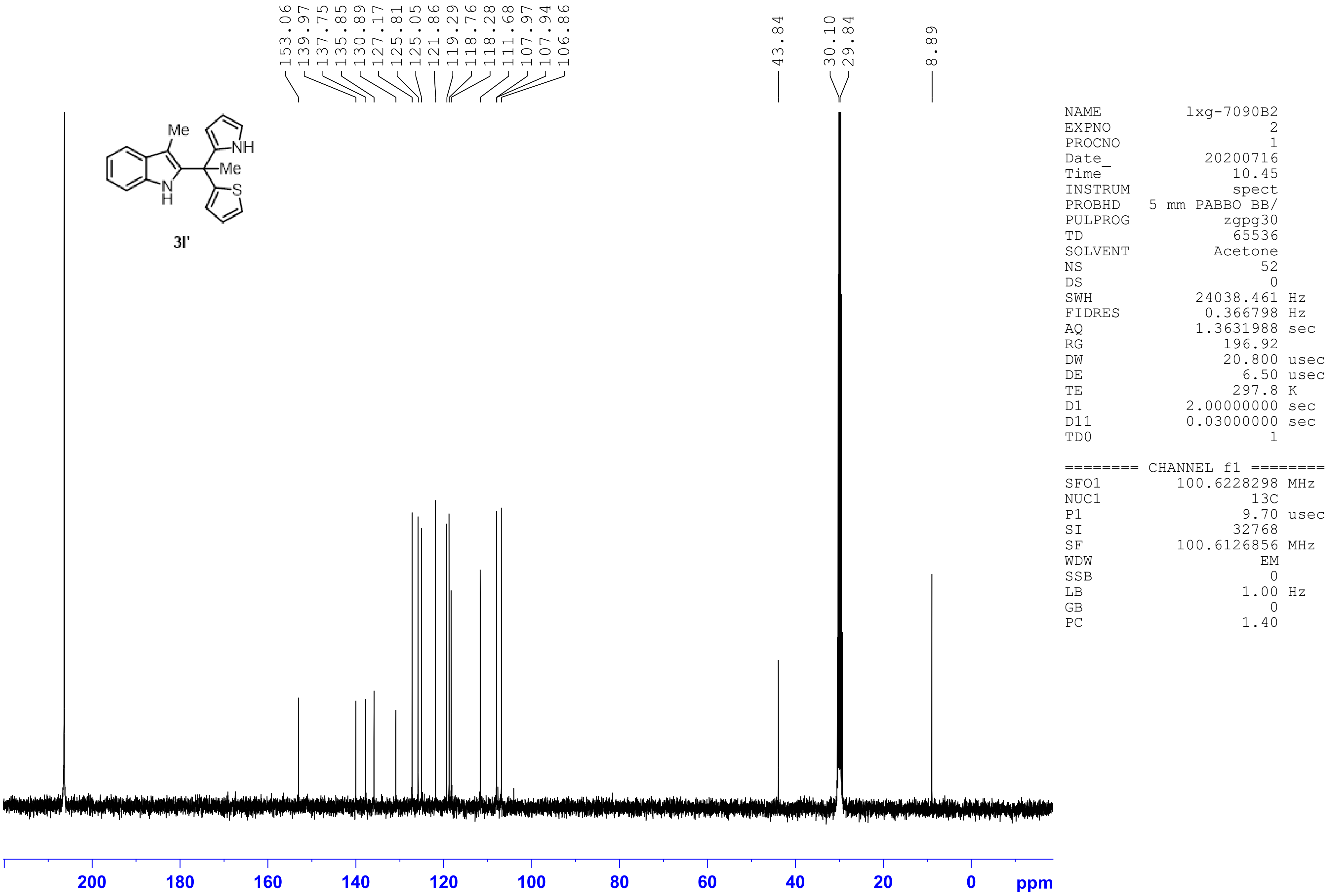

Supplementary Figure 88. ^{13}C NMR spectrum of **3l'**

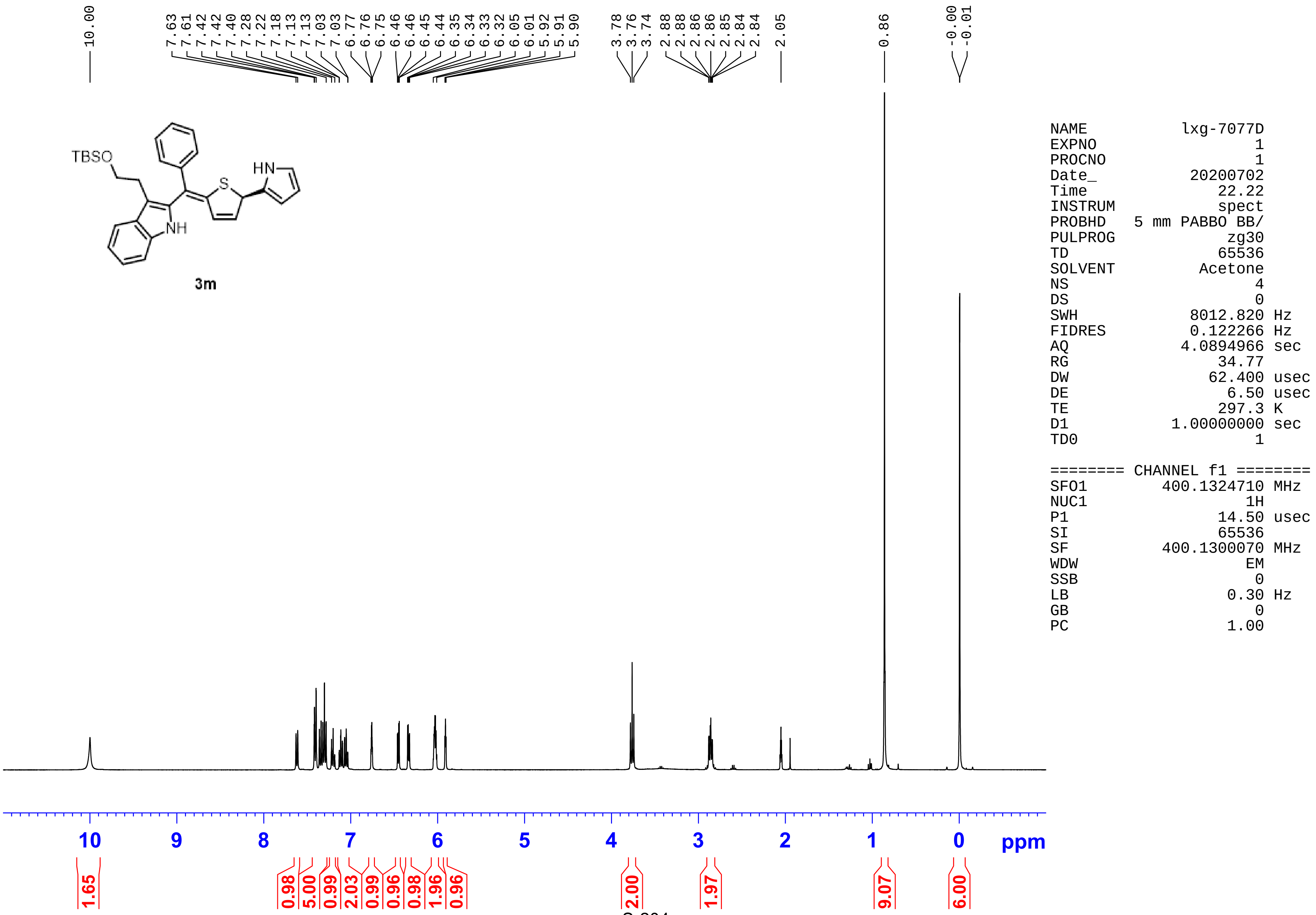

Supplementary Figure 89. ^{1}H NMR spectrum of **3m**

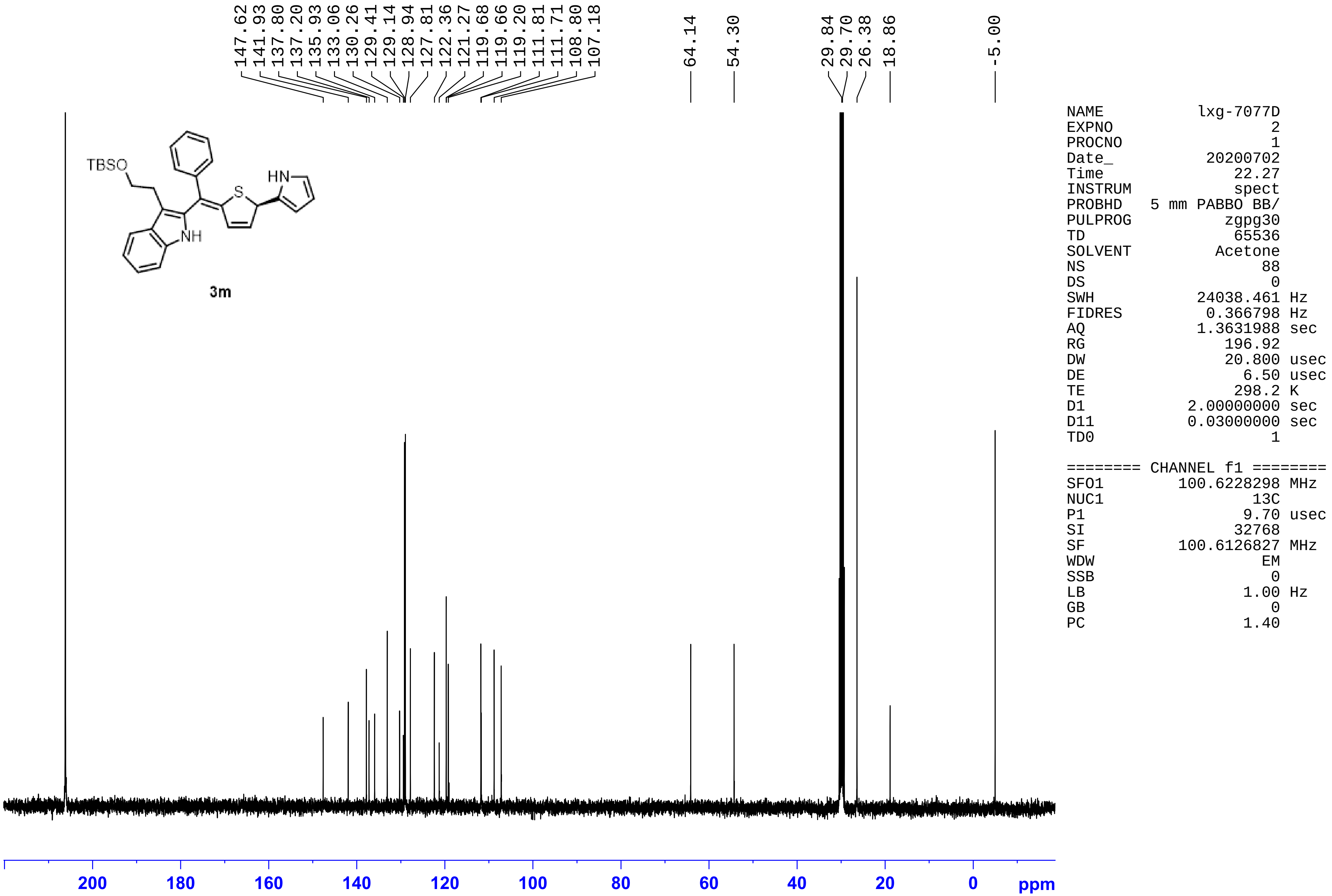

Supplementary Figure 90. ^{1}H NMR spectrum of **3m**

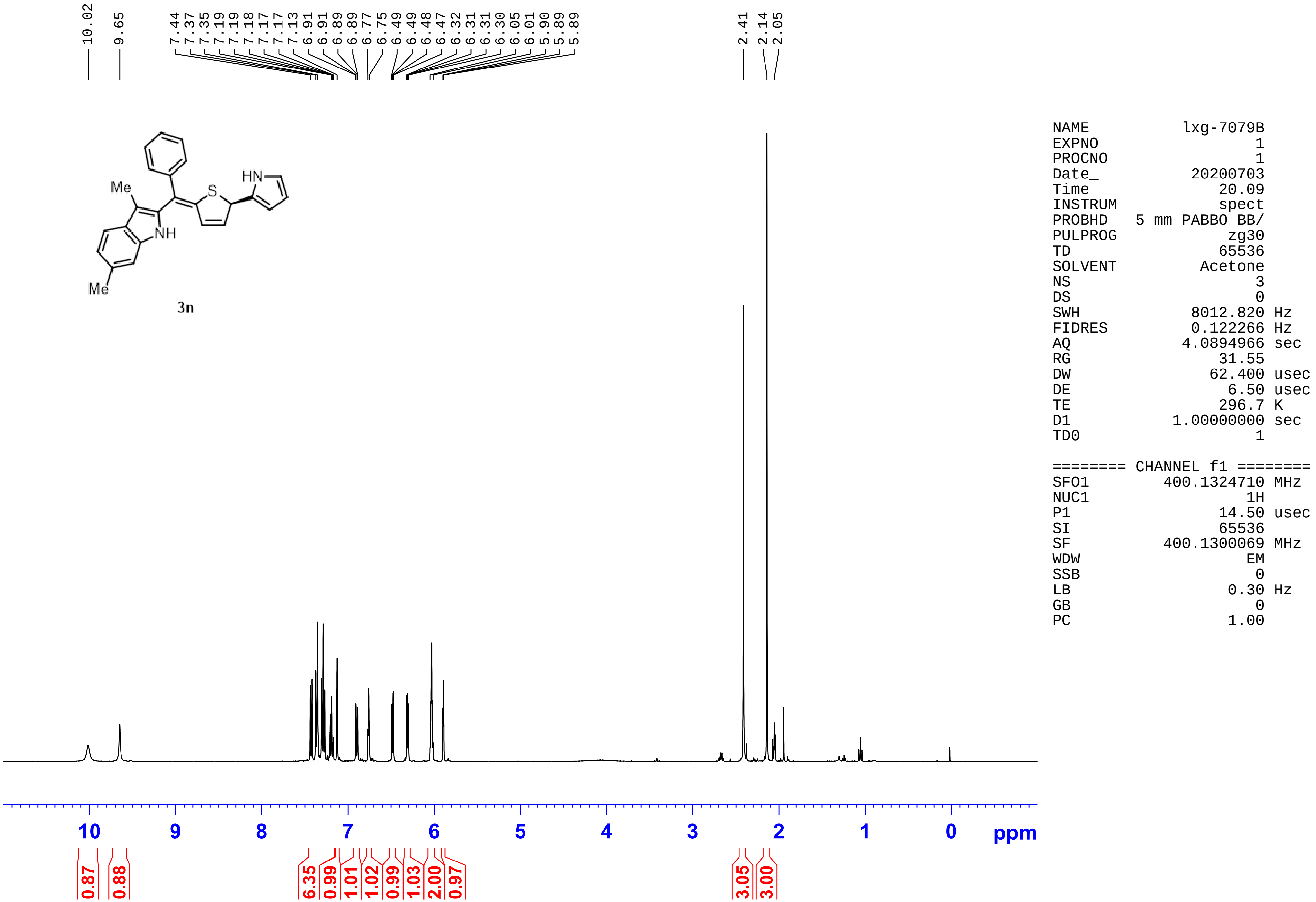

Supplementary Figure 91. ^{1}H NMR spectrum of **3n**

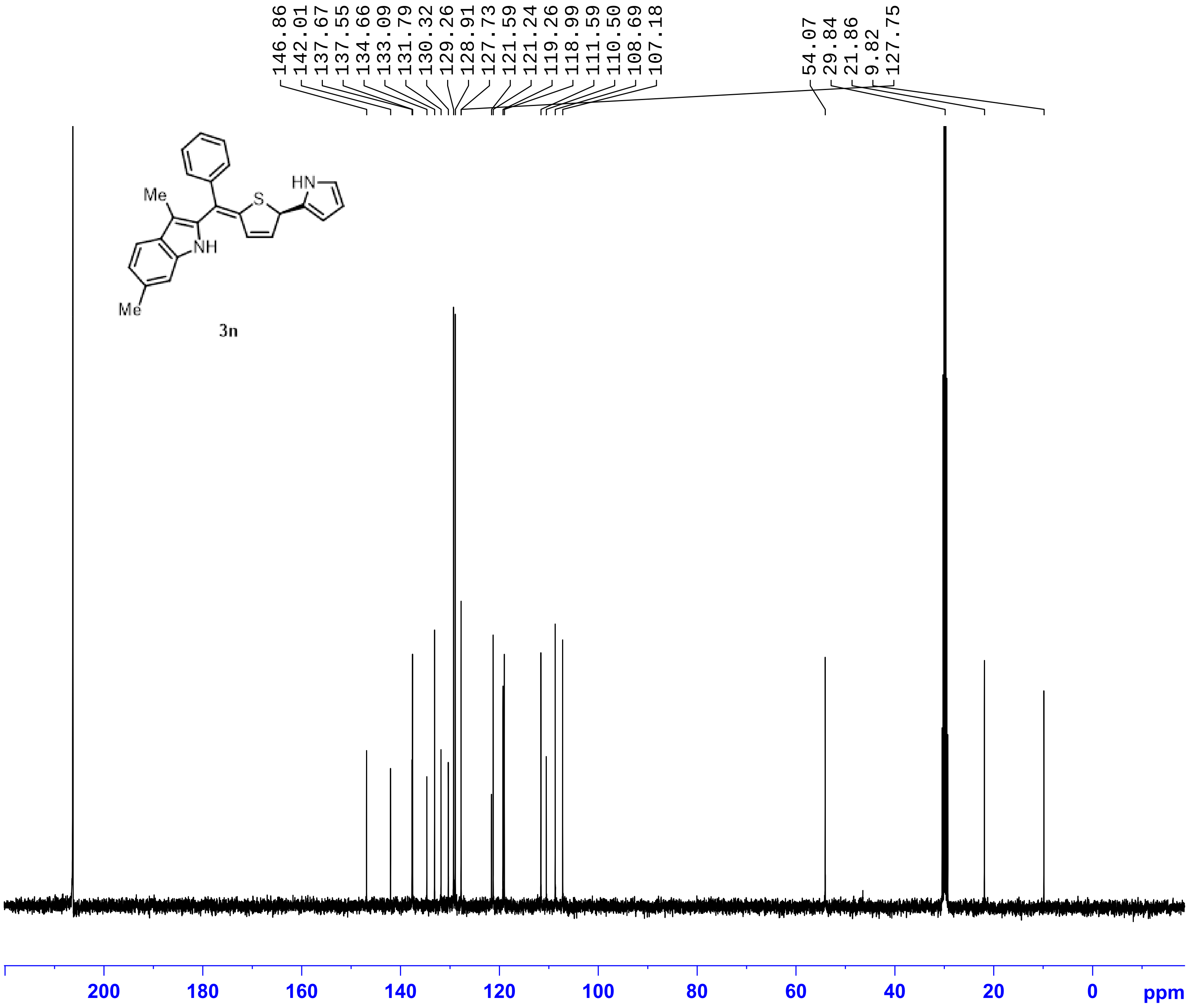

NAME	lxg-7079B
EXPNO	2
PROCNO	1
Date_	20200703
Time	20.12
INSTRUM	spect
PROBHD	5 mm PABBO BB/
PULPROG	zgpg30
TD	65536
SOLVENT	Acetone
NS	53
DS	0
SWH	24038.461 Hz
FIDRES	0.366798 Hz
AQ	1.3631988 sec
RG	196.92
DW	20.800 usec
DE	6.50 usec
TE	297.3 K
D1	2.00000000 sec
D11	0.03000000 sec
TD0	1

======== CHANNEL f1 ========

SFO1	100.6228298 MHz
NUC1	13C
P1	9.70 usec
SI	32768
SF	100.6126863 MHz
WDW	EM
SSB	0
LB	1.00 Hz
GB	0
PC	1.40

Supplementary Figure 92. ^{13}C NMR spectrum of **3n**

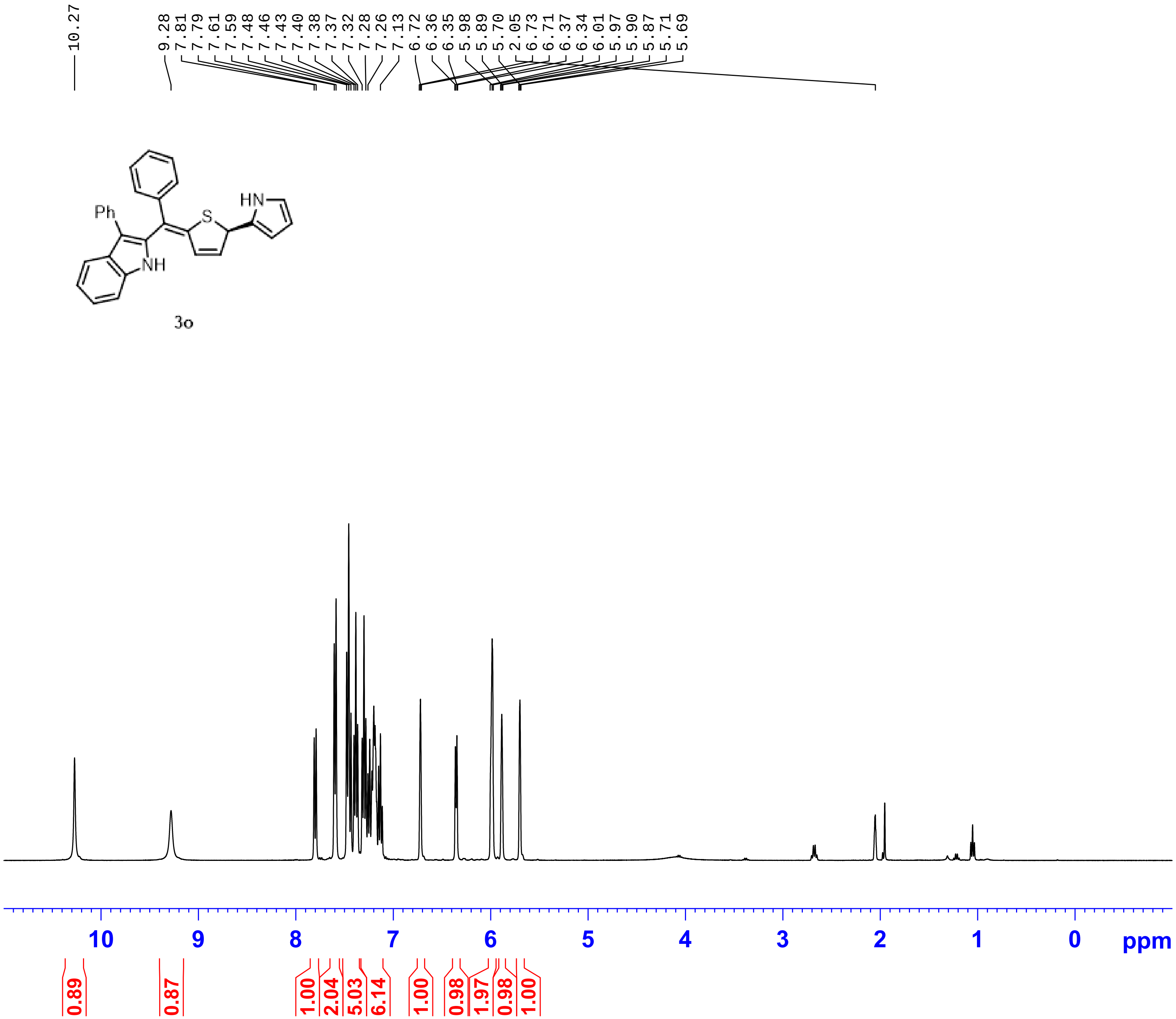

NAME	lxg-7077C
EXPNO	1
PROCNO	1
Date_	20200701
Time	17.08
INSTRUM	spect
PROBHD	5 mm PABBO BB/
PULPROG	zg30
TD	65536
SOLVENT	Acetone
NS	4
DS	0
SWH	8012.820 Hz
FIDRES	0.122266 Hz
AQ	4.0894966 sec
RG	31.55
DW	62.400 usec
DE	6.50 usec
TE	296.8 K
D1	1.00000000 sec
TD0	1

======== CHANNEL f1 ========

SFO1	400.1324710 MHz
NUC1	1H
P1	14.50 usec
SI	65536
SF	400.1300062 MHz
WDW	EM
SSB	0
LB	0.30 Hz
GB	0
PC	1.00

Supplementary Figure 93. ^{1}H NMR spectrum of **3o**

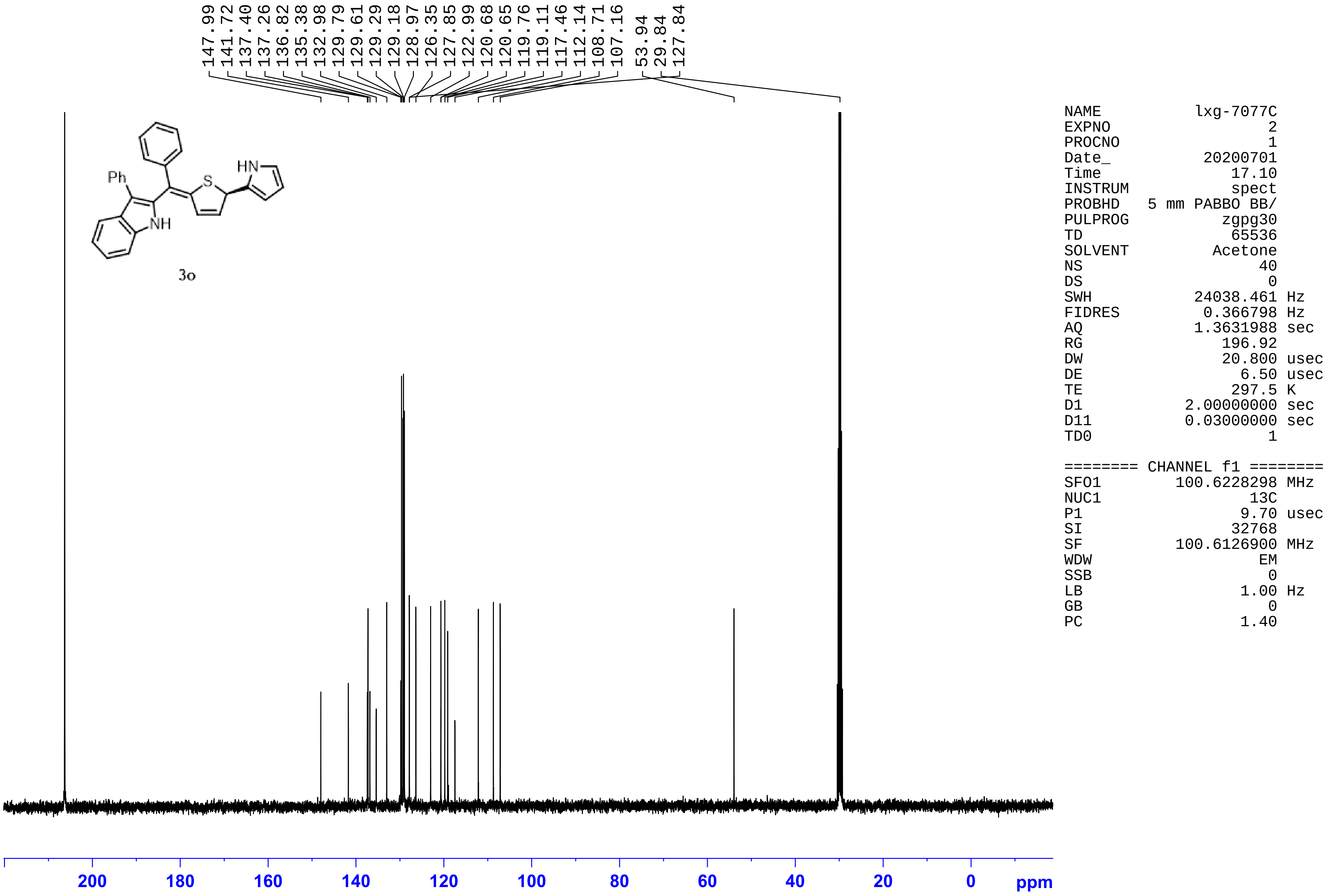

Supplementary Figure 94. ^{13}C NMR spectrum of **3o**

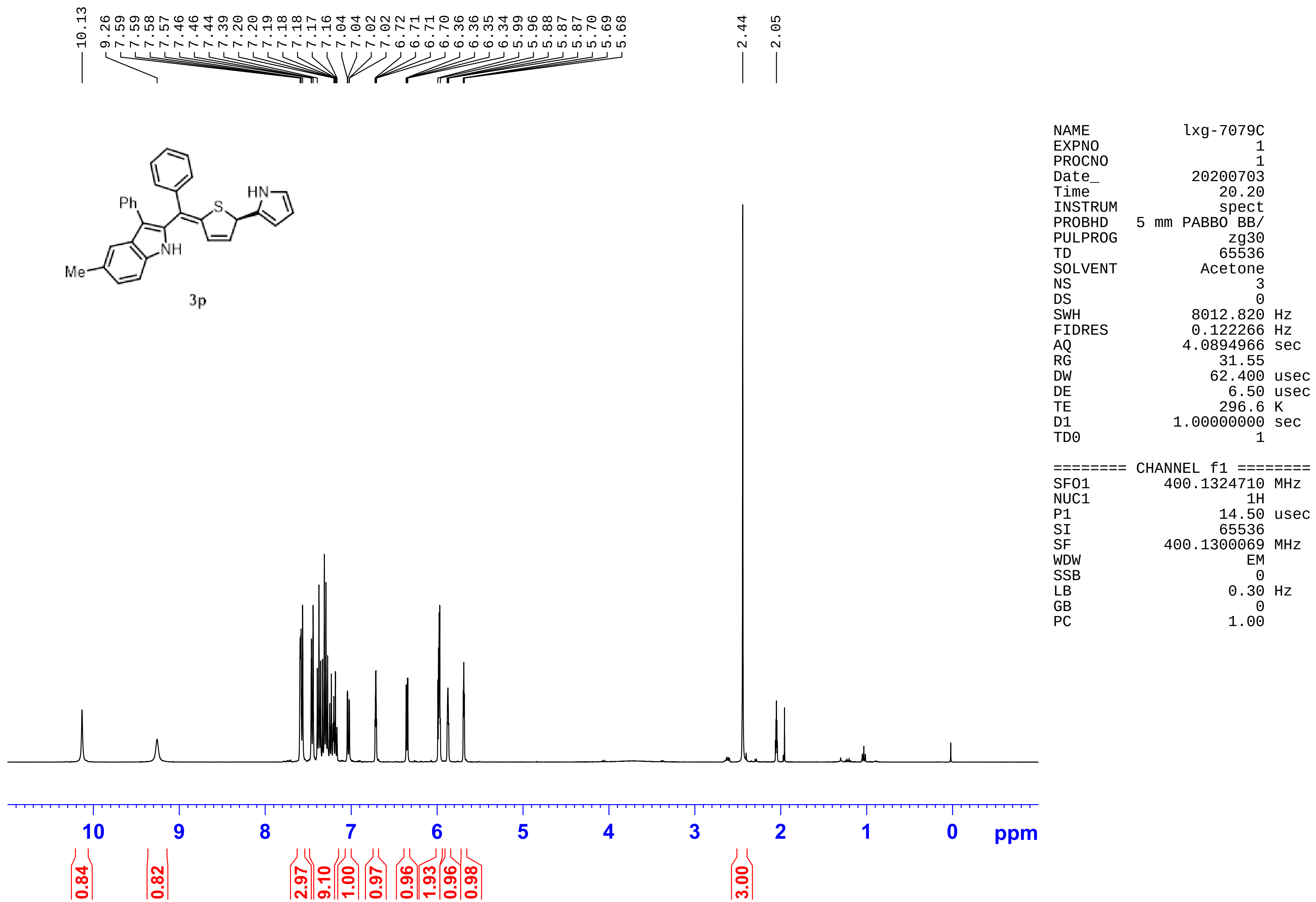

Supplementary Figure 95. ^{1}H NMR spectrum of **3p**

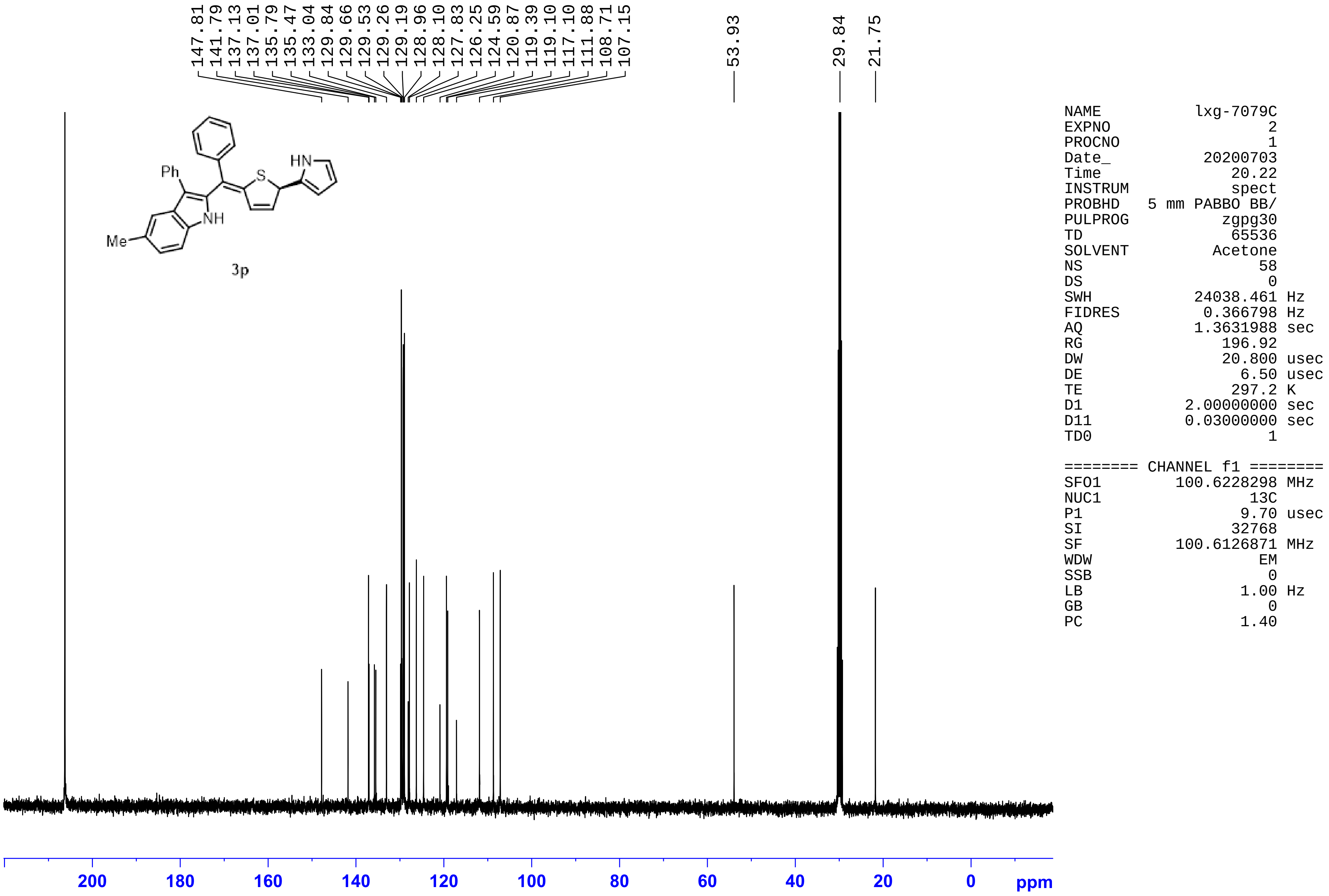

Supplementary Figure 96. ^{13}C NMR spectrum of **3p**

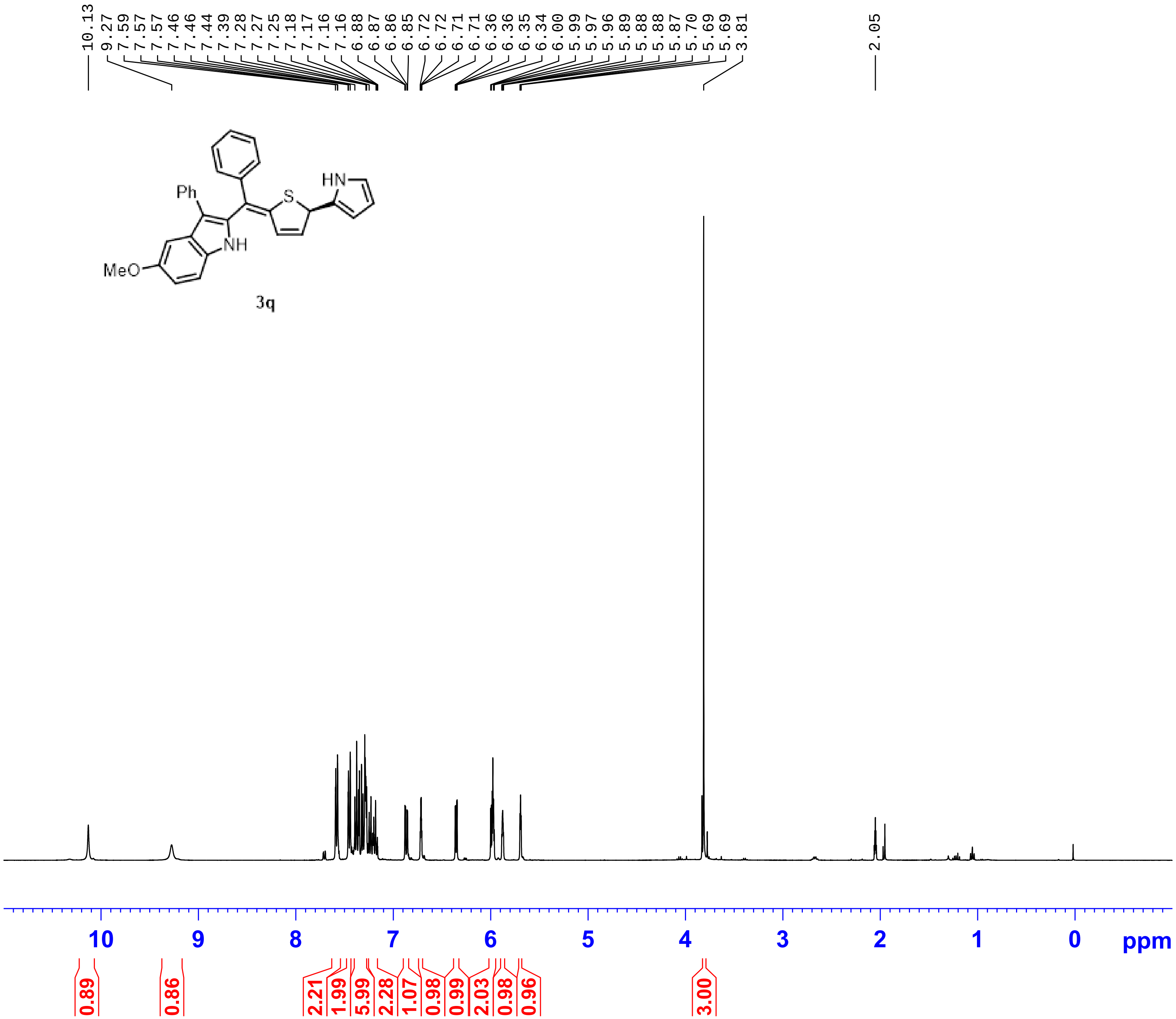

3q

NAME	lxg-7079D
EXPNO	11
PROCNO	1
Date_	20200704
Time	10.50
INSTRUM	spect
PROBHD	5 mm PABBO BB/
PULPROG	zg30
TD	65536
SOLVENT	Acetone
NS	4
DS	0
SWH	8012.820 Hz
FIDRES	0.122266 Hz
AQ	4.0894966 sec
RG	31.55
DW	62.400 usec
DE	6.50 usec
TE	296.8 K
D1	1.00000000 sec
TD0	1

======== CHANNEL f1 ========

SFO1	400.1324710 MHz
NUC1	1H
P1	14.50 usec
SI	65536
SF	400.1300069 MHz
WDW	EM
SSB	0
LB	0.30 Hz
GB	0
PC	1.00

Supplementary Figure 97. ^{1}H NMR spectrum of **3q**

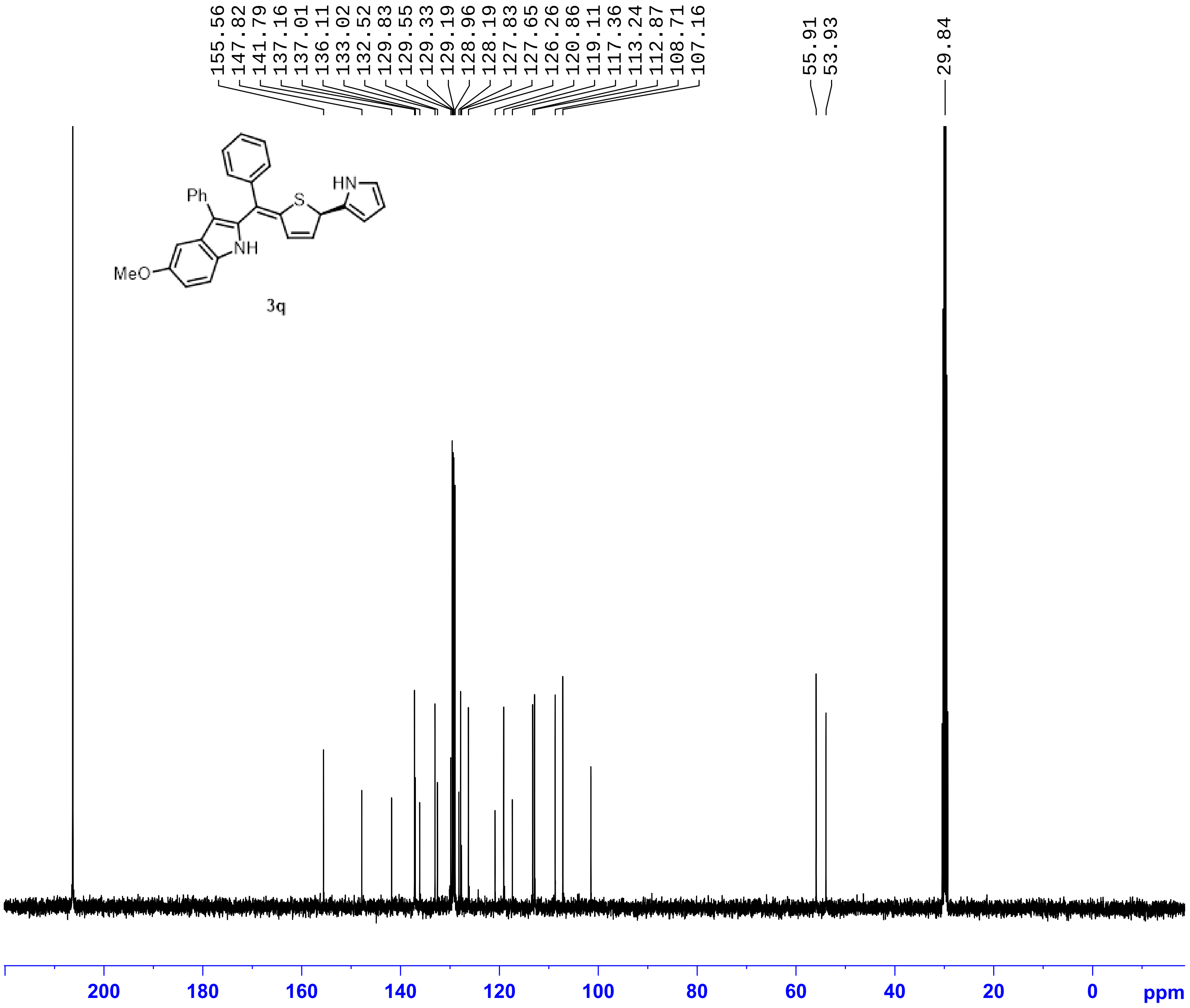

NAME	lxg-7079D	
EXPNO	2	
PROCNO	1	
Date_	20200703	
Time	22.44	
INSTRUM	spect	
PROBHD	5 mm PABBO BB/	
PULPROG	zgpg30	
TD	65536	
SOLVENT	Acetone	
NS	39	
DS	0	
SWH	24038.461	Hz
FIDRES	0.366798	Hz
AQ	1.3631988	sec
RG	196.92	
DW	20.800	usec
DE	6.50	usec
TE	298.0	K
D1	2.00000000	sec
D11	0.03000000	sec
TD0	1	
======== CHANNEL f1 ========		
SFO1	100.6228298	MHz
NUC1	13C	
P1	9.70	usec
SI	32768	
SF	100.6126878	MHz
WDW	EM	
SSB	0	
LB	1.00	Hz
GB	0	
PC	1.40	

Supplementary Figure 98. ^{13}C NMR spectrum of **3q**

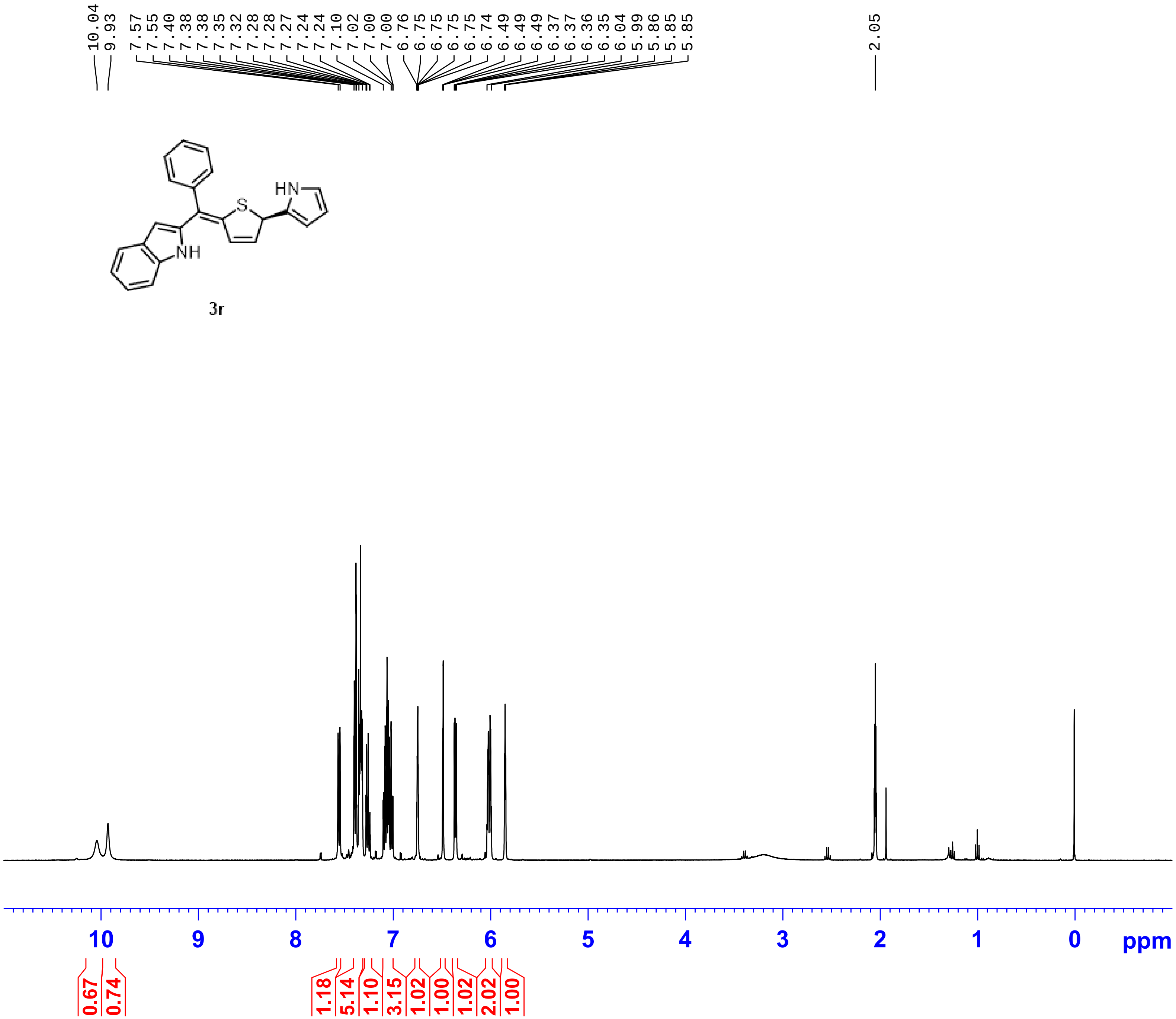

NAME	lxg-7096B
EXPNO	1
PROCNO	1
Date_	20200723
Time	22.47
INSTRUM	spect
PROBHD	5 mm PABBO BB/
PULPROG	zg30
TD	65536
SOLVENT	Acetone
NS	3
DS	0
SWH	8012.820 Hz
FIDRES	0.122266 Hz
AQ	4.0894966 sec
RG	70.97
DW	62.400 usec
DE	6.50 usec
TE	297.5 K
D1	1.00000000 sec
TD0	1

======== CHANNEL f1 ========

SFO1	400.1324710 MHz
NUC1	1H
P1	14.50 usec
SI	65536
SF	400.1300070 MHz
WDW	EM
SSB	0
LB	0.30 Hz
GB	0
PC	1.00

Supplementary Figure 99. ^{1}H NMR spectrum of **3r**

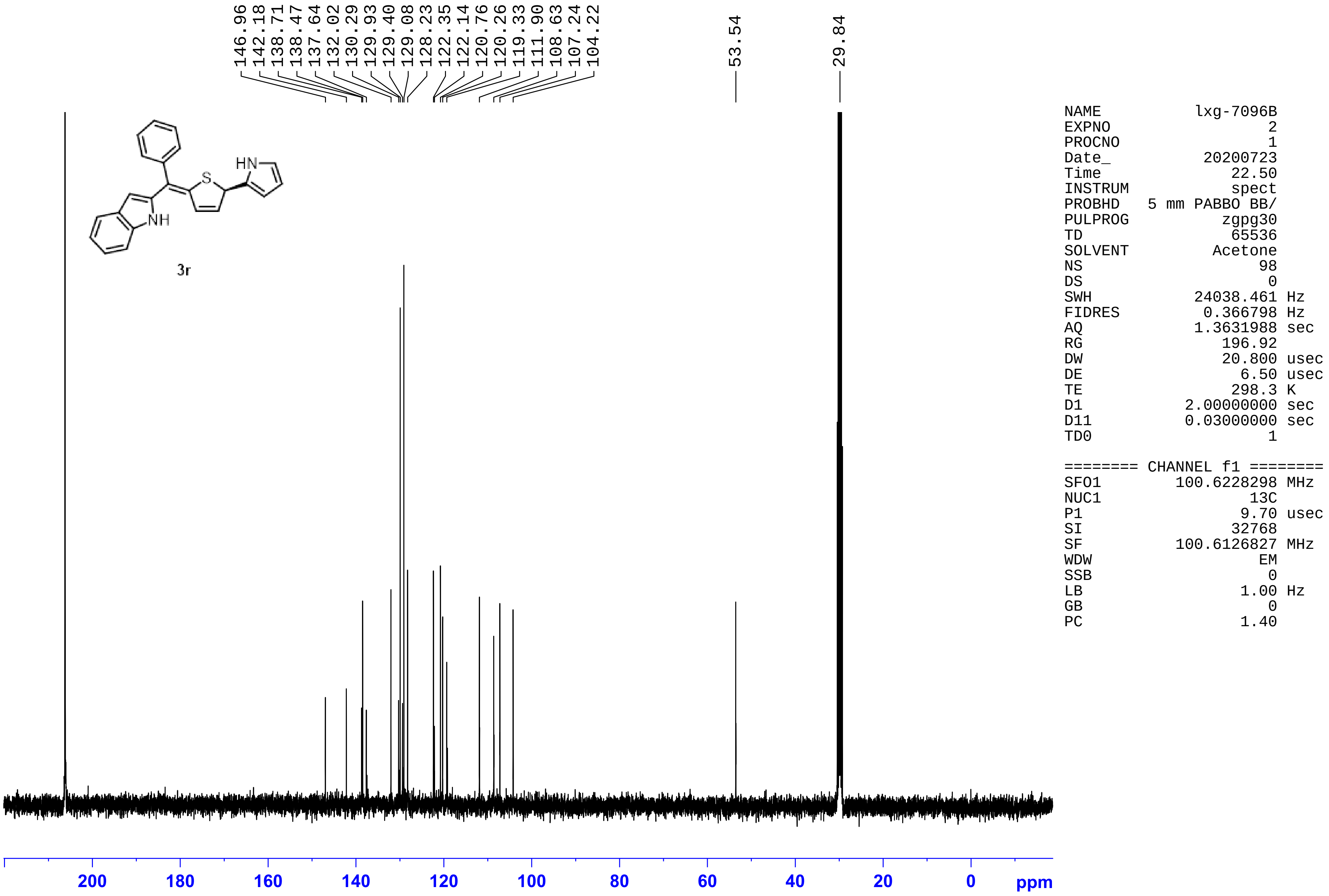

Supplementary Figure 100. ^{13}C NMR spectrum of **3r**

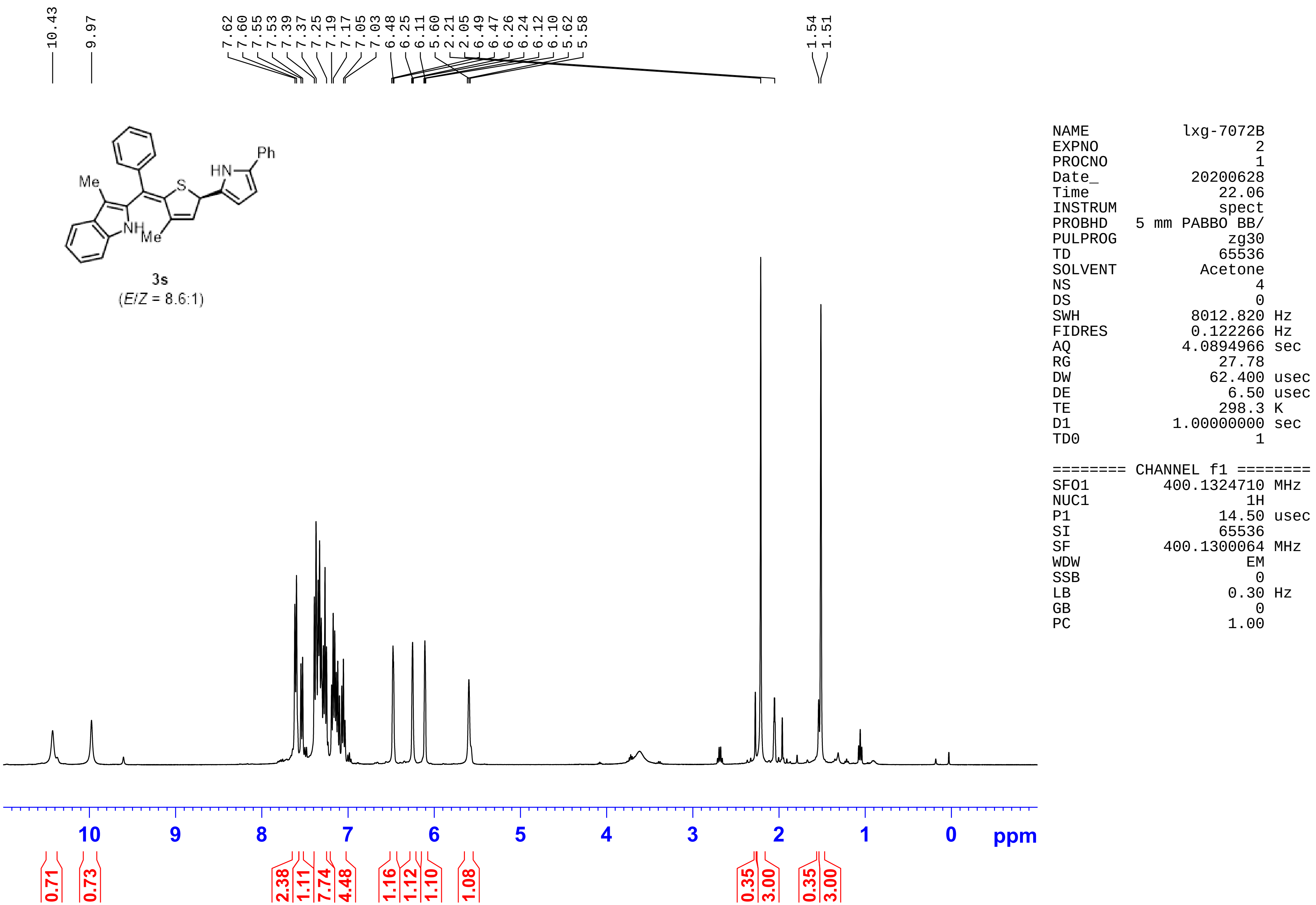

Supplementary Figure 101. ^{1}H NMR spectrum of **3s**

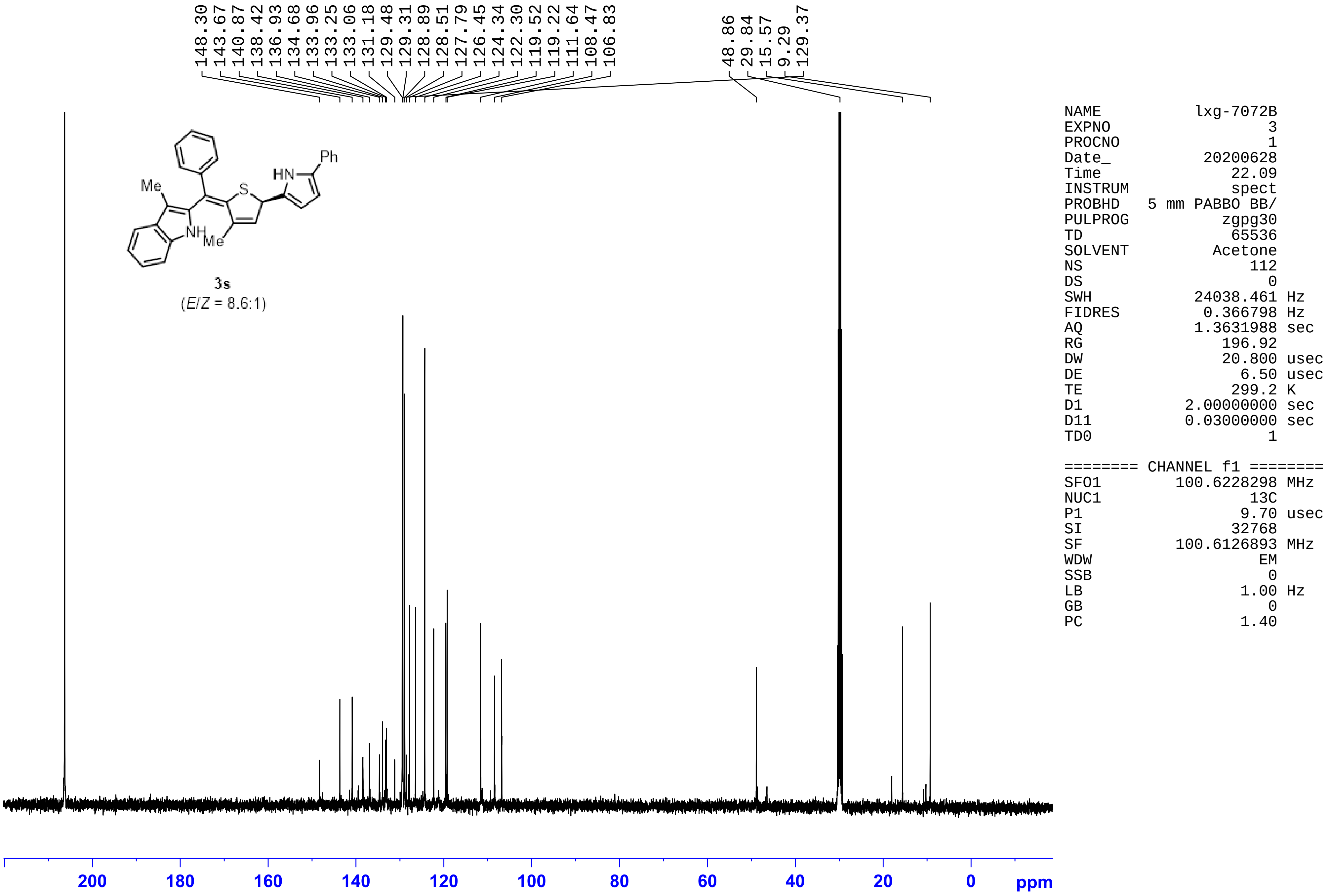

Supplementary Figure 102. ^{13}C NMR spectrum of **3s**

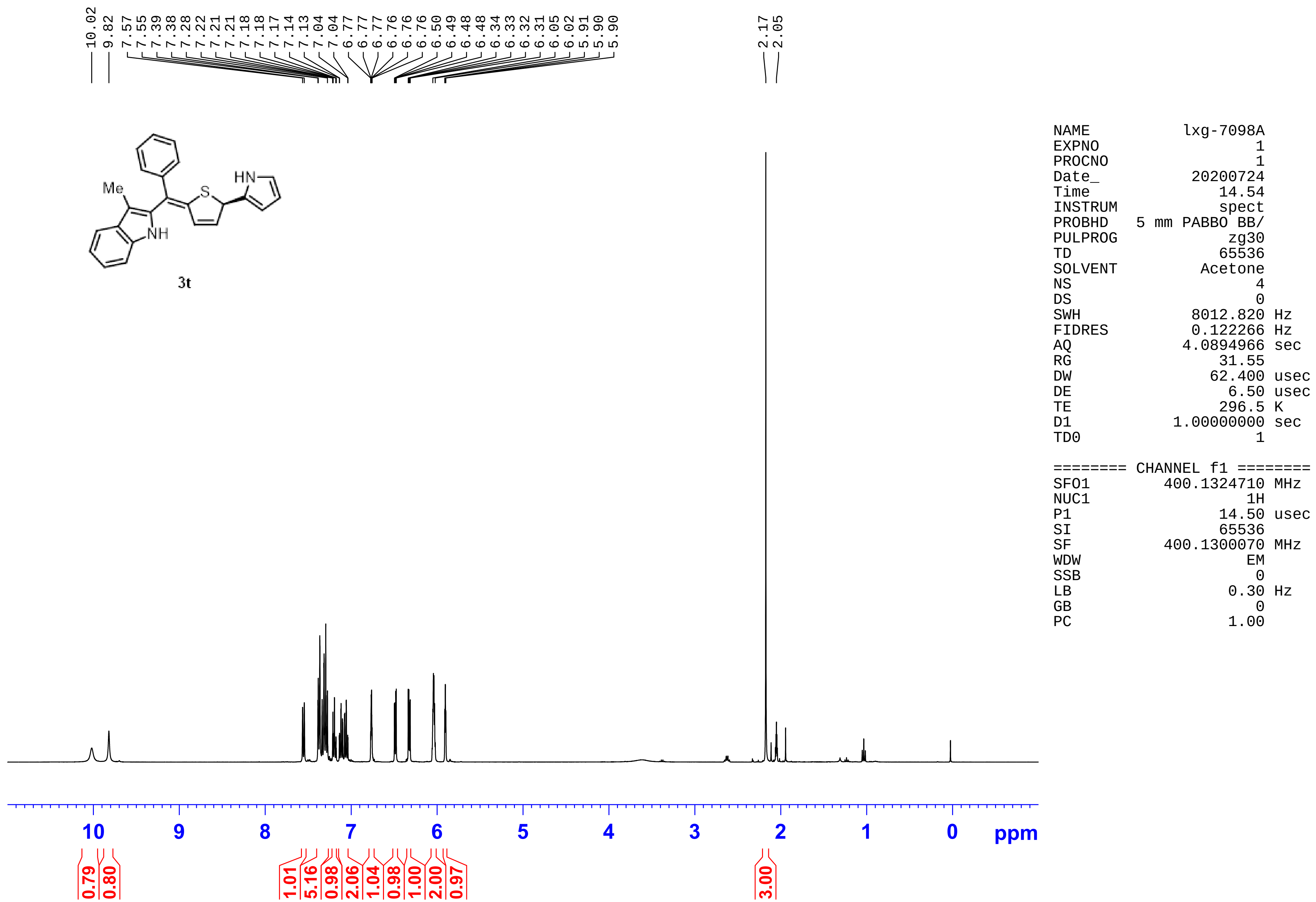

Supplementary Figure 103. ^{1}H NMR spectrum of **3t**

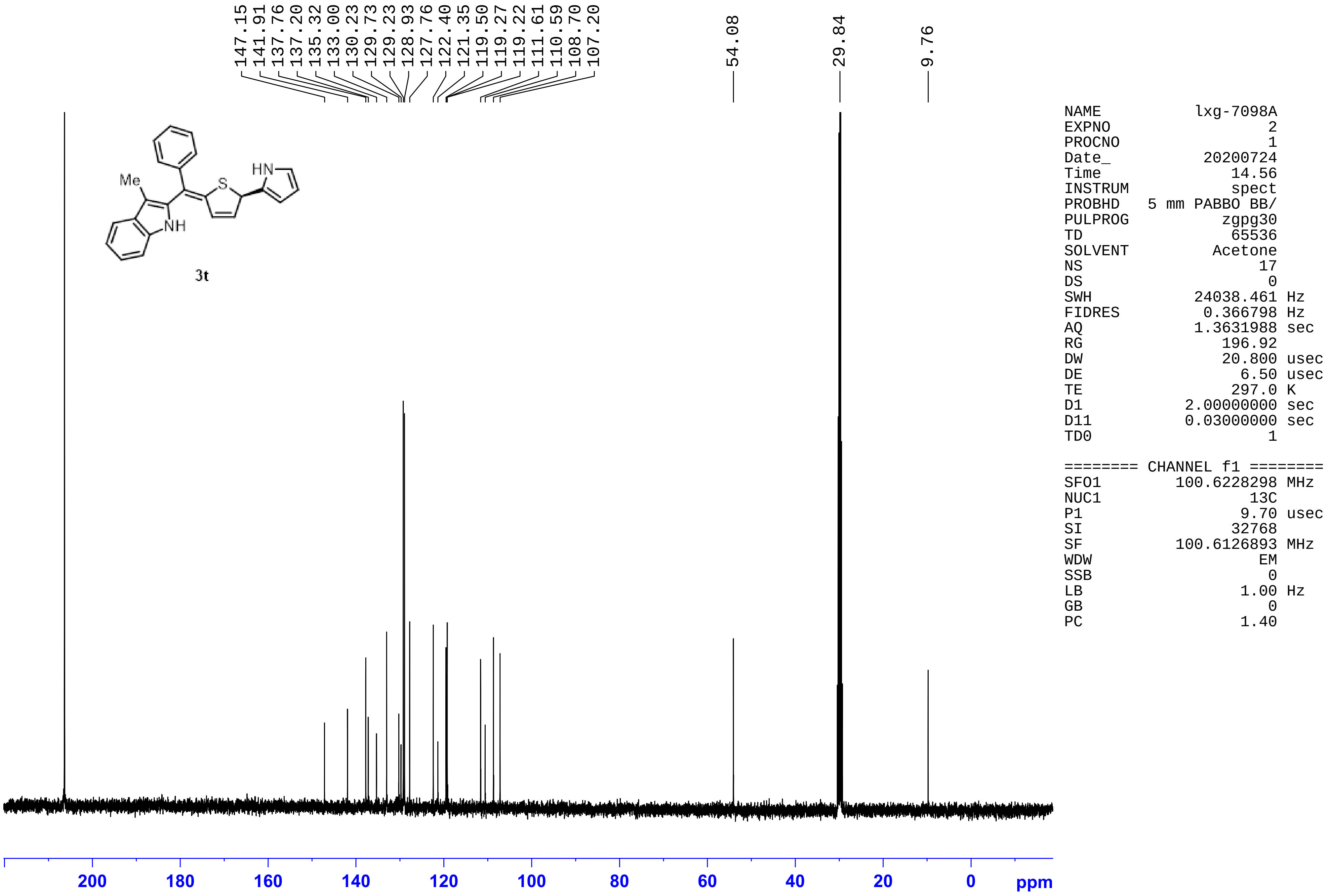

Supplementary Figure 104. ^{13}C NMR spectrum of **3t**

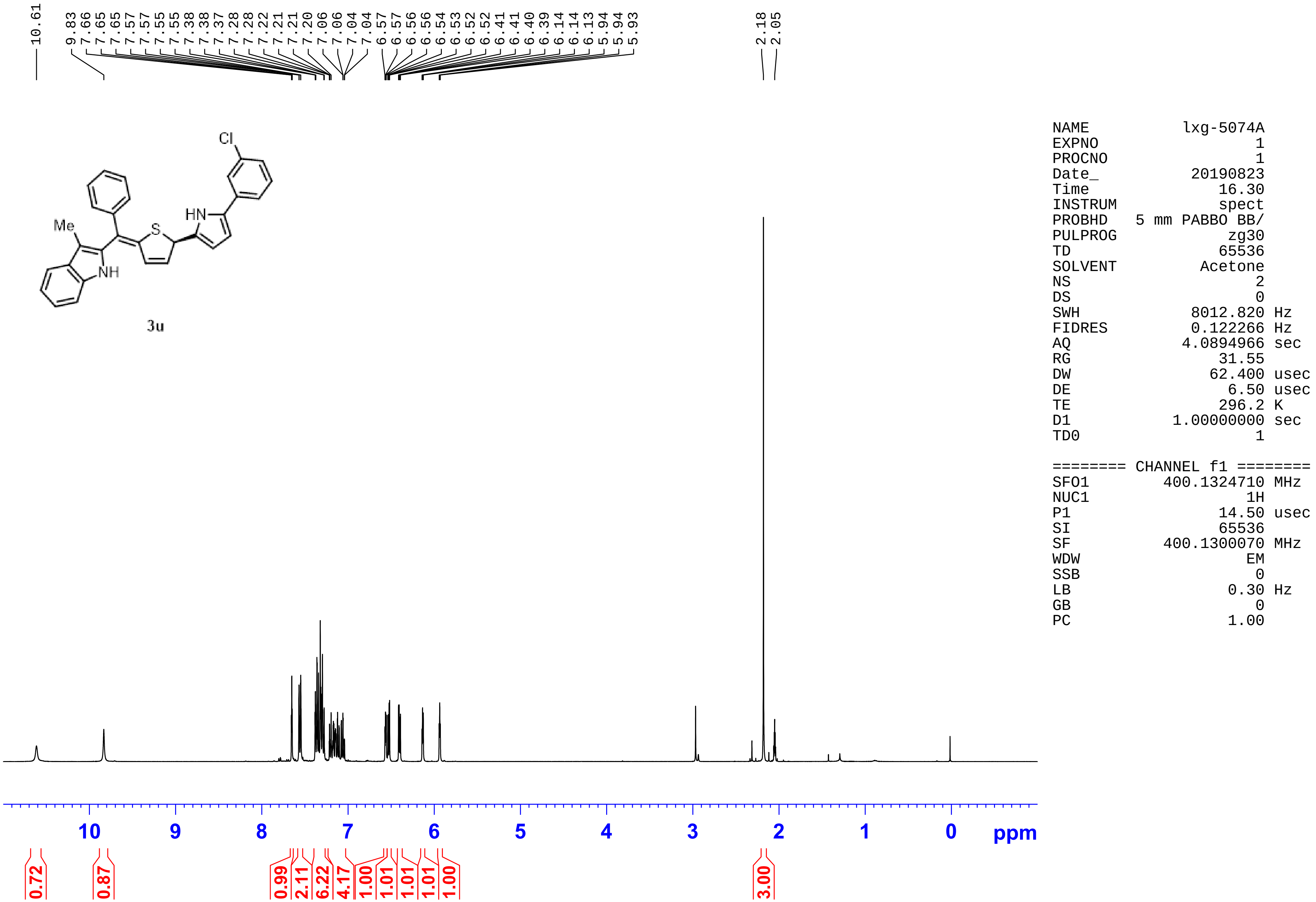

Supplementary Figure 105. ^{1}H NMR spectrum of **3u**

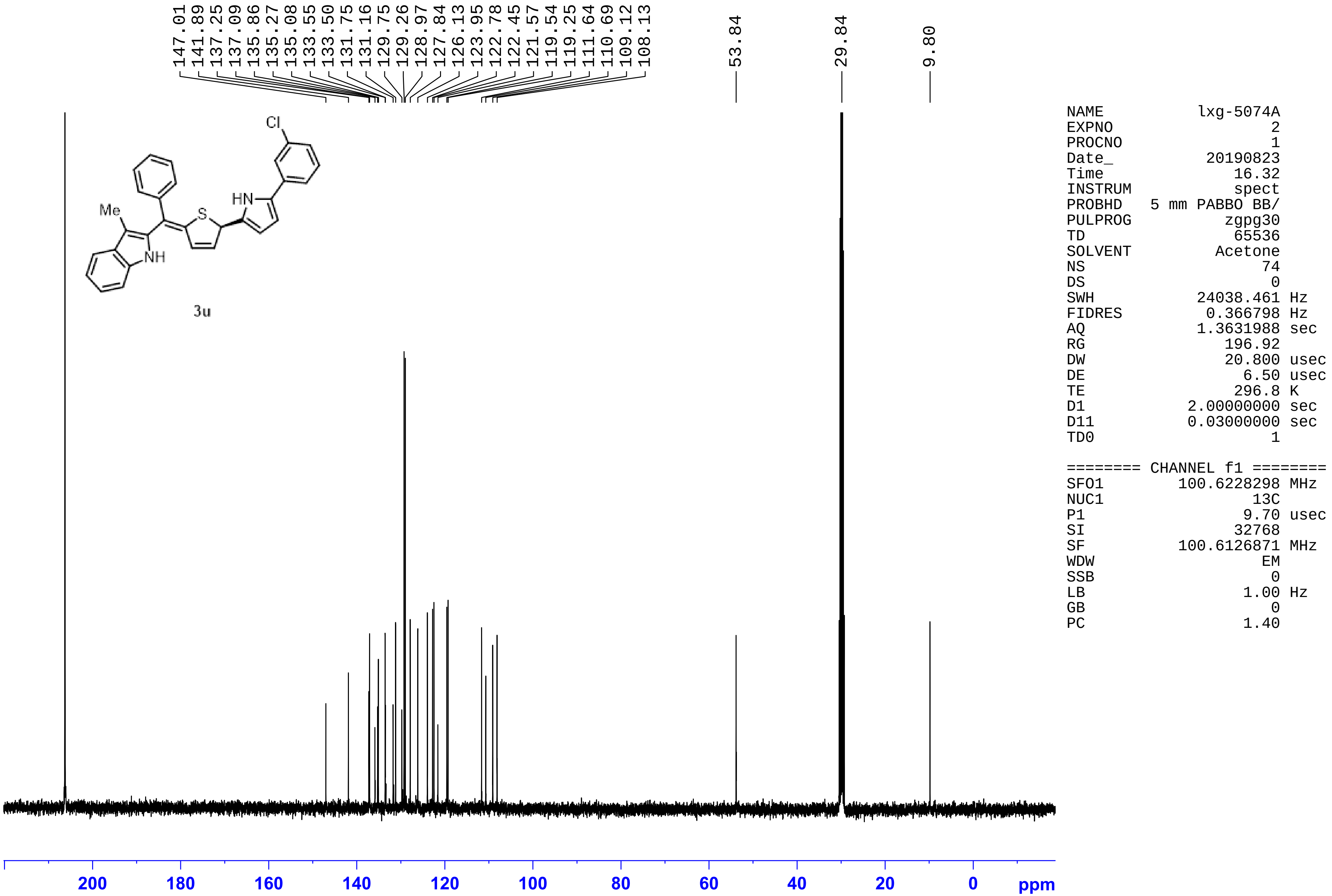

Supplementary Figure 106. ^{13}C NMR spectrum of **3u**

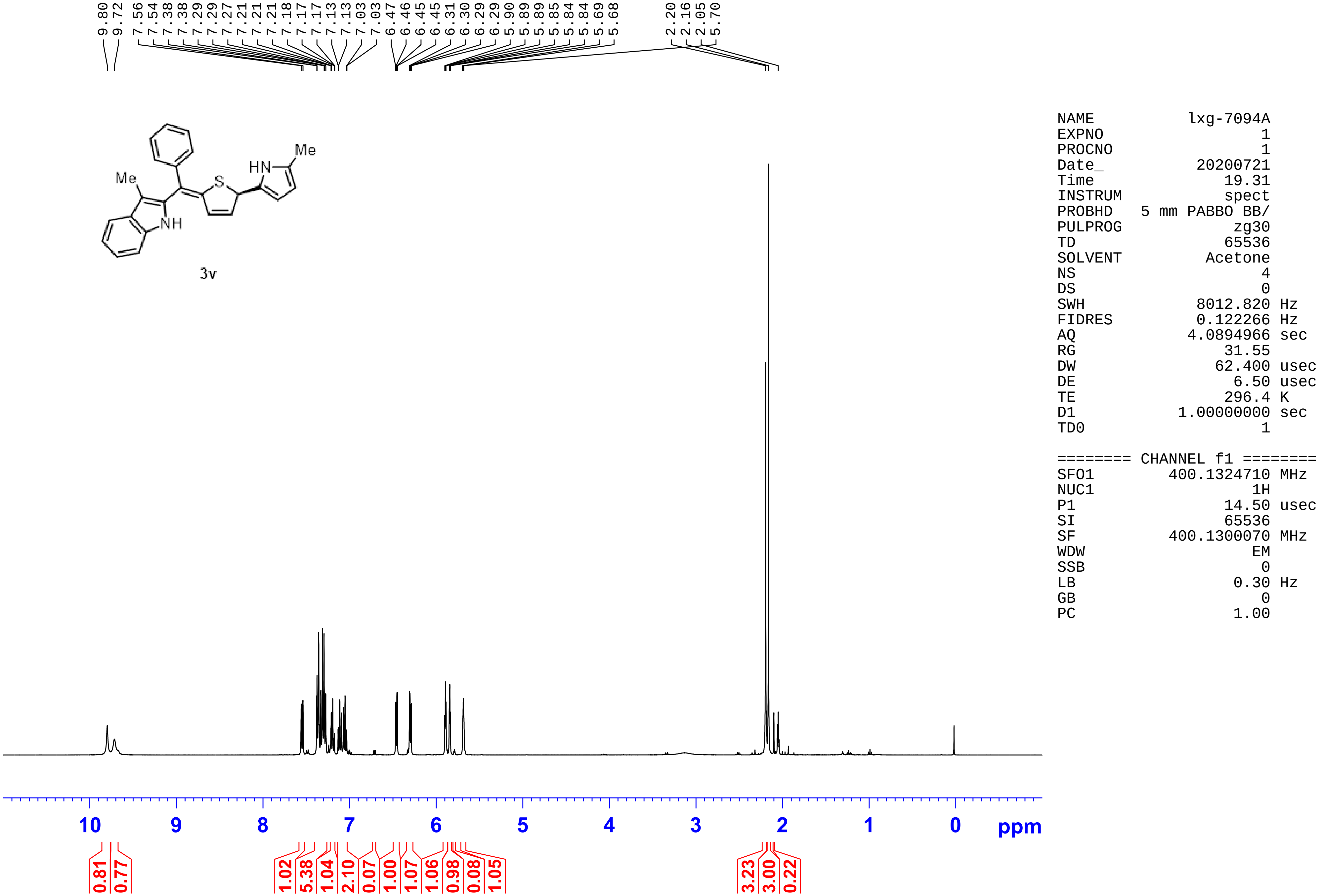

Supplementary Figure 107. ^{1}H NMR spectrum of **3v**

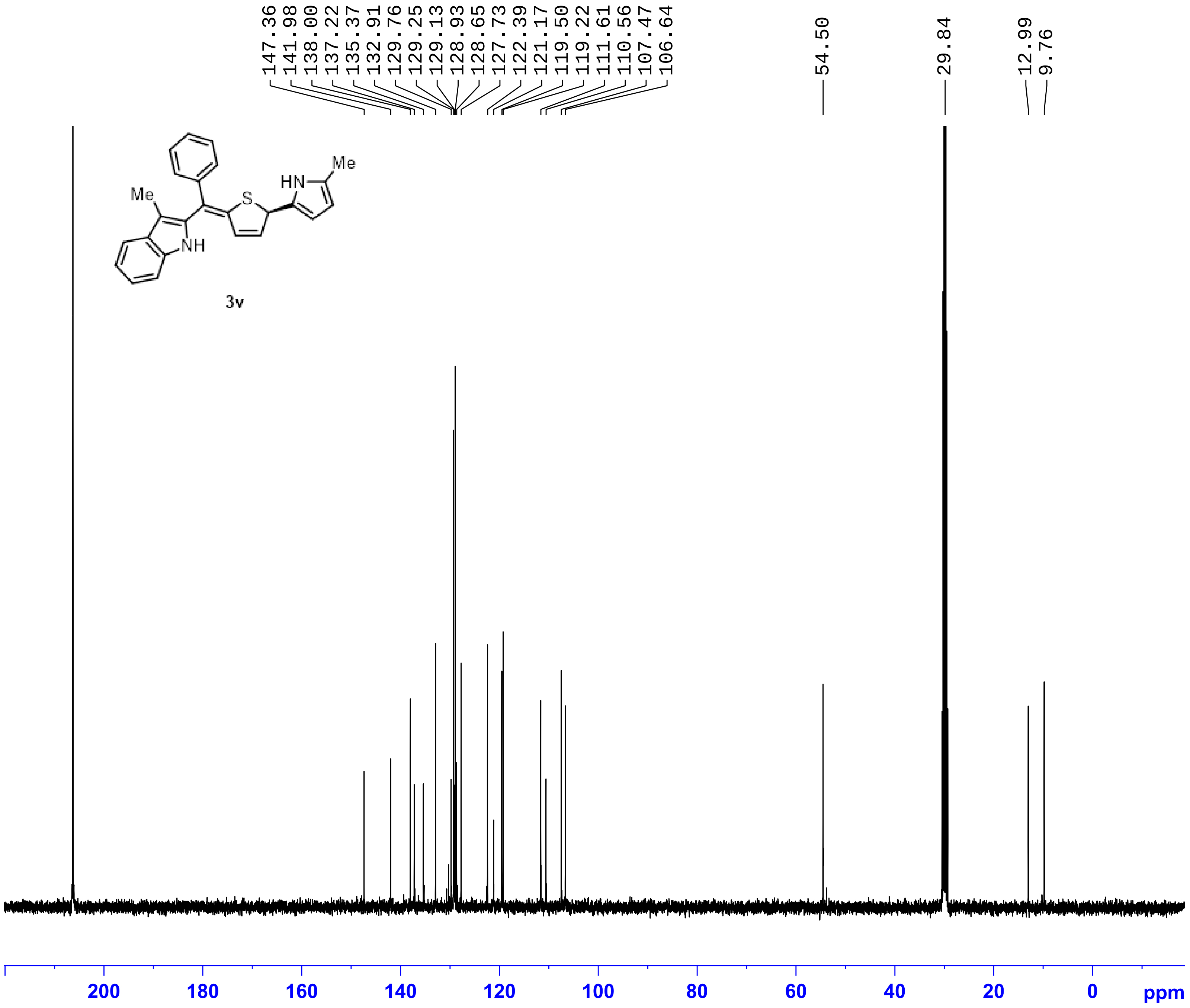

NAME	lxg-7094A
EXPNO	2
PROCNO	1
Date_	20200721
Time	19.34
INSTRUM	spect
PROBHD	5 mm PABBO BB/
PULPROG	zgpg30
TD	65536
SOLVENT	Acetone
NS	89
DS	0
SWH	24038.461 Hz
FIDRES	0.366798 Hz
AQ	1.3631988 sec
RG	196.92
DW	20.800 usec
DE	6.50 usec
TE	297.2 K
D1	2.00000000 sec
D11	0.03000000 sec
TD0	1

======== CHANNEL f1 ========

SFO1	100.6228298 MHz
NUC1	13C
P1	9.70 usec
SI	32768
SF	100.6126863 MHz
WDW	EM
SSB	0
LB	1.00 Hz
GB	0
PC	1.40

Supplementary Figure 108. ^{13}C NMR spectrum of **3v**

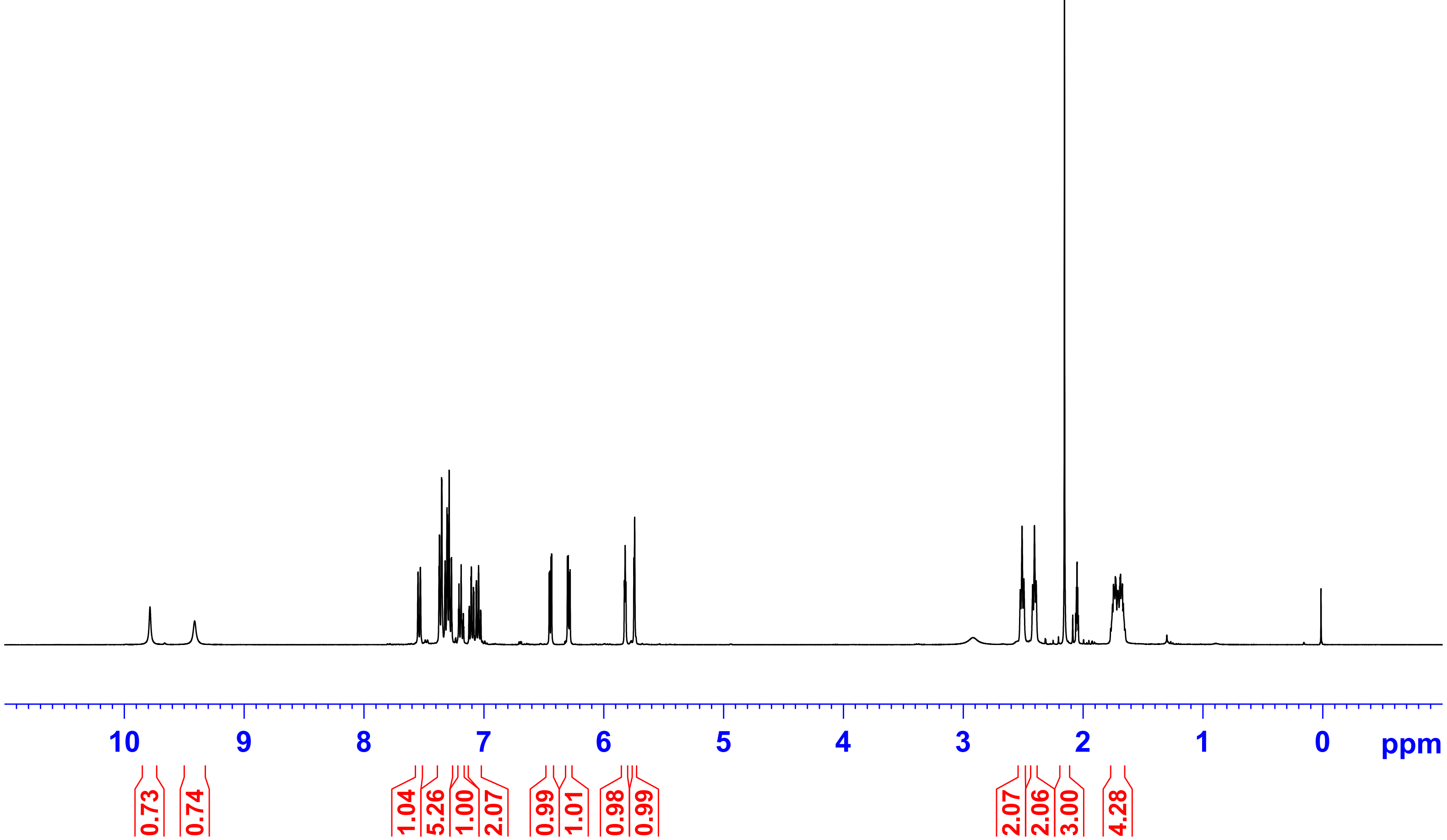

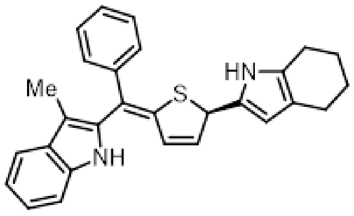

3w

```
NAME              lxg-7094B
EXPNO                     1
PROCNO                    1
Date_              20200721
Time                  22.44
INSTRUM               spect
PROBHD     5 mm PABBO BB/
PULPROG                zg30
TD                    65536
SOLVENT             Acetone
NS                        4
DS                        0
SWH             8012.820 Hz
FIDRES          0.122266 Hz
AQ             4.0894966 sec
RG                    34.77
DW               62.400 usec
DE                 6.50 usec
TE                  297.7 K
D1            1.00000000 sec
TD0                       1

======== CHANNEL f1 ========
SFO1         400.1324710 MHz
NUC1                     1H
P1                14.50 usec
SI                    65536
SF           400.1300070 MHz
WDW                      EM
SSB                       0
LB                  0.30 Hz
GB                        0
PC                     1.00
```


Supplementary Figure 109. ^{1}H NMR spectrum of **3w**

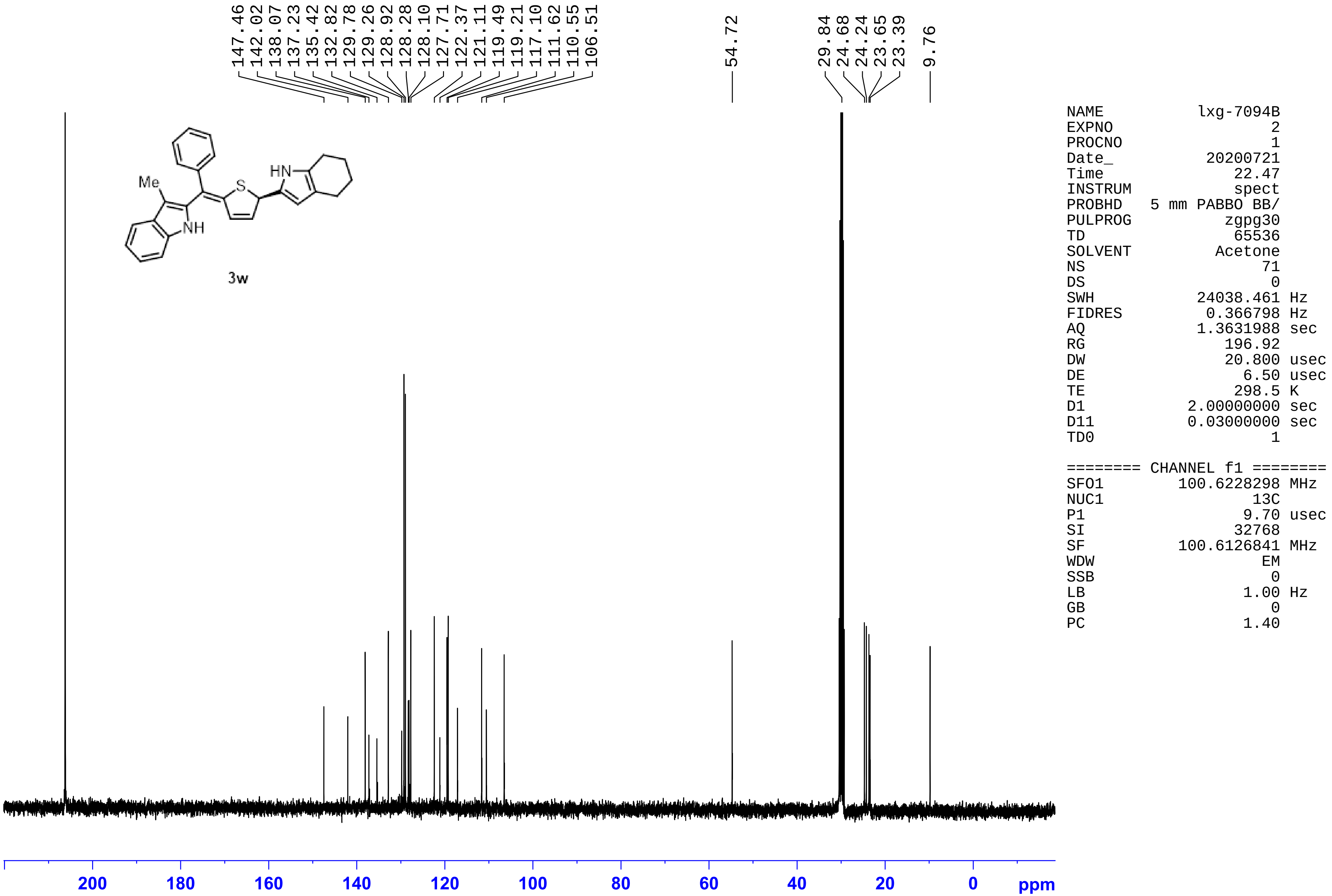

3w

Supplementary Figure 110. ^{13}C NMR spectrum of **3w**

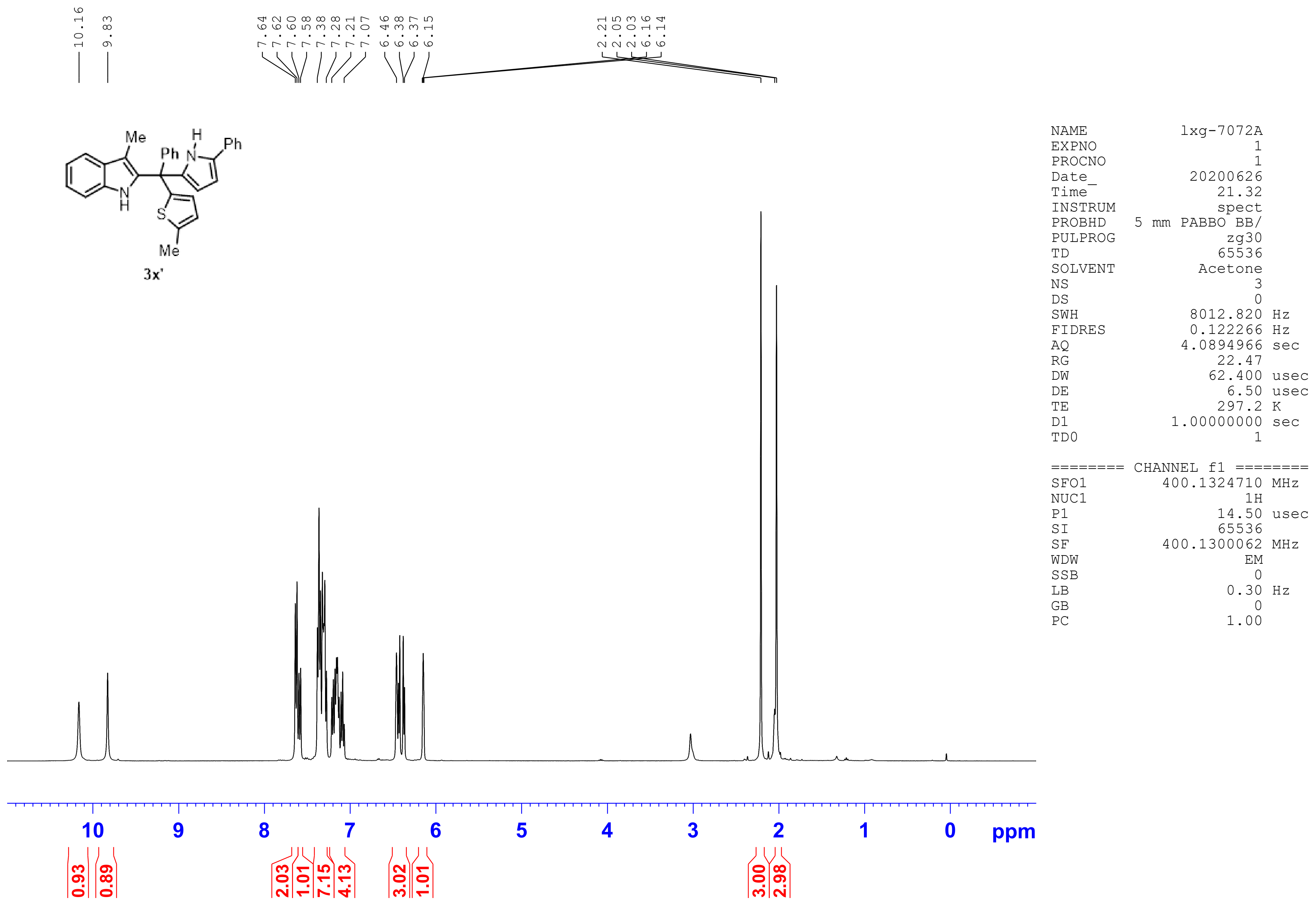

Supplementary Figure 111. ^{1}H NMR spectrum of **3x'**

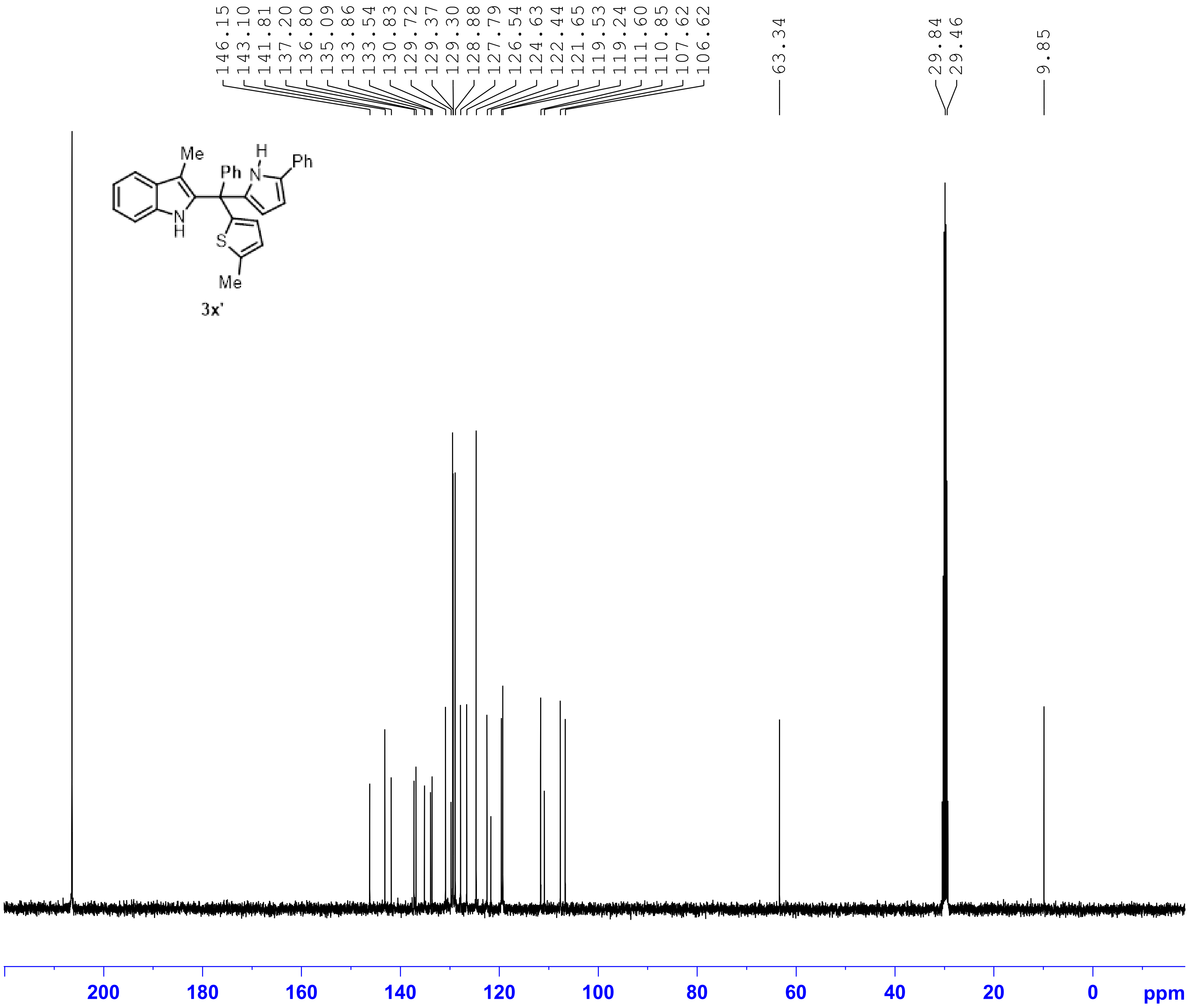

NAME	lxg-7072A
EXPNO	2
PROCNO	1
Date_	20200626
Time	21.35
INSTRUM	spect
PROBHD	5 mm PABBO BB/
PULPROG	zgpg30
TD	65536
SOLVENT	Acetone
NS	24
DS	0
SWH	24038.461 Hz
FIDRES	0.366798 Hz
AQ	1.3631988 sec
RG	196.92
DW	20.800 usec
DE	6.50 usec
TE	297.8 K
D1	2.00000000 sec
D11	0.03000000 sec
TD0	1
======== CHANNEL f1 ========	
SFO1	100.6228298 MHz
NUC1	13C
P1	9.70 usec
SI	32768
SF	100.6126951 MHz
WDW	EM
SSB	0
LB	1.00 Hz
GB	0
PC	1.40

Supplementary Figure 112. ^{13}C NMR spectrum of **3x'**

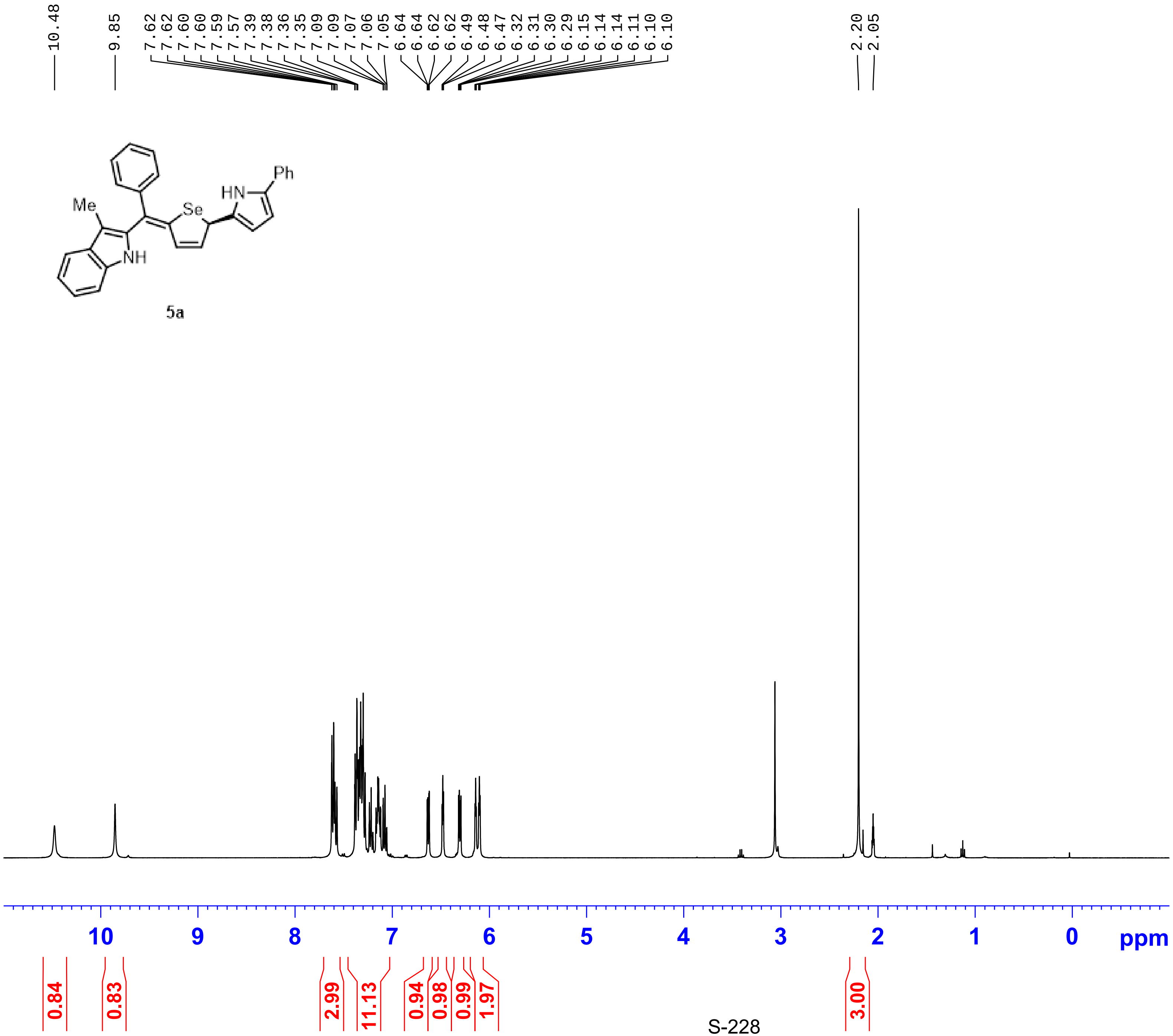

NAME	lxg-5069A	
EXPNO	1	
PROCNO	1	
Date_	20190821	
Time	19.28	
INSTRUM	spect	
PROBHD	5 mm PABBO BB/	
PULPROG	zg30	
TD	65536	
SOLVENT	Acetone	
NS	2	
DS	0	
SWH	8012.820	Hz
FIDRES	0.122266	Hz
AQ	4.0894966	sec
RG	25.32	
DW	62.400	usec
DE	6.50	usec
TE	296.4	K
D1	1.00000000	sec
TD0	1	

======== CHANNEL f1 ========

SFO1	400.1324710	MHz
NUC1	1H	
P1	14.50	usec
SI	65536	
SF	400.1300070	MHz
WDW	EM	
SSB	0	
LB	0.30	Hz
GB	0	
PC	1.00	

Supplementary Figure 113. ^{1}H NMR spectrum of **5a**

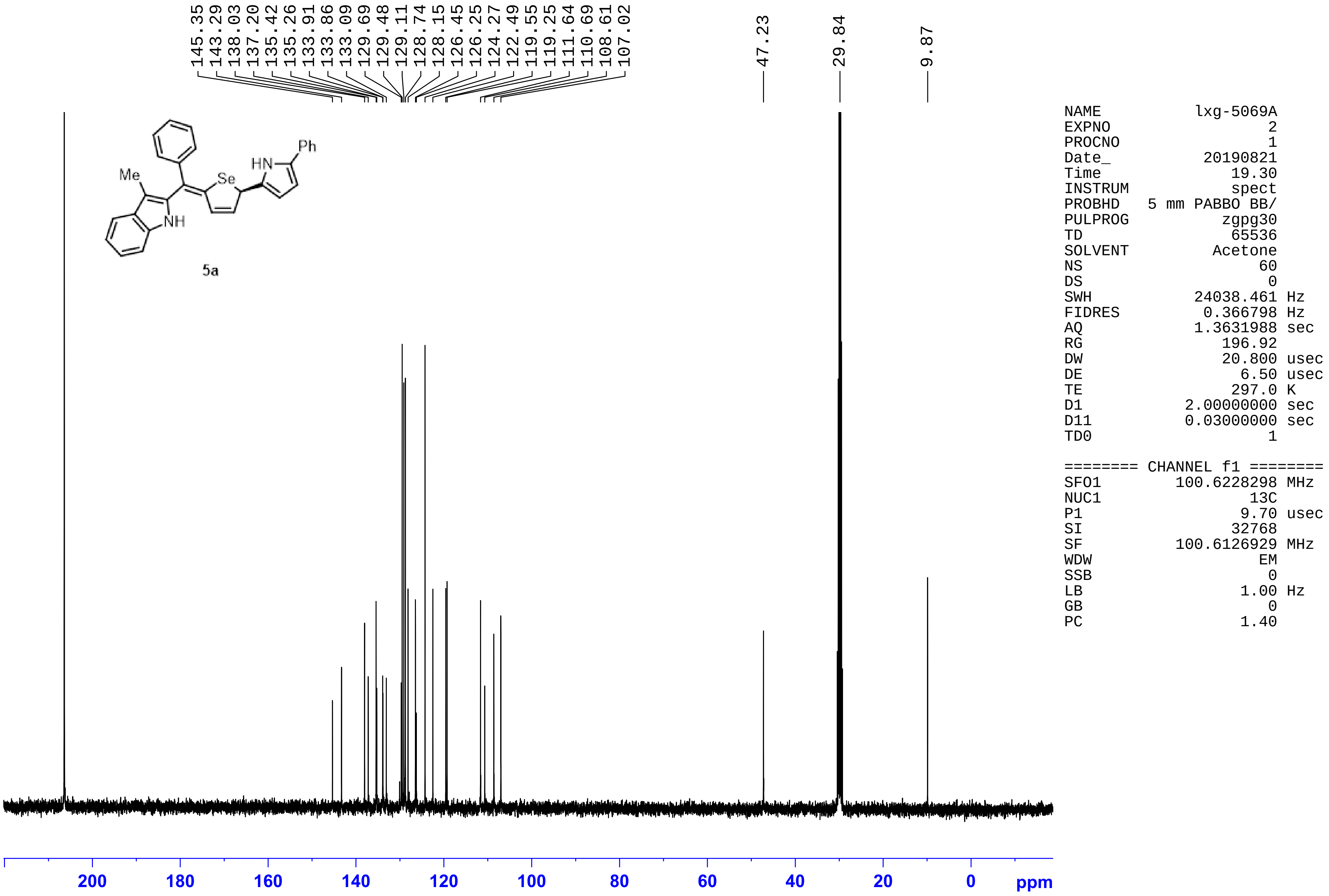

Supplementary Figure 114. ^{13}C NMR spectrum of **5a**

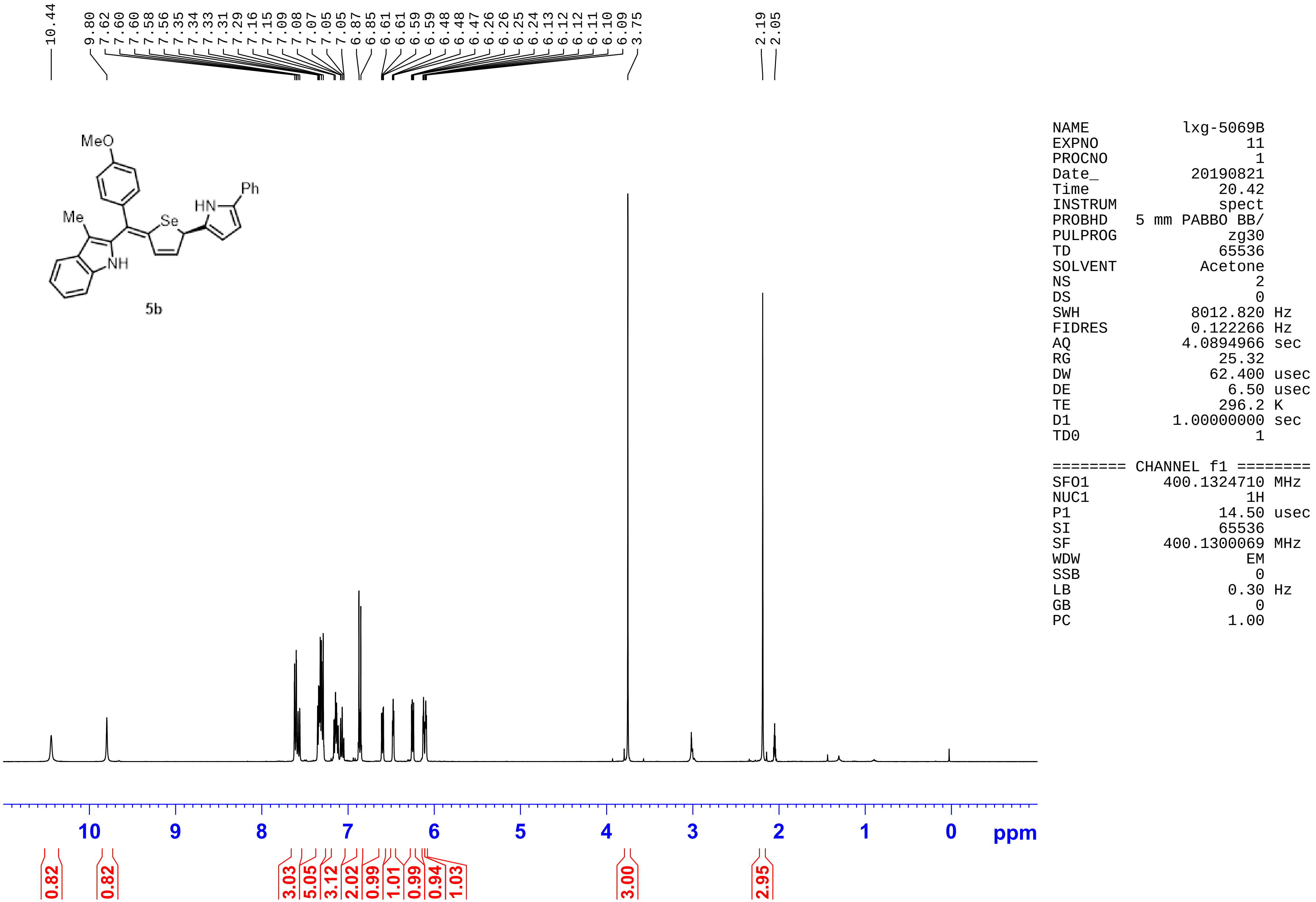

Supplementary Figure 115. ^{1}H NMR spectrum of **5b**

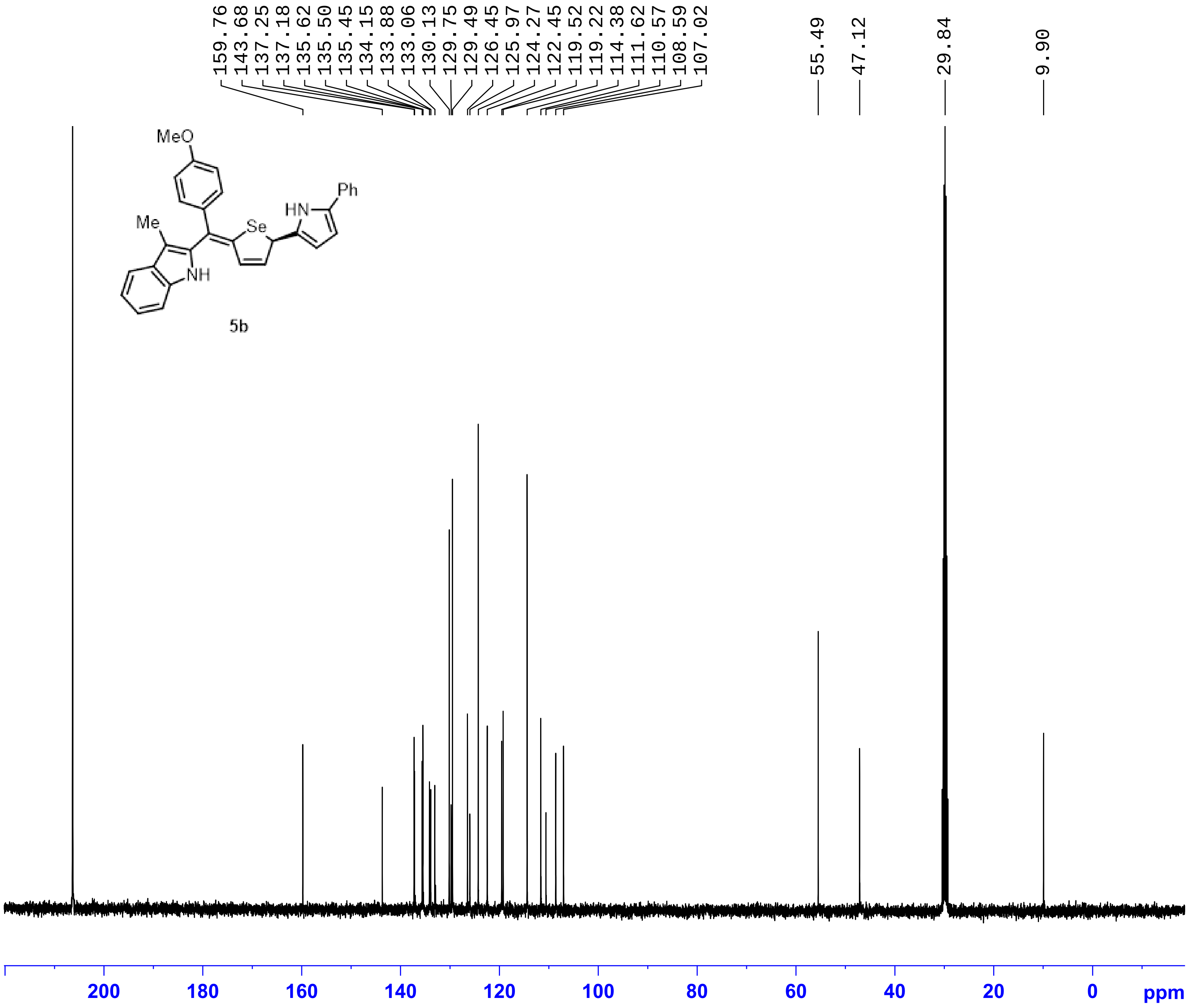

NAME	lxg-5069B
EXPNO	12
PROCNO	1
Date_	20190821
Time	20.44
INSTRUM	spect
PROBHD	5 mm PABBO BB/
PULPROG	zgpg30
TD	65536
SOLVENT	Acetone
NS	49
DS	0
SWH	24038.461 Hz
FIDRES	0.366798 Hz
AQ	1.3631988 sec
RG	196.92
DW	20.800 usec
DE	6.50 usec
TE	297.0 K
D1	2.00000000 sec
D11	0.03000000 sec
TD0	1

======== CHANNEL f1 ========

SFO1	100.6228298 MHz
NUC1	13C
P1	9.70 usec
SI	32768
SF	100.6126915 MHz
WDW	EM
SSB	0
LB	1.00 Hz
GB	0
PC	1.40

Supplementary Figure 116. ^{1}H NMR spectrum of **5b**

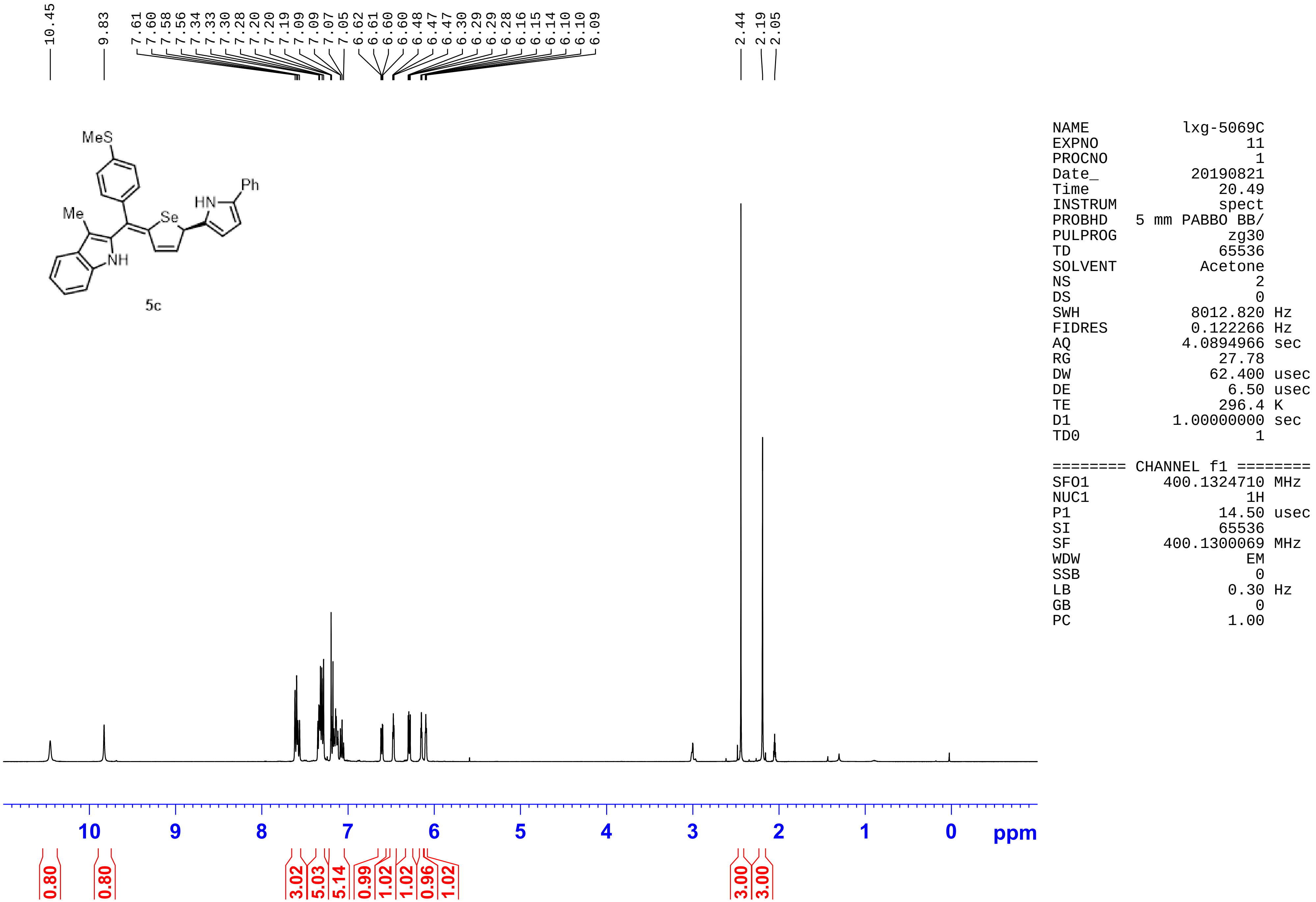

Supplementary Figure 117. ^{1}H NMR spectrum of **5c**

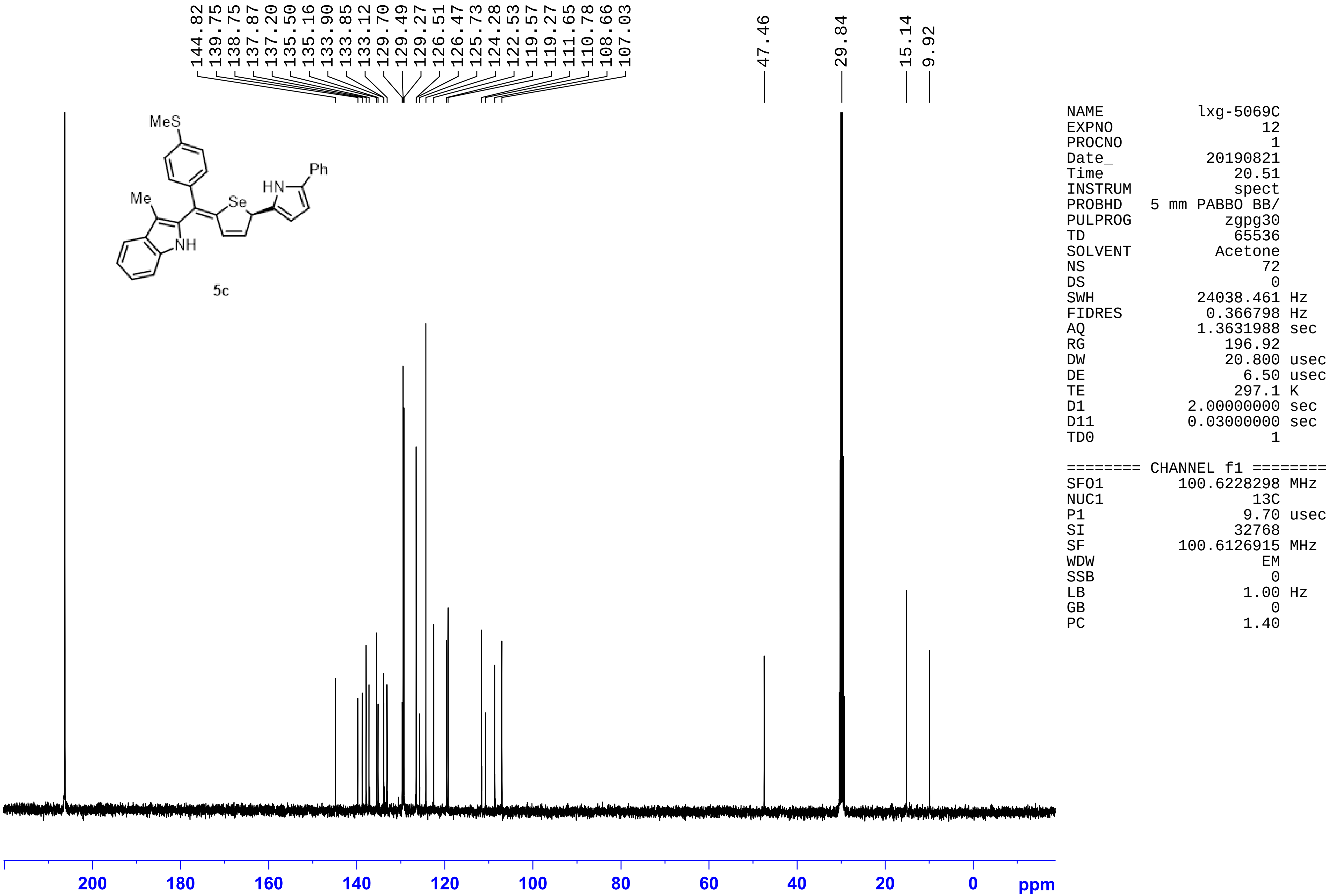

Supplementary Figure 118. ^{13}C NMR spectrum of **5c**

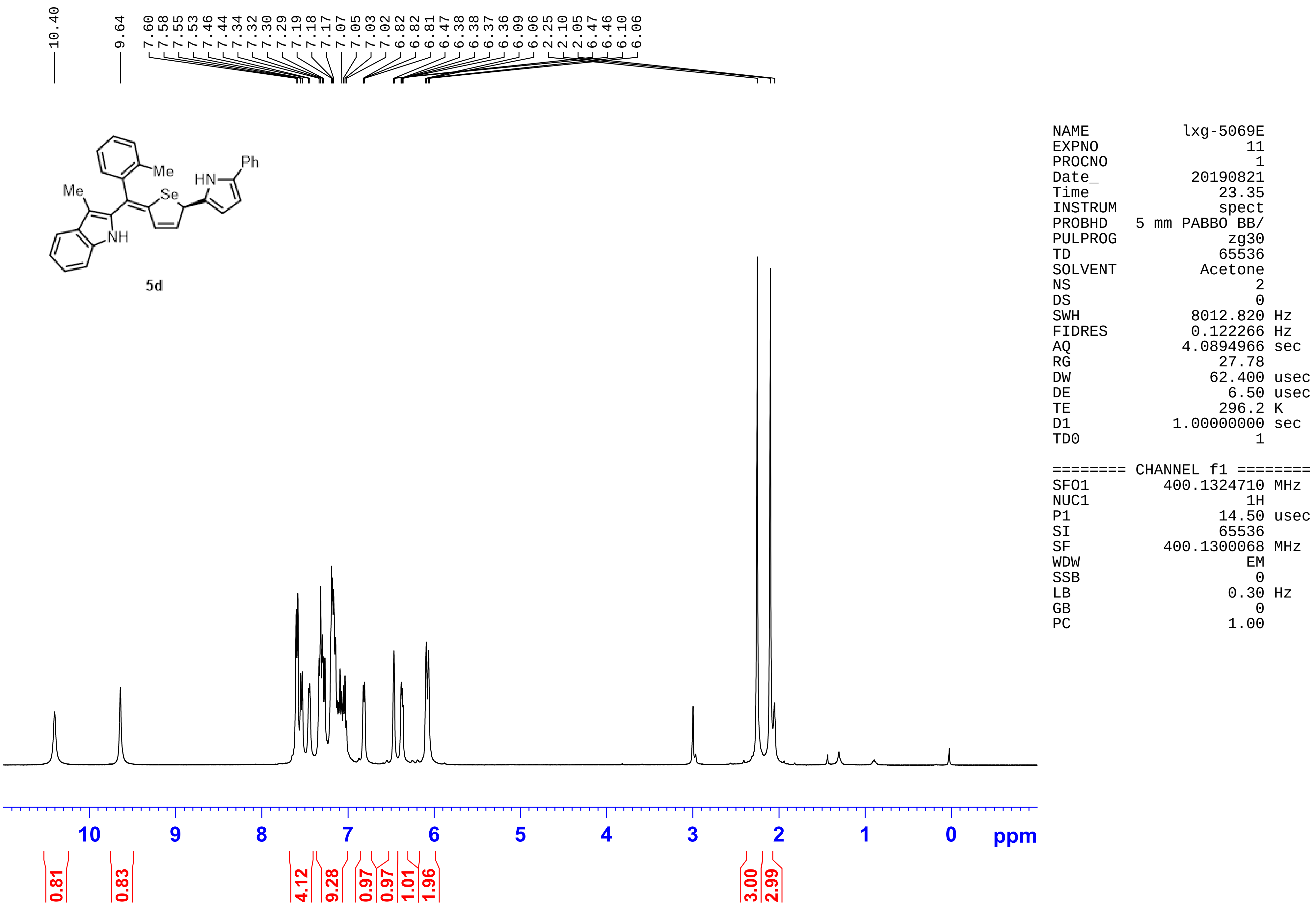

Supplementary Figure 119. ^{1}H NMR spectrum of **3d**

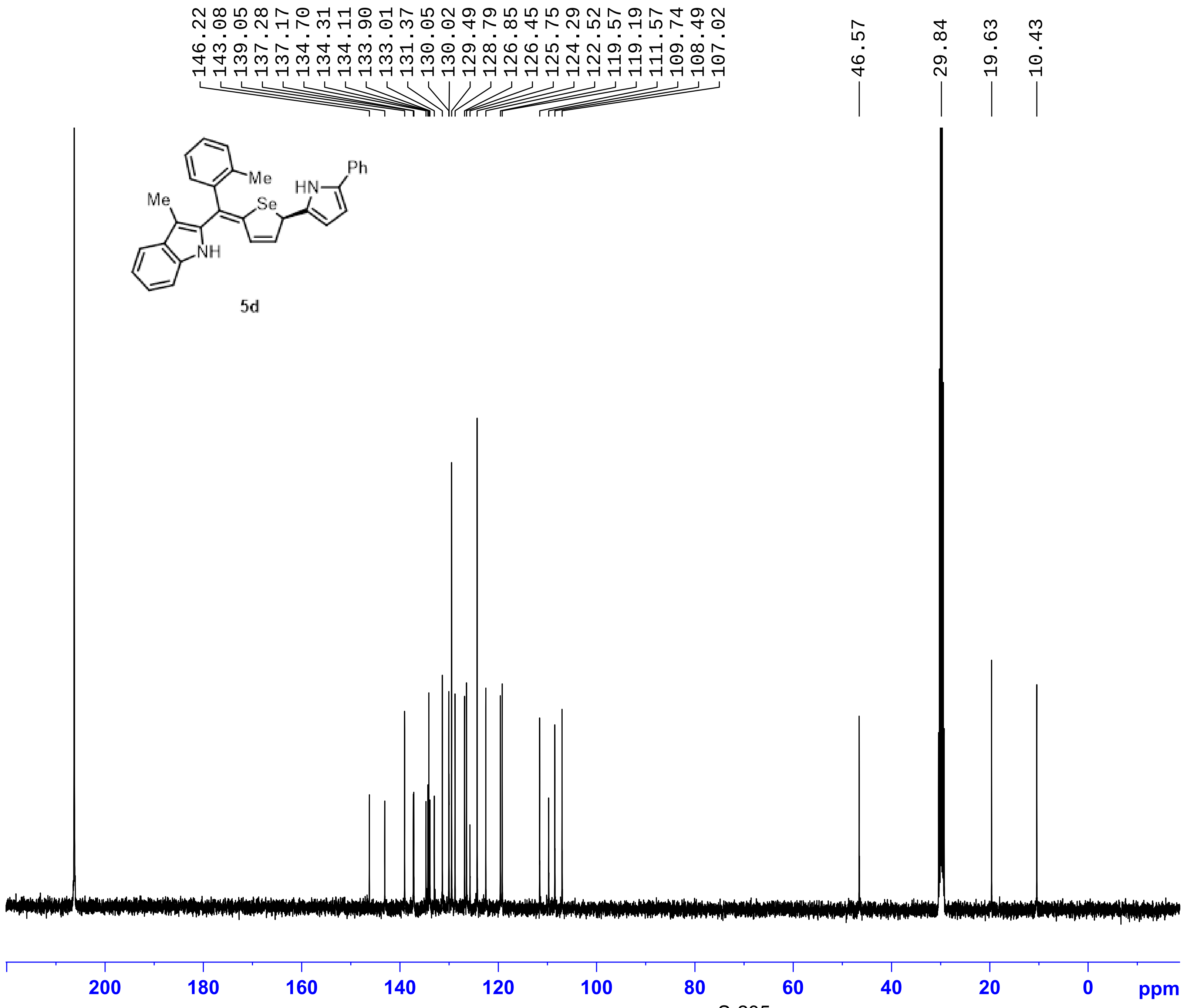

NAME	lxg-5069E
EXPNO	12
PROCNO	1
Date_	20190821
Time	23.38
INSTRUM	spect
PROBHD	5 mm PABBO BB/
PULPROG	zgpg30
TD	65536
SOLVENT	Acetone
NS	100
DS	0
SWH	24038.461 Hz
FIDRES	0.366798 Hz
AQ	1.3631988 sec
RG	196.92
DW	20.800 usec
DE	6.50 usec
TE	297.0 K
D1	2.00000000 sec
D11	0.03000000 sec
TD0	1

======== CHANNEL f1 ========

SFO1	100.6228298 MHz
NUC1	13C
P1	9.70 usec
SI	32768
SF	100.6126893 MHz
WDW	EM
SSB	0
LB	1.00 Hz
GB	0
PC	1.40

Supplementary Figure 119. ^{13}C NMR spectrum of **5d**

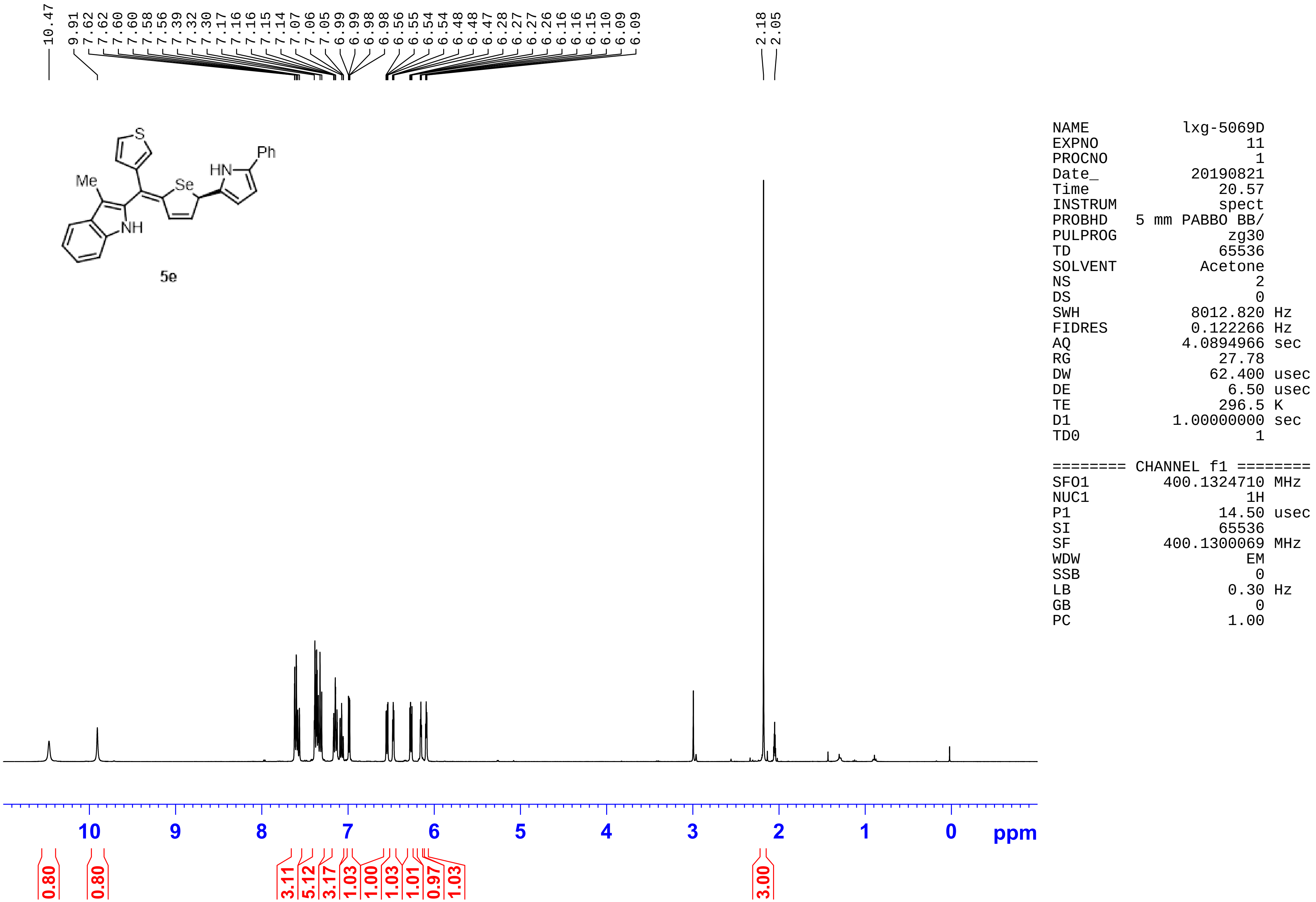

Supplementary Figure 121. ^{1}H NMR spectrum of **5e**

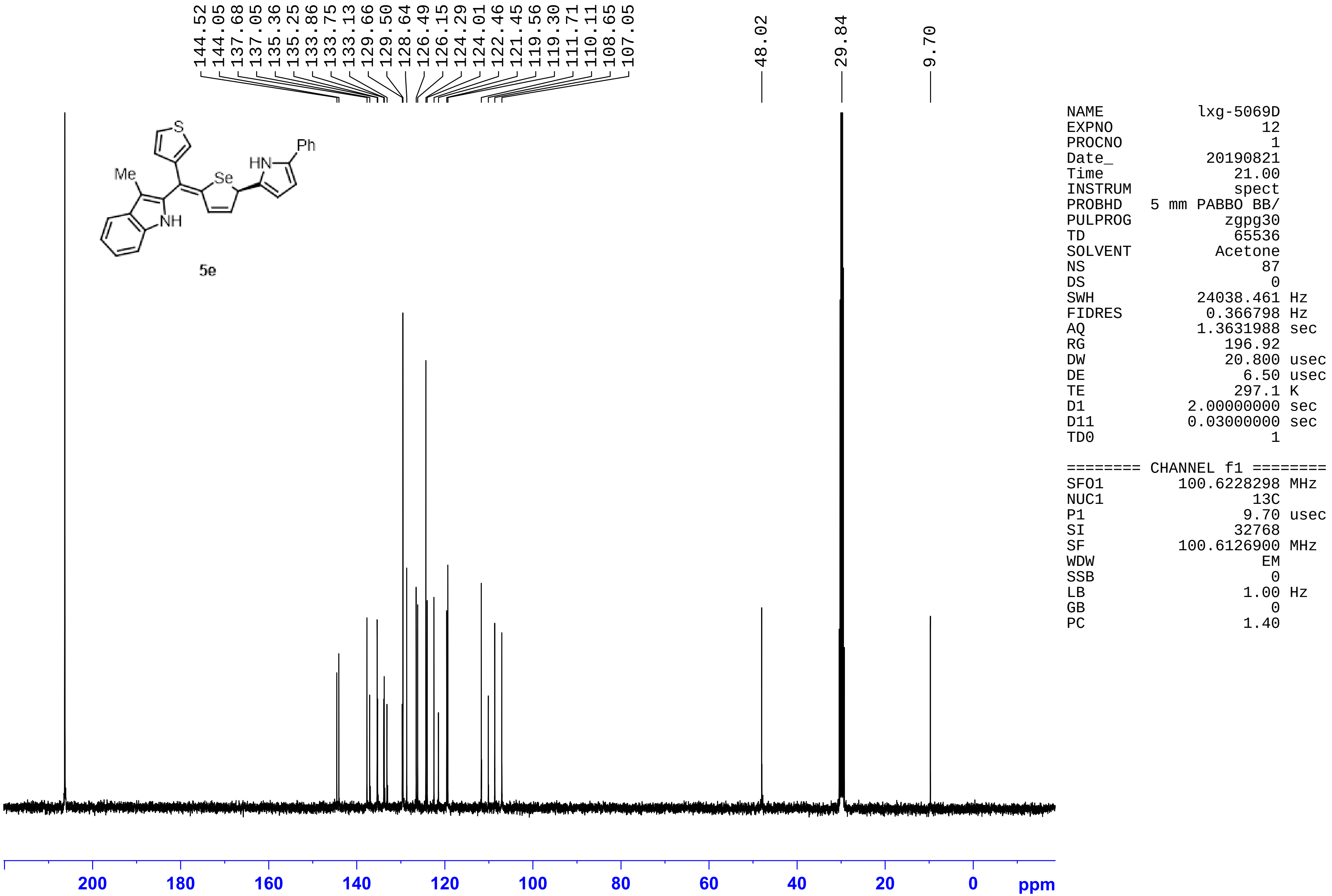

Supplementary Figure 122. ^{13}C NMR spectrum of **5e**

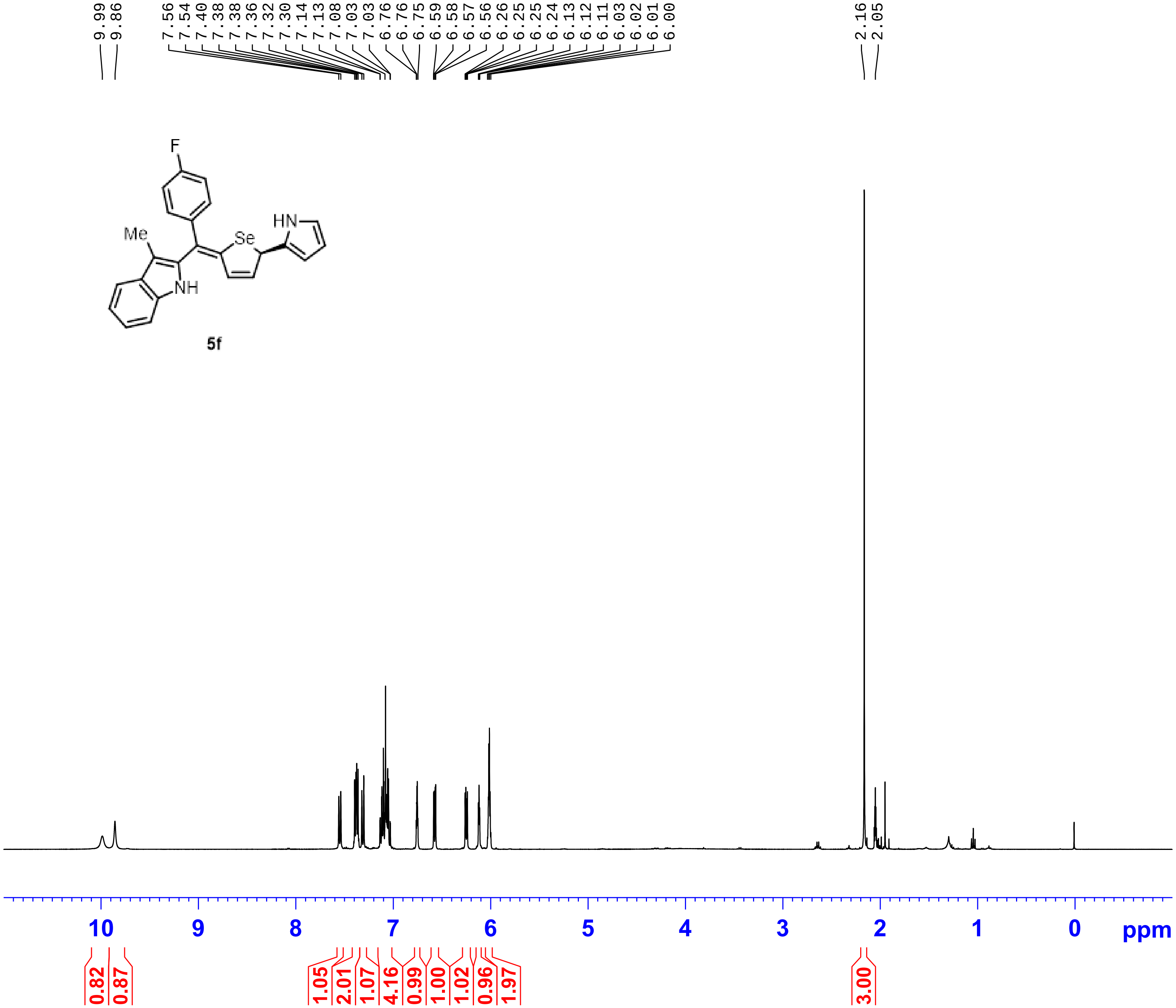

NAME	lxg-7081A	
EXPNO	1	
PROCNO	1	
Date_	20200704	
Time	14.17	
INSTRUM	spect	
PROBHD	5 mm PABBO BB/	
PULPROG	zg30	
TD	65536	
SOLVENT	Acetone	
NS	3	
DS	0	
SWH	8012.820	Hz
FIDRES	0.122266	Hz
AQ	4.0894966	sec
RG	39.46	
DW	62.400	usec
DE	6.50	usec
TE	296.6	K
D1	1.00000000	sec
TD0	1	

======== CHANNEL f1 ========

SFO1	400.1324710	MHz
NUC1	1H	
P1	14.50	usec
SI	65536	
SF	400.1300069	MHz
WDW	EM	
SSB	0	
LB	0.30	Hz
GB	0	
PC	1.00	

Supplementary Figure 123. ^{1}H NMR spectrum of **5f**

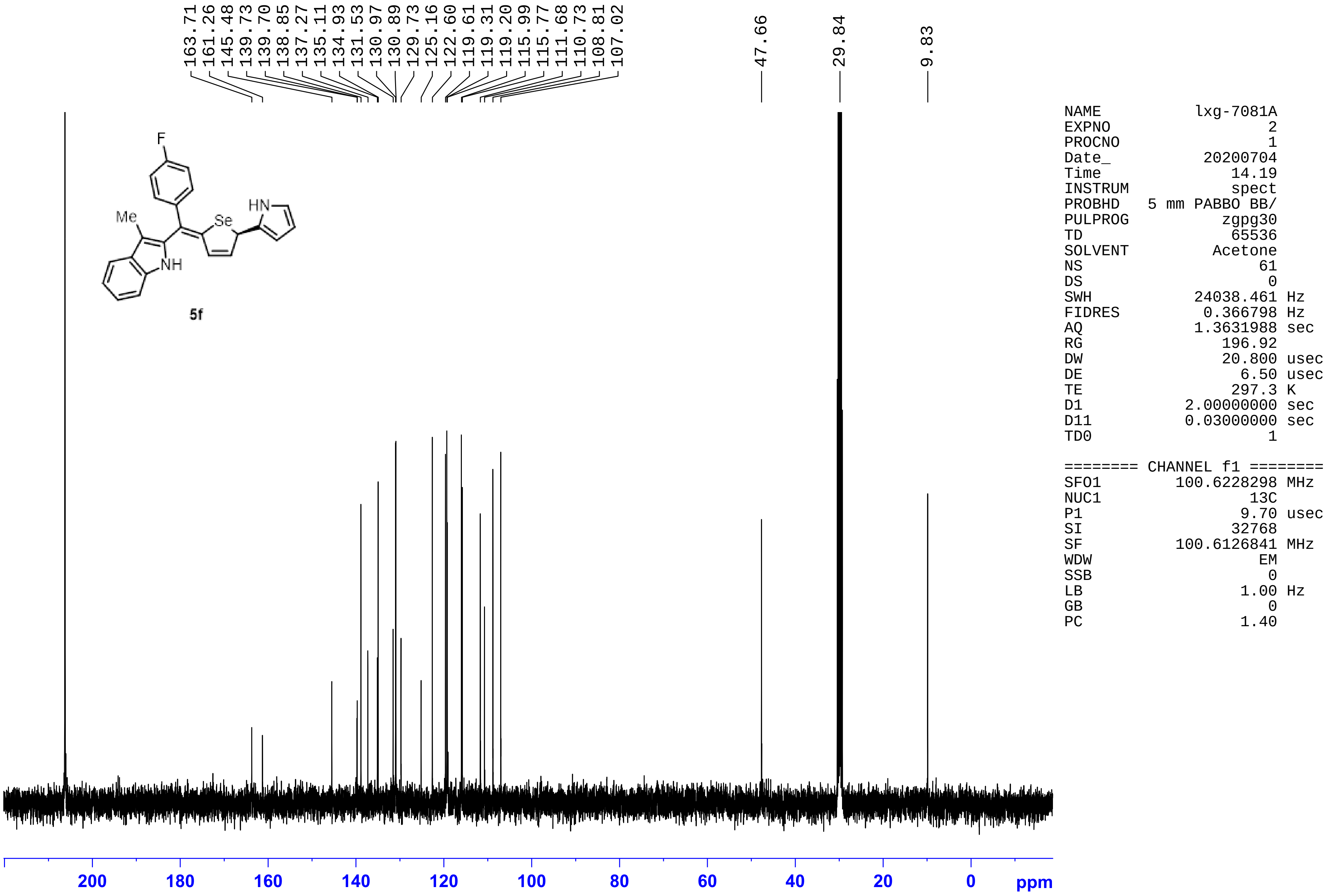

Supplementary Figure 124. ^{13}C NMR spectrum of **5f**

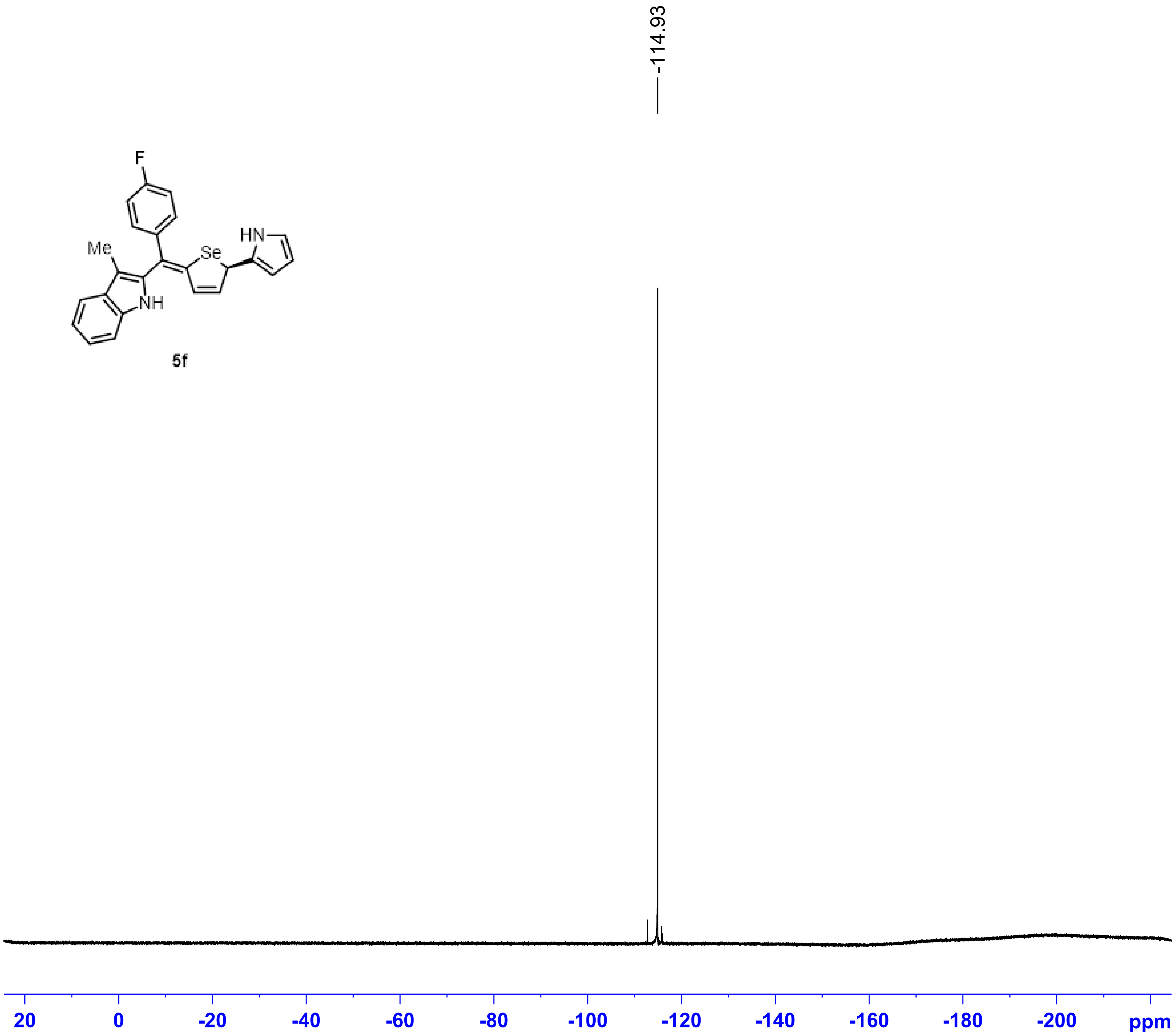

5f

NAME	lxg-7081A
EXPNO	11
PROCNO	1
Date_	20200724
Time	15.04
INSTRUM	spect
PROBHD	5 mm PABBO BB/
PULPROG	zgpg30
TD	65536
SOLVENT	Acetone
NS	16
DS	0
SWH	93750.000 Hz
FIDRES	1.430511 Hz
AQ	0.3495753 sec
RG	196.92
DW	5.333 usec
DE	6.50 usec
TE	297.1 K
D1	2.00000000 sec
D11	0.03000000 sec
TD0	1

======== CHANNEL f1 ========

SFO1	376.4607162 MHz
NUC1	19F
P1	14.70 usec
SI	32768
SF	376.4983660 MHz
WDW	EM
SSB	0
LB	1.00 Hz
GB	0
PC	1.40

Supplementary Figure 125. ^{19}F NMR spectrum of **5f**

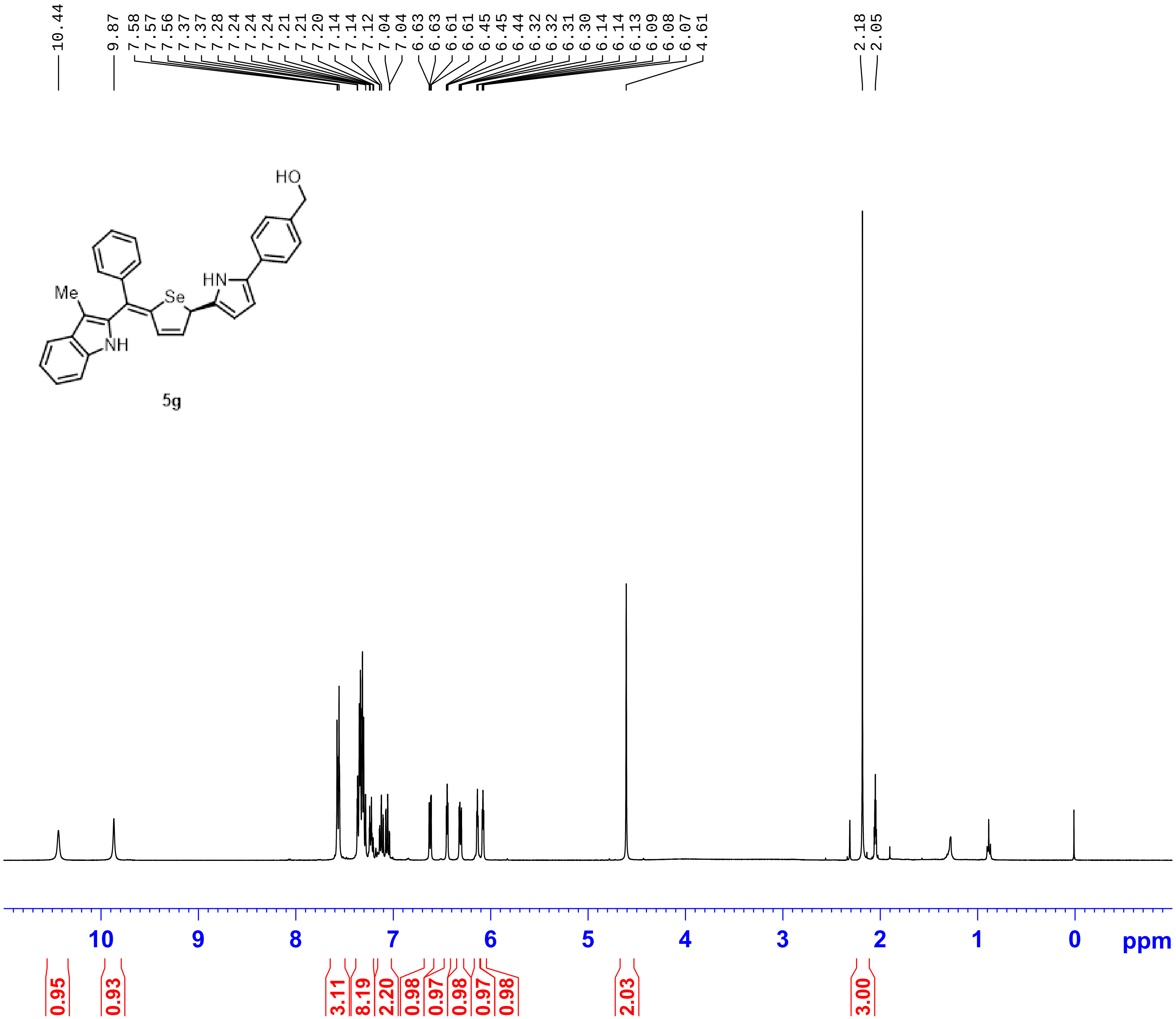

NAME	lxg-5074B-
EXPNO	2
PROCNO	1
Date_	20190824
Time	14.10
INSTRUM	spect
PROBHD	5 mm PABBO BB/
PULPROG	zg30
TD	65536
SOLVENT	Acetone
NS	3
DS	0
SWH	8012.820 Hz
FIDRES	0.122266 Hz
AQ	4.0894966 sec
RG	54.81
DW	62.400 usec
DE	6.50 usec
TE	296.7 K
D1	1.00000000 sec
TD0	1

======== CHANNEL f1 ========

SFO1	400.1324710 MHz
NUC1	1H
P1	14.50 usec
SI	65536
SF	400.1300069 MHz
WDW	EM
SSB	0
LB	0.30 Hz
GB	0
PC	1.00

Supplementary Figure 126. ^{1}H NMR spectrum of **5g**

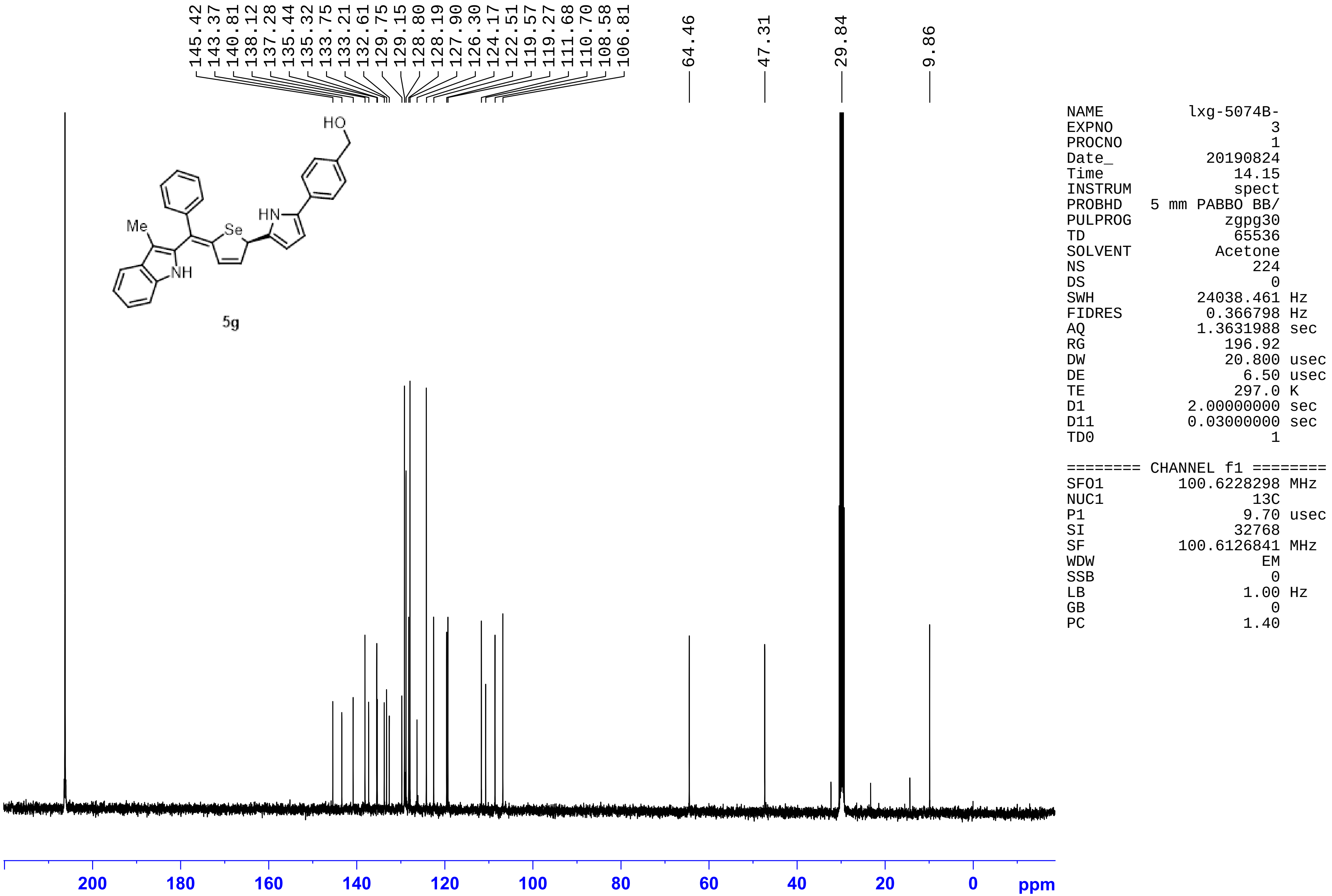

Supplementary Figure 127. ^{13}C NMR spectrum of **5g**

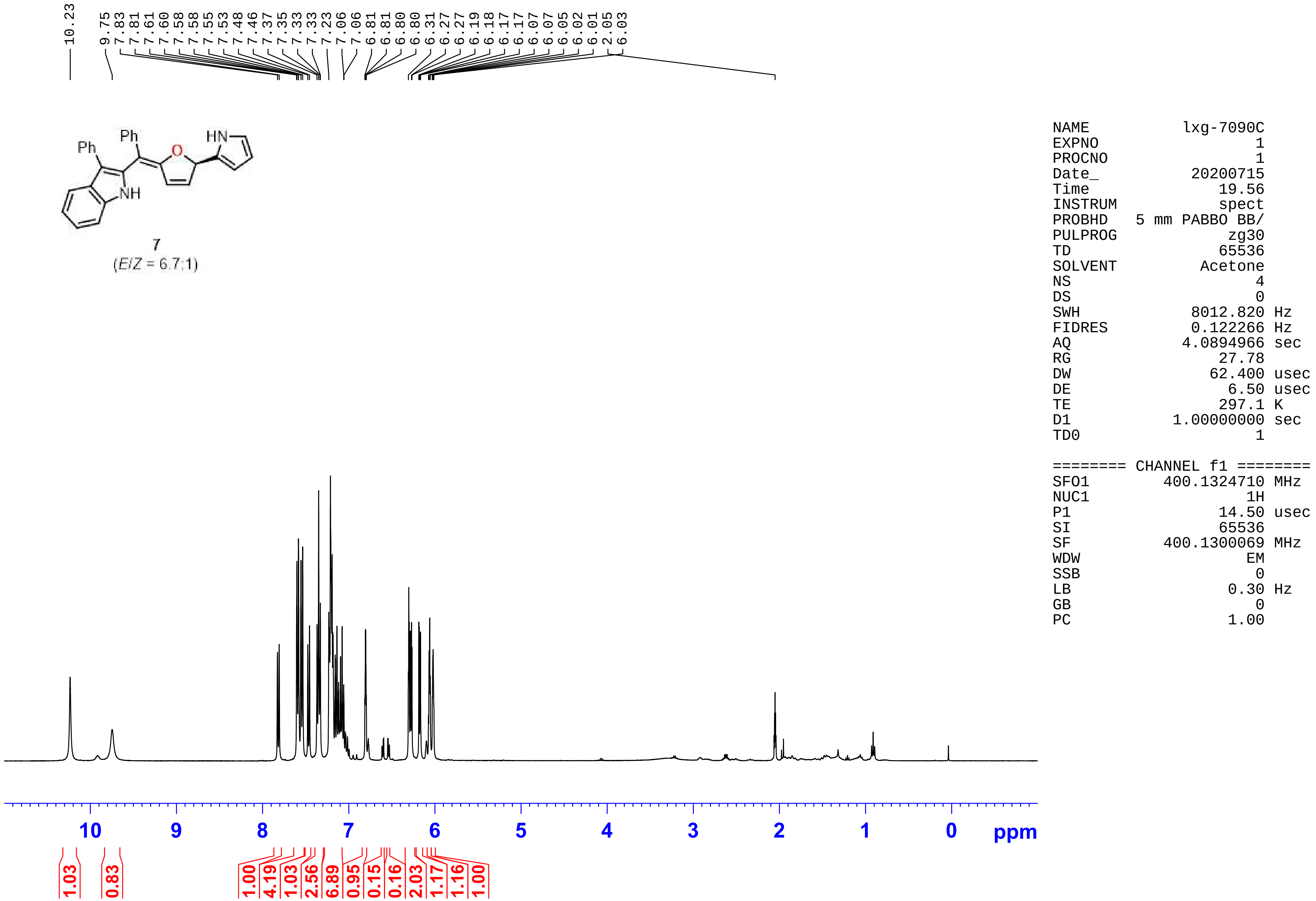

Supplementary Figure 128. ^1H NMR spectrum of **7**

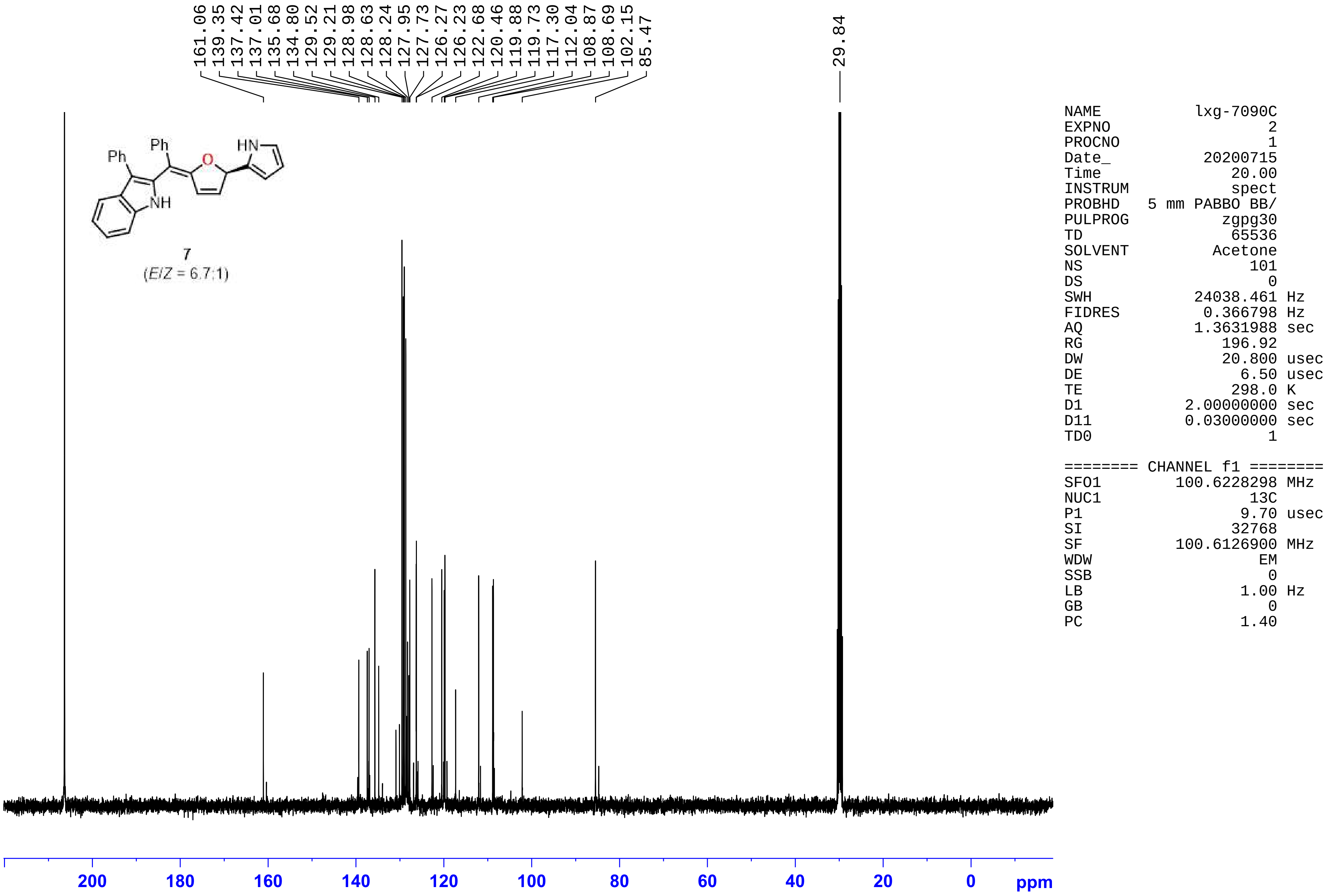

Supplementary Figure 129. ^{13}C NMR spectrum of **7**

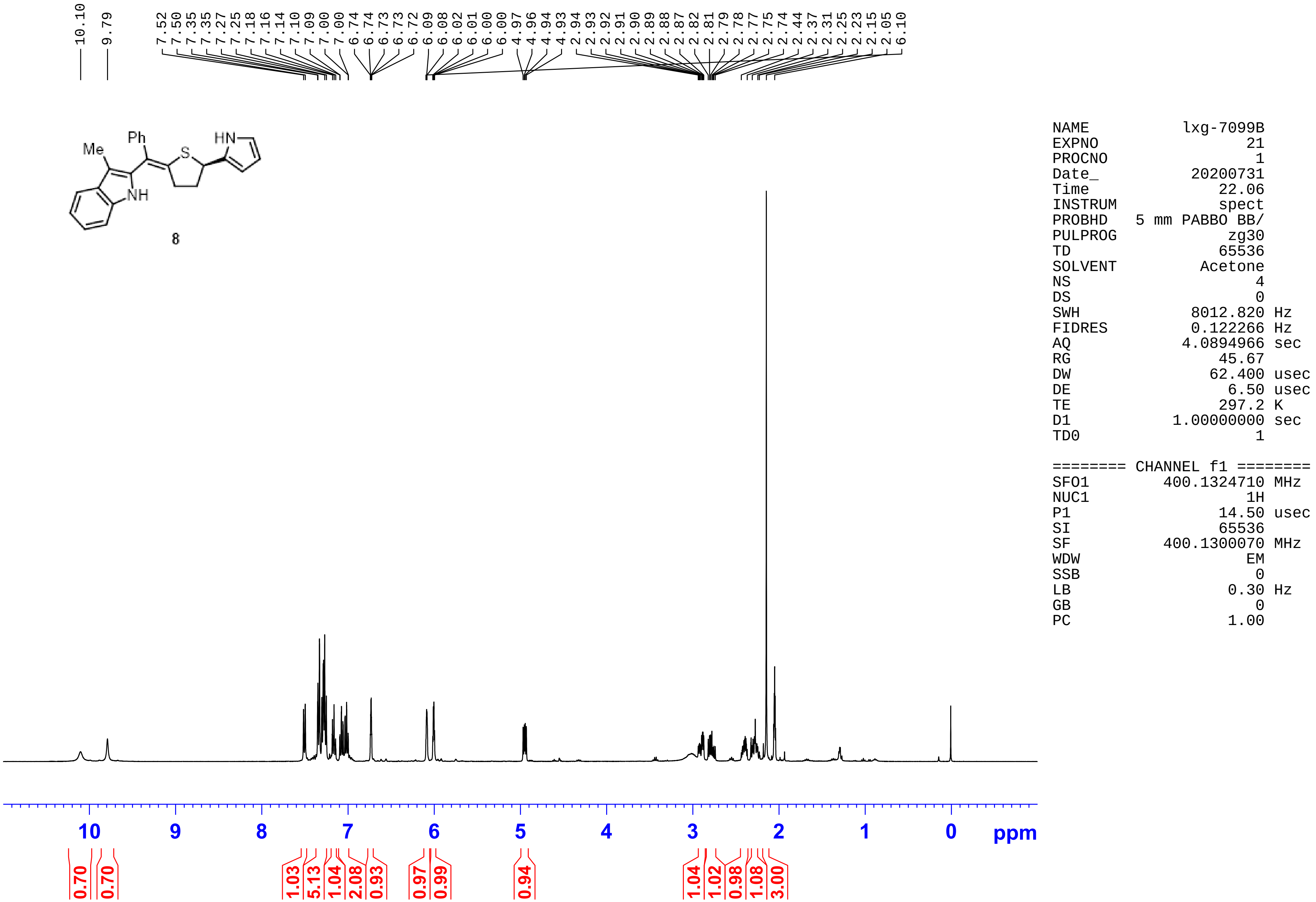

Supplementary Figure 13. ^{1}H NMR spectrum of **8**

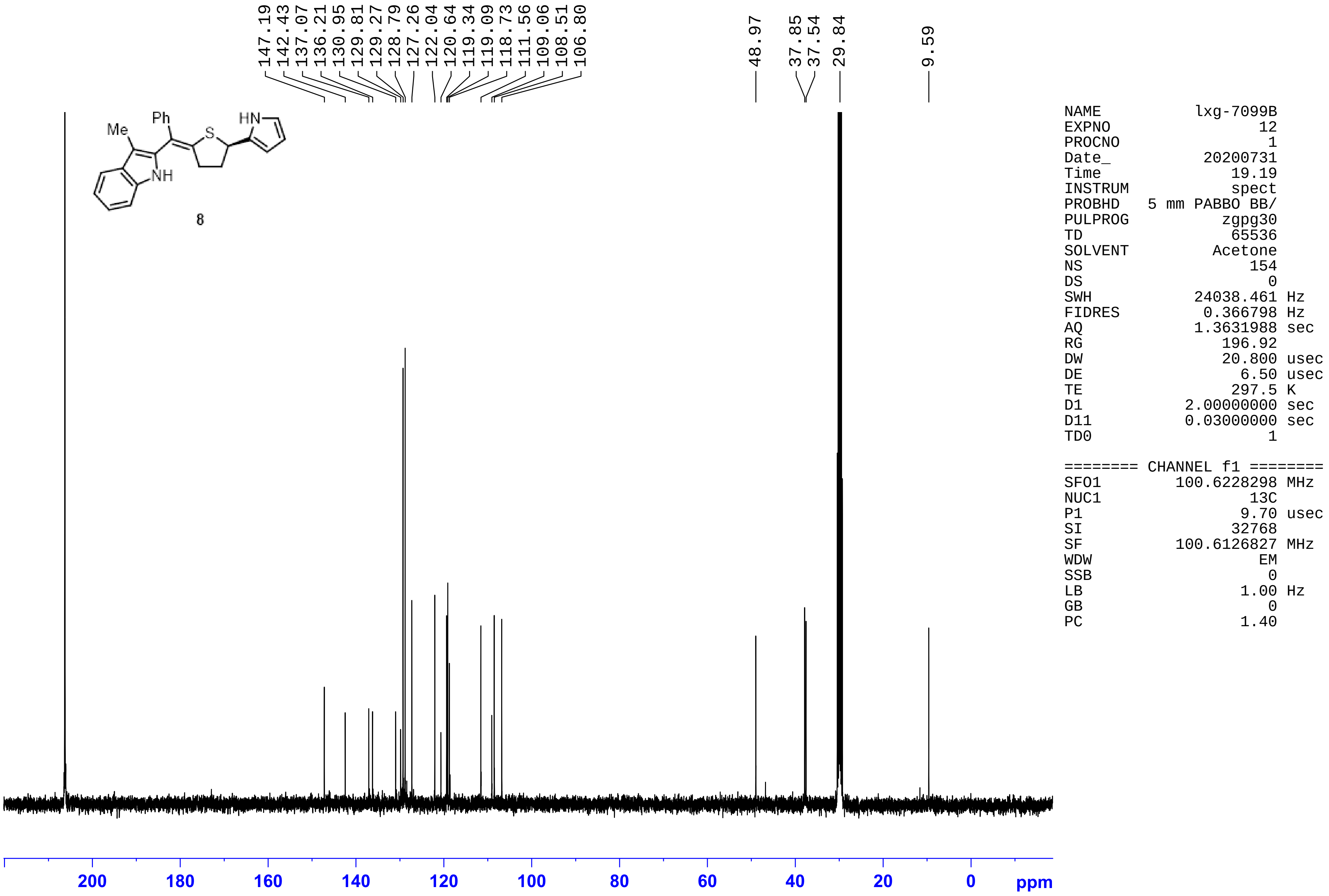

Supplementary Figure 131. ^{13}C NMR spectrum of **8**

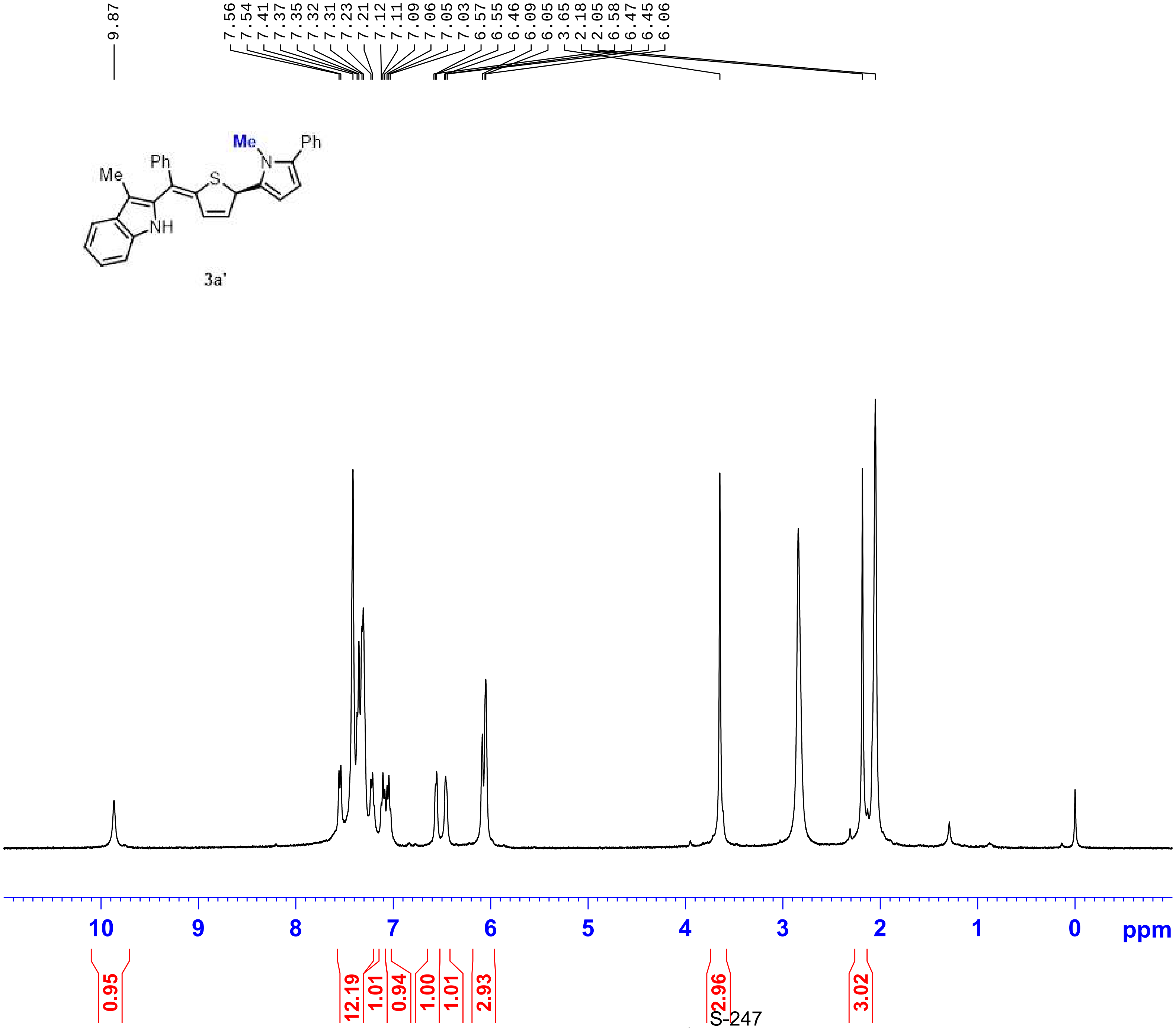

NAME lxg-7092A
EXPNO 12
PROCNO 1
Date_ 20200719
Time 22.45
INSTRUM spect
PROBHD 5 mm PABBO BB/
PULPROG zg30
TD 65536
SOLVENT Acetone
NS 6
DS 0
SWH 8012.820 Hz
FIDRES 0.122266 Hz
AQ 4.0894966 sec
RG 88.84
DW 62.400 usec
DE 6.50 usec
TE 298.8 K
D1 1.00000000 sec
TD0 1

======== CHANNEL f1 ========
SFO1 400.1324710 MHz
NUC1 1H
P1 14.50 usec
SI 65536
SF 400.1300076 MHz
WDW EM
SSB 0
LB 0.30 Hz
GB 0
PC 1.00

Supplementary Figure 132. ^{1}H NMR spectrum of **3a'**

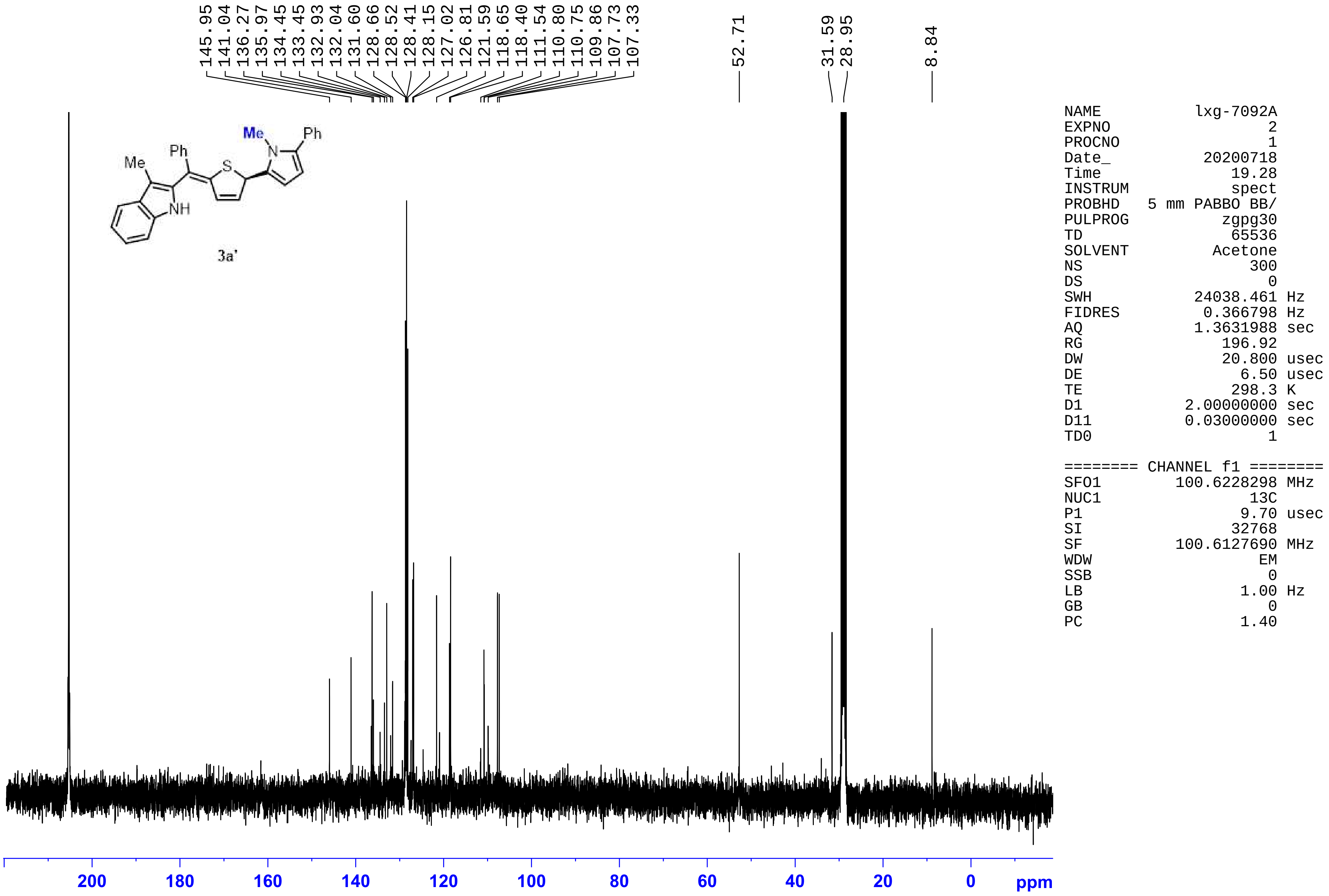

Supplementary Figure 133. ^{13}C NMR spectrum of **3a'**

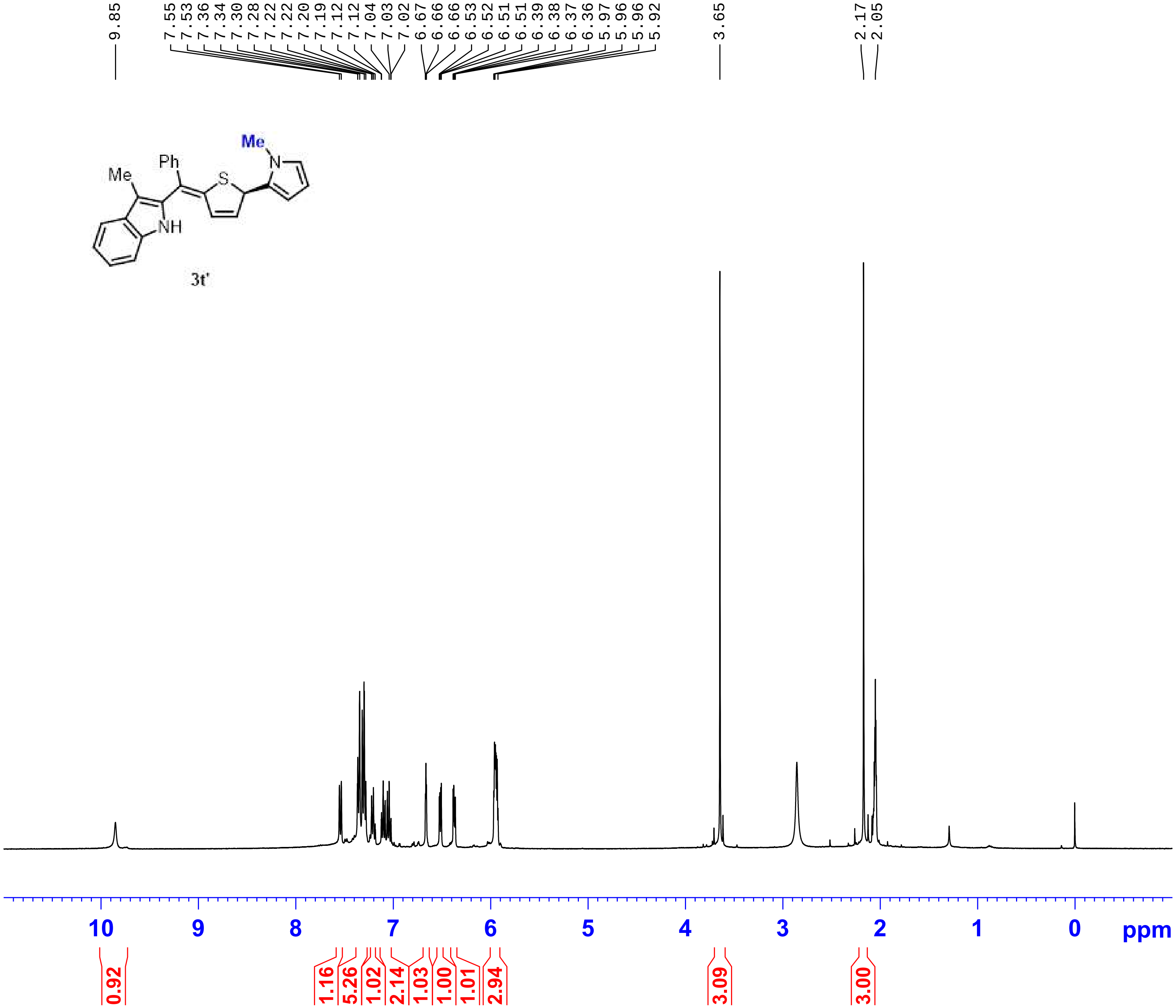

3t'

NAME	lxg-7092B
EXPNO	1
PROCNO	1
Date_	20200718
Time	19.53
INSTRUM	spect
PROBHD	5 mm PABBO BB/
PULPROG	zg30
TD	65536
SOLVENT	Acetone
NS	4
DS	0
SWH	8012.820 Hz
FIDRES	0.122266 Hz
AQ	4.0894966 sec
RG	103.52
DW	62.400 usec
DE	6.50 usec
TE	298.1 K
D1	1.00000000 sec
TD0	1

======== CHANNEL f1 ========

SFO1	400.1324710 MHz
NUC1	1H
P1	14.50 usec
SI	65536
SF	400.1300070 MHz
WDW	EM
SSB	0
LB	0.30 Hz
GB	0
PC	1.00

Supplementary Figure 134. ^{1}H NMR spectrum of **3t'**

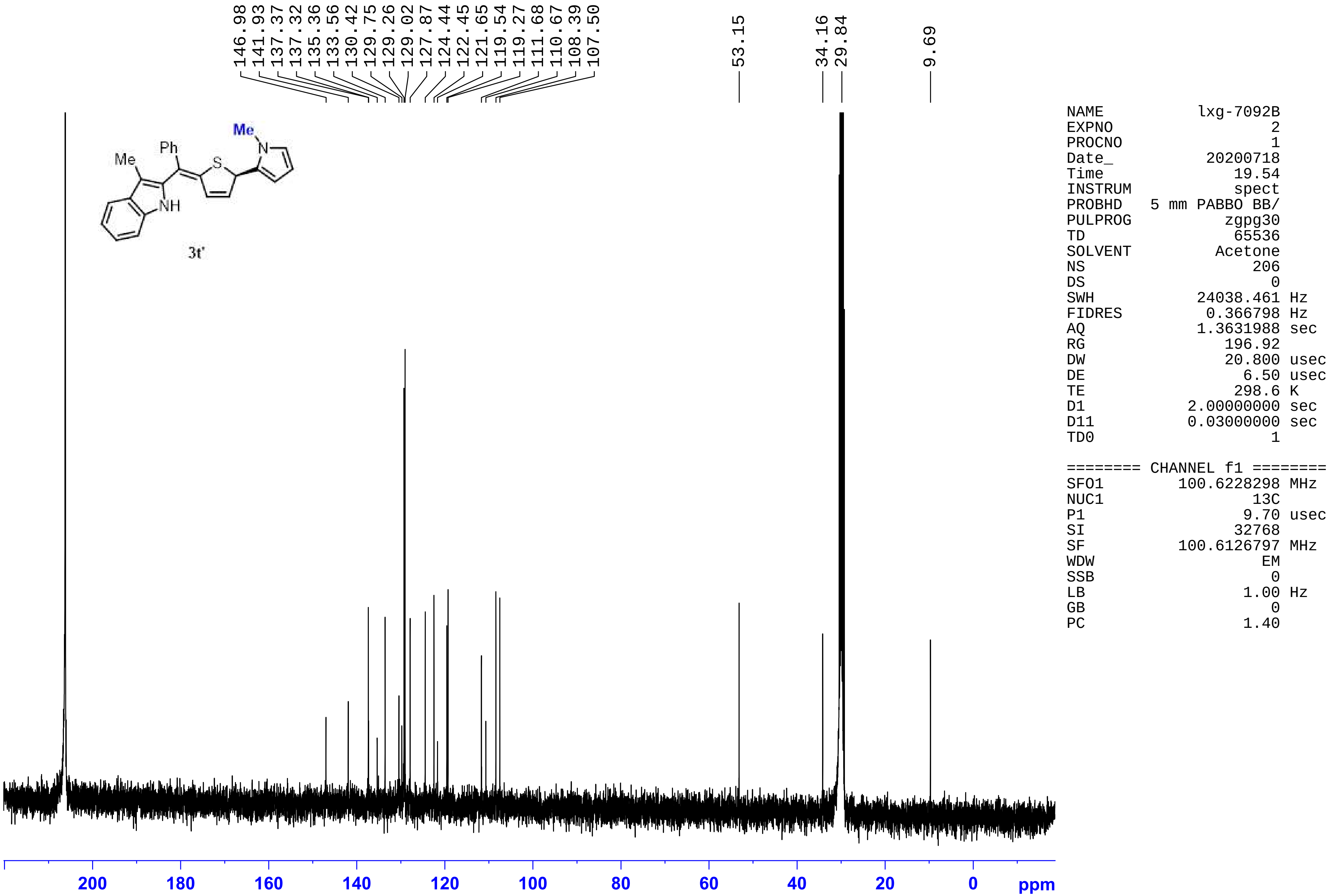

Supplementary Figure 135. ^{13}C NMR spectrum of **3t'**

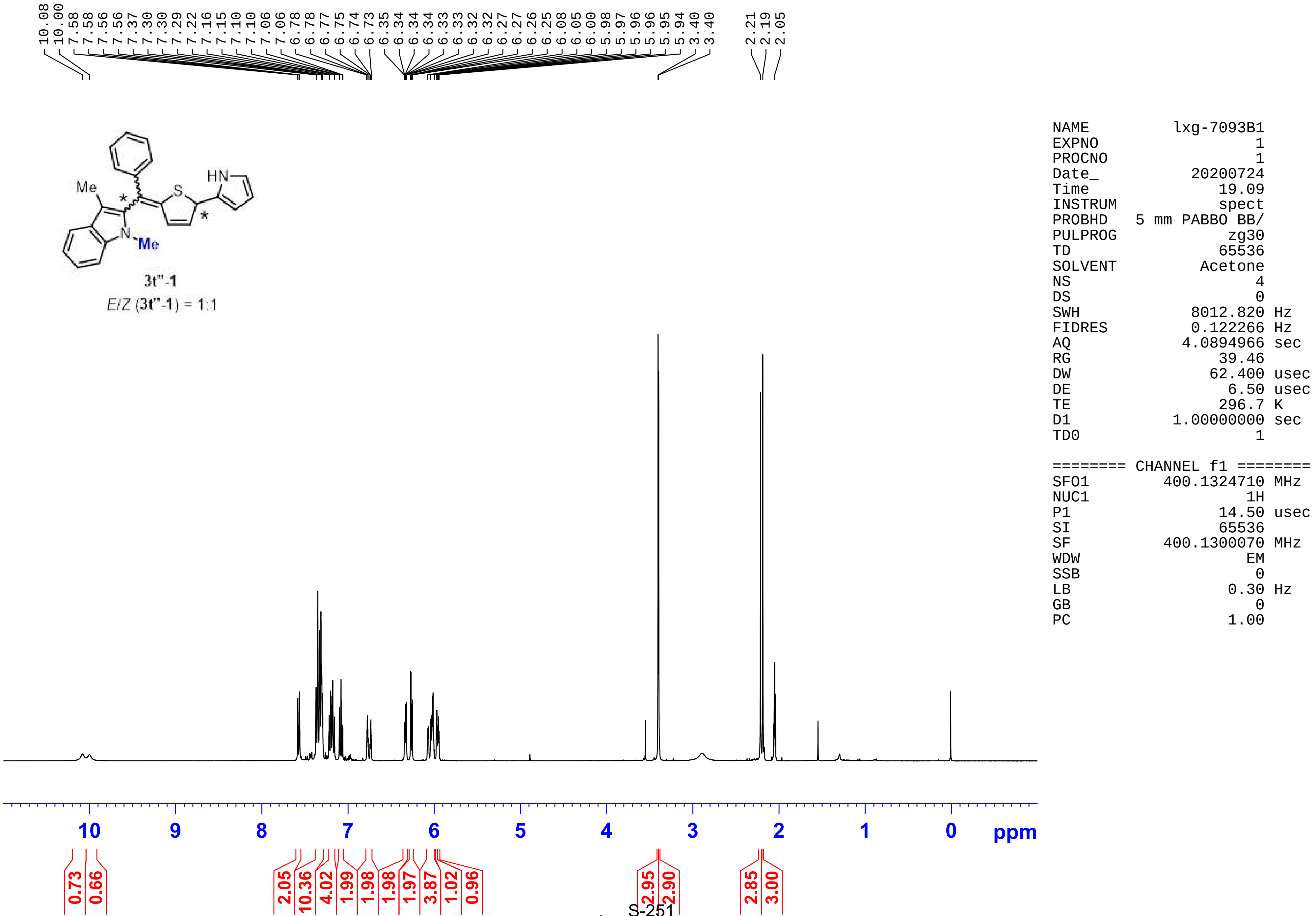

Supplementary Figure 136. ^{1}H NMR spectrum of **3t''-1**

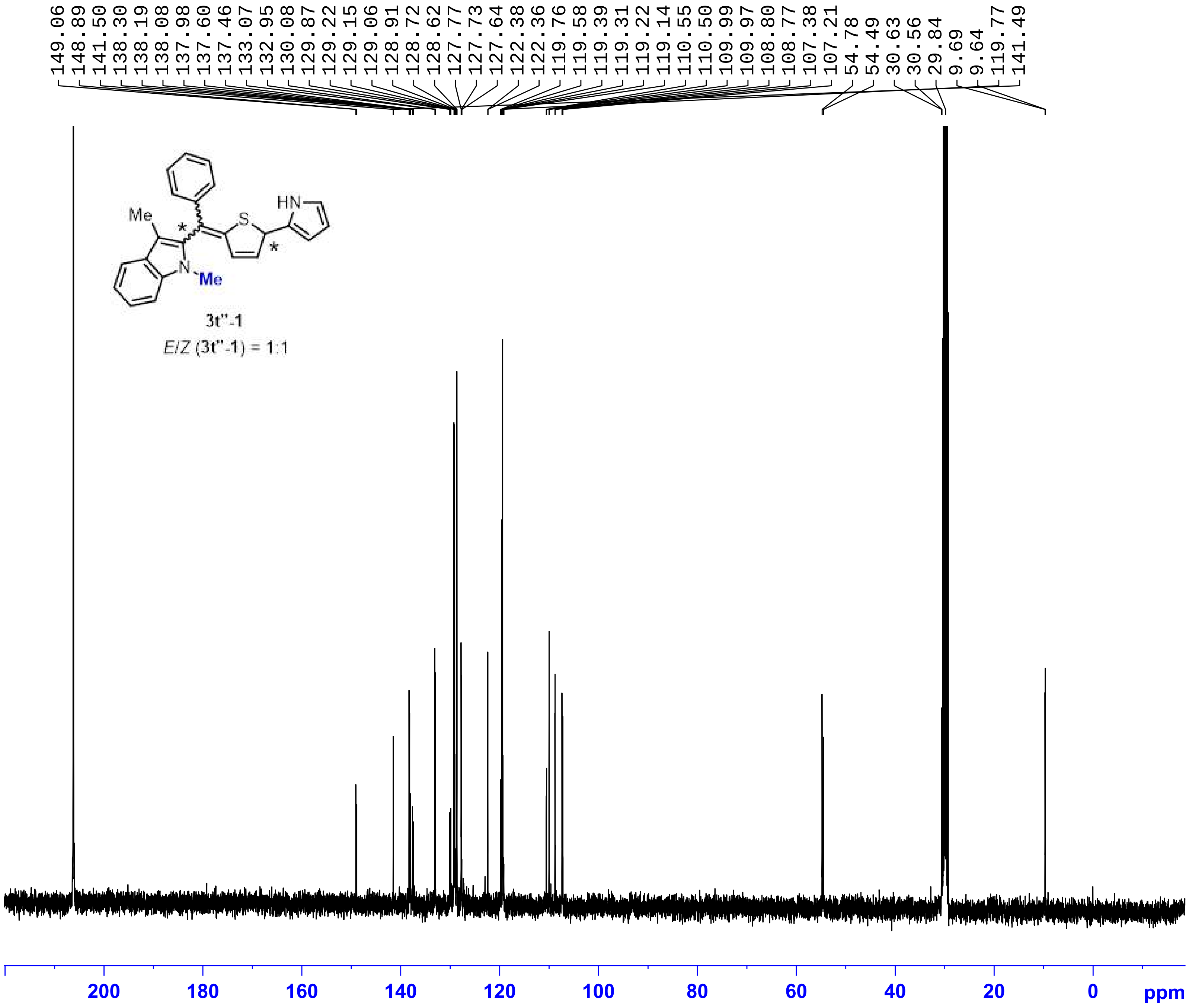

NAME lxg-7093B1
EXPNO 2
PROCNO 1
Date_ 20200724
Time 19.12
INSTRUM spect
PROBHD 5 mm PABBO BB/
PULPROG zgpg30
TD 65536
SOLVENT Acetone
NS 198
DS 0
SWH 24038.461 Hz
FIDRES 0.366798 Hz
AQ 1.3631988 sec
RG 196.92
DW 20.800 usec
DE 6.50 usec
TE 297.5 K
D1 2.00000000 sec
D11 0.03000000 sec
TD0 1

======== CHANNEL f1 ========
SFO1 100.6228298 MHz
NUC1 13C
P1 9.70 usec
SI 32768
SF 100.6126827 MHz
WDW EM
SSB 0
LB 1.00 Hz
GB 0
PC 1.40

Supplementary Figure 137. ^{13}C NMR spectrum of **3t''-1**

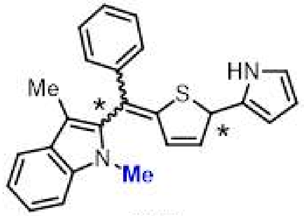

3t''-2

E/Z (3t''-2) = 1:1

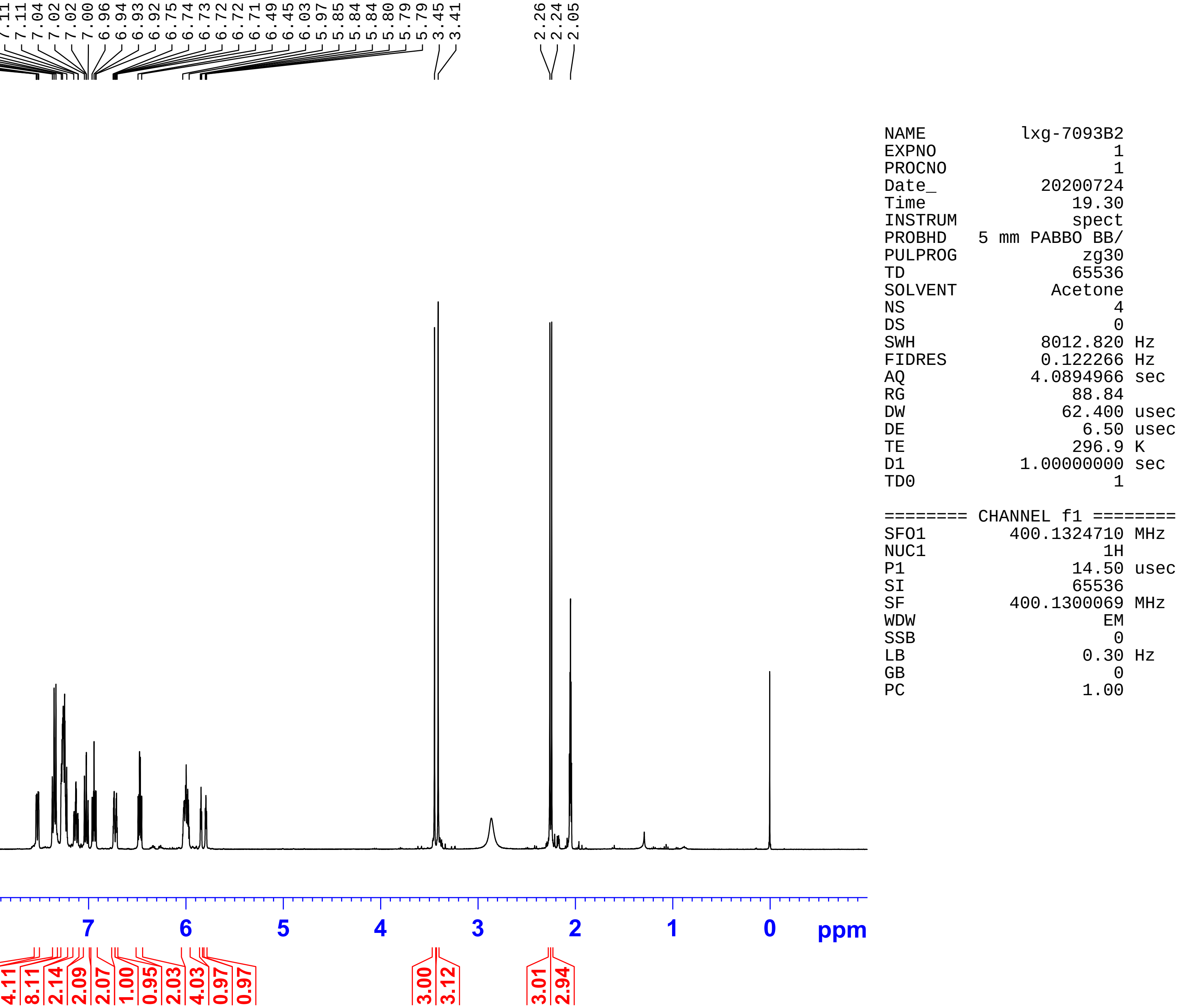

Supplementary Figure 138. ^{1}H NMR spectrum of **3t''-2**

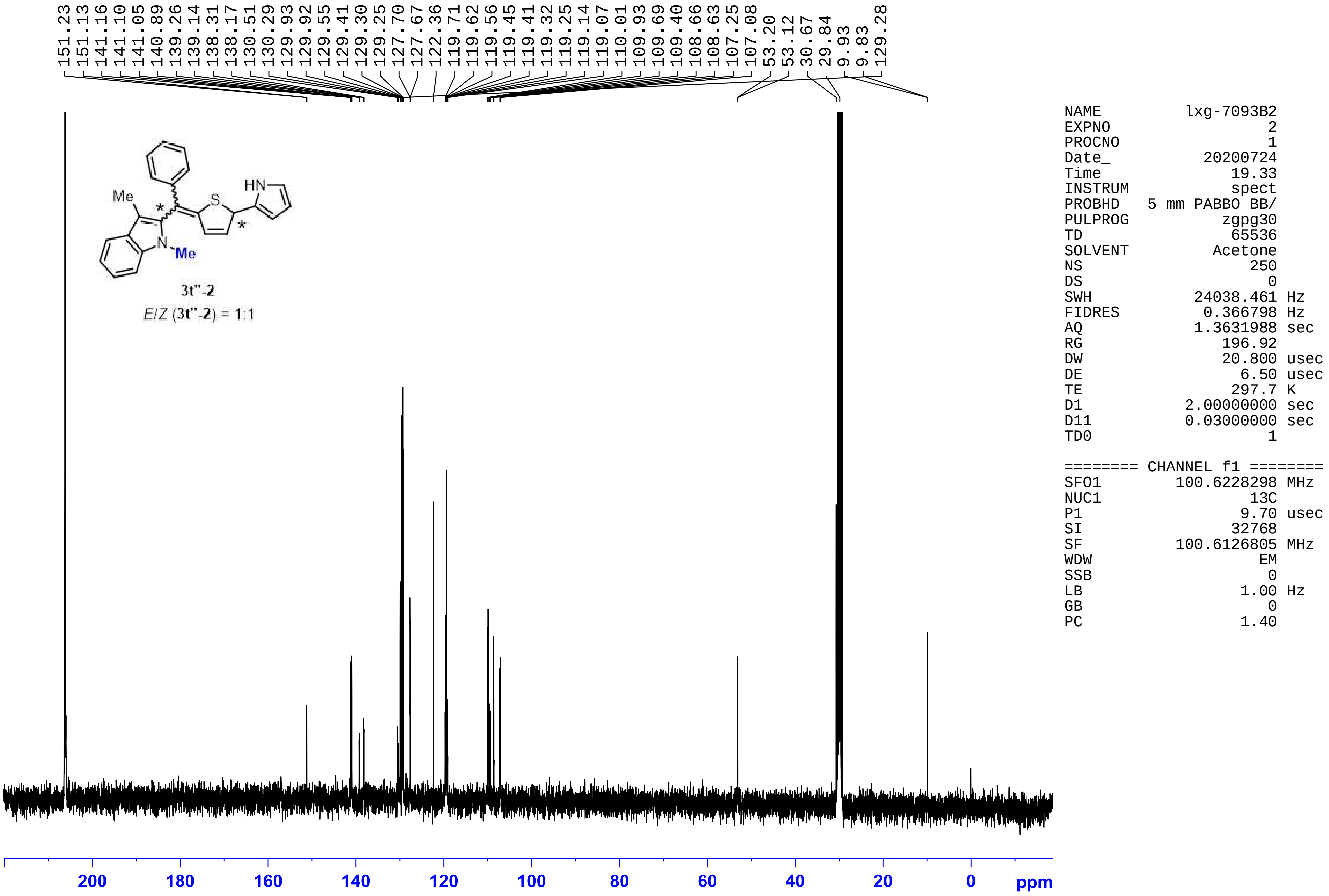

Supplementary Figure 139. ^{13}C NMR spectrum of **3t''-2**

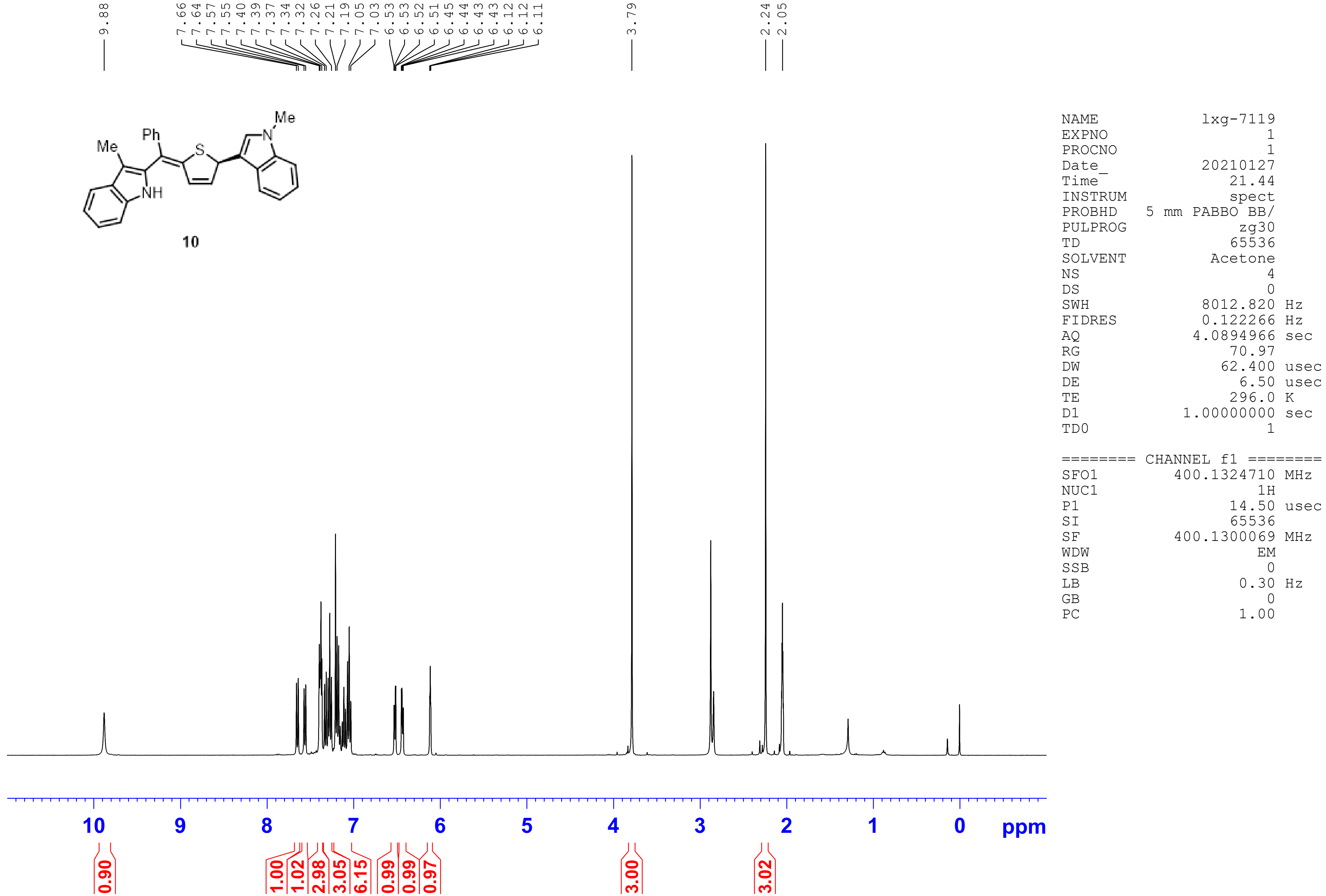

Supplementary Figure 140. ^{1}H NMR spectrum of **10**

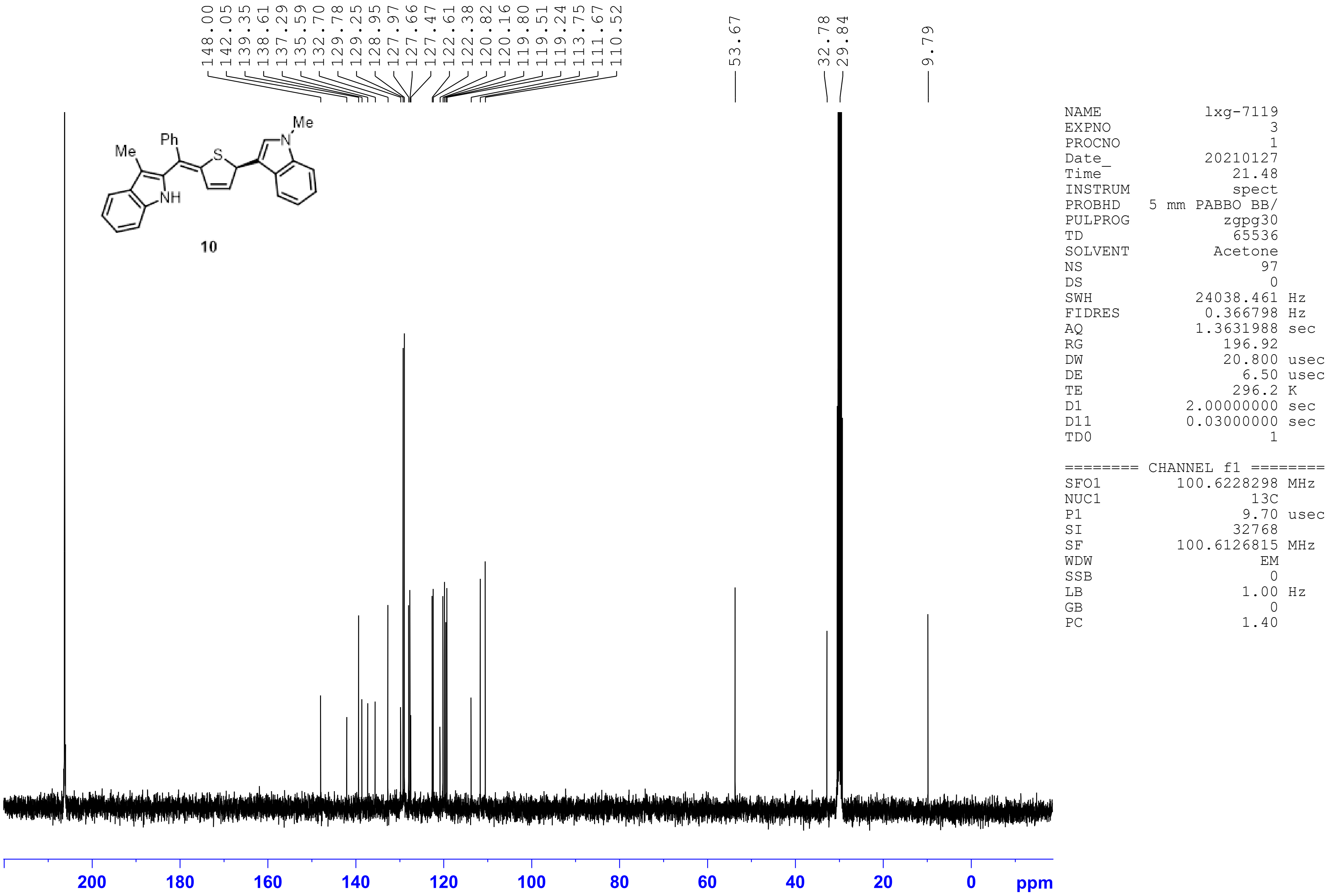

Supplementary Figure 141. ^{13}C NMR spectrum of **10**

Data File C:\CHEM32\1\DATA\QDY 2020-08-21 10-37-21\LXG-3157E-RAC.D
Sample Name:

```
=====================================================================
Acq. Operator   :                               Seq. Line :   2
Acq. Instrument : Instrument 1                   Location : Vial 62
Injection Date  : 8/21/2020 10:50:24 AM               Inj :   1
                                               Inj Volume : 5.000 µl
Different Inj Volume from Sequence !     Actual Inj Volume : 4.000 µl
Acq. Method     : C:\CHEM32\1\DATA\QDY 2020-08-21 10-37-21\OD-10-45.M
Last changed    : 1/4/2019 2:01:36 PM
Analysis Method : C:\CHEM32\1\METHODS\OD-01-45-0.5.M
Last changed    : 8/20/2020 10:26:25 PM
                  (modified after loading)
Additional Info : Peak(s) manually integrated
```

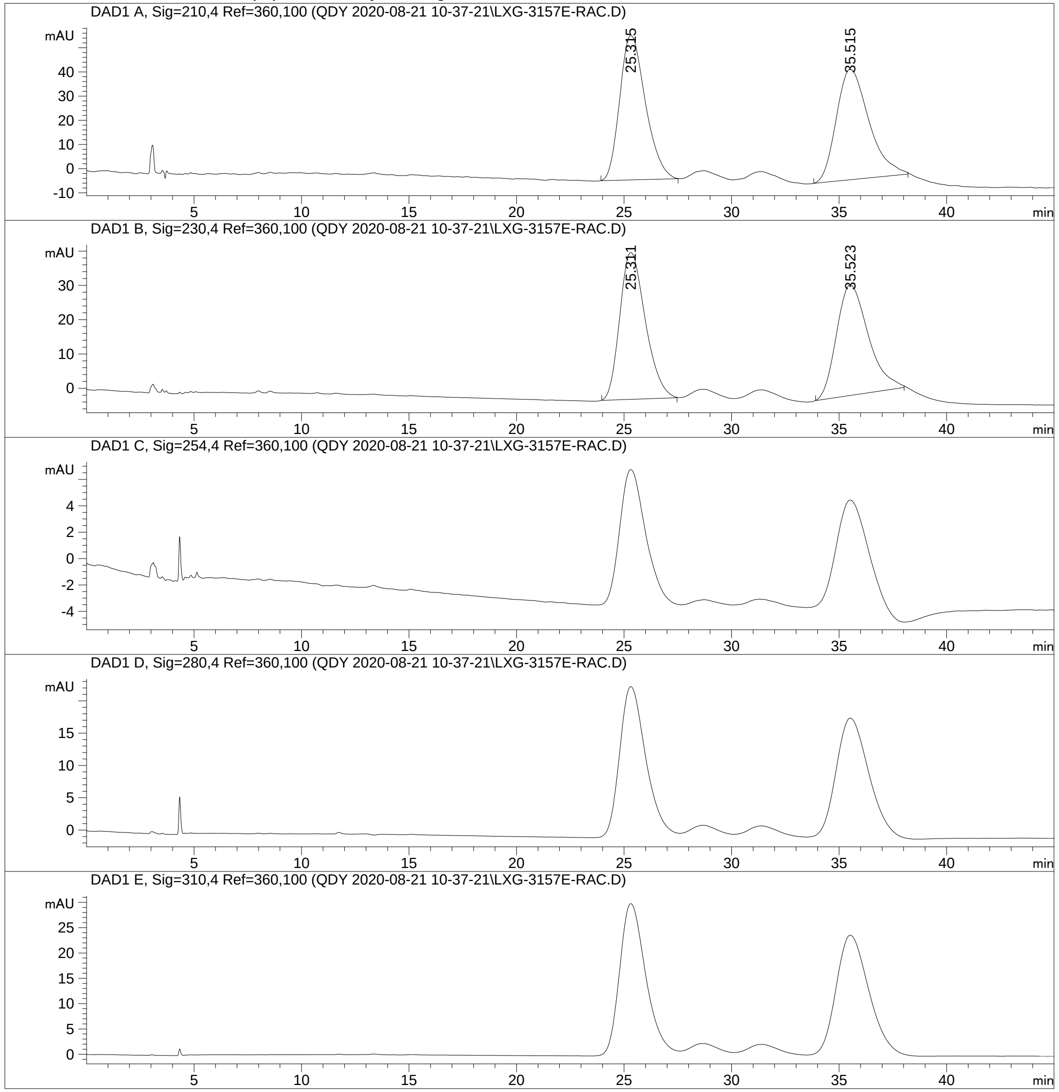

Data File C:\CHEM32\1\DATA\QDY 2020-08-21 10-37-21\LXG-3157E-RAC.D
Sample Name:

Area Percent Report

```
Sorted By             :      Signal
Multiplier            :      1.0000
Dilution              :      1.0000
Use Multiplier & Dilution Factor with ISTDs
```

Signal 1: DAD1 A, Sig=210,4 Ref=360,100

Peak #	RetTime [min]	Type	Width [min]	Area [mAU*s]	Height [mAU]	Area %
1	25.315	BB	1.1874	4961.68115	59.71409	50.7872
2	35.515	BB	1.3096	4807.87109	45.69316	49.2128

Totals : 9769.55225 105.40725

Signal 2: DAD1 B, Sig=230,4 Ref=360,100

Peak #	RetTime [min]	Type	Width [min]	Area [mAU*s]	Height [mAU]	Area %
1	25.311	BB	1.2761	3536.50269	42.79575	51.6442
2	35.523	BB	1.5276	3311.31250	32.20456	48.3558

Totals : 6847.81519 75.00031

Signal 3: DAD1 C, Sig=254,4 Ref=360,100

Signal 4: DAD1 D, Sig=280,4 Ref=360,100

Signal 5: DAD1 E, Sig=310,4 Ref=360,100

*** End of Report ***

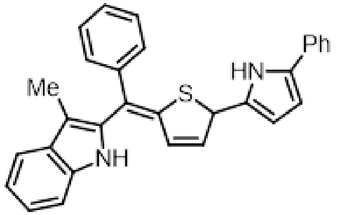

3a
(racemic)

Supplementary Figure 142. HPLC spectrum of **3a** (racemic)

```
=====================================================================
Acq. Operator   :                              Seq. Line :  23
Acq. Instrument : Instrument 1                  Location : Vial 78
Injection Date  : 5/11/2019 1:03:51 AM               Inj :   1
                                              Inj Volume : 5.000 µl
Acq. Method     : C:\CHEM32\1\DATA\QDY 2019-05-10 15-31-07\OD-10-45.M
Last changed    : 1/4/2019 2:01:36 PM
Analysis Method : C:\CHEM32\1\METHODS\AD-07-60.M
Last changed    : 5/10/2019 8:11:55 PM
Additional Info : Peak(s) manually integrated
```

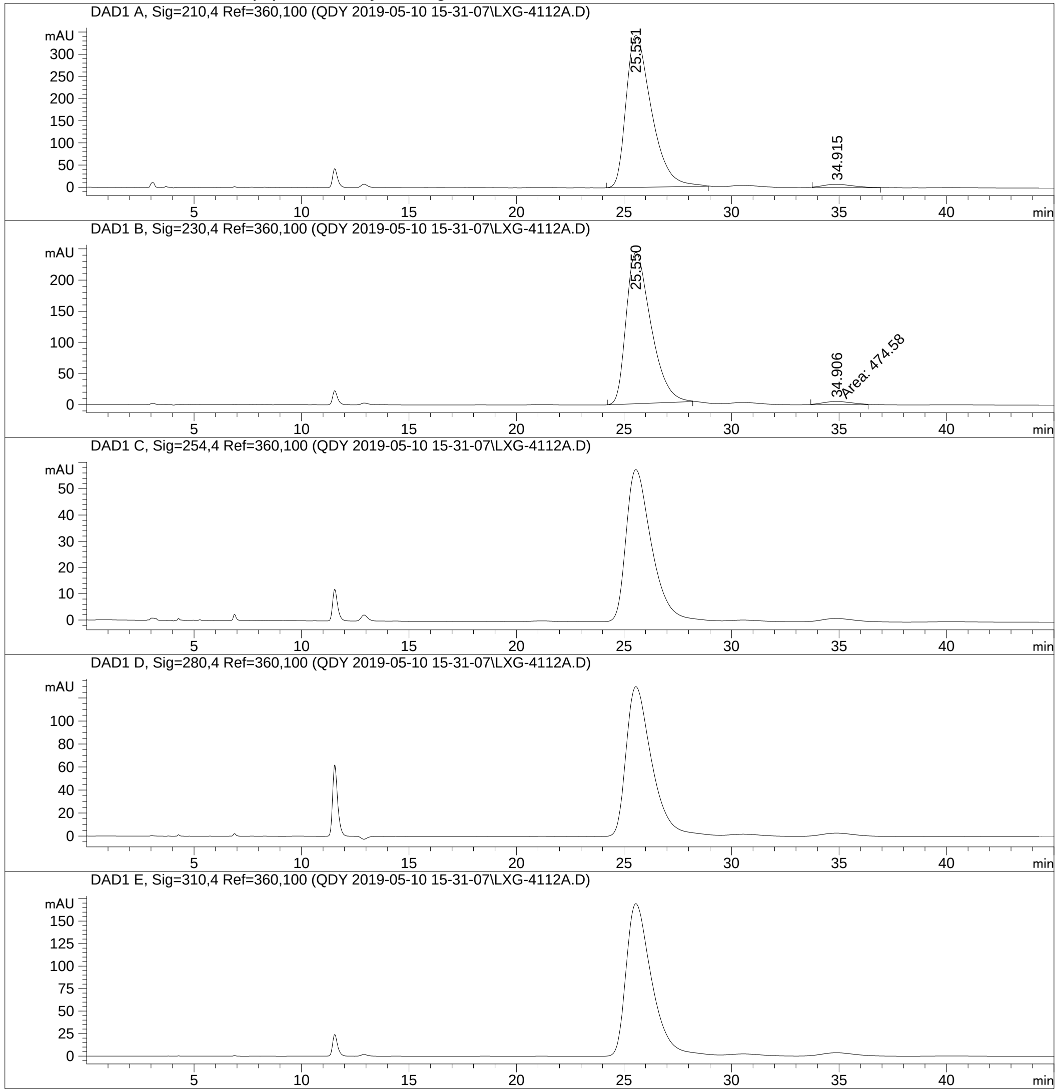

Data File C:\CHEM32\1\DATA\QDY 2019-05-10 15-31-07\LXG-4112A.D
Sample Name:

```
=====================================================================
                         Area Percent Report
=====================================================================

Sorted By             :      Signal
Multiplier            :      1.0000
Dilution              :      1.0000
Use Multiplier & Dilution Factor with ISTDs
```

Signal 1: DAD1 A, Sig=210,4 Ref=360,100

Peak #	RetTime [min]	Type	Width [min]	Area [mAU*s]	Height [mAU]	Area %
1	25.551	BB	1.2333	2.80972e4	342.43576	97.5087
2	34.915	BB	1.1400	717.87836	7.38589	2.4913

Totals : 2.88150e4 349.82165

Signal 2: DAD1 B, Sig=230,4 Ref=360,100

Peak #	RetTime [min]	Type	Width [min]	Area [mAU*s]	Height [mAU]	Area %
1	25.550	BB	1.2127	1.94362e4	242.58496	97.6165
2	34.906	MM	1.5723	474.58044	5.03063	2.3835

Totals : 1.99108e4 247.61560

Signal 3: DAD1 C, Sig=254,4 Ref=360,100

Signal 4: DAD1 D, Sig=280,4 Ref=360,100

Signal 5: DAD1 E, Sig=310,4 Ref=360,100

```
=====================================================================
                         *** End of Report ***
```

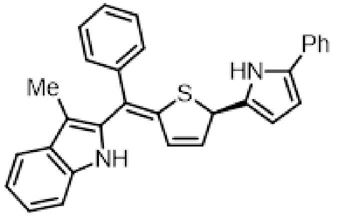

3a
(enantioenriched)

Supplementary Figure 143. HPLC spectrum of **3a** (enantioenriched)

```
Data File C:\CHEM32\1\DATA\QDY 2019-05-12 21-38-28\LXG-4114A-RAC.D
Sample Name:

    =====================================================================
    Acq. Operator   :                                Seq. Line :   2
    Acq. Instrument : Instrument 1                    Location : Vial 74
    Injection Date  : 5/12/2019 9:51:36 PM                 Inj :   1
                                                    Inj Volume : 5.000 µl
    Acq. Method     : C:\CHEM32\1\DATA\QDY 2019-05-12 21-38-28\OD-20-40.M
    Last changed    : 5/12/2019 9:50:42 PM
                      (modified after loading)
    Analysis Method : C:\CHEM32\1\METHODS\AD-07-60.M
    Last changed    : 5/14/2019 9:50:00 PM
                      (modified after loading)
    Additional Info : Peak(s) manually integrated
```

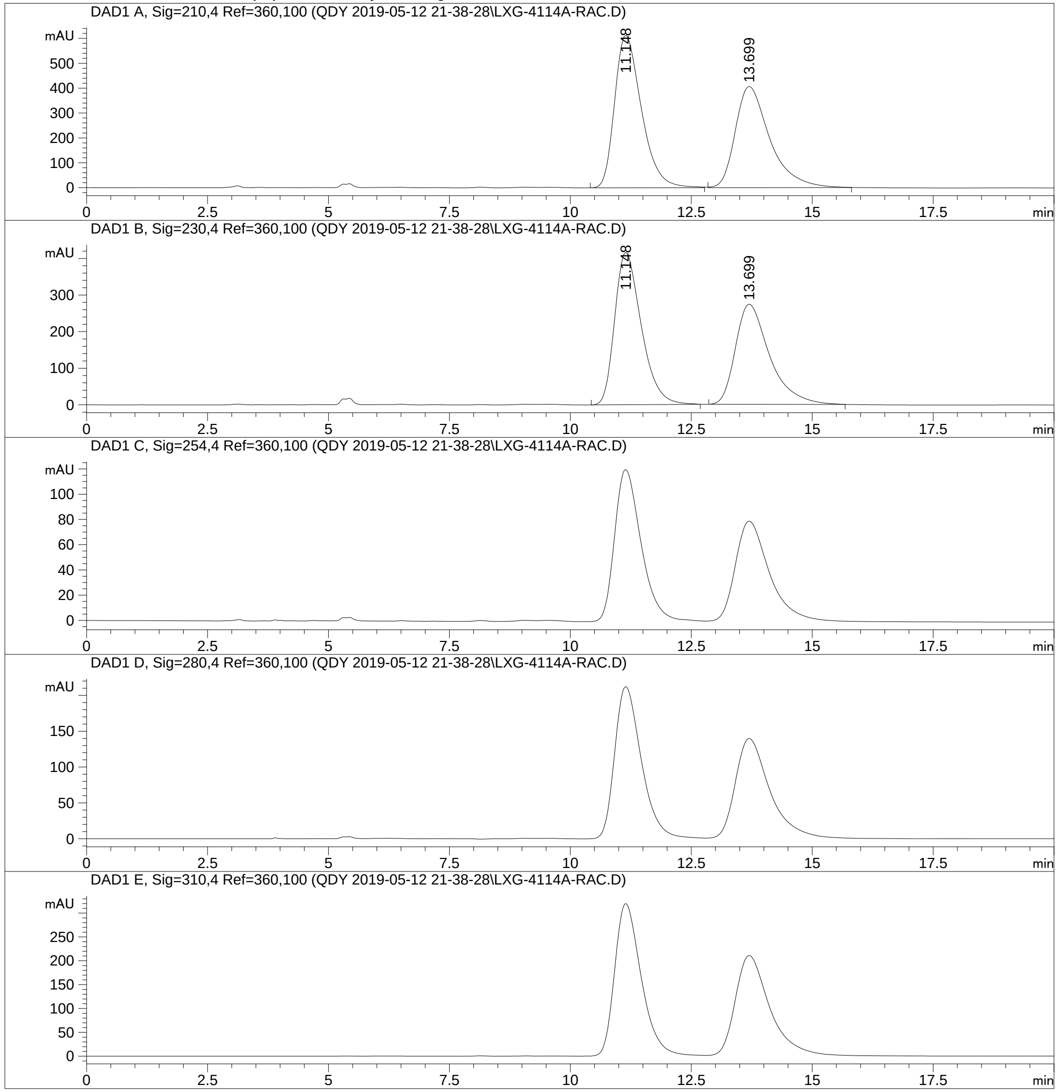


```
Data File C:\CHEM32\1\DATA\QDY 2019-05-12 21-38-28\LXG-4114A-RAC.D
Sample Name:

    =====================================================================
                             Area Percent Report
    =====================================================================

    Sorted By             :      Signal
    Multiplier            :      1.0000
    Dilution              :      1.0000
    Use Multiplier & Dilution Factor with ISTDs


    Signal 1: DAD1 A, Sig=210,4 Ref=360,100

    Peak RetTime Type  Width     Area      Height     Area
      #   [min]        [min]   [mAU*s]     [mAU]        %
    ----|-------|----|-------|----------|----------|--------|
       1  11.148 BB    0.5940 2.36213e4  612.36841  53.9615
       2  13.699 BB    0.7485 2.01531e4  406.44949  46.0385

    Totals :                  4.37744e4  1018.81790


    Signal 2: DAD1 B, Sig=230,4 Ref=360,100

    Peak RetTime Type  Width     Area      Height     Area
      #   [min]        [min]   [mAU*s]     [mAU]        %
    ----|-------|----|-------|----------|----------|--------|
       1  11.148 BB    0.5876 1.58890e4  415.99933  54.1782
       2  13.699 BB    0.7412 1.34383e4  273.52963  45.8218

    Totals :                  2.93273e4   689.52896


    Signal 3: DAD1 C, Sig=254,4 Ref=360,100


    Signal 4: DAD1 D, Sig=280,4 Ref=360,100


    Signal 5: DAD1 E, Sig=310,4 Ref=360,100

    =====================================================================
                              *** End of Report ***
```

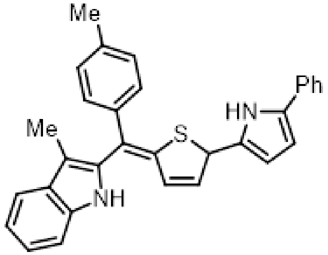

3b
(racemic)

Supplementary Figure 144. HPLC spectrum of **3b** (racemic)

Data File C:\CHEM32\1\DATA\QDY 2019-05-14 13-58-33\LXG-4116A-AGAIN.D
Sample Name:

===

Acq. Operator	:	Seq. Line :	11
Acq. Instrument	: Instrument 1	Location :	Vial 71
Injection Date	: 5/14/2019 5:03:43 PM	Inj :	1
		Inj Volume :	5.000 µl
Acq. Method	: C:\CHEM32\1\DATA\QDY 2019-05-14 13-58-33\OD-20-20.M		
Last changed	: 1/7/2016 10:22:18 AM		
Analysis Method	: C:\CHEM32\1\METHODS\AD-07-60.M		
Last changed	: 5/11/2019 10:45:40 AM (modified after loading)		
Additional Info	: Peak(s) manually integrated		

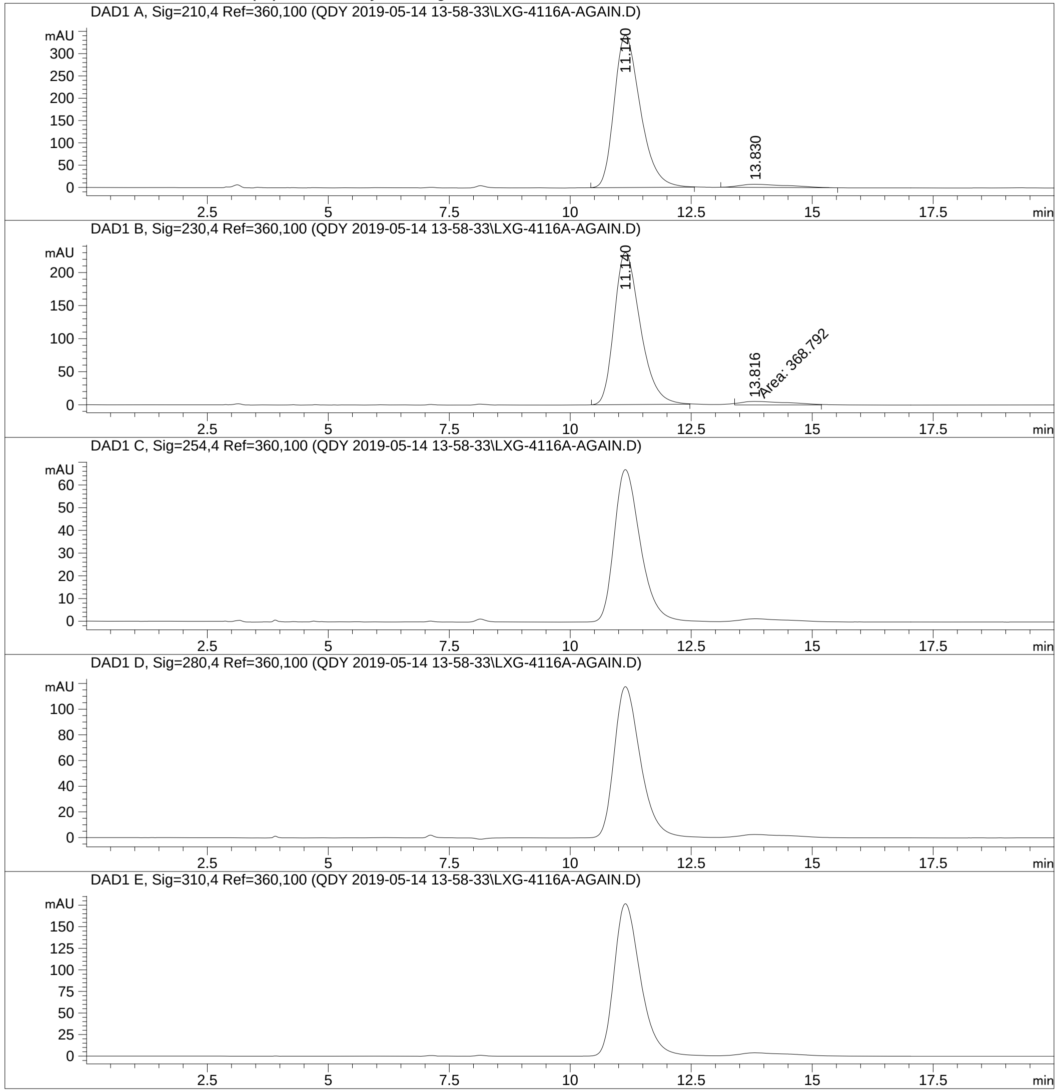

Data File C:\CHEM32\1\DATA\QDY 2019-05-14 13-58-33\LXG-4116A-AGAIN.D
Sample Name:

===

Area Percent Report

===

Sorted By : Signal
Multiplier : 1.0000
Dilution : 1.0000
Use Multiplier & Dilution Factor with ISTDs

Signal 1: DAD1 A, Sig=210,4 Ref=360,100

Peak #	RetTime [min]	Type	Width [min]	Area [mAU*s]	Height [mAU]	Area %
1	11.140	BB	0.5783	1.28095e4	340.94614	96.2167
2	13.830	BB	0.8642	503.67200	7.18166	3.7833

Totals : 1.33132e4 348.12780

Signal 2: DAD1 B, Sig=230,4 Ref=360,100

Peak #	RetTime [min]	Type	Width [min]	Area [mAU*s]	Height [mAU]	Area %
1	11.140	BB	0.5749	8615.99609	230.06705	95.8954
2	13.816	MM	1.1363	368.79227	5.40923	4.1046

Totals : 8984.78836 235.47627

Signal 3: DAD1 C, Sig=254,4 Ref=360,100

Signal 4: DAD1 D, Sig=280,4 Ref=360,100

Signal 5: DAD1 E, Sig=310,4 Ref=360,100

===

*** End of Report ***

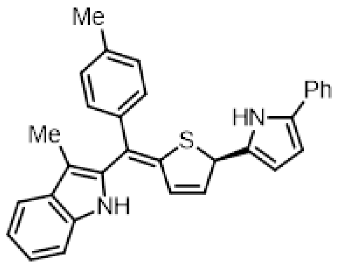

3b
(enantioenriched)

Supplementary Figure 145. HPLC spectrum of **3b** (enantioenriched)

```
Data File C:\CHEM32\1\DATA\QDY 2019-04-30 13-56-49\LXG-4092A-RAC4.D
Sample Name:

    =================================================================
    Acq. Operator   :                              Seq. Line :  20
    Acq. Instrument : Instrument 1                  Location : Vial 64
    Injection Date  : 4/30/2019 10:13:48 PM              Inj :   1
                                                  Inj Volume : 5.000 µl
    Acq. Method     : C:\CHEM32\1\DATA\QDY 2019-04-30 13-56-49\OD-20-40.M
    Last changed    : 4/30/2019 10:12:53 PM
                      (modified after loading)
    Analysis Method : C:\CHEM32\1\METHODS\AD-07-60.M
    Last changed    : 5/10/2019 8:11:55 PM
    Additional Info : Peak(s) manually integrated
```

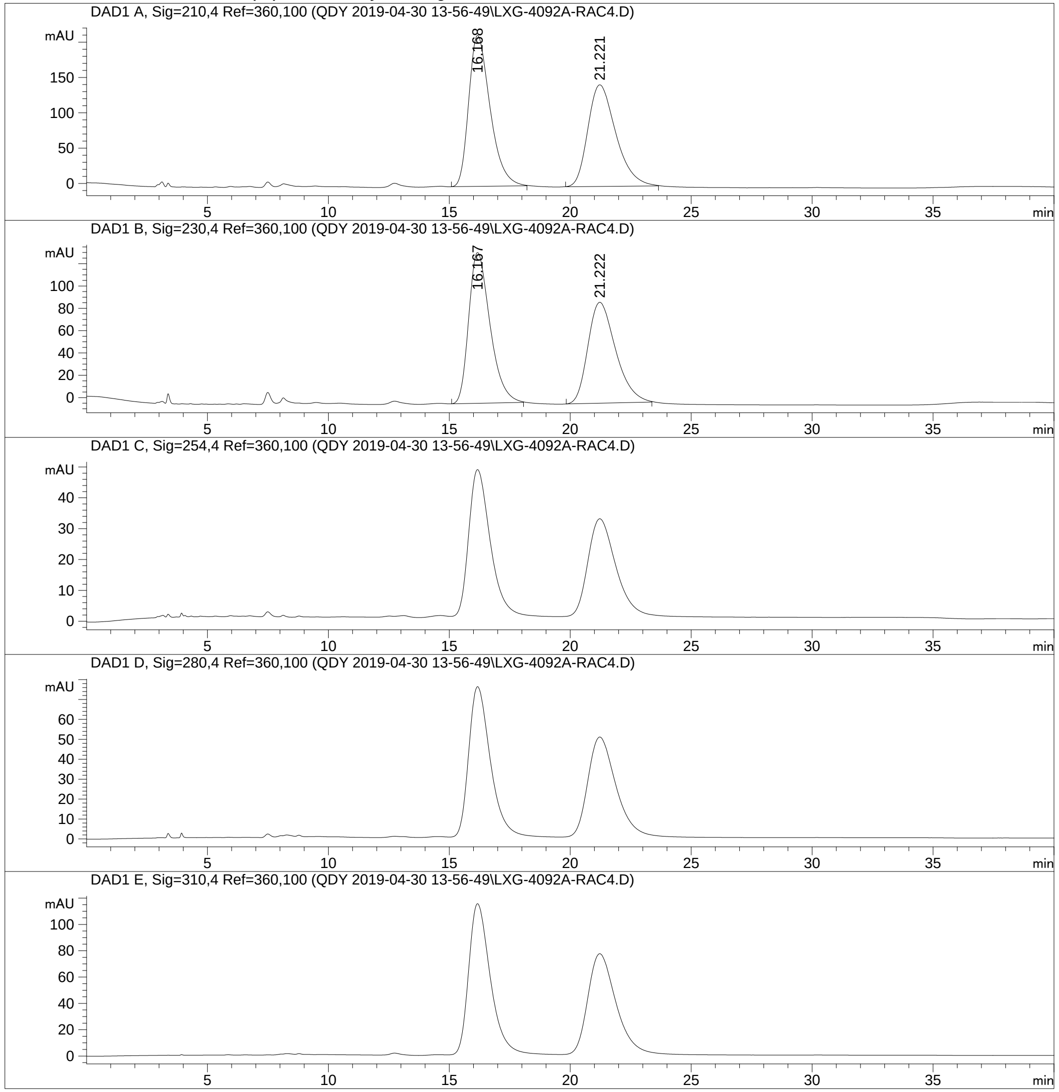


```
Data File C:\CHEM32\1\DATA\QDY 2019-04-30 13-56-49\LXG-4092A-RAC4.D
Sample Name:

    =====================================================================
                             Area Percent Report
    =====================================================================

    Sorted By             :      Signal
    Multiplier            :      1.0000
    Dilution              :      1.0000
    Use Multiplier & Dilution Factor with ISTDs


    Signal 1: DAD1 A, Sig=210,4 Ref=360,100

    Peak RetTime Type  Width     Area      Height     Area
      #   [min]        [min]   [mAU*s]     [mAU]        %
    ----|-------|----|-------|----------|----------|--------|
       1  16.168 BB    0.9450 1.31236e4  213.53802  53.1241
       2  21.221 BB    1.2213 1.15801e4  143.82903  46.8759

    Totals :                  2.47036e4   357.36705


    Signal 2: DAD1 B, Sig=230,4 Ref=360,100

    Peak RetTime Type  Width     Area      Height     Area
      #   [min]        [min]   [mAU*s]     [mAU]        %
    ----|-------|----|-------|----------|----------|--------|
       1  16.167 BB    0.9437 8262.72656  135.06790  53.4149
       2  21.222 BB    1.2216 7206.22119   90.43851  46.5851

    Totals :                  1.54689e4   225.50641


    Signal 3: DAD1 C, Sig=254,4 Ref=360,100


    Signal 4: DAD1 D, Sig=280,4 Ref=360,100


    Signal 5: DAD1 E, Sig=310,4 Ref=360,100

    =====================================================================
                              *** End of Report ***
```

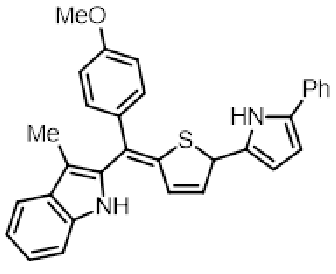

3c
(racemic)

Supplementary Figure 146. HPLC spectrum of **3c** (racemic)

```
=====================================================================
Acq. Operator   :                                Seq. Line :  25
Acq. Instrument : Instrument 1                    Location : Vial 79
Injection Date  : 5/11/2019 2:01:39 AM                 Inj :   1
                                                Inj Volume : 5.000 µl
Acq. Method     : C:\CHEM32\1\DATA\QDY 2019-05-10 15-31-07\OD-20-40.M
Last changed    : 5/11/2019 2:00:46 AM
                  (modified after loading)
Analysis Method : C:\CHEM32\1\METHODS\AD-07-60.M
Last changed    : 5/10/2019 8:11:55 PM
Additional Info : Peak(s) manually integrated
```

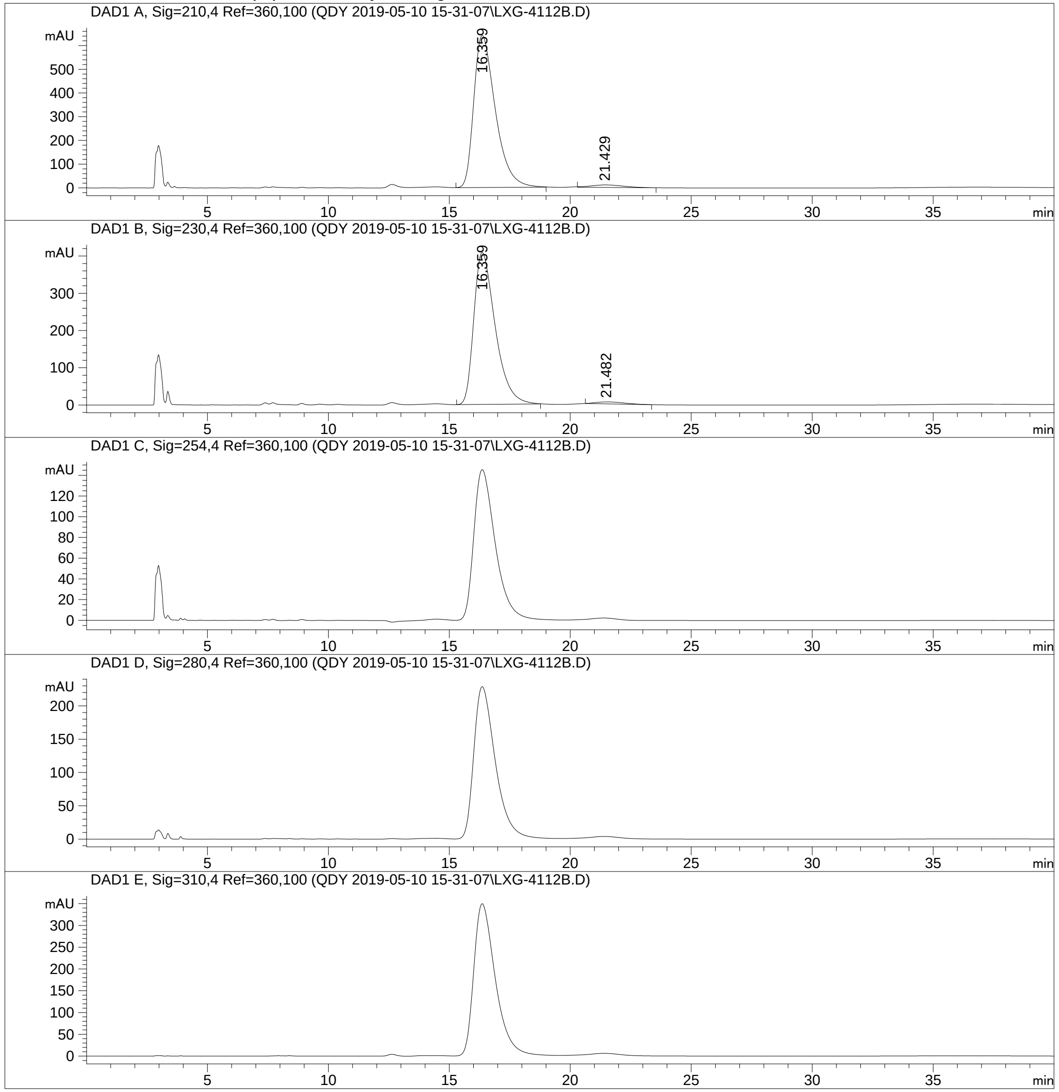

Data File C:\CHEM32\1\DATA\QDY 2019-05-10 15-31-07\LXG-4112B.D
Sample Name:

Area Percent Report

```
Sorted By          :      Signal
Multiplier         :      1.0000
Dilution           :      1.0000
Use Multiplier & Dilution Factor with ISTDs
```

Signal 1: DAD1 A, Sig=210,4 Ref=360,100

Peak #	RetTime [min]	Type	Width [min]	Area [mAU*s]	Height [mAU]	Area %
1	16.359	BB	0.9547	3.96769e4	640.54059	97.5310
2	21.429	BB	1.1596	1004.41107	10.73484	2.4690

Totals : 4.06813e4 651.27542

Signal 2: DAD1 B, Sig=230,4 Ref=360,100

Peak #	RetTime [min]	Type	Width [min]	Area [mAU*s]	Height [mAU]	Area %
1	16.359	BB	0.9498	2.50930e4	407.84644	98.2266
2	21.482	BB	1.0759	453.02261	5.37173	1.7734

Totals : 2.55460e4 413.21817

Signal 3: DAD1 C, Sig=254,4 Ref=360,100

Signal 4: DAD1 D, Sig=280,4 Ref=360,100

Signal 5: DAD1 E, Sig=310,4 Ref=360,100

*** End of Report ***

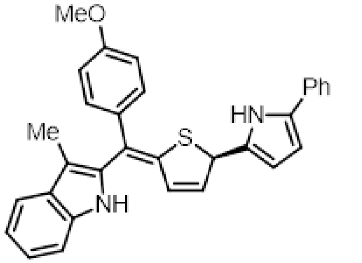

3c
(enantioenriched)

Supplementary Figure 147. HPLC spectrum of **3c** (enantioenriched)

```
=====================================================================
Acq. Operator   :                                Seq. Line :  11
Acq. Instrument : Instrument 1                    Location : Vial 62
Injection Date  : 4/30/2019 3:22:49 AM                 Inj :   1
                                                Inj Volume : 5.000 µl
Acq. Method     : C:\CHEM32\1\DATA\QDY 2019-04-29 22-16-51\OD-20-60.M
Last changed    : 4/30/2019 2:20:20 AM
                  (modified after loading)
Analysis Method : C:\CHEM32\1\METHODS\AD-07-60.M
Last changed    : 5/11/2019 10:41:57 AM
                  (modified after loading)
Additional Info : Peak(s) manually integrated
```

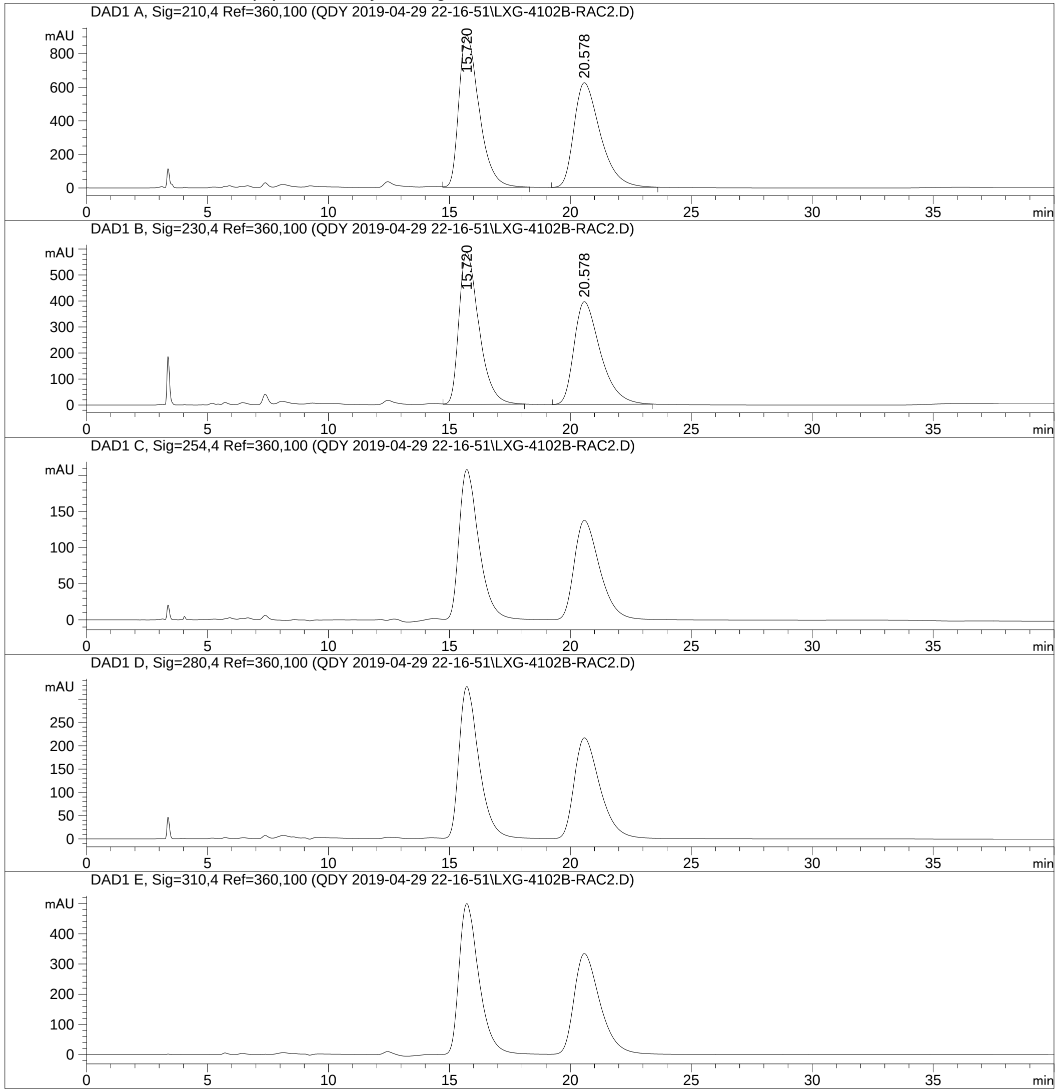

Data File C:\CHEM32\1\DATA\QDY 2019-04-29 22-16-51\LXG-4102B-RAC2.D
Sample Name:

```
=====================================================================
                        Area Percent Report
=====================================================================

Sorted By             :      Signal
Multiplier            :      1.0000
Dilution              :      1.0000
Use Multiplier & Dilution Factor with ISTDs


Signal 1: DAD1 A, Sig=210,4 Ref=360,100

Peak RetTime Type  Width     Area      Height     Area
  #   [min]        [min]   [mAU*s]     [mAU]        %
----|-------|----|-------|----------|----------|--------|
   1  15.720 VB    0.8926 5.27028e4   906.06335  52.6512
   2  20.578 BB    1.1581 4.73952e4   623.99194  47.3488

Totals :                  1.00098e5  1530.05530


Signal 2: DAD1 B, Sig=230,4 Ref=360,100

Peak RetTime Type  Width     Area      Height     Area
  #   [min]        [min]   [mAU*s]     [mAU]        %
----|-------|----|-------|----------|----------|--------|
   1  15.720 VB    0.8745 3.34375e4   583.57526  52.7437
   2  20.578 BB    1.1531 2.99586e4   394.87662  47.2563

Totals :                  6.33961e4   978.45187


Signal 3: DAD1 C, Sig=254,4 Ref=360,100


Signal 4: DAD1 D, Sig=280,4 Ref=360,100


Signal 5: DAD1 E, Sig=310,4 Ref=360,100

=====================================================================
                          *** End of Report ***
```

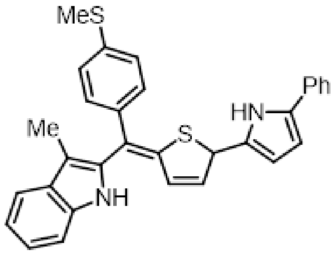

3d
(racemic)

Supplementary Figure 148. HPLC spectrum of **3d** (racemic)

===

Acq. Operator : Seq. Line : 26
Acq. Instrument : Instrument 1 Location : Vial 80
Injection Date : 5/11/2019 2:42:47 AM Inj : 1
Inj Volume : 5.000 µl
Acq. Method : C:\CHEM32\1\DATA\QDY 2019-05-10 15-31-07\OD-20-40.M
Last changed : 5/11/2019 2:00:46 AM
(modified after loading)
Analysis Method : C:\CHEM32\1\METHODS\AD-07-60.M
Last changed : 5/10/2019 8:11:55 PM
Additional Info : Peak(s) manually integrated

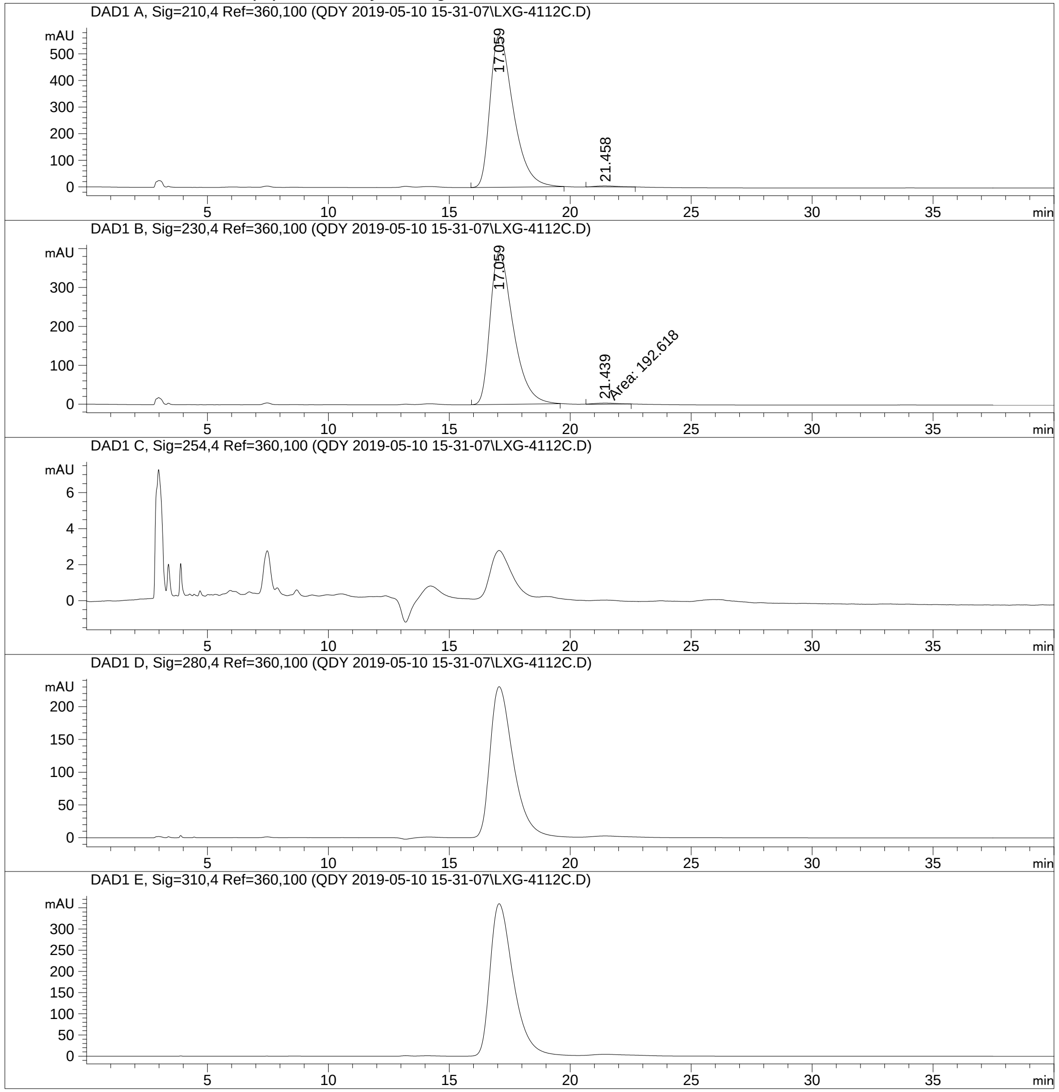

Data File C:\CHEM32\1\DATA\QDY 2019-05-10 15-31-07\LXG-4112C.D
Sample Name:

===

Area Percent Report

===

Sorted By : Signal
Multiplier : 1.0000
Dilution : 1.0000
Use Multiplier & Dilution Factor with ISTDs

Signal 1: DAD1 A, Sig=210,4 Ref=360,100

Peak #	RetTime [min]	Type	Width [min]	Area [mAU*s]	Height [mAU]	Area %
1	17.059	BB	1.0195	3.80362e4	570.76141	99.3399
2	21.458	BB	0.7876	252.76334	3.81169	0.6601

Totals : 3.82890e4 574.57310

Signal 2: DAD1 B, Sig=230,4 Ref=360,100

Peak #	RetTime [min]	Type	Width [min]	Area [mAU*s]	Height [mAU]	Area %
1	17.059	BB	1.0122	2.58474e4	390.51089	99.2603
2	21.439	MM	1.1052	192.61755	2.90478	0.7397

Totals : 2.60401e4 393.41568

Signal 3: DAD1 C, Sig=254,4 Ref=360,100

Signal 4: DAD1 D, Sig=280,4 Ref=360,100

Signal 5: DAD1 E, Sig=310,4 Ref=360,100

===

*** End of Report ***

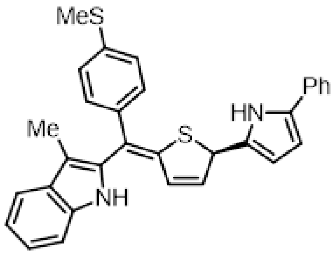

3d
(enantioenriched)

Supplementary Figure 149. HPLC spectrum of **3d** (enantioenriched)

Data File C:\CHEM32\1\DATA\QDY 2020-06-28 16-23-58\LXG-7074A-RAC.D
Sample Name:

===

Acq. Operator : Seq. Line : 5
Acq. Instrument : Instrument 1 Location : Vial 64
Injection Date : 6/28/2020 6:09:56 PM Inj : 1
Inj Volume : 5.000 µl
Different Inj Volume from Sequence ! Actual Inj Volume : 4.000 µl
Acq. Method : C:\CHEM32\1\DATA\QDY 2020-06-28 16-23-58\AD-20-30.M
Last changed : 6/15/2018 10:29:43 AM
Analysis Method : C:\CHEM32\1\METHODS\LXG-IC-10-10.M
Last changed : 7/4/2020 3:07:23 PM
(modified after loading)
Additional Info : Peak(s) manually integrated

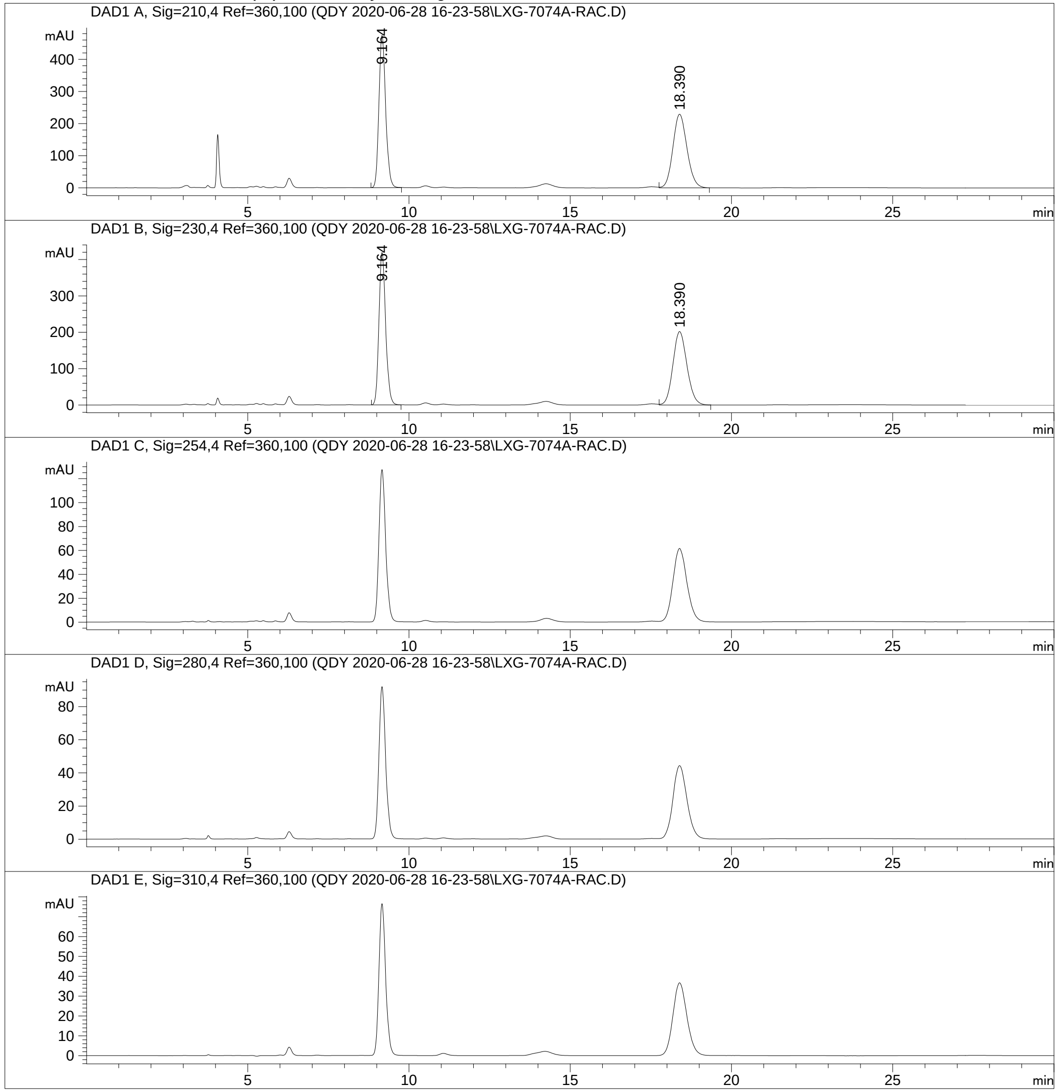

Data File C:\CHEM32\1\DATA\QDY 2020-06-28 16-23-58\LXG-7074A-RAC.D
Sample Name:

===
Area Percent Report
===

Sorted By : Signal
Multiplier : 1.0000
Dilution : 1.0000
Use Multiplier & Dilution Factor with ISTDs

Signal 1: DAD1 A, Sig=210,4 Ref=360,100

Peak #	RetTime [min]	Type	Width [min]	Area [mAU*s]	Height [mAU]	Area %
1	9.164	BB	0.2239	6896.58789	473.69022	49.8099
2	18.390	VB	0.4676	6949.21729	229.29904	50.1901

Totals : 1.38458e4 702.98926

Signal 2: DAD1 B, Sig=230,4 Ref=360,100

Peak #	RetTime [min]	Type	Width [min]	Area [mAU*s]	Height [mAU]	Area %
1	9.164	BB	0.2233	6077.68115	418.79807	49.8301
2	18.390	VB	0.4696	6119.12939	201.88904	50.1699

Totals : 1.21968e4 620.68710

Signal 3: DAD1 C, Sig=254,4 Ref=360,100

Signal 4: DAD1 D, Sig=280,4 Ref=360,100

Signal 5: DAD1 E, Sig=310,4 Ref=360,100

===
*** End of Report ***

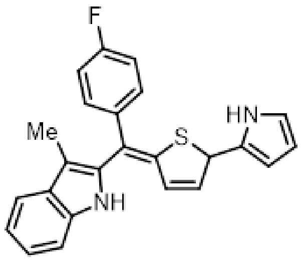

3e
(racemic)

Supplementary Figure 150. HPLC spectrum of **3e** (racemic)

```
=====================================================================
Acq. Operator   :                               Seq. Line :   2
Acq. Instrument : Instrument 1                   Location : Vial 65
Injection Date  : 7/1/2020 2:26:44 PM                 Inj :   1
                                               Inj Volume : 5.000 µl
Different Inj Volume from Sequence !     Actual Inj Volume : 4.000 µl
Acq. Method     : C:\CHEM32\1\DATA\QDY 2020-07-01 14-13-48\AD-20-30.M
Last changed    : 6/15/2018 10:29:43 AM
Analysis Method : C:\CHEM32\1\METHODS\LXG-IC-10-10.M
Last changed    : 7/4/2020 12:06:36 PM
                  (modified after loading)
Additional Info : Peak(s) manually integrated
```

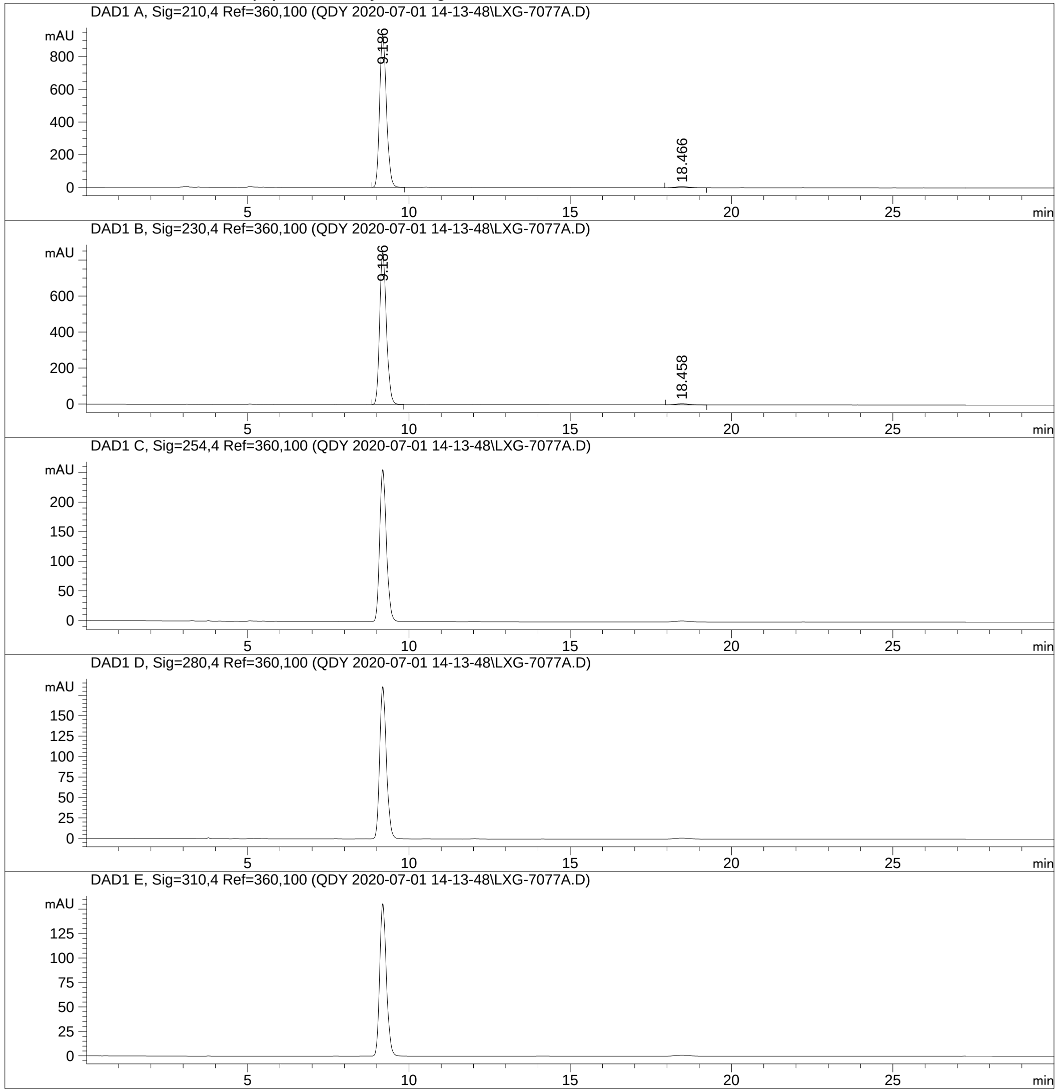

Data File C:\CHEM32\1\DATA\QDY 2020-07-01 14-13-48\LXG-7077A.D
Sample Name:

```
=====================================================================
                         Area Percent Report
=====================================================================

Sorted By             :      Signal
Multiplier            :      1.0000
Dilution              :      1.0000
Use Multiplier & Dilution Factor with ISTDs


Signal 1: DAD1 A, Sig=210,4 Ref=360,100

Peak RetTime Type  Width     Area      Height     Area
  #   [min]        [min]   [mAU*s]     [mAU]        %
----|-------|----|-------|----------|----------|--------|
   1   9.186 BB    0.2279 1.37054e4   930.15314  98.6113
   2  18.466 BB    0.4517  193.00525    6.55141   1.3887

Totals :                  1.38984e4   936.70454


Signal 2: DAD1 B, Sig=230,4 Ref=360,100

Peak RetTime Type  Width     Area      Height     Area
  #   [min]        [min]   [mAU*s]     [mAU]        %
----|-------|----|-------|----------|----------|--------|
   1   9.186 BB    0.2234 1.22628e4   844.31873  98.6389
   2  18.458 BB    0.4592  169.20894    5.75184   1.3611

Totals :                  1.24320e4   850.07057


Signal 3: DAD1 C, Sig=254,4 Ref=360,100


Signal 4: DAD1 D, Sig=280,4 Ref=360,100


Signal 5: DAD1 E, Sig=310,4 Ref=360,100

=====================================================================
                          *** End of Report ***
```

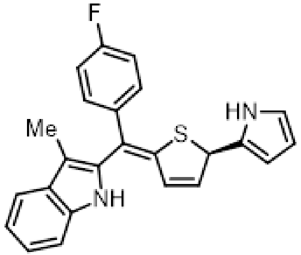

3e
(enantioenriched)

Supplementary Figure 151. HPLC spectrum of **3e** (enantioenriched)

Data File C:\CHEM32\1\DATA\QDY 2020-07-01 20-49-19\LXG-7074F-RAC.D
Sample Name:

```
=====================================================================
Acq. Operator   :                                 Seq. Line :   9
Acq. Instrument : Instrument 1                     Location : Vial 68
Injection Date  : 7/1/2020 11:50:21 PM                  Inj :   1
                                                 Inj Volume : 5.000 µl
Different Inj Volume from Sequence !     Actual Inj Volume : 4.000 µl
Acq. Method     : C:\CHEM32\1\DATA\QDY 2020-07-01 20-49-19\AD-20-40.M
Last changed    : 7/1/2020 11:49:28 PM
                  (modified after loading)
Analysis Method : C:\CHEM32\1\METHODS\LXG-IC-10-10.M
Last changed    : 7/4/2020 3:25:56 PM
                  (modified after loading)
Additional Info : Peak(s) manually integrated
```

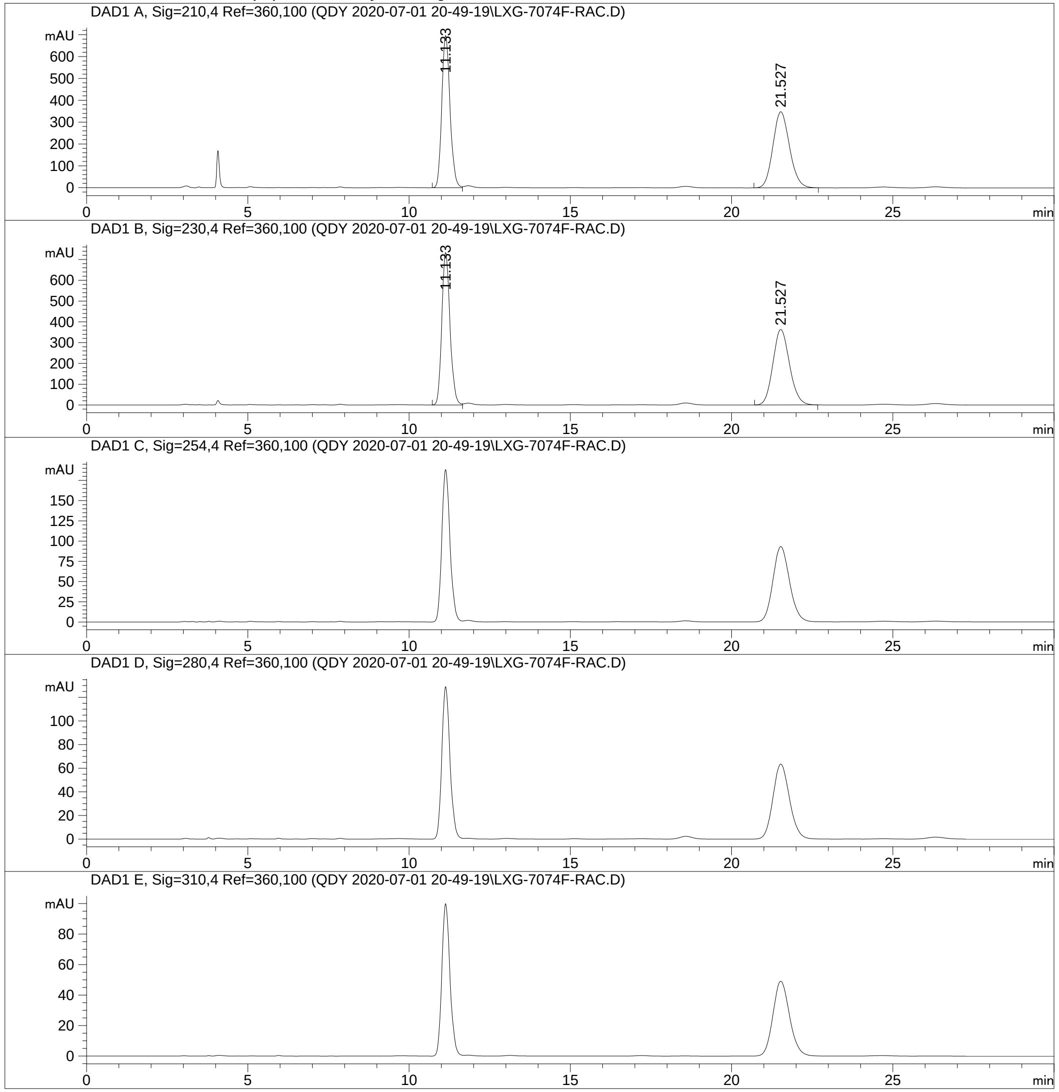

Data File C:\CHEM32\1\DATA\QDY 2020-07-01 20-49-19\LXG-7074F-RAC.D
Sample Name:

```
=====================================================================
                         Area Percent Report
=====================================================================

Sorted By             :      Signal
Multiplier            :      1.0000
Dilution              :      1.0000
Use Multiplier & Dilution Factor with ISTDs


Signal 1: DAD1 A, Sig=210,4 Ref=360,100

Peak RetTime Type  Width     Area      Height     Area
  #   [min]        [min]   [mAU*s]     [mAU]        %
----|-------|----|-------|----------|----------|--------|
   1  11.133 BV    0.2727 1.23559e4   697.36481  49.7126
   2  21.527 BB    0.5565 1.24988e4   348.41791  50.2874

Totals :                  2.48547e4  1045.78271


Signal 2: DAD1 B, Sig=230,4 Ref=360,100

Peak RetTime Type  Width     Area      Height     Area
  #   [min]        [min]   [mAU*s]     [mAU]        %
----|-------|----|-------|----------|----------|--------|
   1  11.133 BV    0.2717 1.29368e4   733.56958  49.8737
   2  21.527 BB    0.5534 1.30024e4   363.40698  50.1263

Totals :                  2.59392e4  1096.97656


Signal 3: DAD1 C, Sig=254,4 Ref=360,100


Signal 4: DAD1 D, Sig=280,4 Ref=360,100


Signal 5: DAD1 E, Sig=310,4 Ref=360,100

=====================================================================
                          *** End of Report ***
```

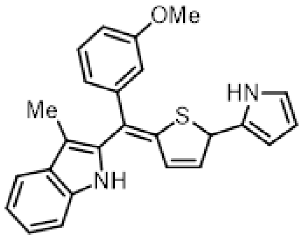

3f
(racemic)

Supplementary Figure 152. HPLC spectrum of **3f** (racemic)

Data File C:\CHEM32\1\DATA\QDY 2020-07-03 11-16-20\LXG-7079A-AGAIN.D
Sample Name:

===

```
Acq. Operator   :                               Seq. Line :  23
Acq. Instrument : Instrument 1                   Location : Vial 64
Injection Date  : 7/3/2020 7:12:32 PM                 Inj :   1
                                               Inj Volume : 5.000 µl
Different Inj Volume from Sequence !     Actual Inj Volume : 4.000 µl
Acq. Method     : C:\CHEM32\1\DATA\QDY 2020-07-03 11-16-20\AD-20-30.M
Last changed    : 6/15/2018 10:29:43 AM
Analysis Method : C:\CHEM32\1\METHODS\LXG-IC-10-10.M
Last changed    : 7/4/2020 3:16:15 PM
                  (modified after loading)
Additional Info : Peak(s) manually integrated
```

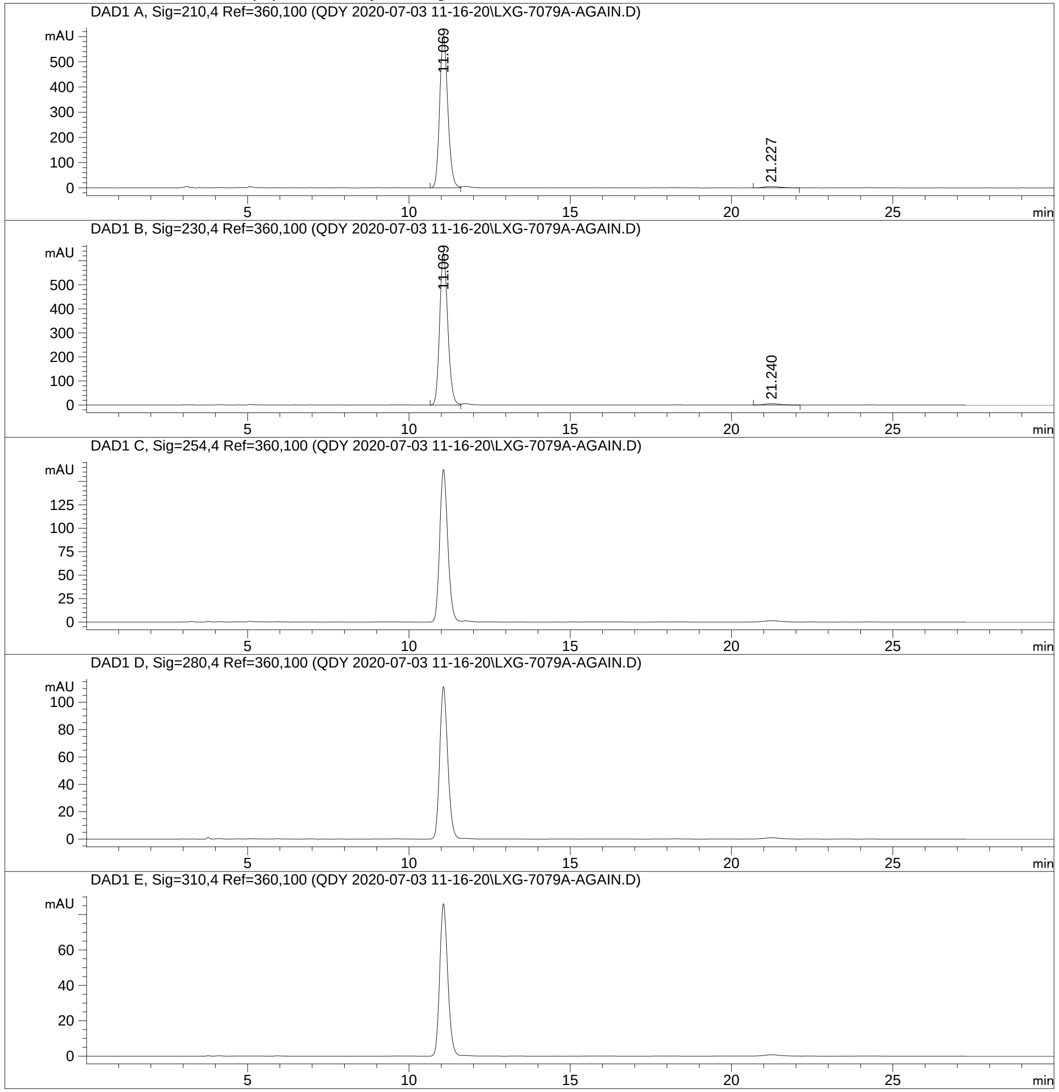

Data File C:\CHEM32\1\DATA\QDY 2020-07-03 11-16-20\LXG-7079A-AGAIN.D
Sample Name:

===
Area Percent Report
===

```
Sorted By             :      Signal
Multiplier            :      1.0000
Dilution              :      1.0000
Use Multiplier & Dilution Factor with ISTDs
```

Signal 1: DAD1 A, Sig=210,4 Ref=360,100

Peak #	RetTime [min]	Type	Width [min]	Area [mAU*s]	Height [mAU]	Area %
1	11.069	BV	0.2696	1.05527e4	604.80225	98.2917
2	21.227	BB	0.4792	183.40189	5.17820	1.7083

Totals : 1.07361e4 609.98045

Signal 2: DAD1 B, Sig=230,4 Ref=360,100

Peak #	RetTime [min]	Type	Width [min]	Area [mAU*s]	Height [mAU]	Area %
1	11.069	BV	0.2668	1.10218e4	634.18201	98.3362
2	21.240	BB	0.5339	186.48531	5.41394	1.6638

Totals : 1.12082e4 639.59595

Signal 3: DAD1 C, Sig=254,4 Ref=360,100

Signal 4: DAD1 D, Sig=280,4 Ref=360,100

Signal 5: DAD1 E, Sig=310,4 Ref=360,100

===
*** End of Report ***

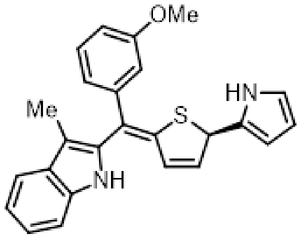

3f
(enantioenriched)

Supplementary Figure 153. HPLC spectrum of **3f** (enantioenriched)

Data File C:\CHEM32\1\DATA\QDY 2019-05-08 10-13-40\LXG-4104B-RAC3.D
Sample Name:

===

Acq. Operator	:	Seq. Line :	7
Acq. Instrument	: Instrument 1	Location :	Vial 72
Injection Date	: 5/8/2019 1:23:46 PM	Inj :	1
		Inj Volume :	5.000 µl

Different Inj Volume from Sequence ! Actual Inj Volume : 2.000 µl
Acq. Method : C:\CHEM32\1\DATA\QDY 2019-05-08 10-13-40\OD-20-60.M
Last changed : 5/8/2019 1:22:54 PM
(modified after loading)
Analysis Method : C:\CHEM32\1\METHODS\AD-07-60.M
Last changed : 5/14/2019 9:51:54 PM
(modified after loading)
Additional Info : Peak(s) manually integrated

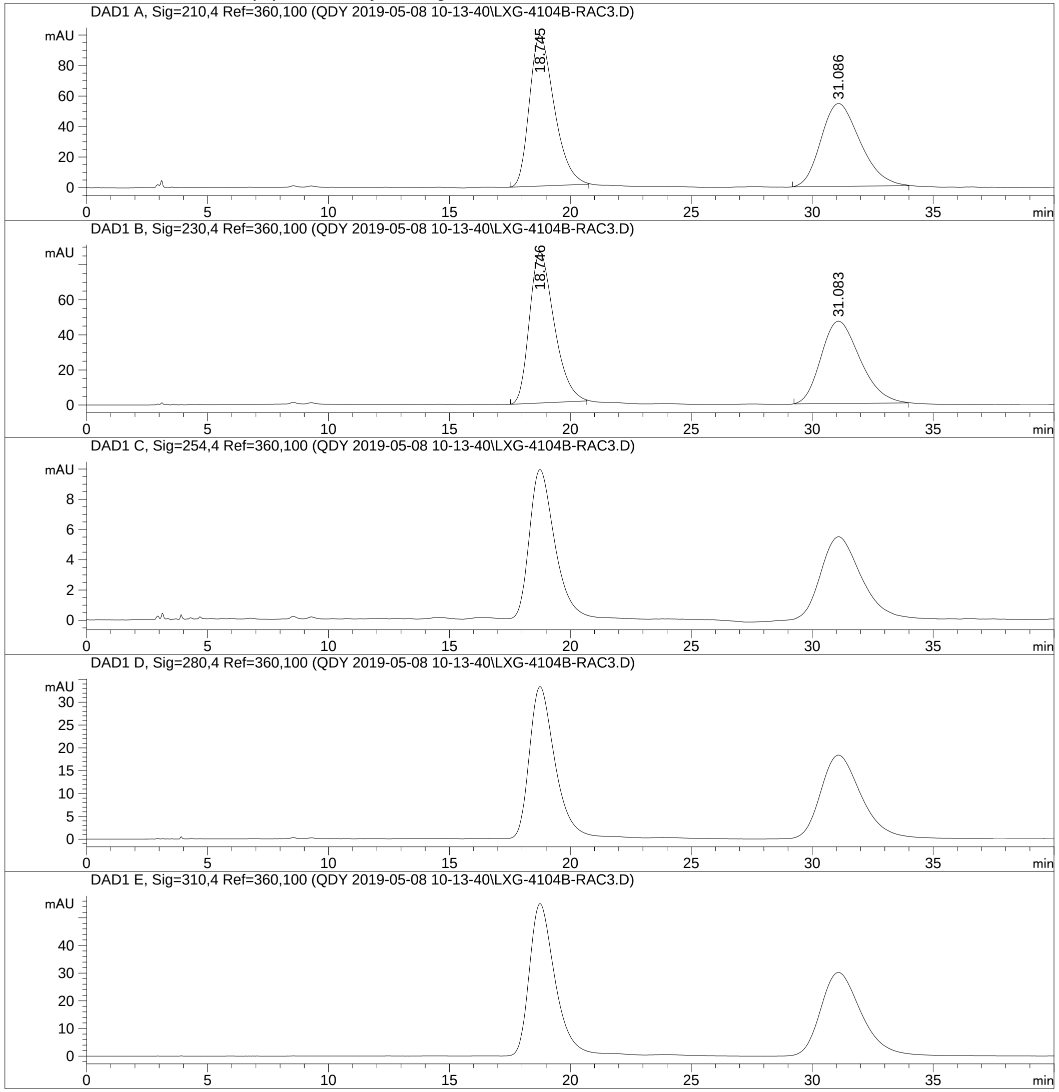

Data File C:\CHEM32\1\DATA\QDY 2019-05-08 10-13-40\LXG-4104B-RAC3.D
Sample Name:

===

Area Percent Report

===

Sorted By : Signal
Multiplier : 1.0000
Dilution : 1.0000
Use Multiplier & Dilution Factor with ISTDs

Signal 1: DAD1 A, Sig=210,4 Ref=360,100

Peak #	RetTime [min]	Type	Width [min]	Area [mAU*s]	Height [mAU]	Area %
1	18.745	BB	1.0800	7038.53906	98.69179	53.4883
2	31.086	BB	1.5403	6120.47754	54.32919	46.5117

Totals : 1.31590e4 153.02098

Signal 2: DAD1 B, Sig=230,4 Ref=360,100

Peak #	RetTime [min]	Type	Width [min]	Area [mAU*s]	Height [mAU]	Area %
1	18.746	BB	1.0908	6096.00684	85.81546	53.6420
2	31.083	BB	1.6792	5268.23389	46.94790	46.3580

Totals : 1.13642e4 132.76336

Signal 3: DAD1 C, Sig=254,4 Ref=360,100

Signal 4: DAD1 D, Sig=280,4 Ref=360,100

Signal 5: DAD1 E, Sig=310,4 Ref=360,100

===

*** End of Report ***

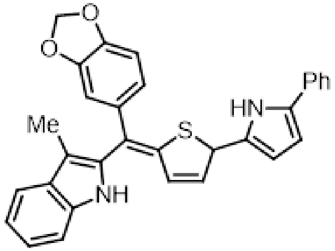

3g
(racemic)

Supplementary Figure 154. HPLC spectrum of **3g** (racemic)

Data File C:\CHEM32\1\DATA\QDY 2019-05-14 13-58-33\LXG-4116B.D
Sample Name:

```
=====================================================================
Acq. Operator   :                              Seq. Line :  12
Acq. Instrument : Instrument 1                  Location : Vial 72
Injection Date  : 5/14/2019 5:24:48 PM               Inj :   1
                                              Inj Volume : 5.000 µl
Acq. Method     : C:\CHEM32\1\DATA\QDY 2019-05-14 13-58-33\OD-20-40.M
Last changed    : 5/14/2019 5:23:58 PM
                  (modified after loading)
Analysis Method : C:\CHEM32\1\METHODS\AD-07-60.M
Last changed    : 5/11/2019 10:45:40 AM
                  (modified after loading)
Additional Info : Peak(s) manually integrated
```

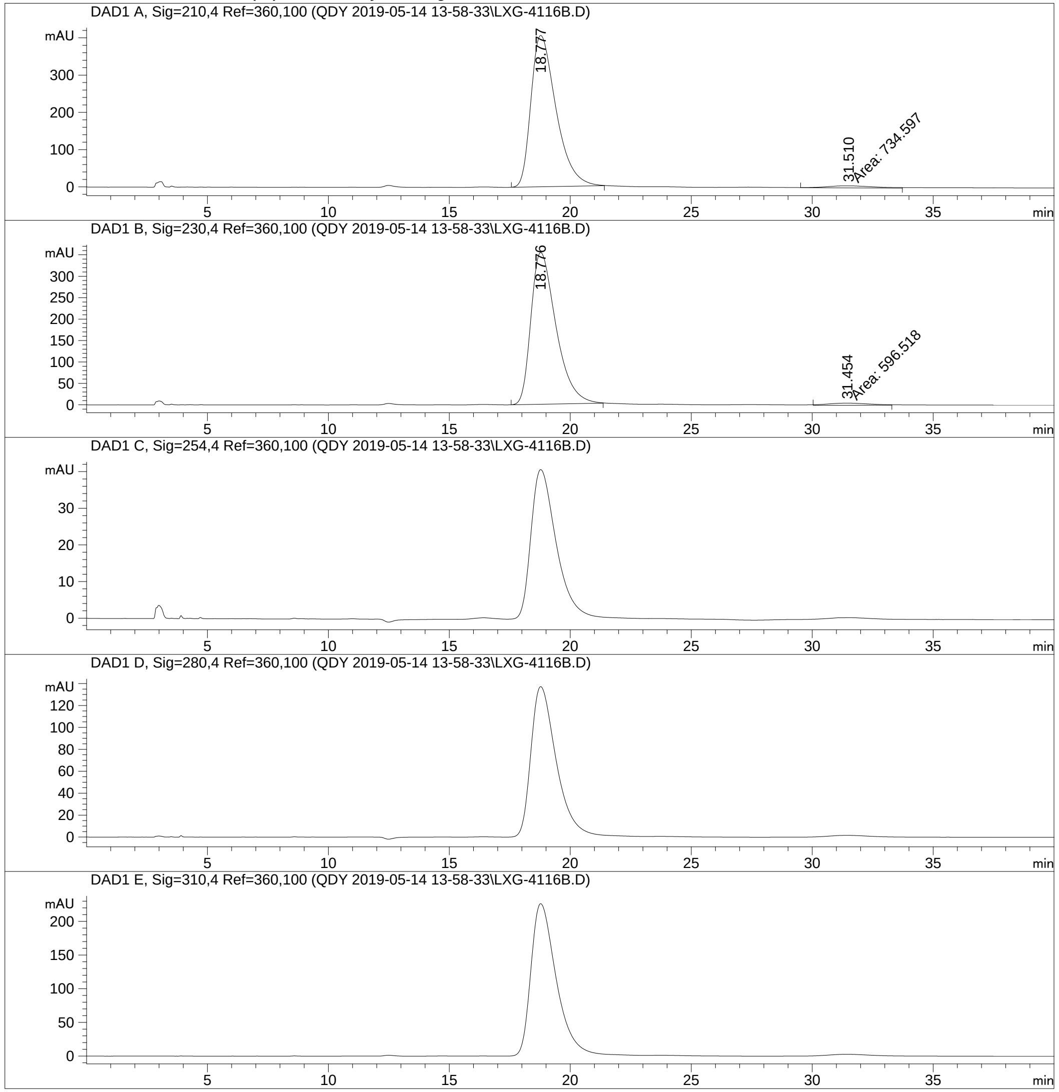

Data File C:\CHEM32\1\DATA\QDY 2019-05-14 13-58-33\LXG-4116B.D
Sample Name:

```
=====================================================================
                         Area Percent Report
=====================================================================

Sorted By             :      Signal
Multiplier            :      1.0000
Dilution              :      1.0000
Use Multiplier & Dilution Factor with ISTDs
```

Signal 1: DAD1 A, Sig=210,4 Ref=360,100

Peak #	RetTime [min]	Type	Width [min]	Area [mAU*s]	Height [mAU]	Area %
1	18.777	BB	1.0965	2.92674e4	406.26563	97.5515
2	31.510	MM	2.2217	734.59674	5.51070	2.4485

Totals : 3.00020e4 411.77632

Signal 2: DAD1 B, Sig=230,4 Ref=360,100

Peak #	RetTime [min]	Type	Width [min]	Area [mAU*s]	Height [mAU]	Area %
1	18.776	BB	1.0948	2.54552e4	354.03140	97.7103
2	31.454	MM	2.0271	596.51813	4.90447	2.2897

Totals : 2.60517e4 358.93587

Signal 3: DAD1 C, Sig=254,4 Ref=360,100

Signal 4: DAD1 D, Sig=280,4 Ref=360,100

Signal 5: DAD1 E, Sig=310,4 Ref=360,100

===
*** End of Report ***

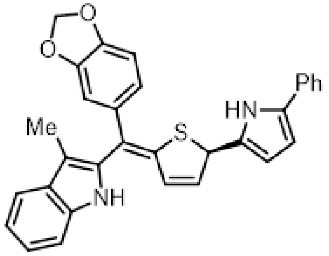

3g
(enantioenriched)

Supplementary Figure 155. HPLC spectrum of **3g** (enantioenriched)

```
=====================================================================
Acq. Operator   :                              Seq. Line :   2
Acq. Instrument : Instrument 1                  Location : Vial 78
Injection Date  : 5/30/2019 11:15:01 AM              Inj :   1
                                              Inj Volume : 5.000 µl
Acq. Method     : C:\CHEM32\1\DATA\QDY 2019-05-30 11-01-23\OD-20-40.M
Last changed    : 5/30/2019 11:14:08 AM
                  (modified after loading)
Analysis Method : C:\CHEM32\1\METHODS\OD-01-45-0.5.M
Last changed    : 8/20/2020 7:42:57 PM
                  (modified after loading)
Additional Info : Peak(s) manually integrated
```

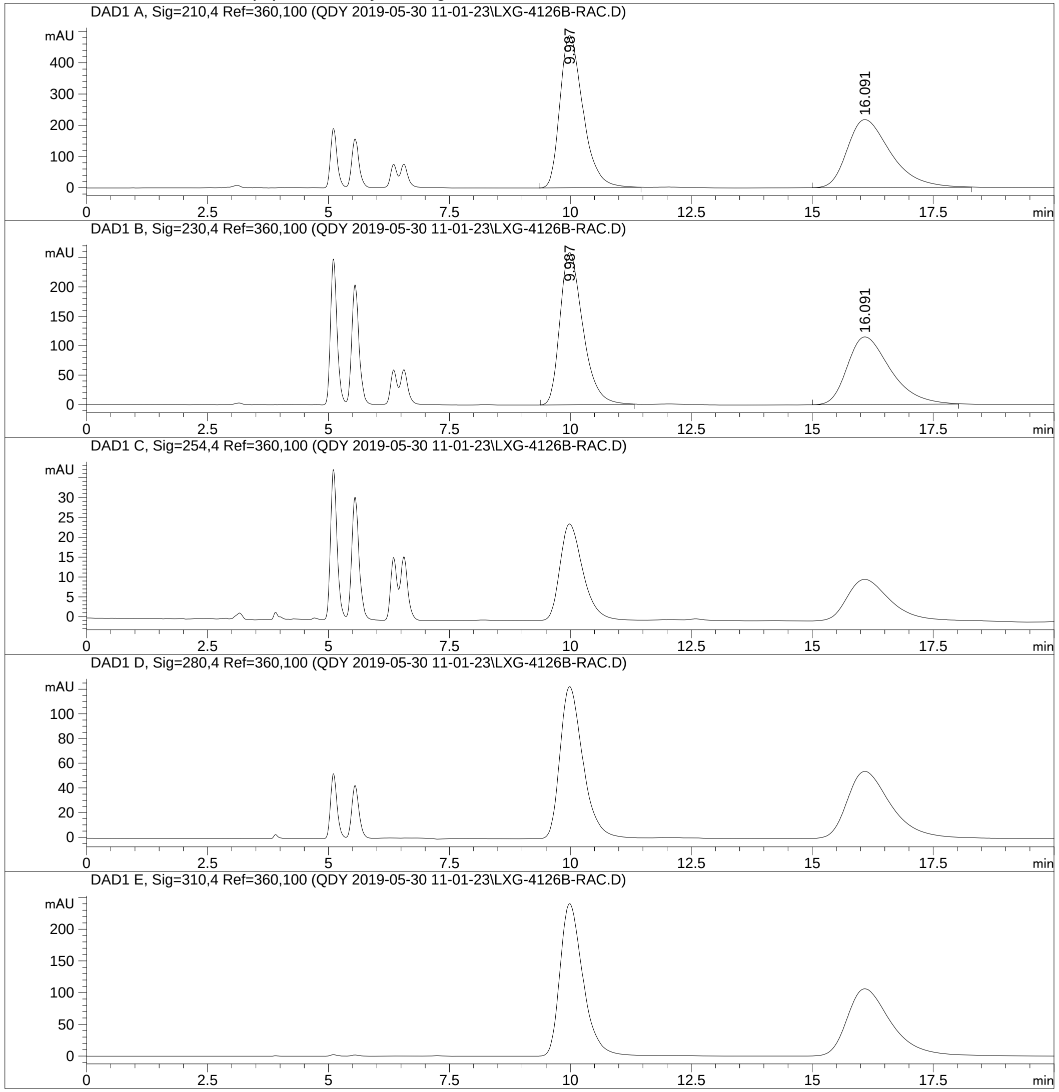

Data File C:\CHEM32\1\DATA\QDY 2019-05-30 11-01-23\LXG-4126B-RAC.D
Sample Name:

```
=====================================================================
                        Area Percent Report
=====================================================================

Sorted By           :      Signal
Multiplier          :      1.0000
Dilution            :      1.0000
Use Multiplier & Dilution Factor with ISTDs


Signal 1: DAD1 A, Sig=210,4 Ref=360,100

Peak RetTime Type  Width     Area      Height     Area
  #   [min]        [min]   [mAU*s]     [mAU]        %
----|-------|----|-------|----------|----------|--------|
   1   9.987 BB    0.5077 1.61082e4  487.29367  54.1869
   2  16.091 BB    0.9504 1.36189e4  217.53532  45.8131

Totals :                  2.97271e4  704.82899


Signal 2: DAD1 B, Sig=230,4 Ref=360,100

Peak RetTime Type  Width     Area      Height     Area
  #   [min]        [min]   [mAU*s]     [mAU]        %
----|-------|----|-------|----------|----------|--------|
   1   9.987 BB    0.5033 8513.64551 259.14014  54.3847
   2  16.091 BB    0.9463 7140.83887 115.02266  45.6153

Totals :                  1.56545e4  374.16280


Signal 3: DAD1 C, Sig=254,4 Ref=360,100


Signal 4: DAD1 D, Sig=280,4 Ref=360,100


Signal 5: DAD1 E, Sig=310,4 Ref=360,100

=====================================================================
                      *** End of Report ***
```

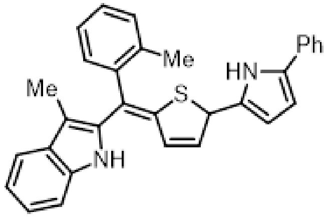

3h
(racemic)

Supplementary Figure 156. HPLC spectrum of **3h** (racemic)

Data File C:\CHEM32\1\DATA\QDY 2019-06-14 21-42-49\LXG-4142C.D
Sample Name:

===

Acq. Operator	:	Seq. Line :	4
Acq. Instrument	: Instrument 1	Location :	Vial 67
Injection Date	: 6/14/2019 10:58:30 PM	Inj :	1
		Inj Volume :	5.000 µl

Acq. Method : C:\CHEM32\1\DATA\QDY 2019-06-14 21-42-49\OD-20-20.M
Last changed : 6/14/2019 10:57:33 PM
(modified after loading)
Analysis Method : C:\CHEM32\1\METHODS\OD-01-45-0.5.M
Last changed : 8/20/2020 6:53:30 PM
(modified after loading)
Additional Info : Peak(s) manually integrated

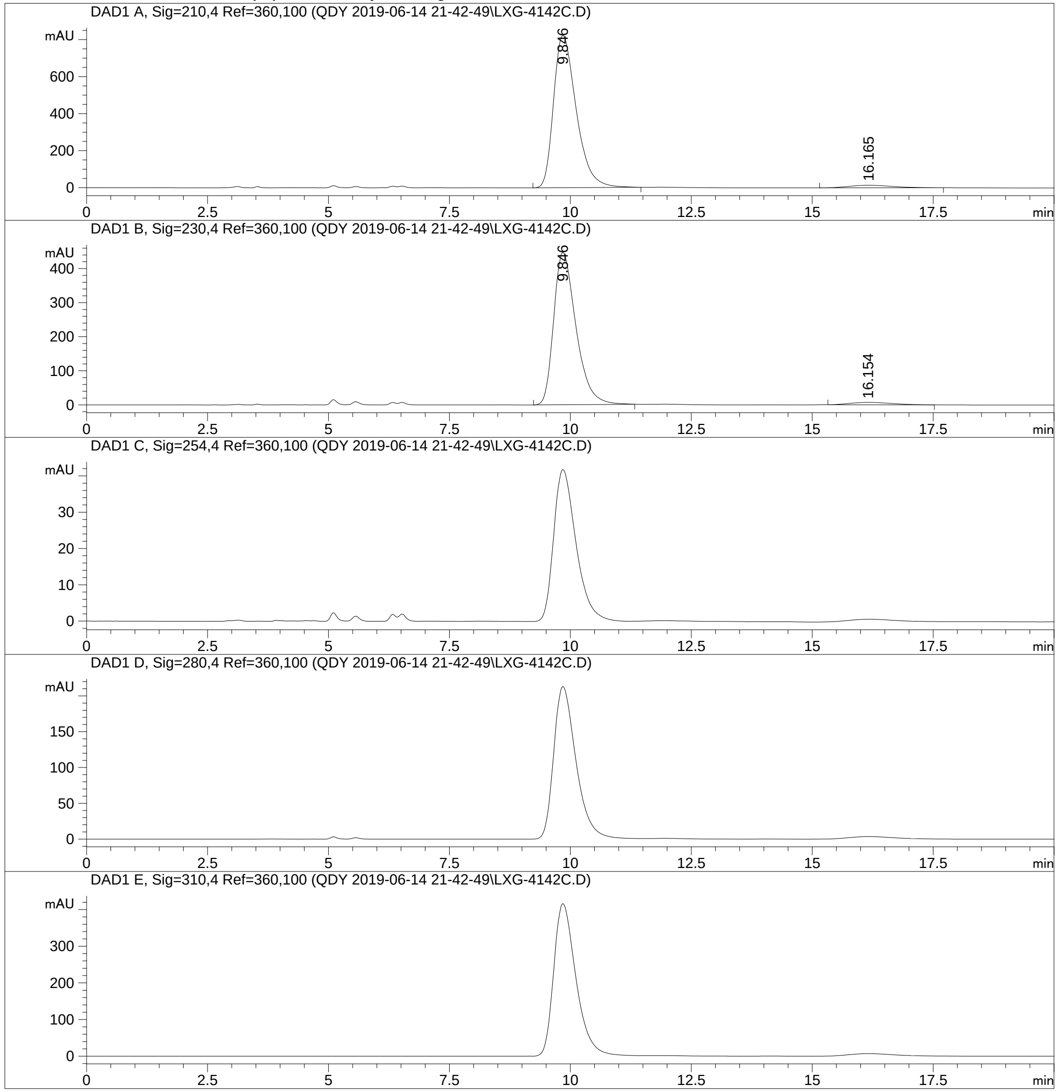

Data File C:\CHEM32\1\DATA\QDY 2019-06-14 21-42-49\LXG-4142C.D
Sample Name:

===
Area Percent Report
===

Sorted By : Signal
Multiplier : 1.0000
Dilution : 1.0000
Use Multiplier & Dilution Factor with ISTDs

Signal 1: DAD1 A, Sig=210,4 Ref=360,100

Peak #	RetTime [min]	Type	Width [min]	Area [mAU*s]	Height [mAU]	Area %
1	9.846	BB	0.5020	2.67951e4	822.72748	96.8372
2	16.165	BB	0.8935	875.14276	13.95693	3.1628

Totals : 2.76703e4 836.68441

Signal 2: DAD1 B, Sig=230,4 Ref=360,100

Peak #	RetTime [min]	Type	Width [min]	Area [mAU*s]	Height [mAU]	Area %
1	9.846	BB	0.4903	1.43359e4	446.82480	97.0729
2	16.154	BB	0.8596	432.28616	7.16802	2.9271

Totals : 1.47682e4 453.99282

Signal 3: DAD1 C, Sig=254,4 Ref=360,100

Signal 4: DAD1 D, Sig=280,4 Ref=360,100

Signal 5: DAD1 E, Sig=310,4 Ref=360,100

===
*** End of Report ***

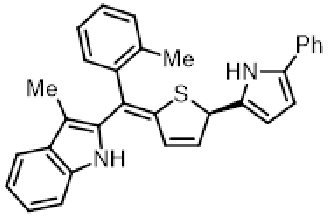

3h
(enantioenriched)

Supplementary Figure 157. HPLC spectrum of **3h** (enantioenriched)

Data File C:\CHEM32\1\DATA\QDY 2020-06-28 16-23-58\LXG-7074C-RAC3.D
Sample Name:

```
=====================================================================
Acq. Operator   :                                 Seq. Line :  15
Acq. Instrument : Instrument 1                     Location : Vial 65
Injection Date  : 6/28/2020 10:41:11 PM                 Inj :   1
                                                 Inj Volume : 5.000 µl
Different Inj Volume from Sequence !     Actual Inj Volume : 4.000 µl
Acq. Method     : C:\CHEM32\1\DATA\QDY 2020-06-28 16-23-58\AD-30-40.M
Last changed    : 6/28/2020 10:40:18 PM
                  (modified after loading)
Analysis Method : C:\CHEM32\1\METHODS\LXG-IC-10-10.M
Last changed    : 7/4/2020 3:11:19 PM
                  (modified after loading)
Additional Info : Peak(s) manually integrated
```

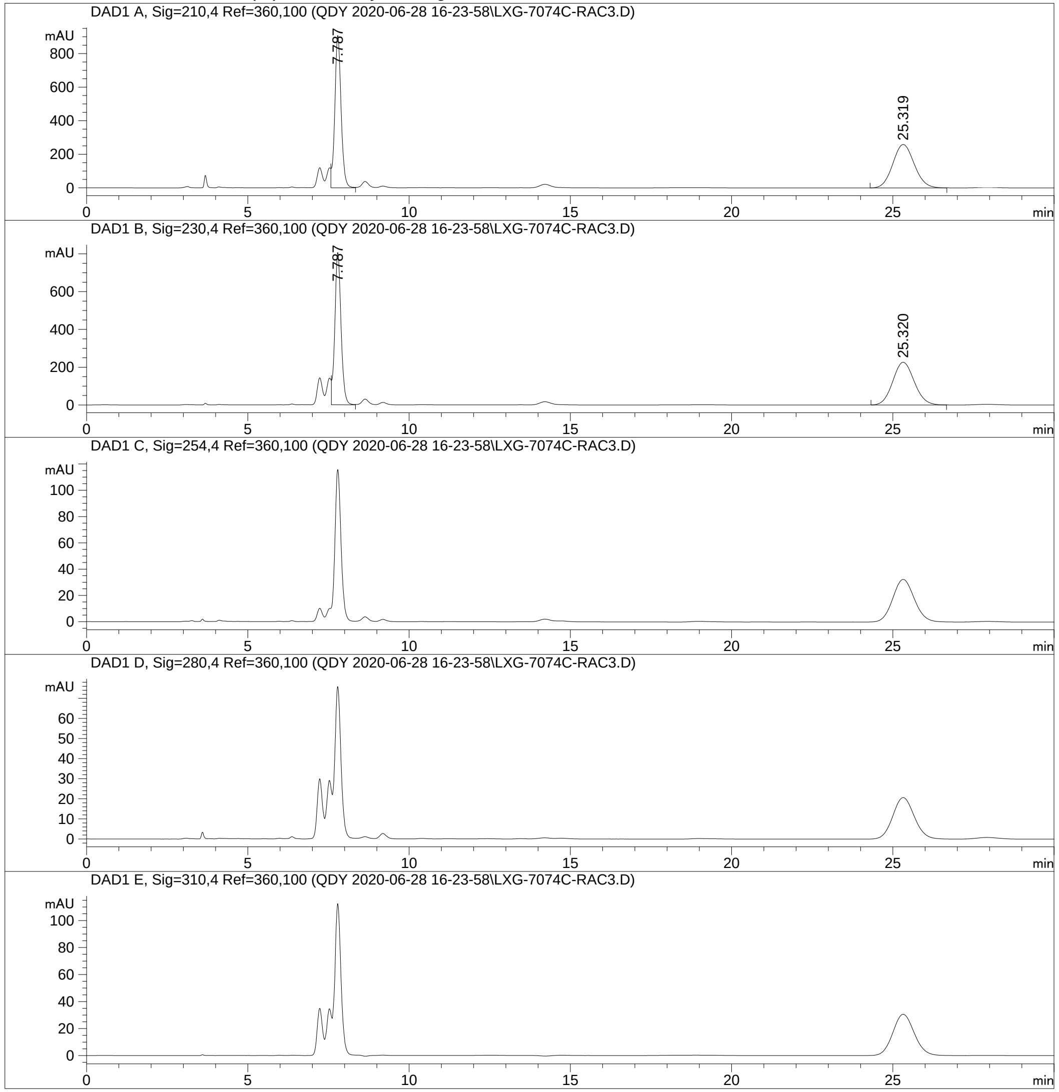

Data File C:\CHEM32\1\DATA\QDY 2020-06-28 16-23-58\LXG-7074C-RAC3.D
Sample Name:

===
Area Percent Report
===

Sorted By : Signal
Multiplier : 1.0000
Dilution : 1.0000
Use Multiplier & Dilution Factor with ISTDs

Signal 1: DAD1 A, Sig=210,4 Ref=360,100

Peak #	RetTime [min]	Type	Width [min]	Area [mAU*s]	Height [mAU]	Area %
1	7.787	VV	0.2058	1.22812e4	908.22375	50.5301
2	25.319	BB	0.7240	1.20235e4	258.81998	49.4699

Totals : 2.43047e4 1167.04373

Signal 2: DAD1 B, Sig=230,4 Ref=360,100

Peak #	RetTime [min]	Type	Width [min]	Area [mAU*s]	Height [mAU]	Area %
1	7.787	VV	0.2047	1.08387e4	806.81927	50.7838
2	25.320	BB	0.7236	1.05042e4	226.28265	49.2162

Totals : 2.13429e4 1033.10193

Signal 3: DAD1 C, Sig=254,4 Ref=360,100

Signal 4: DAD1 D, Sig=280,4 Ref=360,100

Signal 5: DAD1 E, Sig=310,4 Ref=360,100

===
*** End of Report ***

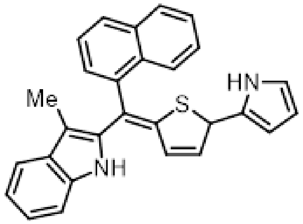

3i
(racemic)

Supplementary Figure 158. HPLC spectrum of **3i** (racemic)

===

Acq. Operator : Seq. Line : 7
Acq. Instrument : Instrument 1 Location : Vial 67
Injection Date : 7/1/2020 10:58:10 PM Inj : 1
Inj Volume : 5.000 µl
Different Inj Volume from Sequence ! Actual Inj Volume : 4.000 µl
Acq. Method : C:\CHEM32\1\DATA\QDY 2020-07-01 20-49-19\AD-30-40.M
Last changed : 7/1/2020 10:57:18 PM
(modified after loading)
Analysis Method : C:\CHEM32\1\METHODS\LXG-IC-10-10.M
Last changed : 7/4/2020 3:05:35 PM
(modified after loading)
Additional Info : Peak(s) manually integrated

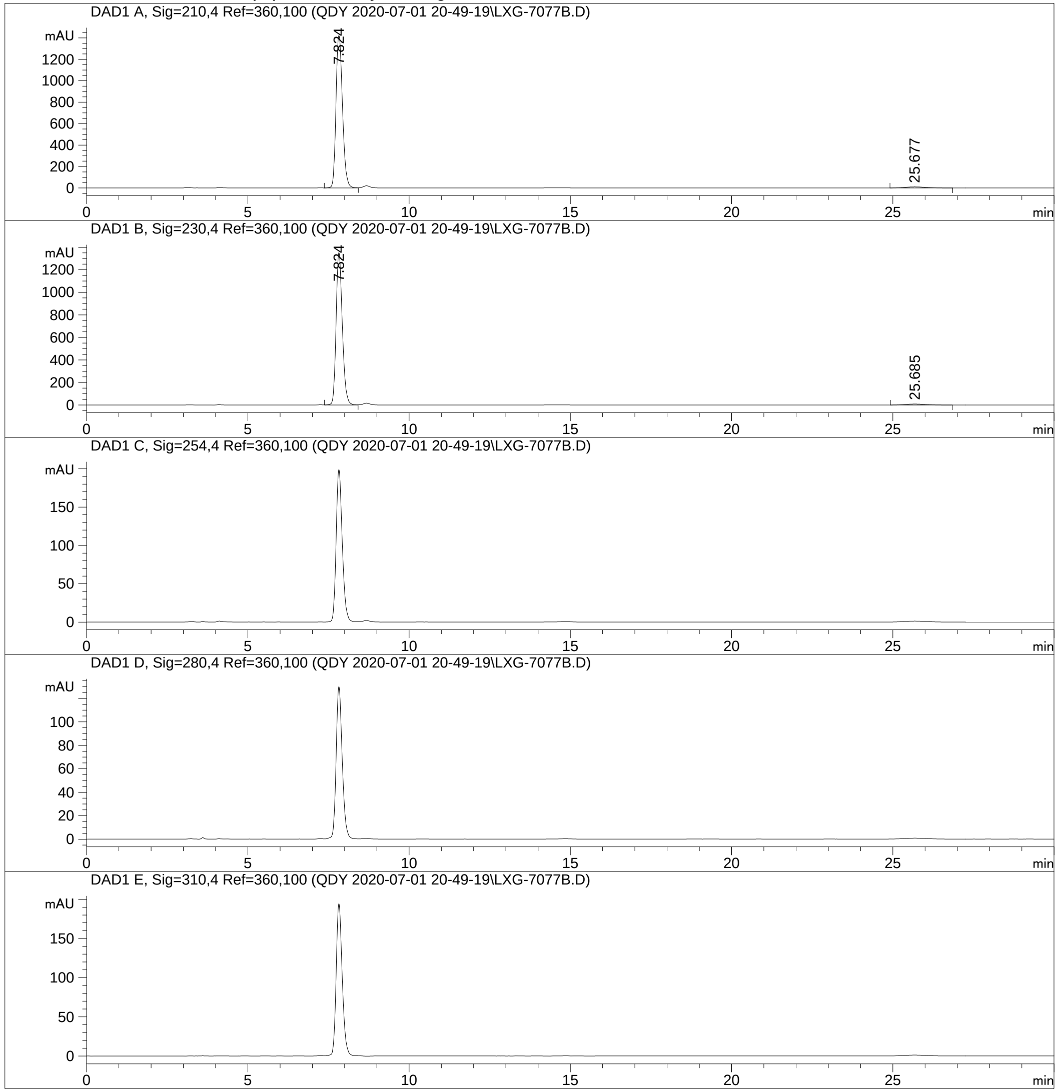

Data File C:\CHEM32\1\DATA\QDY 2020-07-01 20-49-19\LXG-7077B.D
Sample Name:

===

Area Percent Report

===

Sorted By : Signal
Multiplier : 1.0000
Dilution : 1.0000
Use Multiplier & Dilution Factor with ISTDs

Signal 1: DAD1 A, Sig=210,4 Ref=360,100

Peak #	RetTime [min]	Type	Width [min]	Area [mAU*s]	Height [mAU]	Area %
1	7.824	VB	0.2172	1.99111e4	1422.99902	97.7720
2	25.677	BB	0.6922	453.73734	9.65661	2.2280

Totals : 2.03648e4 1432.65563

Signal 2: DAD1 B, Sig=230,4 Ref=360,100

Peak #	RetTime [min]	Type	Width [min]	Area [mAU*s]	Height [mAU]	Area %
1	7.824	VB	0.2063	1.81337e4	1353.30127	97.8936
2	25.685	BB	0.7127	390.19366	8.38851	2.1064

Totals : 1.85239e4 1361.68978

Signal 3: DAD1 C, Sig=254,4 Ref=360,100

Signal 4: DAD1 D, Sig=280,4 Ref=360,100

Signal 5: DAD1 E, Sig=310,4 Ref=360,100

===

*** End of Report ***

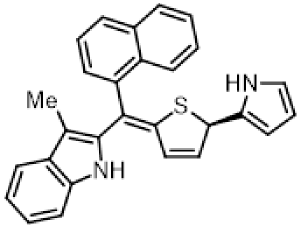

3i
(enantioenriched)

Supplementary Figure 159. HPLC spectrum of **3i** (enantioenriched)

```
Data File C:\CHEM32\1\DATA\QDY 2019-05-12 21-38-28\LXG-4114C-RAC.D
Sample Name:

    =====================================================================
    Acq. Operator   :                               Seq. Line :   3
    Acq. Instrument : Instrument 1                   Location : Vial 75
    Injection Date  : 5/12/2019 10:33:12 PM               Inj :   1
                                                   Inj Volume : 5.000 µl
    Acq. Method     : C:\CHEM32\1\DATA\QDY 2019-05-12 21-38-28\OD-20-40.M
    Last changed    : 5/12/2019 9:50:42 PM
                      (modified after loading)
    Analysis Method : C:\CHEM32\1\METHODS\AD-07-60.M
    Last changed    : 5/14/2019 9:51:54 PM
                      (modified after loading)
    Additional Info : Peak(s) manually integrated
```

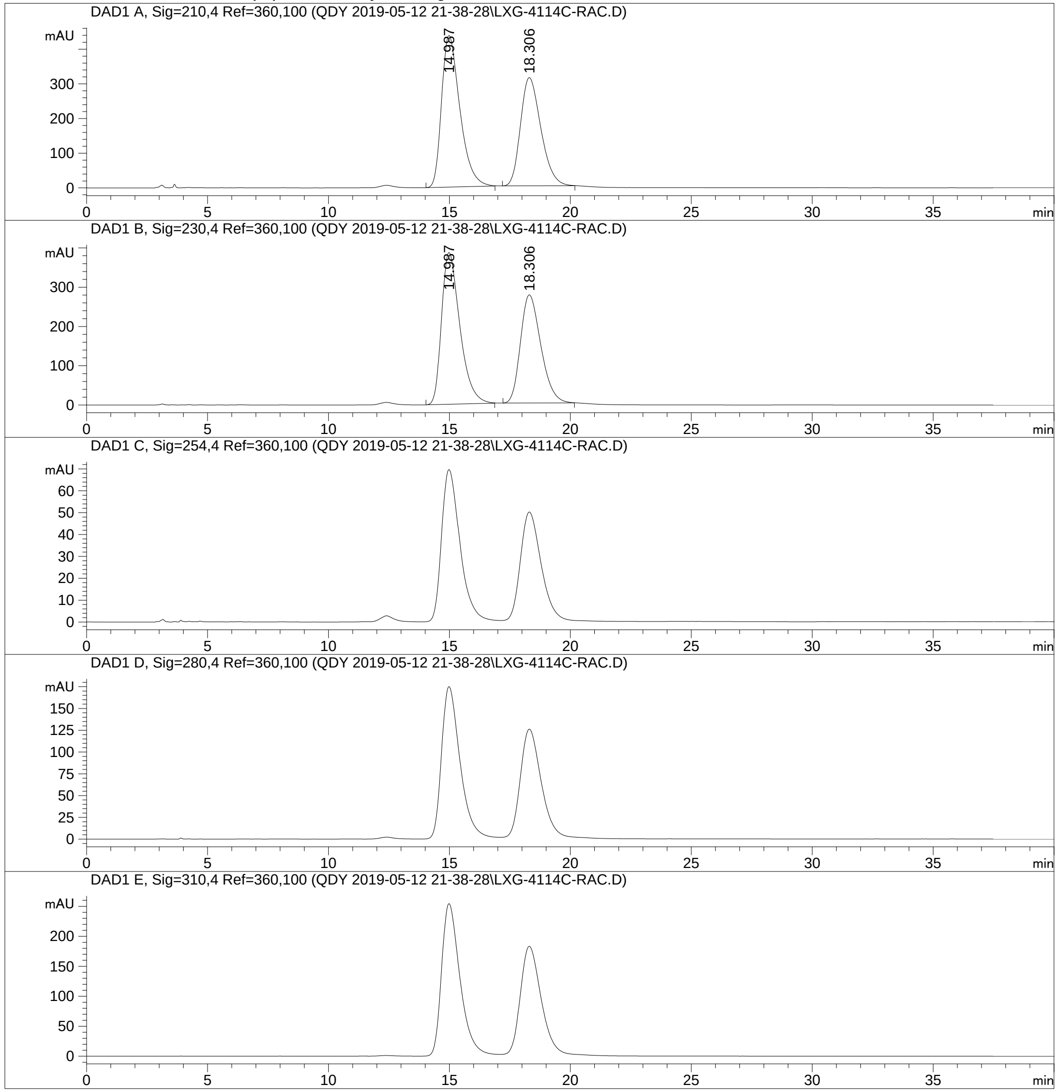


```
Data File C:\CHEM32\1\DATA\QDY 2019-05-12 21-38-28\LXG-4114C-RAC.D
Sample Name:
```

===

Area Percent Report

===

Sorted By : Signal
Multiplier : 1.0000
Dilution : 1.0000
Use Multiplier & Dilution Factor with ISTDs

Signal 1: DAD1 A, Sig=210,4 Ref=360,100

Peak #	RetTime [min]	Type	Width [min]	Area [mAU*s]	Height [mAU]	Area %
1	14.987	BB	0.8076	2.29960e4	437.23770	55.6941
2	18.306	BB	0.9118	1.82939e4	312.10123	44.3059

Totals : 4.12899e4 749.33893

Signal 2: DAD1 B, Sig=230,4 Ref=360,100

Peak #	RetTime [min]	Type	Width [min]	Area [mAU*s]	Height [mAU]	Area %
1	14.987	BB	0.8091	2.03070e4	386.39313	55.7363
2	18.306	BB	0.9070	1.61271e4	275.48026	44.2637

Totals : 3.64341e4 661.87338

Signal 3: DAD1 C, Sig=254,4 Ref=360,100

Signal 4: DAD1 D, Sig=280,4 Ref=360,100

Signal 5: DAD1 E, Sig=310,4 Ref=360,100

===

*** End of Report ***

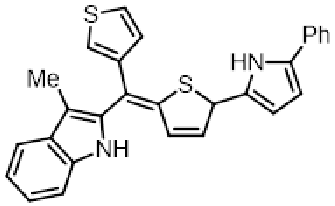

3l
(racemic)

Supplementary Figure 160. HPLC spectrum of **3l** (racemic)

Data File C:\CHEM32\1\DATA\QDY 2019-05-14 13-58-33\LXG-4116C.D
Sample Name:

===

Acq. Operator	:	Seq. Line :	13
Acq. Instrument	: Instrument 1	Location :	Vial 73
Injection Date	: 5/14/2019 6:06:42 PM	Inj :	1
		Inj Volume :	5.000 µl

Acq. Method : C:\CHEM32\1\DATA\QDY 2019-05-14 13-58-33\OD-20-40.M
Last changed : 5/14/2019 5:23:58 PM
(modified after loading)
Analysis Method : C:\CHEM32\1\METHODS\AD-07-60.M
Last changed : 5/11/2019 10:45:40 AM
(modified after loading)
Additional Info : Peak(s) manually integrated

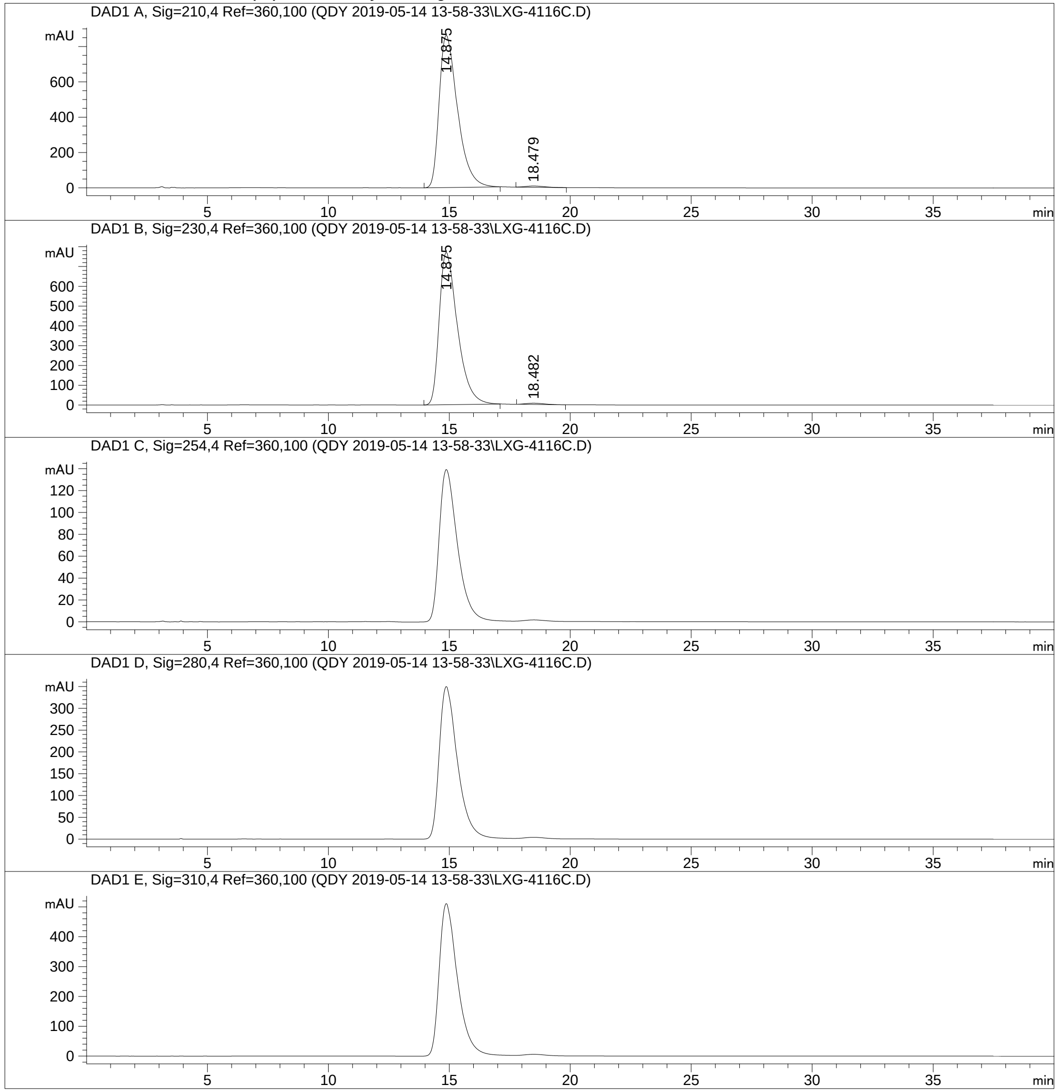

Data File C:\CHEM32\1\DATA\QDY 2019-05-14 13-58-33\LXG-4116C.D
Sample Name:

===
Area Percent Report
===

Sorted By : Signal
Multiplier : 1.0000
Dilution : 1.0000
Use Multiplier & Dilution Factor with ISTDs

Signal 1: DAD1 A, Sig=210,4 Ref=360,100

Peak #	RetTime [min]	Type	Width [min]	Area [mAU*s]	Height [mAU]	Area %
1	14.875	BB	0.8070	4.55299e4	860.87372	99.1609
2	18.479	BB	0.7376	385.25839	7.25471	0.8391
Totals :				4.59151e4	868.12842	

Signal 2: DAD1 B, Sig=230,4 Ref=360,100

Peak #	RetTime [min]	Type	Width [min]	Area [mAU*s]	Height [mAU]	Area %
1	14.875	BB	0.8032	4.04170e4	768.98621	99.1820
2	18.482	BB	0.7998	333.31796	6.39790	0.8180
Totals :				4.07503e4	775.38411	

Signal 3: DAD1 C, Sig=254,4 Ref=360,100

Signal 4: DAD1 D, Sig=280,4 Ref=360,100

Signal 5: DAD1 E, Sig=310,4 Ref=360,100

===
*** End of Report ***

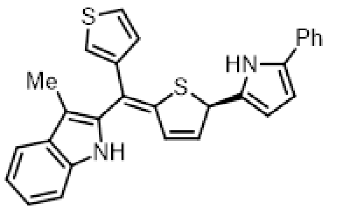

3j
(enantioenriched)

Supplementary Figure 161. HPLC spectrum of **3j** (enantioenriched)

```
=====================================================================
Acq. Operator   :                                 Seq. Line :   5
Acq. Instrument : Instrument 1                     Location : Vial 63
Injection Date  : 6/12/2019 10:05:17 PM                 Inj :   1
                                               Inj Volume : 5.000 µl
Acq. Method     : C:\CHEM32\1\DATA\QDY 2019-06-12 20-18-23\OD-20-30.M
Last changed    : 5/3/2016 10:14:35 AM
Analysis Method : C:\CHEM32\1\METHODS\OD-01-45-0.5.M
Last changed    : 8/20/2020 6:54:41 PM
                  (modified after loading)
Additional Info : Peak(s) manually integrated
```

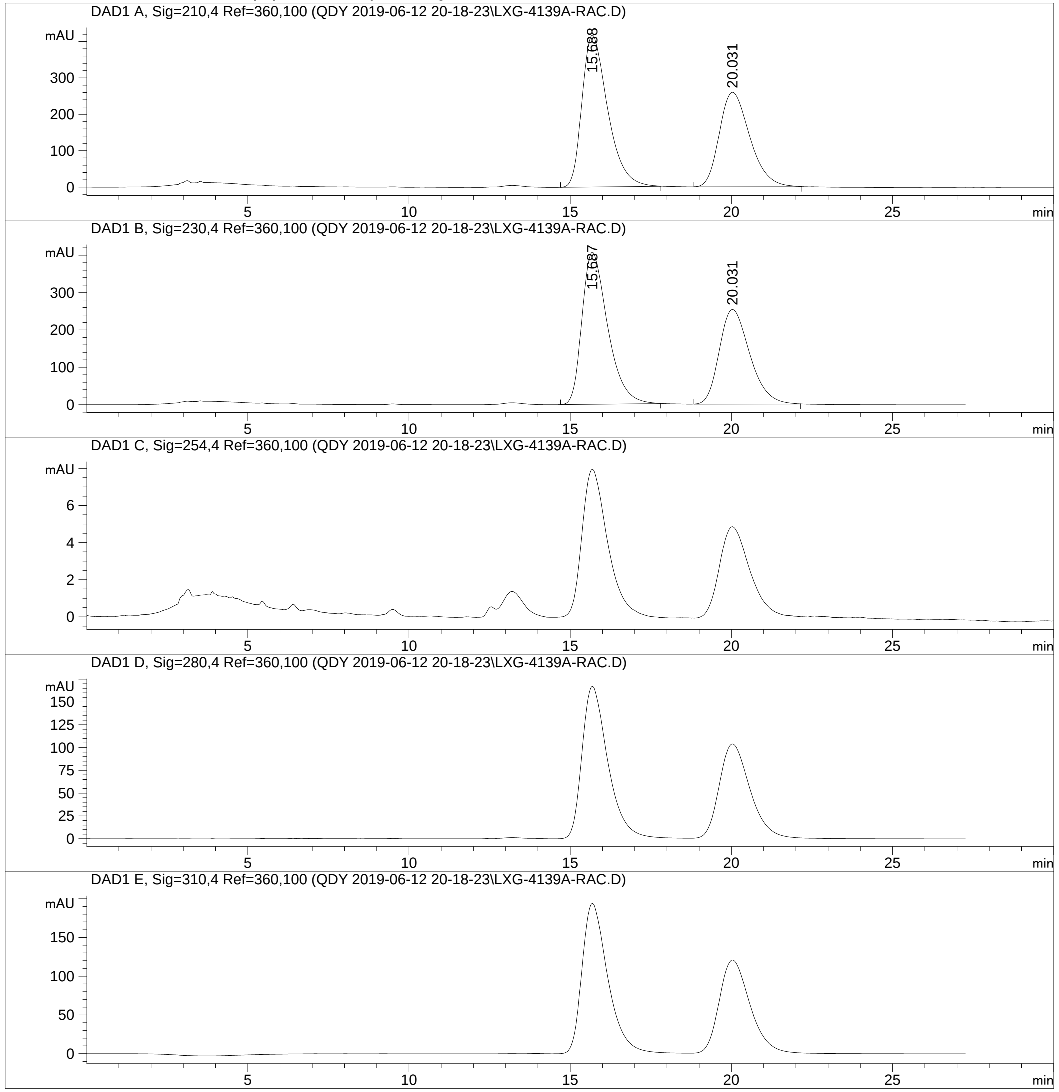

Data File C:\CHEM32\1\DATA\QDY 2019-06-12 20-18-23\LXG-4139A-RAC.D
Sample Name:

```
=====================================================================
                         Area Percent Report
=====================================================================

Sorted By             :      Signal
Multiplier            :      1.0000
Dilution              :      1.0000
Use Multiplier & Dilution Factor with ISTDs


Signal 1: DAD1 A, Sig=210,4 Ref=360,100

Peak RetTime Type  Width     Area      Height     Area
  #   [min]        [min]   [mAU*s]     [mAU]        %
----|-------|----|-------|----------|----------|--------|
   1  15.688 BB    0.8527 2.31229e4   417.12521  57.4932
   2  20.031 BB    1.0194 1.70956e4   259.88626  42.5068

Totals :                  4.02185e4   677.01147


Signal 2: DAD1 B, Sig=230,4 Ref=360,100

Peak RetTime Type  Width     Area      Height     Area
  #   [min]        [min]   [mAU*s]     [mAU]        %
----|-------|----|-------|----------|----------|--------|
   1  15.687 BB    0.8498 2.25266e4   406.93353  57.5159
   2  20.031 BB    1.0204 1.66393e4   253.28590  42.4841

Totals :                  3.91658e4   660.21944


Signal 3: DAD1 C, Sig=254,4 Ref=360,100


Signal 4: DAD1 D, Sig=280,4 Ref=360,100


Signal 5: DAD1 E, Sig=310,4 Ref=360,100

=====================================================================
                          *** End of Report ***
```

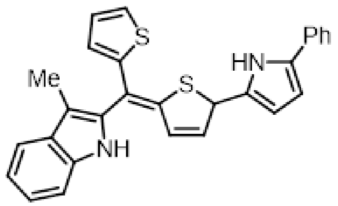

3k
(racemic)

Supplementary Figure 162. HPLC spectrum of **3k** (racemic)

Acq. Operator	:	Seq. Line :	2
Acq. Instrument	: Instrument 1	Location :	Vial 65
Injection Date	: 6/14/2019 9:56:07 PM	Inj :	1
		Inj Volume :	5.000 µl

Acq. Method : C:\CHEM32\1\DATA\QDY 2019-06-14 21-42-49\OD-20-30.M
Last changed : 5/3/2016 10:14:35 AM
Analysis Method : C:\CHEM32\1\METHODS\OD-01-45-0.5.M
Last changed : 8/20/2020 6:54:41 PM
(modified after loading)
Additional Info : Peak(s) manually integrated

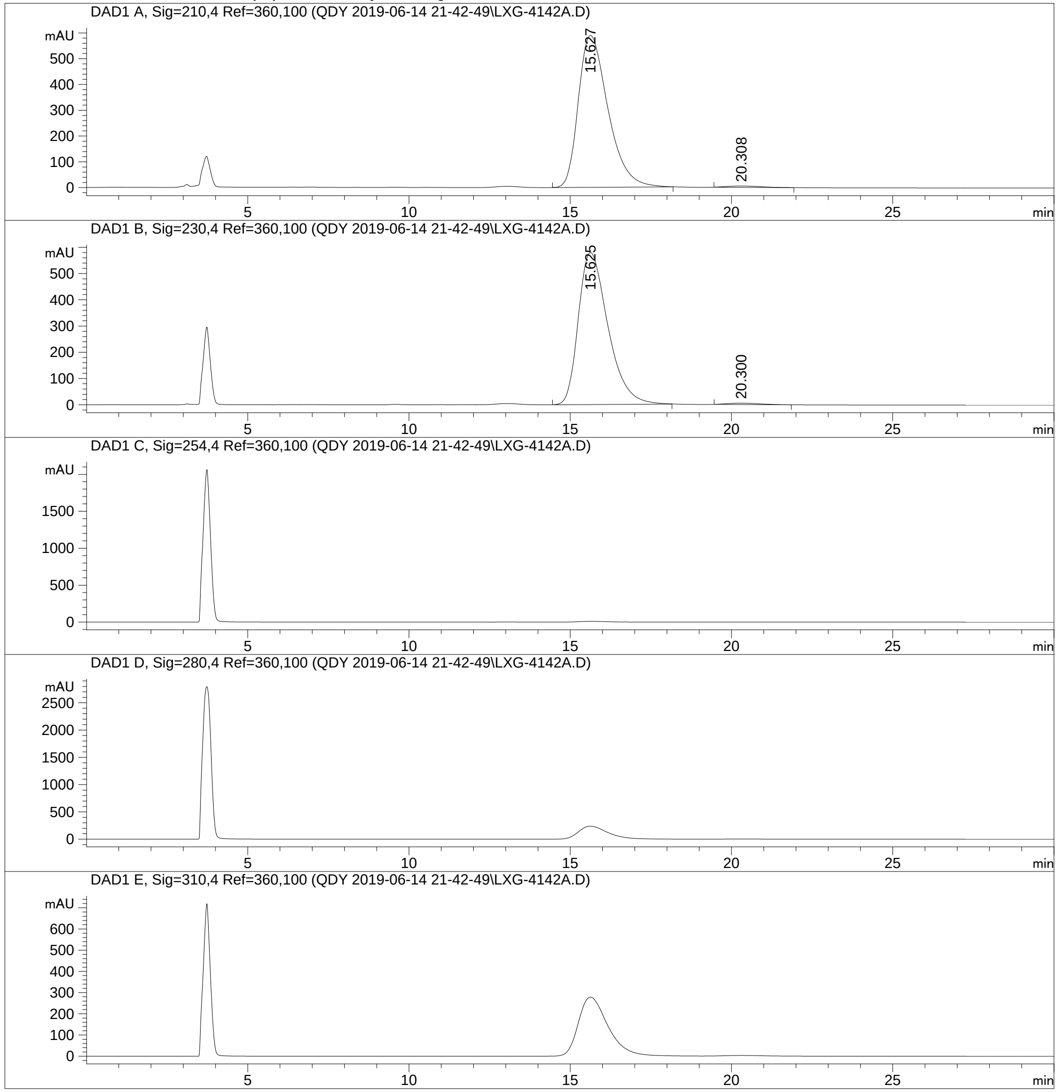

Data File C:\CHEM32\1\DATA\QDY 2019-06-14 21-42-49\LXG-4142A.D
Sample Name:

Area Percent Report

Sorted By : Signal
Multiplier : 1.0000
Dilution : 1.0000
Use Multiplier & Dilution Factor with ISTDs

Signal 1: DAD1 A, Sig=210,4 Ref=360,100

Peak #	RetTime [min]	Type	Width [min]	Area [mAU*s]	Height [mAU]	Area %
1	15.627	BB	0.9779	3.76749e4	589.29059	98.9965
2	20.308	BB	0.8524	381.91217	5.43105	1.0035

Totals : 3.80568e4 594.72163

Signal 2: DAD1 B, Sig=230,4 Ref=360,100

Peak #	RetTime [min]	Type	Width [min]	Area [mAU*s]	Height [mAU]	Area %
1	15.625	BB	0.9774	3.69196e4	579.39685	99.0134
2	20.300	BB	1.0100	367.89136	5.25984	0.9866

Totals : 3.72875e4 584.65669

Signal 3: DAD1 C, Sig=254,4 Ref=360,100

Signal 4: DAD1 D, Sig=280,4 Ref=360,100

Signal 5: DAD1 E, Sig=310,4 Ref=360,100

*** End of Report ***

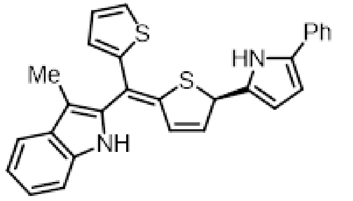

3k
(enantioenriched)

Supplementary Figure 163. HPLC spectrum of **3k** (enantioenriched)

Data File C:\CHEM32\1\DATA\QDY 2020-07-13 23-41-55\LXG-7089B3-RAC.D
Sample Name:

===

Acq. Operator : Seq. Line : 4
Acq. Instrument : Instrument 1 Location : Vial 63
Injection Date : 7/14/2020 12:57:18 AM Inj : 1
Inj Volume : 5.000 µl
Different Inj Volume from Sequence ! Actual Inj Volume : 3.000 µl
Acq. Method : C:\CHEM32\1\DATA\QDY 2020-07-13 23-41-55\AD-20-30.M
Last changed : 6/15/2018 10:29:43 AM
Analysis Method : C:\CHEM32\1\METHODS\AD-008-50.M
Last changed : 7/10/2020 9:21:13 AM
Additional Info : Peak(s) manually integrated

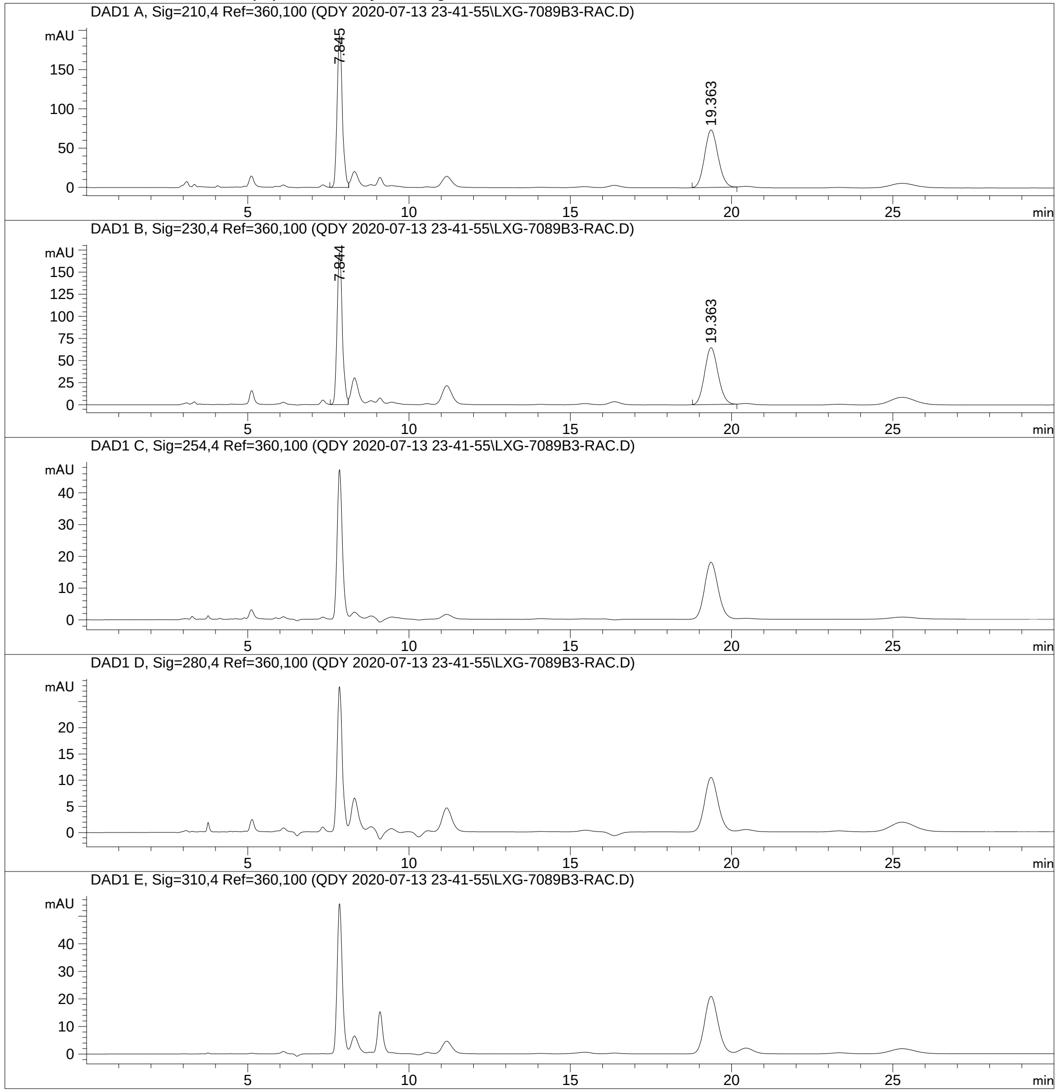

Data File C:\CHEM32\1\DATA\QDY 2020-07-13 23-41-55\LXG-7089B3-RAC.D
Sample Name:

===

Area Percent Report

===

Sorted By : Signal
Multiplier : 1.0000
Dilution : 1.0000
Use Multiplier & Dilution Factor with ISTDs

Signal 1: DAD1 A, Sig=210,4 Ref=360,100

Peak #	RetTime [min]	Type	Width [min]	Area [mAU*s]	Height [mAU]	Area %
1	7.845	VV	0.1740	2189.89258	193.54507	50.7406
2	19.363	BB	0.4509	2125.96704	73.18436	49.2594

Totals : 4315.85962 266.72943

Signal 2: DAD1 B, Sig=230,4 Ref=360,100

Peak #	RetTime [min]	Type	Width [min]	Area [mAU*s]	Height [mAU]	Area %
1	7.844	VV	0.1742	1949.09778	171.97502	51.0868
2	19.363	BB	0.4507	1866.16663	64.28641	48.9132

Totals : 3815.26440 236.26143

Signal 3: DAD1 C, Sig=254,4 Ref=360,100

Signal 4: DAD1 D, Sig=280,4 Ref=360,100

Signal 5: DAD1 E, Sig=310,4 Ref=360,100

===

*** End of Report ***

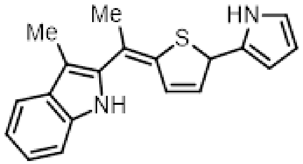

3l
(racemic)

Supplementary Figure 164. HPLC spectrum of **3l** (racemic)

```
=====================================================================
Acq. Operator   :                                Seq. Line :  24
Acq. Instrument : Instrument 1                    Location : Vial 62
Injection Date  : 7/16/2020 12:21:39 AM                Inj :   1
                                               Inj Volume : 5.000 µl
Different Inj Volume from Sequence !     Actual Inj Volume : 4.000 µl
Acq. Method     : C:\CHEM32\1\DATA\QDY 2020-07-15 16-33-43\AD-20-30.M
Last changed    : 6/15/2018 10:29:43 AM
Analysis Method : C:\CHEM32\1\METHODS\AD-008-50.M
Last changed    : 7/10/2020 9:21:13 AM
Additional Info : Peak(s) manually integrated
```

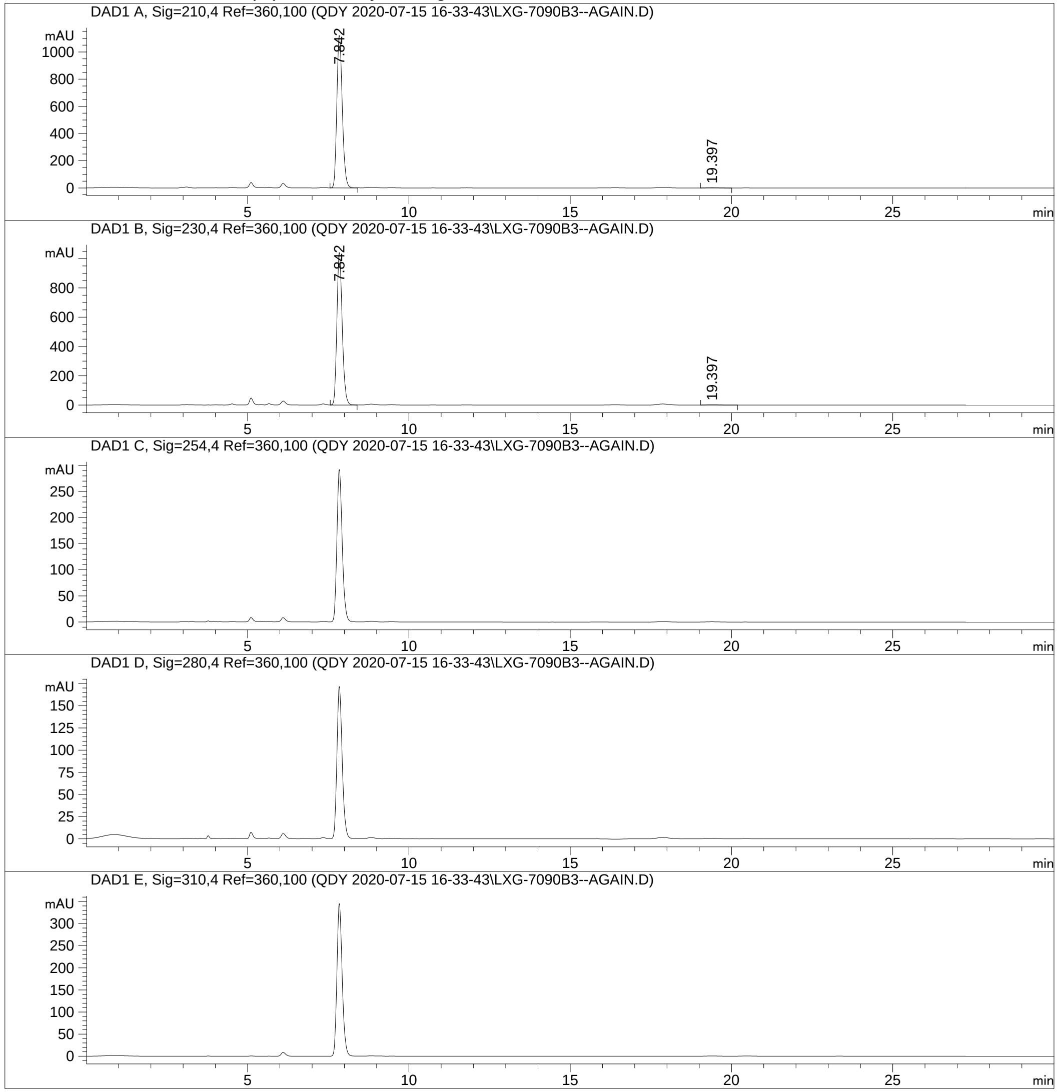

Data File C:\CHEM32\1\DATA\QDY 2020-07-15 16-33-43\LXG-7090B3--AGAIN.D
Sample Name:

```
=====================================================================
                         Area Percent Report
=====================================================================

Sorted By             :      Signal
Multiplier            :      1.0000
Dilution              :      1.0000
Use Multiplier & Dilution Factor with ISTDs


Signal 1: DAD1 A, Sig=210,4 Ref=360,100

Peak RetTime Type  Width     Area      Height     Area
  #   [min]        [min]   [mAU*s]     [mAU]        %
----|-------|----|-------|----------|----------|--------|
   1   7.842 VB    0.1811 1.31779e4  1121.64160  99.5431
   2  19.397 BB    0.3991   60.47977    2.10594   0.4569

Totals :                  1.32384e4  1123.74754


Signal 2: DAD1 B, Sig=230,4 Ref=360,100

Peak RetTime Type  Width     Area      Height     Area
  #   [min]        [min]   [mAU*s]     [mAU]        %
----|-------|----|-------|----------|----------|--------|
   1   7.842 VB    0.1757 1.19394e4  1042.07349  99.6063
   2  19.397 BB    0.4287   47.18557    1.77169   0.3937

Totals :                  1.19866e4  1043.84518


Signal 3: DAD1 C, Sig=254,4 Ref=360,100


Signal 4: DAD1 D, Sig=280,4 Ref=360,100


Signal 5: DAD1 E, Sig=310,4 Ref=360,100

=====================================================================
                         *** End of Report ***
```

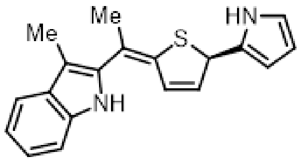

3l
(enantioenriched)

Supplementary Figure 165. HPLC spectrum of **3l** (enantioenriched)

Data File C:\CHEM32\1\DATA\QDY 2020-07-13 23-41-55\LXG-7089B2-RAC2.D
Sample Name:

```
=====================================================================
Acq. Operator   :                              Seq. Line :  10
Acq. Instrument : Instrument 1                  Location : Vial 62
Injection Date  : 7/14/2020 3:23:45 AM               Inj :   1
                                              Inj Volume : 5.000 µl
Different Inj Volume from Sequence !     Actual Inj Volume : 3.000 µl
Acq. Method     : C:\CHEM32\1\DATA\QDY 2020-07-13 23-41-55\OD-20-30.M
Last changed    : 5/3/2016 10:14:35 AM
Analysis Method : C:\CHEM32\1\DATA\QDY 2020-12-12 14-50-34\L1.D\DA.M
Last changed    : 1/23/2021 10:49:11 PM
                  (modified after loading)
Additional Info : Peak(s) manually integrated
```

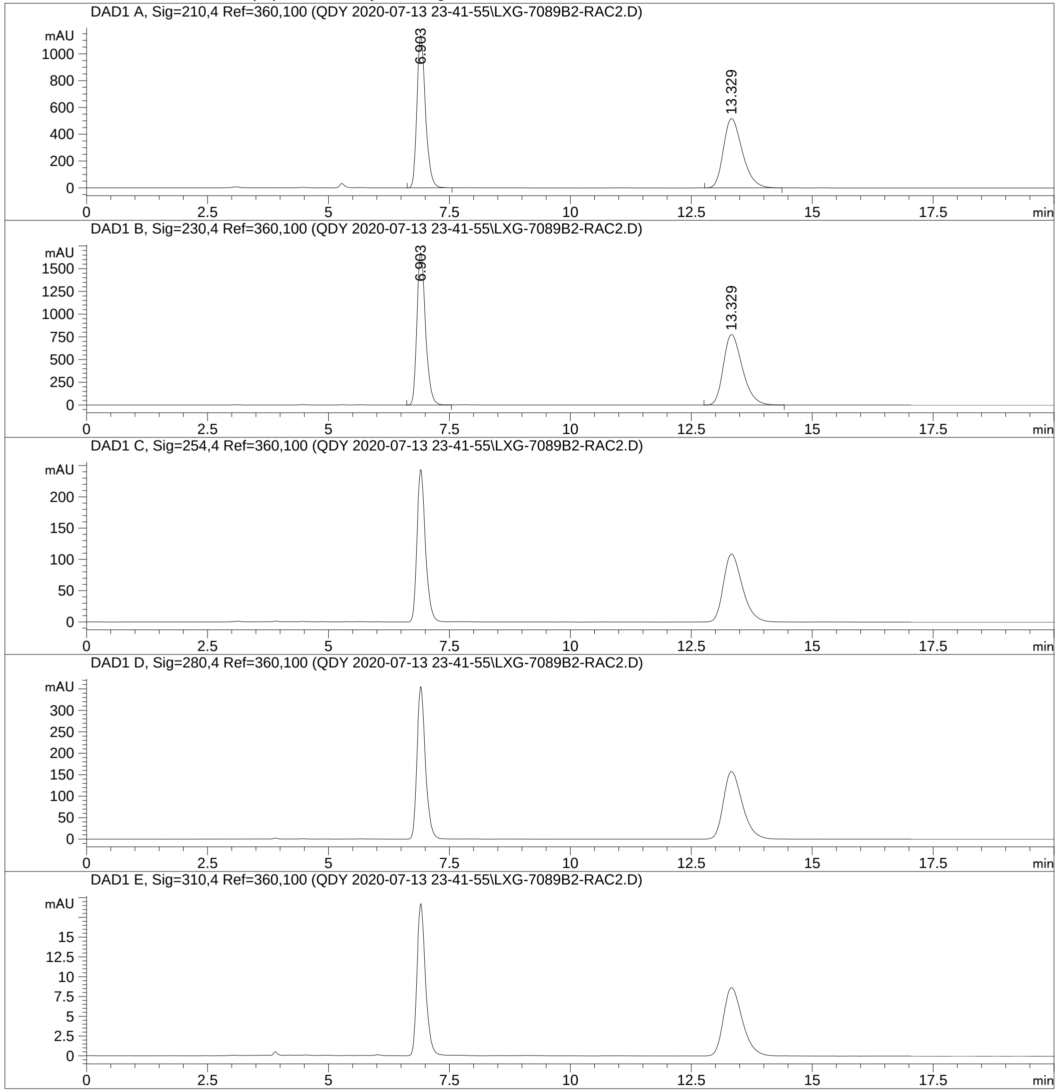

Data File C:\CHEM32\1\DATA\QDY 2020-07-13 23-41-55\LXG-7089B2-RAC2.D
Sample Name:

===
Area Percent Report
===

Sorted By : Signal
Multiplier : 1.0000
Dilution : 1.0000
Use Multiplier & Dilution Factor with ISTDs

Signal 1: DAD1 A, Sig=210,4 Ref=360,100

Peak #	RetTime [min]	Type	Width [min]	Area [mAU*s]	Height [mAU]	Area %
1	6.903	BB	0.1888	1.39130e4	1136.78003	49.6651
2	13.329	BB	0.4197	1.41007e4	517.81897	50.3349
Totals :				2.80138e4	1654.59900	

Signal 2: DAD1 B, Sig=230,4 Ref=360,100

Peak #	RetTime [min]	Type	Width [min]	Area [mAU*s]	Height [mAU]	Area %
1	6.903	BB	0.1902	2.07432e4	1677.82849	49.4895
2	13.329	BB	0.4202	2.11712e4	776.22736	50.5105
Totals :				4.19144e4	2454.05585	

Signal 3: DAD1 C, Sig=254,4 Ref=360,100

Signal 4: DAD1 D, Sig=280,4 Ref=360,100

Signal 5: DAD1 E, Sig=310,4 Ref=360,100

===
*** End of Report ***

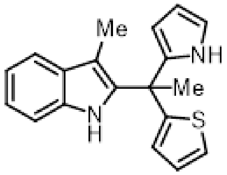

3l'
(racemic)

Supplementary Figure 166. HPLC spectrum of **3l'** (racemic)

```
=====================================================================
Acq. Operator   :                              Seq. Line :  20
Acq. Instrument : Instrument 1                  Location : Vial 61
Injection Date  : 7/15/2020 11:06:54 PM              Inj :   1
                                              Inj Volume : 5.000 µl
Different Inj Volume from Sequence !     Actual Inj Volume : 4.000 µl
Acq. Method     : C:\CHEM32\1\DATA\QDY 2020-07-15 16-33-43\OD-20-20.M
Last changed    : 1/7/2016 10:22:18 AM
Analysis Method : C:\CHEM32\1\DATA\QDY 2020-12-12 14-50-34\L1.D\DA.M
Last changed    : 1/23/2021 10:49:11 PM
                  (modified after loading)
Additional Info : Peak(s) manually integrated
```

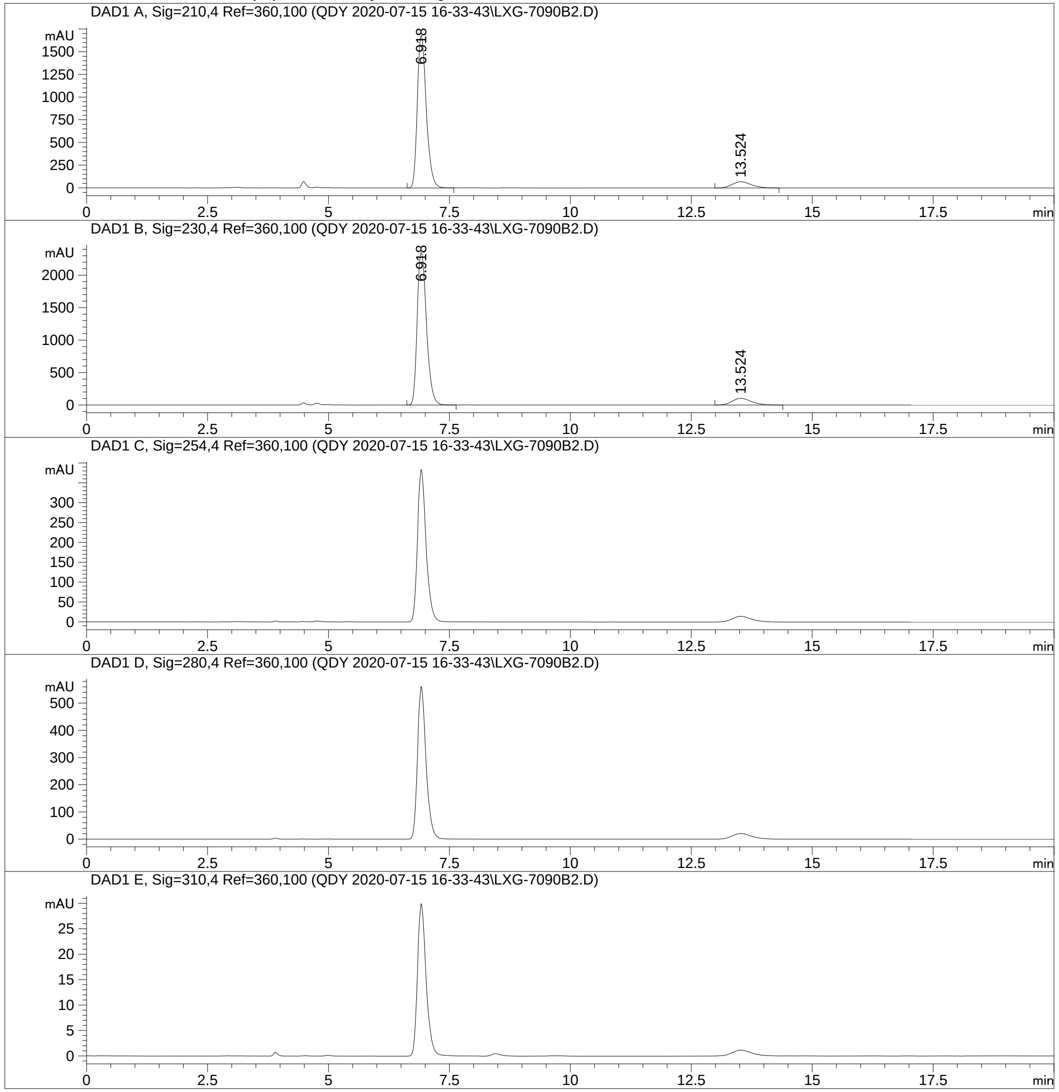

Data File C:\CHEM32\1\DATA\QDY 2020-07-15 16-33-43\LXG-7090B2.D
Sample Name:

```
=====================================================================
                        Area Percent Report
=====================================================================

Sorted By             :      Signal
Multiplier            :      1.0000
Dilution              :      1.0000
Use Multiplier & Dilution Factor with ISTDs


Signal 1: DAD1 A, Sig=210,4 Ref=360,100

Peak RetTime Type  Width     Area      Height     Area
  #   [min]        [min]   [mAU*s]     [mAU]        %
----|-------|----|-------|----------|----------|--------|
   1   6.918 BB    0.2006 2.16919e4  1680.42749  91.9022
   2  13.524 BB    0.4260 1911.34253   68.81486   8.0978

Totals :                  2.36032e4  1749.24235


Signal 2: DAD1 B, Sig=230,4 Ref=360,100

Peak RetTime Type  Width     Area      Height     Area
  #   [min]        [min]   [mAU*s]     [mAU]        %
----|-------|----|-------|----------|----------|--------|
   1   6.918 BB    0.2102 3.14638e4  2350.51831  91.5961
   2  13.524 BB    0.4286 2886.77588  103.76046   8.4039

Totals :                  3.43506e4  2454.27877


Signal 3: DAD1 C, Sig=254,4 Ref=360,100


Signal 4: DAD1 D, Sig=280,4 Ref=360,100


Signal 5: DAD1 E, Sig=310,4 Ref=360,100

=====================================================================
                       *** End of Report ***
```

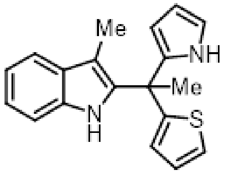

3l'
(enantioenriched)

Supplementary Figure 167. HPLC spectrum of **3l'** (enantioenriched)

Data File C:\CHEM32\1\DATA\QDY 2020-06-29 20-05-44\LXG-7074E-RAC3.D
Sample Name:

===

Acq. Operator : Seq. Line : 4
Acq. Instrument : Instrument 1 Location : Vial 61
Injection Date : 6/29/2020 9:10:49 PM Inj : 1
Inj Volume : 5.000 µl
Different Inj Volume from Sequence ! Actual Inj Volume : 3.000 µl
Acq. Method : C:\CHEM32\1\DATA\QDY 2020-06-29 20-05-44\AD-10-20.M
Last changed : 6/29/2020 9:09:59 PM
(modified after loading)
Analysis Method : C:\CHEM32\1\METHODS\LXG-IC-10-10.M
Last changed : 7/4/2020 3:16:15 PM
(modified after loading)
Additional Info : Peak(s) manually integrated

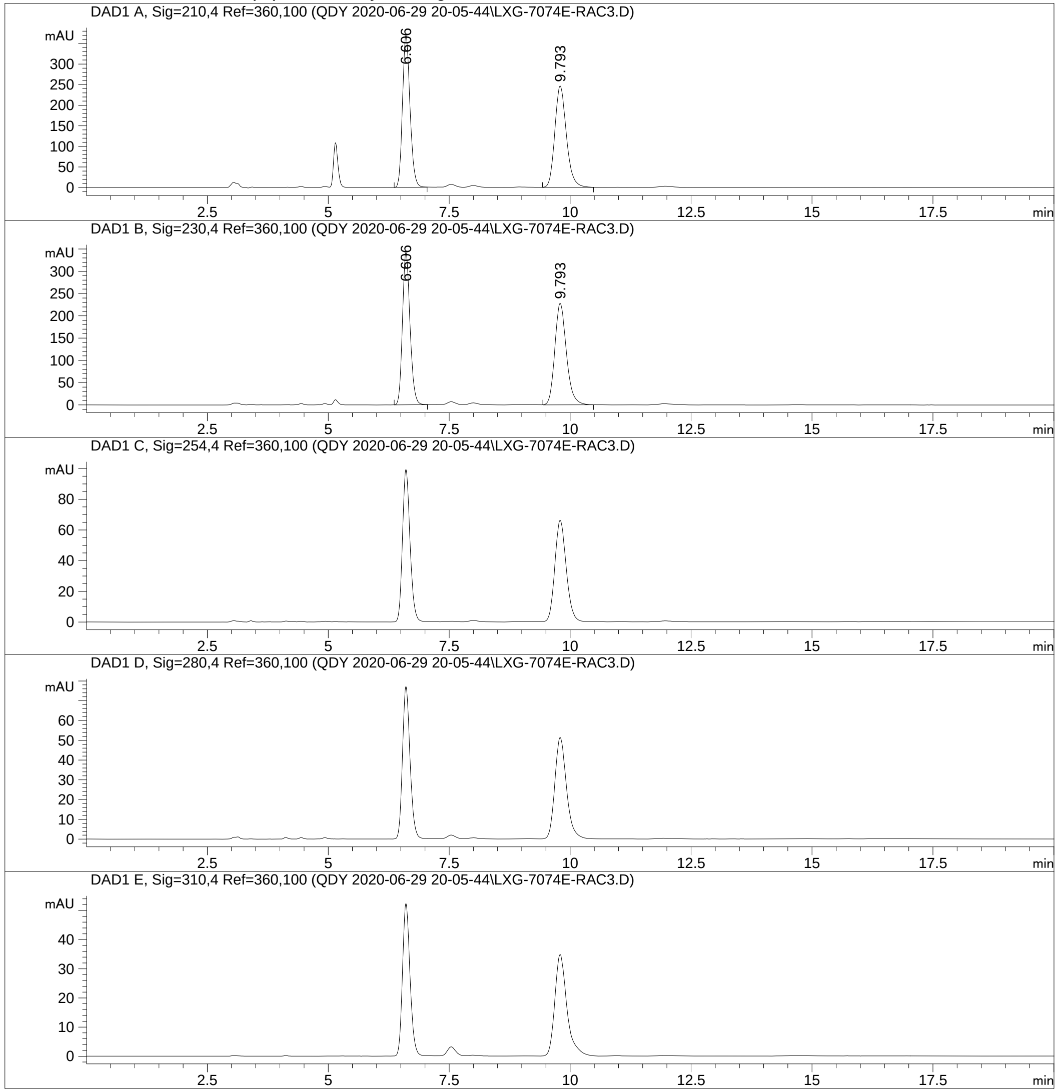

Data File C:\CHEM32\1\DATA\QDY 2020-06-29 20-05-44\LXG-7074E-RAC3.D
Sample Name:

===

Area Percent Report

===

Sorted By : Signal
Multiplier : 1.0000
Dilution : 1.0000
Use Multiplier & Dilution Factor with ISTDs

Signal 1: DAD1 A, Sig=210,4 Ref=360,100

Peak #	RetTime [min]	Type	Width [min]	Area [mAU*s]	Height [mAU]	Area %
1	6.606	BB	0.1663	3998.21582	369.46530	49.3345
2	9.793	BB	0.2563	4106.08936	246.48531	50.6655

Totals : 8104.30518 615.95061

Signal 2: DAD1 B, Sig=230,4 Ref=360,100

Peak #	RetTime [min]	Type	Width [min]	Area [mAU*s]	Height [mAU]	Area %
1	6.606	BB	0.1661	3697.47021	342.10355	49.3400
2	9.793	BB	0.2564	3796.38501	227.85043	50.6600

Totals : 7493.85522 569.95398

Signal 3: DAD1 C, Sig=254,4 Ref=360,100

Signal 4: DAD1 D, Sig=280,4 Ref=360,100

Signal 5: DAD1 E, Sig=310,4 Ref=360,100

===

*** End of Report ***

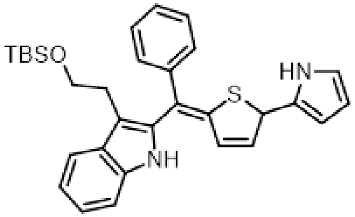

3m
(racemic)

Supplementary Figure 168. HPLC spectrum of **3m** (racemic)

```
=====================================================================
Acq. Operator   :                               Seq. Line :   2
Acq. Instrument : Instrument 1                   Location : Vial 69
Injection Date  : 7/2/2020 10:44:01 PM                Inj :   1
                                               Inj Volume : 5.000 µl
Different Inj Volume from Sequence !     Actual Inj Volume : 4.000 µl
Acq. Method     : C:\CHEM32\1\DATA\QDY 2020-07-02 22-31-08\AD-10-20.M
Last changed    : 7/2/2020 10:43:09 PM
                  (modified after loading)
Analysis Method : C:\CHEM32\1\METHODS\LXG-IC-10-10.M
Last changed    : 7/4/2020 3:07:23 PM
                  (modified after loading)
Additional Info : Peak(s) manually integrated
```

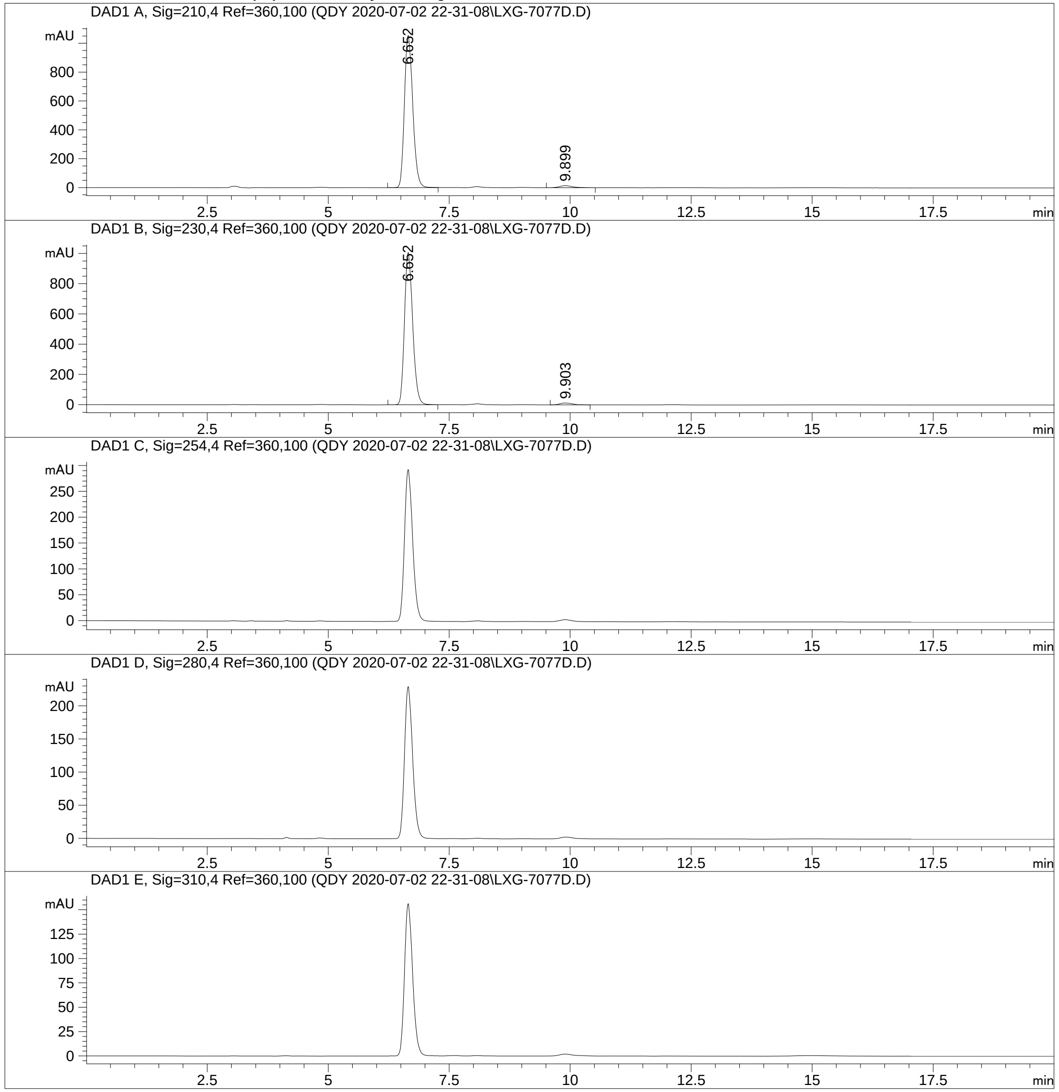

Data File C:\CHEM32\1\DATA\QDY 2020-07-02 22-31-08\LXG-7077D.D
Sample Name:

```
=====================================================================
                         Area Percent Report
=====================================================================

Sorted By             :      Signal
Multiplier            :      1.0000
Dilution              :      1.0000
Use Multiplier & Dilution Factor with ISTDs
```

Signal 1: DAD1 A, Sig=210,4 Ref=360,100

Peak #	RetTime [min]	Type	Width [min]	Area [mAU*s]	Height [mAU]	Area %
1	6.652	BB	0.1882	1.26663e4	1054.37170	97.9792
2	9.899	VB	0.2809	261.24567	14.18415	2.0208

Totals : 1.29275e4 1068.55585

Signal 2: DAD1 B, Sig=230,4 Ref=360,100

Peak #	RetTime [min]	Type	Width [min]	Area [mAU*s]	Height [mAU]	Area %
1	6.652	BB	0.1840	1.19065e4	1006.84686	98.1388
2	9.903	BB	0.2729	225.81224	12.60866	1.8612

Totals : 1.21323e4 1019.45552

Signal 3: DAD1 C, Sig=254,4 Ref=360,100

Signal 4: DAD1 D, Sig=280,4 Ref=360,100

Signal 5: DAD1 E, Sig=310,4 Ref=360,100

```
=====================================================================
                        *** End of Report ***
```

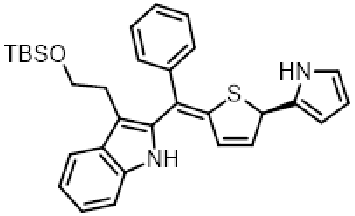

3m
(enantioenriched)

Supplementary Figure 169. HPLC spectrum of **3m** (enantioenriched)

Data File C:\CHEM32\1\DATA\QDY 2020-06-30 09-25-27\LXG-7076A-RAC.D
Sample Name:

```
=====================================================================
Acq. Operator   :                               Seq. Line :  12
Acq. Instrument : Instrument 1                   Location : Vial 62
Injection Date  : 6/30/2020 1:29:23 PM                Inj :   1
                                               Inj Volume : 5.000 µl
Different Inj Volume from Sequence !     Actual Inj Volume : 4.000 µl
Acq. Method     : C:\CHEM32\1\DATA\QDY 2020-06-30 09-25-27\AD-20-30.M
Last changed    : 6/15/2018 10:29:43 AM
Analysis Method : C:\CHEM32\1\METHODS\LXG-IC-10-10.M
Last changed    : 7/4/2020 3:28:28 PM
                  (modified after loading)
Additional Info : Peak(s) manually integrated
```

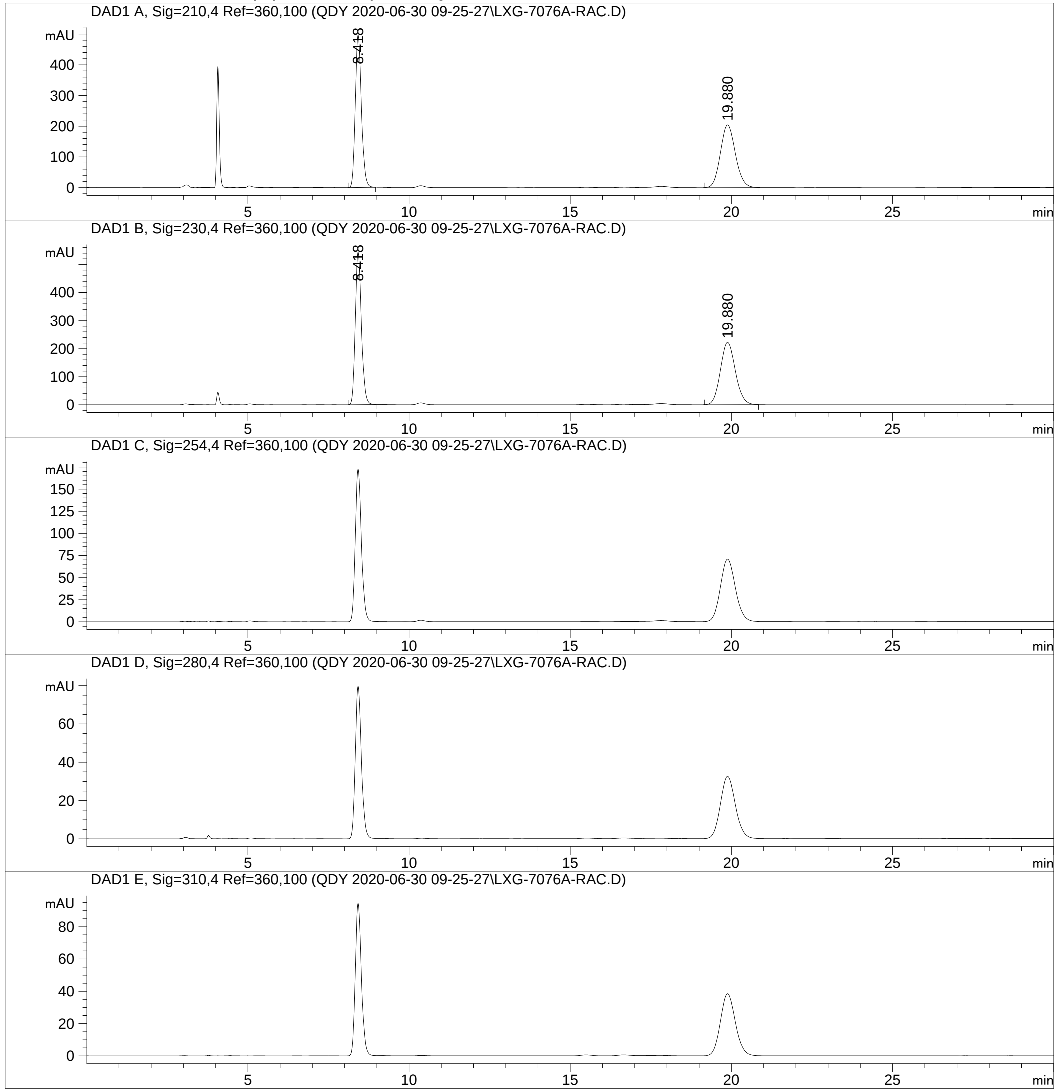

Data File C:\CHEM32\1\DATA\QDY 2020-06-30 09-25-27\LXG-7076A-RAC.D
Sample Name:

Area Percent Report

Sorted By : Signal
Multiplier : 1.0000
Dilution : 1.0000
Use Multiplier & Dilution Factor with ISTDs

Signal 1: DAD1 A, Sig=210,4 Ref=360,100

Peak #	RetTime [min]	Type	Width [min]	Area [mAU*s]	Height [mAU]	Area %
1	8.418	BB	0.2023	6566.19678	496.47937	49.7740
2	19.880	BB	0.5021	6625.81348	204.46458	50.2260

Totals : 1.31920e4 700.94395

Signal 2: DAD1 B, Sig=230,4 Ref=360,100

Peak #	RetTime [min]	Type	Width [min]	Area [mAU*s]	Height [mAU]	Area %
1	8.418	BB	0.2018	7164.24072	543.28973	49.9198
2	19.880	BB	0.5011	7187.27441	222.34601	50.0802

Totals : 1.43515e4 765.63574

Signal 3: DAD1 C, Sig=254,4 Ref=360,100

Signal 4: DAD1 D, Sig=280,4 Ref=360,100

Signal 5: DAD1 E, Sig=310,4 Ref=360,100

*** End of Report ***

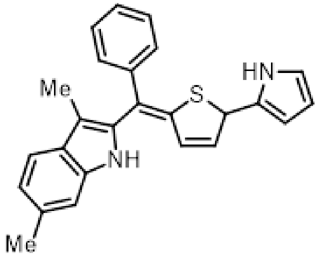

3n
(racemic)

Supplementary Figure 170. HPLC spectrum of **3n** (racemic)

```
=====================================================================
Acq. Operator   :                             Seq. Line :  20
Acq. Instrument : Instrument 1                 Location : Vial 65
Injection Date  : 7/3/2020 6:19:13 PM               Inj :   1
                                             Inj Volume : 5.000 µl
Different Inj Volume from Sequence !     Actual Inj Volume : 4.000 µl
Acq. Method     : C:\CHEM32\1\DATA\QDY 2020-07-03 11-16-20\AD-20-30.M
Last changed    : 6/15/2018 10:29:43 AM
Analysis Method : C:\CHEM32\1\METHODS\LXG-IC-10-10.M
Last changed    : 7/4/2020 3:16:15 PM
                  (modified after loading)
Additional Info : Peak(s) manually integrated
```

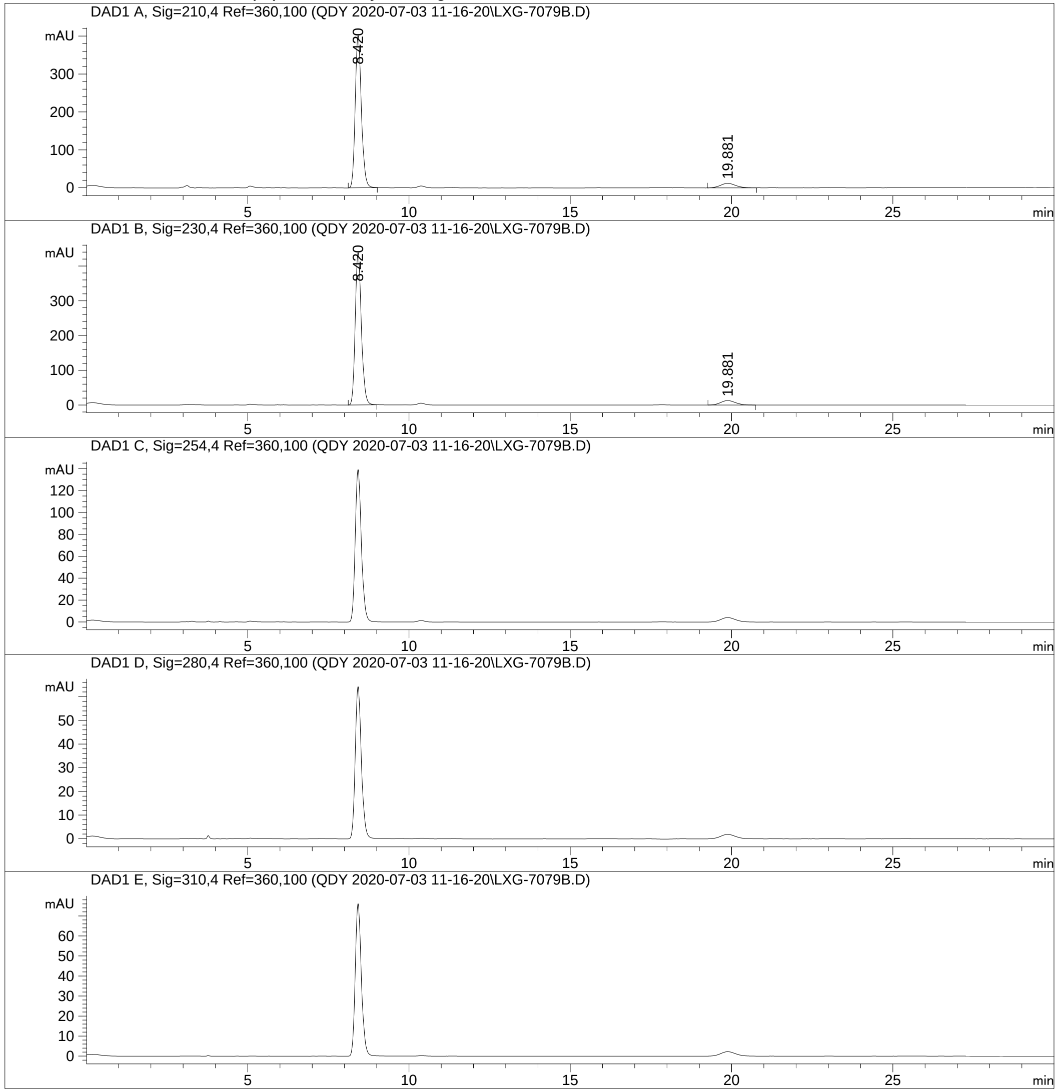

Data File C:\CHEM32\1\DATA\QDY 2020-07-03 11-16-20\LXG-7079B.D
Sample Name:

```
=====================================================================
                         Area Percent Report
=====================================================================

Sorted By             :      Signal
Multiplier            :      1.0000
Dilution              :      1.0000
Use Multiplier & Dilution Factor with ISTDs


Signal 1: DAD1 A, Sig=210,4 Ref=360,100

Peak RetTime Type  Width     Area      Height     Area
  #   [min]        [min]   [mAU*s]     [mAU]        %
----|-------|----|-------|----------|----------|--------|
   1   8.420 BB    0.2025 5321.66992  401.91852  93.1099
   2  19.881 BB    0.4943  393.80081   11.89208   6.8901

Totals :                  5715.47073  413.81060


Signal 2: DAD1 B, Sig=230,4 Ref=360,100

Peak RetTime Type  Width     Area      Height     Area
  #   [min]        [min]   [mAU*s]     [mAU]        %
----|-------|----|-------|----------|----------|--------|
   1   8.420 BB    0.2020 5795.16406  439.01126  93.3268
   2  19.881 BB    0.5043  414.37753   12.84661   6.6732

Totals :                  6209.54160  451.85787


Signal 3: DAD1 C, Sig=254,4 Ref=360,100


Signal 4: DAD1 D, Sig=280,4 Ref=360,100


Signal 5: DAD1 E, Sig=310,4 Ref=360,100

=====================================================================
                         *** End of Report ***
```

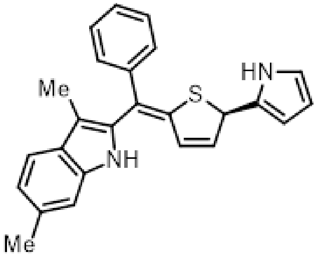

3n
(enantioenriched)

Supplementary Figure 171. HPLC spectrum of **3n** (enantioenriched)

```
Data File C:\CHEM32\1\DATA\QDY 2020-06-28 16-23-58\LXG-7074D-RAC3.D
Sample Name:

    =====================================================================
    Acq. Operator   :                               Seq. Line :   8
    Acq. Instrument : Instrument 1                   Location : Vial 66
    Injection Date  : 6/28/2020 7:43:09 PM                Inj :   1
                                                   Inj Volume : 5.000 µl
    Different Inj Volume from Sequence !     Actual Inj Volume : 4.000 µl
    Acq. Method     : C:\CHEM32\1\DATA\QDY 2020-06-28 16-23-58\AD-20-30.M
    Last changed    : 6/15/2018 10:29:43 AM
    Analysis Method : C:\CHEM32\1\METHODS\LXG-IC-10-10.M
    Last changed    : 7/4/2020 3:11:19 PM
                      (modified after loading)
    Additional Info : Peak(s) manually integrated
```

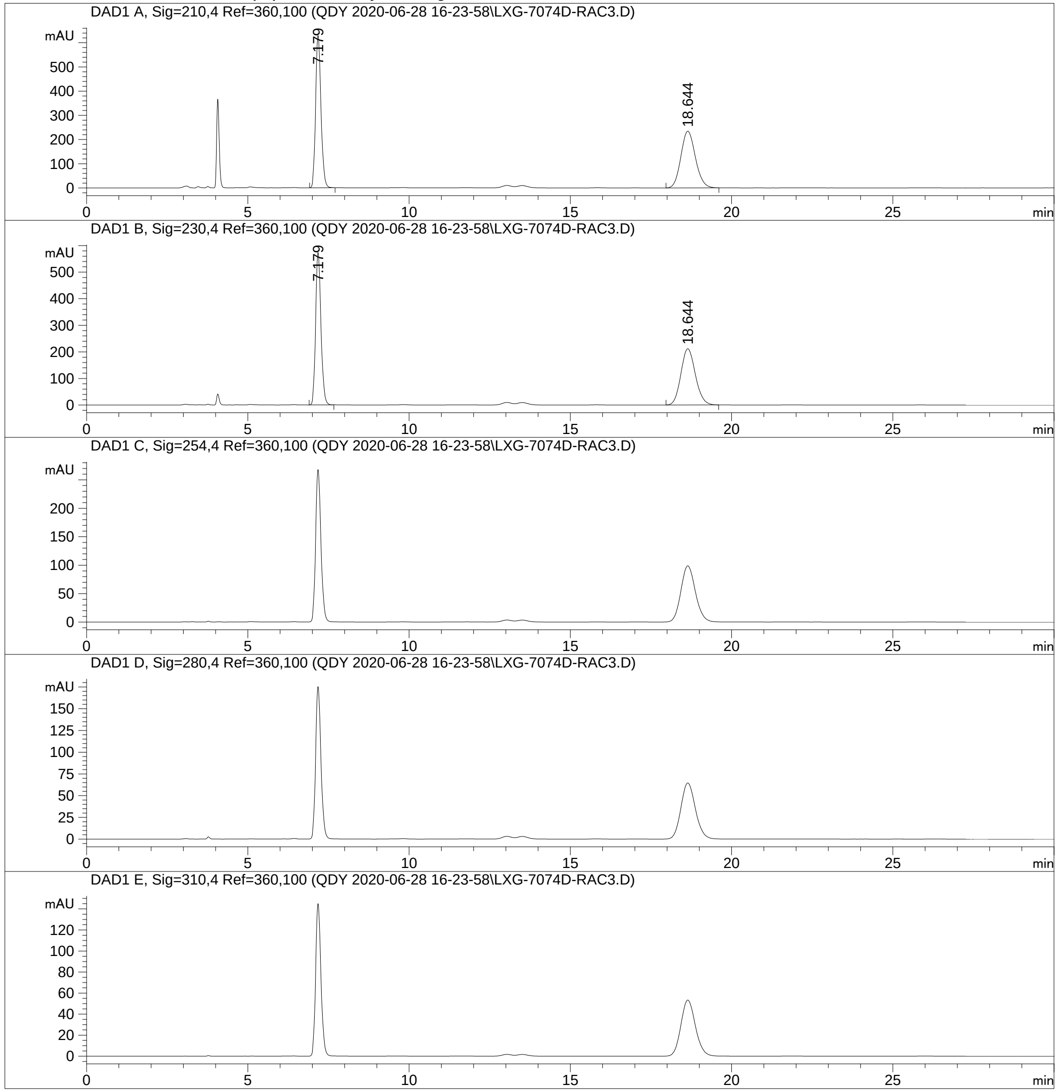


```
Data File C:\CHEM32\1\DATA\QDY 2020-06-28 16-23-58\LXG-7074D-RAC3.D
Sample Name:

    =====================================================================
                             Area Percent Report
    =====================================================================

    Sorted By             :      Signal
    Multiplier            :      1.0000
    Dilution              :      1.0000
    Use Multiplier & Dilution Factor with ISTDs


    Signal 1: DAD1 A, Sig=210,4 Ref=360,100

    Peak RetTime Type  Width     Area      Height     Area
      #   [min]        [min]   [mAU*s]     [mAU]        %
    ----|-------|----|-------|----------|----------|--------|
       1   7.179 BB    0.1786 7264.91260  629.84473  49.7575
       2  18.644 BB    0.4848 7335.71191  234.59599  50.2425

    Totals :                  1.46006e4   864.44072


    Signal 2: DAD1 B, Sig=230,4 Ref=360,100

    Peak RetTime Type  Width     Area      Height     Area
      #   [min]        [min]   [mAU*s]     [mAU]        %
    ----|-------|----|-------|----------|----------|--------|
       1   7.179 BB    0.1779 6584.26807  573.82117  49.9065
       2  18.644 BB    0.4823 6608.92773  211.65363  50.0935

    Totals :                  1.31932e4   785.47479


    Signal 3: DAD1 C, Sig=254,4 Ref=360,100


    Signal 4: DAD1 D, Sig=280,4 Ref=360,100


    Signal 5: DAD1 E, Sig=310,4 Ref=360,100

    =====================================================================
                              *** End of Report ***
```

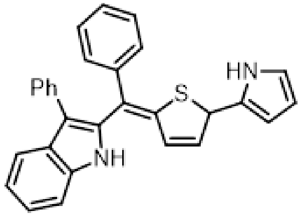

3o
(racemic)

Supplementary Figure 172. HPLC spectrum of **3o** (racemic)

Data File C:\CHEM32\1\DATA\QDY 2020-07-01 14-13-48\LXG-7077C.D
Sample Name:

```
=====================================================================
Acq. Operator   :                               Seq. Line :   5
Acq. Instrument : Instrument 1                   Location : Vial 66
Injection Date  : 7/1/2020 3:20:51 PM                 Inj :   1
                                               Inj Volume : 5.000 µl
Different Inj Volume from Sequence !     Actual Inj Volume : 4.000 µl
Acq. Method     : C:\CHEM32\1\DATA\QDY 2020-07-01 14-13-48\AD-20-30.M
Last changed    : 6/15/2018 10:29:43 AM
Analysis Method : C:\CHEM32\1\METHODS\LXG-IC-10-10.M
Last changed    : 7/4/2020 12:06:36 PM
                  (modified after loading)
Additional Info : Peak(s) manually integrated
```

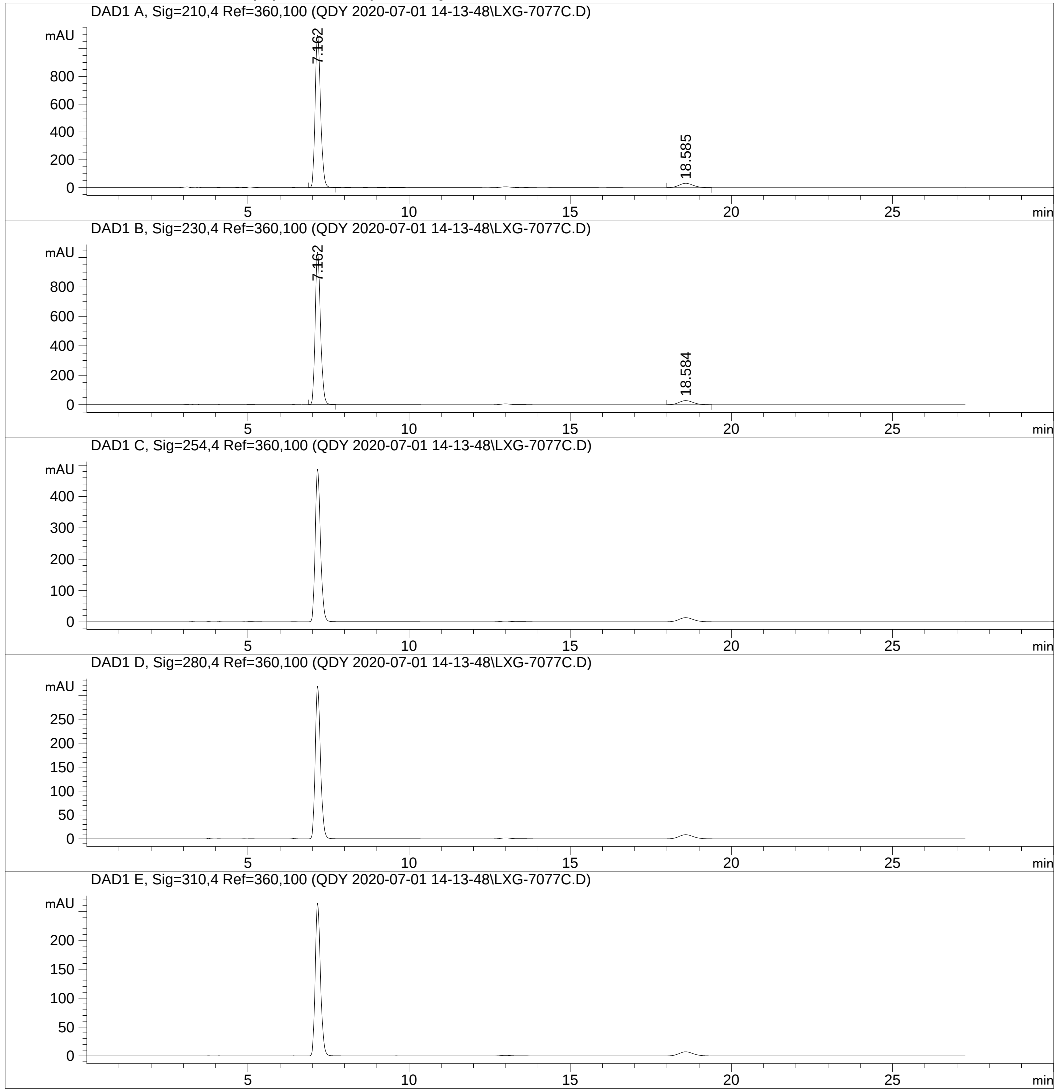

Data File C:\CHEM32\1\DATA\QDY 2020-07-01 14-13-48\LXG-7077C.D
Sample Name:

```
=====================================================================
                        Area Percent Report
=====================================================================

Sorted By             :      Signal
Multiplier            :      1.0000
Dilution              :      1.0000
Use Multiplier & Dilution Factor with ISTDs


Signal 1: DAD1 A, Sig=210,4 Ref=360,100

Peak RetTime Type  Width     Area      Height     Area
  #   [min]        [min]   [mAU*s]     [mAU]        %
----|-------|----|-------|----------|----------|--------|
   1   7.162 BB    0.1820 1.27756e4  1096.18677  92.9054
   2  18.585 BB    0.4792  975.58557   31.50851   7.0946

Totals :                  1.37512e4  1127.69527


Signal 2: DAD1 B, Sig=230,4 Ref=360,100

Peak RetTime Type  Width     Area      Height     Area
  #   [min]        [min]   [mAU*s]     [mAU]        %
----|-------|----|-------|----------|----------|--------|
   1   7.162 BB    0.1750 1.18066e4  1035.32043  93.0994
   2  18.584 BB    0.4802  875.12103   28.34671   6.9006

Totals :                  1.26817e4  1063.66715


Signal 3: DAD1 C, Sig=254,4 Ref=360,100


Signal 4: DAD1 D, Sig=280,4 Ref=360,100


Signal 5: DAD1 E, Sig=310,4 Ref=360,100

=====================================================================
                          *** End of Report ***
```

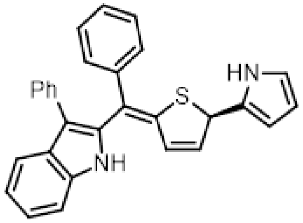

3o
(enantioenriched)

Supplementary Figure 173. HPLC spectrum of **3o** (enantioenriched)

===

Acq. Operator   :   Seq. Line :   3
Acq. Instrument : Instrument 1   Location : Vial 64
Injection Date : 6/30/2020 5:20:12 PM   Inj :   1
                                                Inj Volume : 5.000 µl
Different Inj Volume from Sequence !   Actual Inj Volume : 4.000 µl
Acq. Method   : C:\CHEM32\1\DATA\QDY 2020-06-30 16-56-13\AD-20-30.M
Last changed   : 6/15/2018 10:29:43 AM
Analysis Method : C:\CHEM32\1\METHODS\OD-01-45-0.5.M
Last changed   : 8/22/2020 12:07:56 PM
                  (modified after loading)
Additional Info : Peak(s) manually integrated

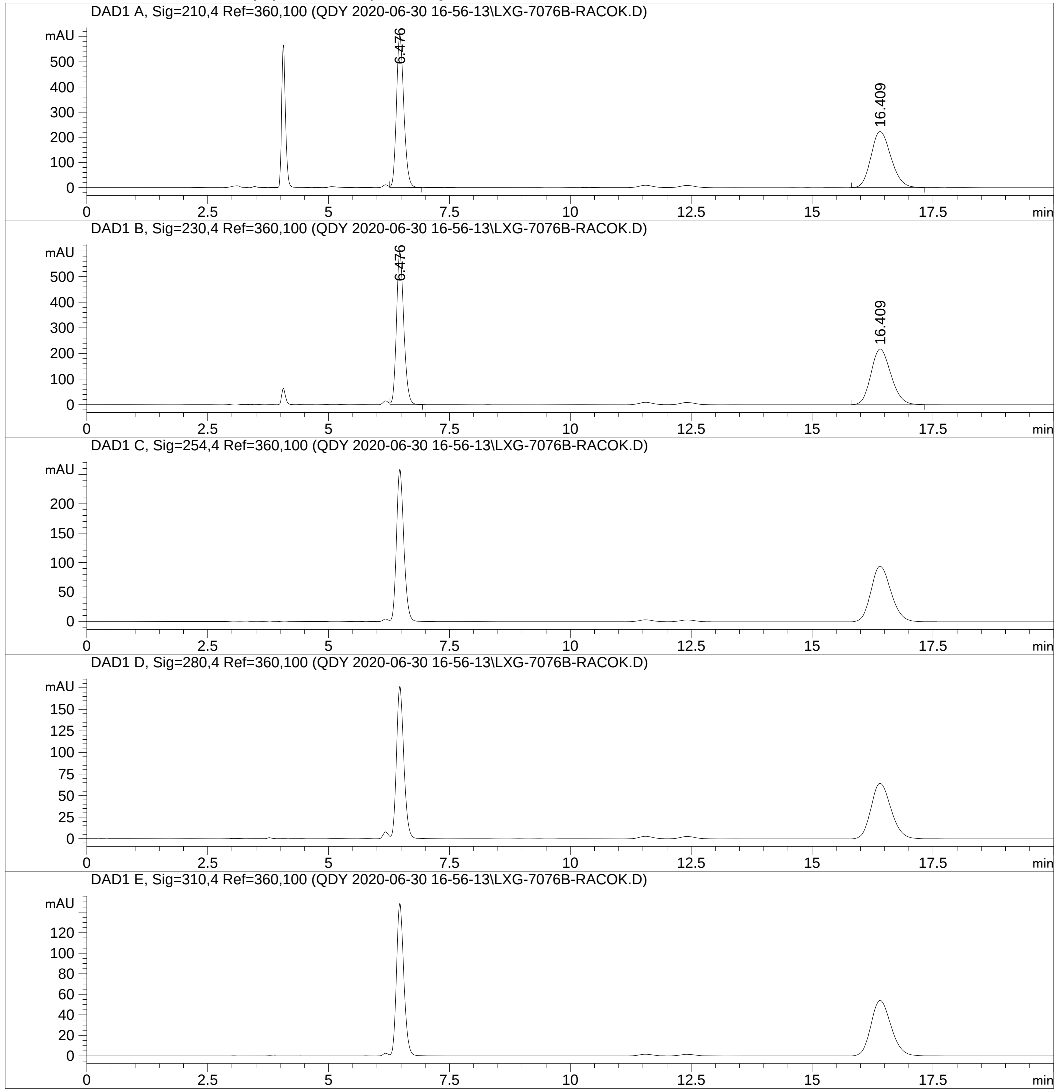

Data File C:\CHEM32\1\DATA\QDY 2020-06-30 16-56-13\LXG-7076B-RACOK.D
Sample Name:

===

Area Percent Report

===

Sorted By   :   Signal
Multiplier   :   1.0000
Dilution   :   1.0000
Use Multiplier & Dilution Factor with ISTDs

Signal 1: DAD1 A, Sig=210,4 Ref=360,100

Peak #	RetTime [min]	Type	Width [min]	Area [mAU*s]	Height [mAU]	Area %
1	6.476	VB	0.1633	6397.48779	605.55389	49.8967
2	16.409	BB	0.4476	6423.98975	223.29907	50.1033

Totals :   1.28215e4   828.85297

Signal 2: DAD1 B, Sig=230,4 Ref=360,100

Peak #	RetTime [min]	Type	Width [min]	Area [mAU*s]	Height [mAU]	Area %
1	6.476	VB	0.1607	6258.13525	595.15112	50.0114
2	16.409	BB	0.4474	6255.27832	217.57716	49.9886

Totals :   1.25134e4   812.72829

Signal 3: DAD1 C, Sig=254,4 Ref=360,100

Signal 4: DAD1 D, Sig=280,4 Ref=360,100

Signal 5: DAD1 E, Sig=310,4 Ref=360,100

===

*** End of Report ***

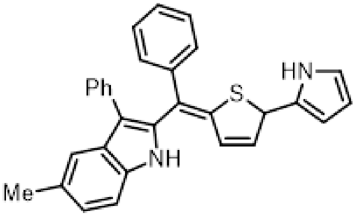

3p
(racemic)

Supplementary Figure 174. HPLC spectrum of **3p** (racemic)

===

Acq. Operator : Seq. Line : 24
Acq. Instrument : Instrument 1 Location : Vial 66
Injection Date : 7/3/2020 7:43:37 PM Inj : 1
Inj Volume : 5.000 µl
Different Inj Volume from Sequence ! Actual Inj Volume : 4.000 µl
Acq. Method : C:\CHEM32\1\DATA\QDY 2020-07-03 11-16-20\AD-20-20.M
Last changed : 7/4/2015 10:27:19 AM
Analysis Method : C:\CHEM32\1\METHODS\LXG-IC-10-10.M
Last changed : 7/4/2020 3:16:15 PM
(modified after loading)
Additional Info : Peak(s) manually integrated

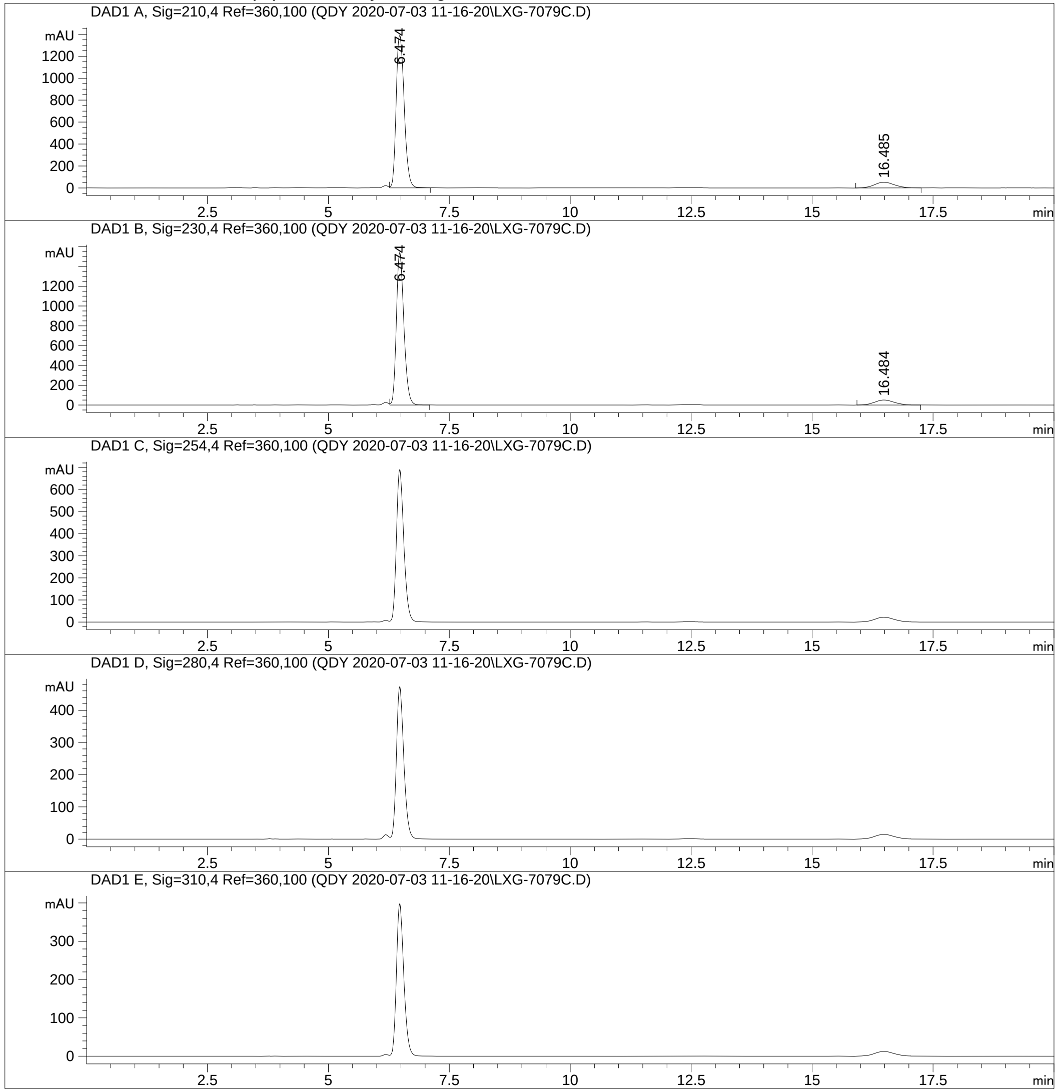

Data File C:\CHEM32\1\DATA\QDY 2020-07-03 11-16-20\LXG-7079C.D
Sample Name:

===

Area Percent Report

===

Sorted By : Signal
Multiplier : 1.0000
Dilution : 1.0000
Use Multiplier & Dilution Factor with ISTDs

Signal 1: DAD1 A, Sig=210,4 Ref=360,100

Peak #	RetTime [min]	Type	Width [min]	Area [mAU*s]	Height [mAU]	Area %
1	6.474	VB	0.1827	1.60315e4	1388.66321	91.4658
2	16.485	BB	0.4434	1495.81299	52.34620	8.5342

Totals : 1.75273e4 1441.00940

Signal 2: DAD1 B, Sig=230,4 Ref=360,100

Peak #	RetTime [min]	Type	Width [min]	Area [mAU*s]	Height [mAU]	Area %
1	6.474	VB	0.1682	1.66535e4	1540.03833	91.9762
2	16.484	BB	0.4432	1452.81128	50.86245	8.0238

Totals : 1.81064e4 1590.90078

Signal 3: DAD1 C, Sig=254,4 Ref=360,100

Signal 4: DAD1 D, Sig=280,4 Ref=360,100

Signal 5: DAD1 E, Sig=310,4 Ref=360,100

===

*** End of Report ***

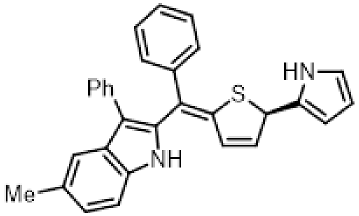

3p
(enantioenriched)

Supplementary Figure 175. HPLC spectrum of **3p** (enantioenriched)

Data File C:\CHEM32\1\DATA\QDY 2020-06-30 16-56-13\LXG-7076C-RACOK.D
Sample Name:

===

Acq. Operator : Seq. Line : 4
Acq. Instrument : Instrument 1 Location : Vial 63
Injection Date : 6/30/2020 5:51:17 PM Inj : 1
Inj Volume : 5.000 µl
Different Inj Volume from Sequence ! Actual Inj Volume : 4.000 µl
Acq. Method : C:\CHEM32\1\DATA\QDY 2020-06-30 16-56-13\AD-20-30.M
Last changed : 6/15/2018 10:29:43 AM
Analysis Method : C:\CHEM32\1\METHODS\LXG-IC-10-10.M
Last changed : 7/4/2020 3:29:47 PM
(modified after loading)
Additional Info : Peak(s) manually integrated

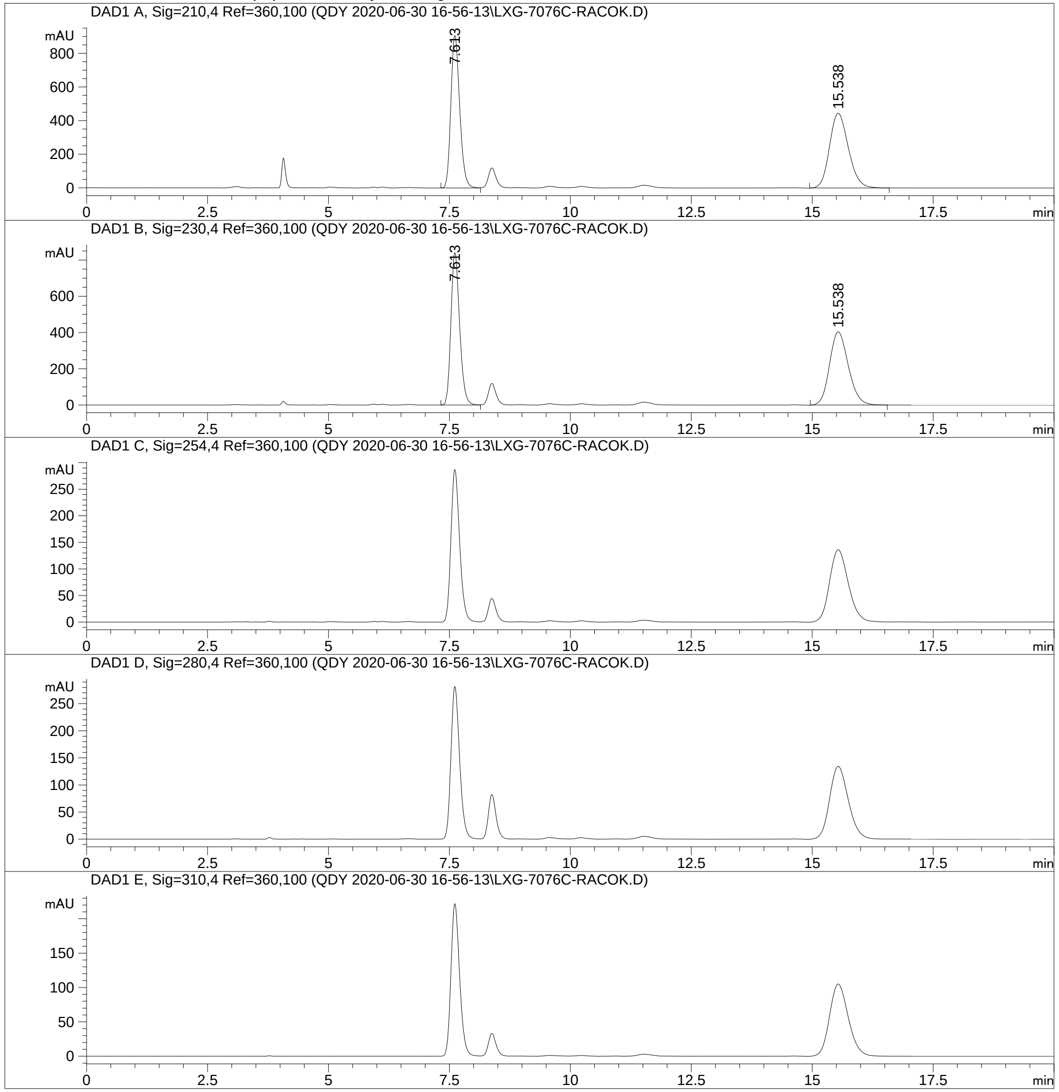

Data File C:\CHEM32\1\DATA\QDY 2020-06-30 16-56-13\LXG-7076C-RACOK.D
Sample Name:

===
Area Percent Report
===

Sorted By : Signal
Multiplier : 1.0000
Dilution : 1.0000
Use Multiplier & Dilution Factor with ISTDs

Signal 1: DAD1 A, Sig=210,4 Ref=360,100

Peak #	RetTime [min]	Type	Width [min]	Area [mAU*s]	Height [mAU]	Area %
1	7.613	BV	0.1960	1.15018e4	906.76239	48.8909
2	15.538	BB	0.4197	1.20237e4	444.39545	51.1091

Totals : 2.35255e4 1351.15784

Signal 2: DAD1 B, Sig=230,4 Ref=360,100

Peak #	RetTime [min]	Type	Width [min]	Area [mAU*s]	Height [mAU]	Area %
1	7.613	BV	0.1921	1.05353e4	841.55798	49.1840
2	15.538	BB	0.4165	1.08849e4	403.81821	50.8160

Totals : 2.14202e4 1245.37619

Signal 3: DAD1 C, Sig=254,4 Ref=360,100

Signal 4: DAD1 D, Sig=280,4 Ref=360,100

Signal 5: DAD1 E, Sig=310,4 Ref=360,100

===
*** End of Report ***

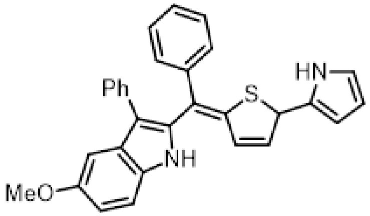

3q
(racemic)

Supplementary Figure 176. HPLC spectrum of **3q** (racemic)

===

Acq. Operator : Seq. Line : 35
Acq. Instrument : Instrument 1 Location : Vial 67
Injection Date : 7/3/2020 11:45:25 PM Inj : 1
Inj Volume : 5.000 µl
Different Inj Volume from Sequence ! Actual Inj Volume : 4.000 µl
Acq. Method : C:\CHEM32\1\DATA\QDY 2020-07-03 11-16-20\AD-20-20.M
Last changed : 7/4/2015 10:27:19 AM
Analysis Method : C:\CHEM32\1\METHODS\OD-01-45-0.5.M
Last changed : 8/20/2020 7:42:57 PM
(modified after loading)
Additional Info : Peak(s) manually integrated

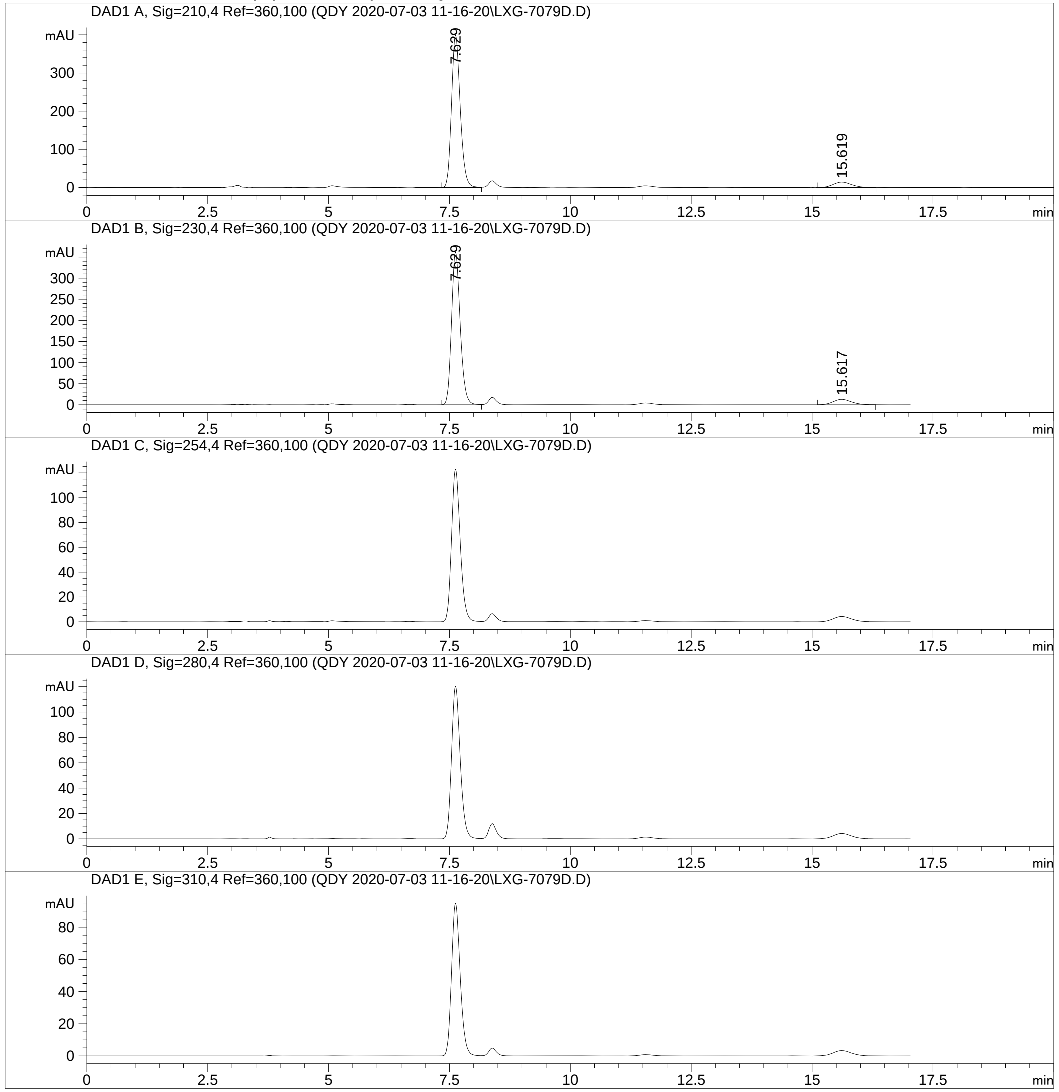

Data File C:\CHEM32\1\DATA\QDY 2020-07-03 11-16-20\LXG-7079D.D
Sample Name:

===
Area Percent Report
===

Sorted By : Signal
Multiplier : 1.0000
Dilution : 1.0000
Use Multiplier & Dilution Factor with ISTDs

Signal 1: DAD1 A, Sig=210,4 Ref=360,100

Peak #	RetTime [min]	Type	Width [min]	Area [mAU*s]	Height [mAU]	Area %
1	7.629	BB	0.1923	5002.79199	398.98288	92.9864
2	15.619	BB	0.4132	377.34076	14.23963	7.0136

Totals : 5380.13275 413.22251

Signal 2: DAD1 B, Sig=230,4 Ref=360,100

Peak #	RetTime [min]	Type	Width [min]	Area [mAU*s]	Height [mAU]	Area %
1	7.629	BB	0.1919	4517.89209	361.29172	92.9591
2	15.617	BB	0.4122	342.19412	12.86958	7.0409

Totals : 4860.08621 374.16129

Signal 3: DAD1 C, Sig=254,4 Ref=360,100

Signal 4: DAD1 D, Sig=280,4 Ref=360,100

Signal 5: DAD1 E, Sig=310,4 Ref=360,100

===
*** End of Report ***

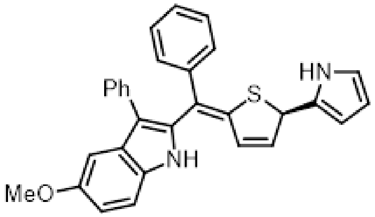

3q
(enantioenriched)

Supplementary Figure 177. HPLC spectrum of **3q** (enantioenriched)

Data File C:\CHEM32\1\DATA\QDY 2020-07-23 21-52-40\LXG-7078CC-RAC1.D
Sample Name:

===

Acq. Operator :	Seq. Line : 2
Acq. Instrument : Instrument 1	Location : Vial 61
Injection Date : 7/23/2020 10:25:53 PM	Inj : 1
	Inj Volume : 5.000 µl

Different Inj Volume from Sequence ! Actual Inj Volume : 4.000 µl
Acq. Method : C:\CHEM32\1\DATA\QDY 2020-07-23 21-52-40\AD-20-30.M
Last changed : 6/15/2018 10:29:43 AM
Analysis Method : C:\CHEM32\1\METHODS\AD-008-50.M
Last changed : 7/25/2020 7:11:23 PM
(modified after loading)
Additional Info : Peak(s) manually integrated

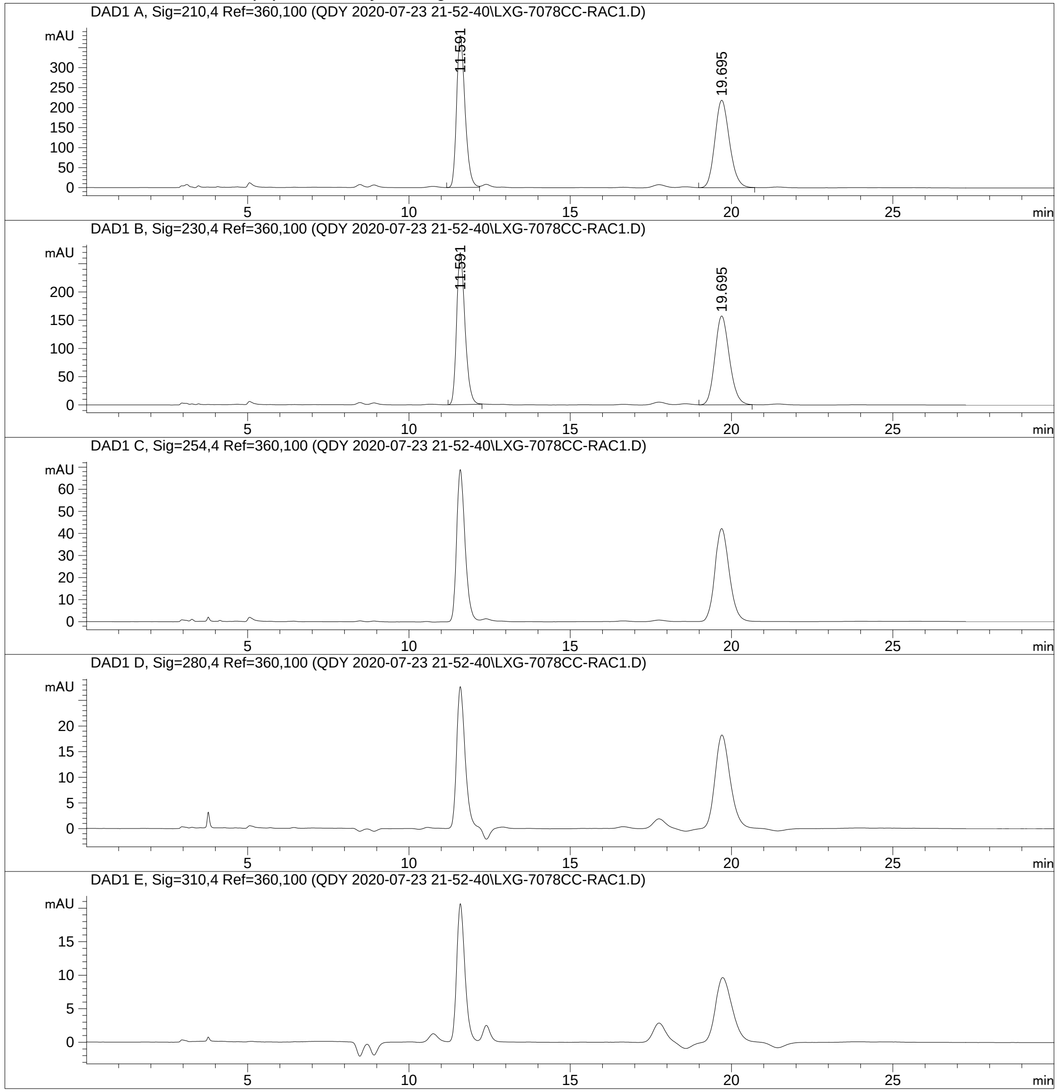

Data File C:\CHEM32\1\DATA\QDY 2020-07-23 21-52-40\LXG-7078CC-RAC1.D
Sample Name:

===
Area Percent Report
===

Sorted By : Signal
Multiplier : 1.0000
Dilution : 1.0000
Use Multiplier & Dilution Factor with ISTDs

Signal 1: DAD1 A, Sig=210,4 Ref=360,100

Peak #	RetTime [min]	Type	Width [min]	Area [mAU*s]	Height [mAU]	Area %
1	11.591	VV	0.2783	6912.13135	379.90875	50.1463
2	19.695	VB	0.4846	6871.79346	218.69592	49.8537

Totals : 1.37839e4 598.60468

Signal 2: DAD1 B, Sig=230,4 Ref=360,100

Peak #	RetTime [min]	Type	Width [min]	Area [mAU*s]	Height [mAU]	Area %
1	11.591	BB	0.2774	4877.55859	269.19696	49.7728
2	19.695	VB	0.4827	4922.08057	157.45436	50.2272

Totals : 9799.63916 426.65132

Signal 3: DAD1 C, Sig=254,4 Ref=360,100

Signal 4: DAD1 D, Sig=280,4 Ref=360,100

Signal 5: DAD1 E, Sig=310,4 Ref=360,100

===
*** End of Report ***

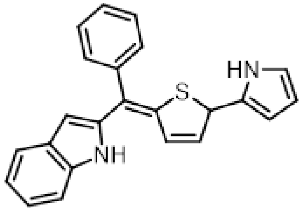

3r
(racemic)

Supplementary Figure 178. HPLC spectrum of **3r** (racemic)

Data File C:\CHEM32\1\DATA\QDY 2020-07-23 21-52-40\LXG-7096B.D
Sample Name:

===

Acq. Operator   :   Seq. Line :   1
Acq. Instrument : Instrument 1   Location : Vial 62
Injection Date : 7/23/2020 9:54:29 PM   Inj :   1
                                              Inj Volume : 5.000 µl
Different Inj Volume from Sequence !   Actual Inj Volume : 4.000 µl
Acq. Method   : C:\CHEM32\1\DATA\QDY 2020-07-23 21-52-40\AD-20-30.M
Last changed   : 6/15/2018 10:29:43 AM
Analysis Method : C:\CHEM32\1\METHODS\AD-008-50.M
Last changed   : 7/25/2020 7:11:23 PM
                  (modified after loading)
Additional Info : Peak(s) manually integrated

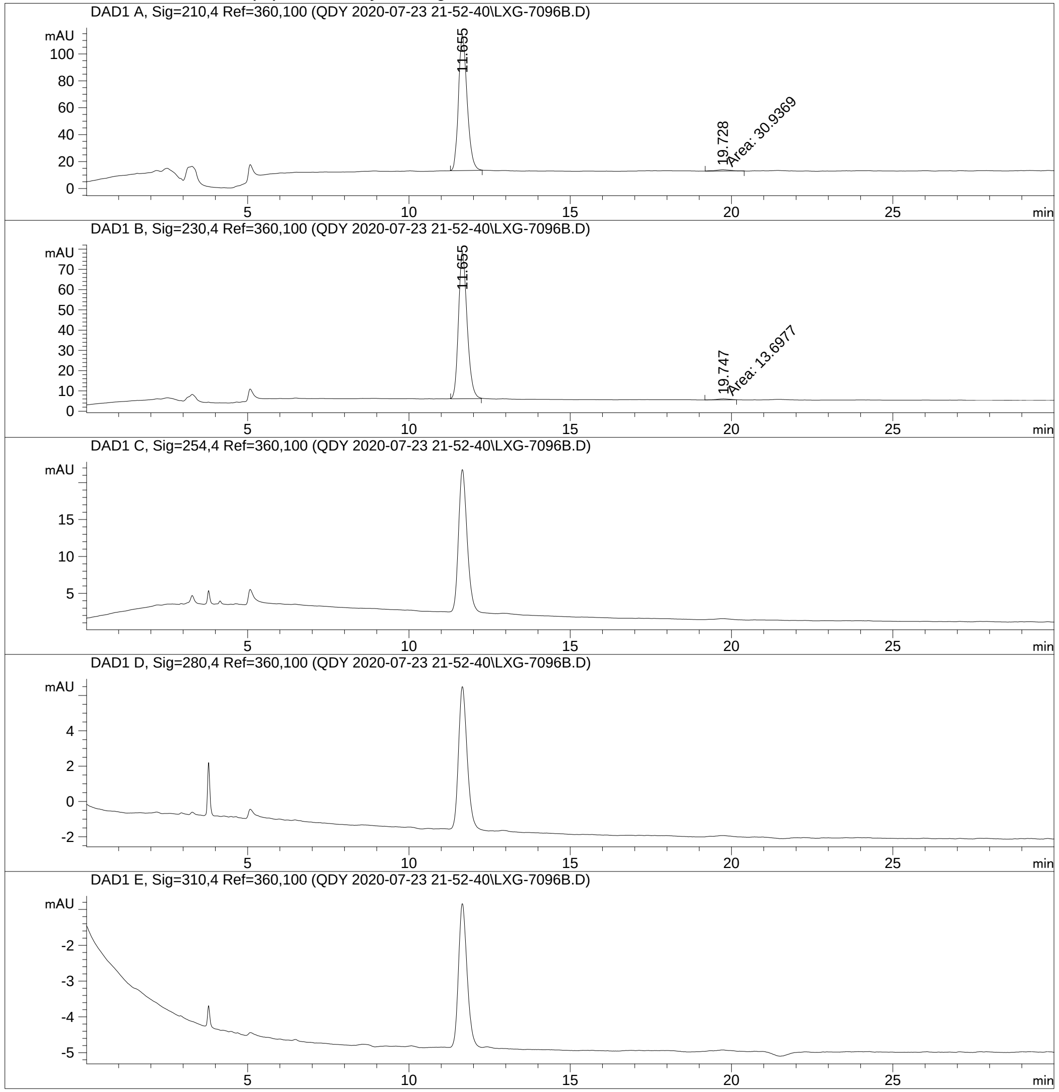

Data File C:\CHEM32\1\DATA\QDY 2020-07-23 21-52-40\LXG-7096B.D
Sample Name:

===

Area Percent Report

===

Sorted By : Signal
Multiplier : 1.0000
Dilution : 1.0000
Use Multiplier & Dilution Factor with ISTDs

Signal 1: DAD1 A, Sig=210,4 Ref=360,100

Peak #	RetTime [min]	Type	Width [min]	Area [mAU*s]	Height [mAU]	Area %
1	11.655	BB	0.2849	1867.79529	100.47170	98.3707
2	19.728	MM	0.5511	30.93695	9.35695e-1	1.6293

Totals : 1898.73224 101.40740

Signal 2: DAD1 B, Sig=230,4 Ref=360,100

Peak #	RetTime [min]	Type	Width [min]	Area [mAU*s]	Height [mAU]	Area %
1	11.655	BB	0.2847	1341.24048	72.21307	98.9891
2	19.747	MM	0.4835	13.69767	4.72175e-1	1.0109

Totals : 1354.93815 72.68524

Signal 3: DAD1 C, Sig=254,4 Ref=360,100

Signal 4: DAD1 D, Sig=280,4 Ref=360,100

Signal 5: DAD1 E, Sig=310,4 Ref=360,100

===

*** End of Report ***

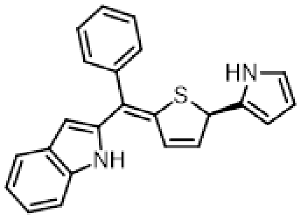

3r
(enantioenriched)

Supplementary Figure 179. HPLC spectrum of **3r** (enantioenriched)

```
=====================================================================
Acq. Operator   :                              Seq. Line :   8
Acq. Instrument : Instrument 1                  Location : Vial 64
Injection Date  : 6/24/2020 4:52:15 PM               Inj :   1
                                              Inj Volume : 5.000 µl
Different Inj Volume from Sequence !     Actual Inj Volume : 4.000 µl
Acq. Method     : C:\CHEM32\1\DATA\QDY 2020-06-24 13-47-56\OD-10-40.M
Last changed    : 6/24/2020 3:29:18 PM
                  (modified after loading)
Analysis Method : C:\CHEM32\1\METHODS\LXG-IC-10-10.M
Last changed    : 7/4/2020 12:06:36 PM
                  (modified after loading)
Additional Info : Peak(s) manually integrated
```

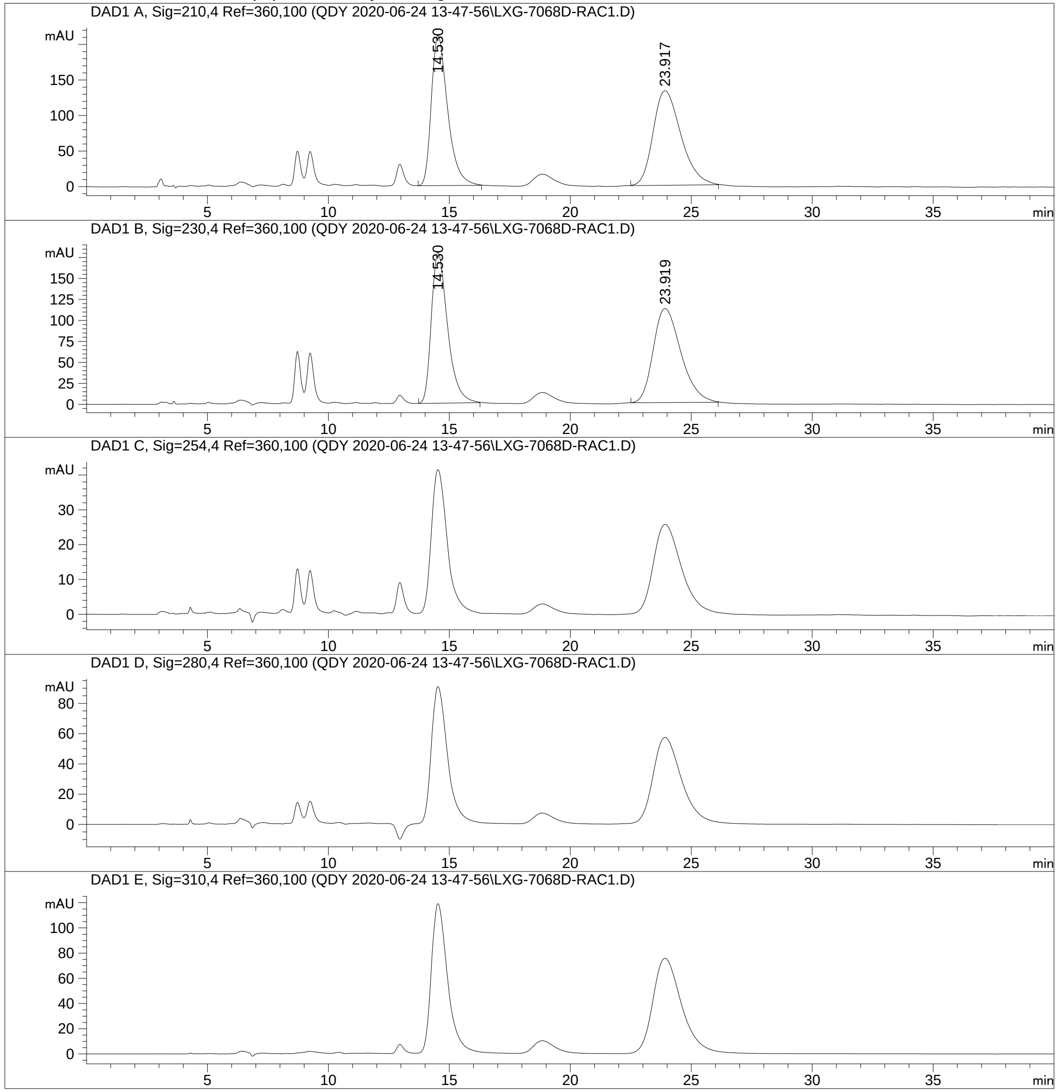

Data File C:\CHEM32\1\DATA\QDY 2020-06-24 13-47-56\LXG-7068D-RAC1.D
Sample Name:

```
=====================================================================
                        Area Percent Report
=====================================================================

Sorted By             :      Signal
Multiplier            :      1.0000
Dilution              :      1.0000
Use Multiplier & Dilution Factor with ISTDs


Signal 1: DAD1 A, Sig=210,4 Ref=360,100

Peak RetTime Type  Width     Area      Height     Area
  #   [min]        [min]   [mAU*s]     [mAU]        %
----|-------|----|-------|----------|----------|--------|
   1  14.530 BB    0.7216 9914.00586  211.21516  48.1994
   2  23.917 BB    1.2231 1.06547e4   132.93356  51.8006

Totals :                  2.05687e4   344.14873


Signal 2: DAD1 B, Sig=230,4 Ref=360,100

Peak RetTime Type  Width     Area      Height     Area
  #   [min]        [min]   [mAU*s]     [mAU]        %
----|-------|----|-------|----------|----------|--------|
   1  14.530 BB    0.7203 8385.44629  179.72000  48.3618
   2  23.919 BB    1.2266 8953.55469  112.24313  51.6382

Totals :                  1.73390e4   291.96313


Signal 3: DAD1 C, Sig=254,4 Ref=360,100


Signal 4: DAD1 D, Sig=280,4 Ref=360,100


Signal 5: DAD1 E, Sig=310,4 Ref=360,100

=====================================================================
                        *** End of Report ***
```

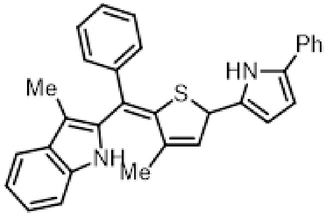

3s
(racemic)

Supplementary Figure 180. HPLC spectrum of **3s** (racemic)

```
=====================================================================
Acq. Operator   :                              Seq. Line :  11
Acq. Instrument : Instrument 1                  Location : Vial 68
Injection Date  : 6/28/2020 8:56:48 PM               Inj :   1
                                              Inj Volume : 5.000 µl
Different Inj Volume from Sequence !     Actual Inj Volume : 4.000 µl
Acq. Method     : C:\CHEM32\1\DATA\QDY 2020-06-28 16-23-58\OD-10-40.M
Last changed    : 6/28/2020 8:55:55 PM
                  (modified after loading)
Analysis Method : C:\CHEM32\1\METHODS\LXG-IC-10-10.M
Last changed    : 7/4/2020 12:06:36 PM
                  (modified after loading)
Additional Info : Peak(s) manually integrated
```

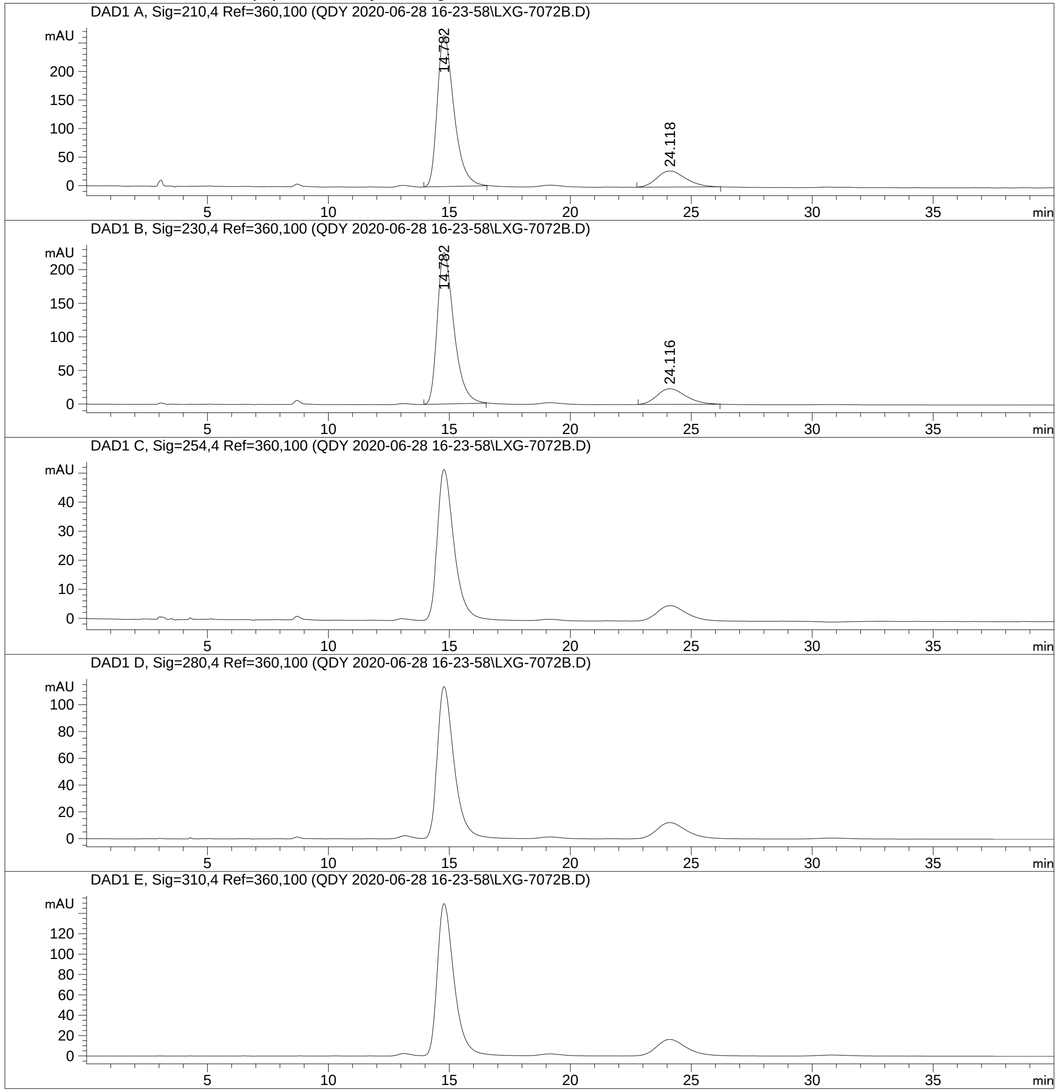

Data File C:\CHEM32\1\DATA\QDY 2020-06-28 16-23-58\LXG-7072B.D
Sample Name:

```
=====================================================================
                         Area Percent Report
=====================================================================

Sorted By             :      Signal
Multiplier            :      1.0000
Dilution              :      1.0000
Use Multiplier & Dilution Factor with ISTDs
```

Signal 1: DAD1 A, Sig=210,4 Ref=360,100

Peak #	RetTime [min]	Type	Width [min]	Area [mAU*s]	Height [mAU]	Area %
1	14.782	BB	0.7299	1.26581e4	264.72620	84.3879
2	24.118	BB	1.1321	2341.79321	28.10609	15.6121

Totals : 1.49999e4 292.83229

Signal 2: DAD1 B, Sig=230,4 Ref=360,100

Peak #	RetTime [min]	Type	Width [min]	Area [mAU*s]	Height [mAU]	Area %
1	14.782	BB	0.7264	1.07576e4	225.56746	84.8413
2	24.116	BB	1.2257	1922.07410	23.22079	15.1587

Totals : 1.26797e4 248.78825

Signal 3: DAD1 C, Sig=254,4 Ref=360,100

Signal 4: DAD1 D, Sig=280,4 Ref=360,100

Signal 5: DAD1 E, Sig=310,4 Ref=360,100

===
*** End of Report ***

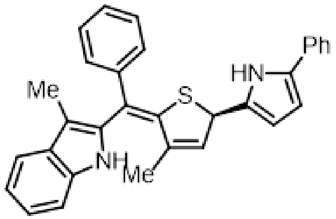

3s
(enantioenriched)

Supplementary Figure 181. HPLC spectrum of **3s** (enantioenriched)

===

Acq. Operator   :   Seq. Line :   6
Acq. Instrument : Instrument 1   Location : Vial 63
Injection Date : 7/24/2020 12:40:20 PM   Inj :   1
                                                Inj Volume : 5.000 µl
Different Inj Volume from Sequence !   Actual Inj Volume : 4.000 µl
Acq. Method   : C:\CHEM32\1\DATA\QDY 2020-07-24 10-42-46\AD-20-30.M
Last changed   : 6/15/2018 10:29:43 AM
Analysis Method : C:\CHEM32\1\METHODS\AD-008-50.M
Last changed   : 7/10/2020 9:21:13 AM
Additional Info : Peak(s) manually integrated

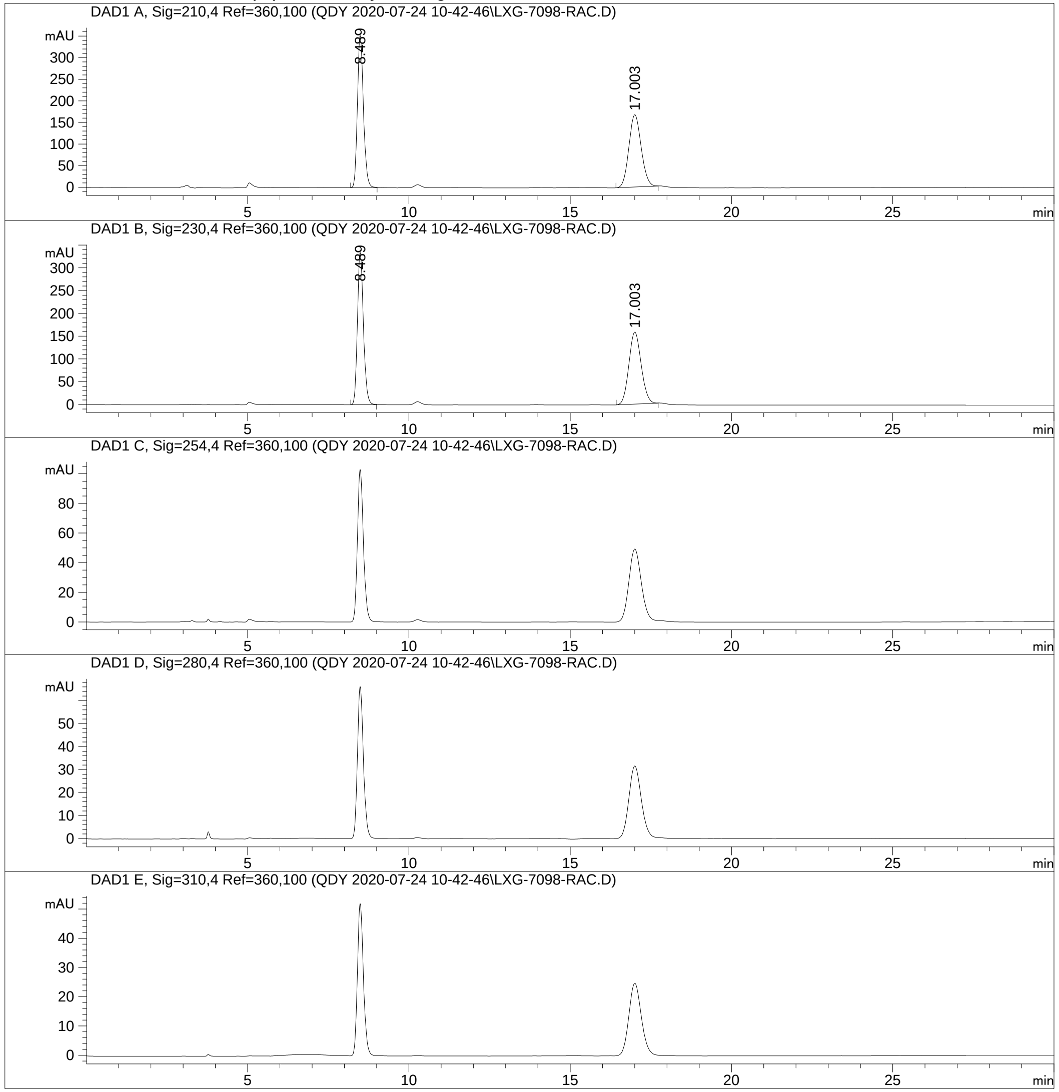

Data File C:\CHEM32\1\DATA\QDY 2020-07-24 10-42-46\LXG-7098-RAC.D
Sample Name:

===

Area Percent Report

===

Sorted By   :   Signal
Multiplier   :   1.0000
Dilution   :   1.0000
Use Multiplier & Dilution Factor with ISTDs

Signal 1: DAD1 A, Sig=210,4 Ref=360,100

Peak #	RetTime [min]	Type	Width [min]	Area [mAU*s]	Height [mAU]	Area %
1	8.489	BB	0.1986	4548.04297	352.30460	50.6533
2	17.003	BB	0.4129	4430.71875	167.31779	49.3467

Totals :   8978.76172 519.62239

Signal 2: DAD1 B, Sig=230,4 Ref=360,100

Peak #	RetTime [min]	Type	Width [min]	Area [mAU*s]	Height [mAU]	Area %
1	8.489	BB	0.1983	4307.11865	334.25439	50.7287
2	17.003	BB	0.4106	4183.38037	158.15608	49.2713

Totals :   8490.49902 492.41048

Signal 3: DAD1 C, Sig=254,4 Ref=360,100

Signal 4: DAD1 D, Sig=280,4 Ref=360,100

Signal 5: DAD1 E, Sig=310,4 Ref=360,100

===

*** End of Report ***

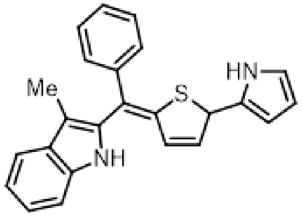

3t
(racemic)

Supplementary Figure 182. HPLC spectrum of **3t** (racemic)

```
=====================================================================
Acq. Operator   :                               Seq. Line :   2
Acq. Instrument : Instrument 1                   Location : Vial 68
Injection Date  : 7/26/2020 7:35:19 PM                Inj :   1
                                               Inj Volume : 5.000 µl
Different Inj Volume from Sequence !     Actual Inj Volume : 4.000 µl
Acq. Method     : C:\CHEM32\1\DATA\QDY 2020-07-26 19-20-01\AD-20-30.M
Last changed    : 6/15/2018 10:29:43 AM
Analysis Method : C:\CHEM32\1\METHODS\AD-008-50.M
Last changed    : 7/10/2020 9:21:13 AM
Additional Info : Peak(s) manually integrated
```

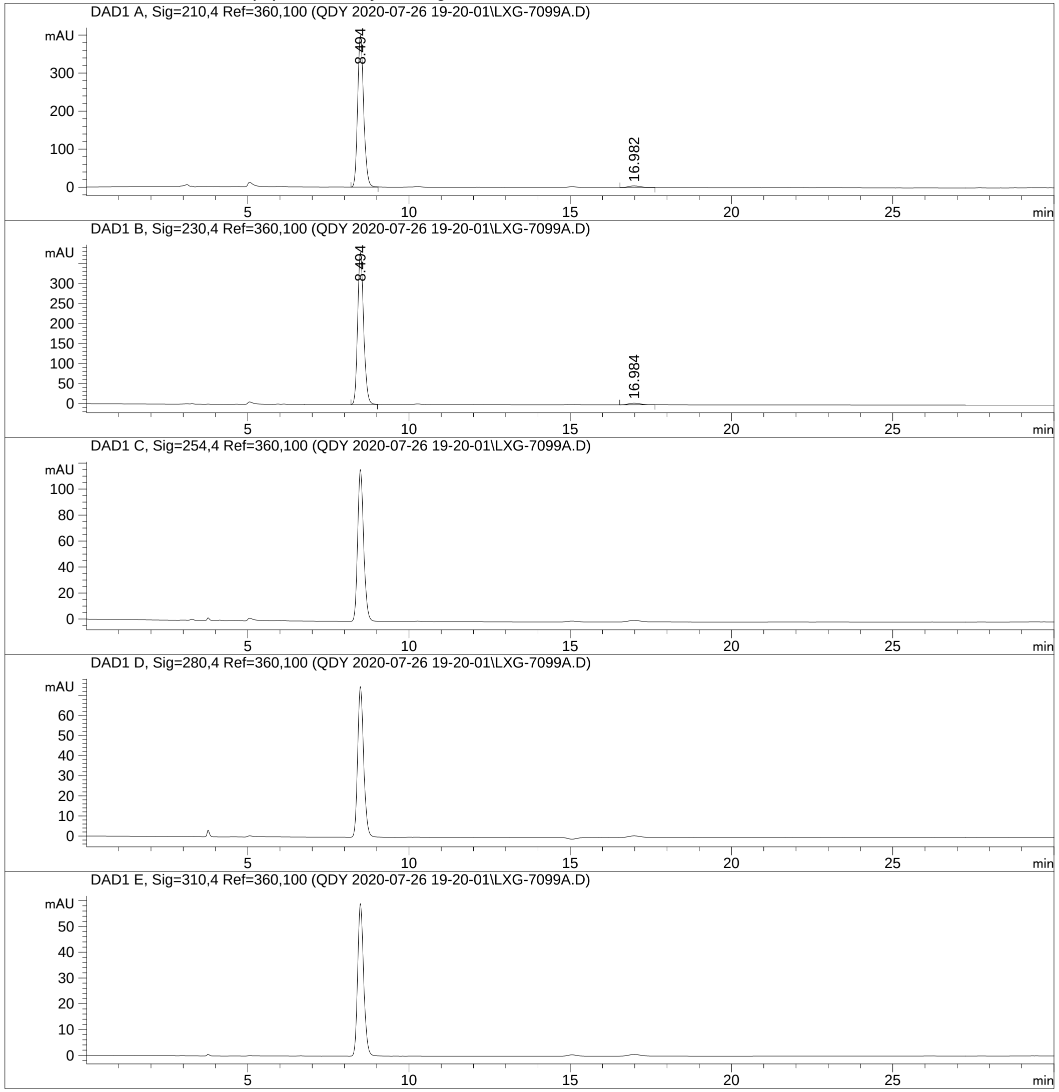

Data File C:\CHEM32\1\DATA\QDY 2020-07-26 19-20-01\LXG-7099A.D
Sample Name:

```
=====================================================================
                          Area Percent Report
=====================================================================

Sorted By             :      Signal
Multiplier            :      1.0000
Dilution              :      1.0000
Use Multiplier & Dilution Factor with ISTDs
```

Signal 1: DAD1 A, Sig=210,4 Ref=360,100

Peak #	RetTime [min]	Type	Width [min]	Area [mAU*s]	Height [mAU]	Area %
1	8.494	BB	0.1991	5162.85303	398.64743	98.0465
2	16.982	BB	0.3791	102.86679	4.20987	1.9535

Totals : 5265.71982 402.85730

Signal 2: DAD1 B, Sig=230,4 Ref=360,100

Peak #	RetTime [min]	Type	Width [min]	Area [mAU*s]	Height [mAU]	Area %
1	8.494	BB	0.1986	4893.23730	379.18848	97.9982
2	16.984	BB	0.3858	99.95373	4.02419	2.0018

Totals : 4993.19103 383.21267

Signal 3: DAD1 C, Sig=254,4 Ref=360,100

Signal 4: DAD1 D, Sig=280,4 Ref=360,100

Signal 5: DAD1 E, Sig=310,4 Ref=360,100

```
=====================================================================
                         *** End of Report ***
```

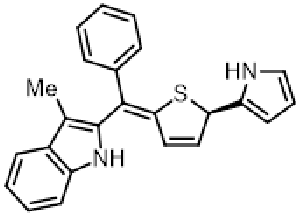

3t
(enantioenriched)

Supplementary Figure 183. HPLC spectrum of **3t** (enantioenriched)

===

Acq. Operator	:	Seq. Line :	4
Acq. Instrument	: Instrument 1	Location :	Vial 63
Injection Date	: 7/25/2019 11:56:33 PM	Inj :	1
		Inj Volume :	5.000 µl

Acq. Method : C:\CHEM32\1\DATA\QDY 2019-07-25 22-20-24\OD-20-40.M
Last changed : 7/25/2019 10:32:57 PM
(modified after loading)
Analysis Method : C:\CHEM32\1\METHODS\OD-001-10-0.7.M
Last changed : 8/23/2019 7:32:17 PM
(modified after loading)
Additional Info : Peak(s) manually integrated

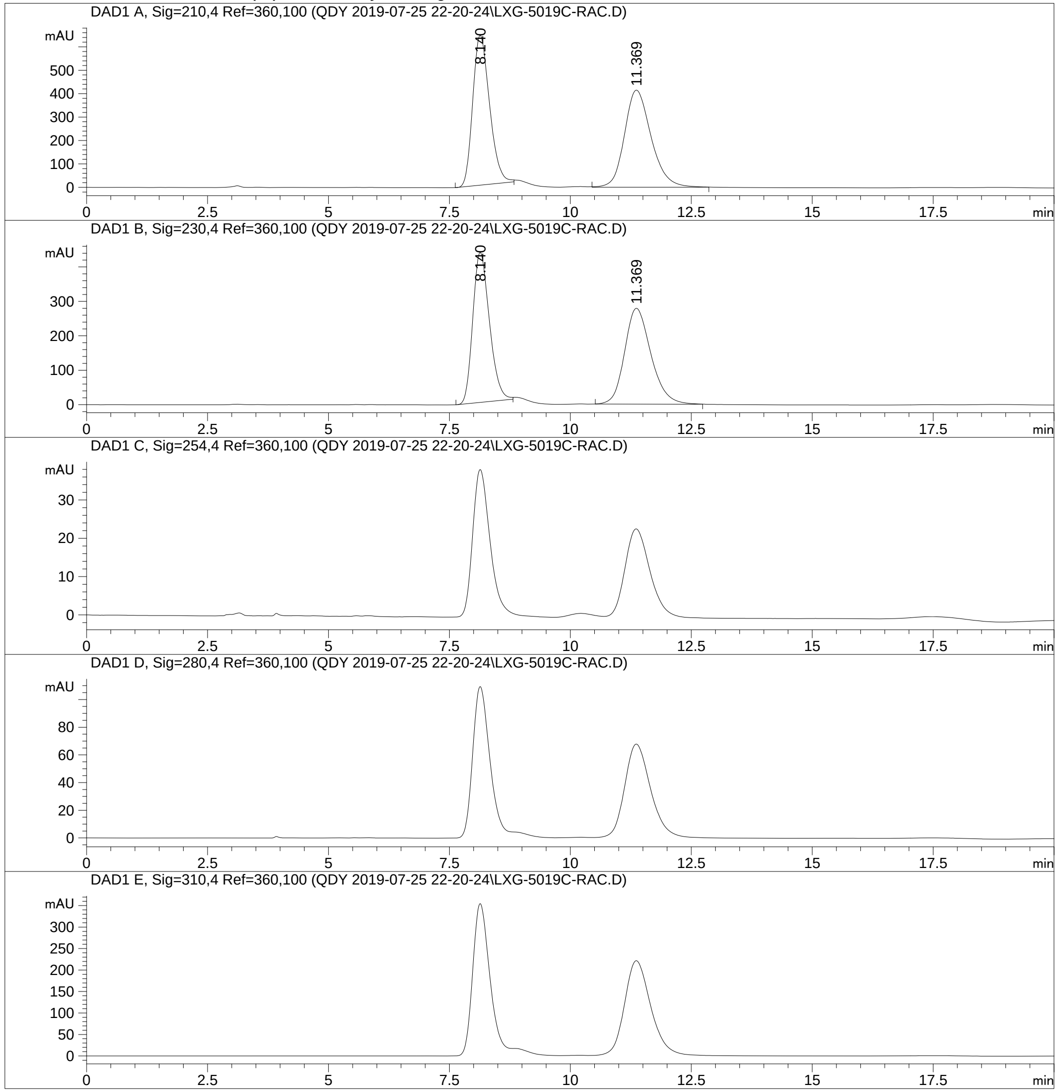

Data File C:\CHEM32\1\DATA\QDY 2019-07-25 22-20-24\LXG-5019C-RAC.D
Sample Name:

===
Area Percent Report
===

Sorted By : Signal
Multiplier : 1.0000
Dilution : 1.0000
Use Multiplier & Dilution Factor with ISTDs

Signal 1: DAD1 A, Sig=210,4 Ref=360,100

Peak #	RetTime [min]	Type	Width [min]	Area [mAU*s]	Height [mAU]	Area %
1	8.140	BB	0.3739	1.54492e4	639.37677	49.3760
2	11.369	VB	0.5859	1.58397e4	414.41437	50.6240

Totals : 3.12888e4 1053.79114

Signal 2: DAD1 B, Sig=230,4 Ref=360,100

Peak #	RetTime [min]	Type	Width [min]	Area [mAU*s]	Height [mAU]	Area %
1	8.140	BB	0.3713	1.04251e4	435.41559	49.7444
2	11.369	BB	0.5796	1.05322e4	278.27737	50.2556

Totals : 2.09573e4 713.69296

Signal 3: DAD1 C, Sig=254,4 Ref=360,100

Signal 4: DAD1 D, Sig=280,4 Ref=360,100

Signal 5: DAD1 E, Sig=310,4 Ref=360,100

===
*** End of Report ***

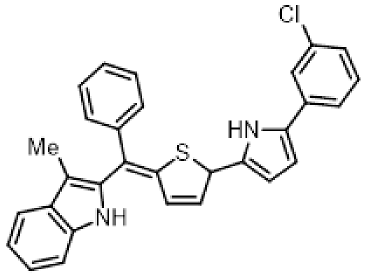

3u
(racemic)

Supplementary Figure 184. HPLC spectrum of **3u** (racemic)

Data File C:\CHEM32\1\DATA\QDY 2019-08-23 12-44-31\LXG-5073A.D
Sample Name:

===

Acq. Operator : Seq. Line : 11
Acq. Instrument : Instrument 1 Location : Vial 65
Injection Date : 8/23/2019 4:59:31 PM Inj : 1
Inj Volume : 5.000 µl
Acq. Method : C:\CHEM32\1\DATA\QDY 2019-08-23 12-44-31\OD-20-20.M
Last changed : 8/23/2019 4:58:39 PM
(modified after loading)
Analysis Method : C:\CHEM32\1\METHODS\OD-001-10-0.7.M
Last changed : 8/21/2019 8:26:51 PM
Additional Info : Peak(s) manually integrated

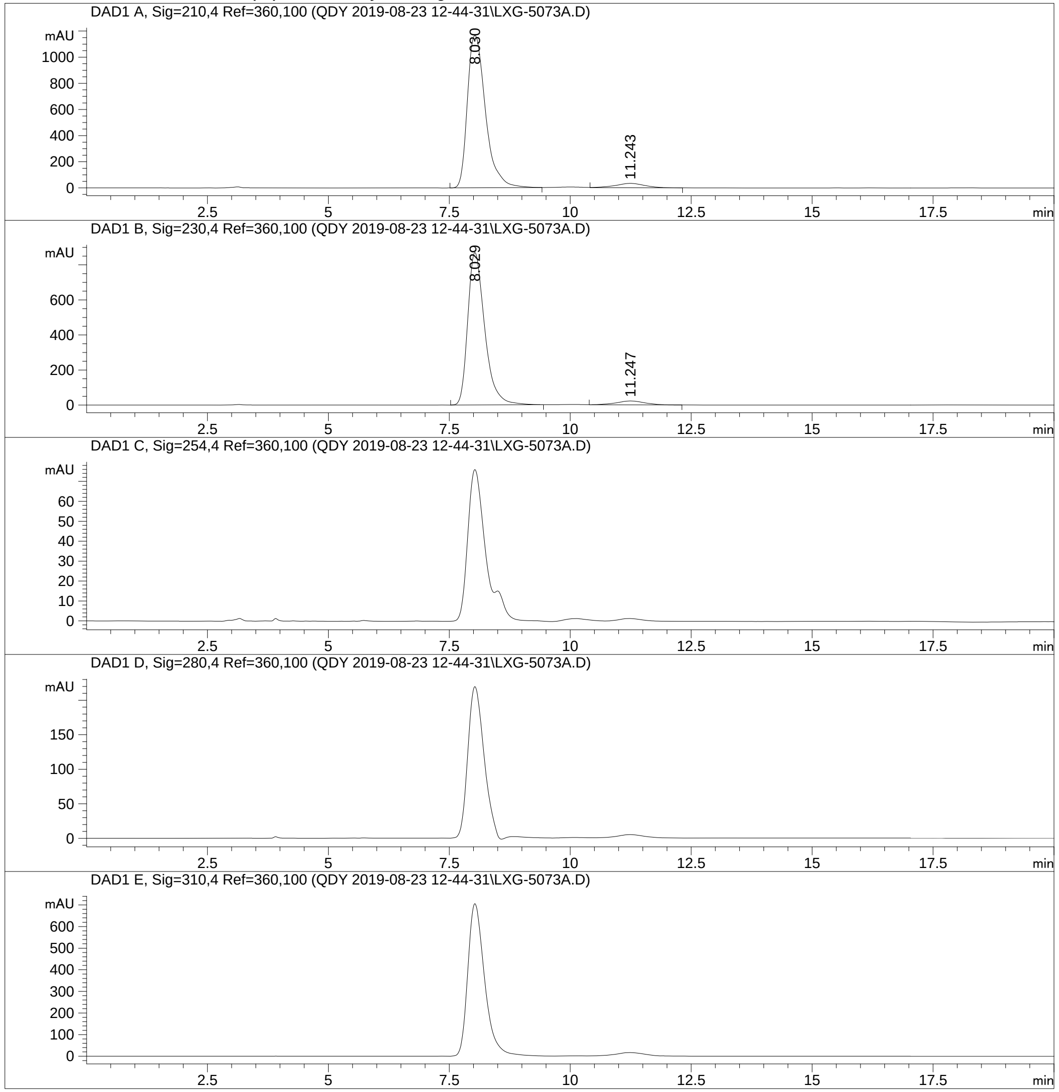

Data File C:\CHEM32\1\DATA\QDY 2019-08-23 12-44-31\LXG-5073A.D
Sample Name:

===

Area Percent Report

===

Sorted By : Signal
Multiplier : 1.0000
Dilution : 1.0000
Use Multiplier & Dilution Factor with ISTDs

Signal 1: DAD1 A, Sig=210,4 Ref=360,100

Peak #	RetTime [min]	Type	Width [min]	Area [mAU*s]	Height [mAU]	Area %
1	8.030	BB	0.4014	3.03079e4	1165.40076	95.5616
2	11.243	VB	0.6249	1407.65125	33.45370	4.4384

Totals : 3.17156e4 1198.85445

Signal 2: DAD1 B, Sig=230,4 Ref=360,100

Peak #	RetTime [min]	Type	Width [min]	Area [mAU*s]	Height [mAU]	Area %
1	8.029	BB	0.3727	2.12207e4	869.62476	95.7986
2	11.247	VB	0.6219	930.66052	22.34653	4.2014

Totals : 2.21514e4 891.97128

Signal 3: DAD1 C, Sig=254,4 Ref=360,100

Signal 4: DAD1 D, Sig=280,4 Ref=360,100

Signal 5: DAD1 E, Sig=310,4 Ref=360,100

===

*** End of Report ***

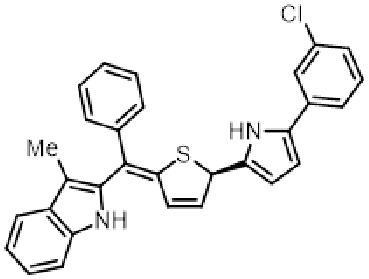

3u
(enantioenriched)

Supplementary Figure 185. HPLC spectrum of **3u** (enantioenriched)

```
=====================================================================
Acq. Operator   :                               Seq. Line :   2
Acq. Instrument : Instrument 1                   Location : Vial 69
Injection Date  : 6/27/2020 1:10:37 PM                Inj :   1
                                               Inj Volume : 5.000 µl
Different Inj Volume from Sequence !     Actual Inj Volume : 4.000 µl
Acq. Method     : C:\CHEM32\1\DATA\QDY 2020-06-27 12-57-40\AD-20-30.M
Last changed    : 6/15/2018 10:29:43 AM
Analysis Method : C:\CHEM32\1\METHODS\AD-008-50.M
Last changed    : 7/25/2020 7:09:18 PM
                  (modified after loading)
Additional Info : Peak(s) manually integrated
```

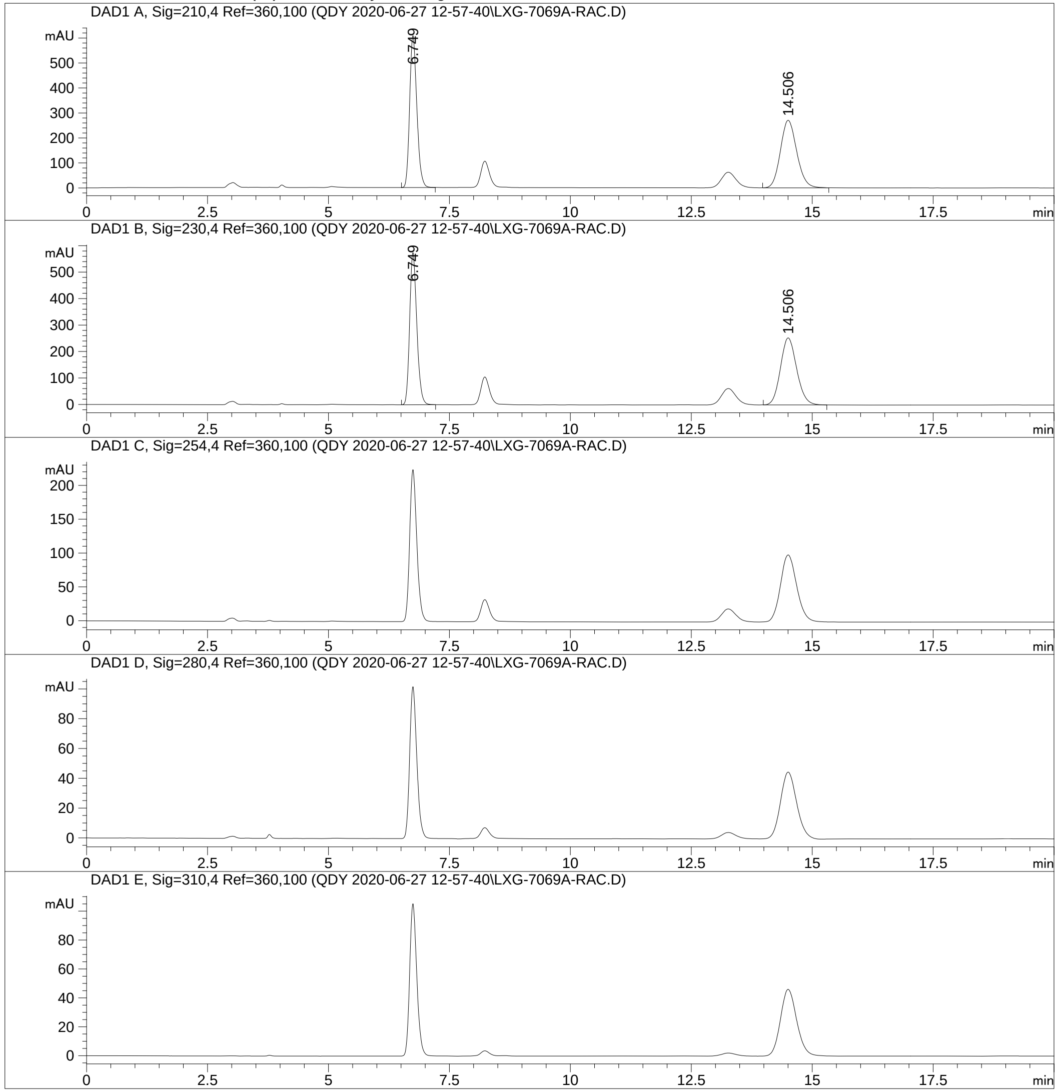

Data File C:\CHEM32\1\DATA\QDY 2020-06-27 12-57-40\LXG-7069A-RAC.D
Sample Name:

```
=====================================================================
                         Area Percent Report
=====================================================================

Sorted By             :      Signal
Multiplier            :      1.0000
Dilution              :      1.0000
Use Multiplier & Dilution Factor with ISTDs
```

Signal 1: DAD1 A, Sig=210,4 Ref=360,100

Peak #	RetTime [min]	Type	Width [min]	Area [mAU*s]	Height [mAU]	Area %
1	6.749	BB	0.1583	6273.87695	608.65845	49.7314
2	14.506	BB	0.3617	6341.64795	270.30951	50.2686

Totals : 1.26155e4 878.96796

Signal 2: DAD1 B, Sig=230,4 Ref=360,100

Peak #	RetTime [min]	Type	Width [min]	Area [mAU*s]	Height [mAU]	Area %
1	6.749	BB	0.1577	5902.71289	575.31378	49.9236
2	14.506	BB	0.3607	5920.78662	253.34920	50.0764

Totals : 1.18235e4 828.66298

Signal 3: DAD1 C, Sig=254,4 Ref=360,100

Signal 4: DAD1 D, Sig=280,4 Ref=360,100

Signal 5: DAD1 E, Sig=310,4 Ref=360,100

===
*** End of Report ***

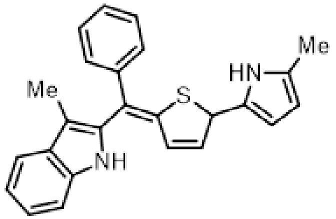

3v
(racemic)

Supplementary Figure 186. HPLC spectrum of **3v** (racemic)

Data File C:\CHEM32\1\DATA\QDY 2020-07-21 15-09-37\LXG-7094A.D
Sample Name:

```
=====================================================================
Acq. Operator   :                               Seq. Line :  10
Acq. Instrument : Instrument 1                   Location : Vial 64
Injection Date  : 7/21/2020 8:02:04 PM                Inj :   1
                                               Inj Volume : 5.000 µl
Different Inj Volume from Sequence !     Actual Inj Volume : 4.000 µl
Acq. Method     : C:\CHEM32\1\DATA\QDY 2020-07-21 15-09-37\AD-20-20.M
Last changed    : 7/21/2020 8:01:12 PM
                  (modified after loading)
Analysis Method : C:\CHEM32\1\METHODS\AD-008-50.M
Last changed    : 7/25/2020 7:05:21 PM
                  (modified after loading)
Additional Info : Peak(s) manually integrated
```

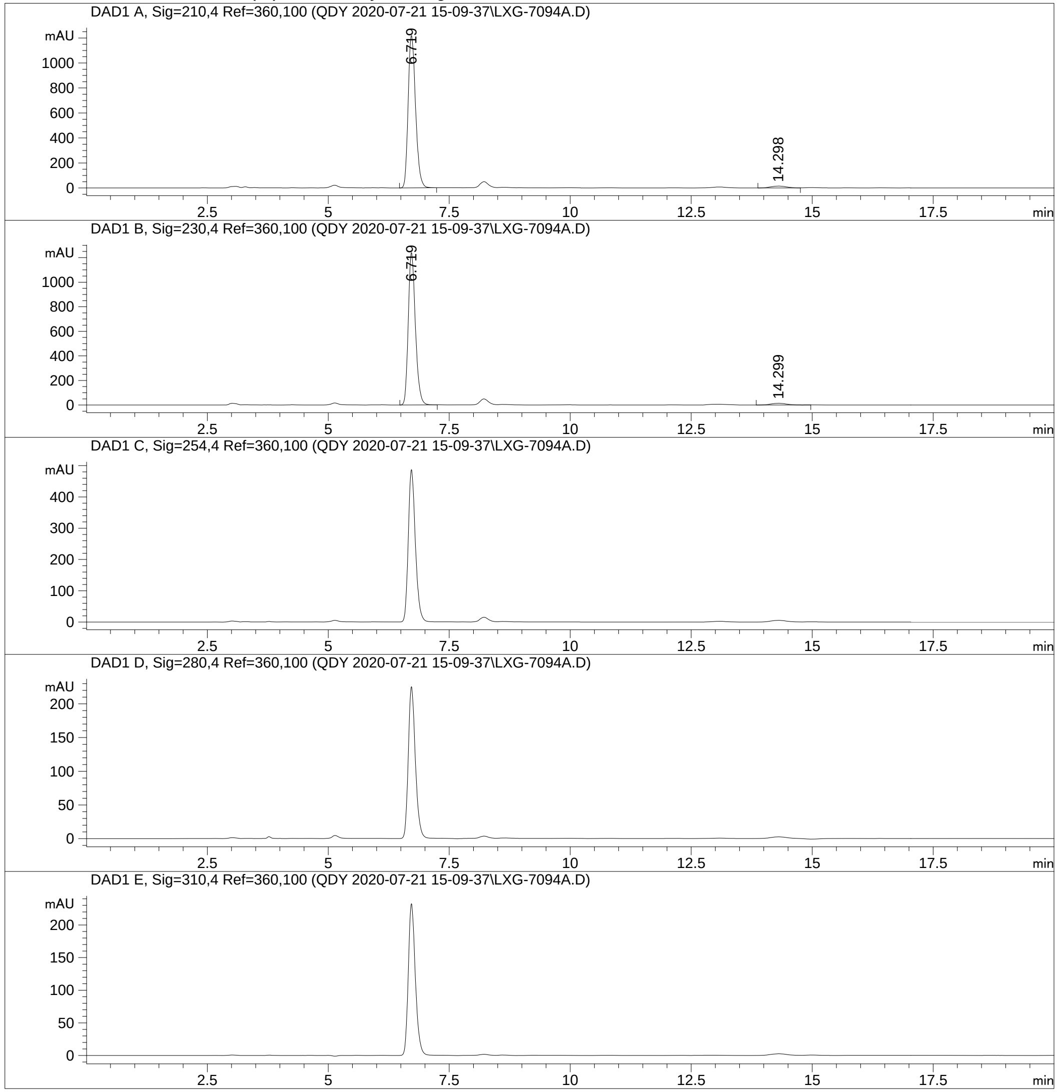

Data File C:\CHEM32\1\DATA\QDY 2020-07-21 15-09-37\LXG-7094A.D
Sample Name:

```
=====================================================================
                          Area Percent Report
=====================================================================

Sorted By             :      Signal
Multiplier            :      1.0000
Dilution              :      1.0000
Use Multiplier & Dilution Factor with ISTDs
```

Signal 1: DAD1 A, Sig=210,4 Ref=360,100

Peak #	RetTime [min]	Type	Width [min]	Area [mAU*s]	Height [mAU]	Area %
1	6.719	BB	0.1663	1.29935e4	1219.68762	97.4356
2	14.298	BV	0.3488	341.97498	15.18033	2.5644

Totals : 1.33354e4 1234.86795

Signal 2: DAD1 B, Sig=230,4 Ref=360,100

Peak #	RetTime [min]	Type	Width [min]	Area [mAU*s]	Height [mAU]	Area %
1	6.719	BB	0.1574	1.27013e4	1241.25940	97.5656
2	14.299	BB	0.3497	316.91843	14.02218	2.4344

Totals : 1.30182e4 1255.28158

Signal 3: DAD1 C, Sig=254,4 Ref=360,100

Signal 4: DAD1 D, Sig=280,4 Ref=360,100

Signal 5: DAD1 E, Sig=310,4 Ref=360,100

===
*** End of Report ***

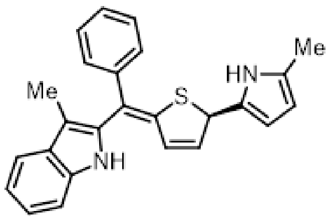

3v
(enantioenriched)

Supplementary Figure 187. HPLC spectrum of **3v** (enantioenriched)

```
=====================================================================
Acq. Operator   :                               Seq. Line :  13
Acq. Instrument : Instrument 1                   Location : Vial 70
Injection Date  : 6/28/2020 9:49:01 PM                Inj :   1
                                               Inj Volume : 5.000 µl
Different Inj Volume from Sequence !     Actual Inj Volume : 4.000 µl
Acq. Method     : C:\CHEM32\1\DATA\QDY 2020-06-28 16-23-58\AD-12-40.M
Last changed    : 11/15/2017 2:34:57 PM
Analysis Method : C:\CHEM32\1\METHODS\AD-008-50.M
Last changed    : 7/25/2020 7:11:23 PM
                  (modified after loading)
Additional Info : Peak(s) manually integrated
```

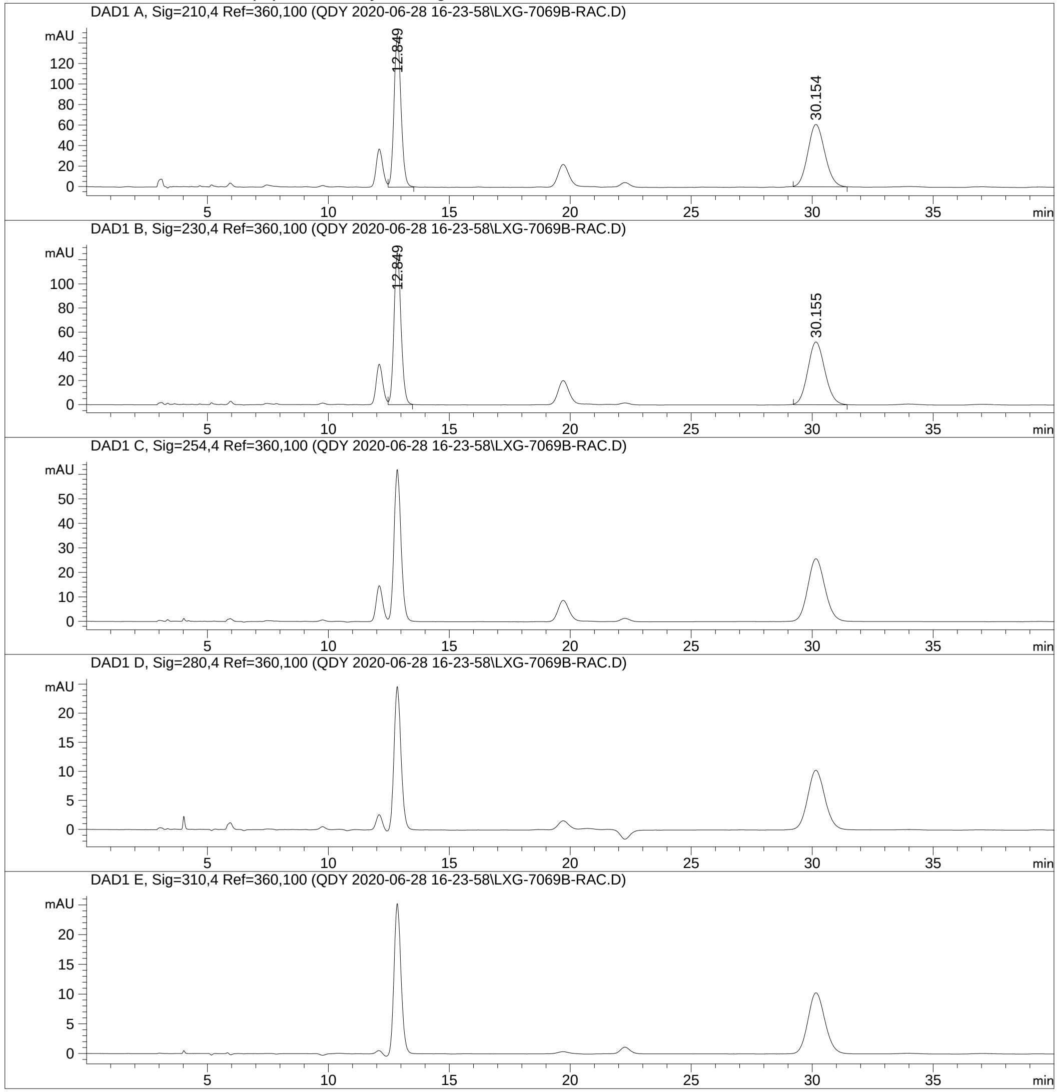

Data File C:\CHEM32\1\DATA\QDY 2020-06-28 16-23-58\LXG-7069B-RAC.D
Sample Name:

```
=====================================================================
                          Area Percent Report
=====================================================================

Sorted By             :      Signal
Multiplier            :      1.0000
Dilution              :      1.0000
Use Multiplier & Dilution Factor with ISTDs


Signal 1: DAD1 A, Sig=210,4 Ref=360,100

Peak RetTime Type  Width     Area      Height     Area
  #   [min]        [min]   [mAU*s]     [mAU]        %
----|-------|----|-------|----------|----------|--------|
   1  12.849 VB    0.3125 2997.79565  147.90913  50.1303
   2  30.154 BB    0.7560 2982.20581   60.82712  49.8697

Totals :                  5980.00146  208.73625


Signal 2: DAD1 B, Sig=230,4 Ref=360,100

Peak RetTime Type  Width     Area      Height     Area
  #   [min]        [min]   [mAU*s]     [mAU]        %
----|-------|----|-------|----------|----------|--------|
   1  12.849 VB    0.3113 2541.47949  125.98605  49.9009
   2  30.155 BB    0.7592 2551.57446   51.93589  50.0991

Totals :                  5093.05396  177.92194


Signal 3: DAD1 C, Sig=254,4 Ref=360,100


Signal 4: DAD1 D, Sig=280,4 Ref=360,100


Signal 5: DAD1 E, Sig=310,4 Ref=360,100

=====================================================================
                          *** End of Report ***
```

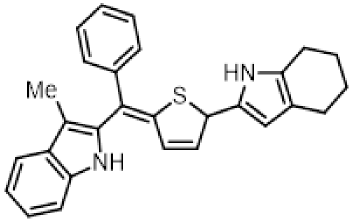

3w
(racemic)

Supplementary Figure 188. HPLC spectrum of **3w** (racemic)

```
=====================================================================
Acq. Operator   :                               Seq. Line :  19
Acq. Instrument : Instrument 1                   Location : Vial 65
Injection Date  : 7/21/2020 11:02:44 PM               Inj :   1
                                               Inj Volume : 5.000 µl
Different Inj Volume from Sequence !     Actual Inj Volume : 3.000 µl
Acq. Method     : C:\CHEM32\1\DATA\QDY 2020-07-21 15-09-37\AD-12-40.M
Last changed    : 11/15/2017 2:34:57 PM
Analysis Method : C:\CHEM32\1\METHODS\AD-008-50.M
Last changed    : 7/25/2020 7:05:21 PM
                  (modified after loading)
Additional Info : Peak(s) manually integrated
```

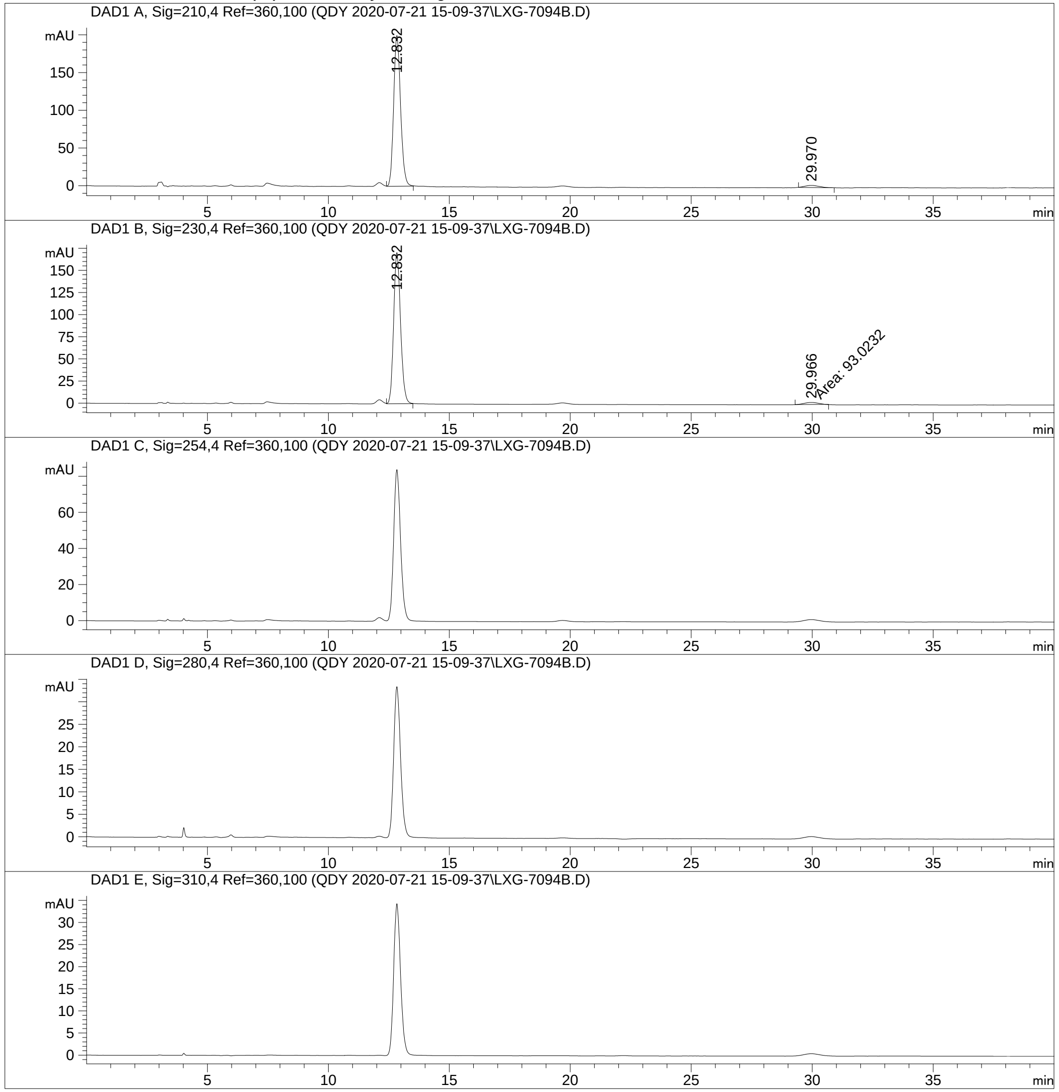

Data File C:\CHEM32\1\DATA\QDY 2020-07-21 15-09-37\LXG-7094B.D
Sample Name:

```
=====================================================================
                          Area Percent Report
=====================================================================

Sorted By             :      Signal
Multiplier            :      1.0000
Dilution              :      1.0000
Use Multiplier & Dilution Factor with ISTDs


Signal 1: DAD1 A, Sig=210,4 Ref=360,100

Peak RetTime Type  Width     Area      Height     Area
  #   [min]        [min]   [mAU*s]     [mAU]        %
----|-------|----|-------|----------|----------|--------|
   1  12.832 VB    0.3085 3983.05957  199.86339  97.0836
   2  29.970 BB    0.4977  119.65260    2.91032   2.9164

Totals :                  4102.71217  202.77371


Signal 2: DAD1 B, Sig=230,4 Ref=360,100

Peak RetTime Type  Width     Area      Height     Area
  #   [min]        [min]   [mAU*s]     [mAU]        %
----|-------|----|-------|----------|----------|--------|
   1  12.832 VB    0.3060 3396.93945  170.79012  97.3345
   2  29.966 MM    0.6613   93.02322    2.34452   2.6655

Totals :                  3489.96267  173.13463


Signal 3: DAD1 C, Sig=254,4 Ref=360,100


Signal 4: DAD1 D, Sig=280,4 Ref=360,100


Signal 5: DAD1 E, Sig=310,4 Ref=360,100

=====================================================================
                          *** End of Report ***
```

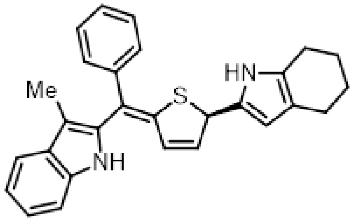

3w
(enantioenriched)

Supplementary Figure 189. HPLC spectrum of **3w** (enantioenriched)

Data File C:\CHEM32\1\DATA\QDY 2019-05-28 18-50-54\LXG-4123B-RAC.D
Sample Name:

===

Acq. Operator : Seq. Line : 2
Acq. Instrument : Instrument 1 Location : Vial 67
Injection Date : 5/28/2019 7:33:31 PM Inj : 1
Inj Volume : 5.000 µl
Acq. Method : C:\CHEM32\1\DATA\QDY 2019-05-28 18-50-54\AD-15-30.M
Last changed : 5/28/2019 7:32:38 PM
(modified after loading)
Analysis Method : C:\CHEM32\1\METHODS\OD-01-45-0.5.M
Last changed : 8/20/2020 6:37:54 PM
(modified after loading)
Additional Info : Peak(s) manually integrated

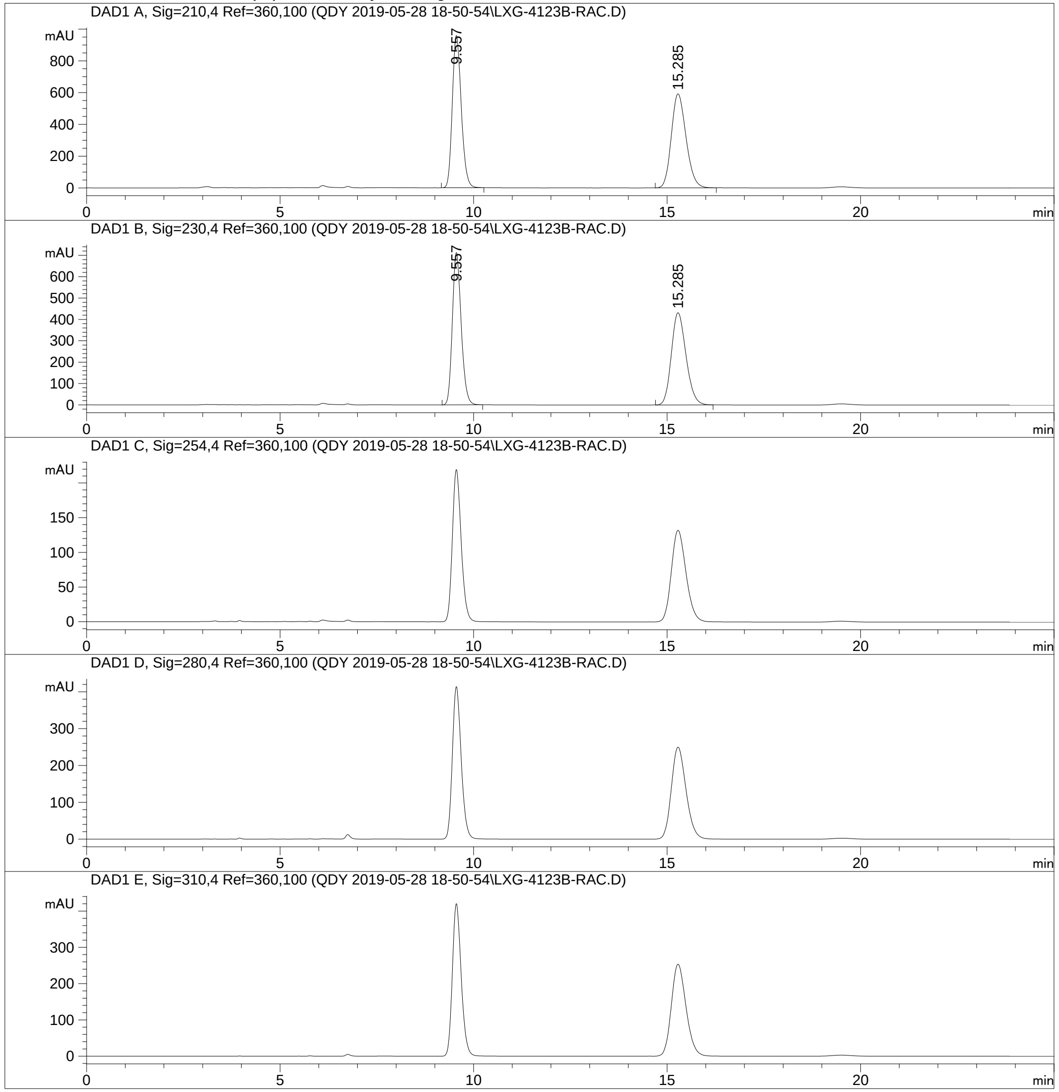

Data File C:\CHEM32\1\DATA\QDY 2019-05-28 18-50-54\LXG-4123B-RAC.D
Sample Name:

===

Area Percent Report

===

Sorted By : Signal
Multiplier : 1.0000
Dilution : 1.0000
Use Multiplier & Dilution Factor with ISTDs

Signal 1: DAD1 A, Sig=210,4 Ref=360,100

Peak #	RetTime [min]	Type	Width [min]	Area [mAU*s]	Height [mAU]	Area %
1	9.557	BB	0.2482	1.54742e4	959.04773	49.7055
2	15.285	BB	0.4108	1.56576e4	591.53687	50.2945

Totals : 3.11318e4 1550.58459

Signal 2: DAD1 B, Sig=230,4 Ref=360,100

Peak #	RetTime [min]	Type	Width [min]	Area [mAU*s]	Height [mAU]	Area %
1	9.557	BB	0.2456	1.13503e4	713.50891	49.9574
2	15.285	BB	0.4093	1.13697e4	431.63516	50.0426

Totals : 2.27199e4 1145.14407

Signal 3: DAD1 C, Sig=254,4 Ref=360,100

Signal 4: DAD1 D, Sig=280,4 Ref=360,100

Signal 5: DAD1 E, Sig=310,4 Ref=360,100

===

*** End of Report ***

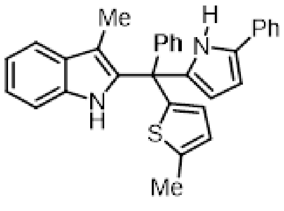

3x'
(racemic)

Supplementary Figure 190. HPLC spectrum of **3x'** (racemic)

Data File C:\CHEM32\1\DATA\QDY 2020-06-26 18-08-23\LXG-7072A.D
Sample Name:

===

Acq. Operator   :   Seq. Line :   2
Acq. Instrument : Instrument 1   Location : Vial 67
Injection Date : 6/26/2020 6:21:17 PM   Inj :   1
                                                  Inj Volume : 5.000 µl
Different Inj Volume from Sequence !   Actual Inj Volume : 4.000 µl
Acq. Method   : C:\CHEM32\1\DATA\QDY 2020-06-26 18-08-23\AD-15-25.M
Last changed   : 12/10/2016 9:14:33 PM
Analysis Method : C:\CHEM32\1\METHODS\LXG-IC-10-10.M
Last changed   : 7/4/2020 12:06:36 PM
                  (modified after loading)
Additional Info : Peak(s) manually integrated

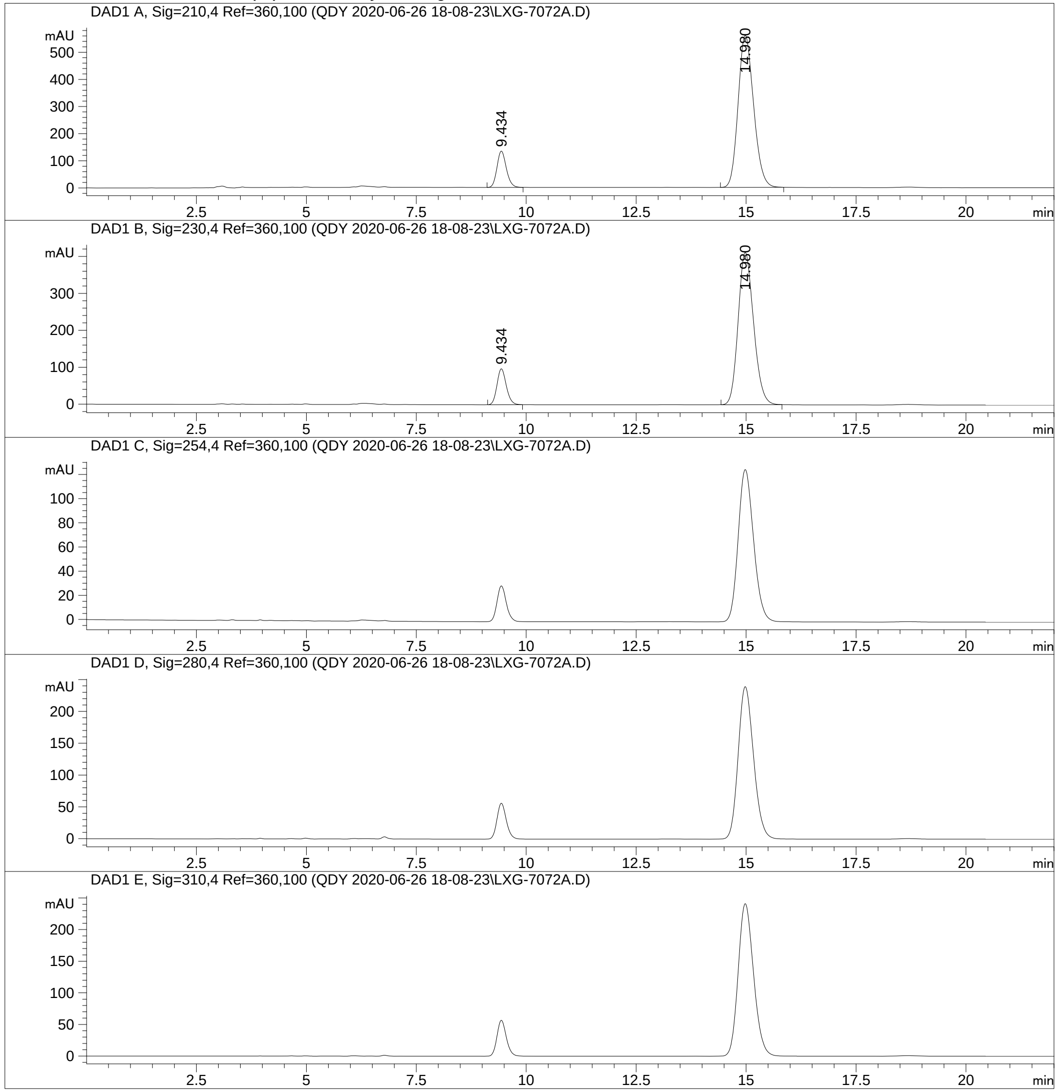

Data File C:\CHEM32\1\DATA\QDY 2020-06-26 18-08-23\LXG-7072A.D
Sample Name:

===

Area Percent Report

===

Sorted By   :   Signal
Multiplier   :   1.0000
Dilution   :   1.0000
Use Multiplier & Dilution Factor with ISTDs

Signal 1: DAD1 A, Sig=210,4 Ref=360,100

Peak #	RetTime [min]	Type	Width [min]	Area [mAU*s]	Height [mAU]	Area %
1	9.434	BB	0.2286	1982.63293	134.04240	12.6282
2	14.980	BB	0.3798	1.37174e4	559.99109	87.3718

Totals :   1.57000e4   694.03349

Signal 2: DAD1 B, Sig=230,4 Ref=360,100

Peak #	RetTime [min]	Type	Width [min]	Area [mAU*s]	Height [mAU]	Area %
1	9.434	BB	0.2283	1437.11633	97.32681	12.5179
2	14.980	BB	0.3782	1.00434e4	412.26346	87.4821

Totals :   1.14805e4   509.59027

Signal 3: DAD1 C, Sig=254,4 Ref=360,100

Signal 4: DAD1 D, Sig=280,4 Ref=360,100

Signal 5: DAD1 E, Sig=310,4 Ref=360,100

===

*** End of Report ***

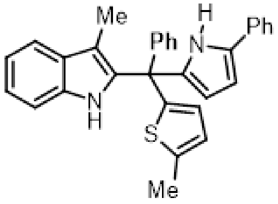

3x'
(enantioenriched)

Supplementary Figure 191. HPLC spectrum of **3x'** (enantioenriched)

```
Data File C:\CHEM32\1\DATA\QDY 2019-01-19 09-54-50\LXG-3073A-RAC.D
Sample Name:

    =====================================================================
    Acq. Operator   :                              Seq. Line :  21
    Acq. Instrument : Instrument 1                  Location : Vial 65
    Injection Date  : 1/19/2019 7:32:31 PM               Inj :   1
                                                  Inj Volume : 5.000 µl
    Acq. Method     : C:\CHEM32\1\DATA\QDY 2019-01-19 09-54-50\OD-10-40.M
    Last changed    : 1/19/2019 7:31:39 PM
                      (modified after loading)
    Analysis Method : C:\CHEM32\1\METHODS\OD-001-10-0.7.M
    Last changed    : 8/21/2019 8:26:51 PM
    Additional Info : Peak(s) manually integrated
```

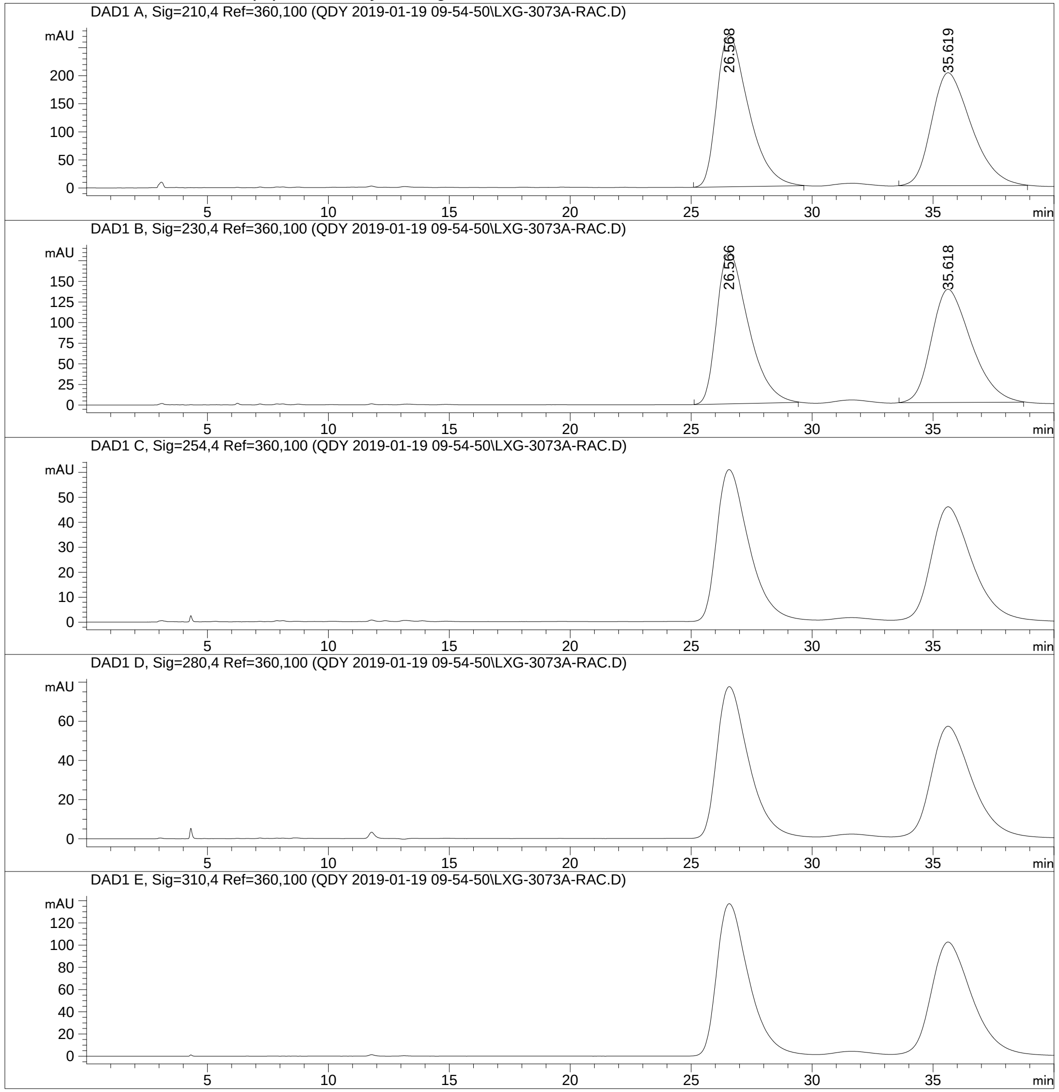


```
Data File C:\CHEM32\1\DATA\QDY 2019-01-19 09-54-50\LXG-3073A-RAC.D
Sample Name:

    =====================================================================
                             Area Percent Report
    =====================================================================

    Sorted By             :      Signal
    Multiplier            :      1.0000
    Dilution              :      1.0000
    Use Multiplier & Dilution Factor with ISTDs


    Signal 1: DAD1 A, Sig=210,4 Ref=360,100

    Peak RetTime Type  Width     Area      Height     Area
      #   [min]        [min]   [mAU*s]     [mAU]       %
    ----|-------|----|-------|----------|----------|--------|
       1  26.568 BB    1.3730 2.46620e4   269.65048  51.6475
       2  35.619 BB    1.7563 2.30886e4   201.27979  48.3525

    Totals :                  4.77506e4   470.93027


    Signal 2: DAD1 B, Sig=230,4 Ref=360,100

    Peak RetTime Type  Width     Area      Height     Area
      #   [min]        [min]   [mAU*s]     [mAU]       %
    ----|-------|----|-------|----------|----------|--------|
       1  26.566 BB    1.3693 1.66925e4   183.50626  51.5215
       2  35.618 BB    1.7553 1.57066e4   137.43654  48.4785

    Totals :                  3.23992e4   320.94279


    Signal 3: DAD1 C, Sig=254,4 Ref=360,100


    Signal 4: DAD1 D, Sig=280,4 Ref=360,100


    Signal 5: DAD1 E, Sig=310,4 Ref=360,100

    =====================================================================
                              *** End of Report ***
```

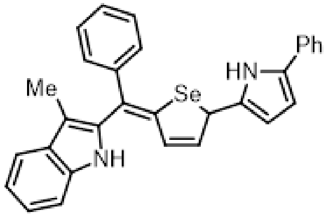

5a
(racemic)

Supplementary Figure 192. HPLC spectrum of **5a** (racemic)

```
=====================================================================
Acq. Operator   :                               Seq. Line :   2
Acq. Instrument : Instrument 1                   Location : Vial 77
Injection Date  : 8/21/2019 6:58:04 PM                Inj :   1
                                               Inj Volume : 5.000 µl
Acq. Method     : C:\CHEM32\1\DATA\QDY 2019-08-21 18-44-25\OD-10-40.M
Last changed    : 8/21/2019 6:57:10 PM
                  (modified after loading)
Analysis Method : C:\CHEM32\1\METHODS\OD-001-10-0.7.M
Last changed    : 8/21/2019 8:26:51 PM
Additional Info : Peak(s) manually integrated
```

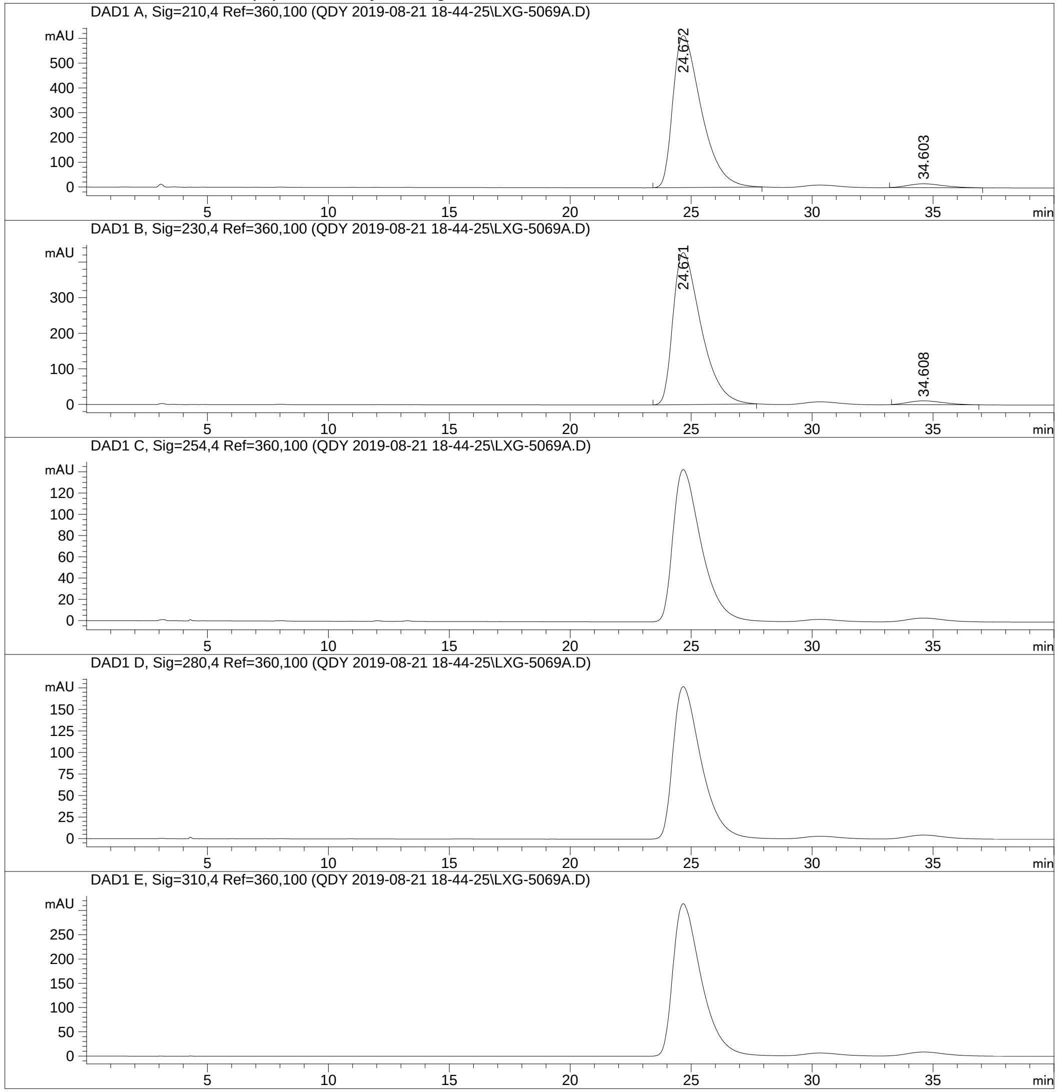

Data File C:\CHEM32\1\DATA\QDY 2019-08-21 18-44-25\LXG-5069A.D
Sample Name:

Area Percent Report

```
Sorted By             :      Signal
Multiplier            :      1.0000
Dilution              :      1.0000
Use Multiplier & Dilution Factor with ISTDs
```

Signal 1: DAD1 A, Sig=210,4 Ref=360,100

Peak #	RetTime [min]	Type	Width [min]	Area [mAU*s]	Height [mAU]	Area %
1	24.672	BB	1.2346	5.00090e4	613.77332	96.9052
2	34.603	BB	1.2039	1597.08594	15.63325	3.0948

Totals : 5.16060e4 629.40657

Signal 2: DAD1 B, Sig=230,4 Ref=360,100

Peak #	RetTime [min]	Type	Width [min]	Area [mAU*s]	Height [mAU]	Area %
1	24.671	BB	1.2190	3.44732e4	427.39185	96.9604
2	34.608	BB	1.3018	1080.71167	10.76000	3.0396

Totals : 3.55539e4 438.15184

Signal 3: DAD1 C, Sig=254,4 Ref=360,100

Signal 4: DAD1 D, Sig=280,4 Ref=360,100

Signal 5: DAD1 E, Sig=310,4 Ref=360,100

*** End of Report ***

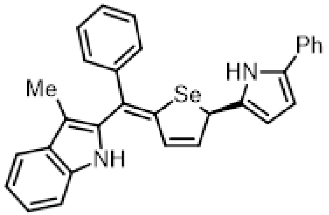

5a
(enantioenriched)

Supplementary Figure 193. HPLC spectrum of **5a** (enantioenriched)

===

Acq. Operator : Seq. Line : 6
Acq. Instrument : Instrument 1 Location : Vial 62
Injection Date : 7/1/2019 5:34:20 PM Inj : 1
Inj Volume : 5.000 µl
Acq. Method : C:\CHEM32\1\DATA\QDY 2019-07-01 16-06-16\OD-20-30.M
Last changed : 5/3/2016 10:14:35 AM
Analysis Method : C:\CHEM32\1\METHODS\OD-001-10-0.7.M
Last changed : 8/21/2019 8:26:51 PM
Additional Info : Peak(s) manually integrated

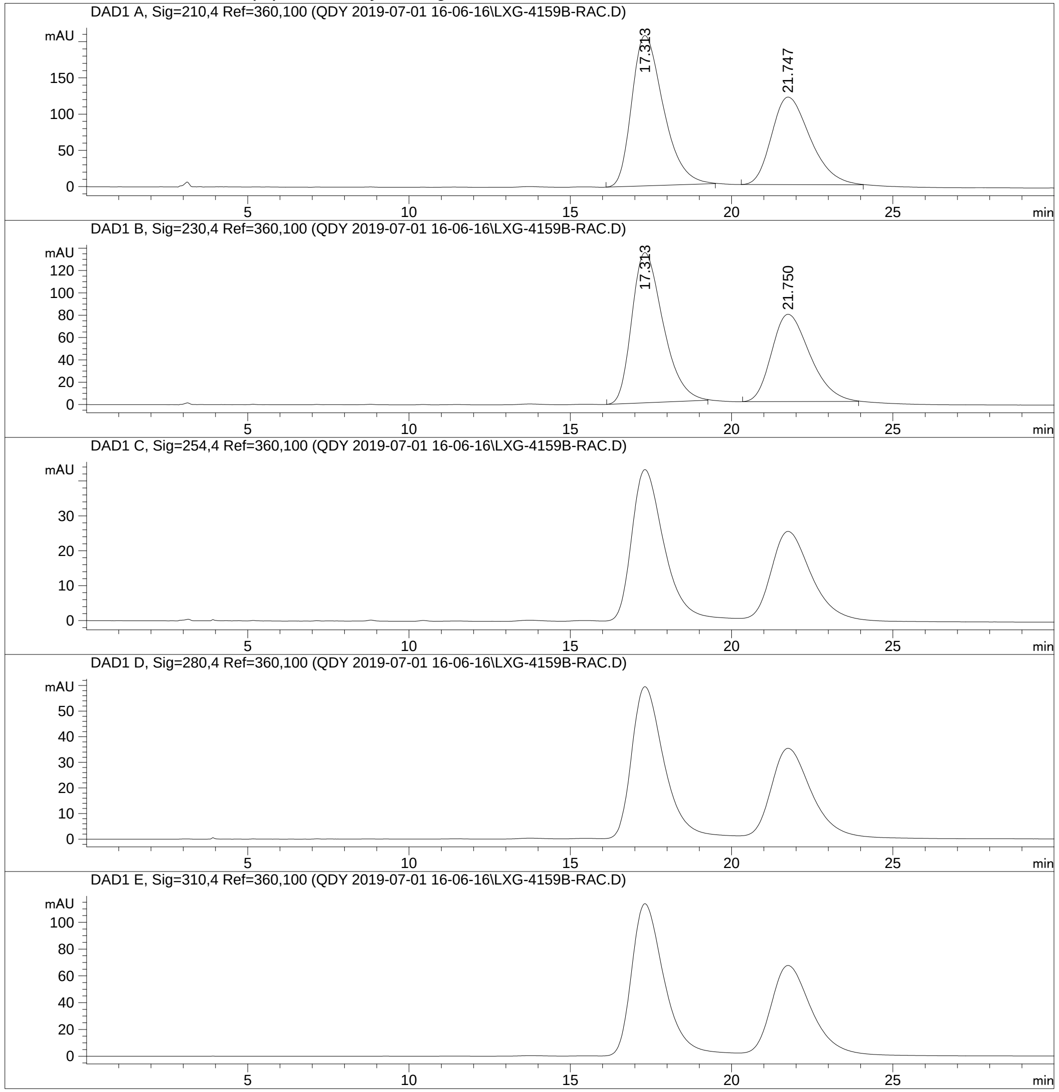

Data File C:\CHEM32\1\DATA\QDY 2019-07-01 16-06-16\LXG-4159B-RAC.D
Sample Name:

===

Area Percent Report

===

Sorted By : Signal
Multiplier : 1.0000
Dilution : 1.0000
Use Multiplier & Dilution Factor with ISTDs

Signal 1: DAD1 A, Sig=210,4 Ref=360,100

Peak #	RetTime [min]	Type	Width [min]	Area [mAU*s]	Height [mAU]	Area %
1	17.313	BB	1.0336	1.41090e4	207.41411	58.3510
2	21.747	BB	1.2449	1.00705e4	120.76174	41.6490

Totals : 2.41795e4 328.17585

Signal 2: DAD1 B, Sig=230,4 Ref=360,100

Peak #	RetTime [min]	Type	Width [min]	Area [mAU*s]	Height [mAU]	Area %
1	17.313	BB	1.0355	9056.10059	134.51042	58.3394
2	21.750	BB	1.2714	6467.02539	78.15504	41.6606

Totals : 1.55231e4 212.66546

Signal 3: DAD1 C, Sig=254,4 Ref=360,100

Signal 4: DAD1 D, Sig=280,4 Ref=360,100

Signal 5: DAD1 E, Sig=310,4 Ref=360,100

===

*** End of Report ***

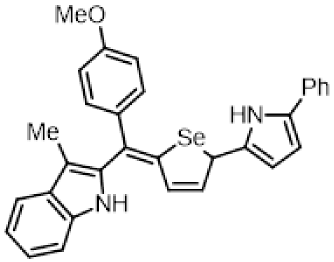

5b
(racemic)

Supplementary Figure 194. HPLC spectrum of **5b** (racemic)

Data File C:\CHEM32\1\DATA\QDY 2019-08-21 16-14-06\LXG-5069B.D
Sample Name:

===

Acq. Operator	:	Seq. Line :	2
Acq. Instrument	: Instrument 1	Location :	Vial 74
Injection Date	: 8/21/2019 4:27:17 PM	Inj :	1
		Inj Volume :	5.000 µl

Acq. Method : C:\CHEM32\1\DATA\QDY 2019-08-21 16-14-06\OD-20-30.M
Last changed : 5/3/2016 10:14:35 AM
Analysis Method : C:\CHEM32\1\METHODS\OD-001-10-0.7.M
Last changed : 8/21/2019 8:26:51 PM
Additional Info : Peak(s) manually integrated

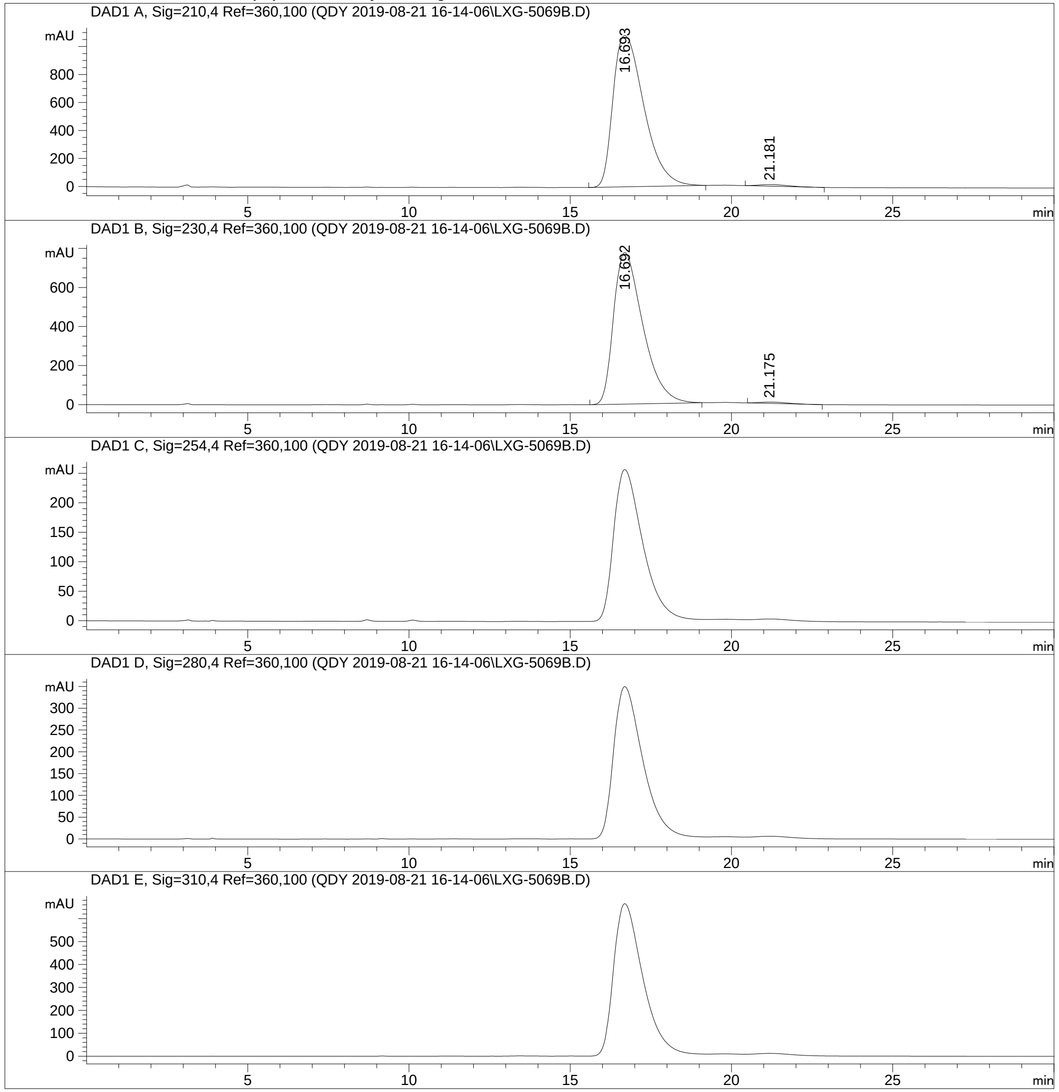

Data File C:\CHEM32\1\DATA\QDY 2019-08-21 16-14-06\LXG-5069B.D
Sample Name:

===
Area Percent Report
===

Sorted By : Signal
Multiplier : 1.0000
Dilution : 1.0000
Use Multiplier & Dilution Factor with ISTDs

Signal 1: DAD1 A, Sig=210,4 Ref=360,100

Peak #	RetTime [min]	Type	Width [min]	Area [mAU*s]	Height [mAU]	Area %
1	16.693	BB	1.0374	7.20678e4	1081.57385	99.0203
2	21.181	BB	0.7996	713.00836	11.33909	0.9797
Totals :				7.27808e4	1092.91294	

Signal 2: DAD1 B, Sig=230,4 Ref=360,100

Peak #	RetTime [min]	Type	Width [min]	Area [mAU*s]	Height [mAU]	Area %
1	16.692	BB	0.9693	4.90714e4	776.52466	99.1475
2	21.175	BB	0.8957	421.91968	6.91649	0.8525
Totals :				4.94934e4	783.44115	

Signal 3: DAD1 C, Sig=254,4 Ref=360,100

Signal 4: DAD1 D, Sig=280,4 Ref=360,100

Signal 5: DAD1 E, Sig=310,4 Ref=360,100

===
*** End of Report ***

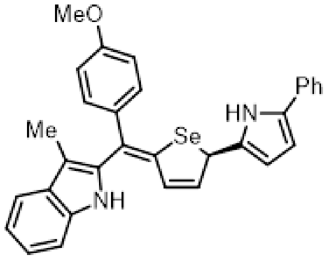

5b
(enantioenriched)

Supplementary Figure 195. HPLC spectrum of **5b** (enantioenriched)

Data File C:\CHEM32\1\DATA\QDY 2019-07-01 16-06-16\LXG-4159C-RAC.D
Sample Name:

===

Acq. Operator   :   Seq. Line :   7
Acq. Instrument : Instrument 1   Location : Vial 63
Injection Date : 7/1/2019 6:05:27 PM   Inj :   1
                                                  Inj Volume : 5.000 µl
Acq. Method   : C:\CHEM32\1\DATA\QDY 2019-07-01 16-06-16\OD-20-30.M
Last changed   : 5/3/2016 10:14:35 AM
Analysis Method : C:\CHEM32\1\METHODS\OD-001-10-0.7.M
Last changed   : 8/21/2019 8:26:51 PM
Additional Info : Peak(s) manually integrated

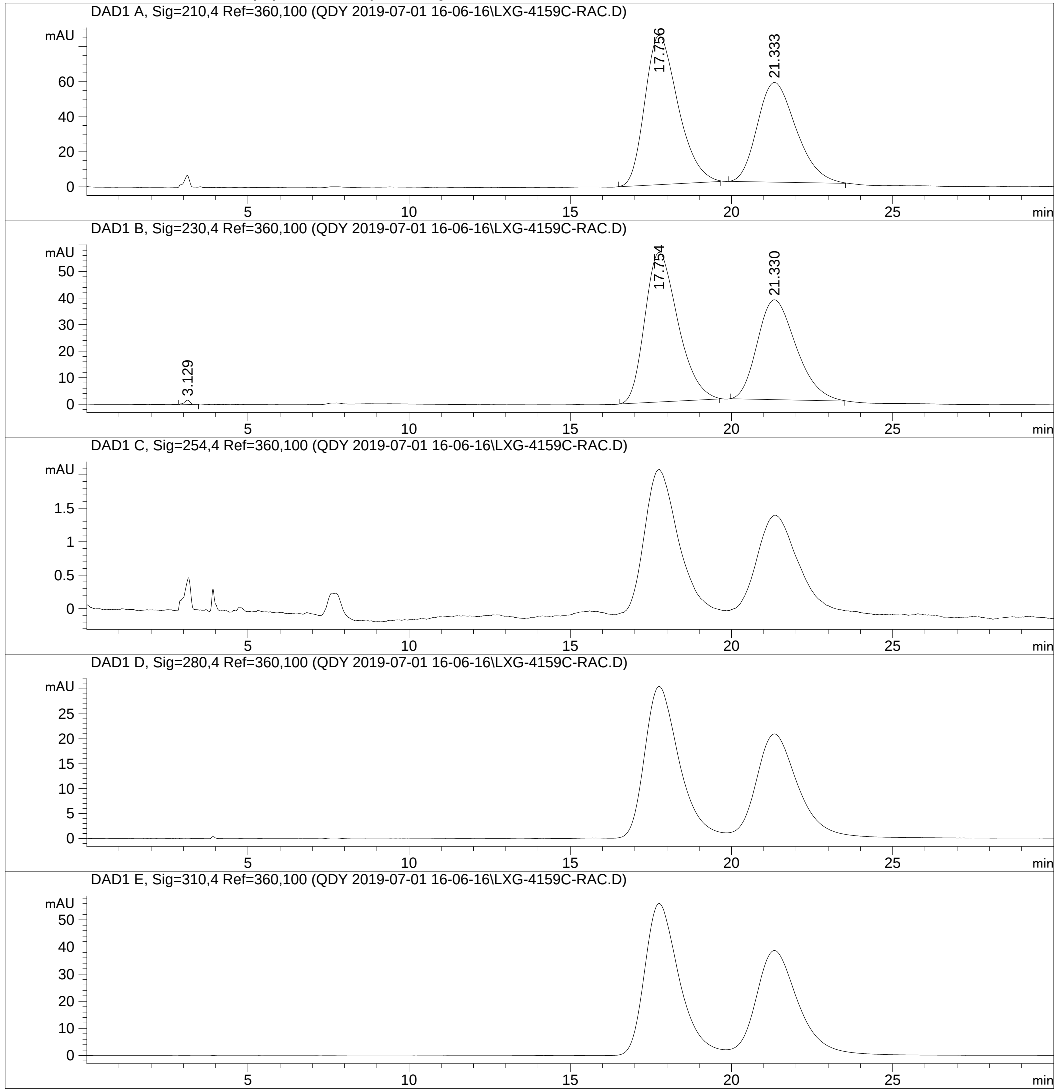

Data File C:\CHEM32\1\DATA\QDY 2019-07-01 16-06-16\LXG-4159C-RAC.D
Sample Name:

===

Area Percent Report

===

Sorted By   :   Signal
Multiplier   :   1.0000
Dilution   :   1.0000
Use Multiplier & Dilution Factor with ISTDs

Signal 1: DAD1 A, Sig=210,4 Ref=360,100

Peak #	RetTime [min]	Type	Width [min]	Area [mAU*s]	Height [mAU]	Area %
1	17.756	BB	1.0835	6086.35791	85.18929	56.0648
2	21.333	BB	1.2569	4769.57471	56.84240	43.9352

Totals :   1.08559e4   142.03168

Signal 2: DAD1 B, Sig=230,4 Ref=360,100

Peak #	RetTime [min]	Type	Width [min]	Area [mAU*s]	Height [mAU]	Area %
1	3.129	BB	0.1905	21.22253	1.66784	0.2952
2	17.754	BB	1.1010	4016.74316	56.39124	55.8705
3	21.330	BB	1.2950	3151.41309	37.63034	43.8343

Totals :   7189.37878   95.68942

Signal 3: DAD1 C, Sig=254,4 Ref=360,100

Signal 4: DAD1 D, Sig=280,4 Ref=360,100

Signal 5: DAD1 E, Sig=310,4 Ref=360,100

===

*** End of Report ***

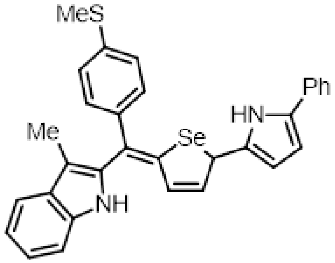

5c
(racemic)

Supplementary Figure 196. HPLC spectrum of **5c** (racemic)

```
=====================================================================
Acq. Operator   :                               Seq. Line :   3
Acq. Instrument : Instrument 1                   Location : Vial 75
Injection Date  : 8/21/2019 4:58:24 PM                Inj :   1
                                               Inj Volume : 5.000 µl
Acq. Method     : C:\CHEM32\1\DATA\QDY 2019-08-21 16-14-06\OD-20-30.M
Last changed    : 5/3/2016 10:14:35 AM
Analysis Method : C:\CHEM32\1\METHODS\OD-001-10-0.7.M
Last changed    : 8/21/2019 8:26:51 PM
Additional Info : Peak(s) manually integrated
```

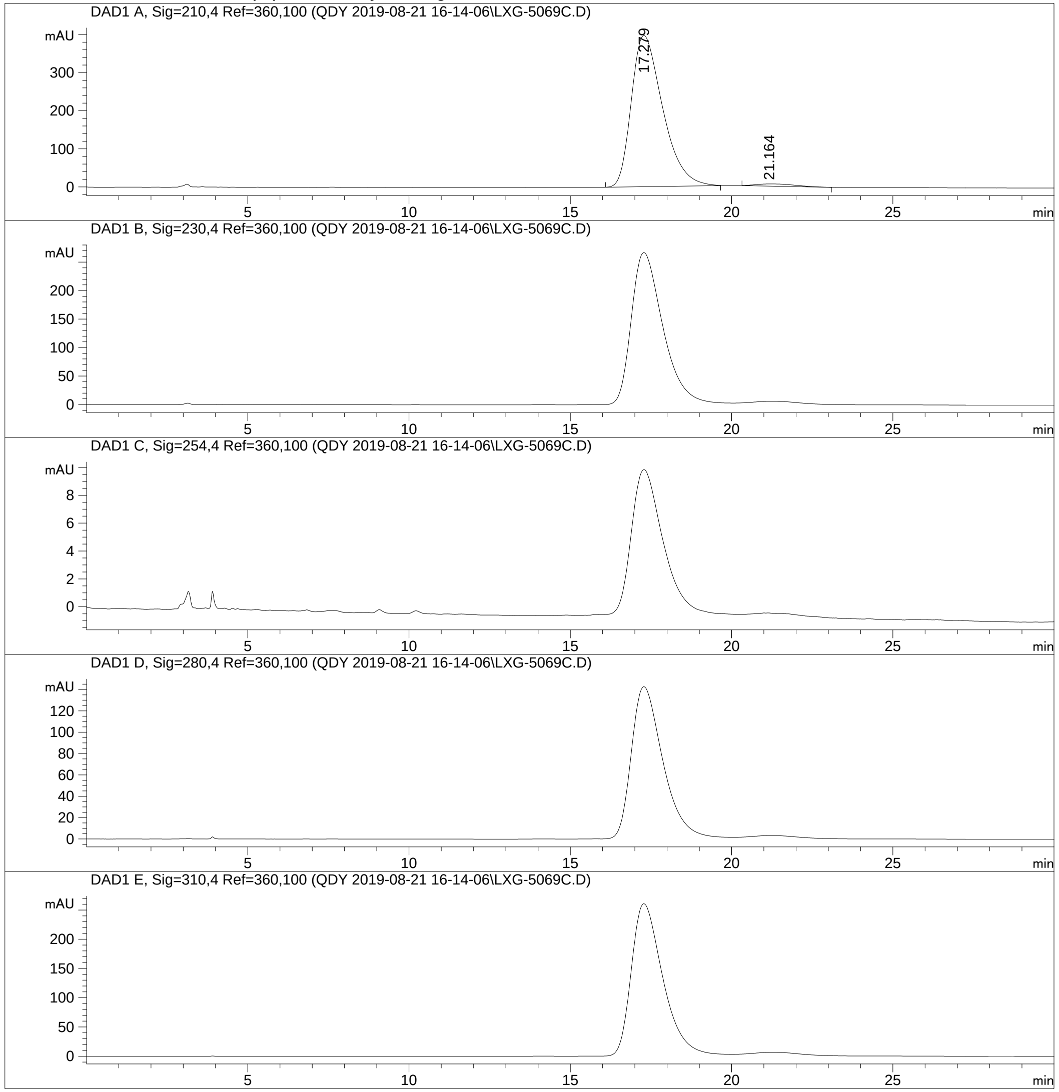

Data File C:\CHEM32\1\DATA\QDY 2019-08-21 16-14-06\LXG-5069C.D
Sample Name:

```
=====================================================================
                         Area Percent Report
=====================================================================

Sorted By             :      Signal
Multiplier            :      1.0000
Dilution              :      1.0000
Use Multiplier & Dilution Factor with ISTDs


Signal 1: DAD1 A, Sig=210,4 Ref=360,100

Peak RetTime Type  Width     Area      Height     Area
  #   [min]        [min]   [mAU*s]     [mAU]        %
----|-------|----|-------|----------|----------|--------|
   1  17.279 BB    1.0237 2.67879e4  396.73367  98.1361
   2  21.164 BB    1.0184  508.78308    5.92134   1.8639

Totals :                  2.72967e4   402.65501


Signal 2: DAD1 B, Sig=230,4 Ref=360,100


Signal 3: DAD1 C, Sig=254,4 Ref=360,100


Signal 4: DAD1 D, Sig=280,4 Ref=360,100


Signal 5: DAD1 E, Sig=310,4 Ref=360,100

=====================================================================
                          *** End of Report ***
```

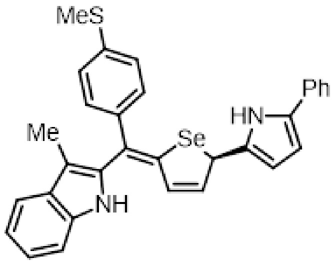

5c
(enantioenriched)

Supplementary Figure 197. HPLC spectrum of **5c** (enantioenriched)

===

Acq. Operator : Seq. Line : 9
Acq. Instrument : Instrument 1 Location : Vial 65
Injection Date : 7/1/2019 7:07:50 PM Inj : 1
Inj Volume : 5.000 µl
Acq. Method : C:\CHEM32\1\DATA\QDY 2019-07-01 16-06-16\OD-20-30.M
Last changed : 5/3/2016 10:14:35 AM
Analysis Method : C:\CHEM32\1\METHODS\OD-01-45-0.5.M
Last changed : 8/22/2020 12:14:44 PM
(modified after loading)
Additional Info : Peak(s) manually integrated

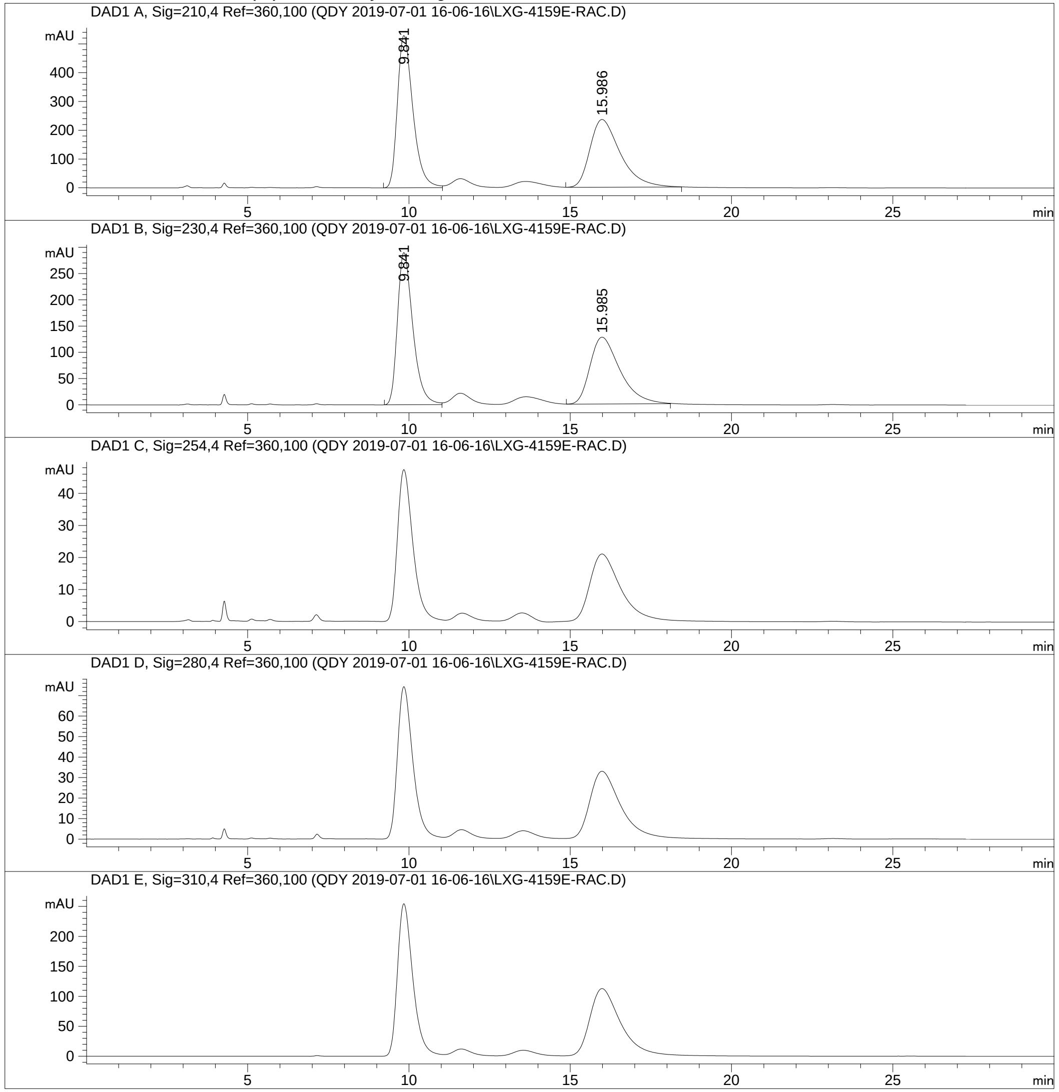

Data File C:\CHEM32\1\DATA\QDY 2019-07-01 16-06-16\LXG-4159E-RAC.D
Sample Name:

===

Area Percent Report

===

Sorted By : Signal
Multiplier : 1.0000
Dilution : 1.0000
Use Multiplier & Dilution Factor with ISTDs

Signal 1: DAD1 A, Sig=210,4 Ref=360,100

Peak #	RetTime [min]	Type	Width [min]	Area [mAU*s]	Height [mAU]	Area %
1	9.841	BV	0.5130	1.77344e4	529.16589	53.4554
2	15.986	VB	0.9867	1.54416e4	235.59125	46.5446

Totals : 3.31760e4 764.75714

Signal 2: DAD1 B, Sig=230,4 Ref=360,100

Peak #	RetTime [min]	Type	Width [min]	Area [mAU*s]	Height [mAU]	Area %
1	9.841	BB	0.5083	9648.12891	289.88367	53.9886
2	15.985	VB	0.9839	8222.54004	127.24164	46.0114

Totals : 1.78707e4 417.12531

Signal 3: DAD1 C, Sig=254,4 Ref=360,100

Signal 4: DAD1 D, Sig=280,4 Ref=360,100

Signal 5: DAD1 E, Sig=310,4 Ref=360,100

===

*** End of Report ***

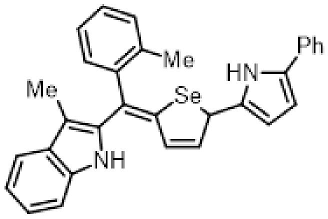

5d
(racemic)

Supplementary Figure 198. HPLC spectrum of **5d** (racemic)

==

Acq. Operator : Seq. Line : 5
Acq. Instrument : Instrument 1 Location : Vial 78
Injection Date : 8/22/2019 12:25:37 AM Inj : 1
Inj Volume : 5.000 µl
Acq. Method : C:\CHEM32\1\DATA\QDY 2019-08-21 21-17-50\OD-20-30.M
Last changed : 5/3/2016 10:14:35 AM
Analysis Method : C:\CHEM32\1\METHODS\OD-01-45-0.5.M
Last changed : 8/22/2020 12:14:44 PM
(modified after loading)
Additional Info : Peak(s) manually integrated

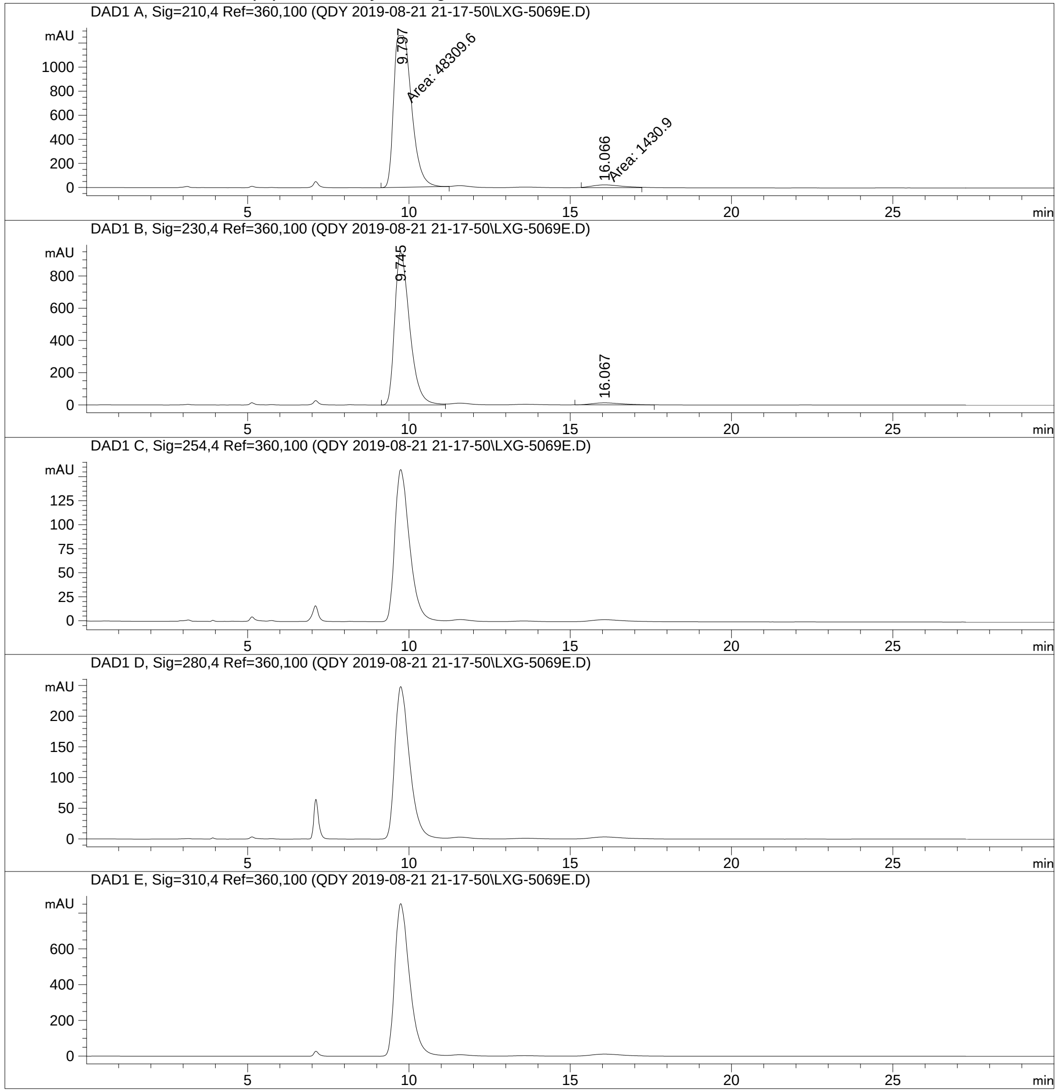

==
Area Percent Report
==

Sorted By : Signal
Multiplier : 1.0000
Dilution : 1.0000
Use Multiplier & Dilution Factor with ISTDs

Signal 1: DAD1 A, Sig=210,4 Ref=360,100

Peak #	RetTime [min]	Type	Width [min]	Area [mAU*s]	Height [mAU]	Area %
1	9.797	MM	0.6383	4.83096e4	1261.36279	97.1233
2	16.066	MM	1.0433	1430.90393	22.85865	2.8767

Totals : 4.97405e4 1284.22144

Signal 2: DAD1 B, Sig=230,4 Ref=360,100

Peak #	RetTime [min]	Type	Width [min]	Area [mAU*s]	Height [mAU]	Area %
1	9.745	BB	0.5004	3.06799e4	945.89362	97.3828
2	16.067	BB	0.9766	824.52002	12.51598	2.6172

Totals : 3.15044e4 958.40960

Signal 3: DAD1 C, Sig=254,4 Ref=360,100

Signal 4: DAD1 D, Sig=280,4 Ref=360,100

Signal 5: DAD1 E, Sig=310,4 Ref=360,100

==
*** End of Report ***

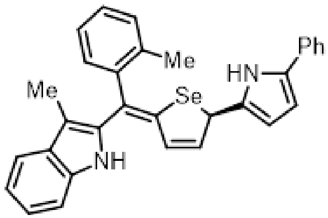

5d
(enantioenriched)

Supplementary Figure 199. HPLC spectrum of **5d** (enantioenriched)

Data File C:\CHEM32\1\DATA\QDY 2019-07-01 16-06-16\LXG-4159F-RAC.D
Sample Name:

===

```
Acq. Operator   :                        Seq. Line :  10
Acq. Instrument : Instrument 1            Location : Vial 66
Injection Date  : 7/1/2019 7:39:26 PM          Inj :   1
                                        Inj Volume : 5.000 µl
Acq. Method     : C:\CHEM32\1\DATA\QDY 2019-07-01 16-06-16\OD-20-30.M
Last changed    : 5/3/2016 10:14:35 AM
Analysis Method : C:\CHEM32\1\METHODS\OD-001-10-0.7.M
Last changed    : 8/21/2019 8:26:51 PM
Additional Info : Peak(s) manually integrated
```

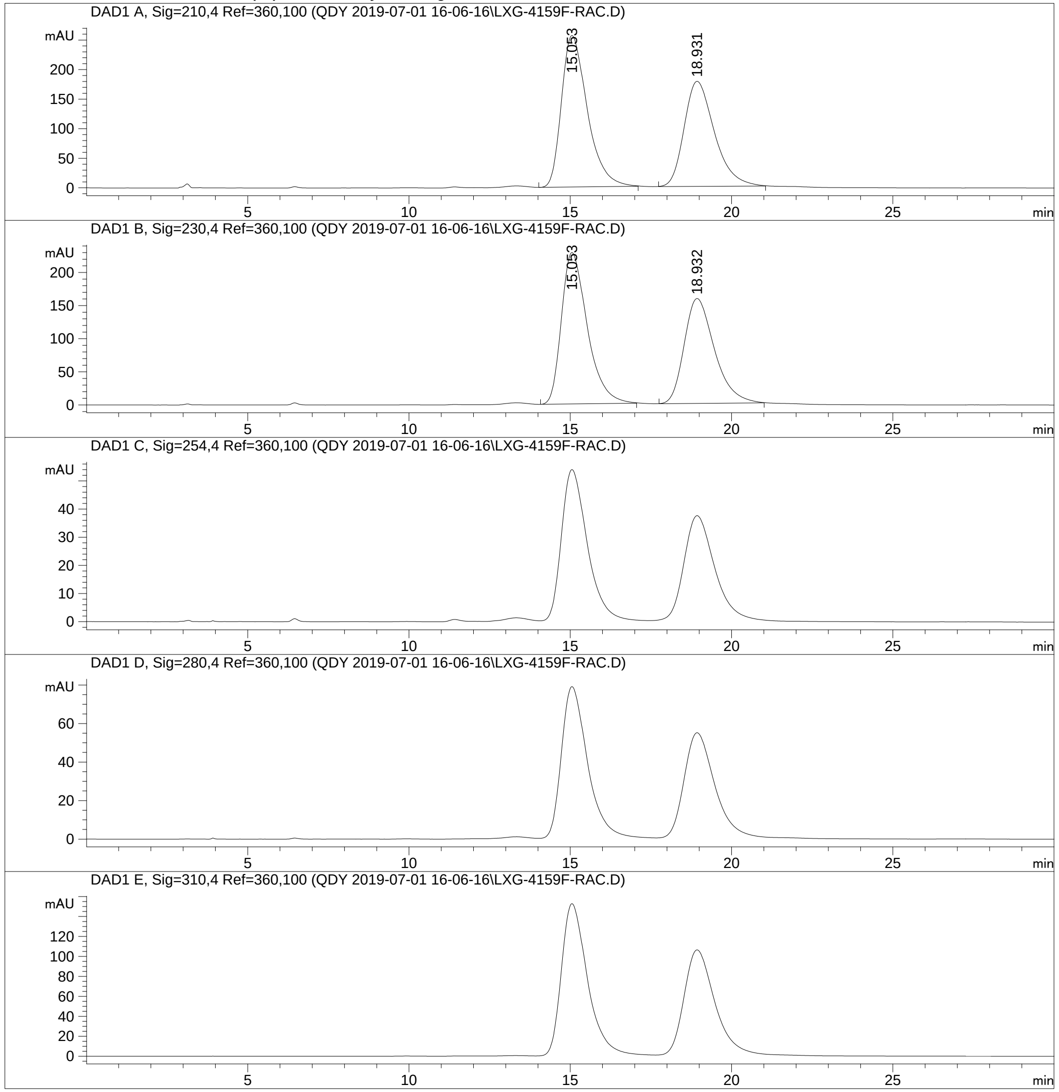

Data File C:\CHEM32\1\DATA\QDY 2019-07-01 16-06-16\LXG-4159F-RAC.D
Sample Name:

===

Area Percent Report

===

Sorted By : Signal
Multiplier : 1.0000
Dilution : 1.0000
Use Multiplier & Dilution Factor with ISTDs

Signal 1: DAD1 A, Sig=210,4 Ref=360,100

Peak #	RetTime [min]	Type	Width [min]	Area [mAU*s]	Height [mAU]	Area %
1	15.053	VB	0.8594	1.42659e4	256.28500	55.1132
2	18.931	BB	0.9986	1.16188e4	177.75638	44.8868
Totals :				2.58847e4	434.04138	

Signal 2: DAD1 B, Sig=230,4 Ref=360,100

Peak #	RetTime [min]	Type	Width [min]	Area [mAU*s]	Height [mAU]	Area %
1	15.053	BB	0.8566	1.27061e4	228.56815	55.1172
2	18.932	BB	1.0018	1.03468e4	158.45694	44.8828
Totals :				2.30529e4	387.02509	

Signal 3: DAD1 C, Sig=254,4 Ref=360,100

Signal 4: DAD1 D, Sig=280,4 Ref=360,100

Signal 5: DAD1 E, Sig=310,4 Ref=360,100

===

*** End of Report ***

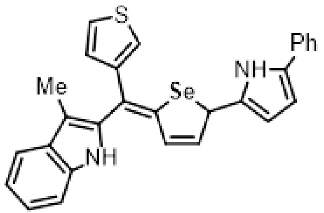

5e
(racemic)

Supplementary Figure 200. HPLC spectrum of **5e** (racemic)

Data File C:\CHEM32\1\DATA\QDY 2019-08-21 16-14-06\LXG-5069D.D
Sample Name:

```
=====================================================================
Acq. Operator   :                              Seq. Line :   4
Acq. Instrument : Instrument 1                  Location : Vial 76
Injection Date  : 8/21/2019 5:29:53 PM               Inj :   1
                                              Inj Volume : 5.000 µl
Acq. Method     : C:\CHEM32\1\DATA\QDY 2019-08-21 16-14-06\OD-20-30.M
Last changed    : 5/3/2016 10:14:35 AM
Analysis Method : C:\CHEM32\1\METHODS\OD-001-10-0.7.M
Last changed    : 8/21/2019 8:26:51 PM
Additional Info : Peak(s) manually integrated
```

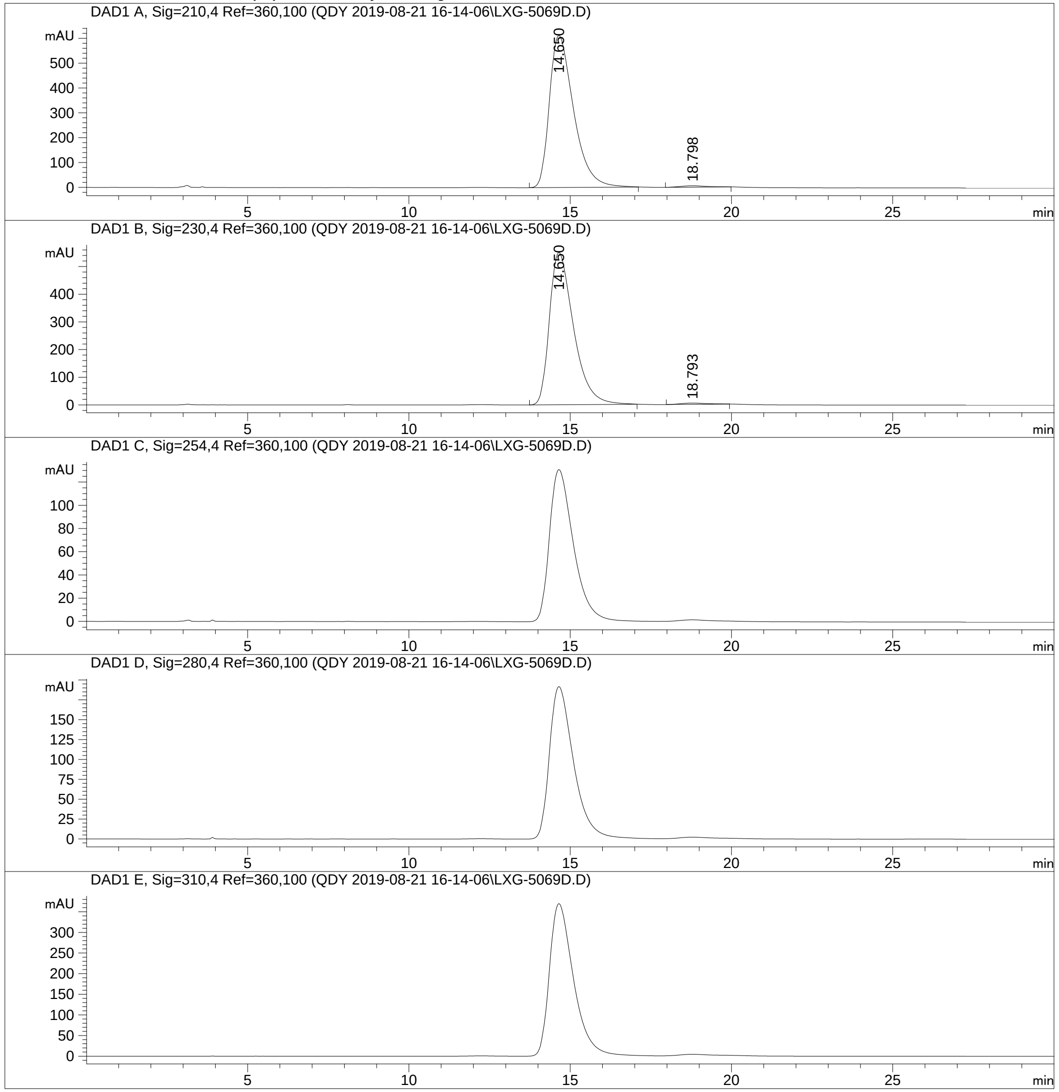

Data File C:\CHEM32\1\DATA\QDY 2019-08-21 16-14-06\LXG-5069D.D
Sample Name:

```
=====================================================================
                         Area Percent Report
=====================================================================

Sorted By             :      Signal
Multiplier            :      1.0000
Dilution              :      1.0000
Use Multiplier & Dilution Factor with ISTDs
```

Signal 1: DAD1 A, Sig=210,4 Ref=360,100

Peak #	RetTime [min]	Type	Width [min]	Area [mAU*s]	Height [mAU]	Area %
1	14.650	BB	0.8012	3.19468e4	611.85522	98.9937
2	18.798	BB	0.7090	324.73480	5.56324	1.0063

Totals : 3.22715e4 617.41846

Signal 2: DAD1 B, Sig=230,4 Ref=360,100

Peak #	RetTime [min]	Type	Width [min]	Area [mAU*s]	Height [mAU]	Area %
1	14.650	BB	0.7961	2.85797e4	550.14484	99.0468
2	18.793	BB	0.8295	275.05164	4.79296	0.9532

Totals : 2.88548e4 554.93780

Signal 3: DAD1 C, Sig=254,4 Ref=360,100

Signal 4: DAD1 D, Sig=280,4 Ref=360,100

Signal 5: DAD1 E, Sig=310,4 Ref=360,100

===
*** End of Report ***

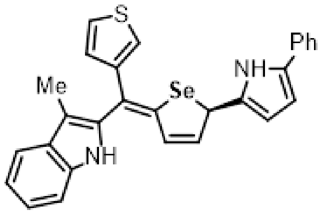

5e
(enantioenriched)

Supplementary Figure 201. HPLC spectrum of **5e** (enantioenriched)

===

Acq. Operator	:	Seq. Line :	2
Acq. Instrument	: Instrument 1	Location :	Vial 61
Injection Date	: 7/3/2020 11:29:15 AM	Inj :	1
		Inj Volume :	5.000 µl

Acq. Method : C:\CHEM32\1\DATA\QDY 2020-07-03 11-16-20\AD-20-30.M
Last changed : 6/15/2018 10:29:43 AM
Analysis Method : C:\CHEM32\1\METHODS\LXG-IC-10-10.M
Last changed : 7/6/2020 6:52:33 PM
(modified after loading)
Additional Info : Peak(s) manually integrated

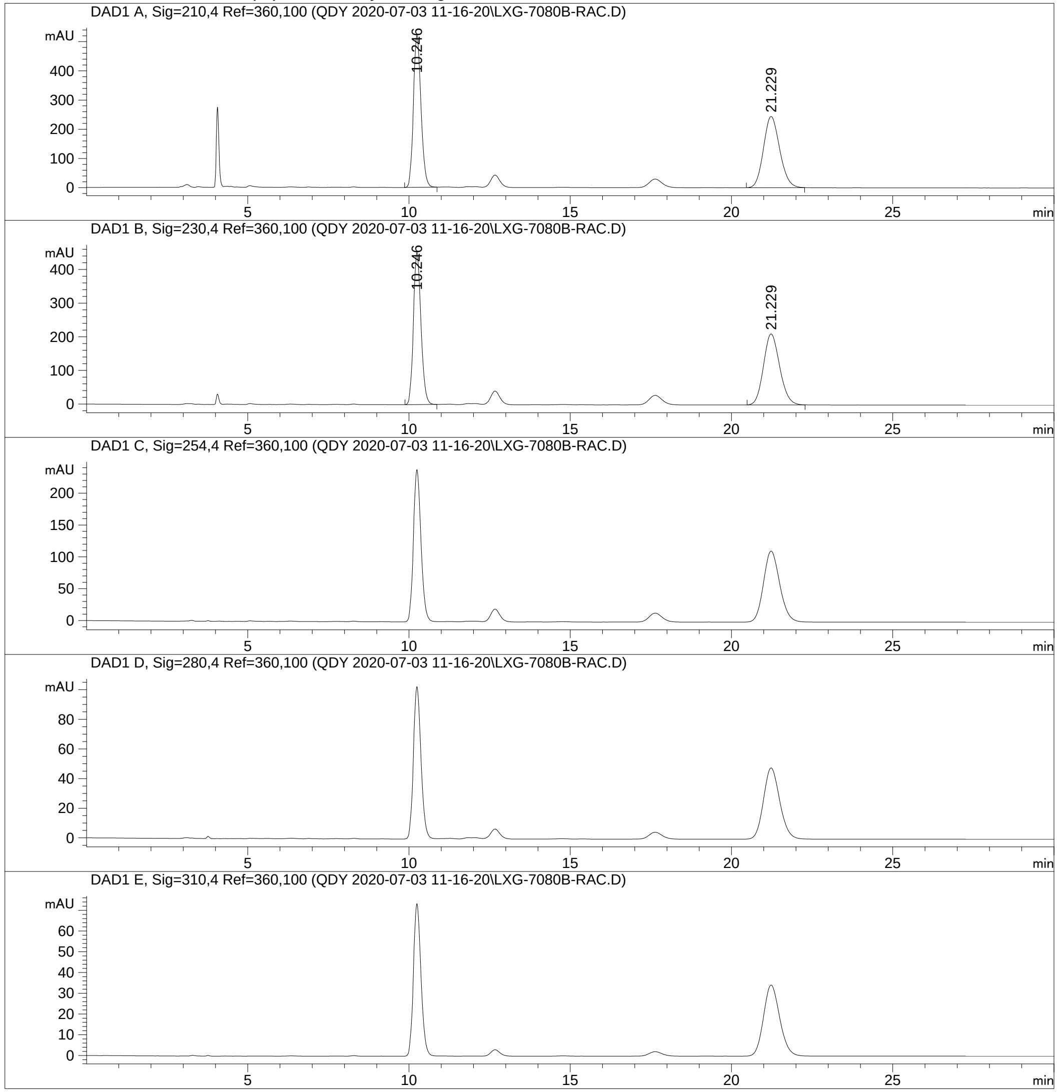

Data File C:\CHEM32\1\DATA\QDY 2020-07-03 11-16-20\LXG-7080B-RAC.D
Sample Name:

===
Area Percent Report
===

Sorted By : Signal
Multiplier : 1.0000
Dilution : 1.0000
Use Multiplier & Dilution Factor with ISTDs

Signal 1: DAD1 A, Sig=210,4 Ref=360,100

Peak #	RetTime [min]	Type	Width [min]	Area [mAU*s]	Height [mAU]	Area %
1	10.246	BB	0.2524	8486.69434	520.03217	49.8361
2	21.229	BB	0.5401	8542.52637	244.19833	50.1639

Totals : 1.70292e4 764.23050

Signal 2: DAD1 B, Sig=230,4 Ref=360,100

Peak #	RetTime [min]	Type	Width [min]	Area [mAU*s]	Height [mAU]	Area %
1	10.246	BB	0.2496	7347.05469	452.02606	49.9152
2	21.229	BB	0.5414	7372.02881	211.10132	50.0848

Totals : 1.47191e4 663.12738

Signal 3: DAD1 C, Sig=254,4 Ref=360,100

Signal 4: DAD1 D, Sig=280,4 Ref=360,100

Signal 5: DAD1 E, Sig=310,4 Ref=360,100

===
*** End of Report ***

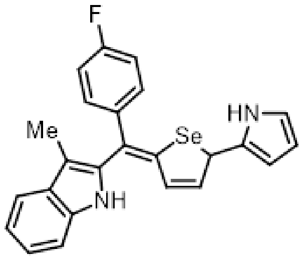

5f
(racemic)

Supplementary Figure 205. HPLC spectrum of **5f** (racemic)

```
=====================================================================
Acq. Operator   :                                 Seq. Line :   3
Acq. Instrument : Instrument 1                     Location : Vial 68
Injection Date  : 7/4/2020 2:22:56 PM                   Inj :   1
                                                 Inj Volume : 5.000 µl
Different Inj Volume from Sequence !     Actual Inj Volume : 4.000 µl
Acq. Method     : C:\CHEM32\1\DATA\QDY 2020-07-04 13-48-53\AD-20-30.M
Last changed    : 6/15/2018 10:29:43 AM
Analysis Method : C:\CHEM32\1\METHODS\LXG-IC-10-10.M
Last changed    : 7/4/2020 3:31:39 PM
                  (modified after loading)
Additional Info : Peak(s) manually integrated
```

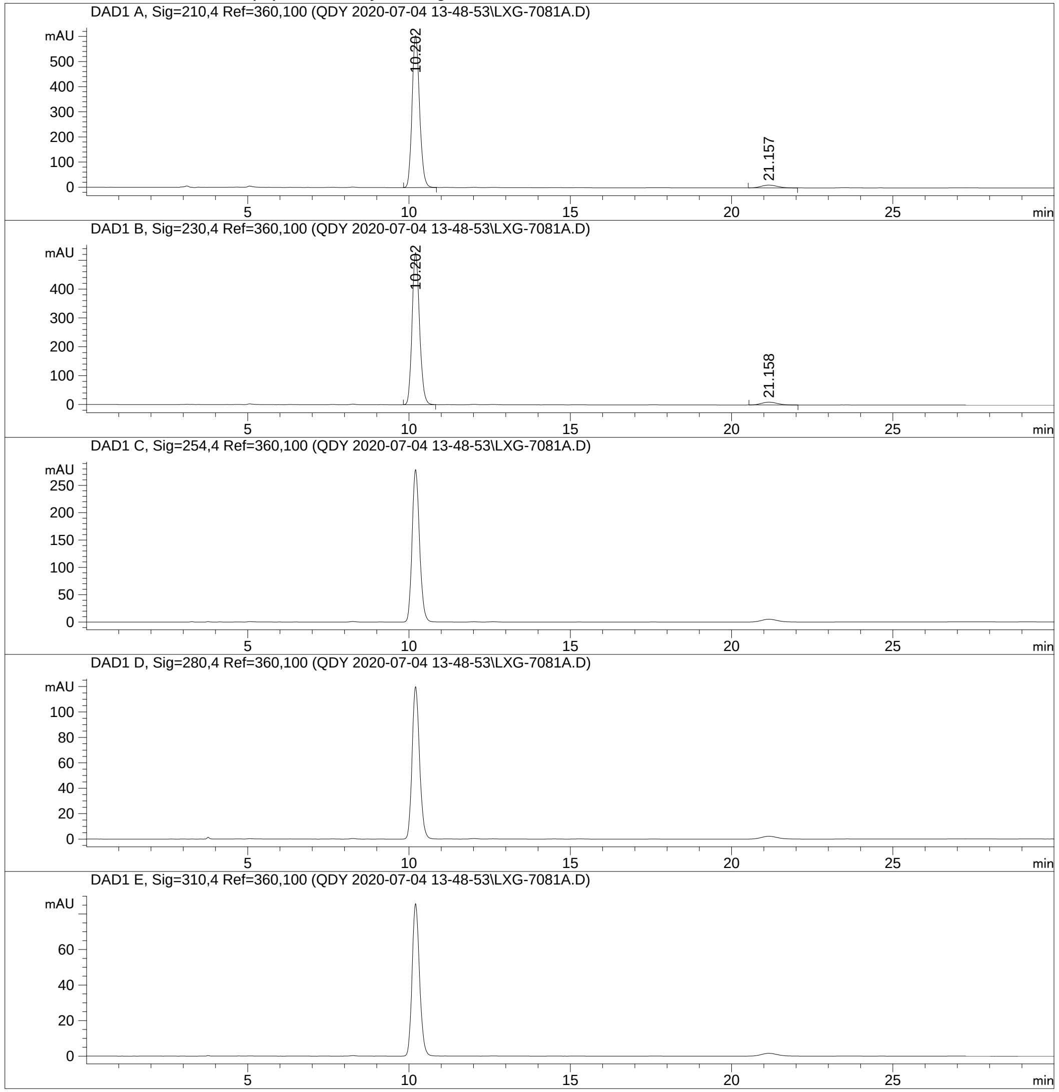

Data File C:\CHEM32\1\DATA\QDY 2020-07-04 13-48-53\LXG-7081A.D
Sample Name:

```
=====================================================================
                          Area Percent Report
=====================================================================

Sorted By             :      Signal
Multiplier            :      1.0000
Dilution              :      1.0000
Use Multiplier & Dilution Factor with ISTDs


Signal 1: DAD1 A, Sig=210,4 Ref=360,100

Peak RetTime Type  Width     Area      Height     Area
  #   [min]        [min]   [mAU*s]     [mAU]        %
----|-------|----|-------|----------|----------|--------|
   1  10.202 BB    0.2490 9701.01563  605.15961  96.1062
   2  21.157 BB    0.5394  393.03787   11.25435   3.8938

Totals :                  1.00941e4   616.41396


Signal 2: DAD1 B, Sig=230,4 Ref=360,100

Peak RetTime Type  Width     Area      Height     Area
  #   [min]        [min]   [mAU*s]     [mAU]        %
----|-------|----|-------|----------|----------|--------|
   1  10.202 BB    0.2462 8420.31055  527.64069  96.1535
   2  21.158 BB    0.5361  336.84317    9.77255   3.8465

Totals :                  8757.15372  537.41323


Signal 3: DAD1 C, Sig=254,4 Ref=360,100


Signal 4: DAD1 D, Sig=280,4 Ref=360,100


Signal 5: DAD1 E, Sig=310,4 Ref=360,100

=====================================================================
                          *** End of Report ***
```

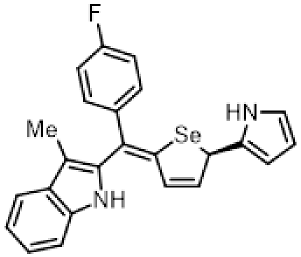

5f
(enantioenriched)

Supplementary Figure 203. HPLC spectrum of **5f** (enantioenriched)

Data File C:\CHEM32\1\DATA\QDY 2019-08-22 14-18-14\LXG-5057E-RAC2.D
Sample Name:

===

Acq. Operator : Seq. Line : 5
Acq. Instrument : Instrument 1 Location : Vial 62
Injection Date : 8/22/2019 3:35:40 PM Inj : 1
Inj Volume : 5.000 µl
Acq. Method : C:\CHEM32\1\DATA\QDY 2019-08-22 14-18-14\AD-30-40.M
Last changed : 8/22/2019 3:34:47 PM
(modified after loading)
Analysis Method : C:\CHEM32\1\METHODS\OD-001-10-0.7.M
Last changed : 8/23/2019 7:50:41 PM
(modified after loading)
Additional Info : Peak(s) manually integrated

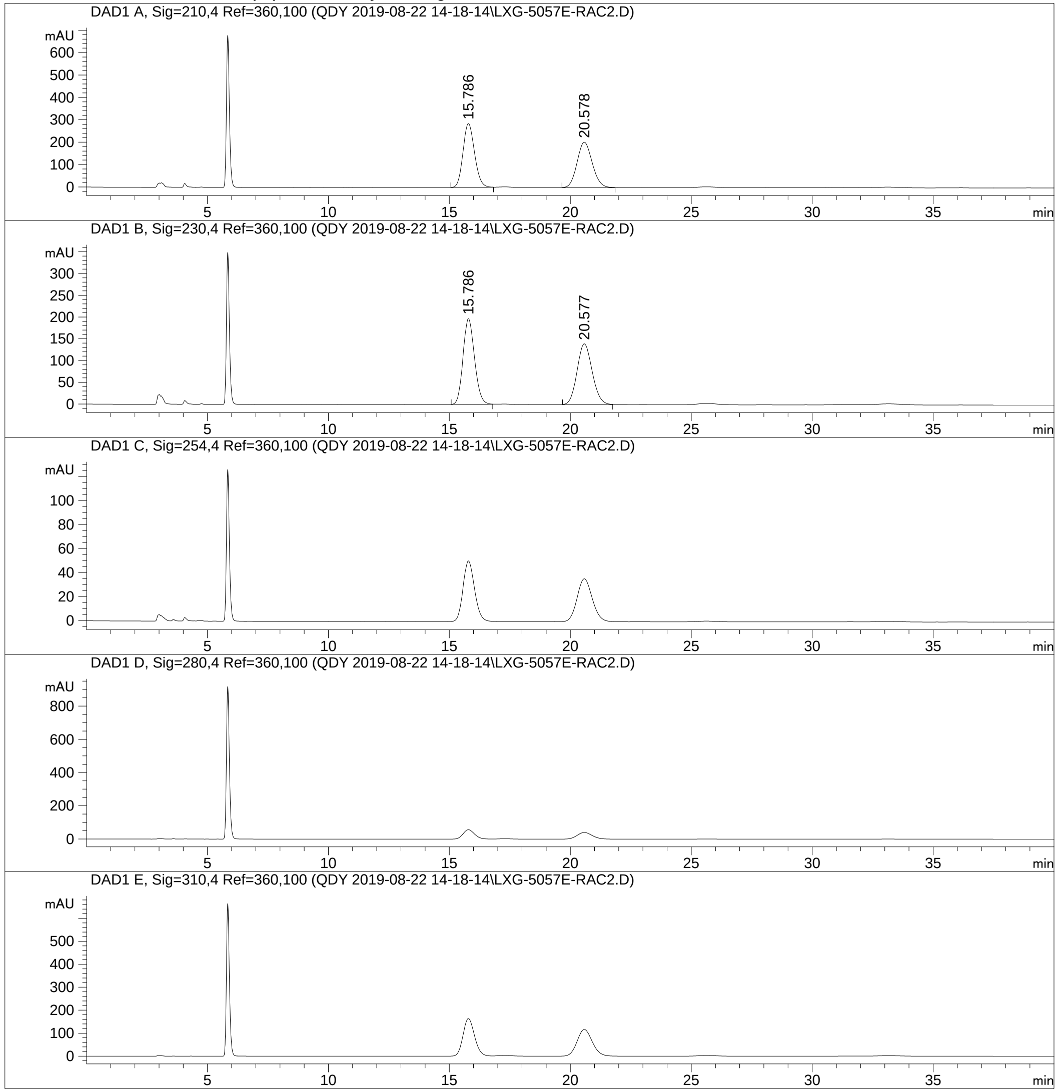

Data File C:\CHEM32\1\DATA\QDY 2019-08-22 14-18-14\LXG-5057E-RAC2.D
Sample Name:

===

Area Percent Report

===

Sorted By : Signal
Multiplier : 1.0000
Dilution : 1.0000
Use Multiplier & Dilution Factor with ISTDs

Signal 1: DAD1 A, Sig=210,4 Ref=360,100

Peak #	RetTime [min]	Type	Width [min]	Area [mAU*s]	Height [mAU]	Area %
1	15.786	BB	0.5160	9434.80957	285.17068	51.6033
2	20.578	BB	0.6792	8848.51953	202.74086	48.3967

Totals : 1.82833e4 487.91154

Signal 2: DAD1 B, Sig=230,4 Ref=360,100

Peak #	RetTime [min]	Type	Width [min]	Area [mAU*s]	Height [mAU]	Area %
1	15.786	BB	0.5137	6511.24707	196.96703	51.7182
2	20.577	BB	0.6776	6078.59863	139.70050	48.2818

Totals : 1.25898e4 336.66753

Signal 3: DAD1 C, Sig=254,4 Ref=360,100

Signal 4: DAD1 D, Sig=280,4 Ref=360,100

Signal 5: DAD1 E, Sig=310,4 Ref=360,100

===

*** End of Report ***

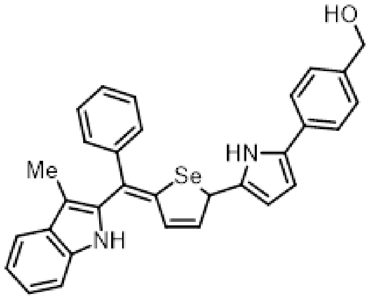

5g
(racemic)

Supplementary Figure 204. HPLC spectrum of **5g** (racemic)

```
=====================================================================
Acq. Operator   :                               Seq. Line :   2
Acq. Instrument : Instrument 1                   Location : Vial 66
Injection Date  : 8/24/2019 12:49:22 PM               Inj :   1
                                              Inj Volume : 5.000 µl
Acq. Method     : C:\CHEM32\1\DATA\QDY 2019-08-24 12-36-20\AD-30-40.M
Last changed    : 8/24/2019 12:48:29 PM
                  (modified after loading)
Analysis Method : C:\CHEM32\1\METHODS\OD-001-10-0.7.M
Last changed    : 8/23/2019 7:50:41 PM
                  (modified after loading)
Additional Info : Peak(s) manually integrated
```

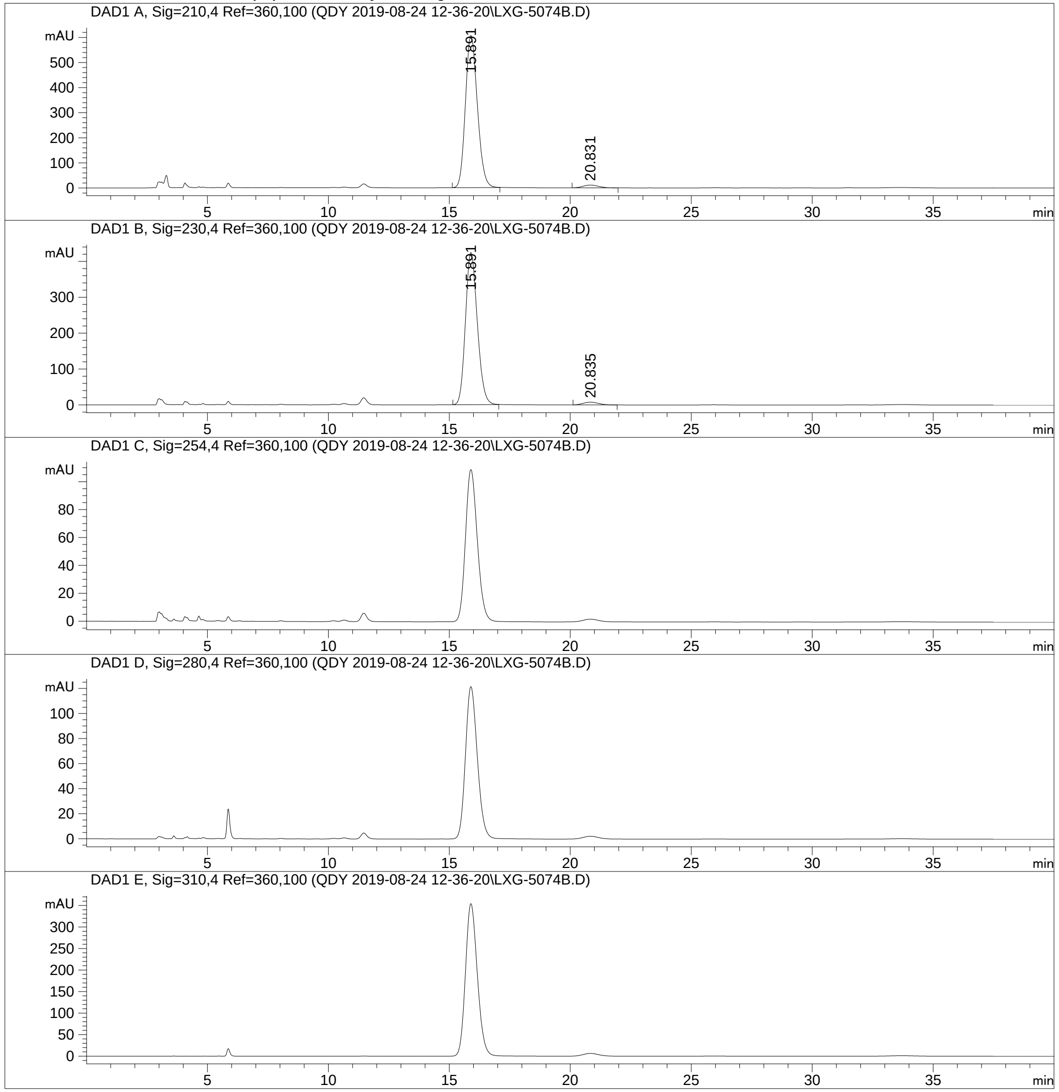

Data File C:\CHEM32\1\DATA\QDY 2019-08-24 12-36-20\LXG-5074B.D
Sample Name:

```
=====================================================================
                         Area Percent Report
=====================================================================

Sorted By             :      Signal
Multiplier            :      1.0000
Dilution              :      1.0000
Use Multiplier & Dilution Factor with ISTDs


Signal 1: DAD1 A, Sig=210,4 Ref=360,100

Peak RetTime Type  Width     Area      Height     Area
  #   [min]        [min]   [mAU*s]     [mAU]        %
----|-------|----|-------|----------|----------|--------|
   1  15.891 BB    0.5345 2.08257e4  606.72327  97.6474
   2  20.831 BB    0.6835  501.74051   11.22448   2.3526

Totals :                  2.13275e4  617.94774


Signal 2: DAD1 B, Sig=230,4 Ref=360,100

Peak RetTime Type  Width     Area      Height     Area
  #   [min]        [min]   [mAU*s]     [mAU]        %
----|-------|----|-------|----------|----------|--------|
   1  15.891 BB    0.5294 1.44505e4  424.22931  97.6875
   2  20.835 BB    0.6690  342.07294    7.71936   2.3125

Totals :                  1.47926e4  431.94867


Signal 3: DAD1 C, Sig=254,4 Ref=360,100


Signal 4: DAD1 D, Sig=280,4 Ref=360,100


Signal 5: DAD1 E, Sig=310,4 Ref=360,100

=====================================================================
                          *** End of Report ***
```

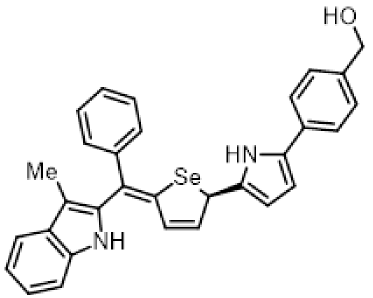

5g
(enantioenriched)

Supplementary Figure 205. HPLC spectrum of **5g** (enantioenriched)

```
=====================================================================
Acq. Operator   :                               Seq. Line :   5
Acq. Instrument : Instrument 1                   Location : Vial 64
Injection Date  : 7/14/2020 1:28:21 AM                Inj :   1
                                               Inj Volume : 5.000 µl
Different Inj Volume from Sequence !     Actual Inj Volume : 3.000 µl
Acq. Method     : C:\CHEM32\1\DATA\QDY 2020-07-13 23-41-55\AD-20-30.M
Last changed    : 6/15/2018 10:29:43 AM
Analysis Method : C:\CHEM32\1\METHODS\AD-008-50.M
Last changed    : 7/25/2020 7:02:41 PM
                  (modified after loading)
Additional Info : Peak(s) manually integrated
```

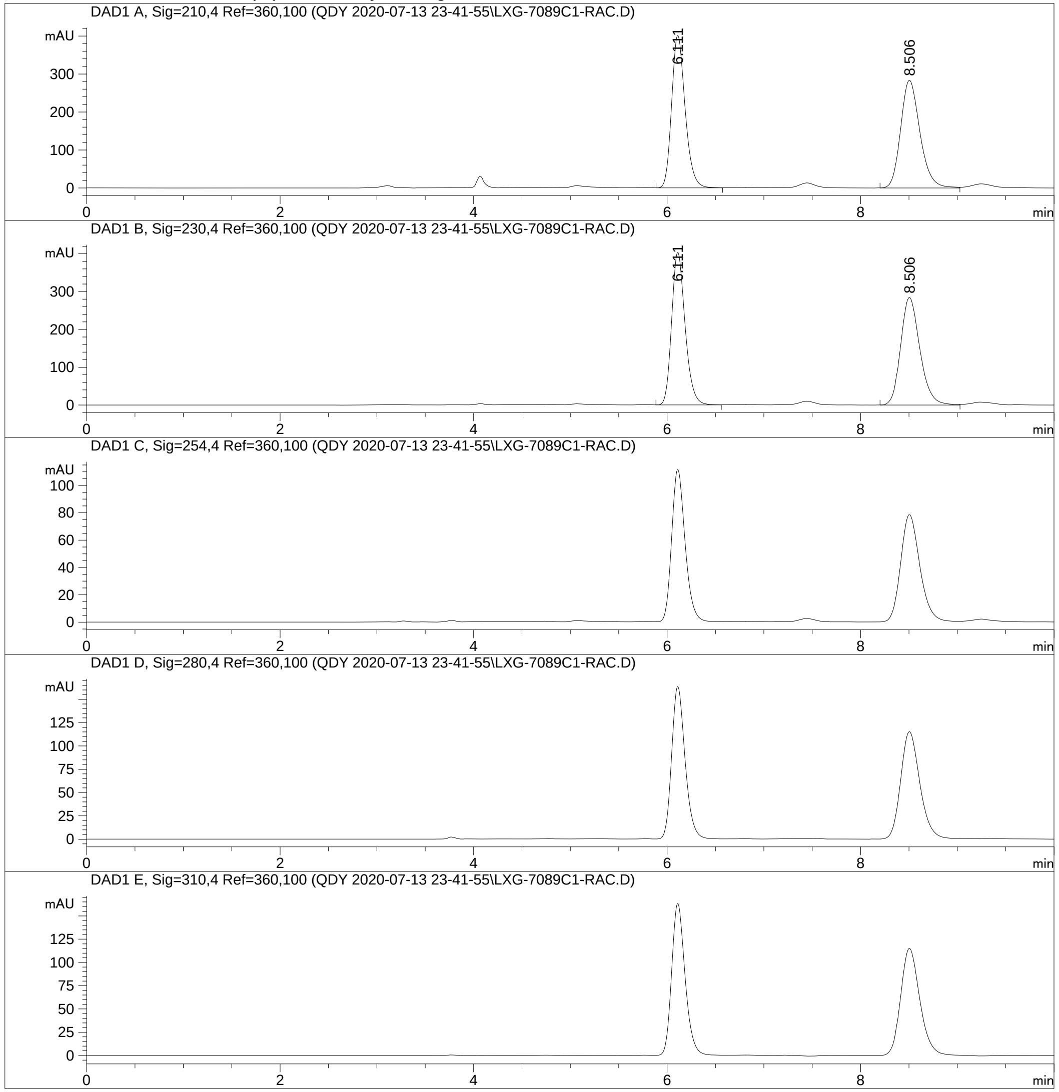

Data File C:\CHEM32\1\DATA\QDY 2020-07-13 23-41-55\LXG-7089C1-RAC.D
Sample Name:

```
=====================================================================
                         Area Percent Report
=====================================================================

Sorted By             :      Signal
Multiplier            :      1.0000
Dilution              :      1.0000
Use Multiplier & Dilution Factor with ISTDs


Signal 1: DAD1 A, Sig=210,4 Ref=360,100

Peak RetTime Type  Width     Area      Height     Area
  #   [min]        [min]   [mAU*s]     [mAU]        %
----|-------|----|-------|----------|----------|--------|
   1   6.111 VB    0.1490 3892.64526  401.89789  49.7598
   2   8.506 BV    0.2137 3930.22095  283.60449  50.2402

Totals :                  7822.86621  685.50238


Signal 2: DAD1 B, Sig=230,4 Ref=360,100

Peak RetTime Type  Width     Area      Height     Area
  #   [min]        [min]   [mAU*s]     [mAU]        %
----|-------|----|-------|----------|----------|--------|
   1   6.111 VB    0.1487 3899.03467  403.65198  49.8315
   2   8.506 BV    0.2130 3925.40625  284.38757  50.1685

Totals :                  7824.44092  688.03955


Signal 3: DAD1 C, Sig=254,4 Ref=360,100


Signal 4: DAD1 D, Sig=280,4 Ref=360,100


Signal 5: DAD1 E, Sig=310,4 Ref=360,100

=====================================================================
                         *** End of Report ***
```

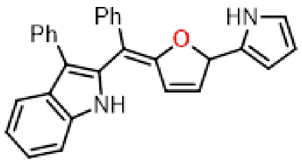

7
(racemic)

Supplementary Figure 206. HPLC spectrum of **7** (racemic)

```
=================================================================
Acq. Operator   :                             Seq. Line :  16
Acq. Instrument : Instrument 1                 Location : Vial 70
Injection Date  : 7/15/2020 9:22:33 PM              Inj :   1
                                             Inj Volume : 5.000 µl
Different Inj Volume from Sequence !     Actual Inj Volume : 4.000 µl
Acq. Method     : C:\CHEM32\1\DATA\QDY 2020-07-15 16-33-43\AD-20-10.M
Last changed    : 2/7/2015 11:42:28 AM
Analysis Method : C:\CHEM32\1\METHODS\AD-008-50.M
Last changed    : 7/10/2020 9:21:13 AM
Additional Info : Peak(s) manually integrated
```

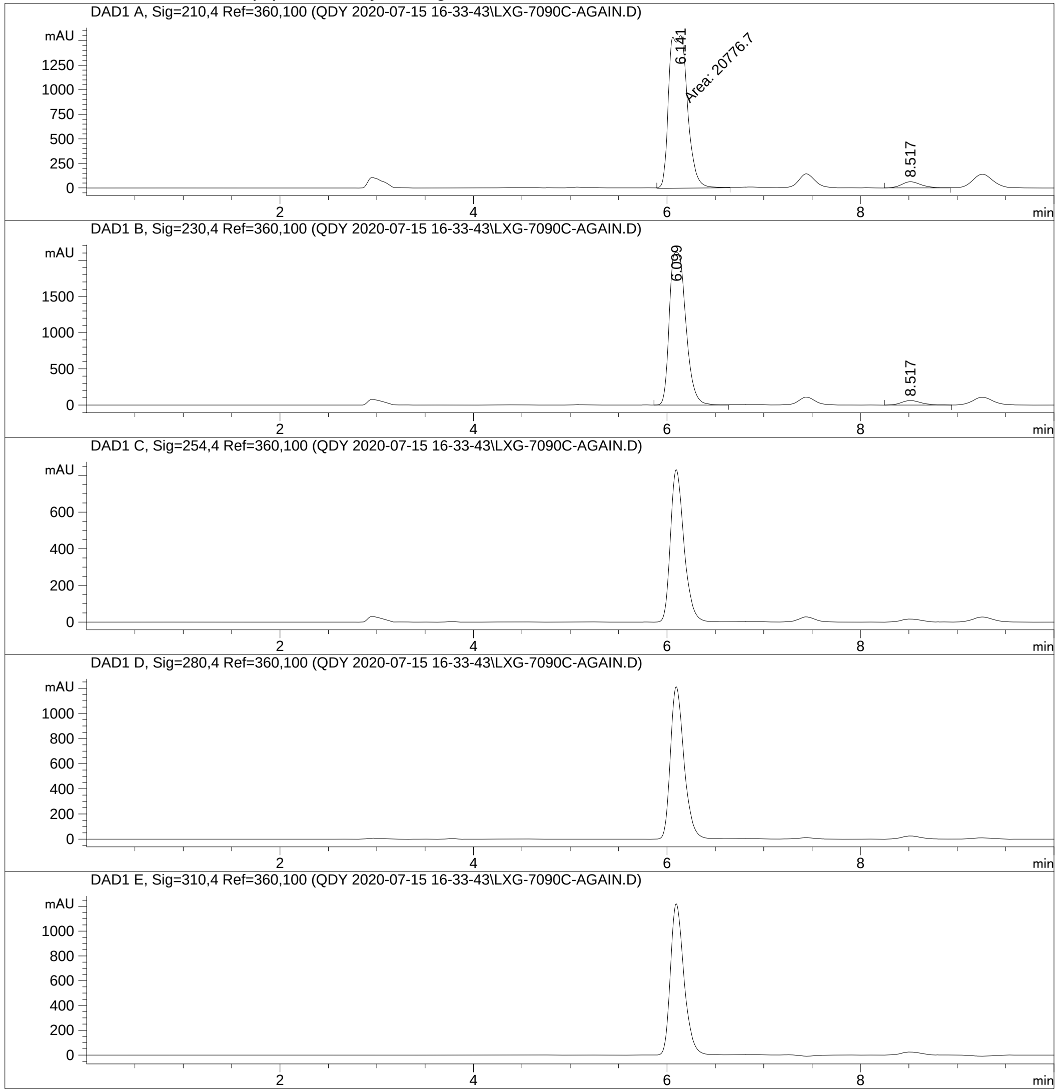

Data File C:\CHEM32\1\DATA\QDY 2020-07-15 16-33-43\LXG-7090C-AGAIN.D
Sample Name:

```
=================================================================
                      Area Percent Report
=================================================================

Sorted By             :      Signal
Multiplier            :      1.0000
Dilution              :      1.0000
Use Multiplier & Dilution Factor with ISTDs


Signal 1: DAD1 A, Sig=210,4 Ref=360,100

Peak RetTime Type  Width     Area      Height     Area
  #   [min]        [min]   [mAU*s]     [mAU]        %
----|-------|----|-------|----------|----------|--------|
   1   6.141 MM    0.2226 2.07767e4  1555.80688  95.8682
   2   8.517 BB    0.2161  895.45807   62.89251   4.1318

Totals :                  2.16722e4  1618.69939


Signal 2: DAD1 B, Sig=230,4 Ref=360,100

Peak RetTime Type  Width     Area      Height     Area
  #   [min]        [min]   [mAU*s]     [mAU]        %
----|-------|----|-------|----------|----------|--------|
   1   6.099 VV    0.1853 2.47872e4  2106.66748  96.5491
   2   8.517 BB    0.2148  885.95630   62.72213   3.4509

Totals :                  2.56731e4  2169.38961


Signal 3: DAD1 C, Sig=254,4 Ref=360,100


Signal 4: DAD1 D, Sig=280,4 Ref=360,100


Signal 5: DAD1 E, Sig=310,4 Ref=360,100

=================================================================
                     *** End of Report ***
```

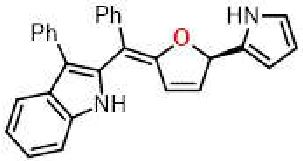

7
(enantioenriched)

Supplementary Figure 207. HPLC spectrum of **7** (enantioenriched)

Data File C:\CHEM32\1\DATA\QDY 2020-07-24 18-22-17\LXG-7098B-RAC.D
Sample Name:

```
=====================================================================
Acq. Operator   :                               Seq. Line :  57
Acq. Instrument : Instrument 1                   Location : Vial 67
Injection Date  : 7/25/2020 2:57:17 PM                Inj :   1
                                               Inj Volume : 5.000 µl
Acq. Method     : C:\CHEM32\1\DATA\QDY 2020-07-24 18-22-17\OD-20-40.M
Last changed    : 7/25/2020 2:56:25 PM
                  (modified after loading)
Analysis Method : C:\CHEM32\1\METHODS\AD-008-50.M
Last changed    : 7/31/2020 7:38:22 PM
                  (modified after loading)
Additional Info : Peak(s) manually integrated
```

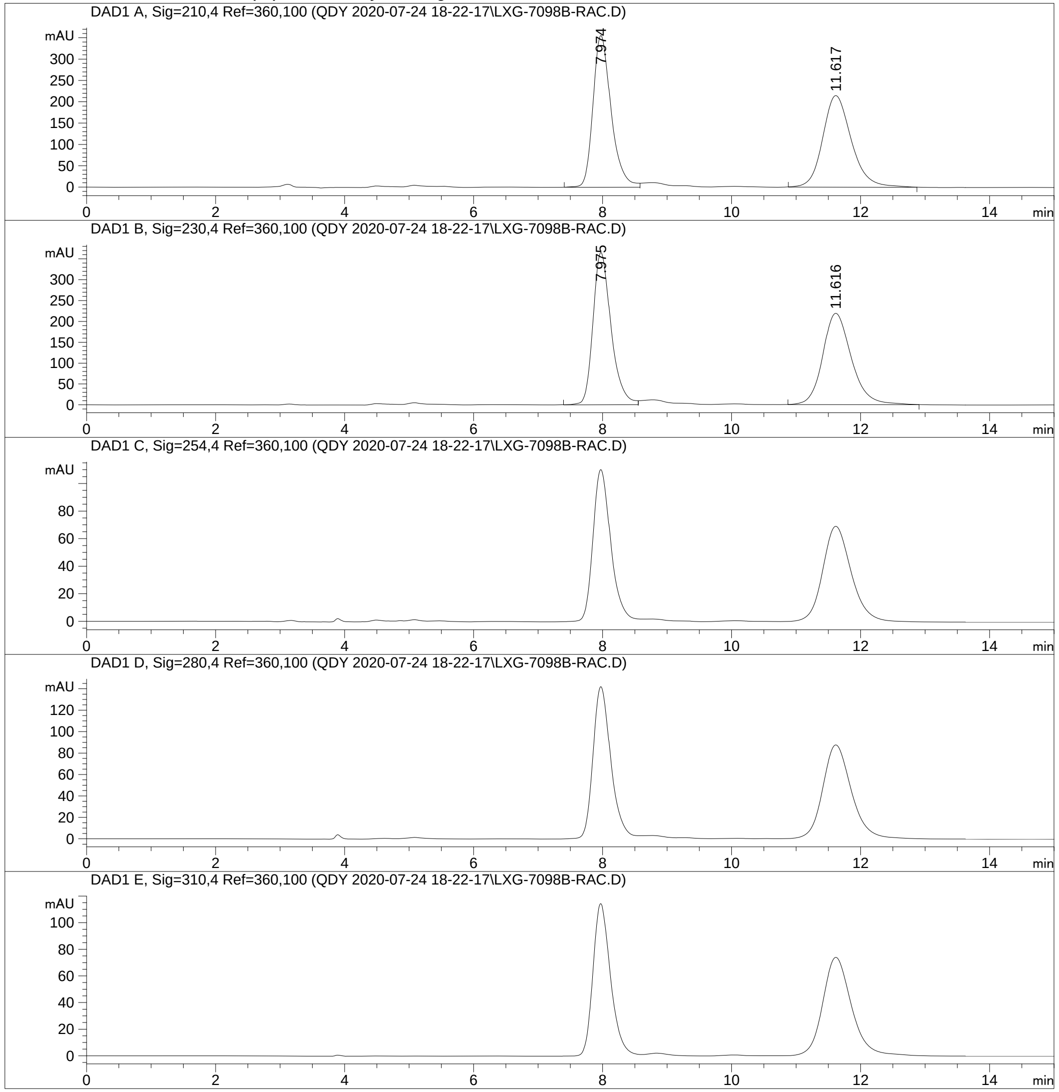

Data File C:\CHEM32\1\DATA\QDY 2020-07-24 18-22-17\LXG-7098B-RAC.D
Sample Name:

```
=====================================================================
                         Area Percent Report
=====================================================================

Sorted By             :      Signal
Multiplier            :      1.0000
Dilution              :      1.0000
Use Multiplier & Dilution Factor with ISTDs
```

Signal 1: DAD1 A, Sig=210,4 Ref=360,100

Peak #	RetTime [min]	Type	Width [min]	Area [mAU*s]	Height [mAU]	Area %
1	7.974	BB	0.2956	6940.48926	355.68835	51.1597
2	11.617	BB	0.4701	6625.82324	214.68097	48.8403

Totals : 1.35663e4 570.36932

Signal 2: DAD1 B, Sig=230,4 Ref=360,100

Peak #	RetTime [min]	Type	Width [min]	Area [mAU*s]	Height [mAU]	Area %
1	7.975	BV	0.2952	7109.08398	365.05750	51.1949
2	11.616	BB	0.4710	6777.24072	219.02634	48.8051

Totals : 1.38863e4 584.08383

Signal 3: DAD1 C, Sig=254,4 Ref=360,100

Signal 4: DAD1 D, Sig=280,4 Ref=360,100

Signal 5: DAD1 E, Sig=310,4 Ref=360,100

```
=====================================================================
                        *** End of Report ***
```

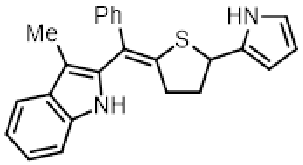

8
(racemic)

Supplementary Figure 208. HPLC spectrum of **8** (racemic)

```
=====================================================================
Acq. Operator   :                              Seq. Line :  11
Acq. Instrument : Instrument 1                  Location : Vial 70
Injection Date  : 7/31/2020 6:11:50 PM               Inj :   1
                                              Inj Volume : 5.000 µl
Different Inj Volume from Sequence !     Actual Inj Volume : 4.000 µl
Acq. Method     : C:\CHEM32\1\DATA\QDY 2020-07-31 14-48-08\OD-20-40.M
Last changed    : 7/31/2020 6:10:55 PM
                  (modified after loading)
Analysis Method : C:\CHEM32\1\METHODS\AD-008-50.M
Last changed    : 7/31/2020 7:38:22 PM
                  (modified after loading)
Additional Info : Peak(s) manually integrated
```

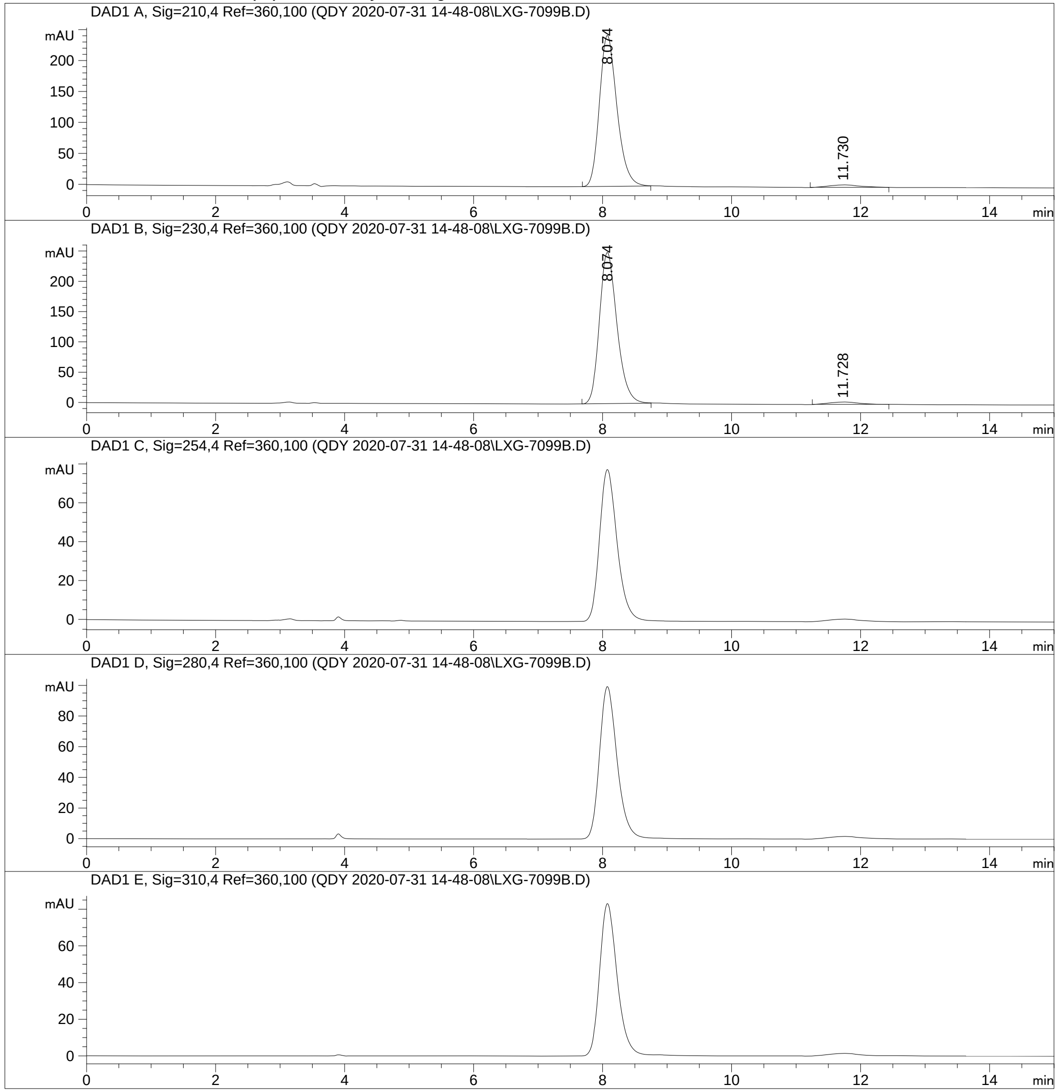

Data File C:\CHEM32\1\DATA\QDY 2020-07-31 14-48-08\LXG-7099B.D
Sample Name:

```
=====================================================================
                        Area Percent Report
=====================================================================

Sorted By            :      Signal
Multiplier           :      1.0000
Dilution             :      1.0000
Use Multiplier & Dilution Factor with ISTDs
```

Signal 1: DAD1 A, Sig=210,4 Ref=360,100

Peak #	RetTime [min]	Type	Width [min]	Area [mAU*s]	Height [mAU]	Area %
1	8.074	BB	0.2905	4613.42578	244.08182	97.3442
2	11.730	BB	0.4262	125.86478	4.19339	2.6558
Totals :				4739.29057	248.27521	

Signal 2: DAD1 B, Sig=230,4 Ref=360,100

Peak #	RetTime [min]	Type	Width [min]	Area [mAU*s]	Height [mAU]	Area %
1	8.074	BB	0.2904	4712.54053	249.45163	94.9553
2	11.728	BB	0.4470	120.56906	4.12544	2.4294
3	18.823	BB	0.6525	129.79692	2.77089	2.6153
Totals :				4962.90651	256.34796	

Signal 3: DAD1 C, Sig=254,4 Ref=360,100

Signal 4: DAD1 D, Sig=280,4 Ref=360,100

Signal 5: DAD1 E, Sig=310,4 Ref=360,100

```
=====================================================================
                      *** End of Report ***
```

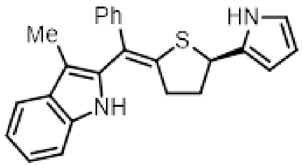

8
(enantioenriched)

Supplementary Figure 209. HPLC spectrum of **8** (enantioenriched)

Data File C:\CHEM32\1\DATA\QDY 2020-07-06 22-26-53\LXG-7078B-RAC.D
Sample Name:

===

Acq. Operator	:	Seq. Line :	9
Acq. Instrument	: Instrument 1	Location :	Vial 65
Injection Date	: 7/7/2020 1:12:53 AM	Inj :	1
		Inj Volume :	5.000 µl

Acq. Method : C:\CHEM32\1\DATA\QDY 2020-07-06 22-26-53\AD-20-40.M
Last changed : 7/7/2020 12:30:52 AM
(modified after loading)
Analysis Method : C:\CHEM32\1\METHODS\AD-008-50.M
Last changed : 7/25/2020 7:11:23 PM
(modified after loading)
Additional Info : Peak(s) manually integrated

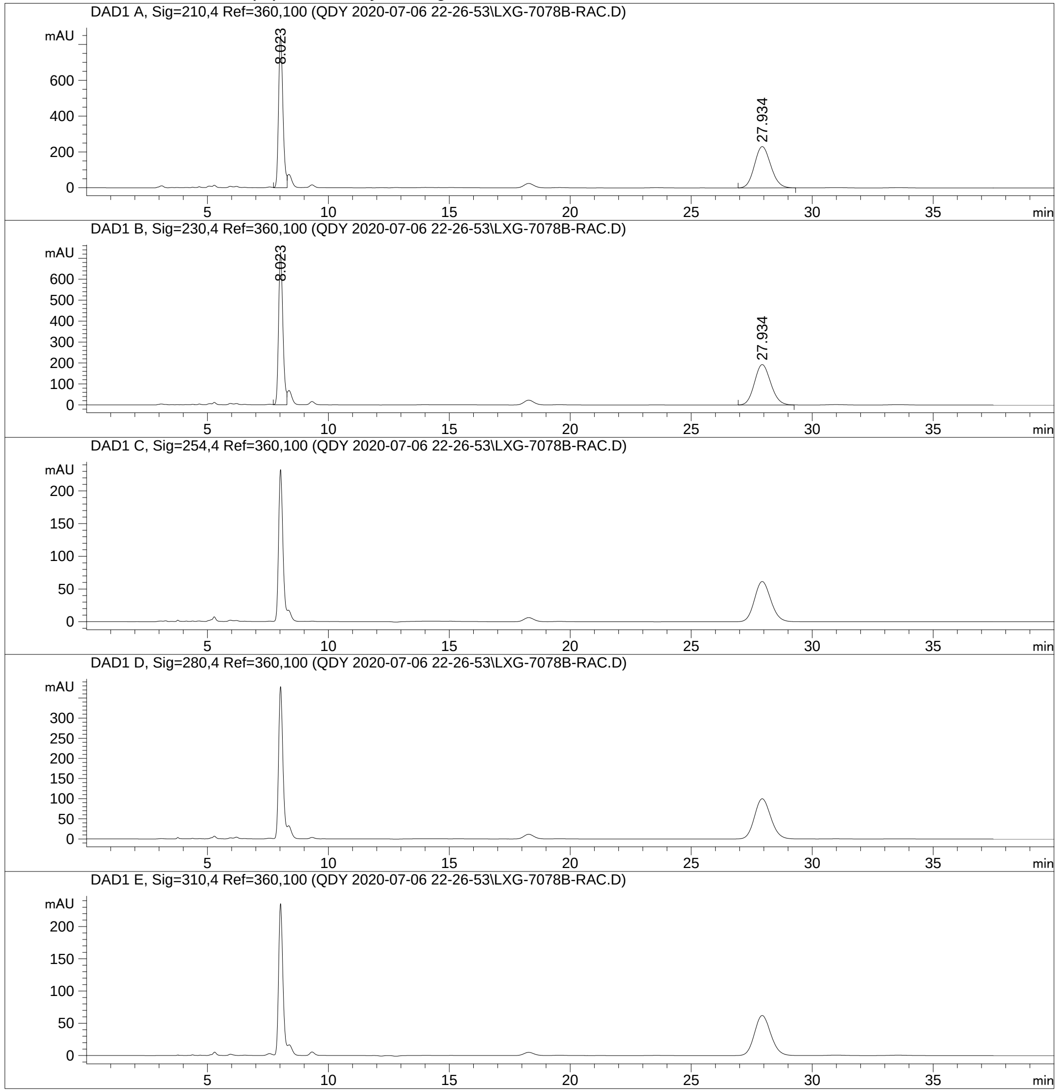

Data File C:\CHEM32\1\DATA\QDY 2020-07-06 22-26-53\LXG-7078B-RAC.D
Sample Name:

===
Area Percent Report
===

Sorted By : Signal
Multiplier : 1.0000
Dilution : 1.0000
Use Multiplier & Dilution Factor with ISTDs

Signal 1: DAD1 A, Sig=210,4 Ref=360,100

Peak #	RetTime [min]	Type	Width [min]	Area [mAU*s]	Height [mAU]	Area %
1	8.023	VV	0.1926	1.06791e4	849.79663	49.6574
2	27.934	BB	0.7291	1.08264e4	230.85995	50.3426

Totals : 2.15055e4 1080.65659

Signal 2: DAD1 B, Sig=230,4 Ref=360,100

Peak #	RetTime [min]	Type	Width [min]	Area [mAU*s]	Height [mAU]	Area %
1	8.023	VV	0.1908	9025.42285	727.09631	49.9839
2	27.934	BB	0.7262	9031.23047	192.90782	50.0161

Totals : 1.80567e4 920.00414

Signal 3: DAD1 C, Sig=254,4 Ref=360,100

Signal 4: DAD1 D, Sig=280,4 Ref=360,100

Signal 5: DAD1 E, Sig=310,4 Ref=360,100

===
*** End of Report ***

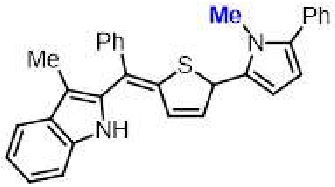

3a'
(racemic)

Supplementary Figure 210. HPLC spectrum of **3a'** (racemic)

```
=====================================================================
Acq. Operator   :                               Seq. Line :  19
Acq. Instrument : Instrument 1                   Location : Vial 67
Injection Date  : 7/18/2020 3:00:13 PM                Inj :   1
                                               Inj Volume : 5.000 µl
Different Inj Volume from Sequence !     Actual Inj Volume : 4.000 µl
Acq. Method     : C:\CHEM32\1\DATA\QDY 2020-07-18 09-42-31\AD-20-40.M
Last changed    : 7/18/2020 2:59:21 PM
                  (modified after loading)
Analysis Method : C:\CHEM32\1\METHODS\AD-008-50.M
Last changed    : 7/25/2020 7:11:23 PM
                  (modified after loading)
Additional Info : Peak(s) manually integrated
```

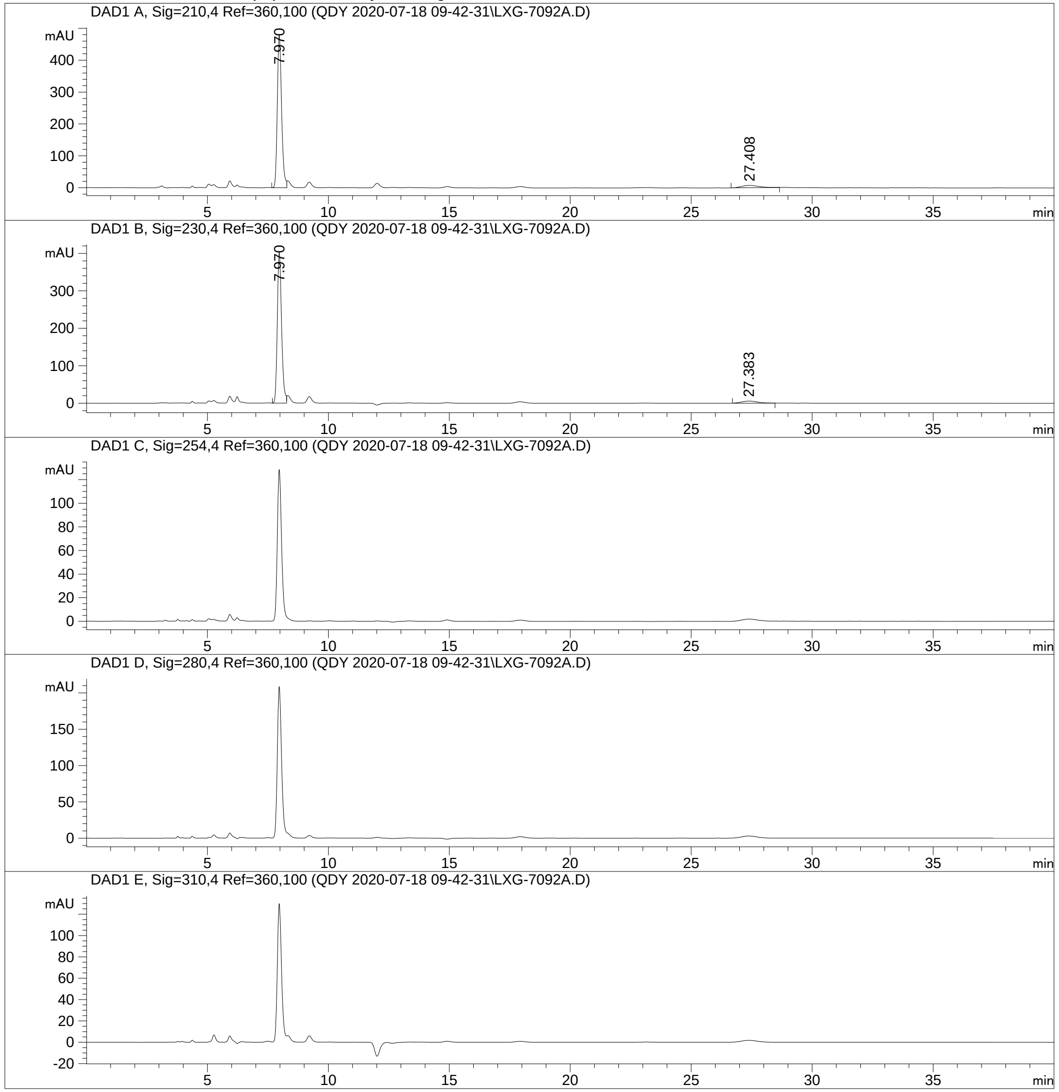

Data File C:\CHEM32\1\DATA\QDY 2020-07-18 09-42-31\LXG-7092A.D
Sample Name:

```
=====================================================================
                         Area Percent Report
=====================================================================

Sorted By             :      Signal
Multiplier            :      1.0000
Dilution              :      1.0000
Use Multiplier & Dilution Factor with ISTDs
```

Signal 1: DAD1 A, Sig=210,4 Ref=360,100

Peak #	RetTime [min]	Type	Width [min]	Area [mAU*s]	Height [mAU]	Area %
1	7.970	VV	0.1897	5888.96143	478.02817	94.2839
2	27.408	BB	0.7033	357.02933	7.29058	5.7161

Totals : 6245.99075 485.31875

Signal 2: DAD1 B, Sig=230,4 Ref=360,100

Peak #	RetTime [min]	Type	Width [min]	Area [mAU*s]	Height [mAU]	Area %
1	7.970	BV	0.1870	4930.58838	402.14981	95.1420
2	27.383	BB	0.6629	251.76088	5.57428	4.8580

Totals : 5182.34926 407.72410

Signal 3: DAD1 C, Sig=254,4 Ref=360,100

Signal 4: DAD1 D, Sig=280,4 Ref=360,100

Signal 5: DAD1 E, Sig=310,4 Ref=360,100

===
*** End of Report ***

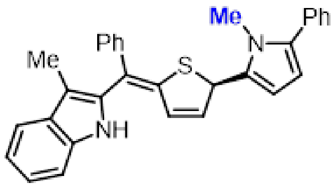

3a'
(enantioenriched)

Supplementary Figure 211 HPLC spectrum of **3a'** (enantioenriched)

Data File C:\CHEM32\1\DATA\QDY 2020-07-06 22-26-53\LXG-7078A-RAC.D
Sample Name:

===

Acq. Operator : Seq. Line : 8
Acq. Instrument : Instrument 1 Location : Vial 64
Injection Date : 7/7/2020 12:31:45 AM Inj : 1
Inj Volume : 5.000 µl
Acq. Method : C:\CHEM32\1\DATA\QDY 2020-07-06 22-26-53\AD-20-40.M
Last changed : 7/7/2020 12:30:52 AM
(modified after loading)
Analysis Method : C:\CHEM32\1\METHODS\AD-008-50.M
Last changed : 7/25/2020 7:11:23 PM
(modified after loading)
Additional Info : Peak(s) manually integrated

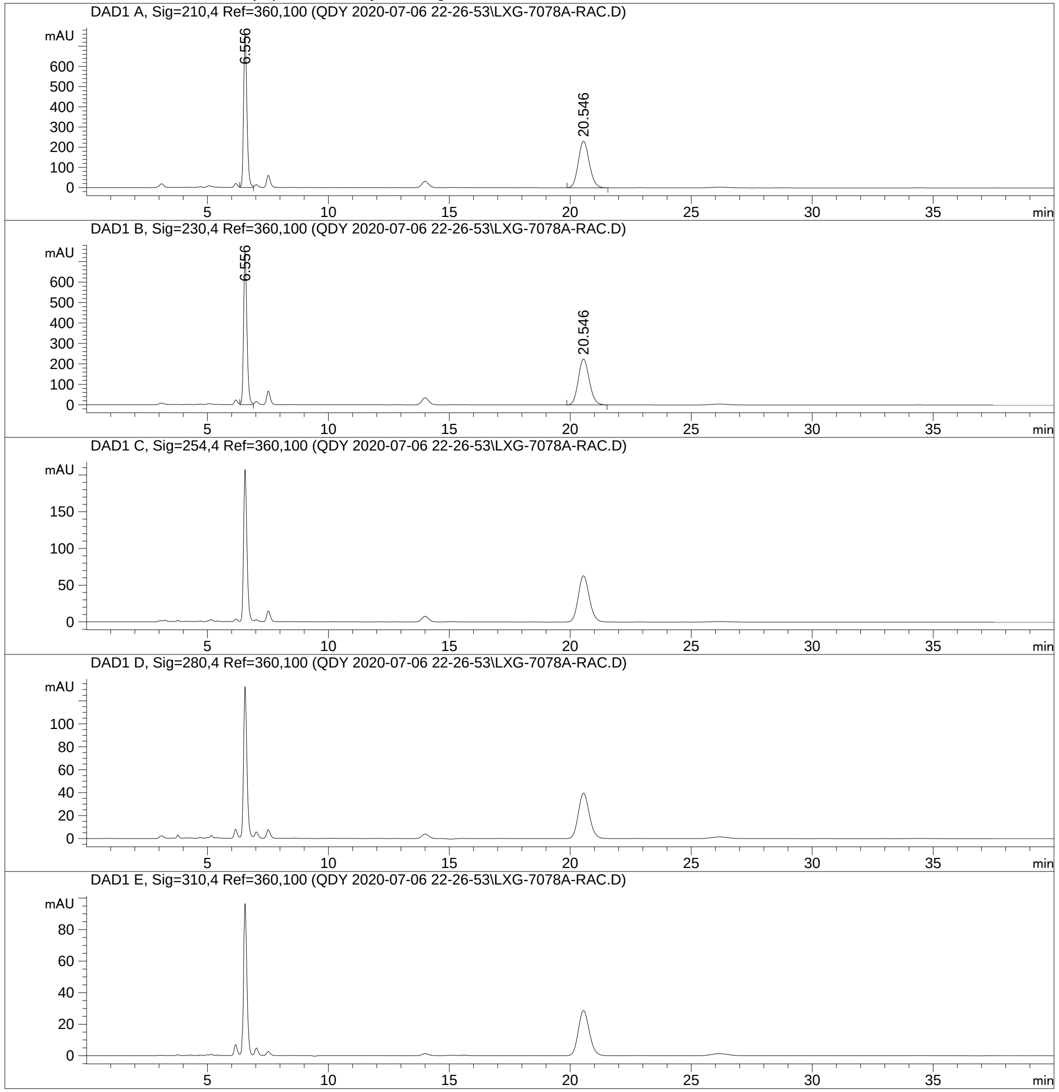

Data File C:\CHEM32\1\DATA\QDY 2020-07-06 22-26-53\LXG-7078A-RAC.D
Sample Name:

===

Area Percent Report

===

Sorted By : Signal
Multiplier : 1.0000
Dilution : 1.0000
Use Multiplier & Dilution Factor with ISTDs

Signal 1: DAD1 A, Sig=210,4 Ref=360,100

Peak #	RetTime [min]	Type	Width [min]	Area [mAU*s]	Height [mAU]	Area %
1	6.556	VV	0.1488	7279.98633	752.79919	49.9059
2	20.546	BB	0.4911	7307.43311	230.95979	50.0941

Totals : 1.45874e4 983.75899

Signal 2: DAD1 B, Sig=230,4 Ref=360,100

Peak #	RetTime [min]	Type	Width [min]	Area [mAU*s]	Height [mAU]	Area %
1	6.556	VV	0.1460	7142.89111	744.14386	50.1425
2	20.546	BB	0.4905	7102.28369	224.84528	49.8575

Totals : 1.42452e4 968.98914

Signal 3: DAD1 C, Sig=254,4 Ref=360,100

Signal 4: DAD1 D, Sig=280,4 Ref=360,100

Signal 5: DAD1 E, Sig=310,4 Ref=360,100

===

*** End of Report ***

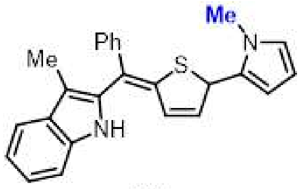

3t'
(racemic)

Supplementary Figure 212. HPLC spectrum of **3t'** (racemic)

Data File C:\CHEM32\1\DATA\QDY 2020-07-18 09-42-31\LXG-7092B.D
Sample Name:

===

Acq. Operator	:	Seq. Line :	20
Acq. Instrument	: Instrument 1	Location :	Vial 68
Injection Date	: 7/18/2020 3:41:19 PM	Inj :	1
		Inj Volume :	5.000 µl

Different Inj Volume from Sequence ! Actual Inj Volume : 4.000 µl
Acq. Method : C:\CHEM32\1\DATA\QDY 2020-07-18 09-42-31\AD-20-40.M
Last changed : 7/18/2020 2:59:21 PM
(modified after loading)
Analysis Method : C:\CHEM32\1\METHODS\AD-008-50.M
Last changed : 7/25/2020 7:11:23 PM
(modified after loading)
Additional Info : Peak(s) manually integrated

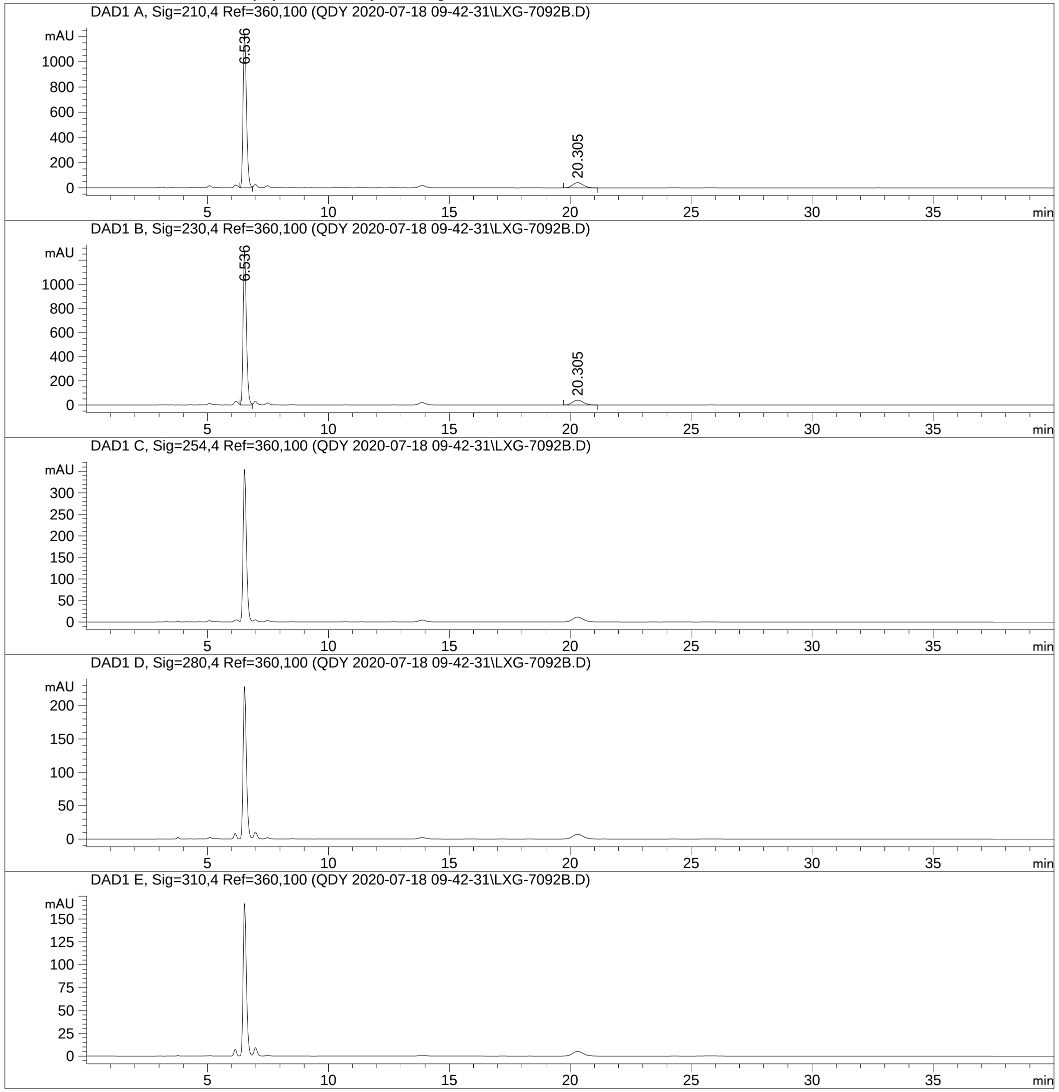

Data File C:\CHEM32\1\DATA\QDY 2020-07-18 09-42-31\LXG-7092B.D
Sample Name:

===
Area Percent Report
===

Sorted By : Signal
Multiplier : 1.0000
Dilution : 1.0000
Use Multiplier & Dilution Factor with ISTDs

Signal 1: DAD1 A, Sig=210,4 Ref=360,100

Peak #	RetTime [min]	Type	Width [min]	Area [mAU*s]	Height [mAU]	Area %
1	6.536	VV	0.1523	1.18542e4	1209.83167	90.2199
2	20.305	BB	0.4813	1285.03748	41.72567	9.7801

Totals : 1.31393e4 1251.55733

Signal 2: DAD1 B, Sig=230,4 Ref=360,100

Peak #	RetTime [min]	Type	Width [min]	Area [mAU*s]	Height [mAU]	Area %
1	6.536	VV	0.1464	1.19689e4	1264.49561	90.6123
2	20.305	BB	0.4761	1240.01331	40.40369	9.3877

Totals : 1.32089e4 1304.89930

Signal 3: DAD1 C, Sig=254,4 Ref=360,100

Signal 4: DAD1 D, Sig=280,4 Ref=360,100

Signal 5: DAD1 E, Sig=310,4 Ref=360,100

===
*** End of Report ***

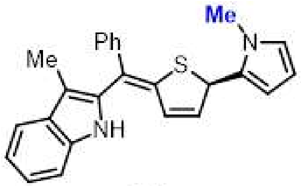

3t'
(enantioenriched)

Supplementary Figure 213. HPLC spectrum of **3t'** (enantioenriched)

Data File C:\CHEM32\1\DATA\QDY 2021-01-26 21-32-58\LXG-7118B-RAC.D
Sample Name:

===

Acq. Operator : Seq. Line : 12
Acq. Instrument : Instrument 1 Location : Vial 32
Injection Date : 1/27/2021 1:36:35 AM Inj : 1
Inj Volume : 5.000 µl
Acq. Method : C:\CHEM32\1\DATA\QDY 2021-01-26 21-32-58\OD-15-40.M
Last changed : 9/24/2015 2:07:59 PM
Analysis Method : C:\CHEM32\1\DATA\QDY 2021-01-24 19-32-15\OD-07-10.M
Last changed : 1/9/2019 4:35:28 PM
Additional Info : Peak(s) manually integrated

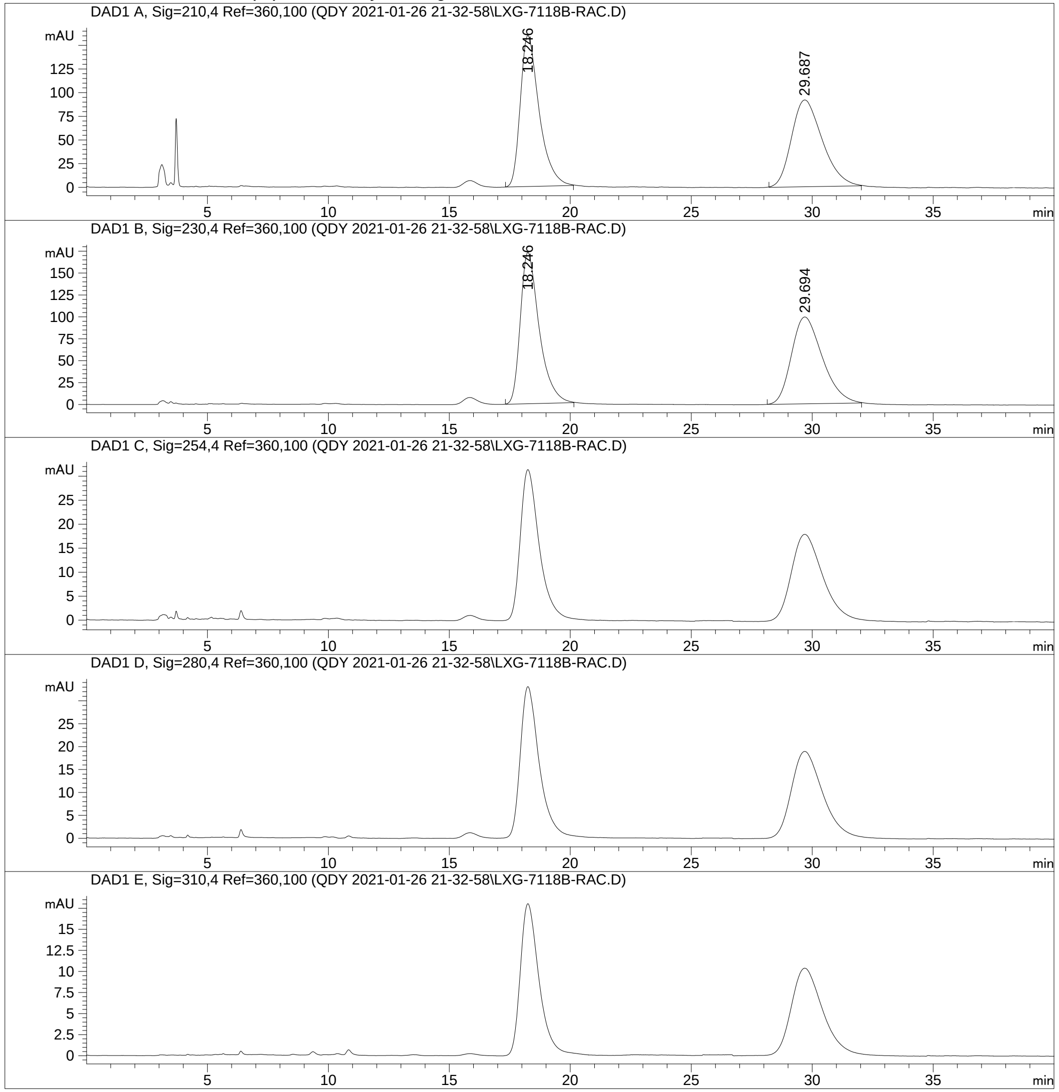

Data File C:\CHEM32\1\DATA\QDY 2021-01-26 21-32-58\LXG-7118B-RAC.D
Sample Name:

===

Area Percent Report

===

Sorted By : Signal
Multiplier : 1.0000
Dilution : 1.0000
Use Multiplier & Dilution Factor with ISTDs

Signal 1: DAD1 A, Sig=210,4 Ref=360,100

Peak #	RetTime [min]	Type	Width [min]	Area [mAU*s]	Height [mAU]	Area %
1	18.246	BB	0.8204	8649.07520	159.56844	51.4866
2	29.687	BB	1.2352	8149.62891	91.66927	48.5134
Totals :				1.67987e4	251.23770	

Signal 2: DAD1 B, Sig=230,4 Ref=360,100

Peak #	RetTime [min]	Type	Width [min]	Area [mAU*s]	Height [mAU]	Area %
1	18.246	BB	0.8235	9369.46582	172.55296	51.5250
2	29.694	BB	1.3633	8814.84668	99.14123	48.4750
Totals :				1.81843e4	271.69419	

Signal 3: DAD1 C, Sig=254,4 Ref=360,100

Signal 4: DAD1 D, Sig=280,4 Ref=360,100

Signal 5: DAD1 E, Sig=310,4 Ref=360,100

===

*** End of Report ***

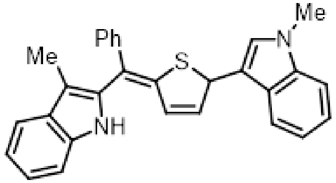

10
(racemic)

Supplementary Figure 214. HPLC spectrum of **10** (racemic)

```
=====================================================================
Acq. Operator   :                              Seq. Line :   2
Acq. Instrument : Instrument 1                  Location : Vial 33
Injection Date  : 1/27/2021 9:23:48 PM               Inj :   1
                                              Inj Volume : 5.000 µl
Different Inj Volume from Sequence !     Actual Inj Volume : 6.000 µl
Acq. Method     : C:\CHEM32\1\DATA\QDY 2021-01-27 21-10-57\OD-15-40.M
Last changed    : 1/27/2021 9:22:55 PM
                  (modified after loading)
Analysis Method : C:\CHEM32\1\DATA\QDY 2021-01-24 19-32-15\OD-07-10.M
Last changed    : 1/9/2019 4:35:28 PM
Additional Info : Peak(s) manually integrated
```

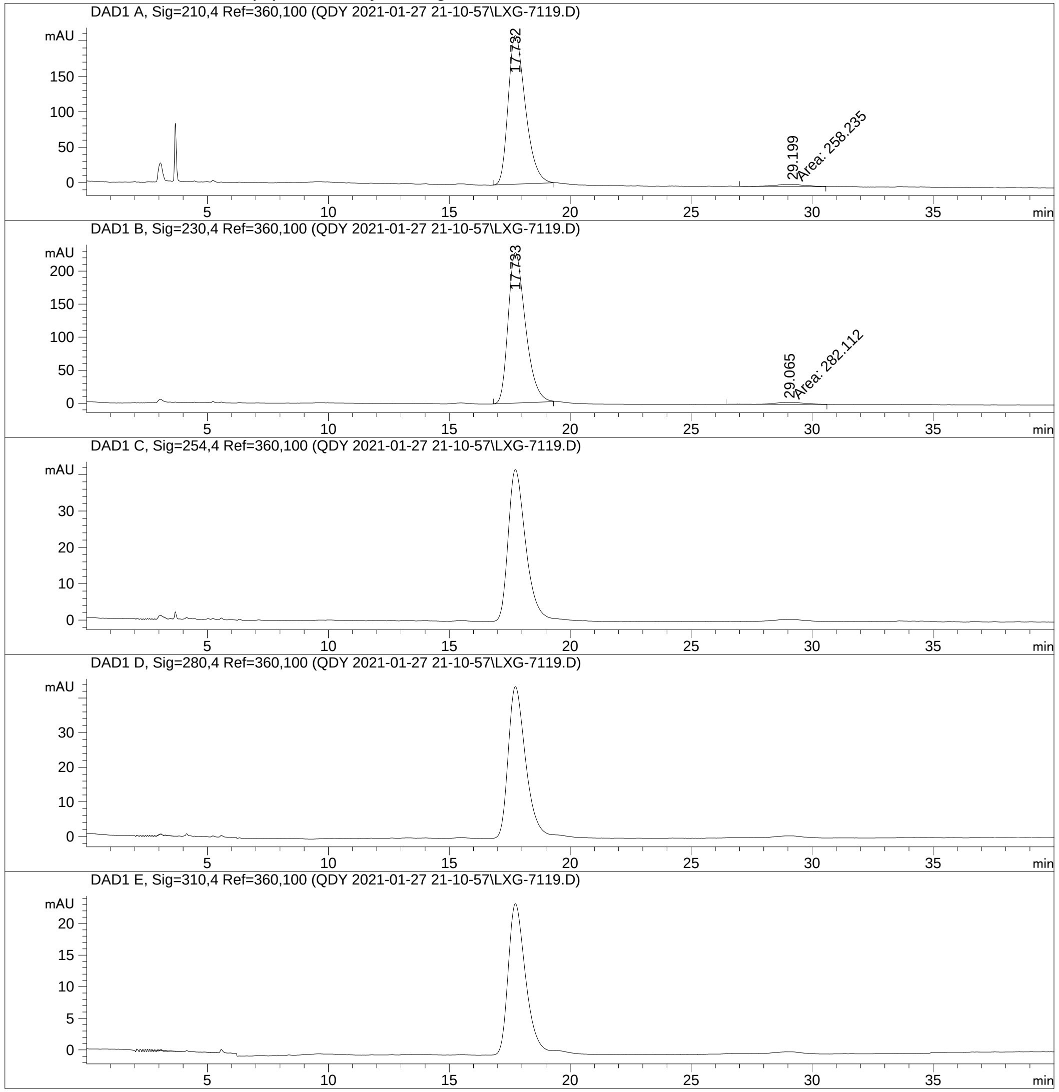

Data File C:\CHEM32\1\DATA\QDY 2021-01-27 21-10-57\LXG-7119.D
Sample Name:

```
=====================================================================
                         Area Percent Report
=====================================================================

Sorted By             :      Signal
Multiplier            :      1.0000
Dilution              :      1.0000
Use Multiplier & Dilution Factor with ISTDs
```

Signal 1: DAD1 A, Sig=210,4 Ref=360,100

Peak #	RetTime [min]	Type	Width [min]	Area [mAU*s]	Height [mAU]	Area %
1	17.732	BB	0.7599	1.03510e4	209.69389	97.5659
2	29.199	MM	1.4392	258.23492	2.99052	2.4341

Totals : 1.06093e4 212.68441

Signal 2: DAD1 B, Sig=230,4 Ref=360,100

Peak #	RetTime [min]	Type	Width [min]	Area [mAU*s]	Height [mAU]	Area %
1	17.733	BB	0.7647	1.12584e4	227.75662	97.5555
2	29.065	MM	1.5051	282.11191	3.12401	2.4445

Totals : 1.15405e4 230.88064

Signal 3: DAD1 C, Sig=254,4 Ref=360,100

Signal 4: DAD1 D, Sig=280,4 Ref=360,100

Signal 5: DAD1 E, Sig=310,4 Ref=360,100

```
=====================================================================
                         *** End of Report ***
```

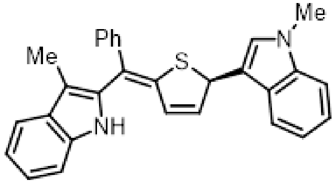

10
(enantioenriched)

Supplementary Figure 215. HPLC spectrum of **10** (enantioenriched)
